# Supplementary material for: Alkyne–Alkene [2 + 2] cycloaddition based on visible light photocatalysis
Source: Nat Commun. 2020 May 19;11:2509. doi: 10.1038/s41467-020-16283-9 (PMC7237675; doi:10.1038/s41467-020-16283-9)
Supplement: Supplementary file 1 — Supplementary Information [file 41467_2020_16283_MOESM1_ESM.pdf]

Supplementary Information

Alkyne-Alkene [2+2] Cycloaddition based on Visible Light Photocatalysis

Ha *et al*

Supplementary Methods

General Information

All reactions were conducted under a nitrogen atmosphere with oven-dried glassware and standard Schlenk or vacuum line techniques. All solutions were handled under nitrogen or argon and transferred via syringe. Anhydrous solvents were purchased and stored over activated 4 Å molecular sieves. Unless otherwise stated, reagents were commercially available and used as purchased without further purification. Chemicals were purchased from Sigma-Aldrich, Acros, Alfa Aesar or TCI. Photocatalysts PC I[1], PC II[2], PC III - VI[1] and $Ru(bpy)_3PF_6$[3] were prepared according to literature procedures. Progress of reactions was monitored by thin-layer chromatography (TLC) using Merck 60 F254 precoated silica gel plate and visualized by short-wave ultraviolet light as well as by treatment with basic solution of potassium permanganate or acidic solution of ceric molybdate. Flash chromatography was performed with Silica Flash P60 silica gel (230 – 400 mesh). ^{1}H and ^{13}C NMR spectra were obtained using an Agilent 400-MR DD2 Fourier-transform NMR spectrometer at 400 and 100 MHz, respectively. Chemical shifts were reported in units of parts per million (ppm) downfield from tetramethylsilane (TMS), and all coupling constants were reported in hertz. The residual solvent signals were taken as the reference ($CDCl_3$ 7.26 ppm, DMSO-d_6 2.50 ppm and CD_3OD 3.31 ppm for ^{1}H NMR spectra and $CDCl_3$ 77.0 ppm, DMSO-d_6, 39.52 ppm, CD_3OD 49.00 ppm and CD_2Cl_2 53.84 ppm for ^{13}C NMR spectra). The signals observed are described as: s (singlet), d (doublet), t (triplet), q (quartet), m (multiplet), br (broad signal). Mass analysis were carried out using Advion Expression CMS mass spectrometer and LC-ELSD analysis was carried out using Agilent 1260 Infinity ELSD coupled with Agilent 1220 HPLC. High resolution mass analysis was performed with JOEL AccuTOF 4G+ DART-HRMS and on Bruker, 1200 Series & HCT Basic System. Voltammetric measurements were proceeded using a Potentiostat (WizECM – 1200 Premium). Luminescence quenching studies were performed with Varian Cary Eclipse.

Optimization of reaction conditions

Optimization procedure : Di(*p*-tolyl)acetylene **1a** (0.05 mmol, 1.0 equiv.), *N*-methylmaleimide **2a** (1.5 equiv.), and photocatalyst Ir[dF(CF_3)ppy]$_2$(dtbbpy)PF_6 (**PC I**) were added to an oven-dried 4 mL vial equipped with a stir bar. The combined materials were dissolved in solvent under argon atmosphere in glovebox. The reaction mixture was then irradiated by 12 W blue LED strip at room temperature (maintained with a cooling fan). After completion of the reaction as indicated by TLC, the solution was concentrated under reduced pressure. The yield was determined by ^{1}H NMR analysis ($CDCl_3$) of the crude reaction mixture using trichloroethylene as the internal standard.

Supplementary Table 1. Optimization of reaction condition for cyclobutene

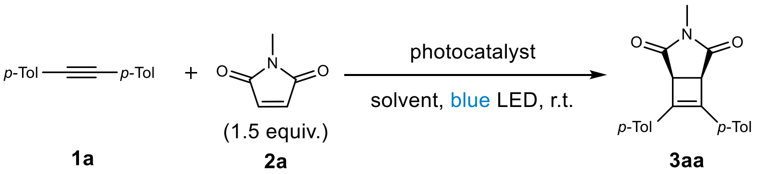

Entry	Catalyst [mol%]	Solvent	Concentration	Yield
1	PC I [5]	CH_2Cl_2	0.1 M	76%
2	PC I [2.5]	CH_2Cl_2	0.1 M	74%
3	PC I [2.5]	$CHCl_3$	0.1 M	73%
4	PC I [2.5]	THF	0.1 M	15%
5	PC I [2.5]	DMF	0.1 M	53%
6	PC I [2.5]	acetone	0.1 M	58%
7	PC I [2.5]	CH_2Cl_2	0.05 M	83% (76%[a])
8	PC I [1]	CH_2Cl_2	0.05 M	75%
9	PC I [2.5]	CH_2Cl_2	0.02 M	80%
10[b]	PC I [2.5]	CH_2Cl_2	0.05 M	48%
11	-	CH_2Cl_2	0.05 M	trace
12[c]	PC I [2.5]	CH_2Cl_2	0.05 M	n.r.

[a] Isolated yield after 4 h [b] Reaction was conducted under air [c] Reaction was conducted in the dark

General procedure and characterization of cyclobutenes

General procedure A (for the synthesis of cyclobutenes)

Alkyne (0.1 mmol, 1.0 equiv.), alkene (1.5 equiv.), and photocatalyst Ir[dF(CF_3)ppy]$_2$(dtbbpy)PF_6 (2.5 mol%) were added to an oven-dried 4 mL vial equipped with a stir bar. The combined materials were dissolved in CH_2Cl_2 (2 mL) under argon atmosphere in glovebox. The reaction mixture was then irradiated by 12 W blue LED strip at room temperature (maintained with a cooling fan). After completion of the reaction as indicated by TLC, the solution was concentrated under reduced pressure. The residue was purified by flash column chromatography on silica gel to give the desired product.

3-methyl-6,7-di-*p*-tolyl-3-azabicyclo[3.2.0]hept-6-ene-2,4-dione (3aa)

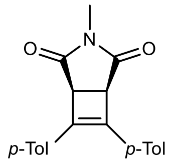

Prepared according to the ***General Procedure A*** using di(*p*-tolyl)acetylene **1a** (0.1 mmol, 1.0 equiv.) and *N*-methylmaleimide **2a** (1.5 equiv.), 24 mg, 76% yield; white solid; m.p. 134 – 135 °C; ^{1}H NMR (400 MHz, $CDCl_3$) δ 7.64 (d, J = 8.2 Hz, 4H), 7.18 (d, J = 7.9 Hz, 4H), 4.06 (s, 2H), 2.97 (s, 3H), 2.37 (s, 6H); ^{13}C NMR (100 MHz, $CDCl_3$) δ 175.2, 139.2, 138.3, 130.3, 129.3, 126.8, 44.9, 24.8, 21.4; HRMS *m/z* calculated for $[C_{21}H_{20}NO_2]^+$ ($[M+H]^+$): 318.1489, observed : 318.1490

3-methyl-6,7-di-*o*-tolyl-3-azabicyclo[3.2.0]hept-6-ene-2,4-dione (3ba)

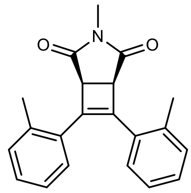

Prepared according to the ***General Procedure A*** using 1,2-di-*o*-tolylethyne **1b** (0.1 mmol, 1.0 equiv.) and *N*-methylmaleimide **2a** (1.5 equiv.), 30 mg, 95% yield; white solid; m.p. 202 – 203 °C; ^{1}H NMR (400 MHz, $CDCl_3$) δ 7.46 (dd, J = 7.2, 1.7 Hz, 2H), 7.25 – 7.12 (m, 6H), 4.16 (s, 2H), 3.02 (s, 3H), 2.01 (s, 6H); ^{13}C NMR (100 MHz, $CDCl_3$) δ 175.2, 141.9, 136.4, 133.7, 130.8, 129.0, 128.5, 126.0, 47.1, 25.1, 20.9; HRMS *m/z* calculated for $[C_{21}H_{20}NO_2]^+$ ($[M+H]^+$): 318.1489, observed : 318.1491

3-methyl-6,7-diphenyl-3-azabicyclo[3.2.0]hept-6-ene-2,4-dione (3ca)

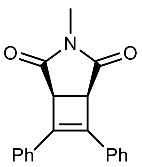

Prepared according to the ***General Procedure A*** using 1,2-diphenylethyne **1c** (0.1 mmol, 1.0 equiv.) and *N*-methylmaleimide **2a** (1.5 equiv.), 18 mg, 62% yield; white solid; m.p. 129 – 130 °C; ^{1}H NMR (400 MHz, $CDCl_3$) δ 7.76 – 7.73 (m, 4H), 7.41 – 7.31 (m, 6H), 4.10 (s, 2H), 2.98 (s, 3H) ^{13}C NMR (100 MHz, $CDCl_3$) δ 175.1, 139.4, 133.0, 129.4, 128.8, 127.0, 45.2, 25.0; HRMS *m/z* calculated for $[C_{19}H_{16}NO_2]^+$ ($[M+H]^+$): 290.1176, observed : 290.1183

6,7-bis(4-methoxyphenyl)-3-methyl-3-azabicyclo[3.2.0]hept-6-ene-2,4-dione (3da)

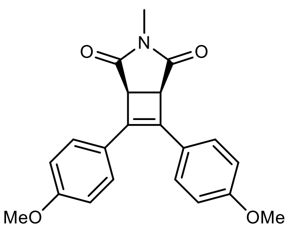

Prepared according to the ***General Procedure A*** using 1,2-bis(4-methoxyphenyl)ethyne **1d** (0.1 mmol, 1.0 equiv.) and *N*-methylmaleimide **2a** (1.5 equiv.), 19 mg, 54% yield; white solid; m.p. 131 – 132 °C; ^{1}H NMR (400 MHz, $CDCl_3$) δ 7.69 (d, *J* = 8.9 Hz, 4H), 6.90 (d, *J* = 8.9 Hz, 4H), 4.04 (s, 2H), 3.83 (s, 6H), 2.97 (s, 3H); ^{13}C NMR (100 MHz, $CDCl_3$) δ 175.6, 160.1, 136.7, 128.4, 126.1, 114.2, 55.5, 45.0, 24.9; HRMS *m/z* calculated for $[C_{21}H_{20}NO_4]^+$ ($[M+H]^+$): 350.1387, observed : 350.1389

6,7-bis(2-methoxyphenyl)-3-methyl-3-azabicyclo[3.2.0]hept-6-ene-2,4-dione (3ea)

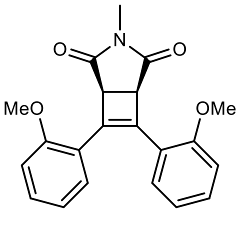

Prepared according to the ***General Procedure A*** using 1,2-bis(2-methoxyphenyl)ethyne **1e** (0.1 mmol, 1.0 equiv.) and *N*-methylmaleimide **2a** (1.5 equiv.), 17 mg, 49% yield; white solid; m.p. 178 – 179 °C; ^{1}H NMR (400 MHz, $CDCl_3$) δ 7.48 (dd, *J* = 7.6, 1.7 Hz, 2H), 7.27 (ddd, *J* = 8.3, 7.4, 1.7 Hz, 2H), 6.93 (td, *J* = 7.5, 1.0 Hz, 2H), 6.83 (d, *J* = 8.3 Hz, 2H), 4.19 (s, 2H), 3.53 (s, 6H), 2.98 (s, 3H); ^{13}C NMR (100 MHz, $CDCl_3$) δ 175.7, 157.3, 138.0,

129.9, 129.5, 123.7, 120.0, 110.3, 54.9, 46.9, 24.9; HRMS *m/z* calculated for $[C_{21}H_{20}NO_4]^+$ ($[M+H]^+$): 350.1387 , observed : 350.1387

Dimethyl 4,4'-(3-methyl-2,4-dioxo-3-azabicyclo[3.2.0]hept-6-ene-6,7-diyl)dibenzoate (3fa)

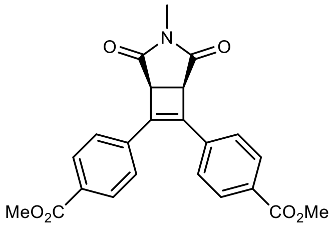

Prepared according to the ***General Procedure A*** using dimethyl 4,4'-(ethyne-1,2-diyl)dibenzoate **1f** (0.1 mmol, 1.0 equiv.) and *N*-methylmaleimide **2a** (1.5 equiv.), 30 mg, 74% yield; white solid; m.p. 207 – 209 °C; ^{1}H NMR (400 MHz, $CDCl_3$) δ 8.06 (d, *J* = 8.7 Hz, 4H), 7.80 (d, *J* = 8.7 Hz, 4H), 4.17 (s, 2H), 3.94 (s, 6H), 3.00 (s, 3H); ^{13}C NMR (100 MHz, $CDCl_3$) δ 174.4, 166.5, 140.7, 136.6, 130.9, 130.2, 127.1, 52.4, 45.4, 25.2; HRMS *m/z* calculated for $[C_{23}H_{20}NO_6]^+$ ($[M+H]^+$): 406.1285, observed : 406.1297

6-(2-bromophenyl)-3-methyl-7-phenyl-3-azabicyclo[3.2.0]hept-6-ene-2,4-dione (3ga)

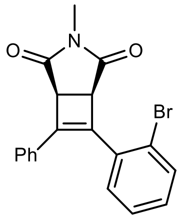

Prepared according to the ***General Procedure A*** using 1-bromo-2-(phenylethynyl)benzene **1g** (0.1 mmol, 1.0 equiv.) and *N*-methylmaleimide **2a** (1.5 equiv.), 26 mg, 71% yield; white solid; m.p. 150 – 151 °C; ^{1}H NMR (400 MHz, $CDCl_3$) δ 7.65 (d, *J* = 8.0 Hz, 1H), 7.48 – 7.46 (m, 2H), 7.38 – 7.23 (m, 6H), 4.30 (d, *J* = 3.4 Hz, 1H), 4.18 (d, *J* = 3.4 Hz, 1H), 3.00 (s, 3H); ^{13}C NMR (100 MHz, $CDCl_3$) δ 174.8, 174.6, 143.2, 138.7, 134.6, 133.4, 132.1, 130.5, 130.2, 129.5, 128.7, 127.8, 127.0, 122.6, 47.4, 45.2, 25.0; HRMS *m/z* calculated for $[C_{19}H_{15}BrNO_2]^+$ ($[M+H]^+$): 368.0281, observed : 368.0281

3-methyl-6-phenyl-7-(2-((trifluoromethoxy)amino)phenyl)-3-azabicyclo[3.2.0]hept-6-ene-2,4-dione (3ha)

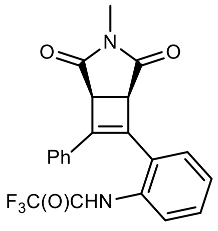

Prepared according to the ***General Procedure A*** using 2,2,2-trifluoro-*N*-(2-(phenylethynyl)phenyl)acetamide **1h** (0.1 mmol, 1.0 equiv.) and *N*-methylmaleimide **2a** (1.5 equiv.), 35 mg, 88% yield; yellow viscous oil; ^{1}H NMR (400 MHz, $CDCl_3$) 8.38 (s, 1H), 7.93 (d, *J* = 8.2 Hz, 1H), 7.78 (d, *J* = 7.8 Hz, 1H), 7.51 – 7.41 (m, 3H), 7.40 – 7.35 (m, 3H), 7.32 (t, *J* = 7.6 Hz, 1H), 4.21 (d, *J* = 3.5 Hz, 1H), 4.15 (d, *J* = 3.5 Hz, 1H), 3.02 (s, 3H); ^{13}C NMR (100 MHz, $CDCl_3$) δ175.3, 174.0, 143.0, 134.6, 131.7, 131.5, 130.31, 130.26, 129.0, 128.7, 127.0, 125.91, 125.90, 124.2, 46.2, 45.6, 25.1; HRMS *m/z* calculated for $[C_{21}H_{15}F_3N_2NaO_3]^+$ ($[M+Na]^+$): 423.0927, observed : 423.0932.

3-methyl-6-(pyridin-2-yl)-7-(*p*-tolyl)-3-azabicyclo[3.2.0]hept-6-ene-2,4-dione (3ia)

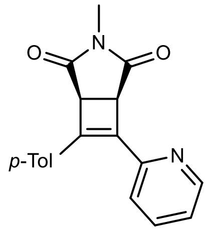

Prepared according to the ***General Procedure A*** using 2-(*p*-tolylethynyl)pyridine **1i** (0.1 mmol, 1.0 equiv.) and *N*-methylmaleimide **2a** (1.5 equiv.), 16 mg, 53% yield; white solid; m.p. 171 – 173 °C; ^{1}H NMR (400 MHz, $CDCl_3$) δ 8.73 (ddd, *J* = 4.8, 1.8, 1.0 Hz, 1H), 8.42 (d, *J* = 8.3 Hz, 2H), 7.83 (dt, *J* = 7.9, 1.1 Hz, 1H), 7.80 – 7.75 (m, 1H), 7.26 (d, *J* = 8.0 Hz, 2H), 7.23 (ddd, *J* = 7.4, 4.8, 1.3 Hz, 1H), 4.19 (d, *J* = 3.7 Hz, 1H), 4.15 (d, *J* = 3.7 Hz, 1H), 2.97 (s, 3H), 2.40 (s, 3H); ^{13}C NMR (100 MHz, $CDCl_3$) δ 175.2, 174.8, 151.7, 149.4, 143.7, 140.3, 136.6, 136.2, 129.7, 129.1, 122.9, 122.7, 44.6, 43.9, 24.8, 21.6; HRMS *m/z* calculated for $[C_{19}H_{17}N_2O_2]^+$ ($[M+H]^+$): 305.1285, observed : 305.1298

3-methyl-6,7-di(pyridin-2-yl)-3-azabicyclo[3.2.0]hept-6-ene-2,4-dione (**3ja**)

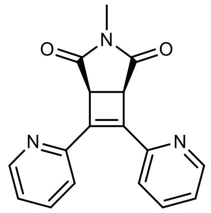

Prepared according to the ***General Procedure A*** using 1,2-di(pyridin-2-yl)ethyne **1j** (0.1 mmol, 1.0 equiv.) and *N*-methylmaleimide **2a** (1.5 equiv.), 15 mg, 51% yield; pale yellow solid; m.p. 212 – 214 °C; ^{1}H NMR (400 MHz, $CDCl_3$) δ 8.77 – 8.76 (m, 2H), 8.61 (d, *J* = 7.9 Hz, 2H), 7.80 (td, *J* = 7.8, 1.9 Hz, 2H), 7.29 – 7.26 (m, 2H), 4.33 (s, 2H), 2.97 (s, 3H); ^{13}C NMR (100 MHz, $CDCl_3$) δ 174.6, 151.0, 149.7, 142.2, 136.6, 124.8, 123.7, 44.4, 25.1; HRMS *m/z* calculated for $[C_{17}H_{13}N_3NaO_2]^+$ ($[M+Na]^+$): 314.0900 , observed : 314.0899

3-methyl-6-(pyrazin-2-yl)-7-(pyridin-2-yl)-3-azabicyclo[3.2.0]hept-6-ene-2,4-dione (3ka)

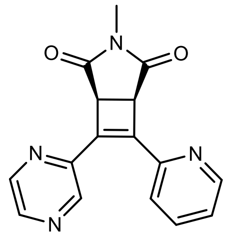

Prepared according to the ***General Procedure A*** using 2-(pyridin-2-ylethynyl)pyrazine **1k** (0.1 mmol, 1.0 equiv.) and *N*-methylmaleimide **2a** (1.5 equiv.), 15 mg, 51% yield; pale yellow solid; m.p. 222 – 223 °C; ^{1}H NMR (400 MHz, $CDCl_3$) δ 10.23 (s, 1H), 8.79– 8.78 (m, 1H), 8.73 (m, 1H), 8.55– 8.53 (m, 1H), 8.19 (d, *J* = 7.9 Hz, 1H), 7.84 (td, *J* = 7.8, 1.8 Hz, 1H), 7.32 (ddd, 7.6, 4.8. 1.1 Hz, 1H), 4.41 (d, *J* = 3.6 Hz, 1H), 4.30 (d, *J* = 3.6 Hz, 1H), 2.99 (s, 3H); ^{13}C NMR (100 MHz, $CDCl_3$) δ 174.2, 173.9, 150.6, 150.0, 147.0, 144.1, 143.9, 137.0, 132.2, 128.7, 124.4, 124.3, 121.6, 44.8, 44.1, 25.2; HRMS *m/z* calculated for $[C_{16}H_{13}N_4O_2]^+$ ($[M+H]^+$): 293.1033, observed : 293.1033

6-cyclopropyl-3-methyl-7-(*p*-tolyl)-3-azabicyclo[3.2.0]hept-6-ene-2,4-dione (3la)

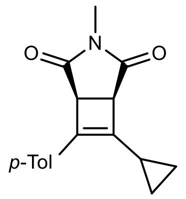

Prepared according to the ***General Procedure A*** using 1-(cyclopropylethynyl)-4-methylbenzene **1l** (0.1 mmol, 1.0 equiv.) and *N*-methylmaleimide **2a** (1.5 equiv.), 18 mg, 67% yield; white solid; m.p. 98 – 100 °C; ^{1}H NMR (400 MHz, $CDCl_3$) δ 7.53 (d, *J* = 8.2 Hz, 2H), 7.18 (d, *J* = 8.3 Hz, 2H), 3.89 (d, *J* = 3.5, 1H), 3.50 (d, *J* = 3.5 Hz, 1H), 2.94 (s, 3H), 2.35 (s, 3H), 1.96 – 1.89 (m, 1H), 1.16 – 1.10 (m, 1H), 1.00 – 0.80 (m, 3H) ^{13}C NMR (100 MHz, $CDCl_3$) δ 175.7, 175.5, 143.1, 138.8, 138.2, 130.5, 129.5, 126.3, 43.8, 24.9, 21.5, 11.4, 6.8, 6.5; HRMS *m/z* calculated for $[C_{17}H_{18}NO_2]^+$ ($[M+H]^+$): 268.1332, observed : 268.1334

6,7-bis(((4-chlorobenzyl)oxy)methyl)-3-methyl-3-azabicyclo[3.2.0]hept-6-ene-2,4-dione (3ma)

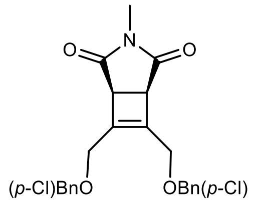

Prepared according to the ***General Procedure A*** using 1,4-bis((4-chlorobenzyl)oxy)but-2-yne **1m** (0.1 mmol, 1.0 equiv.) and *N*-methylmaleimide **2a** (1.5 equiv.), 25 mg, 56% yield; colorless oil; ^{1}H NMR (400 MHz, $CDCl_3$) δ 7.31 (d, *J* = 8.4 Hz, 4H), 7.24 (d, *J* = 8.3 Hz, 4H), 4.48 (s, 4H), 4.17 (d, *J* = 13.1 Hz, 2H), 4.09 (d, *J* = 13.2 Hz, 2H), 3.73 (s, 2H), 2.95 (s, 3H) ^{13}C NMR (100 MHz, $CDCl_3$) δ 174.5, 142.7, 136.2, 133.7, 129.1, 128.8, 72.5, 65.3, 45.4, 24.9; HRMS *m/z* calculated for $[C_{23}H_{21}Cl_2NNaO_4]^+$ ($[M+Na]^+$): 468.0740, observed : 468.0740

6,7-bis(hydroxymethyl)-3-methyl-3-azabicyclo[3.2.0]hept-6-ene-2,4-dione (3na)

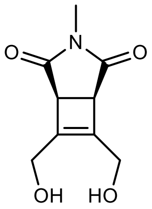

Prepared according to the ***General Procedure A*** using but-2-yne-1,4-diol (0.1 mmol, 1.0 equiv.) **1n** and *N*-methylmaleimide **2a** (1.5 equiv.), 18 mg, 91% yield; colorless oil; ^{1}H NMR (400 MHz, $CDCl_3$) δ 4.34 (s, 4H), 3.68 (s, 2H), 2.96 (s, 3H); ^{13}C NMR (100 MHz, $CDCl_3$) δ 174.9, 142.5, 58.7, 44.7, 24.8; HRMS *m/z* calculated for $[C_9H_{11}NNaO_4]^+$ ($[M+Na]^+$): 220.0580, observed : 220.0580

6-benzyl-7-(benzyldimethylsilyl)-3-methyl-3-azabicyclo[3.2.0]hept-6-ene-2,4-dione (3oa)

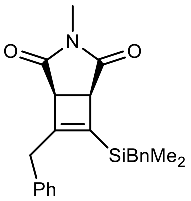

Prepared according to the ***General Procedure A*** using benzyldimethyl(3-phenylprop-1-yn-1-yl)silane **1o** (0.1 mmol, 1.0 equiv.) and *N*-methylmaleimide **2a** (1.5 equiv.), 27 mg, 72% yield; colorless oil; ^{1}H NMR (400 MHz, $CDCl_3$) δ 7.31 – 7.28 (m, 2H), 7.24 – 7.18 (m, 3H), 7.12 – 7.07 (m, 3H), 6.95 – 6.92 (m, 2H), 3.61 (d, *J* = 3.2 Hz, 1H), 3.44 (d, *J* = 3.2 Hz, 1H), 3.26 (s, 2H), 2.96 (s, 3H), 2.22 (d, *J* = 2.6 Hz, 2H), 0.23 (s, 3H), 0.19 (s, 3H) ^{13}C NMR (100 MHz, $CDCl_3$) δ 176.2, 174.7, 162.7, 146.6, 139.1, 136.7, 129.0, 128.9, 128.4, 128.3, 126.8, 124.5,

48.8, 45.1, 37.5, 25.5, 24.8, -2.9, -3.0; HRMS *m/z* calculated for $[C_{23}H_{25}NNaO_2Si]^+$ ($[M+Na]^+$): 398.1547, observed : 398.1548

6-(1-hydroxycyclohexyl)-3-methyl-7-(trimethylsilyl)-3-azabicyclo[3.2.0]hept-6-ene-2,4-dione (3pa)

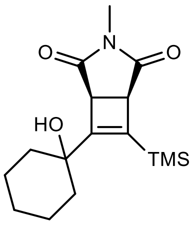

Prepared according to the ***General Procedure A*** using 1-((trimethylsilyl)ethynyl)cyclohexan-1-ol **1p** (0.1 mmol, 1.0 equiv.) and *N*-methylmaleimide **2a** (1.5 equiv.), 29 mg, 94% yield; white solid; m.p. 89 – 91 °C; ^{1}H NMR (400 MHz, $CDCl_3$) δ 3.83 (d, *J* = 3.2 Hz, 1H), 3.48 (d, *J* = 3.2 Hz, 1H), 2.95 (s, 3H), 1.72 – 1.49 (m, 9H), 1.30 – 1.16 (m, 1H), 0.21 (s, 9H); ^{13}C NMR (100 MHz, $CDCl_3$) δ 175.9, 175.8, 168.0, 146.2, 72.7, 47.1, 43.8, 36.4, 36.0, 25.2, 25.0, 21.24, 21.18, -0.4; HRMS *m/z* calculated for $[C_{16}H_{26}NO_3Si]^+$ ($[M+H]^+$): 308.1676, observed 308.1679.

6-(1-hydroxycyclohexyl)-3-methyl-3-azabicyclo[3.2.0]hept-6-ene-2,4-dione (3qa)

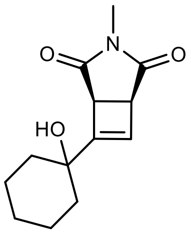

Prepared according to the ***General Procedure A*** using 1-ethynylcyclohexan-1-ol **1q** (0.1 mmol, 1.0 equiv.) and *N*-methylmaleimide **2a** (1.5 equiv.), 14 mg, 60% yield; white solid; m.p. 120 – 122 °C; ^{1}H NMR (400 MHz, $CDCl_3$) δ 6.18 (d, *J* = 1.1 Hz, 1H), 3.85 (d, *J* = 3.2 Hz, 1H), 3.63 (dd, *J* = 3.2, 1.0 Hz, 1H), 2.97 (s, 3H), 1.69 – 1.28 (m, 10H); ^{13}C NMR (100 MHz, $CDCl_3$) δ 175.6, 175.2, 158.3, 128.3, 71.0, 46.4, 43.1, 35.9, 35.2, 25.4, 25.1, 21.8, 21.7; HRMS *m/z* calculated for $[C_{13}H_{18}NO_3]^+$ ($[M+H]^+$): 236.1281, observed : 236.1289

***N*-(1-(3-methyl-2,4-dioxo-3-azabicyclo[3.2.0]hept-6-en-6-yl)cyclohexyl)acetamide (3ra)**

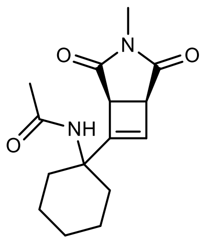

Prepared according to the ***General Procedure A*** using *N*-(1-ethynylcyclohexyl)acetamide **1r** (0.1 mmol, 1.0 equiv.) and *N*-methylmaleimide **2a** (1.5 equiv.), 27 mg, 98% yield; white solid; ^{1}H NMR (400 MHz, $CDCl_3$) δ 6.16 (d, *J* = 1.2 Hz, 1H), 5.48 (br s, 1 H), 3.71 (d, *J* = 3.2 Hz, 1H), 3.61 (dd, *J* = 3.2, 1.2 Hz, 1H), 2.93 (s, 3H), 2.09 – 2.02 (m, 2H), 1.97 (s, 3H), 1.62 – 1.26 (m, 8H) ^{13}C NMR (100 MHz, $CDCl_3$) δ 175.5, 175.3, 169.8, 156.0, 128.7, 55.3, 46.7, 42.9, 33.4, 33.1, 25.3, 25.0, 24.1, 21.5, 21.4; HRMS *m/z* calculated for $[C_{15}H_{20}N_2NaO_3]^+$ ($[M+Na]^+$): 299.1366, observed : 299.1366

2-(3-cyclohexyl-2,4-dioxo-3-azabicyclo[3.2.0]hept-6-en-6-yl)-2-hydroxypropanoic acid (**3si**)

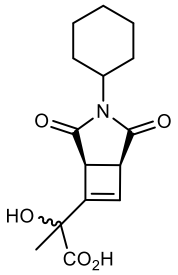

Prepared according to the ***General Procedure A*** using 2-hydroxy-2-methylbut-3-ynoic acid **1s** (0.1 mmol, 1.0 equiv.) and 1-cyclohexyl-1*H*-pyrrole-2,5-dione **2i** (1.5 equiv.), 25 mg, 85% yield (d.r. = 1.3:1); pale yellow solid; The NMR spectrum was obtained on a partially purified material as a mixture of diastereomers; ^{1}H NMR (400 MHz, CD_3OD) δ 6.31 (dd, *J* = 4.8, 1.1 Hz, 2H), 3.92 – 3.82 (m, 3H), 3.74 (d, *J* = 3.2 Hz, 1H), 3.5 (ddd, *J* = 5.5, 3.2, 1.0 Hz, 2H), 2.16 – 2.01 (m, 4H), 1.84 – 1.81 (m, 4H), 1.68 – 1.51 (m, 12H), 1.40 – 1.13 (m, 6H) ^{13}C NMR (100 MHz, CD_3OD) δ 177.8, 177.7, 177.5, 177.4, 177.11, 177.07, 156.2, 155.7, 131.7, 131.5, 75.5, 72.6, 52.7, 52.7, 48.0, 47.6, 44.0, 44.0, 29.6, 29.5, 29.5, 27.02, 27.00, 26.3, 24.6, 23.9; HRMS *m/z* calculated for $[C_{15}H_{19}NNaO_5]^+$ ($[M+Na]^+$): 316.1155, observed : 316.1155

6-((1*r*,3*r*,5*r*,7*r*)-adamantan-2-yl)-3-methyl-3-azabicyclo[3.2.0]hept-6-ene-2,4-dione (3ta)

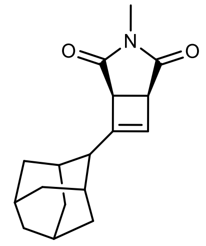

Prepared according to the ***General Procedure A*** using (3*r*,5*r*,7*r*)-1-ethynyladamantane **1t** (0.1 mmol, 1.0 equiv.) and *N*-methylmaleimide **2a** (1.5 equiv.), 13 mg, 48% yield; white solid; m.p. 138-139 °C; ^{1}H NMR (400 MHz, $CDCl_3$) δ 5.97 (d, *J* = 0.9 Hz, 1H), 3.76 (d, *J* = 3.2 Hz, 1H), 3.55 (dd, *J* = 3.2, 1.1 Hz, 1H), 2.95 (s, 3H), 2.00 (s, 3H), 1.77 – 1.62 (m, 12H).; ^{13}C NMR (100 MHz, $CDCl_3$) δ 175.9, 175.4, 162.1, 125.8, 46.2, 43.1, 39.6, 36.5, 36.1, 27.8, 24.8; HRMS *m/z* calculated for $[C_{17}H_{22}NO_2]^+$ ($[M+H]^+$): 272.1645, observed 272.1645.

6-(2-hydroxy-2-phenylethyl)-3-methyl-3-azabicyclo[3.2.0]hept-6-ene-2,4-dione (3ua)

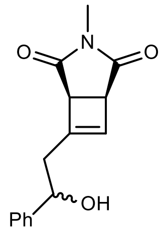

Prepared according to the ***General Procedure A*** using 1-phenylbut-3-yn-1-ol **1u** (0.1 mmol, 1.0 equiv.) and *N*-methylmaleimide **2a** (1.5 equiv.), 20 mg, 78% yield (d.r. = 1:1); pale yellow solid; m.p. 104 – 106 °C; The NMR spectrum was obtained on a partially purified material as a mixture of diastereomers; ^{1}H NMR (400 MHz, $CDCl_3$) δ 7.35 – 7.34 (m, 6.13H), 7.31 – 7.28 (m, 1.28H), 6.17 (d, *J* = 1.4 Hz, 1H), 6.14 (d, *J* = 1.4 Hz, 0.5H), 4.98 (dt, *J* = 7.9, 3.7 Hz, 1H), 4.89 (dt, *J* = 8.1, 3.9 Hz, 0.5H), 3.78 (d, *J* = 3.0 Hz, 0.5H), 3.74 (d, *J* = 2.8 Hz, 1H), 3.64 (m, 1.5H), 3.01 (d, *J* = 3.7 Hz, 0.5H), 2.96 (s, 1.5H), 2.91 (s, 3H), 2.73 – 2.57 (m, 3.4H), 2.39 (d, *J* = 4.1 Hz, 1H) ^{13}C NMR (100 MHz, $CDCl_3$) δ 175.8, 175.8, 175.5, 175.4, 150.5, 150.5, 143.5, 143.5, 132.4, 132.2, 128.7, 128.7, 128.0, 128.0, 125.8, 125.8, 71.8, 71.7, 49.2, 48.9, 44.7, 44.6, 39.9, 39.5, 25.0, 24.9; HRMS *m/z* calculated for $[C_{15}H_{15}NNaO_3]^+$ ($[M+Na]^+$): 280.0944, observed : 280.0944

3-methyl-6-(*p*-tolyl)-7-(*p*-tolylethynyl)-3-azabicyclo[3.2.0]hept-6-ene-2,4-dione (3va)

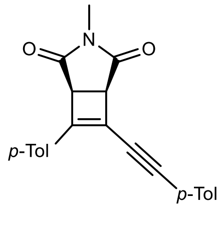

Prepared according to the ***General Procedure A*** using 1,4-di-*p*-tolylbuta-1,3-diyne **1v** (0.1 mmol, 1.0 equiv.) and *N*-methylmaleimide **2a** (1.5 equiv.), 24 mg, 70% yield; white solid; m.p. 163 – 165 °C; ^{1}H NMR (400 MHz, $CDCl_3$) δ 7.78 (d, *J* =8.2 Hz, 2H), 7.43 (d, *J* = 8.1 Hz, 2H), 7.23 (d, *J* = 7.9 Hz, 2H), 7.18 (d, *J* = 7.9 Hz, 2H), 4.08 (d, *J* = 3.6 Hz, 1H), 3.95 (d, *J* = 3.6 Hz, 1H), 2.98 (s, 3H), 2.38 (s, 6H); ^{13}C NMR (100 MHz, $CDCl_3$) δ 174.2, 174.0, 148.6, 140.2, 139.5, 131.7, 129.6, 129.4, 129.2, 126.4, 119.2, 118.0, 99.3, 82.5, 46.6, 45.4, 24.9, 21.6, 21.6; HRMS *m/z* calculated for $[C_{23}H_{20}NO_2]^+$ ($[M+H]^+$): 342.1489, observed : 342.1490

4-(benzylcarbamoyl)-2,3-di-*p*-tolylcyclobut-2-ene-1-carboxylic acid (3ab′)

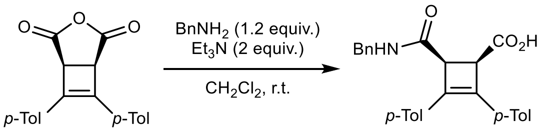

6,7-di-*p*-tolyl-3-oxabicyclo[3.2.0]hept-6-ene-2,4-dione **3ab** was prepared according to the ***General Procedure A*** using di(*p*-tolyl)acetylene **1a** (0.1 mmol, 1.0 equiv.) and maleic anhydride (1.5 equiv.), and used without further purification. To a solution of 6,7-di-*p*-tolyl-3-oxabicyclo[3.2.0]hept-6-ene-2,4-dione **3ab** (1.0 equiv.) in CH_2Cl_2 (1 mL) was added Et_3N (0.2 mmol, 2.0 equiv.) and benzylamine (1.2 equiv.). The solution was stirred for 1 h, and then the mixture was concentrated *in vacuo*. The resulting crude product was purified by flash column chromatography on silica gel to afford the desired product., 40 mg, 97% yield; white solid; m.p. 189 – 190 °C; ^{1}H NMR (400 MHz, DMSO-d_6) δ 12.09 (br s, 1H), 8.61 (t, *J* = 5.9 Hz, 1H), 7.45 (d, *J* = 8.1 Hz, 2H), 7.32 – 7.24 (m, 7H), 7.17 (d, *J* = 8.0 Hz, 2H), 7.14 (d, *J* = 8.0 Hz, 2H), 4.31 (dd, *J* = 5.9, 2.6 Hz, 2H), 4.12 – 4.09 (m, 2H), 2.31 (s, 3H), 2.30 (s, 3H) ^{13}C NMR (100 MHz, DMSO-d_6) δ 171.6, 169.7, 139.4, 137.8, 137.7, 137.5, 137.3, 131.5, 131.3, 129.0, 128.8, 128.2, 127.3, 126.73, 126.71, 126.1, 48.1, 45.1, 42.3, 29.9, 20.9; HRMS *m/z* calculated for $[C_{27}H_{25}NNaO_3]^+$ ($[M+Na]^+$): 434.1727, observed : 434.1734

(3,4-di-*p*-tolylcyclobut-3-ene-1,2-diyl)bis(pyrrolidin-1-ylmethanone) (3ac)

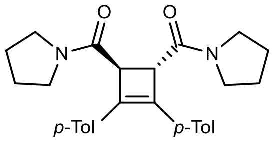

Prepared according to the ***General Procedure A*** using di(*p*-tolyl)acetylene **1a** (0.1 mmol, 1.0 equiv.) and *(E)*-1,4-di(pyrrolidin-1-yl)but-2-ene-1,4-dione **2c** (1.5 equiv.), 27 mg, 63% yield; white solid; m.p. 183 – 184 °C; ^{1}H NMR (400 MHz, $CDCl_3$) δ 7.25 (d, *J* = 8.1 Hz, 4H), 7.07 (d, *J* = 7.9 Hz, 4H), 4.21 (s, 2H), 3.58 – 3.51 (m, 6H), 3.48 – 3.42 (m, 2H), 2.31 (s, 6H), 1.95 – 1.82 (m, 8H) ^{13}C NMR (100 MHz, $CDCl_3$) δ 170.0, 139.3, 137.7, 131.8, 129.0, 126.5, 46.8, 46.5, 46.0, 26.2, 24.3, 21.4; HRMS *m/z* calculated for $[C_{28}H_{33}N_2O_2]^+$ ($[M+H]^+$): 429.2537, observed : 429.2542; The stereochemistry was confirmed by desymmetrization to **3ac′**

pyrrolidin-1-yl(4-(pyrrolidin-1-ylmethyl)-2,3-di-*p*-tolylcyclobut-2-en-1-yl)methanone (3ac′)

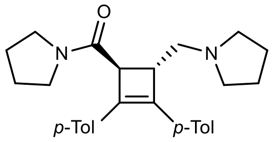

^{1}H NMR (400 MHz, $CDCl_3$) δ 7.34 (d, *J* = 8.1 Hz, 2H), 7.29 (d, *J* = 8.1 Hz, 2H), 7.15 (d, *J* = 7.8 Hz, 2H), 7.08 (d, *J* = 7.9 Hz, 2H), 4.14 (d, *J* = 1.5 Hz, 1H), 3.98 (dt, *J* = 10.0, 6.6 Hz, 1H), 3.79 (dt, *J* = 9.9, 2.0 Hz, 1H), 3.61 (dt, *J* = 10.0, 6.9 Hz, 1H), 3.53 (dt, *J* = 12.1, 7.1 Hz, 1H), 3.42 (ddd, *J* = 12.1, 7.5, 6.0 Hz, 1H), 3.36 – 3.30 (m, 1H), 3.27 – 3.21 (m, 1H), 3.01 – 2.91 (m, 2H), 2.81 – 2.75 (m, 1H), 2.73 – 2.66 (m, 1H), 2.36 (s, 3H), 2.32 (s, 3H), 2.21 – 2.16 (m, 2H), 1.95 – 1.74 (m, 6H); MS (APCI): *m/z* 416.0 $[M+H]^+$

***N*[1],*N*[2]-dimethyl-*N*[1],*N*[2]-diphenyl-3,4-di-*p*-tolylcyclobut-3-ene-1,2-dicarboxamide (3ad)**

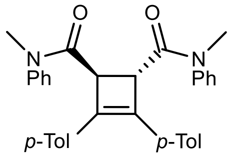

Prepared according to the ***General Procedure A*** using di(*p*-tolyl)acetylene **1a** (0.1 mmol, 1.0 equiv.) and *N*[1],*N*[4]-dimethyl-*N*[1],*N*[4]-diphenylmaleamide **2d** (1.5 equiv.), 27 mg, 54% yield; white solid; m.p. 167 – 169 °C; ^{1}H NMR (400 MHz, $CDCl_3$) δ 7.49 – 7.29 (m, 6H), 7.19 – 6.99 (m, 12H), 3.96 (s, 2H), 3.20 (s, 6H), 2.32 (s, 6H); ^{13}C NMR (100 MHz, $CDCl_3$) δ 170.7, 143.4, 139.3, 137.7, 131.3, 129.7, 128.9, 128.0, 127.2, 126.6, 46.0, 37.9, 21.4; HRMS *m/z* calculated for $[C_{34}H_{32}N_2NaO_2]^+$ ($[M+Na]^+$) : 523.2356, observed 523.2356; The stereochemistry was confirmed by desymmetrization to **3ad′**.

(4-((methyl(phenyl)amino)methyl)-2,3-di-*p*-tolylcyclobut-2-en-1-yl)methanol (3ad′)

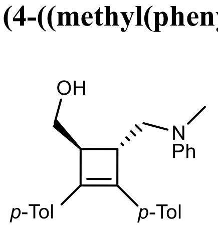

^{1}H NMR (400 MHz, $CDCl_3$) δ 7.38 (d, *J* = 8.1 Hz, 4H), 7.28 – 7.24 (m, 2H), 7.15 – 7.11 (m, 4H), 6.79 (d, *J* = 7.9 Hz, 2H), 6.73 (t, *J* = 7.3 Hz, 1H), 3.85 (dd, *J* = 11.1, 4.4 Hz, 1H), 3.71 – 3.64 (m, 2H), 3.41 (dd, *J* = 14.7, 8.9 Hz, 1H), 3.31 (ddd, *J* = 8.8, 4.7, 1.5 Hz, 1H), 3.13 (ddd, *J* = 7.3, 4.3, 1.5 Hz, 1H), 2.90 (s, 3H), 2.36 (s, 3H), 2.34 (s, 3H); MS (APCI): *m/z* 384.1 $[M+H]^+$

Diethyl 3,4-di-*p*-tolylcyclobut-3-ene-1,2-dicarboxylate (3ae)

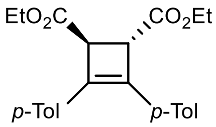

Prepared according to the ***General Procedure A*** using di(*p*-tolyl)acetylene **1a** (0.1 mmol, 1.0 equiv.) and Diethyl fumarate **2e** (1.5 equiv.), 19 mg, 51% yield; white solid; m.p. 91 – 93 °C; ^{1}H NMR (400 MHz, $CDCl_3$) δ 7.43 (d, *J* = 8.2 Hz, 4H), 7.12 (d, *J* = 7.9 Hz, 4H), 4.18 – 4.08 (m, 6H), 2.35 (s, 6H), 1.19 (t, *J* = 7.1 Hz, 6H); ^{13}C NMR (100 MHz, $CDCl_3$) δ 171.5, 138.3, 138.0, 130.6, 129.0, 126.6, 60.9, 45.9, 21.4, 14.1; HRMS *m/z* calculated for $[C_{24}H_{27}O_4]^+$ ($[M+H]^+$): 379.1904, observed : 379.1918

3-(*p*-tolyl)-4-(*p*-tolylethynyl)cyclobut-3-ene-1,2-dicarbonitrile (3vf)

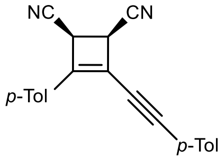

***cis*-3vf**

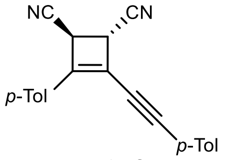

***trans*-3vf**

Prepared according to the ***General Procedure A*** using di(*p*-tolyl)acetylene **1a** (0.1 mmol, 1.0 equiv.) and fumaronitrile (1.5 equiv.), 16 mg, 51% yield, (*cis*/*trans* 1.3:1); **(*cis*-3vf)**: yellow solid; m.p. 163 – 164 °C; ^{1}H NMR (400 MHz, $CDCl_3$) δ 7.62 (d, *J* = 8.1 Hz, 2H), 7.45 (d, *J* = 8.1 Hz, 2H), 7.27 (d, *J* = 8.1 Hz, 2H), 7.21 (d, *J* = 7.9 Hz, 2H), 4.23 (d, *J* = 5.1 Hz, 1H), 4.17 (d, *J* = 5.1 Hz, 1H), 2.41 (s, 3H), 2.40 (s, 3H); ^{13}C NMR (100 MHz, $CDCl_3$) δ 143.8, 141.5, 140.3, 131.9, 129.7, 129.4, 127.8, 125.9, 118.2, 115.2, 115.1, 114.7, 99.8, 81.0, 33.9, 31.3, 21.71, 21.67; HRMS *m/z* calculated for $[C_{22}H_{16}N_2Na]^+$ ($[M+Na]^+$): 331.1206, observed 331.1206; **(*trans*-3vf)**: yellow solid; m.p. 57 – 58 °C; ^{1}H NMR (400 MHz, $CDCl_3$); δ 7.61 (d, *J* = 8.1 Hz, 2H), 7.45 (d, *J* = 8.1 Hz, 2H), 7.28 (d, *J* = 8.0 Hz, 2H), 7.21 (d, *J* = 7.9 Hz, 2H), 4.11 (d, *J* = 2.4 Hz, 1H), 4.07 (d, *J* = 2.3 Hz, 1H), 2.41 (s, 3H), 2.40 (s, 3H); ^{13}C NMR (100 MHz, $CDCl_3$) δ 143.6, 141.6, 140.4, 131.9, 129.8, 129.4, 127.7, 125.9, 118.2, 115.92, 115.90, 114.7, 99.9, 80.9, 34.1, 31.4, 21.71, 21.67; HRMS *m/z* calculated for $[C_{22}H_{16}N_2Na]^+$ ($[M+Na]^+$): 331.1206, observed 331.1206.

Methyl 4-(ethyl(*p*-tolyl)carbamoyl)-2,3-diphenylcyclobut-2-ene-1-carboxylate (3cg)

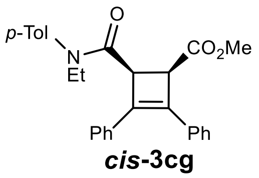

***cis*-3cg**

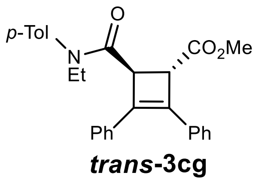

***trans*-3cg**

Prepared according to the ***General Procedure A*** using 1,2-diphenylethyne **1c** (0.1 mmol, 1.0 equiv.) and methyl (*Z*)-4-(ethyl(*p*-tolyl)amino)-4-oxobut-2-enoate **2g** (1.5 equiv.), 18 mg, 42% yield (*cis*/*trans* 1:5); white solid; (***cis*-3cg):** ^{1}H NMR (400 MHz, $CDCl_3$) δ 7.52 – 7.49 (m, 2H), 7.41 – 7.38 (m, 2H), 7.31 – 7.22 (m, 10H), 4.07 – 4.02 (m, 2H), 3.86 (d, *J* = 5.5 Hz, 1H), 3.72 (s, 3H), 3.53 – 3.45 (m, 1H), 2.40 (s, 3H), 1.12 (t, *J* = 7.1 Hz, 3H) ^{13}C NMR (100 MHz, $CDCl_3$) δ 171.5, 169.3, 140.0, 139.7, 138.1, 138.0, 134.3, 134.2, 130.5, 128.5, 128.39, 128.35, 128.3, 128.2, 127.1, 126.8, 52.1, 46.9, 45.9, 44.7, 21.2, 13.1; HRMS *m/z* calculated for $[C_{28}H_{27}NNaO_3]^+$ ($[M+Na]^+$): 448.1883, observed : 448.1884; (***trans*-3cg):** ^{1}H NMR (400 MHz, $CDCl_3$) δ 7.45 – 7.41 (m, 4H), 7.34 – 7.22 (m, 8H), 7.08 (d, *J* = 8.1 Hz, 2H), 3.95 (d, *J* = 2.0 Hz, 1H), 3.93 (d, *J* = 2.1 Hz, 1H), 3.83 (dq, *J* = 14.2, 7.1 Hz, 1H), 3.66 (dq, *J* = 14.2, 7.1 Hz, 1H), 3.50 (s, 3H), 2.39 (s, 3H), 1.10 (t, *J* = 7.1 Hz, 3H) ^{13}C NMR (100 MHz, $CDCl_3$) δ 171.9, 170.1, 140.4, 139.1, 138.2, 138.1, 134.2, 133.6, 130.4, 128.5, 128.44, 128.42, 128.38, 128.36, 126.8, 126.5, 51.9, 47.5, 45.1, 44.4, 21.2, 13.2; HRMS *m/z* calculated for $[C_{28}H_{27}NNaO_3]^+$ ($[M+Na]^+$): 448.1883, observed : 448.1885

6,7-di-*p*-tolyl-3-azabicyclo[3.2.0]hept-6-ene-2,4-dione (3ah)

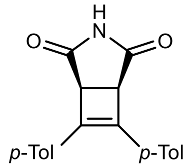

Prepared according to the ***General Procedure A*** using di(*p*-tolyl)acetylene **1a** (0.1 mmol, 1.0 equiv.) and maleimide **2h** (1.5 equiv.), 25 mg, 82% yield; white solid; m.p. 227 – 229 °C; ^{1}H NMR (400 MHz, $CDCl_3$) δ 7.62 (d, *J* = 8.2 Hz, 4H), 7.58 (br s, 1H), 7.19 (d, *J* = 8.0 Hz, 4H), 4.08 (d, *J* = 1.0 Hz, 2H), 2.37 (s, 6H) ^{13}C NMR (100 MHz, $CDCl_3$) δ 175.4, 139.4, 138.1, 130.3, 129.5, 126.9, 46.4, 21.6; HRMS *m/z* calculated for $[C_{20}H_{18}NO_2]^+$ ($[M+H]^+$): 304.1332, observed : 304.1330

3-cyclohexyl-6,7-di-*p*-tolyl-3-azabicyclo[3.2.0]hept-6-ene-2,4-dione (3ai)

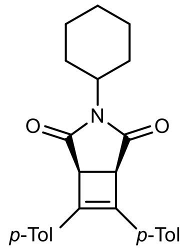

Prepared according to the ***General Procedure A*** using di(*p*-tolyl)acetylene **1a** (0.1 mmol, 1.0 equiv.) and 1-cyclohexyl-1*H*-pyrrole-2,5-dione **2i** (1.5 equiv.), 31 mg, 80% yield; white solid; m.p. 178 – 180 °C; ^{1}H NMR (400 MHz, $CDCl_3$) δ 7.63 (d, *J* = 8.1 Hz, 4H), 7.17 (d, *J* = 8.0 Hz, 4H), 3.97 (s, 2H), 3.91 (tt, *J* = 12.3, 3.9 Hz, 1H), 2.36 (s, 6H), 2.15 – 2.00 (m, 2H), 1.85 – 1.75 (m, 2H), 1.68 – 1.56 (m, 2H), 1.36 – 1.11 (m, 4H) ^{13}C NMR (100

MHz, $CDCl_3$) δ 175.5, 139.2, 138.8, 130.5, 129.4, 126.9, 51.6, 44.6, 28.7, 26.0, 25.2, 21.6; HRMS *m/z* calculated for $[C_{26}H_{28}NO_2]^+$ ($[M+H]^+$): 386.2115, observed : 386.2115

3-(benzyloxy)-6,7-di-*p*-tolyl-3-azabicyclo[3.2.0]hept-6-ene-2,4-dione (3aj)

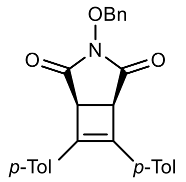

Prepared according to the ***General Procedure A*** using di(*p*-tolyl)acetylene **1a** (0.1 mmol, 1.0 equiv.) and 1-(benzyloxy)-1*H*-pyrrole-2,5-dione **2j** (1.5 equiv.), 17 mg, 42% yield; white solid; m.p. 167 – 169 °C; ^{1}H NMR (400 MHz, $CDCl_3$) δ 7.56 (d, *J* = 8.2 Hz, 4H), 7.39 – 7.36 (m, 2H), 7.21 – 7.17 (m, 7H), 5.07 (s, 2H), 3.97 (s, 2H), 2.38 (s, 6H) ^{13}C NMR (100 MHz, $CDCl_3$) δ 170.3, 139.5, 138.1, 133.3, 130.2, 130.1, 129.42, 129.36, 128.5, 126.9, 78.4, 42.2, 21.6; HRMS *m/z* calculated for $[C_{27}H_{23}NNaO_3]^+$ ($[M+Na]^+$): 432.1570, observed : 432.1583

4-(2,4-dioxo-6,7-di-*p*-tolyl-3-azabicyclo[3.2.0]hept-6-en-3-yl)benzonitrile (3ak)

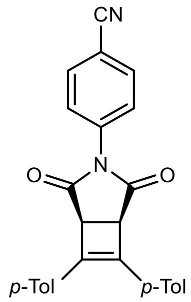

Prepared according to the ***General Procedure A*** using di(*p*-tolyl)acetylene **1a** (0.1 mmol, 1.0 equiv.) and 4-(2,5-dioxo-2,5-dihydro-1*H*-pyrrol-1-yl)benzonitrile **2k** (1.5 equiv.), 24 mg, 59% yield; white solid; m.p. 214 – 215 °C; ^{1}H NMR (400 MHz, $CDCl_3$) δ 7.71 (d, *J* = 8.9 Hz, 2H), 7.67 (d, *J* = 8.2 Hz, 4H), 7.47 (d, *J* = 8.9 Hz, 2H), 7.20 (d, *J* = 7.9 Hz, 4H), 4.24 (s, 2H), 2.38 (s, 6H); ^{13}C NMR (100 MHz, $CDCl_3$) δ 173.2, 139.6, 138.1, 135.9, 129.9, 129.4, 127.0, 126.8, 118.1, 111.9, 44.8, 21.5; HRMS *m/z* calculated for $[C_{27}H_{21}N_2O_2]^+$ ($[M+H]^+$): 405.1598, observed : 405.1612

Methyl 4-(2,4-dioxo-6,7-di-*p*-tolyl-3-azabicyclo[3.2.0]hept-6-en-3-yl)benzoate (3al)

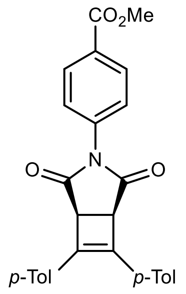

Prepared according to the ***General Procedure A*** using di(*p*-tolyl)acetylene **1a** (0.1 mmol, 1.0 equiv.) and methyl 4-(2,5-dioxo-2,5-dihydro-1*H*-pyrrol-1-yl)benzoate **2l** (1.5 equiv.), 20 mg, 46% yield; white solid; m.p. 227 – 229 °C; ^{1}H NMR (400 MHz, $CDCl_3$) δ 8.09 (d, *J* = 8.5 Hz, 2H), 7.68 (d, *J* = 8.3, 4H), 7.39 (d, *J* = 8.5 Hz, 2H), 7.20 (d, *J* = 8.4 Hz, 4H), 4.23 (s, 2H), 3.91 (s, 3H), 2.38 (s, 6H) ^{13}C NMR (100 MHz, $CDCl_3$) δ 173.7, 166.4, 139.6, 138.4, 136.2, 130.4, 130.2, 129.9, 129.5, 127.0, 126.5, 52.4, 45.0, 21.6; HRMS *m/z* calculated for $[C_{28}H_{24}NO_4]^+$ ($[M+H]^+$): 438.1700, observed : 438.1719

3-phenyl-6,7-di-*p*-tolyl-3-azabicyclo[3.2.0]hept-6-ene-2,4-dione (3am)

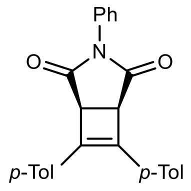

Prepared according to the ***General Procedure A*** using di(*p*-tolyl)acetylene **1a** (0.1 mmol, 1.0 equiv.) and 1-phenyl-1*H*-pyrrole-2,5-dione **2m** (1.5 equiv.), 11 mg, 29% yield; white solid; m.p. 185 – 187 °C; ^{1}H NMR (400 MHz, $CDCl_3$) δ 7.69 (d, *J* = 8.1 Hz, 4H), 7.44 – 7.40 (m, 2H), 7.36 – 7.32 (m, 1H), 7.27 – 7.24 (m, 2H), 7.19 (d, *J* = 7.9 Hz, 4H), 4.21 (s, 2H), 2.37 (s, 6H) ^{13}C NMR (100 MHz, $CDCl_3$) δ 174.2, 139.5, 138.5, 132.1, 130.4, 129.5, 129.1, 128.6, 127.0, 126.7, 45.1, 21.6; HRMS *m/z* calculated for $[C_{26}H_{22}NO_2]^+$ ($[M+H]^+$): 380.1645, observed : 380.1645

3-(pyridin-3-yl)-6,7-di-*p*-tolyl-3-azabicyclo[3.2.0]hept-6-ene-2,4-dione (3an)

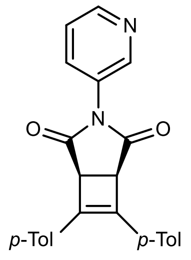

Prepared according to the ***General Procedure A*** using di(*p*-tolyl)acetylene **1a** (0.1 mmol, 1.0 equiv.) and 1-(pyridin-3-yl)-1*H*-pyrrole-2,5-dione **2n** (1.5 equiv.), 21 mg, 55% yield; white solid; m.p. 195 – 196 °C; ^{1}H NMR

(400 MHz, $CDCl_3$) δ 8.60 – 8.58 (m, 2H), 7.68 (d, *J* = 8.3 Hz, 4H), 7.66 – 7.63 (m, 1H), 7.36 (dd, *J* = 8.2, 4.8 Hz, 1H), 7.20 (d, *J* = 8.3 Hz, 4H), 4.25 (s, 2H), 2.38 (s, 6H) ^{13}C NMR (100 MHz, $CDCl_3$) δ 173.7, 149.3, 147.6, 139.7, 138.3, 134.0, 130.1, 129.5, 128.1, 127.0, 123.6, 45.1, 21.6; HRMS *m/z* calculated for $[C_{25}H_{21}N_2O_2]^+$ ($[M+H]^+$): 381.1598, observed : 381.1599

1-bromo-3-methyl-6,7-di-*p*-tolyl-3-azabicyclo[3.2.0]hept-6-ene-2,4-dione (3ao)

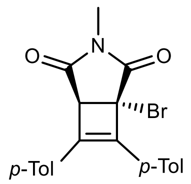

Prepared according to the ***General Procedure A*** using di(*p*-tolyl)acetylene **1a** (0.1 mmol, 1.0 equiv.) and 3-bromo-1-methyl-1*H*-pyrrole-2,5-dione **2o** (1.5 equiv.), 37 mg, 93% yield; pale yellow solid; m.p. 214 – 215 °C; ^1H NMR (400 MHz, $CDCl_3$) δ 7.71 (d, *J* = 8.2 Hz, 2H), 7.63 (d, *J* = 8.2 Hz, 2H), 7.24 (d, *J* = 8.0 Hz, 2H), 7.20 (d, *J* = 7.9 Hz, 2H), 4.30 (s, 1H), 3.01 (s, 3H), 2.40 (s, 3H), 2.37 (s, 3H) ^{13}C NMR (100 MHz, CD_2Cl_2) δ 172.9, 172.2, 141.4, 140.7, 138.9, 138.7, 130.00, 129.95, 129.4, 128.4, 128.2, 128.0, 56.0, 52.0, 25.8, 21.9, 21.8; HRMS *m/z* calculated for $[C_{21}H_{18}BrNNaO_2]^+$ ($[M+Na]^+$): 418.0413, observed : 418.0413

1-methoxy-3-methyl-6,7-di-*p*-tolyl-3-azabicyclo[3.2.0]hept-6-ene-2,4-dione (3ap)

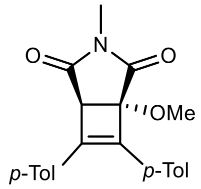

Prepared according to the ***General Procedure A*** using di(*p*-tolyl)acetylene **1a** (0.1 mmol, 1.0 equiv.) and 3-methoxy-1-methyl-1*H*-pyrrole-2,5-dione **2p** (1.5 equiv.), 34 mg, 98% yield; white solid; m.p. 152 – 154 °C; ^1H NMR (400 MHz, $CDCl_3$) δ 7.71 (d, *J* = 8.2 Hz, 2H), 7.66 (d, *J* = 8.2 Hz, 2H), 7.20 (d, *J* = 8.2 Hz, 4H), 4.11 (s, 1H), 3.42 (s, 3H), 2.99 (s, 3H), 2.37 (s, 6H); ^{13}C NMR (100 MHz, $CDCl_3$) δ 174.0, 173.5, 140.1, 139.4, 139.3, 138.7, 129.4, 129.4, 129.3, 128.9, 127.3, 127.1, 81.7, 53.4, 49.1, 24.7, 21.49, 21.48; HRMS *m/z* calculated for $[C_{22}H_{22}NO_3]^+$ ($[M+H]^+$): 348.1594, observed 348.1595.

1,3-dimethyl-6,7-di-*p*-tolyl-3-azabicyclo[3.2.0]hept-6-ene-2,4-dione (3aq)

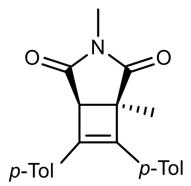

Prepared according to the ***General Procedure A*** using di(*p*-tolyl)acetylene **1a** (0.1 mmol, 1.0 equiv.) and 1,3-dimethyl-1*H*-pyrrole-2,5-dione **2q** (1.5 equiv.), 29 mg, 88% yield; white solid; m.p. 182 – 183 °C; ^{1}H NMR (400 MHz, $CDCl_3$) δ 7.61 (d, *J* = 8.2 Hz, 2H), 7.58 (d, *J* = 8.2 Hz, 2H), 7.18 (d, *J* = 7.9 Hz, 2H), 7.16 (d, *J* = 7.9 Hz, 2H), 3.78 (s, 1H), 2.97 (s, 3H), 2.37 (s, 3H), 2.35 (s, 3H), 1.63 (s, 3H) ^{13}C NMR (100 MHz, $CDCl_3$) δ 178.7, 175.0, 142.1, 139.3, 139.1, 137.2, 130.0, 129.9, 129.5, 129.4, 127.3, 126.9, 51.6, 50.9, 29.8, 25.0, 21.6, 15.8; HRMS *m/z* calculated for $[C_{22}H_{21}NNaO_2]^+$ ($[M+Na]^+$): 354.1465, observed : 354.1472.

1-methyl-3-phenyl-6,7-di-*p*-tolyl-3-azabicyclo[3.2.0]hept-6-ene-2,4-dione (3ar)

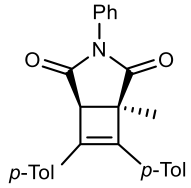

Prepared according to the ***General Procedure A*** using di(*p*-tolyl)acetylene **1a** (0.1 mmol, 1.0 equiv.) and 3-methyl-1-phenyl-1*H*-pyrrole-2,5-dione **2r** (1.5 equiv.), 39 mg, 99% yield; white solid; m.p. 210 – 211 °C; ^{1}H NMR (400 MHz, $CDCl_3$) δ 7.65 (t, *J* = 8.0 Hz, 4H), 7.47 – 7.31 (m, 3H), 7.27 (d, *J* = 8.6 Hz, 2H), 7.23 – 7.14 (m, 4H), 3.94 (s, 1H), 2.39 (s, 3H), 2.37 (s, 3H), 1.74 (s, 3H); ^{13}C NMR (100 MHz, $CDCl_3$) δ 177.4, 173.6, 142.1, 139.3, 139.0, 137.2, 132.1, 129.8, 129.7, 129.4, 129.3, 128.9, 128.3, 127.3, 126.9, 126.6, 51.3, 50.6, 21.48, 21.47, 15.8; HRMS *m/z* calculated for $[C_{27}H_{24}NO_2]^+$ ($[M+H]^+$): 394.1802, observed 394.1807.

1,3,5-trimethyl-6,7-di-*p*-tolyl-3-azabicyclo[3.2.0]hept-6-ene-2,4-dione (**3as**)

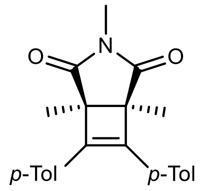

Prepared according to the ***General Procedure A*** using di(*p*-tolyl)acetylene **1a** (0.1 mmol, 1.0 equiv.) and 1,3,4-trimethyl-1*H*-pyrrole-2,5-dione **2s** (1.5 equiv.), 19 mg, 55% yield; white solid; m.p. 180 – 181 °C; ^{1}H NMR (400 MHz, $CDCl_3$) δ 7.54 (d, *J* = 8.2 Hz, 4H), 7.14 (d, *J* = 7.9 Hz, 4H), 2.97 (s, 3H), 2.35 (s, 6H), 1.58 (s, 6H); ^{13}C NMR (100 MHz, $CDCl_3$) δ 178.5, 142.1, 138.8, 129.7, 129.3, 127.2, 54.3, 24.8, 21.4, 13.3; HRMS *m/z* calculated for $[C_{23}H_{24}NO_2]^+$ ($[M+H]^+$): 346.1802, observed : 346.1808

4-(((*S*)-1-methoxy-1-oxopropan-2-yl)carbamoyl)-2,3-di-*p*-tolylcyclobut-2-ene-1-carboxylic acid (3at)

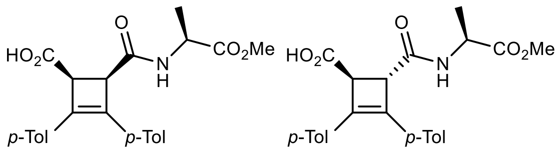

***cis*-3at** ***trans*-3at**

Prepared according to the ***General Procedure A*** using di(*p*-tolyl)acetylene **1a** (0.1 mmol, 1.0 equiv.) and (*S,Z*)-4-((1-methoxy-1-oxopropan-2-yl)amino)-4-oxobut-2-enoic acid **2t** (1.5 equiv.), 38 mg, 93% yield (*cis*/*trans* 4.4:1); The NMR spectrum was obtained on a partially purified material as a mixture of diastereomers; **(*cis*-3at) (d.r. 1:1) :** white solid; m.p. 168 – 169 °C; ^{1}H NMR (400 MHz, DMSO-d$_6$) δ 8.63 (d, *J* = 6.9 Hz, 0.5H), 8.50 (d, *J* = 7.7 Hz, 0.5H), 7.44 – 7.27 (m, 4H), 7.19 – 7.10 (m, 4H), 4.29 (td, *J* = 7.3, 5.4 Hz, 1H), 4.11 – 4.01 (m, 2H), 3.61 (s, 1.5H), 3.59 (s, 1.5H), 2.29 (s, 3H), 2.28 (s, 3H), 1.29 (d, *J* = 7.3 Hz, 1.5H), 1.25 (d, *J* = 7.1 Hz, 1.5H); ^{13}C NMR (100 MHz, DMSO-d$_6$) δ 173.5, 173.1, 171.8, 171.7, 170.1, 169.7, 138.4, 138.2, 138.12, 138.07, 137.8, 137.7, 137.6, 137.4, 131.92, 131.86, 131.60, 131.57, 129.5, 129.4, 129.22, 129.20, 127.2, 127.1, 126.48, 126.45, 52.3, 52.2, 48.11, 48.02, 47.97, 47.9, 45.5, 45.4, 21.4, 21.3, 17.8, 17.5.; HRMS m/z calculated for $[C_{24}H_{25}NNaO_5]^+$ ($[M+Na]^+$) : 430.1625, observed 430.1626; **(*trans*-3at) (d.r. 1.5:1)** : white solid; m.p. 190 – 192 °C; ^{1}H NMR (400 MHz, DMSO-d$_6$) δ 8.75 (t, *J* = 8.7 Hz, 1H), 7.42-7.36 (m, 4H), 7.19 (t, *J* = 7.5 Hz, 4H), 4.29 (dp, *J* = 14.5, 7.2 Hz, 1H), 3.98 (d, *J* = 1.9 Hz, 0.6H), 3.91 (d, *J* = 1.8 Hz, 0.4H), 3.77 (d, *J* = 1.8 Hz, 0.6H), 3.72 (d, *J* = 1.8 Hz, 0.4H), 3.61 (s, 1.8H), 3.52 (s, 1.2H), 2.31 (s, 3.6H), 2.30 (s, 2.4H), 1.31 (d, *J* = 7.3 Hz, 1.2H), 1.24 (d, *J* = 7.2 Hz, 1.8H) ^{13}C NMR (100 MHz, DMSO-d$_6$) δ 173.5, 173.4, 173.35, 173.26, 170.4, 170.3, 138.83, 138.80, 138.4, 138.33, 138.33, 138.30, 138.0, 137.9, 131.3, 131.15, 131.13, 131.13, 129.6, 129.6, 129.5, 129.4, 126.53, 126.49, 126.46, 126.4, 52.3, 52.2, 48.2, 48.0, 46.8, 46.7, 46.0, 45.6, 40.6, 21.39, 21.38, 17.6, 17.4; HRMS m/z calculated for $[C_{24}H_{25}NNaO_5]^+$ ($[M+Na]^+$) : 430.1625, observed 430.1625.

3-methyl-1,6,7-tri-*p*-tolyl-3-azabicyclo[3.2.0]hept-6-en-2-one (3av)

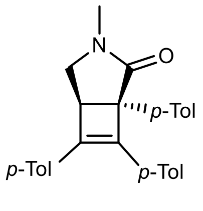

Prepared according to the ***General Procedure A*** using di(*p*-tolyl)acetylene **1a** (0.1 mmol, 1.0 equiv.) and 1-methyl-3-(p-tolyl)-1,5-dihydro-2H-pyrrol-2-one **2v** (1.5 equiv.), 17 mg, 43% yield; white solid; m.p. 170 – 171 °C; ^{1}H NMR (400 MHz, $CDCl_3$) δ 7.68 (d, *J* = 8.2 Hz, 2H), 7.45 (d, *J* = 8.1 Hz, 2H), 7.20 (d, *J* = 8.1 Hz, 2H), 7.17 (d, *J* = 7.9 Hz, 2H), 7.08 – 7.05 (m, 4H), 3.60 (dd, *J* = 10.2, 8.2 Hz, 1H), 3.50 (dd, *J* = 8.2, 1.6 Hz, 1H), 3.30 (dd,

J = 10.2, 1.7 Hz, 1H), 2.38 (s, 3H), 2.30 (s, 3H), 2.28 (s, 3H); ^{13}C NMR (100 MHz, $CDCl_3$) δ 174.2, 143.0, 139.8, 138.6, 138.1, 136.5, 135.5, 130.6, 130.5, 129.5, 129.3, 129.2, 127.8, 127.4, 126.9, 59.0, 48.4, 45.8, 30.8, 21.58, 21.57, 21.2; HRMS *m/z* calculated for $[C_{28}H_{28}NO]^+$ ($[M+H]^+$): 394.2165, observed : 394.2165.

1,2-di-*p*-tolyl-2a,8b-dihydro-3*H*-cyclobuta[*c*]chromen-3-one (3aw)

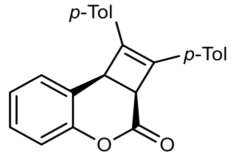

Prepared according to the ***General Procedure A*** using di(*p*-tolyl)acetylene **1a** (0.1 mmol, 1.0 equiv.) and coumarin **2w** (1.5 equiv.), 25 mg, 71% yield; white solid; m.p. 183 – 184 °C; ^{1}H NMR (400 MHz, $CDCl_3$) δ 7.60 (d, J = 8.2 Hz, 2H), 7.30 (d, J = 8.1 Hz, 2H), 7.23 – 7.18 (m, 2H), 7.14 (d, J = 8.0 Hz, 2H), 7.11 (d, J = 8.0 Hz, 2H), 7.05 – 7.01 (m, 2H), 4.45 (d, J = 4.6 Hz, 1H), 4.35 (d, J = 4.6 Hz, 1H), 2.34 (s, 3H), 2.32 (s, 3H); ^{13}C NMR (100 MHz, $CDCl_3$) δ 168.0, 151.1, 144.2, 138.8, 138.6, 136.3, 131.2, 130.4, 129.4, 129.2, 129.1, 128.5, 127.4, 126.8, 124.4, 121.6, 117.6, 42.8, 41.8, 21.5; HRMS *m/z* calculated for $[C_{25}H_{21}O_2]^+$ ($[M+H]^+$): 353.1536, observed : 353.1538.

1-(2-((*tert*-butyldimethylsilyl)oxy)phenyl)-2-(*p*-tolyl)-2a,8b-dihydro-3*H*-cyclobuta[*c*]chromen-3-one (3ww_a)

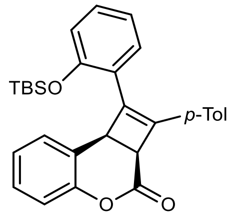

Prepared according to the ***General Procedure A*** using *tert*-butyldimethyl(2-(*p*-tolylethynyl)phenoxy)silane **1w** (0.1 mmol, 1.0 equiv.) and coumarin **2w** (1.5 equiv.), 27 mg, 58% yield; white solid; m.p. 51 – 52 °C; ^{1}H NMR (400 MHz, $CDCl_3$) δ 7.55 (d, J = 8.2 Hz, 2H), 7.23 – 7.13 (m, 3H), 7.08 – 7.06 (m, 2H), 7.03 – 7.01 (m, 2H), 6.96 – 6.87 (m, 3H), 4.59 (d, J = 4.6 Hz, 1H), 4.38 (d, J = 4.6 Hz, 1H), 2.31 (s, 3H), 0.80 (s, 9H), 0.09 (s, 3H), -0.08 (s, 3H); ^{13}C NMR (100 MHz, $CDCl_3$) δ 168.1, 153.6, 150.7, 142.4, 138.5, 137.2, 130.8, 130.1, 129.7, 129.0, 128.4, 128.2, 126.8, 125.9, 124.5, 121.7, 121.4, 120.3, 117.3, 42.6, 42.1, 25.7, 21.5, 18.2, -4.0, -4.6; HRMS *m/z* calculated for $[C_{30}H_{33}O_3Si]^+$ ($[M+H]^+$): 469.2193, observed : 469.2193.

2-(2-((*tert*-butyldimethylsilyl)oxy)phenyl)-1-(*p*-tolyl)-2a,8b-dihydro-3*H*-cyclobuta[*c*]chromen-3-one (3ww_b)

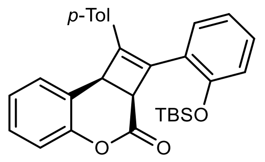

Prepared according to the ***General Procedure A*** using *tert*-butyldimethyl(2-(*p*-tolylethynyl)phenoxy)silane **1w** (0.1 mmol, 1.0 equiv.) and coumarin **2w** (1.5 equiv.), 15 mg, 32% yield; white solid; m.p. 148 – 149 °C; ^{1}H NMR (400 MHz, $CDCl_3$) δ 7.52 (dd, *J* = 7.5, 1.7 Hz, 1H), 7.33 (dd, *J* = 7.6, 1.8 Hz, 1H), 7.28 (d, *J* = 8.2 Hz, 2H), 7.25 – 7.20 (m, 2H), 7.13 (td, *J* = 7.5, 1.3 Hz, 1H), 7.06 – 7.03 (m, 3H), 6.98 (td, *J* = 7.5, 1.1 Hz, 1H), 6.87 (dd, *J* = 8.3, 1.1 Hz, 1H), 4.51 (d, *J* = 4.5 Hz, 1H), 4.39 (d, *J* = 4.5 Hz, 1H), 2.28 (s, 3H), 0.74 (s, 9H), 0.09 (s, 3H), 0.01 (s, 3H); ^{13}C NMR (100 MHz, $CDCl_3$) δ 167.2, 153.8, 151.6, 145.7, 138.5, 135.2, 131.1, 130.3, 129.83, 129.79, 129.0, 128.6, 126.9, 125.4, 124.4, 122.0, 121.3, 119.8, 118.0, 44.6, 41.3, 25.6, 21.5, 18.2, -4.0, -4.5; HRMS *m/z* calculated for $[C_{30}H_{33}O_3Si]^+$ ($[M+H]^+$): 469.2193, observed : 469.2193.

1-(2-aminophenyl)-2-phenyl-2a,8b-dihydro-3*H*-cyclobuta[*c*]chromen-3-one (3xw)

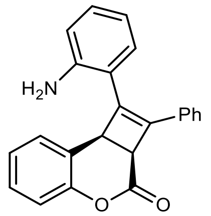

Prepared according to the ***General Procedure A*** using 2-(phenylethynyl)aniline **1x** (0.1 mmol, 1.0 equiv.) and coumarin **2w** (1.5 equiv.), 23 mg, 68% yield; white solid; m.p. 88 – 89 °C; ^{1}H NMR (400 MHz, $CDCl_3$) δ 7.61 – 7.59 (m, 2H), 7.34 – 7.28 (m, 3H), 7.23 – 7.19 (m, 1H), 7.17 – 7.13 (m, 1H), 7.10 – 7.04 (m, 3H), 7.03 – 6.99 (m, 1H), 6.75 (td, *J* = 7.5, 1.1 Hz, 1H), 6.68 (dd, *J* = 8.1, 1.1 Hz, 1H), 4.52 (d, *J* = 4.7 Hz, 1H), 4.50 (d, *J* = 4.6 Hz, 1H), 3.66 (br s, 2H); ^{13}C NMR (100 MHz, $CDCl_3$) δ 167.8, 150.7, 143.9, 142.9, 137.9, 132.9, 129.8, 128.9, 128.8, 128.8, 128.6, 128.5, 127.1, 124.6, 121.2, 119.3, 118.1, 117.5, 115.8, 42.6, 42.5; HRMS *m/z* calculated for $[C_{23}H_{18}NO_2]^+$ ($[M+H]^+$): 340.1332, observed : 340.1331.

6-hydroxy-8b-methyl-1,2-di-*p*-tolyl-2a,8b-dihydro-3*H*-cyclobuta[*c*]chromen-3-one (3ax)

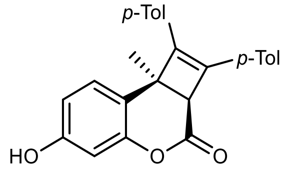

Prepared according to the ***General Procedure A*** using di(*p*-tolyl)acetylene **1a** (0.1 mmol, 1.0 equiv.) and 4-methylumbelliferone **2x** (1.5 equiv.), 29 mg, 76% yield; white solid; m.p. 236 – 237 °C; ^{1}H NMR (400 MHz,

$CDCl_3$) δ 7.43 (d, *J* = 8.2 Hz, 2H), 7.15 – 7.06 (m, 7H), 6.70 (d, *J* = 2.5 Hz, 1H), 6.61 (dd, *J* = 8.5, 2.6 Hz, 1H), 3.98 (s, 1H), 2.36 (s, 3H), 2.30 (s, 3H), 1.71 (s, 3H); ^{13}C NMR (100 MHz, $CDCl_3$) δ 168.9, 156.0, 150.7, 149.0, 138.6, 138.4, 134.7, 131.0, 130.1, 129.4, 129.2, 128.2, 127.5, 126.7, 117.8, 112.1, 104.6, 50.4, 46.3, 24.1, 21.55, 21.52; HRMS *m/z* calculated for $[C_{26}H_{23}O_3]^+$ ($[M+H]^+$): 383.1642, observed : 383.1644.

(8*R*,9*S*,13*S*,14*S*,17*S*)-17-hydroxy-13-methyl-17-(3-methyl-2,4-dioxo-3-azabicyclo[3.2.0]hept-6-en-6-yl)-7,8,9,11,12,13,14,15,16,17-decahydro-6*H*-cyclopenta[*a*]phenanthren-3-yl acetate (3ya)

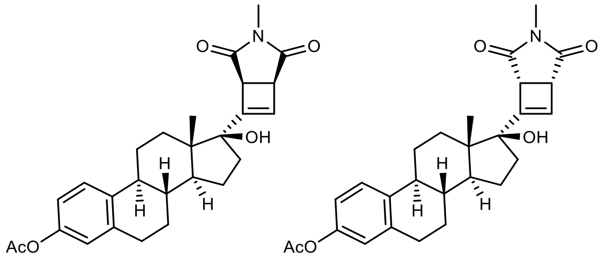

Prepared according to the ***General Procedure A*** using 17α-ethynylestradiol 3-acetate **1y** (0.1 mmol, 1.0 equiv.) and *N*-methylmaleimide **2a** (1.5 equiv.), 30 mg, 66% yield (d.r. 1.4:1); yellow viscous oil; The NMR spectrum was obtained on a partially purified material as a mixture of diastereomers; ^1H NMR (400 MHz, $CDCl_3$) δ 7.23 (t, *J* = 7.7 Hz, 1H), 6.83 (d, *J* = 8.5 Hz, 1H), 6.79 (s, 0.6H), 6.79 (s, 0.4H), 6.19 (d, *J* = 1.0 Hz, 0.6H), 6.18 (d, *J* = 1.0 Hz, 0.4H), 3.98 (d, *J* = 3.2 Hz, 0.4H), 3.87 (d, *J* = 3.2 Hz, 0.6H), 3.73 (dd, *J* = 3.2, 0.9 Hz, 0.6H), 3.65 (dd, *J* = 3.2, 0.9 Hz, 0.4H), 2.99 (s, 1.8H), 2.98 (s, 1.2H), 2.91 – 2.82 (m, 2H), 2.72 (s, 0.4H), 2.49 (s, 0.6H), 2.38 – 2.20 (m, 4H), 2.22 – 2.09 (m, 2H), 2.05 – 1.82 (m, 4H), 1.64 – 1.46 (m, 5H), 1.42 – 1.27 (m, 3H), 0.95 (s, 1.8H), 0.92 (s, 1.2H); ^{13}C NMR (100 MHz, $CDCl_3$) δ 176.05, 175.08, 175.07, 174.8, 169.83, 169.80, 158.7, 157.4, 148.46, 148.46, 138.14, 138.14, 137.6, 137.5, 130.4, 128.5, 126.25, 126.25, 121.52, 121.51, 118.59, 118.58, 84.2, 84.0, 49.7, 48.9, 48.8, 47.75, 47.70, 43.68, 43.65, 42.74, 42.66, 41.4, 39.0, 38.9, 37.5, 36.4, 33.5, 32.0, 29.5, 29.4, 28.2, 27.2, 27.2, 26.1, 26.0, 25.1, 24.8, 23.2, 23.1, 21.1, 14.1, 13.6.; HRMS m/z calculated for $[C_{27}H_{32}NO_5]^+$ ($[M+H^+]$): 450.2275, observed 450.2275.

6-(6-chloro-2-oxo-4-(trifluoromethyl)-1,4-dihydro-2*H*-benzo[*d*][1,3]oxazin-4-yl)-7-cyclopropyl-3-methyl-3-azabicyclo[3.2.0]hept-6-ene-2,4-dione (3za)

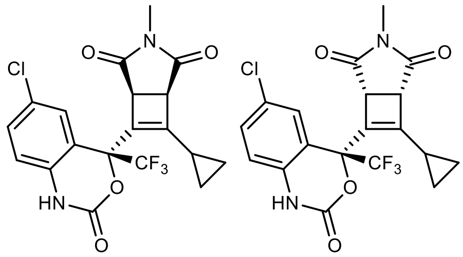

Prepared according to the ***General Procedure A*** using Efavirenz **1z** (0.1 mmol, 1.0 equiv.) and *N*-methylmaleimide **2a** (15.0 equiv.), 19 mg, 45% yield (d.r. = 2.3:1); yellow viscous oil; The NMR spectrum was obtained on a partially purified material as a mixture of diastereomers; ^{1}H NMR (400 MHz, $CDCl_3$) δ 9.28 (s, 1H), 7.39 – 7.35 (m, 1.3H), 7.34 (d, *J* = 2.2 Hz, 0.3H), 7.29 (s, 0.3H), 6.89 (dd, *J* = 8.5, 1.3 Hz, 0.3H), 6.85 (d, *J* = 8.4 Hz, 0.7H), 3.69 (d, *J* = 3.4 Hz, 0.7H), 3.56 (d, *J* = 3.5 Hz, 0.3H), 3.48 (d, *J* = 3.4 Hz, 0.3H), 3.45 (d, *J* = 3.4 Hz, 0.7H), 2.92 (s, 0.9H), 2.90 (s, 2.1H), 1.82 – 1.66 (m, 2H), 1.34 – 1.20 (m, 1H), 1.05 – 0.80 (m, 3H) ; ^{13}C NMR (100 MHz, $CDCl_3$) 173.6, 173.2, 173.2, 173.0, 155.3, 154.3, 148.5, 148.3, 134.1, 133.9, 131.6, 131.5, 130.9, 130.4, 129.2, 128.9, 126.8, 126.6, 116.7, 116.6, 114.1, 114.0, 77.3, 77.2, 43.7, 43.6, 43.0, 42.9, 25.1, 24.9, 10.8, 10.6, 7.9, 7.6, 7.0, 6.9.; HRMS *m/z* calculated for $[C_{19}H_{14}ClF_3N_2NaO_4]^+$ ($[M+Na]^+$): 449.0486, observed 449.0485.

General procedure and characterization of 1,3-dienes

General Procedure B (for the synthesis of 1,3-diene)

Enyne (0.1 mmol, 1.0 equiv.), and photocatalyst $Ir[dF(CF_3)ppy]_2(dtbbpy)PF_6$ (2.5 mol%) were added to an oven-dried 4 mL vial equipped with a stir bar. The combined materials were dissolved in CH_2Cl_2 (2 mL) under argon atmosphere in glovebox. The reaction mixture was then irradiated by 12 W blue LED strip at room temperature (maintained with a cooling fan). After completion of the reaction as indicated by TLC, the solution was concentrated under reduced pressure. The residue was purified by flash column chromatography on silica gel to give the desired product.

4-(1,2-di-*p*-tolylvinyl)-2*H*-chromen-2-one (6a)

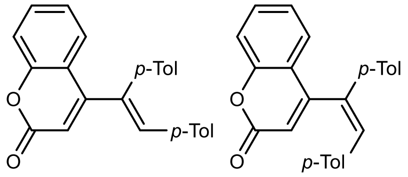

(*E*)-6a **(*Z*)-6a**

Prepared according to the ***General Procedure B*** using 2-(*p*-tolylethynyl)phenyl *(E)*-3-(*p*-tolyl)acrylate **4a** (0.1 mmol, 1.0 equiv.), 26 mg, 73% yield, (*E/Z* 1:1); ***(E)*-6a**: pale yellow solid; m.p. 144 – 146 °C; ^{1}H NMR (400 MHz, $CDCl_3$) δ 7.53 (dd, *J* = 8.0, 1.5 Hz, 1H), 7.45 (ddd, *J* = 8.7, 7.3, 1.6 Hz, 1H), 7.34 (dd, *J* = 8.3, 1.2 Hz, 1H), 7.16 – 7.06 (m, 7H), 7.03 (d, *J* = 8.2 Hz, 2H), 6.83 (s, 1H), 6.41 (s, 1H), 2.33 (s, 3H), 2.32 (s, 3H); ^{13}C NMR (100 MHz, $CDCl_3$) δ 161.2, 157.8, 154.2, 138.3, 138.2, 136.2, 135.0, 133.0, 132.8, 131.6, 129.8, 129.8, 129.4, 129.1, 127.3, 124.1, 119.2, 117.3, 115.7, 21.45, 21.45; HRMS m/z calculated for $[C_{25}H_{21}O_2]$+ ($[M+H]^+$): 353.1536, observed : 353.1536; **(*Z*)-6a** : pale yellow solid; m.p. 76 – 77 °C; ^{1}H NMR (400 MHz, $CDCl_3$) δ 7.49 (t, *J* = 7.8 Hz, 1H), 7.47 – 7.36 (m, 2H), 7.31 (d, *J* = 8.2 Hz, 2H), 7.23 (s, 1H), 7.15 (d, *J* = 8.4 Hz, 2H), 7.14 – 7.09 (m,

1H), 7.05 (d, J = 8.1 Hz, 2H), 6.98 (d, J = 8.2 Hz, 2H), 6.35 (s, 1H), 2.35 (s, 3H), 2.26 (s, 3H); ^{13}C NMR (100 MHz, $CDCl_3$) 160.9, 155.5, 154.3, 138.4, 138.1, 137.2, 134.6, 133.0, 132.1, 130.9, 129.7, 129.4, 128.9, 127.1, 126.3, 124.7, 119.2, 117.4, 117.2, 21.32, 21.28; HRMS m/z calculated for $[C_{25}H_{21}O_2]^+$ ($[M+H]^+$): 353.1536, observed : 353.1536

4-(2-(*p*-tolyl)-1-(trimethylsilyl)vinyl)-2*H*-chromen-2-one (6b)

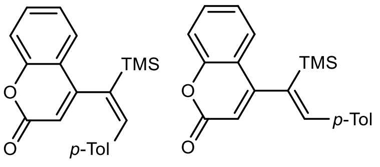

(*E*)-6b **(*Z*)-6b**

Prepared according to the ***General Procedure B*** using 2-((trimethylsilyl)ethynyl)phenyl *(E)*-3-(*p*-tolyl)acrylate **4b** (0.1 mmol, 1.0 equiv.), 20 mg, 60% yield (*E/Z* 1.2:1); white solid; m.p. 103 – 116 °C; The NMR spectrum was obtained on a partially purified material as a mixture of *E/Z* isomers; ^{1}H NMR (400 MHz, $CDCl_3$) δ 7.59 (dd, J = 7.9, 1.6 Hz, 0.4H), 7.54 (ddd, J = 8.6, 7.3, 1.6 Hz, 0.4H), 7.49 (ddd, J = 8.6, 7.2, 1.6 Hz, 0.6H), 7.43 (dd, J = 7.9, 1.6 Hz, 0.6H), 7.37 (m, 1H), 7.32 – 7.24 (m, 1.6H), 7.20 (d, J = 7.8 Hz, 0.8H), 7.13 (t, J = 7.6 Hz, 0.6H), 7.04 (d, J = 8.0 Hz, 1.2H), 7.00 (s, 0.6H), 6.94 (d, J = 8.0 Hz, 1.2H), 6.16 (s, 0.4H), 6.13 (s, 0.6H), 2.39 (s, 1.2H), 2.22 (s, 1.8H), 0.16 (s, 5.4H), 0.01 (s, 3.6H); ^{13}C NMR (100 MHz, $CDCl_3$) δ 161.5, 161.1, 160.9, 158.9, 153.8, 153.7, 146.4, 140.1, 140.0, 138.4, 138.1, 138.0, 135.2, 133.3, 132.0, 131.8, 129.2, 128.9, 128.9, 128.4, 126.9, 126.7, 124.2, 124.0, 120.1, 118.2, 117.2, 117.2, 112.1, 111.8, 21.3, 21.1, 0.3, -1.5.; HRMS *m/z* calculated for $[C_{21}H_{23}O_2Si]^+$ ($[M+H]^+$): 335.1462, observed 335.1462.

4-(1,2-di-*p*-tolylvinyl)-1-ethylquinolin-2(1*H*)-one (6c)

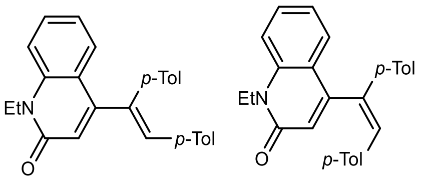

(*E*)-6c **(*Z*)-6c**

Prepared according to the ***General Procedure B*** using *(E)*-*N*-ethyl-3-(*p*-tolyl)-*N*-(2-(*p*-tolylethynyl)phenyl)acrylamide **4c** (0.1 mmol, 1.0 equiv.), 30 mg, 80% yield, (*E/Z* 2:1); **(*E*)-6c :** yellow solid; m.p. 168 – 169 °C; ^{1}H NMR (400 MHz, $CDCl_3$) δ 7.74 (dd, J = 8.1, 1.5 Hz, 1H), 7.49 (ddd, J = 8.6, 7.1, 1.6 Hz, 1H), 7.39 (d, J = 8.1 Hz, 1H), 7.16 (d, J = 8.1 Hz, 2H), 7.10 – 6.99 (m, 7H), 6.74 (s, 1H), 6.73 (s, 1H), 4.39 (q, J = 7.1 Hz, 2H), 2.31 (s, 3H), 2.30 (s, 3H), 1.39 (t, J = 7.1 Hz, 3H); ^{13}C NMR (100 MHz, $CDCl_3$) δ 161.7, 152.8, 139.3, 137.6, 137.5, 137.4, 135.8, 133.5, 131.5, 130.3, 129.4, 129.4, 129.2, 128.9, 128.0, 121.7, 121.6, 120.7, 114.2, 37.3, 21.25, 21.25, 12.8.; HRMS *m/z* calculated for $[C_{27}H_{25}NNaO]^+$ ($[M+Na]^+$): 402.1828, observed :

402.1820; ***(Z)*-6c :** white solid; m.p. 147 – 149 °C; ^{1}H NMR (400 MHz, $CDCl_3$) δ 7.61 – 7.39 (m, 3H), 7.33 – 7.23 (m, 3H), 7.18 (s, 1H), 7.10 (d, *J* = 7.6 Hz, 2H), 7.05 (t, *J* = 7.4 Hz, 1H), 6.98 (d, *J* = 7.8 Hz, 2H), 6.90 (d, *J* = 7.7 Hz, 2H), 6.63 (s, 1H), 4.49 (dq, *J* = 14.1, 7.1 Hz, 1H), 4.36 (dq, *J* = 14.1, 7.1 Hz, 1H), 2.32 (s, 3H), 2.22 (s, 3H), 1.43 (t, *J* = 7.1 Hz, 3H); ^{13}C NMR (100 MHz, $CDCl_3$) δ 161.7, 149.8, 139.3, 138.0, 137.8, 137.3, 136.0, 133.3, 130.6, 129.9, 129.3, 129.0, 128.8, 127.8, 126.1, 122.6, 122.1, 120.6, 114.3, 37.3, 21.12, 21.10, 12.9.; HRMS *m/z* calculated for $[C_{27}H_{25}NNaO]^+$ ($[M+Na]^+$): 402.1828, observed : 402.1820.

4-(2-(2-bromophenyl)-1-phenylvinyl)-1-ethylquinolin-2(1*H*)-one (6d)

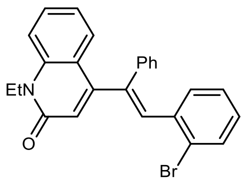

(*E*)-6d

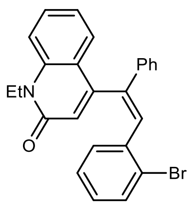

(*Z*)-6d

Prepared according to the ***General Procedure B*** using *(E)*-3-(2-bromophenyl)-*N*-ethyl-*N*-(2-(phenylethynyl)phenyl)acrylamide **4d** (0.1 mmol, 1.0 equiv.). 32 mg, 74% yield (*E/Z* 1.8:1); yellow solid; The NMR spectrum was obtained on a partially purified material as a mixture of *E/Z* isomers; ^{1}H NMR (400 MHz, $CDCl_3$) δ 7.70 (dd, *J* = 8.1, 1.3 Hz, 0.35H), 7.59 (t, *J* = 7.1 Hz, 1H), 7.54 – 7.48 (m, 1.35H), 7.46 – 7.30 (m, 3.35H), 7.20 – 7.13 (m, 3H), 7.11 – 7.00 (m, 3H), 7.01 – 6.92 (m, 0.65H), 6.93 – 6.87 (m, 0.35H), 6.87 (s, 0.65H), 6.83 (s, 0.65H), 6.63 (s, 0.35H), 4.50 – 4.25 (m, 2H), 1.40 (m, 1.45 –1.35, 3H); ^{13}C NMR (100 MHz, $CDCl_3$) δ 161.6, 161.4, 151.7, 148.5, 140.1, 140.0, 139.3, 139.1, 139.0, 137.7, 137.2, 136.6, 132.59, 132.55, 131.4, 131.3, 130.6, 130.4, 130.3, 129.6, 129.3, 129.0, 128.9, 128.7, 128.5, 128.4, 127.99, 127.98, 127.8, 126.94, 126.93, 126.7, 124.7, 124.6, 123.2, 122.1, 122.0, 121.8, 120.7, 120.5, 114.4, 114.3, 37.4, 37.4, 12.9, 12.8; HRMS m/z calculated for $[C_{25}H_{21}BrNO]^+$ ($[M+H]^+$): 430.0801, observed 430.0799.

4-(1,2-di-*p*-tolylvinyl)-1-phenylquinolin-2(1*H*)-one (6e)

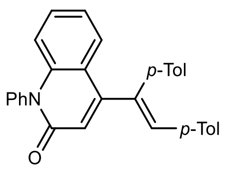

(*E*)-6e

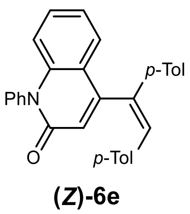

(*Z*)-6e

Prepared according to the ***General Procedure B*** using *(E)*-*N*-phenyl-3-(*p*-tolyl)-*N*-(2-(*p*-tolylethynyl)phenyl)acrylamide **4e** (0.1 mmol, 1.0 equiv.), 40 mg, 94% yield (*E/Z* 1.5:1); ***(E)*-6e** : white solid; m.p. 142 – 144 °C; ^{1}H NMR (400 MHz, $CDCl_3$) δ 7.77 (d, *J* = 8.0 Hz, 1H), 7.62 (m, 2H), 7.53 (m, 1H), 7.33 (d, *J* = 7.6 Hz, 2H), 7.29 – 7.21 (m, 3H), 7.10 (t, *J* = 7.4 Hz, 4H), 7.05 (t, *J* = 8.1 Hz, 3H), 6.83 (s, 2H), 6.67 (d, *J* = 8.5

Hz, 1H), 2.34 (s, 3H), 2.33 (s, 3H); ^{13}C NMR (100 MHz, $CDCl_3$) δ 162.2, 153.8, 141.4, 137.8, 137.7, 137.5, 137.5, 135.8, 133.4, 131.7, 130.2, 129.9, 129.5, 129.5, 129.3, 128.9, 128.9, 128.9, 127.4, 122.0, 122.0, 120.1, 116.3, 21.3, 21.3; HRMS *m/z* calculated for $[C_{31}H_{26}NO]^+$ ($[M+H]^+$): 428.2009, observed 428.2009; **(*Z*)-6e** : white solid; m.p. 144 – 146 °C; ^{1}H NMR (400 MHz, $CDCl_3$) δ 7.67 – 7.51 (m, 4H), 7.40 – 7.34 (m, 4H), 7.28 (ddd, *J* = 8.7, 7.1, 1.5 Hz, 1H), 7.24 (s, 1H), 7.15 (d, *J* = 8.0 Hz, 2H), 7.07 (d, *J* = 8.1 Hz, 2H), 7.03 (t, *J* = 7.6 Hz, 1H), 6.97 (d, *J* = 8.0 Hz, 2H), 6.71 (m, 2H), 2.36 (s, 3H), 2.27 (s, 3H); ^{13}C NMR (100 MHz, $CDCl_3$) δ 162.1, 150.8, 141.4, 137.9, 137.9, 137.7, 137.5, 136.0, 133.3, 130.2, 130.2, 130.2, 130.1, 129.4, 129.1, 129.1, 129.0, 128.9, 128.8, 127.2, 126.2, 123.0, 122.5, 120.1, 116.3, 21.2, 21.1.; HRMS *m/z* calculated for $[C_{31}H_{26}NO]^+$ ($[M+H]^+$): 428.2009, observed 428.2009.

1-ethyl-4-(1-(pyridin-2-yl)-2-(*p*-tolyl)vinyl)quinolin-2(1*H*)-one (6f)

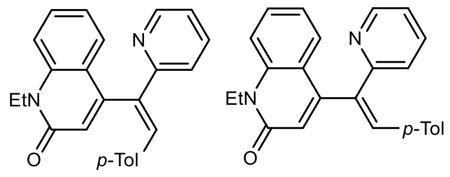

(*E*)-6f **(*Z*)-6f**

Prepared according to the ***General Procedure B*** using *(E)*-*N*-ethyl-*N*-(2-(pyridin-2-ylethynyl)phenyl)-3-(*p*-tolyl)acrylamide **4f** (0.1 mmol, 1.0 equiv.), 25 mg, 69% yield (*E/Z* 1:2.8); ***(E)*-6f :** yellow oil; ^{1}H NMR (400 MHz, $CDCl_3$) δ 8.67 (ddd, *J* = 4.8, 1.9, 0.9 Hz, 1H), 8.12 (s, 1H), 7.60 – 7.52 (m, 2H), 7.52 – 7.45 (m, 2H), 7.15 (ddd, *J* = 7.5, 4.8, 1.1 Hz, 1H), 7.11 – 7.02 (m, 3H), 6.97 (dt, *J* = 8.0, 1.0 Hz, 1H), 6.93 (d, *J* = 8.0 Hz, 2H), 6.70 (s, 1H), 4.53 (dq, *J* = 14.3, 7.2 Hz, 1H), 4.40 (dq, *J* = 14.0, 7.1 Hz, 1H), 2.24 (s, 3H) 1.46 (t, *J* = 7.1 Hz, 3H); ^{13}C NMR (100 MHz, $CDCl_3$) δ 161.7, 156.6, 149.5, 149.0, 139.4, 138.2, 136.7, 133.9, 132.7, 130.9, 129.5, 129.1, 127.5, 123.0, 122.3, 121.4, 120.6, 114.4, 37.5, 21.2, 13.0; HRMS *m/z* calculated for $[C_{25}H_{23}N_2O]^+$ ($[M+H]^+$): 367.1805, observed : 367.1805; **(*Z*)-6f:** yellow oil; ^{1}H NMR (400 MHz, $CDCl_3$) δ 8.59 (ddd, *J* = 4.9, 1.7, 0.9 Hz, 1H), 7.81 (dd, *J* = 8.1, 1.4 Hz, 1H), 7.56 (td, *J* = 7.7, 1.8 Hz, 1H), 7.50 (ddd, *J* = 8.6, 7.1, 1.5 Hz, 1H), 7.39 (d, *J* = 8.4 Hz, 1H), 7.29 (d, *J* = 7.9 Hz, 1H), 7.15 (ddd, *J* = 7.5, 4.9, 1.1 Hz, 1H), 7.10 – 7.05 (m, 1H), 7.04 – 6.97 (m, 4H), 6.96 (s, 1H), 6.75 (s, 1H), 4.39 (q, *J* = 7.1 Hz, 2H), 2.31 (s, 3H), 1.39 (t, *J* = 7.1 Hz, 3H); ^{13}C NMR (100 MHz, $CDCl_3$) δ 161.6, 158.0, 151.6, 150.0, 139.3, 138.0, 137.3, 136.3, 134.6, 132.8, 130.3, 129.5, 128.9, 128.1, 125.0, 122.4, 122.0, 121.7, 120.7, 114.2, 37.3, 21.2, 12.7; HRMS *m/z* calculated for $[C_{25}H_{22}N_2NaO]^+$ ($[M+Na]^+$): 389.1624, observed : 389.1624.

1-ethyl-4-(1-(isoquinolin-4-yl)-2-(*p*-tolyl)vinyl)quinolin-2(1*H*)-one (6g)

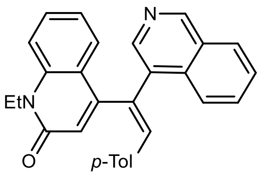

(*E*)-6g

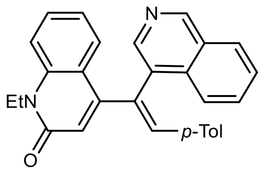

(*Z*)-6g

Prepared according to the ***General Procedure B*** using *(E)*-*N*-ethyl-*N*-(2-(isoquinolin-1-ylethynyl)phenyl)-3-(*p*-tolyl)acrylamide **6g** (0.1 mmol, 1.0 equiv.), 34 mg, 82% yield (*E/Z* 1.5:1); yellow oil; The NMR spectrum was obtained on a partially purified material as a mixture of *E/Z* isomers; ^{1}H NMR (400 MHz, $CDCl_3$) δ 9.23 (s, 1H), 9.17 (s, 0.7H), 8.46 (s, 0.7H), 8.43 (s, 1H), 8.31 (d, *J* = 8.4 Hz, 0.7H), 8.19 (d, *J* = 8.1 Hz, 1.1H), 8.03 – 8.00 (m, 1.7H), 7.84 (d, *J* = 7.8 Hz, 1.1H), 7.78 (d, *J* = 8.0 Hz, 0.8H), 7.73 – 7.42 (m, 7.4H), 7.21 – 7.18 (m, 2.1H), 7.14 (s, 0.7H), 7.08 (d, *J* = 8.0 Hz, 1.4H), 7.02 (t, *J* = 7.5 Hz, 0.7H), 6.96 (d, *J* = 7.9 Hz, 1.4H), 6.88 – 6.82 (m, 4.1H), 6.71 (s, 0.7H), 6.57 (s, 1H), 4.53 – 4.28 (m, 4H), 2.26 (s, 2.1H), 2.21 (s, 3H), 1.41 – 1.35 (m, 5.2H); ^{13}C NMR (100 MHz, $CDCl_3$) δ 161.7, 161.6, 152.7, 152.5, 152.0, 150.1, 144.2, 142.8, 139.8, 139.6, 138.4, 138.3, 137.5, 137.2, 134.1, 134.0, 133.7, 133.6, 132.9, 132.8, 131.3, 131.21, 131.17, 131.1, 130.9, 130.8, 129.4, 129.3, 129.2, 129.2, 129.0, 128.8, 128.51, 128.46, 127.6, 127.53, 127.52, 127.4, 124.6, 124.4, 122.9, 122.2, 122.1, 121.2, 120.5, 119.8, 114.8, 114.7, 37.6, 37.5, 21.4, 21.3, 13.0, 12.9; HRMS *m/z* calculated for $[C_{29}H_{25}N_2O]^+$ ($[M+H]^+$): 417.1961, observed : 417.1961.

1-ethyl-4-(1-(*p*-tolyl)hex-1-en-2-yl)quinolin-2(1*H*)-one (6h)

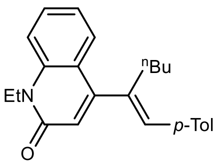

(*E*)-6h

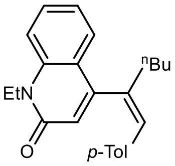

(*Z*)-6h

Prepared according to the ***General Procedure B*** using *(E)*-*N*-ethyl-*N*-(2-(hex-1-yn-1-yl)phenyl)-3-(*p*-tolyl)acrylamide **4h** (0.1 mmol, 1.0 equiv.), 27 mg, 78% yield (*E/Z* 1.7:1); yellow oil; The NMR spectrum was obtained on a partially purified material as a mixture of *E/Z* isomers; ^{1}H NMR (400 MHz, $CDCl_3$) δ 7.78 (dd, *J* = 8.0, 1.5 Hz, 0.7H), 7.65 (dd, *J* = 8.0, 1.5 Hz, 1H), 7.59 – 7.52 (m, 1.9H), 7.44 – 7.41 (m, 1.9H), 7.28 – 7.26 (m, 0.6H), 7.22 – 7.19 (m, 1.9H), 7.13 (ddd, *J* = 8.1, 7.0, 1.1 Hz, 1.3H), 6.90 (d, *J* = 8.3 Hz, 2.2H), 6.86 (d, *J* = 8.2 Hz, 2.2H), 6.63 – 6.62 (m, 1.7H), 6.52 (s, 1H), 6.49 (s, 0.7H), 4.53 – 4.27 (m, 4.1H), 2.66 – 2.62 (m, 1.3H), 2.52 – 2.38 (m, 4.1H), 2.19 (s, 3H), 1.52 – 1.26 (m, 13H), 0.89 (t, *J* = 7.1 Hz, 3H), 0.82 (t, *J* = 7.2 Hz, 2H); ^{13}C NMR (100 MHz, $CDCl_3$) δ 161.9, 161.8, 153.3, 151.6, 139.4, 139.3, 139.0, 137.1, 136.9, 136.7, 134.0, 133.4, 130.8, 130.7, 130.6, 129.2, 129.0, 128.9, 128.8, 128.3, 127.6, 127.3, 122.1, 121.8, 121.0, 120.6, 120.1, 119.9, 114.5, 114.5, 40.0, 37.42, 37.39, 32.2, 30.7, 30.4, 22.9, 22.5, 21.4, 21.2, 14.0, 13.9, 13.0, 12.9; HRMS *m/z* calculated for $[C_{24}H_{28}NO]^+$ ($[M+H]^+$): 346.2165, observed : 346.2165.

1-ethyl-4-(2-(pyridin-2-yl)-1-(*p*-tolyl)vinyl)quinolin-2(1*H*)-one (6i)

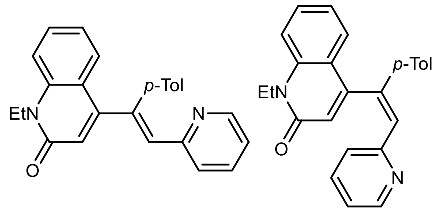

(*E*)-6i **(*Z*)-6i**

Prepared according to the ***General Procedure B*** using (*E*)-*N*-ethyl-3-(pyridin-2-yl)-*N*-(2-(*p*-tolylethynyl)phenyl)acrylamide **4i** (0.1 mmol, 1.0 equiv.), 27 mg, 74% yield (*E/Z* 1.3:1); **(*E*)-6i :** colorless oil; ^{1}H NMR (400 MHz, $CDCl_3$) δ 8.60 – 8.59 (m, 1H), 7.71 (dd, *J* = 8.1, 1.5 Hz, 1H), 7.49 (ddd, *J* = 8.6, 7.1, 1.5 Hz, 1H), 7.45 – 7.38 (m, 2H), 7.15 – 7.01 (m, 7H), 6.89 (s, 1H), 6.78 (s, 1H), 4.40 (q, *J* = 7.1 Hz, 2H), 2.30 (s, 3H), 1.40 (t, *J* = 7.1 Hz, 3H); ^{13}C NMR (100 MHz, $CDCl_3$) δ 161.7, 156.0, 152.1, 149.7, 141.8, 139.4, 138.4, 135.7, 135.1, 131.8, 130.6, 129.6, 129.2, 128.1, 124.5, 122.0, 121.9, 121.8, 120.4, 114.4, 37.5, 21.4, 12.9; HRMS *m/z* calculated for $[C_{25}H_{23}N_2O]^+$ ($[M+H]^+$): 367.1805, observed : 367.1805; **(*Z*)-6i :** white solid; m.p. 131 – 133 °C; ^{1}H NMR (400 MHz, $CDCl_3$) δ 8.46 – 8.44 (m, 1H), 7.59 – 7.51 (m, 2H), 7.45 (d, *J* = 8.5 Hz, 1H), 7.40 – 7.30 (m, 4H), 7.13 (d, *J* = 8.0 Hz, 2H), 7.08 – 7.04 (m, 1H), 6.99 – 6.94 (m, 2H), 6.64 (s, 1H), 4.50 (dq, *J* = 14.2, 7.1 Hz, 1H), 4.38 (dq, *J* = 14.1, 7.1 Hz, 1H), 2.33 (s, 3H), 1.44 (t, *J* = 7.1 Hz, 3H); ^{13}C NMR (100 MHz, $CDCl_3$) δ 161.7, 155.1, 149.6, 149.5, 140.1, 139.4, 138.8, 137.1, 136.0, 130.9, 130.0, 129.6, 127.8, 126.6, 123.2, 122.3, 122.2, 121.8, 120.6, 114.5, 37.5, 21.3, 13.1; HRMS *m/z* calculated for $[C_{25}H_{23}N_2O]^+$ ($[M+H]^+$): 367.1805, observed : 367.1805

1-ethyl-4-(2-(furan-2-yl)-1-(*p*-tolyl)vinyl)quinolin-2(1*H*)-one (6j)

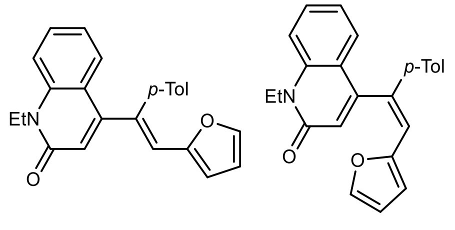

(*E*)-6j **(*Z*)-6j**

Prepared according to the ***General Procedure B*** using (*E*)-*N*-ethyl-3-(furan-2-yl)-*N*-(2-(*p*-tolylethynyl)phenyl)acrylamide **4j** (0.1 mmol, 1.0 equiv.), 36 mg, >99% yield (*E/Z* 1.2:1); brown viscous oil; **(*E*)-6j :** ^{1}H NMR (400 MHz, $CDCl_3$) δ 7.79 – 7.70 (m, 1H), 7.54 – 7.48 (m, 1H), 7.40 – 7.38 (m, 1H), 7.32 – 7.28 (m, 3H), 7.15 – 7.08 (m, 3H), 6.71 (s, 1H), 6.62 (s, 1H), 6.32 – 6.31 (m, 1H), 6.11 – 6.10 (m, 1H), 4.38 (q, *J* = 7.1 Hz, 2H), 2.35 (s, 3H), 1.38 (t, *J* = 7.1 Hz, 3H); ^{13}C NMR (100 MHz, $CDCl_3$) δ 161.8, 152.4, 151.8, 142.3, 139.4, 138.1, 136.02, 135.96, 130.5, 129.4, 129.0, 128.1, 121.84, 121.75, 120.7, 120.2, 114.4, 111.6, 110.9, 37.5, 21.5,

12.9; HRMS *m/z* calculated for $[C_{24}H_{22}NO_2]^+$ ($[M+H]^+$): 356.1645, observed : 356.1645; ***(Z)*-6j :** ^{1}H NMR (400 MHz, $CDCl_3$) δ 7.55 – 7.45 (m, 3H), 7.31 – 7.29 (m, 2H), 7.19 – 7.03 (m, 5H), 6.70 (s, 1H), 6.20 – 6.19 (m, 1H), 5.87 (m, 1H), 4.57 – 4.35 (m, 2H), 2.32 (s, 3H), 1.46 (t, *J* = 6.8 Hz, 3H); ^{13}C NMR (100 MHz, $CDCl_3$) δ 162.0, 151.9, 149.7, 142.5, 139.5, 138.2, 136.6, 133.9, 130.8, 129.6, 127.4, 126.0, 122.3, 122.3, 120.4, 117.0, 114.5, 111.8, 110.6, 37.6, 21.3, 13.1; HRMS *m/z* calculated for $[C_{24}H_{22}NO_2]^+$ ($[M+H]^+$): 356.1645, observed : 356.1645.

Methyl 3-(1-ethyl-2-oxo-1,2-dihydroquinolin-4-yl)-3-(*p*-tolyl)acrylate (6k)

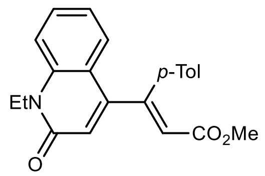

(*E*)-6k

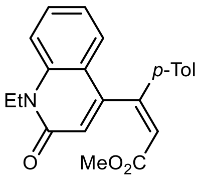

(*Z*)-6k

Prepared according to the ***General Procedure B*** using methyl (*Z*)-4-(ethyl(2-(*p*-tolylethynyl)phenyl)amino)-4-oxobut-2-enoate **4k** (0.1 mmol, 1.0 equiv.), 21 mg, 60% yield, (*E/Z* 1.5:1); colorless oil; ***(E)*-6k:** ^{1}H NMR (400 MHz, $CDCl_3$) δ 7.57 (d, *J* = 8.0 Hz, 1H), 7.51 (t, *J* = 7.8 Hz, 1H), 7.39 (d, *J* = 8.6 Hz, 1H), 7.27 (d, *J* = 8.7 Hz, 2H), 7.13 (d, *J* = 8.0 Hz, 2H), 7.08 (d, *J* = 7.8 Hz, 2H), 6.69 (s, 1H), 6.13 (s, 1H), 4.36 (q, *J* = 7.1 Hz, 2H), 3.71 (s, 3H), 2.23 (s, 3H), 1.39 (t, *J* = 7.1 Hz, 3H); ^{13}C NMR (100 MHz, $CDCl_3$) δ 166.1, 161.2, 151.9, 150.4, 139.4, 139.2, 133.6, 130.7, 128.9, 128.6, 127.6, 121.9, 121.4, 120.6, 119.7, 114.4, 51.6, 37.4, 21.3, 12.7; HRMS *m/z* calculated for $[C_{22}H_{22}NO_3]^+$ ($[M+H]^+$): 348.1594, observed : 348.1590 ***(Z)*-6k:** ^{1}H NMR (400 MHz, $CDCl_3$) δ 7.51 (ddd, *J* = 8.6, 7.1, 1.5 Hz, 1H), 7.43 (d, *J* = 8.9 Hz, 1H), 7.39 (dd, *J* = 8.0, 1.5 Hz, 1H), 7.34 (d, *J* = 8.3 Hz, 2H), 7.13 (d, *J* = 8.6 Hz, 2H), 7.07 (ddd, *J* = 8.1, 7.1, 1.1 Hz, 1H), 6.62 (s, 1H), 6.55 (s, 1H), 4.52 – 4.34 (m, 2H), 2.34 (s, 3H), 1.39 (t, *J* = 7.2 Hz, 3H); ^{13}C NMR (100 MHz, $CDCl_3$) δ 165.3, 161.4, 151.6, 148.6, 140.7, 138.9, 134.4, 130.5, 129.6, 127.1, 126.9, 122.0, 120.4, 120.3, 117.8, 114.4, 51.5, 37.5, 21.3, 12.9; HRMS *m/z* calculated for $[C_{22}H_{22}NO_3]^+$ ($[M+H]^+$): 348.1594, observed : 348.1594.

3-(1-ethyl-2-oxo-1,2-dihydroquinolin-4-yl)-*N,N*-diisopropyl-3-(*p*-tolyl)acrylamide (6l)

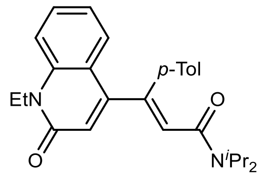

(*E*)-6l

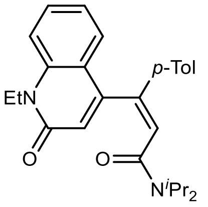

(*Z*)-6l

Prepared according to the ***General Procedure B*** using *N*[1]-ethyl-*N*[4],*N*[4]-diisopropyl-*N*[1]-(2-(*p*-tolylethynyl)phenyl)maleamide **4l** (0.1 mmol, 1.0 equiv.), 34 mg, 82% yield (*E/Z* 1:2.4); yellow oil; The NMR

spectrum was obtained on a partially purified material as a mixture of *E/Z* isomers; ^{1}H NMR (400 MHz, $CDCl_3$) δ 7.52 – 7.27 (m, 7.52H), 7.14 – 7.01 (m, 4.92H), 6.77 (s, 1H), 6.76 (s, 0.5H), 6.59 (s, 0.5H), 6.21 (s, 1H), 4.43 – 4.35 (m, 3H), 4.21 – 4.14 (m, 1.5H), 3.46 – 3.32 (m, 1.5H), 2.33 (s, 1.5H), 2.30 (s, 3H), 1.47 – 1.36 (m, 11H), 1.26 – 1.20 (m, 5.3H), 1.13 – 1.10 (m, 1.3H), 0.85 (d, *J* = 6.7 Hz, 6H); ^{13}C NMR (100 MHz, $CDCl_3$) δ 166.9, 165.9, 161.7, 161.4, 150.8, 148.6, 142.9, 140.1, 139.2, 139.1, 139.1, 138.9, 135.7, 134.3, 130.7, 130.5, 129.7, 129.3, 128.4, 128.4, 128.3, 127.7, 126.5, 126.5, 125.5, 122.0, 122.0, 121.5, 121.4, 120.7, 120.2, 114.3, 53.5, 50.6, 45.9, 45.6, 37.5, 37.4, 30.2, 29.8, 21.4, 21.3, 20.6, 20.3, 13.0, 12.9; HRMS *m/z* calculated for $[C_{27}H_{33}N_2O_2]^+$ ($[M+H]^+$): 417.2537, observed : 417.2532.

1-ethyl-4-(2-phenyl-1-(*p*-tolyl)prop-1-en-1-yl)quinolin-2(1*H*)-one (6m)

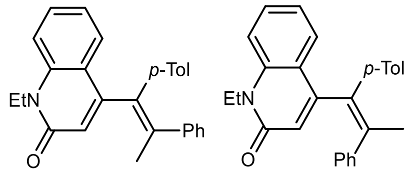

(*E*)-6m **(*Z*)-6m**

Prepared according to the ***General Procedure B*** using (*E*)-*N*-ethyl-3-(*p*-tolyl)-*N*-(2-(*p*-tolylethynyl)phenyl)but-2-enamide **4m** (0.1 mmol, 1.0 equiv.), 16 mg, 43% yield (*E/Z* = 1.2:1); yellow solid; m.p. 86 – 88 °C; The NMR spectrum was obtained on a partially purified material as a mixture of *E/Z* isomers; ^{1}H NMR (400 MHz, $CDCl_3$) δ 7.82 (d, *J* = 7.9 Hz, 1H), 7.54 (t, *J* = 7.8 Hz, 0.6H), 7.46 – 7.37 (m, 1H), 7.34 – 7.02 (m, 8H), 6.92 (d, *J* = 8.0 Hz, 1.2H), 6.84 (d, *J* = 8.0 Hz, 1.2H), 6.75 (s, 0.6H), 6.49 (s, 0.4H), 4.50 – 4.29 (m, 1.6H), 4.14 (dq, *J* = 14.1, 7.1 Hz, 0.4H), 2.32 (s, 1.2H), 2.27 (s, 1.2H), 2.17 (s, 1.8H), 2.02 (s, 1.8H), 1.41 (t, *J* = 7.1 Hz, 1.8H), 1.26 (t, *J* = 7.1 Hz, 1.2H); ^{13}C NMR (100 MHz, $CDCl_3$) δ 161.9, 161.6, 152.0, 151.8, 143.1, 142.6, 139.4, 139.1, 138.9, 137.8, 137.4, 137.00, 136.97, 136.2, 134.4, 133.7, 130.5, 130.0, 129.6, 129.1, 129.05, 129.01, 128.6, 128.1, 128.08, 128.04, 127.6, 127.4, 126.8, 126.8, 122.9, 122.0, 121.6, 121.5, 121.2, 120.5, 114.4, 114.1, 37.4, 37.0, 23.4, 22.8, 21.2, 21.0, 12.9, 12.8; HRMS m/z calculated for $[C_{27}H_{26}NO]^+$ ($[M+H]^+$): 380.2009, observed

4-(1,2-di-*p*-tolylvinyl)-1-ethyl-1H-benzo[*c*][1,2]thiazine 2,2-dioxide (**6n**)

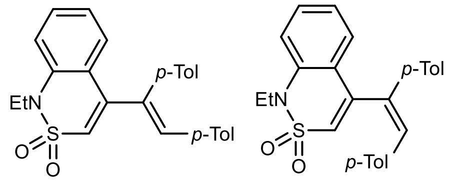

(*E*)-6n **(*Z*)-6n**

Prepared according to the ***General Procedure B*** using (*E*)-*N*-ethyl-2-(*p*-tolyl)-*N*-(2-(*p*-tolylethynyl)phenyl)ethene-1-sulfonamide **4n** (0.1 mmol, 1.0 equiv.), 25 mg, 60% yield (*E/Z* 1.3:1); **(*E*)-6n:** white solid; m.p.110 –111 °C; ^{1}H NMR (400 MHz, $CDCl_3$) δ 7.57 (dd, *J* = 8.0, 1.5 Hz, 1H), 7.38 (td, *J* = 7.8, 7.1, 1.5 Hz, 1H), 7.28 – 7.26 (m, 1H), 7.08 – 6.99 (m, 9H), 6.85 (s, 1H), 6.78 (s, 1H), 4.09 (q, *J* = 7.1 Hz, 2H), 2.31 (s,

3H), 2.29 (s, 3H), 1.39 (t, *J* = 7.1 Hz, 3H); ^{13}C NMR (100 MHz, $CDCl_3$) δ 149.5, 139.6, 138.1, 138.0, 137.2, 135.1, 133.6, 133.2, 130.9, 129.9, 129.8, 129.6, 129.5, 129.1, 122.98, 122.96, 122.6, 118.6, 41.8, 21.4, 21.4, 14.7; HRMS *m/z* calculated for $[C_{26}H_{25}NNaO_2S]^+$ ($[M+Na]^+$): 438.1498, observed : 438.1498; **(*Z*)-6n:** colorless oil; ^{1}H NMR (400 MHz, $CDCl_3$) δ 7.48 – 7.41 (m, 2H), 7.32 (d, *J* = 8.3 Hz, 1H), 7.28 – 7.26 (m, 2H), 7.19 (s, 1H), 7.14 – 7.10 (m, 4H), 7.03 – 6.98 (m, 3H), 6.66 (s, 1H), 4.27 – 4.12 (m, 2H), 2.32 (s, 3H), 2.27 (s, 3H), 1.45 (t, *J* = 7.1 Hz, 3H); ^{13}C NMR (100 MHz, $CDCl_3$) δ 145.8, 139.8, 138.2, 138.1, 137.3, 135.2, 132.7, 131.3, 131.0, 129.6, 129.4, 129.3, 129.1, 126.1, 123.2, 122.7, 122.4, 117.9, 40.9, 21.4, 21.3, 14.9; HRMS *m/z* calculated for $[C_{26}H_{25}NNaO_2S]^+$ ($[M+Na]^+$): 438.1498, observed : 438.1499.

4-(2-(*p*-tolyl)-1-(trimethylsilyl)vinyl)-1-oxaspiro[4.5]dec-3-en-2-one (6o)

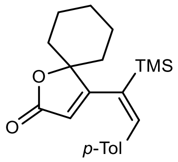

(*E*)-6o

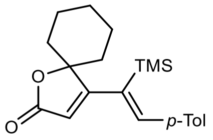

(*Z*)-6o

Prepared according to the ***General Procedure B*** using 1-((trimethylsilyl)ethynyl)cyclohexyl (*E*)-3-(*p*-tolyl)acrylate **4o** (0.05 mmol, 1.0 equiv.) and CH_2Cl_2 (5 mL, 0.01 M), 24 mg, 70% yield (*E/Z* 1:1); **(*E*)-6o:** white solid; ^{1}H NMR (400 MHz, $CDCl_3$) δ 7.19 (d, *J* = 8.1 Hz, 2H), 7.06 (d, *J* = 8.0 Hz, 2H), 6.90 (s, 1H), 5.85 (s, 1H), 2.32 (s, 3H), 1.65 – 1.47 (m, 6H), 1.37 – 1.23 (m, 4H), 0.23 (s, 9H); ^{13}C NMR (100 MHz, $CDCl_3$) δ 177.6, 172.1, 141.4, 138.6, 136.6, 135.6, 129.2, 129.1, 117.0, 91.7, 34.6, 24.5, 22.3, 21.5, 0.2; HRMS *m/z* calculated for $[C_{21}H_{28}NaO_2Si]^+$ ($[M+Na]^+$): 363.1751, observed : 363.1762; **(*Z*)-6o:** white solid; ^{1}H NMR (400 MHz, $CDCl_3$) δ 7.17 – 7.16 (m, 5H), 5.62 (s, 1H), 2.37 (s, 3H), 1.81 – 1.60 (m, 10H), 0.02 (s, 9H); ^{13}C NMR (100 MHz, $CDCl_3$) δ 179.6, 175.3, 146.1, 138.3, 137.4, 135.5, 129.0, 128.6, 114.9, 90.1, 34.0, 24.8, 22.3, 21.4, 1.5; HRMS *m/z* calculated for $[C_{21}H_{29}O_2Si]^+$ ($[M+H]^+$): 341.1931, observed : 341.1931

(*Z*)-1-phenyl-4-(2-(*p*-tolyl)-1-(trimethylsilyl)vinyl)-1-azaspiro[4.5]dec-3-en-2-one (6p)

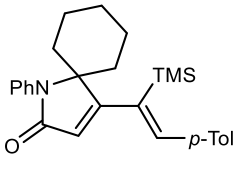

Prepared according to the ***General Procedure B*** using (*E*)-*N*-phenyl-3-(*p*-tolyl)-*N*-(1-((trimethylsilyl)ethynyl)cyclohexyl)acrylamide **4p** (0.1 mmol, 1.0 equiv.), 25 mg, 60% yield; white solid; m.p. 174 – 176 °C; ^{1}H NMR (400 MHz, $CDCl_3$) δ 7.47 – 7.37 (m, 3H), 7.26 – 7.23 (m, 3H), 7.21 – 7.16 (m, 4H), 5.82 (s, 1H), 2.38 (s, 3H), 2.02 – 1.96 (m, 2H), 1.89 – 1.82 (m, 2H), 1.72 – 1.63 (m, 2H), 1.45 – 1.27 (m, 4H), 0.06 (s, 9H); ^{13}C NMR (100 MHz, $CDCl_3$) δ 172.7, 171.0, 147.1, 140.1, 138.0, 137.8, 136.0, 131.5, 129.3, 128.9, 128.5,

128.5, 120.7, 70.3, 33.8, 25.0, 22.4, 21.4, 1.7; HRMS *m/z* calculated for $[C_{27}H_{34}NOSi]^+$ ($[M+H]^+$): 416.2404, observed : 416.2404

2-ethyl-8-methyl-4-(*p*-tolyl)-2,3-dihydro-1H-benzo[*e*]isoindol-1-one (6q′)

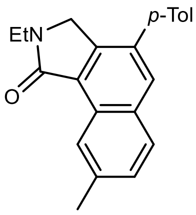

Prepared according to the ***General Procedure B*** using (*E*)-*N*-ethyl-3-(*p*-tolyl)-*N*-(3-(*p*-tolyl)prop-2-yn-1-yl)acrylamide **4q** (0.1 mmol, 1.0 equiv.), 17 mg, 54% yield; yellow oil; ^{1}H NMR (400 MHz, $CDCl_3$) δ 8.33 (s, 1H), 7.94 (d, *J* = 8.4 Hz, 1H), 7.49 (s, 1H), 7.37 – 7.35 (m, 3H), 7.28 (d, *J* = 8.0 Hz, 2H), 4.27 (s, 2H), 3.67 (q, *J* = 7.3 Hz, 2H), 2.49 (s, 3H), 2.43 (s, 3H), 1.24 (t, *J* = 7.3 Hz, 3H); ^{13}C NMR (100 MHz, $CDCl_3$) δ 168.3, 137.8, 137.6, 135.0, 134.2, 134.1, 133.7, 131.8, 129.8, 129.7, 129.64, 129.63, 128.4, 124.9, 123.0, 49.2, 37.3, 22.2, 21.5, 13.6; HRMS *m/z* calculated for $[C_{22}H_{22}NO]^+$ ($[M+H]^+$): 316.1696, observed : 316.1697

4,4-di-*tert*-butyl-8,9-di-*p*-tolyl-3,5-dioxa-4-silabicyclo[5.2.0]no*N*-7-en-2-one (5r)

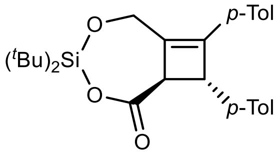

Prepared according to the ***General Procedure B*** using di-*tert*-butyl((3-(*p*-tolyl)prop-2-yn-1-yl)oxy)silyl (*E*)-3-(*p*-tolyl)acrylate **4r** (0.1 mmol, 1.0 equiv.), 28 mg, 62% yield; white solid; m.p. 68 – 70 °C; ^{1}H NMR (400 MHz, $CDCl_3$) δ 7.19 (d, *J* = 8.0 Hz, 2H), 7.14 – 7.05 (m, 4H), 7.02 (d, *J* = 8.1 Hz, 2H), 5.22 (dt, *J* = 15.8, 1.8 Hz, 1H), 4.95 (dt, *J* = 15.9, 2.3 Hz, 1H), 4.45 (d, *J* = 2.1 Hz, 1H), 3.61 (d, *J* = 1.7 Hz, 2H), 2.32 (s, 3H), 2.31 (s, 3H), 1.07 (s, 9H), 1.06 (s, 9H); ^{13}C NMR (100 MHz, $CDCl_3$) δ 69.0, 142.2, 138.2, 137.0, 136.5, 133.9, 130.1, 129.3, 129.3, 127.1, 127.0, 62.5, 56.1, 48.7, 27.3, 27.0, 21.3, 21.1, 21.0.. HRMS *m/z* calculated for $[C_{28}H_{37}O_3Si]^+$ ($[M+H]^+$): 449.2506, observed 449.2506.

Synthesis of substrates

Preparation and characterization of alkynes for intermolecular reaction

Di(*p*-tolyl)acetylene (1a)

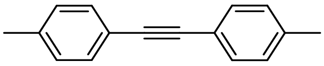

To a suspension of $Pd(PPh_3)_4$ (5 mol%), CuI (10 mol%), and 4-bromotoluene (1.0 equiv.) in degassed piperidine (1 mL, 0.2 M) was added dropwise *p*-tolylacetylene (0.2 mmol, 1.0 equiv.) under nitrogen atmosphere. The reaction mixture was stirred at 80°C for overnight. The reaction mixture was washed with a saturated solution of NH_4Cl and extracted with CH_2Cl_2. The combined organic layer was dried over Na_2SO_4, filtered and concentrated. The crude material was purified by flash chromatography. 77% yield; white solid; ^1H NMR (400 MHz, $CDCl_3$) δ 7.41 (d, *J* = 8.0 Hz, 4H), 7.14 (d, *J* = 7.9 Hz, 4H), 2.36 (s, 6H); The structure was further confirmed by spectral comparison with literature data.[4]

1,2-di-*o*-tolylethyne (1b)

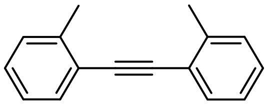

To a suspension of $Pd(PPh_3)_2Cl_2$ (5 mol%) and PPh_3 (10 mol%) in DMSO (1.2 mL, 0.17 M) was sequentially added dropwise DBU (2.0 equiv.), 2-bromotoluene (2.0 equiv.) and propiolic acid (0.2 mmol, 1.0 equiv.) under nitrogen atmosphere. The reaction mixture was stirred at 80 °C for 6 h. When TLC indicated the reaction was complete, the reaction mixture was washed with a saturated aqueous solution of NH_4Cl and extracted with EtOAc. The combined organic layer was dried over $MgSO_4$, filtered and concentrated *in vacuo*. The resulting crude product was purified by flash column chromatography on silica gel. 61% yield; white solid; ^1H NMR (400 MHz, $CDCl_3$) δ 7.51 (d, *J* = 7.3 Hz, 2H), 7.25 – 7.23 (m, 4H), 7.21 – 7.15 (m, 2H) 2.53 (s, 6H); The structure was further confirmed by spectral comparison with literature data.[5]

1,2-bis(4-methoxyphenyl)ethyne (1d)

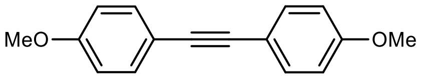

To a suspension of $Pd(PPh_3)_2Cl_2$ (5 mol%) and PPh_3 (10 mol%) in DMSO (1.2 mL, 0.17 M) was sequentially added dropwise DBU (2.0 equiv.), 4-iodoanisole (2.0 equiv.) and propiolic acid (0.2 mmol, 1.0 equiv.) under nitrogen atmosphere. The reaction mixture was stirred at 80 °C for overnight. When TLC indicated the reaction was complete, the reaction mixture was washed with a saturated aqueous solution of NH_4Cl and extracted with EtOAc. The combined organic layer was dried over $MgSO_4$, filtered and concentrated *in vacuo*. The resulting crude product was purified by flash column chromatography on silica gel. 73% yield; white solid; ^1H NMR (400

MHz, $CDCl_3$) δ 7.45 (d, *J* = 8.9 Hz, 4H), 6.87 (d, *J* = 8.8 Hz, 4H), 3.82 (s, 6H); The structure was further confirmed by spectral comparison with literature data.[5]

1,2-bis(2-methoxyphenyl)ethyne (1e)

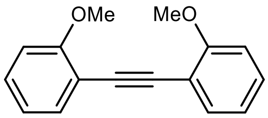

To a suspension of $Pd(OAc)_2$ (5 mol%), CuI (10 mol%), and triphenylphosphine (10 mol%) in degassed MeCN (2.5 mL, 0.4 M) was added Et_3N (3.0 equiv.), 1-iodo-2-methoxybenzene (1.0 mmol, 1.0 equiv.), and calcium carbide (3.0 equiv.) under nitrogen atmosphere. The reaction mixture was stirred at r.t. for overnight. The reaction mixture was filtered through a pad of Celite®, and concentrated. The crude material was purified by flash chromatography. 97% yield; white solid; ^{1}H NMR (400 MHz, $CDCl_3$) δ 7.52 (dd, *J* = 7.6, 1.7 Hz, 2H), 7.29 (ddd, *J* = 8.4, 7.4, 1.8 Hz, 2H), 6.93 (td, *J* = 7.5, 0.9 Hz, 2H), 6.91 (d, J= 8.4 Hz, 2H), 3.93 (s, 6H); The structure was further confirmed by spectral comparison with literature data.[6]

Dimethyl 4,4'-(ethyne-1,2-diyl)dibenzoate (1f)

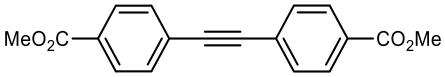

To solution of methyl 4-bromobenzoate (0.5 mmol, 1.0 equiv.), methyl 4-ethynylbenzonate (1.0 equiv.) and piperidine (5.0 equiv.) in degassed acetonitrile (1.4 mL, 0.35 M) was added $Ph(PPh_3)_4$ (2 mol%) under nitrogen atmosphere. The reaction mixture was stirred at 85 °C for 3 hours. The reaction mixture was filtered through a pad of Celite®, and concentrated. The crude material was purified by flash chromatography. 84% yield; yellow solid; ^{1}H NMR (400 MHz, $CDCl_3$) δ 8.04 (d, *J* = 8.6 Hz, 4H), 7.61 (d, *J* = 8.5 Hz, 4H), 3.94 (s, 6H); The structure was further confirmed by spectral comparison with literature data.[7]

Synthesis of 1-bromo-2-(phenylethynyl)benzene (1g)

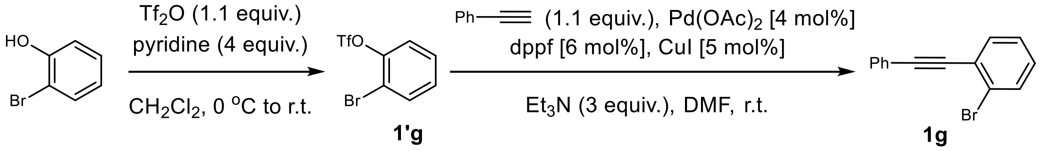

2-bromophenyl trifluoromethanesulfonate (1′g)

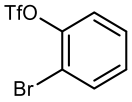

To a suspension of 2-bromophenol (1.0 mmol, 1.0 equiv.) in CH_2Cl_2 (3 mL, 0.3 M) was added pyridine (4.0 equiv.). Then the reaction mixture was cooled to 0 ºC and Trifluoromethanesulfonic anhydride (1.1 equiv.) was added dropwise. The reaction mixture was slowly warmed up to room temperature. and stirred at room temperature for 4 hours. The reaction mixture was washed with water and extracted with CH_2Cl_2. The combined organic layer was dried over Na_2SO_4, filtered and concentrated. The crude material was purified by flash chromatography. 98% yield; colorless liquid; ^{1}H NMR (400 MHz, $CDCl_3$) δ 7.69 (dd, *J* = 8.0, 1.6 Hz, 1H), 7.44 – 7.33 (m, 2H), 7.30 – 7.23 (m, 1H); The structure was further confirmed by spectral comparison with literature data.[8]

1-bromo-2-(phenylethynyl)benzene (1g)

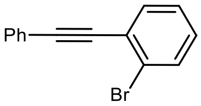

To a suspension of $Pd(OAc)_2$ (4 mol%), dppf (6 mol%), CuI (5 mol%), 2-bromophenyl trifluoromethanesulfonate **1′g** (1.0 mmol, 1.0 equiv.) and Et_3N (3.0 equiv.). in degassed DMF (10 mL, 0.1 M) were added dropwise phenylacetylene (1.1 equiv.). The reaction mixture was stirred at room temperature for 6 hours. The reaction mixture was washed with water and extracted with CH_2Cl_2. The combined organic layer was dried over Na_2SO_4, filtered and concentrated. The crude material was purified by flash chromatography. 70% yield; colorless liquid; ^{1}H NMR (400 MHz, $CDCl_3$) δ 7.65 – 7.48 (m, 4H), 7.39 – 7.33 (m, 3H), 7.29 (td, *J* = 7.6, 1.2 Hz, 1H), 7.18 (td, *J* = 7.8, 1.7 Hz, 1H); The structure was further confirmed by spectral comparison with literature data.[9]

2,2,2-trifluoro-*N*-(2-(phenylethynyl)phenyl)acetamide (1h)

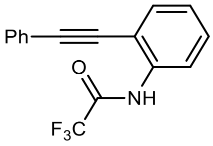

To solution of 2-(phenylethynyl)aniline **1x** (0.2 mmol, 1.0 equiv.) in CH_2Cl_2 (1.3 mL, 0.15 M) was added pyridine (2.0 equiv.) and trifluoroacetic anhydride (1.2 equiv.) at 0 °C. The reaction mixture was slowly warmed up to room temperature and stirred at room temperature for 3 hours. The reaction mixture was washed with water and extracted with CH_2Cl_2. The combined organic layer was dried over Na_2SO_4, filtered and concentrated. The crude material was purified by flash chromatography. 86% yield; white solid; ^{1}H NMR (400 MHz, $CDCl_3$) δ 8.89 (s, 1H), 8.38 (dd, *J* = 8.4, 1.0 Hz, 1H), 7.59 – 7.51 (m, 3H), 7.46 – 7.38 (m, 4H), 7.22 (td, *J* = 7.6, 1.2 Hz, 1H); The structure was further confirmed by spectral comparison with literature data.[10]

2-(*p*-tolylethynyl)pyridine (1i)

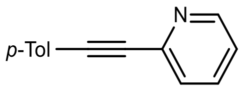

To a suspension of $Pd(PPh_3)_2Cl_2$ (3 mol%), CuI (6 mol%), and 2-bromopyridine (0.3 mmol, 1.0 equiv.) in degassed Et_3N (0.75 mL, 0.4 M) was added dropwise *p*-tolylacetylene (1.5 equiv.) under nitrogen atmosphere. The reaction mixture was stirred at 80 °C for overnight. The reaction mixture was washed with a saturated solution of NH_4Cl and extracted with CH_2Cl_2. The combined organic layer was dried over Na_2SO_4, filtered and concentrated. The crude material was purified by flash chromatography on silica gel. 90% yield; brown solid; ^{1}H NMR (400 MHz, $CDCl_3$) δ 8.61 (ddd, *J* = 4.9, 1.8, 0.9 Hz, 1H), 7.67 (td, 7.7, 1.8 Hz, 1H), 7.53 – 7.48 (m, 3H), 7.23 (ddd, *J* = 7.6, 4.9, 1.2 Hz, 1H), 7.17 (d, 7.8 Hz, 2H), 2.38 (s, 3H); The structure was further confirmed by spectral comparison with literature data.[11]

1,2-di(pyridin-2-yl)ethyne (1j)

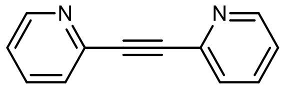

To a suspension of $Pd(PPh_3)_2Cl_2$ (2.5 mol%), CuI (5 mol%), 2-bromopyridine (3.0 mmol, 1.0 equiv.), distilled water (0.5 equiv.), and DBU (6.0 equiv.) in degassed toluene (3 mL, 1.0 M) was added dropwise trimethylsilylacetylene (0.6 equiv.) under nitrogen atmosphere. The reaction mixture was stirred at 80 °C for 1h. The reaction mixture was washed with a saturated solution of NH_4Cl and extracted with ethyl acetate. The combined organic layer was dried over Na_2SO_4, filtered and concentrated. The crude material was purified by flash chromatography on silica gel. 81% yield; brown solid; ^{1}H NMR (400 MHz, $CDCl_3$) δ 8.65 (d, *J* = 4.3 Hz, 2H), 7.71 (td, 7.7, 1.8 Hz, 2H), 7.63 (dt, 7.8, 0.9 Hz, 2H), 7.29 (ddd, *J* = 7.5, 4.9, 1.2 Hz, 2H); The structure was further confirmed by spectral comparison with literature data.[12]

Synthesis of 2-(pyridin-2-ylethynyl)pyrazine (1k)

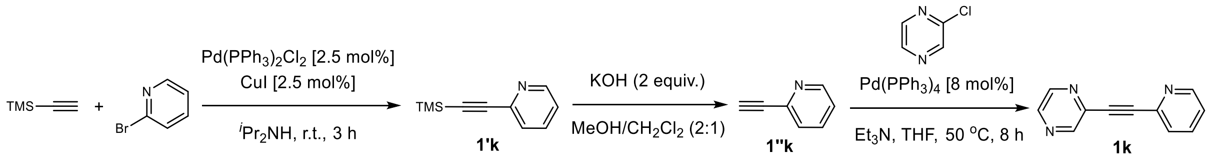

2-((trimethylsilyl)ethynyl)pyridine (1′k)

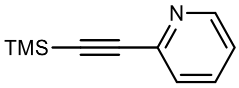

In a three neck round bottom flask, 2-bromopyridine (5.25 mmol, 1.0 equiv.), $Pd(PPh_3)_2Cl_2$ (2.5 mol%) and CuI (2.5 mol%) were added. The flask was flushed with nitrogen and dry $^{i}Pr_2NH$ (8.5 mL) was added into the flask while stirring at room temperature, followed by trimethylsilylacetylene (1.1 equiv.). After 3 h, the reaction mixture was quenched with water; extracted with CH_2Cl_2, dried with Na_2SO_4 and filtered. The solvent was removed under reduced pressure and the crude residue was purified by flash chromatography. 90% yield; yellow solid; ^1H NMR (400 MHz, $CDCl_3$) δ 8.56 (ddd, *J* = 4.9, 1.8, 1.0 Hz, 1H), 7.62 (td, *J* = 7.7, 1.8 Hz, 1H), 7.44 (dt, *J* = 7.9, 1.1 Hz, 1H), 7.24 – 7.18 (m, 1H), 0.26 (s, 9H); The structure was further confirmed by spectral comparison with literature data.[13]

2-ethynylpyridine (1″k)

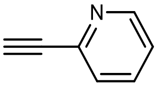

To a stirred solution of 2-((trimethylsilyl)ethynyl)pyridine **1′k** (3.99 mmol, 1.0 equiv.) in dry MeOH/CH_2Cl_2 (10 mL/5 mL), was added KOH (2.0 equiv.) under nitrogen atmosphere. After 2 h, the reaction mixture was quenched with water, extracted with CH_2Cl_2, dried with Na_2SO_4, and filtered. The solvent was removed under reduced pressure and the crude residue was purified by flash chromatography. 91% yield; yellow oil; ^1H NMR (400 MHz, $CDCl_3$) δ 8.55 (ddd, *J* = 4.9, 1.8, 1.0 Hz, 1H), 7.62 (tdd, *J* = 7.8, 1.8, 0.5 Hz, 1H), 7.44 (dt, *J* = 7.8, 1.1 Hz, 1H), 7.22 (ddd, *J* = 7.6, 4.9, 1.2 Hz, 1H), 3.13 (s, 1H); The structure was further confirmed by spectral comparison with literature data.[13]

2-(pyridin-2-ylethynyl)pyrazine (1k)

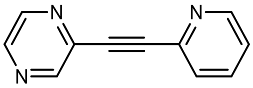

A solution of 2-chloro pyrazine (0.69 mmol, 1.0 equiv.) and Et_3N (0.8 mL) in THF (2 mL) was purged with nitrogen for 10 min at room temperature. 2-ethynylpyridine **1″k** (1.2 equiv.) was added to the solution at room temperature under nitrogen atmosphere, followed by addition of $Pd(PPh_3)_4$ (8 mol%). The mixture was stirred under nitrogen atmosphere at 50 °C for 8 h. The reaction mixture was cooled to room temperature and then concentrated under vacuum. The crude residue was diluted with Et_2O (5 mL) and filtered through a filter paper to get rid of the catalyst. The filtrate was washed with water and the ether layer was separated, dried over anhydrous Na_2SO_4, and concentrated. The crude product was purified by flash chromatography. 75% yield; yellow solid; ^1H NMR (400 MHz, $CDCl_3$) δ 8.84 (d, *J* = 1.6 Hz, 1H), 8.67 (ddd, *J* = 4.9, 1.9, 1.0 Hz, 1H), 8.60 (dd, *J* = 2.5, 1.6

Hz, 1H), 8.53 (d, J = 2.6 Hz, 1H), 7.73 (td, J = 7.7, 1.8 Hz, 1H), 7.64 (dt, J = 7.8, 1.1 Hz, 1H), 7.32 (ddd, J = 7.7, 4.9, 1.3 Hz, 1H); ^{13}C NMR (100 MHz, $CDCl_3$) δ 150.3, 148.2, 144.5, 143.4, 142.0, 139.6, 136.3, 127.9, 123.8, 91.6, 84.8; MS (APCI): *m/z* 182.1 $[M+H]^+$

1-(cyclopropylethynyl)-4-methylbenzene (1l)

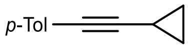

To a suspension of $Pd(PPh_3)_4$ (1 mol%), CuI (2 mol%), and 4-iodotoluene (0.5 mmol, 1.0 equiv.) in dry THF (1.7 mL, 0.3 M) was successively added dropwise dry $^{i}Pr_2NH$ (1.5 equiv.) and cyclopropylacetylene (1.1 equiv.) under nitrogen atmosphere. The reaction mixture was stirred at room temperature for overnight. When TLC indicated the reaction was complete, the reaction mixture was diluted in EtOAc and filtered through a pad of Celite®. The pad was rinsed with an additional EtOAc and the combined filtrate was concentrated *in vacuo*. The resulting crude product was purified by flash column chromatography on silica gel to afford the desired product. >99% yield; colorless oil; ^{1}H NMR (400 MHz, $CDCl_3$) δ 7.26 (d, J = 8.2 Hz, 2H), 7.06 (d, J = 7.9 Hz, 2H), 2.32 (s, 3H), 1.47 – 1.40 (m, 1H), 0.87 – 0.82 (m, 2H), 0.81 – 0.76 (m, 2H); The structure was further confirmed by spectral comparison with literature data.[14]

1,4-bis((4-chlorobenzyl)oxy)but-2-yne (1m)

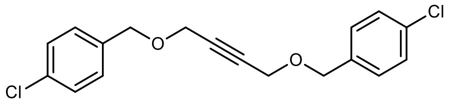

To a suspension of NaH (60% in mineral oil, 2.5 equiv.) in dry DMF was added slowly a solution of 2-butyne-1,4-diol (0.5 mmol, 1.0 equiv.) in DMF (1.6 mL, 0.3 M) at 0 °C under nitrogen atmosphere. The reaction mixture was stirred at 0°C for 1h. 4-chlorobenzyl chloride (2.1 equiv.) in dry DMF was added to the reaction mixture. Then, the reaction temperature was increased to room temperature and the mixture was stirred for 6 h. The reaction mixture was washed with water and extracted with Et_2O. The combined organic layer was dried over Na_2SO_4, filtered and concentrated. The crude material was purified by flash chromatography on silica gel. 69% yield; white solid; ^{1}H NMR (400 MHz, $CDCl_3$) δ 7.34 – 7.26 (m, 8H), 4.56 (s, 4H), 4.23 (s, 4H); ^{13}C NMR (100 MHz, $CDCl_3$) δ 135.9, 133.7, 129.3, 128.6, 82.5, 70.8, 57.5; MS (APCI): *m/z* 335.3 $[M+H]^+$

Benzyldimethyl(3-phenylprop-1-yn-1-yl)silane (1o)

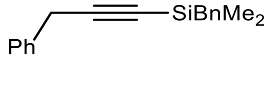

To a solution of prop-2-yn-1-ylbenzene (1 mmol, 1.0 equiv.) in THF (2 mL, 0.5 M) was added dropwise *n*-BuLi (2.5 M in hexane, 1.1 equiv.) at -78 °C. After 1 h, benzyl(chloro)dimethylsilane (1.1 equiv.) was added to the solution. The reaction mixture was slowly warmed up to room temperature and stirred at room temperature for overnight. The reaction mixture was quenched with water and extracted with Et_2O. The combined organic layer was dried over Na_2SO_4, filtered and concentrated. The crude material was purified by flash chromatography. 78%; colorless liquid; ^{1}H NMR (400 MHz, $CDCl_3$) δ 7.36 – 7.29 (m, 4H), 7.28 – 7.17 (m, 3H), 7.13 – 7.03 (m, 3H), 3.65 (s, 2H), 2.21 (s, 2H), 0.15 (s, 6H); ^{13}C NMR (100 MHz, $CDCl_3$) δ 139.2, 136.3, 128.6, 128.5, 128.3, 128.1, 126.7, 124.4, 105.8, 85.5, 26.6, 26.4, -1.8; MS (APCI): *m/z* 265.6 $[M+H]^+$

1-((trimethylsilyl)ethynyl)cyclohexan-1-ol (1p)

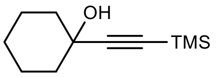

A flame-dried round-bottom flask was charged with *n*-BuLi (2.5 M in hexane, 1.3 equiv.) under an argon atmosphere. Anhydrous THF (6 mL, 0.5 M) was added, and the flask was cooled to -78 °C. Trimethylsilylacetylene (1.3 equiv.) was then added dropwise. After 30 minutes at -78 °C, cyclohexanone (3.05 mmol, 1.0 equiv.) was added dropwise. The reaction mixture was warmed to r.t. and stirred overnight. After completion, saturated NH_4Cl solution was added. The mixture was extracted with Et_2O, and the organic phases were combined and washed with brine, dried over anhydrous Na_2SO_4, filtered and concentrated to yield crude product. The crude product was purified by flash chromatography. 94% yield; white solid; ^{1}H NMR (400 MHz, $CDCl_3$) δ 1.95 – 1.83 (m, 3H), 1.73 – 1.64 (m, 2H), 1.59 – 1.50 (m, 5H), 0.17 (d, J = 0.6 Hz, 9H); The structure was further confirmed by spectral comparison with literature data.[15]

1-ethynylcyclohexan-1-ol (1q)

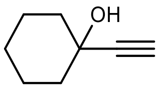

A suspension of cesium carbonate (0.5 equiv.), calcium carbide (2.5 equiv.) in DMSO/H_2O (33.6 mL, 50:1, 0.3 M) was bubbled with nitrogen for 20 min, and cyclohexanone (10 mmol, 1.0 equiv.) was added. The reaction mixture was stirred at 60 °C for overnight. The reaction mixture was washed with water and extracted with Et_2O. The combined organic layer was dried over Na_2SO_4, filtered and concentrated. The crude material was purified by flash chromatography. 77% yield; colorless liquid; ^{1}H NMR (400 MHz, $CDCl_3$) δ 2.47 (s, 1H), 1.96 – 1.89 (m, 2H), 1.80 – 1.65 (m, 2H), 1.64 – 1.44 (m, 5H), 1.33 – 1.17 (m, 1H); The structure was further confirmed by spectral comparison with literature data.[16]

N-(1-ethynylcyclohexyl)acetamide (1r)

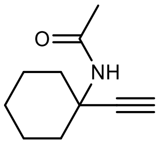

To a solution of 1-ethynylcyclohexa-1-ol **1q** (0.5 mmol, 1.0 equiv.) in CH_2Cl_2/MeCN (0.8 mL, 1:1, 0.5 M) was added dropwise concentrated Sulfuric acid (55 µL, 9 M) at 0 °C. The reaction mixture was slowly warmed up to room temperature and stirred at room temperature for 4 hours. The reaction mixture was washed with saturated aqueous sodium bicarbonate and extracted with CH_2Cl_2. The combined organic layer was dried over Na_2SO_4, filtered and concentrated. The crude material was purified by flash chromatography. 44% yield; white solid; ^{1}H NMR (400 MHz, $CDCl_3$) δ 5.56 (br s, 1H), 2.39 (s, 1H), 2.12 (d, J = 12.1 Hz, 2H), 1.96 (s, 3H), 1.86 – 1.50 (m, 7H), 1.35 – 1.16 (m, 1H); ^{13}C NMR (100 MHz, $CDCl_3$) δ 169.0, 85.5, 71.3, 51.7, 36.8, 25.2, 24.2, 22.4; MS (APCI): *m/z* 166.2 $[M+H]^+$

Synthesis of 2-hydroxy-2-methylbut-3-ynoic acid (1s)

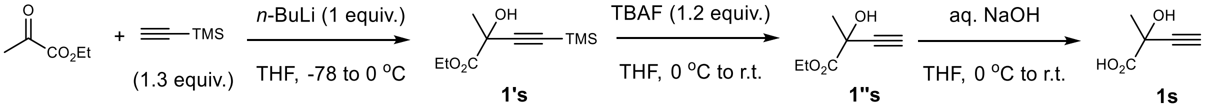

Ethyl 2-hydroxy-2-methyl-4-(trimethylsilyl)but-3-ynoate (1′s)

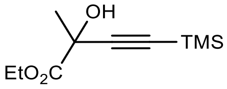

To a solution of (trimethylsilyl)acetylene (1.3 equiv.) in dry THF (20 mL) was added dropwise *n*-BuLi (2.5 M in hexane, 1.0 equiv.) at -78 °C under nitrogen atmosphere. After the reaction mixture was stirred for 30 min., added dropwise to another solution of ethyl pyruvate (5 mmol, 1.0 equiv.) in dry THF (20 mL) at the same temperature under nitrogen atmosphere. Then, the reaction temperature was slowly increased to 0 °C and the mixture was stirred for 1 h. When TLC indicated the reaction was complete, a saturated aqueous solution of NH_4Cl was added to quench the reaction at 0 °C. The solution was washed with water and extracted with EtOAc. The extracts were dried over Na_2SO_4, filtered and concentrated *in vacuo*. The resulting crude product was purified by flash column chromatography on silica gel to afford the desired product. 75% yield; yellow oil; ^{1}H NMR (400 MHz, $CDCl_3$) δ 4.38 – 4.24 (m, 2H), 3.47 (s, 1H), 1.67 (s, 3H), 1.33 (t, J = 7.1 Hz, 3H), 0.17 (s, 9H); ^{13}C NMR (100 MHz, $CDCl_3$) δ 172.7, 104.5, 88.9, 68.3, 63.0, 27.2, 14.1, -0.2; MS (APCI): *m/z* 215.1 $[M+H]^+$

Ethyl 2-hydroxy-2-methylbut-3-ynoate (1″s)

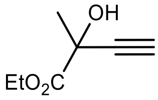

To a solution of ethyl 2-hydroxy-2-methyl-4-(trimethylsilyl)but-3-ynoate **1′s** (3.48 mmol, 1.0 equiv.) in dry THF (34 mL, 0.1 M) was added dropwise TBAF (1.2 equiv.) at 0 °C and stirred the mixture for 1 h at room temperature. When TLC indicated the reaction was complete, a saturated aqueous solution of NH_4Cl was added to quench the reaction. The solution was washed with water and extracted with Et_2O. The extracts were dried over Na_2SO_4, filtered and concentrated *in vacuo*. The resulting crude product was purified by flash column chromatography on silica gel to afford the desired product. 44% yield; colorless oil; ^{1}H NMR (400 MHz, $CDCl_3$) δ 4.32 (q, *J* = 7.1 Hz, 2H), 3.52 (s, 1H), 2.51 (s, 1H), 1.70 (s, 3H), 1.35 (t, *J* = 7.1 Hz, 3H); ^{13}C NMR (100 MHz, $CDCl_3$) δ 172.6, 83.3, 72.3, 67.8, 63.3, 27.3, 14.1; MS (APCI): *m/z* 143.3 $[M+H]^+$

2-hydroxy-2-methylbut-3-ynoic acid (1s)

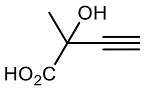

To a solution of ethyl 2-hydroxy-2-methylbut-3-ynoate **1″s** (0.2 mmol, 1.0 equiv.) in THF (1 mL, 0.2 M) was added 1.5 N NaOH (10 equiv.) at 0 °C and the reaction mixture was allowed to warm up to ambient temperature. The mixture was continuously stirred for 2 h. When TLC indicated the reaction was complete, the solution was slowly acidified to pH 3 with 1 N HCl at 0 °C. The solvent was evaporated with a stream of nitrogen. The mixture was diluted in EtOAc, filtered, and concentrated *in vacuo*. The resulting product was used without further purification. 88% yield; white solid; ^{1}H NMR (400 MHz, DMSO-d_6) δ 3.39 (s, 1H), 1.49 (s, 3H); The structure was further confirmed by spectral comparison with literature data.[17]

Synthesis of (3*r*,5*r*,7*r*)-1-ethynyladamantane (1t)

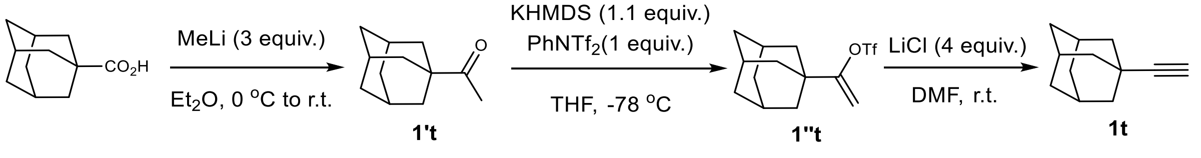

1-((3*r*,5*r*,7*r*)-adamantan-1-yl)ethan-1-one (1′t):

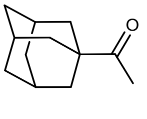

A solution of methyllithium (3.1 M in diethoxymethane, 3.0 equiv.) in anhydrous Et_2O was added at 0 °C to a stirred solution of (3*r*,5*r*,7*r*)-adamantane-1-carboxylic acid (1 mmol, 1.0 equiv.) in anhydrous Et_2O (5 mL), the

mixture was stirred for an additional 30 min at this temp. and for 4 h at 20 °C. The reaction mixture was poured slowly with vigorous stirring into a solution of conc. HCl (2 mL) in ice water (5 mL). The aq. layer was extracted with diethyl ether washed with $NaHCO_3$ solution, dried over anhydrous Na_2SO_4, filtered and concentrated under reduced pressure. The crude product was purified by flash chromatography. 97% yield; white solid; ^{1}H NMR (400 MHz, $CDCl_3$) δ 2.07 (d, J = 0.5 Hz, 3H), 2.06 – 1.99 (m, 3H), 1.79 – 1.70 (m, 8H), 1.69 – 1.61 (m, 4H). The structure was further confirmed by spectral comparison with literature data.[18]

1-((3*r*,5*r*,7*r*)-adamantan-1-yl)vinyl trifluoromethanesulfonate (1″t)

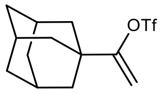

To a stirred solution of 1-((3*r*,5*r*,7*r*)-adamantan-1-yl)ethan-1-one **1′t** (0.8 mmol, 1.0 equiv.) and $PhNTf_2$ (1.0 equiv.) in anhydrous THF (5 mL) was added KHMDS (1.0 M in THF, 1.1 equiv.) at −78 °C. The resultant mixture was stirred at that temperature for 30 min before it was quenched with saturated aq. NH_4Cl and extracted with EtOAc. The combined organic phases were washed with brine, dried over anhydrous Na_2SO_4, filtered, and concentrated under vacuum. The crude residue was purified by flash chromatography. 92% yield; colorless oil; ^{1}H NMR (400 MHz, $CDCl_3$) δ 5.04 (d, J = 4.1 Hz, 1H), 4.87 (d, J = 4.2 Hz, 1H), 2.05 (q, J = 3.5 Hz, 3H), 1.76 (d, J = 3.1 Hz, 9H), 1.67 (dq, J = 12.4, 2.2 Hz, 3H). The structure was further confirmed by spectral comparison with literature data.[19]

(3*r*,5*r*,7*r*)-1-ethynyladamantane (1t)

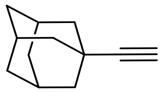

To a stirred solution of 1-((3*r*,5*r*,7*r*)-adamantan-1-yl)vinyl trifluoromethanesulfonate **1″t** (0.6 mmol, 1.0 equiv.) in DMF (2 mL) was added anhydrous LiCl (4.0 equiv.) at room temparature. The resultant mixture was stirred at that temperature for 30 min. The solvent was evaporated under vacuum and the crude was directly subjected to flash column chromatography. 94% yield; colorless solid; ^{1}H NMR (400 MHz, $CDCl_3$) δ 2.10 (s, 1H), 1.99 – 1.92 (m, 3H), 1.88 (d, J = 2.9 Hz, 6H), 1.69 (t, J = 3.0 Hz, 6H). The structure was further confirmed by spectral comparison with literature data.[20]

1-phenylbut-3-yn-1-ol (1u)

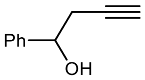

To a suspension of benzaldehyde (5 mmol, 1.0 equiv.) and propargyl bromide (3.0 equiv.) in THF (17 mL, 0.3 M) was added iron chloride (3.0 equiv.) at 0 °C. After stirring the mixture for 10 min, zinc dust (3.0 equiv.) was added in a few portions over a period. The reaction mixture was slowly warmed up to room temperature and stirred at room temperature for overnight. The reaction mixture was washed with 1 N aqueous HCl solution and extracted with CH_2Cl_2. The combined organic layer was dried over Na_2SO_4, filtered and concentrated. The crude material was purified by flash chromatography. 67% yield; colorless liquid; ^{1}H NMR (400 MHz, $CDCl_3$) δ 7.42 – 7.30 (m, 4H), 7.33 – 7.27 (m, 1H), 4.89 (t, *J* = 6.4 Hz, 1H), 2.65 (dd, *J* = 6.1, 2.6 Hz, 2H), 2.35 (s, 1H), 2.08 (t, *J* = 2.5 Hz, 1H); The structure was further confirmed by spectral comparison with literature data.[16]

1,4-di-*p*-tolylbuta-1,3-diyne (1v)

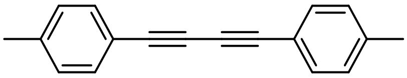

To a suspension of Cu(I)Cl (10 mol%) and piperidine (1.2 equiv.) in CH_2Cl_2 (2 mL, 0.1 M) with freshly activated molecular sieve (3Å) was added *p*-tolylacetylene (0.2 mmol, 1.0 equiv.). The reaction mixture was stirred at room temperature under air for 1 hour. The reaction mixture was filtered to remove the solids, washed with water and extracted with CH_2Cl_2. The combined organic layer was dried over Na_2SO_4, filtered and concentrated. The crude material was purified by flash chromatography. 99% yield; white solid; ^{1}H NMR (400 MHz, $CDCl_3$) δ 7.42 (d, *J* = 8.1 Hz, 4H), 7.14 (d, *J* = 8.2 Hz, 4H), 2.36 (s, 6H); The structure was further confirmed by spectral comparison with literature data.[21]

***tert*-butyldimethyl(2-(*p*-tolylethynyl)phenoxy)silane(1w)**

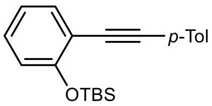

To a solution of the 2-(p-tolylethynyl)phenol (1.0 equiv.), imidazole (1.5 equiv.), and anhydrous CH_2Cl_2 was added TBSCl (1.1 equiv.). The reaction mixture was stirred until the alcohol all consumed. The mixture was quenched with distilled water and extracted with CH_2Cl_2. The organic layer was washed with distilled water, brine and dried over anhydrous $MgSO_4$, and concentrated to provide the desired product. 70% yield, Light orange liquid; ^{1}H NMR (400 MHz, $CDCl_3$) δ 7.47 – 7.45 (dd, *J* = 7.4, 1.8 Hz, 1H), 7.42 – 7.40 (d, *J* = 8.1 Hz, 2H), 7.21 – 7.17 (ddd, *J* = 8.2, 7.4, 1.8 Hz, 1H), 7.16 – 7.13 (m, 2H), 6.95 – 6.91 (m, 1H), 6.85 – 6.83 (dd, *J* = 8.2, 1.1 Hz, 1H), 2.36 (s, 3H), 1.06 – 1.04 (s, 9H), 0.26 - 0.25 (s, 6H); ^{13}C NMR (100 MHz, $CDCl_3$) δ 156.3, 138.0, 133.5, 131.3, 129.3, 129.1, 121.2, 120.7, 119.8, 116.0, 93.0, 86.3, 25.8, 21.5, 18.3, -4.3; MS (APCI): *m/z* 323.2 $[M+H]^+$

17α-ethynylestradiol 3-acetate (1y)

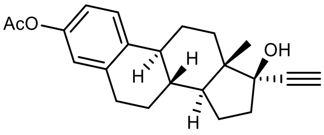

To a solution of Ethinyl estradiol (0.16 mmol, 1.0 equiv.) in CH_2Cl_2 (1.6 mL, 0.1 M) was added acetic anhydride (1.6 equiv.) and Et_3N (2.0 equiv.) at 0 °C. The reaction mixture was warm to room temperature and stirred for 2 hours. The mixture was concentrated *in vacuo* and purified by flash chromatography. 94% yield; ^{1}H NMR (400 MHz, $CDCl_3$) δ 7.30 (d, J = 8.9 Hz, 1H), 6.86 (dd, J = 8.4, 2.5 Hz, 1H), 6.80 (d, J = 2.5 Hz, 1H), 2.90 – 2.85 (m, 2H), 2.62 (s, 1H), 2.43 – 2.31 (m, 2H), 2.29 (s, 3H), 2.29 – 2.21 (m, 1H), 2.10 – 2.00 (m, 1H), 1.98 – 1.85 (m, 3H), 1.85 – 1.67 (m, 3H), 1.53 – 1.35 (m, 4H), 0.90 (s, 3H); ^{13}C NMR (100 MHz, $CDCl_3$) δ 169.8, 148.4, 138.2, 137.9, 126.4, 121.5, 118.6, 87.4, 79.8, 74.1, 49.5, 47.0, 43.7, 39.0, 38.9, 32.7, 29.5, 27.0, 26.2, 22.8, 21.1, 12.6; MS (APCI): *m/z* 339.2 $[M+H]^+$

Preparation and characterization of alkenes for intermolecular reaction

Synthesis of *N*, *N'*-difumaramide

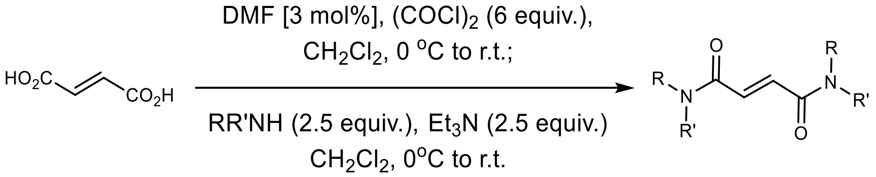

To a suspension of fumaric acid (1.0 equiv., 5.0 mmol), DMF (3 mol%) in dry CH_2Cl_2 (20 mL, 0.25 M) was added dropwise oxalyl chloride (6.0 equiv.) at 0 °C. The reaction mixture was stirred at room temperature for 8 hours. Then, the solvent was removed under reduced pressure. The residue was dissolved in dry CH_2Cl_2 and slowly added dropwise to a solution of amine (2.5 equiv.) and Et_3N (2.5 equiv.) in dry CH_2Cl_2 (25 mL, 0.2 M) at 0°C. The mixture was stirred at room temperature for 2 hours. The reaction mixture was diluted with water and extracted with CH_2Cl_2. The combined organic layer was dried over Na_2SO_4, filtered and concentrated. The crude material was purified by flash chromatography on silica gel.

(*E*)-1,4-di(pyrrolidin-1-yl)but-2-ene-1,4-dione (2c)

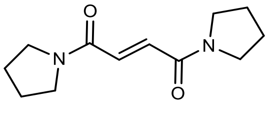

Prepared according to the above procedure using pyrrolidine (2.5 equiv.). 43% yield; yellow solid; ^{1}H NMR (400 MHz, $CDCl_3$) δ 7.23 (s, 2H), 3.60 (t, *J* = 6.8 Hz, 4H), 3.53 (t, *J* = 6.9 Hz, 4H), 1.97 (p, *J* = 6.7 Hz, 4H), 1.87 (p, *J* = 6.7 Hz, 4H); ^{13}C NMR (100 MHz, $CDCl_3$) δ 163.3, 131.6, 46.7, 46.1, 26.0, 24.2.; MS (APCI): *m/z* 223.5 $[M+H]^+$

N^1,N^4-dimethyl-*N^1,N^4*-diphenylfumaramide (2d)

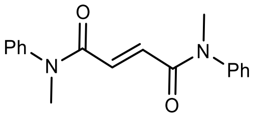

Prepared according to the above procedure using *N*-methyl aniline (2.5 equiv.). 90% yield; white solid; ^{1}H NMR (400 MHz, $CDCl_3$) δ 7.39 (t, *J* = 7.7 Hz, 4H), 7.32 (t, *J* = 7.3 Hz, 2H), 7.12 (d, *J* = 7.7 Hz, 4H), 6.84 (s, 2H), 3.29 (s, 6H).; ^{13}C NMR (100 MHz, $CDCl_3$) δ 164.6, 142.8, 131.5, 129.8, 127.9, 127.0, 37.6.; MS (APCI): *m/z* 295.7 $[M+H]^+$

Synthesis of methyl (*Z*)-4-(ethyl(*p*-tolyl)amino)-4-oxobut-2-enoate (2g)

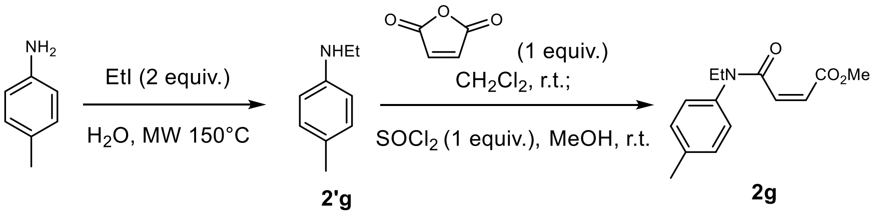

2'g 2g

N-ethyl-4-methylaniline (2′g)

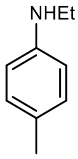

A mixture of *p*-toluidine (0.5 mmol, 1.0 equiv.) and ethyl iodide (2.0 equiv.) in water (0.17mL, 3 M) was microwave irradiated at 150°C for 20 minutes. The reaction mixture was washed with saturated aqueous $NaHCO_3$ and extracted with CH_2Cl_2. The combined organic layer was dried over Na_2SO_4, filtered and concentrated. The crude material was purified by flash chromatography. 44% yield; yellow liquid; ^{1}H NMR (400 MHz, $CDCl_3$) δ 6.99 (d, *J* = 8.4 Hz, 2H), 6.54 (d, *J* = 8.4 Hz, 2H), 3.41 (s, 1H), 3.14 (q, *J* = 7.1 Hz, 2H), 2.24 (s, 3H), 1.24 (t, *J* = 7.1 Hz, 3H); The structure was further confirmed by spectral comparison with literature data.[22]

Methyl (*Z*)-4-(ethyl(*p*-tolyl)amino)-4-oxobut-2-enoate (2g)

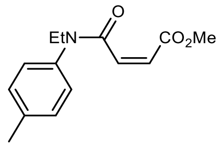

To the solution of *N*-ethyl-4-methylaniline **2′g** (0.18 mmol, 1.0 equiv.) in CH_2Cl_2 (0.9 mL, 0.2 M) was added maleic anhydride (1.0 equiv.). The reaction mixture was stirred at room temperature for 1 hour. The reaction mixture was stirred at room temperature for 1 h. The solvent was evaporated under reduced pressure, the reaction mixture was dissolved in the methanol (0.9 mL, 0.2 M) and added thionyl chloride (1.0 equiv.) at 0 °C. The reaction mixture was stirred at room temperature for 1 hour. The reaction mixture was washed with water and extracted with ethyl acetate. The combined organic layer was dried over Na_2SO_4, filtered and concentrated. The crude material was purified by flash chromatography. 67% yield; colorless liquid; ^1H NMR (400 MHz, $CDCl_3$) δ 7.16 (d, *J* = 8.0 Hz, 2H), 7.07 (d, *J* = 8.3 Hz, 2H), 6.24 (d, *J* = 11.9 Hz, 1H), 5.74 (d, *J* = 11.9 Hz, 1H), 3.82 (q, *J* = 7.2 Hz, 2H), 3.78 (s, 3H), 2.36 (s, 3H), 1.17 (t, *J* = 7.2 Hz, 3H); ^{13}C NMR (100 MHz, $CDCl_3$) δ 165.8, 165.5, 138.5, 138.2, 137.1, 130.0, 127.9, 123.8, 51.8, 43.5, 21.1, 12.8; MS (APCI): *m/z* 248.5 $[M+H]^+$

Synthesis of maleimide derivative (2i, 2k – 2n, 2z)

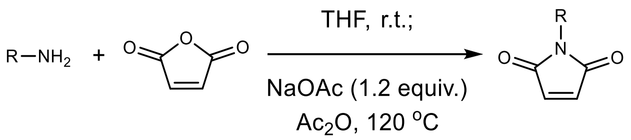

To a solution of maleic anhydride (1.1 equiv.) in THF (0.25 M) was added dropwise amine (1.0 equiv.). After the reaction mixture was stirred at room temperature for 0.5 h, the precipitate was filtered and washed with diethyl ether several time. The collected precipitate was dried under reduced pressure and used without further purification. To a solution of maleic acid (1.0 equiv.) in acetic anhydride (0.5 M) was added sodium acetate (1.2 equiv.) and the solution was heated at 120 °C for overnight. The mixture was concentrated under reduced pressure and purified by flash chromatography on silica gel.

1-cyclohexyl-1*H*-pyrrole-2,5-dione (2i)

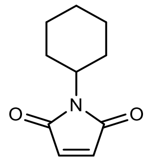

Prepared according to the above procedure using cyclohexylamine (1.0 equiv.). 63% yield; yellow solid; ^1H NMR (400 MHz, $CDCl_3$) δ 6.62 (s, 2H), 3.91 (tt, *J* = 3.9, 12.4 Hz, 1H), 2.05 (qd, *J* = 3.2, 12.4 Hz, 2H), 1.83 (d, *J* =

13.4 Hz, 2H), 1.66 (dd, J = 2.7, 12.2 Hz, 3H) δ 1.37 – 1.19 (m, 3H); The structure was further confirmed by spectral comparison with literature data.[23]

4-(2,5-dioxo-2,5-dihydro-1*H*-pyrrol-1-yl)benzonitrile (2k)

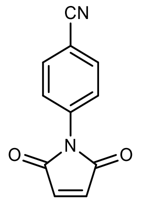

Prepared according to the above procedure using 4-aminobenzonitrile (3 mmol, 1.0 equiv.), 1,4-dioxane (10 mL) as solvent, and additional $(NH_4)_2S_2O_8$ (2.0 equiv.) and DMSO (2.0 equiv.) at 100 °C. 72% yield; pale brown solid; ^{1}H NMR (400 MHz, $CDCl_3$) δ 7.77 (d, J = 8.9 Hz, 2H), 7.60 (d, J = 8.9 Hz, 2H), 6.91 (s, 2H); The structure was further confirmed by spectral comparison with literature data.[23]

Methyl 4-(2,5-dioxo-2,5-dihydro-1H-pyrrol-1-yl)benzoate (2l)

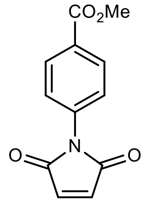

Prepared according to the above procedure using dry toluene (2.5 mL, 0.2 M) as solvent and methyl 4-aminobenzoate (0.5 mmol, 1.0 equiv.). 57% yield; pale yellow solid; ^{1}H NMR (400 MHz, $CDCl_3$): δ 8.14 (d, J = 8.8 Hz, 2H), 7.50 (d, J = 8.8 Hz, 2H), 6.89 (s, 2H), 3.94 (s, 3H); The structure was further confirmed by spectral comparison with literature data.[23]

1-phenyl-1*H*-pyrrole-2,5-dione (2m)

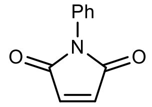

Prepared according to the above procedure using aniline (3.5 mmol, 1.0 equiv.). 82% yield; yellow solid; ^{1}H NMR (400 MHz, $CDCl_3$) δ 7.47 (t, J = 7.6 Hz, 2H), 7.40 – 7.32 (m, 3H), 6.85 (s, 2H); The structure was further confirmed by spectral comparison with literature data.[23]

1-(pyridin-3-yl)-1*H*-pyrrole-2,5-dione (2n)

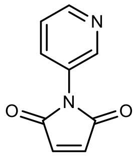

Prepared according to the above procedure using 3-aminopyridine (0.5 mmol, 1.0 equiv.) in Ac_2O (0.2 M) at 80 °C for 1 hour, 50% yield; yellow solid; ^{1}H NMR (400 MHz, $CDCl_3$) δ 8.66 (d, J = 2.3 Hz, 1H), 8.58 (dd, J = 4.8, 1.2 Hz, 1H), 7.71 (ddd, J = 8.2, 2.5, 1.6 Hz, 1H), 7.39 (dd, J = 8.2, 4.8 Hz, 1H), 6.88 (s, 2H); ^{13}C NMR (100 MHz, $CDCl_3$) δ 168.8, 148.6, 146.7, 134.5, 133.0, 128.3, 123.6; MS (APCI): *m/z* 175.4 $[M+H]^+$

1-(4-methoxyphenyl)-1*H*-pyrrole-2,5-dione (2z)

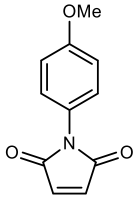

Prepared according to the above procedure using 4-methoxyaniline (1.0 equiv.). 59% yield; white solid; ^{1}H NMR (400 MHz, $CDCl_3$): δ 7.23 (d, J = 9.1 Hz, 2H), 6.98 (d, J = 9.1 Hz, 1H), 6.83 (s, 2H), δ 3.83 (s, 3H); The structure was further confirmed by spectral comparison with literature data.[23]

Synthesis of 1-(benzyloxy)-1*H*-pyrrole-2,5-dione (2j)

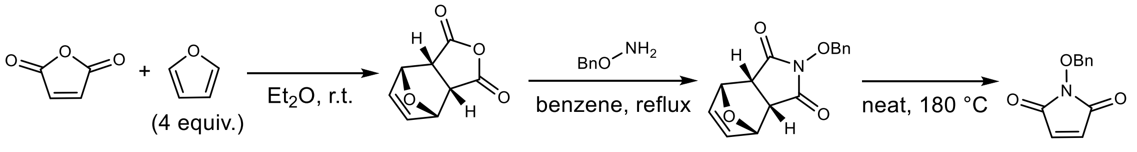

2'j 2''j 2j

(3a*R*,4*R*,7*S*,7a*S*)-3a,4,7,7a-tetrahydro-4,7-epoxyisobenzofuran-1,3-dione (2′j)

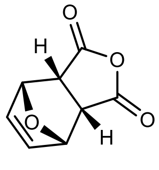

To a solution of maleic anhydride (1.0 equiv.) in anhydrous diethyl ether (0.5 M) was added furan (4.0 equiv.) while stirring at room temperature. The reaction was stirred for 24 hours at room temperature. The product was precipitated out and was collected by vacuum filtration. The colorless precipitation was used without further

purification. 69% yield; ^{1}H NMR (400 MHz, $CDCl_3$): δ 6.58 (s, 2H), δ 5.46 (s, 2H), δ 3.18 (s, 2H); The structure was further confirmed by spectral comparison with literature data.[23]

(3a*R*,4*R*,7*S*,7a*S*)-2-(benzyloxy)-3a,4,7,7a-tetrahydro-1*H*-4,7-epoxyisoindole-1,3(2*H*)-dione (2″j)

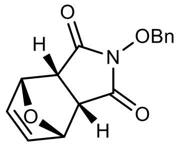

To a solution of (3a*R*,4*R*,7*S*,7a*S*)-3a,4,7,7a-tetrahydro-4,7-epoxyisobenzofuran-1,3-dione **2′j** (1.0 equiv.) in anhydrous benzene (0.25 M) was added solution of *N*-benzyloxyamine (1.0 equiv.) in benzene while stirring at room temperature. The reaction was stirred and refluxed for 3 hours. After completion (monitored by TLC analysis), the solution was cooled and concentrated under reduced pressure. The crude material was purified by flash chromatography. ^{1}H NMR (400 MHz, $CDCl_3$) δ 7.52 – 7.46 (m, 2H), 7.39 – 7.35 (m, 3H), 6.49 (s, 2H), δ 5.25 (s, 2H) δ 5.07 (s, 2H) δ 2.72 (s, 2H); The structure was further confirmed by spectral comparison with literature data.[23]

1-(benzyloxy)-1*H*-pyrrole-2,5-dione (2j)

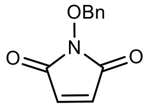

(3a*R*,4*R*,7*S*,7a*S*)-2-(benzyloxy)-3a,4,7,7a-tetrahydro-1H-4,7-epoxyisoindole-1,3(2*H*)-dione **2″j** (1.0 equiv.) was heated in an oil bath at 170-180 °C under reduced pressure. The solid slowly melted with vigorous furan evolution. After completion of furan evolution (monitored by TLC analysis), the yellowish liquid was cooled to RT. The crude material was purified by flash chromatography. 89% yield; white solid; ^{1}H NMR (400 MHz, $CDCl_3$): δ 7.51 – 7.46 (m, 2H), 7.40 – 7.33 (m, 3H), δ 6.58 (s, 2H), δ 5.10 (s, 2H); The structure was further confirmed by spectral comparison with literature data.[23]

Synthesis of 3-bromo-1-methyl-1H-pyrrole-2,5-dione (2o) and 3-methoxy-1-methyl-1H-pyrrole-2,5-dione (2p)

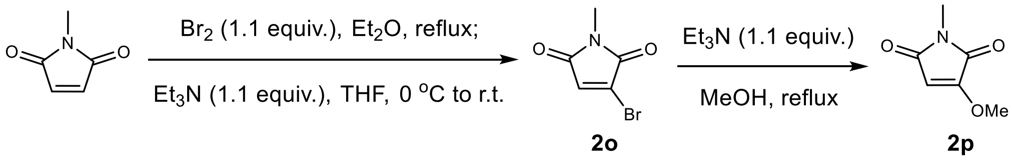

3-bromo-1-methyl-1*H*-pyrrole-2,5-dione (2o)

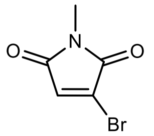

To a solution of *N*-methylmaleimide **2a** (2 mmol, 1.0 equiv.) in Et_2O (2.8 mL) was added Br_2 (1.1 equiv.). The solution was refluxed for 2 h, and evaporated solvent and Br_2 *in vacuo*. The crude was dissolved in THF (6.7 mL), cooled to 0 °C and Et_3N (1.1 equiv.) was added. The reaction mixture was stirred for overnight at room temperature. The reaction mixture was diluted in EtOAc, washed with water and extracted with EtOAc. The combined organic layer was dried over $MgSO_4$, filtered and concentrated under reduced pressure. The resulting crude product was purified by flash column chromatography on silica gel to afford the desired product. 79% yield; pale brown solid; ^1H NMR (400 MHz, $CDCl_3$) δ 6.88 (s, 1H), 3.08 (s, 3H); The structure was further confirmed by spectral comparison with literature data.[24]

3-methoxy-1-methyl-1*H*-pyrrole-2,5-dione (2p)

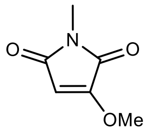

To a solution of 3-bromo-1-methyl-1H-pyrrole-2,5-dione **2o** (0.1 mmol, 1.0 equiv.) in MeOH (0.4 mL, 0.26 M) was added dropwise Et_3N (1.1 equiv.). The solution was refluxed for 3 h, and evaporated solvent under reduced pressure. The resulting crude product was purified by flash column chromatography on silica gel to afford the desired product. 85% yield; white solid; ^1H NMR (400 MHz, $CDCl_3$) δ 5.41 (s, 1H), 3.93 (s, 3H), 3.00 (s, 3H); The structure was further confirmed by spectral comparison with literature data.[25]

Synthesis of *N*-substituted 3-methylmaleimide

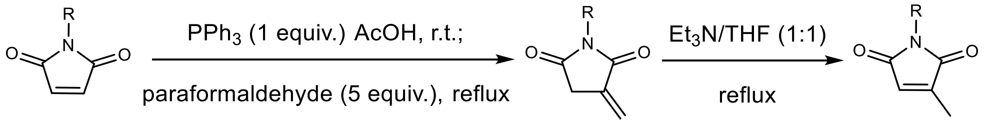

1st step : *N*-methylmaleimide **2a** (0.5 mmol, 1.0 equiv.) and triphenylphosphine (1.0 equiv.) was dissolved in glacial acetic acid (1.25 mL), and the mixture was stirred for 30 min. Paraformaldehyde (5.0 equiv.) was added dropwise, and the reaction mixture was refluxed for 1 h. The mixture was concentrated under reduced pressure and extracted with EtOAc. The combined organic layer was dried over Na_2SO_4, filtered and concentrated *in vacuo*. The resulting crude product was purified by flash column chromatography on silica gel to afford the desired product.

2nd step : To a solution of 3-methylenepyrrolidine-2,5-dione (2.79 mmol, 1.0 equiv.) in THF (3 mL) was added Et_3N (3 mL). The solution was refluxed for overnight, and then the mixture was concentrated *in vacuo*. The reaction mixture was washed with water and extracted with EtOAc. The combined organic layer was dried over Na_2SO_4, filtered and concentrated under reduced pressure. The resulting crude product was purified by flash column chromatography on silica gel to afford the desired product.

1-methyl-3-methylenepyrrolidine-2,5-dione (2′q)

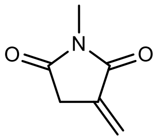

Prepared according to the above procedure using *N*-methylmaleimide **2a** (0.5 mmol, 1.0 equiv.). 93% yield; white solid; ^{1}H NMR (400 MHz, $CDCl_3$) δ 6.35 (m, 1H), 5.64 (m, 1H), 3.34 (m, 2H), 3.08 (s, 3H); The structure was further confirmed by spectral comparison with literature data.[26]

1,3-dimethyl-1*H*-pyrrole-2,5-dione (2q)

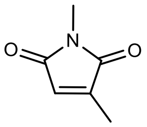

Prepared according to the above procedure using 1-methyl-3-methylenepyrrolidine-2,5-dione **2′q** (2.79 mmol, 1.0 equiv.). 53% yield; colorless oil; ^{1}H NMR (400 MHz, $CDCl_3$) δ 6.33 (q, *J* = 1.9 Hz, 1H), 3.00 (s, 3H), 2.08 (d, *J* = 1.9 Hz, 3H); The structure was further confirmed by spectral comparison with literature data.[27]

3-methylene-1-phenylpyrrolidine-2,5-dione (2′r)

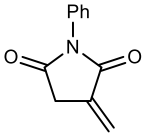

Prepared according to the above procedure using *N*-phenylmaleimide (0.3 mmol, 1.0 equiv.). 99% yield; pale yellow solid; ^{1}H NMR (400 MHz, $CDCl_3$) δ 7.49 (ddd, 2H, *J* = 7.8, 6.5, 1.3 Hz), 7.44 – 7.38 (m, 1H), 7.36 – 7.32 (m, 3H), 6.49 (t, 1H, *J* = 2.5 Hz), 5.75 (t, 1H, *J* = 2.1 Hz), 3.52 (t, 2H, *J* = 2.3 Hz); The structure was further confirmed by spectral comparison with literature data.[28]

3-methyl-1-phenyl-1*H*-pyrrole-2,5-dione (2r)

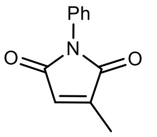

Prepared according to the above procedure using 3-methylene-1-phenylpyrrolidine-2,5-dione **2′r**. 75% yield; pale yellow solid; ^{1}H NMR (400 MHz, $CDCl_3$) δ 7.51 – 7.41 (m, 2H), 7.40 – 7.32 (m, 3H), 6.48 (q, 1H, *J* = 1.8 Hz), 2.18 (d, 3H, *J* = 1.8 Hz); The structure was further confirmed by spectral comparison with literature data.[29]

Synthesis of 1,3,4-trimethyl-1*H*-pyrrole-2,5-dione (2s)

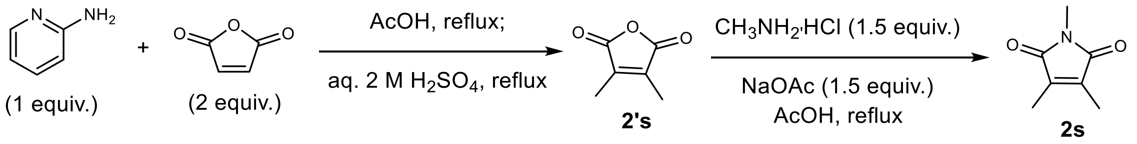

3,4-dimethylfuran-2,5-dione (2′s)

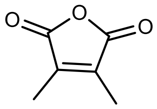

A solution of 2-aminopyridine (12.0 mmol, 1.0 equiv.) in acetic acid (3 mL, 4 M) was refluxed for 1 h, then a solution of maleic anhydride (2.0 equiv.) in acetic acid (3 mL, 4 M) was added. The reaction mixture was refluxed for 3 hours. The solvent was evaporated under reduced pressure, aqueous 2 M sulfuric acid was added. Reaction mixture was refluxed for 2 hours. The reaction mixture was washed with 1 N aqueous HCl solution and extracted with CH_2Cl_2. The combined organic layer was dried over Na_2SO_4, filtered and concentrated. The crude is used without further purification. 60% yield; white solid; ^{1}H NMR (400 MHz, $CDCl_3$) δ 2.08 (s, 6H); The structure was further confirmed by spectral comparison with literature data.[30]

1,3,4-trimethyl-1*H*-pyrrole-2,5-dione (2s)

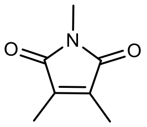

To a solution of 3,4-dimethylfuran-2,5-dione **2′s** (2.4 mmol, 1.0 equiv.) in AcOH (8 mL, 0.3 M) was added methylamine hydrochloride (1.5 equiv.) and sodium Acetate (1.5 equiv.). The reaction mixture was stirred at 100°C for 6 hours. The mixture was concentrated *in vacuo* and, washed with saturated aqueous $NaHCO_3$ and extracted with EtOAc. The combined organic layer was dried over Na_2SO_4, filtered and concentrated. The crude

material was purified by flash chromatography. 72% yield; white solid; ^{1}H NMR (400 MHz, $CDCl_3$) δ 2.99 (s, 3H), 1.96 (s, 6H); The structure was further confirmed by spectral comparison with literature data.[31]

Synthesis of (*S,Z*)-4-((1-methoxy-1-oxopropan-2-yl)amino)-4-oxobut-2-enoic acid (2t)

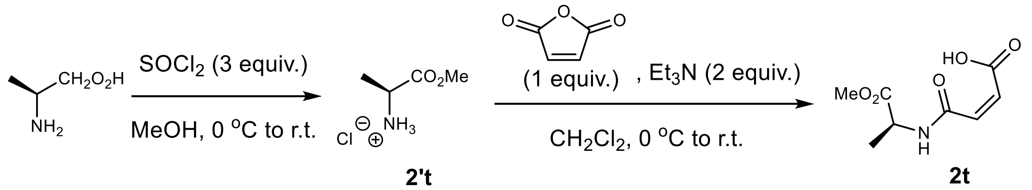

L-Alanine methyl ester hydrochloride (2′t)

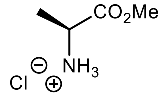

In methanol (3 mL, 1 M), thionyl chloride (3.0 equiv.) was slowly dropped to the solution at 0 °C. The solution was stirred for 1 h at the same temperature. L-Alanine (3 mmol, 1.0 equiv.) was added at 0 °C, and the mixture was allowed to slowly warm to room temperature while being stirred. The reaction was monitored by TLC. When the reaction was completed, the solvent was evaporated *in vacuo*. The crude product was filtered and rinsed with Et_2O several times. The filter cake was dried under air and used without further purification. 89% yield; white solid; ^{1}H NMR (400 MHz, $CDCl_3$): δ 8.71 (br s, 3H), 4.27 (q, J = 7.3 Hz, 1H), 3.82 (s, 3H), 1.73 (d, J = 7.3 Hz, 3H); The structure was further confirmed by spectral comparison with literature data.[32]

(*S,Z*)-4-((1-methoxy-1-oxopropan-2-yl)amino)-4-oxobut-2-enoic acid (2t)

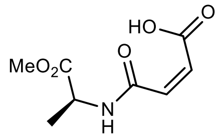

To a mixture of L-alanine methyl ester salt **2′t** (1.0 equiv.) and maleic anhydride (1.0 equiv.), CH_2Cl_2 (0.25 mL, 0.2 M) and Et_3N (2 equiv.) were added at 0 °C. The reaction was stirred at room temperature and monitored by TLC. When the reaction was completed, the solution was concentrated *in vacuo*. The crude product was purified by flash column chromatography on silica gel to afford the desired product. 92% yield; white solid; ^{1}H NMR (400 MHz, $CDCl_3$): δ 7.33 (br s, 1H), 6.41 (d, J = 12.8 Hz, 1H), 6.36 (d, J = 12.9 Hz, 1H), 4.65 (p, J = 7.2 Hz, 1H), 3.81 (s, 3H), 1.52 (d, J = 7.2 Hz, 3H); ^{13}C NMR (100 MHz, $CDCl_3$) δ 172.1, 166.0, 165.8, 136.5, 131.5, 53.0, 49.2, 17.4; MS (APCI): m/z 202.3 $[M+H]^+$

Synthesis of 1-methyl-3-(*p*-tolyl)-1,5-dihydro-2*H*-pyrrol-2-one (2v)

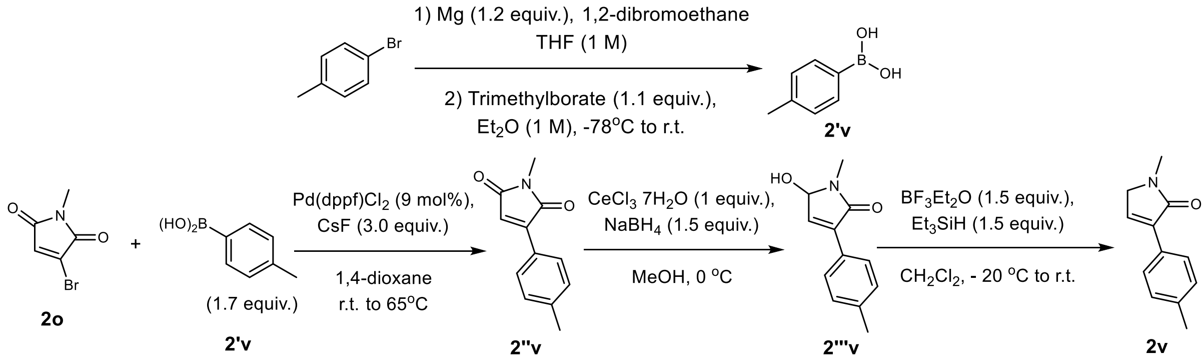

***p*-tolylboronic acid (2′v)**

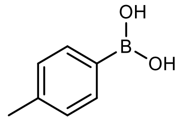

To make Grignard reagent first, put oven dried magnesium (1.2 equiv.) into flask, and dissolved in at least amount of THF (1 M). Using heat-gun, heated up the solution until it was boiling. Quickly added 1 drop of 1,2-dibromoethane and watch if the bubble is shown on surface of magnesium. Then, slowly added about 20% of solution of bromobenzene (1.0 equiv.) dissolved in THF then, slowly added rest of the solution and then warmed to 50 °C until nearly all magnesium wa s dissolved. The Grignard solution was added to another solution of trimethylbromate (1.1 equiv.) in diethyl ether (1 M) at -78 °C under inert atmosphere. The mixture was stirred for 30 minutes and cool bath was removed then stirred 2 h at room temperature. Then, the boronic ester was hydrolyzed by aqua. 1 N HCl solution, followed by 1 h stirring.The reaction mixture was extracted with Et_2O, the combined organic layers dried with $MgSO_4$ and evaporated to dryness in vacuo, recrystalized to obtain pure product., 68% yield, white solid ^{1}H NMR (400 MHz, $CDCl_3$) δ 8.13 – 8.12 (d, J = 7.9 Hz, 2H) 7.32 – 7.30 (d, J = 7.5 Hz, 2H), 2.44 (s, 3H); MS (APCI): *m/z* 160.0 $[M+Na]^+$; The structure was further confirmed by spectral comparison with literature data[33].

1-methyl-3-(*p*-tolyl)-1*H*-pyrrole-2,5-dione (2″v)

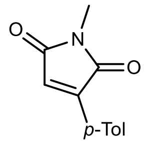

A stirred solution of 3-bromo-1-methyl-1H-pyrrole-2,5-dione (1.0 equiv.) and p-tolylboronic acid (1.7 equiv.) in 1,4-dioxane under nitrogen was degassed with a stream of nitrogen for 10 minutes, treated

with CsF (3.0 equiv.) and Pd(dppf)Cl_2 (9 mol%), then stirred at room temperature for 0.5 h and at 45 °C for 30 minutes, then 65 °C for 45 minutes. The mixture was cooled and diluted with dichloromethane. The mixture was filtered through celite and the brown filtrate concentrated in vacuo. The residue was dissolved in dichloromethane and filtered through a silica column chromatography. 85% yield. yellow solid; ^{1}H NMR (400 MHz, $CDCl_3$) δ 7.84 – 7.82 (d, *J* = 7.6 Hz, 2H), 7.26 – 7.24 (d, *J* = 8.6 Hz, 2H), 6.67 – 6.66 (d, *J* = 1.7 Hz, 1H), 3.06 (s, 3H), 2.40 (s, 3H); ^{13}C NMR (100 MHz, $CDCl_3$) δ 170.9, 170.6, 143.8, 141.7, 129.7, 128.5, 126.0, 122.7, 23.8, 21.6; MS (APCI): *m/z* 202.0 $[M+H]^+$

5-hydroxy-1-methyl-3-(*p*-tolyl)-1,5-dihydro-2*H*-pyrrol-2-one(2′′′v)

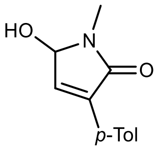

To a solution of 1-methyl-3-(*p*-tolyl)-1*H*-pyrrole-2,5-dione (1.0 equiv.) in methanol was added cerium(III) chloride heptahydrate (1 equiv.), and the mixture was stirred for 5 minutes. After the solution was cooled to 0 °C, $NaBH_4$ (1.5 equiv.) was added portionwise, and stirred for 1 h. The reaction mixture was quenched with ice water, and methanol was removed under reduced pressure. Silica gel column chromatography afforded desired product isolated. 81% yield, white solid; ^{1}H NMR (400 MHz, $CDCl_3$) δ 7.78 – 7.76 (d, *J* = 8.1 Hz, 2H), 7.21 – 7.19 (d, *J* = 8.0 Hz, 2H), 6.97 – 6.96 (d, *J* = 1.5 Hz, 1H), 5.32 – 5.29 (d, *J* = 11.6 Hz, 1H), 3.04 (s, 3H), 2.37 (s, 3H), 1.97 – 1.94 (d, *J* = 11.6 Hz, 1H); ^{13}C NMR (100 MHz, $CDCl_3$) δ 168.9, 139.2, 136.2, 129.8, 129.1, 127.7, 127.2, 127.1, 82.6, 45.6, 37.2, 26.2, 25.2, 21.4, 21.1; MS (APCI): *m/z* 204.1 $[M+H]^+$

1-methyl-3-(*p*-tolyl)-1,5-dihydro-2*H*-pyrrol-2-one(2v)

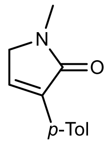

To a stirred solution of 5-hydroxy-1-methyl-3-(*p*-tolyl)-1,5-dihydro-2*H*-pyrrol-2-one (1.0 equiv.) in dry dichloromethane was added triethylsilane (1.5 equiv.). The solution was cooled to -20 °C and BF_3Et_2O (1.5 equiv.) was added dropwise and the resulting mixture was stirred at room temperature. The solvent was evaporated, washed with $NaHCO_3$ and extracted with dichloromethane. The extracts were dried over Na_2SO_4, concentrated and purified by column chromatography to obtain pure product. 100% yield,

white solid; ^{1}H NMR (400 MHz, $CDCl_3$) δ 7.80 – 7.78 (d, J = 8.2 Hz, 2H), 7.20 – 7.18 (d, J = 7.9 Hz, 2H), 7.11 – 7.09 (t, J = 2.1 Hz, 1H), 3.96 - 3.95 (d, J = 1.9 Hz, 2H), 3.10 (s, 3H), 2.38 (s, 3H); ^{13}C NMR (100 MHz, $CDCl_3$) 170.2, 138.3, 137.1, 133.9, 129.09, 129.08, 128.8, 52.1, 29.4, 21.3; MS (APCI): *m/z* 188.0 $[M+H]^+$

Synthesis of 3-cyclopropyl-1-methyl-1*H*-pyrrole-2,5-dione (2y)

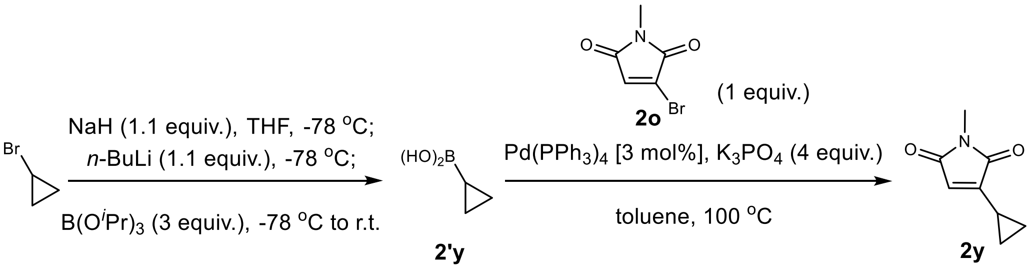

Cyclopropylboronic acid (2′y)

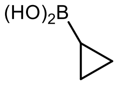

To a suspension of NaH (60% in mineral oil, 1.1 equiv.) in dry THF (2 mL) was added bromocyclopropane (1.5 mmol, 1.0 equiv.) dropwise at -78 °C and stirred for 30 min. A solution of *n*-BuLi (2.5 M in hexane, 1.1 equiv.) was added dropwise and the reaction mixture allowed to stir for another 30 min. followed by addition of a solution of triisopropyl borate (3.0 equiv.) in dry THF (1 mL) dropwise. The reaction mixture was allowed to slowly warm to ambient temperature and stirred for 14 h. An aqueous solution of 1 N HCl was added slowly and the resulting mixture was extracted with EtOAc. The combined organic layers were dried over Na_2SO_4 and concentrated under reduced pressure. The resulting product was usually used without further purification as a brown oil. To obtain pure product, the crude was purified by flash column chromatography on silica gel to afford the desired product as a white solid. 95% yield; ^{1}H NMR (400 MHz, $CDCl_3$) δ 0.68 – 0.63 (m, 2H), 0.58 – 0.54 (m, 2H), -0.16 (tt, J = 9.0, 5.9 Hz, 1H); The structure was further confirmed by spectral comparison with literature data.[34]

3-cyclopropyl-1-methyl-1*H*-pyrrole-2,5-dione (2y)

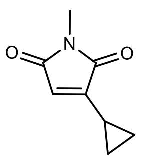

To mixture of $Pd(PPh_3)_4$ (3 mol%), K_3PO_4 (4.0 equiv.) and 3-bromo-1-methyl-1*H*-pyrrole-2,5-dione **2o** (0.1 mmol, 1.0 equiv.) was added a suspension of cyclopropylboronic acid **2′y** (1.1 equiv.) in dry toluene (0.5 mL, 0.2 M)

under nitrogen atmosphere. The reaction mixture was stirred at 100 °C for 2 h. When TLC indicated the reaction was complete, the reaction mixture was diluted in EtOAc and filtered through a pad of celite. The pad was rinsed with an additional EtOAc and the combined filtrate was concentrated *in vacuo*. The resulting crude product was purified by flash column chromatography on silica gel to afford the desired product. 46% yield; white solid; ^{1}H NMR (400 MHz, $CDCl_3$) δ 5.98 (s, 1H), 2.98 (s, 3H), 1.86 (ddd, J = 13.4, 8.3, 5.0 Hz, 1H), 1.19 – 1.14 (m, 2H), 1.01 – 0.97 (m, 2H); ^{13}C NMR (100 MHz, $CDCl_3$) δ 171.2, 170.7, 153.6, 120.9, 23.7, 11.7, 8.4; MS (APCI): *m/z* 152.3 $[M+H]^+$

Preparation and characterization of enynes for intramolecular reaction

PART 1 : Synthesis of alkyne moieties in enyne substrate

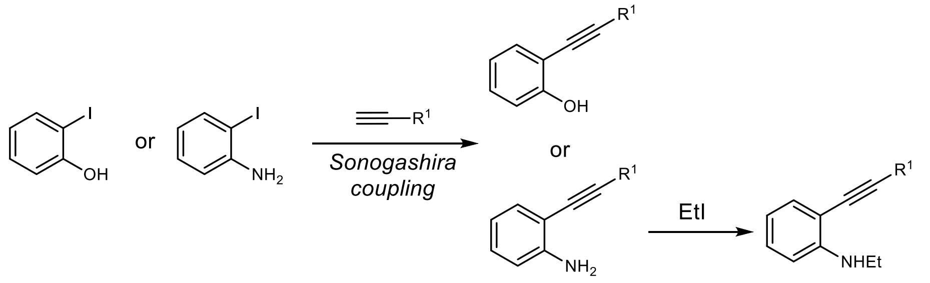

General procedure C (for Sonogashira coupling)

To a suspension of $Pd(PPh_3)_2Cl_2$ (2 mol%), CuI (4 mol%), and 2-iodophenol or 2-iodoaniline (1.0 equiv.) in degassed Et_3N (0.5 M) was added dropwise the corresponding alkyne (2.0 equiv.) under nitrogen atmosphere. The reaction mixture was stirred until TLC indicated complete consumption of the starting material. The reaction mixture was washed with a saturated solution of NH_4Cl and extracted with CH_2Cl_2. The combined organic layer was dried over Na_2SO_4, filtered and concentrated. The crude material was purified by flash chromatography on silica gel.

General procedure D (for ethylation on aniline)

Corresponding aniline (1.0 equiv.) was dissolved in THF (0.2 M) under nitrogen atmosphere and cooled to -78°C. *n*-BuLi solution (2.5 M in hexane, 1.1 equiv.) was added dropwise, and the mixture was stirred for 1h. Iodoethane (1.0 equiv.) was added dropwise at -78 °C, and the reaction mixture was stirred for 1h at room temperature. The reaction mixture was quenched by addition of saturated solution of NH_4Cl and extracted with ethyl acetate. The combined organic layer was dried over Na_2SO_4, filtered and concentrated. The crude material was purified by flash chromatography on silica gel.

2-(*p*-tolylethynyl)phenol (S1)

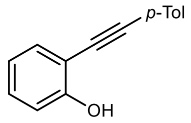

Prepared according to the ***General Procedure C*** using $Pd(PPh_3)_2Cl_2$ (2 mol%), CuI (4 mol%), 2-iodophenol (4.0 mmol, 1.0 equiv.), and *p*-tolylacetylene (2.0 equiv.) in Et_3N (0.5 M) at reflux. 90% yield; pale yellow solid; ^1H NMR (400 MHz, $CDCl_3$) δ 7.45 – 7.39 (m, 3H), 7.29 – 7.23 (m, 1H), 7.18 (dt, *J* = 7.9, 0.7 Hz, 2H), 6.98 (dd, *J* = 8.3, 0.7 Hz, 1H), 6.90 (td, *J* = 7.5, 1.1 Hz, 1H), 5.85 (s, 1H), 2.38 (s, 3H); The structure was further confirmed by spectral comparison with literature data.[35]

2-((trimethylsilyl)ethynyl)phenol (S2)

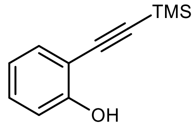

Prepared according to the ***General Procedure C*** using $Pd(PPh_3)_2Cl_2$ (2 mol%), CuI (4 mol%), 2-iodophenol (3.0 mmol, 1.0 equiv.), and trimethylsilylacetylene (1.5 equiv.) in Et_3N (0.5 M) at reflux, 98% yield; colorless oil; ^1H NMR (400 MHz, $CDCl_3$) δ 7.34 (dd, *J* = 7.7, 1.6 Hz, 1H), 7.27 – 7.21 (m, 1H), 6.94 (dd, *J* = 8.3, 0.8 Hz, 1H), 6.85 (td, *J* = 7.6, 1.0 Hz, 1H), 7.11 (d, *J* = 7.9 Hz, 4H), 5.82 (s, 1H), 0.28 (s, 9H); The structure was further confirmed by spectral comparison with literature data.[36]

2-(*p*-tolylethynyl)aniline (S3)

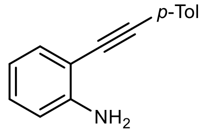

Prepared according to the ***General Procedure C*** using $Pd(PPh_3)_2Cl_2$ (2 mol%), CuI (2 mol%), 2-iodoaniline (3.0 mmol, 1.0 equiv.), and *p*-tolylacetylene (1.2 equiv.) in Et_3N (0.2 M) at room temperature, 99% yield; yellow solid; ^1H NMR (400 MHz, $CDCl_3$) δ 7.42 (d, *J* = 8.1 Hz, 2H), 7.35 (d, *J* = 8.2 Hz, 1H), 7.16 (d, *J* = 7.9 Hz, 2H), 7.12 (dd, *J* = 7.8, 1.2 Hz, 1H), 6.76 – 6.67 (m, 2H) 4.26 (s, 2H), 2.37 (s, 3H); The structure was further confirmed by spectral comparison with literature data.[36]

***N*-ethyl-2-(*p*-tolylethynyl)aniline (S4)**

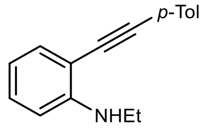

Prepared according to the ***General Procedure D*** using 2-(*p*-tolylethynyl)aniline **S3** (1.2 mmol, 1.0 equiv.). 96% yield; yellow oil; ^{1}H NMR (400 MHz, $CDCl_3$) δ 7.41 (d, *J* = 8.0 Hz, 2H), 7.35 (dd, *J* = 7.4, 1.5 Hz, 1H), 7.20 (ddd, *J* = 8.9, 8.0, 1.6 Hz, 1H), 7.16 (d, *J* = 7.8 Hz, 2H), 6.66 – 6.60 (m, 2H), 4.56 (br s, 1H), 3.24 (qd, *J* = 7.2, 5.5 Hz, 1H), 2.37 (s, 3H), 1.32 (t, *J* = 7.1 Hz, 3H); ^{13}C NMR (100 MHz, $CDCl_3$) δ 149.0, 138.4, 132.2, 131.5, 130.0, 129.3, 120.5, 116.3, 109.6, 107.6, 95.3, 85.5, 38.2, 21.6, 15.0; MS (APCI): *m/z* 236.2 $[M+H]^+$

2-(phenylethynyl)aniline (1x)

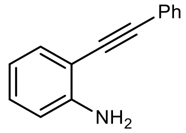

Prepared according to the ***General Procedure C*** using $Pd(PPh_3)_2Cl_2$ (5 mol%), CuI (5 mol%), 2-iodoaniline (1.5 mmol, 1.0 equiv.), and phenylacetylene (1.2 equiv.) in Et_3N (5 mL) at 70 °C. 88% yield; brown solid; ^{1}H NMR (400 MHz, $CDCl_3$) δ 7.57 – 7.52 (m, 2H), 7.43 – 7.31 (m, 4H), 7.19 – 7.12 (m, 1H), 6.77 – 6.71 (m, 2H), 4.28 (s, 2H); ^{13}C NMR (100 MHz, $CDCl_3$) δ 147.8, 132.2, 131.5, 129.7, 128.4, 128.2, 123.3, 118.0, 114.3, 107.9, 94.7, 85.9; MS (APCI): *m/z* 194.1 $[M+H]^+$

***N*-ethyl-2-(phenylethynyl)aniline (S5)**

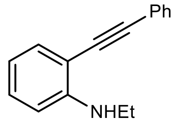

Prepared according to the ***General Procedure D*** using 2-(phenylethynyl)aniline **1x** (1.5 mmol, 1.0 equiv.) and iodoethane (0.9 equiv.) in THF (0.38 M). 75% yield; light yellow oil; ^{1}H NMR (400 MHz, $CDCl_3$) δ 7.52 (dddd, *J* = 5.2, 3.7, 2.2, 1.4 Hz, 2H), 7.40 – 7.33 (m, 4H), 7.25 – 7.20 (m, 1H), 6.68 – 6.61 (m, 2H), 4.57 (s, 1H), 3.26 (qd, *J* = 7.1, 5.2 Hz, 2H), 1.33 (t, *J* = 7.1 Hz, 3H); ^{13}C NMR (100 MHz, $CDCl_3$) δ 149.0, 132.1, 131.4, 131.4, 130.0, 128.4, 128.1, 123.4, 116.1, 107.2, 95.0, 86.1, 38.0, 14.8; MS (APCI): *m/z* 222.1 $[M+H]^+$

2-(pyridin-2-ylethynyl)aniline (S6′)

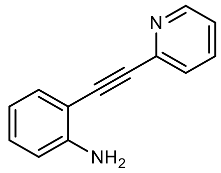

Prepared according to the ***General Procedure C*** using $Pd(PPh_3)_2Cl_2$ (5 mol%), CuI (10 mol%), 2-iodoaniline (1.1 equiv.), and 2-ethylnylpyridine (1.1 mmol, 1.0 equiv.) in Et_3N/THF (2.5 mL/4 mL) at 30 °C. 78% yield; brown solid; ^{1}H NMR (400 MHz, $CDCl_3$) δ 8.61 (ddd, *J* = 4.9, 1.8, 0.9 Hz, 1H), 7.71 – 7.64 (m, 1H), 7.51 (dt, *J* = 7.9, 1.1 Hz, 1H), 7.41 (dd, *J* = 8.2, 1.5 Hz, 1H), 7.23 (ddt, *J* = 7.6, 4.9, 0.8 Hz, 1H), 7.19 – 7.11 (m, 1H), 6.76 – 6.66 (m, 2H), 4.38 (s, 2H); ^{13}C NMR (100 MHz, $CDCl_3$) δ 150.0, 148.5, 143.6, 136.1, 132.6, 130.5, 127.0, 122.6, 117.8, 114.4, 106.6, 94.1, 86.2; MS (APCI): *m/z* 195.1 $[M+H]^+$

N-ethyl-2-(pyridin-2-ylethynyl)aniline (S6)

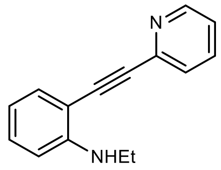

Prepared according to the ***General Procedure D*** using 2-(pyridin-2-ylethynyl)aniline **S6′** (0.5 mmol, 1.0 equiv.) and iodoethane (0.9 equiv.). 79% yield; brown oil; ^{1}H NMR (400 MHz, $CDCl_3$) δ 8.62 (ddd, *J* = 4.9, 1.9, 0.9 Hz, 1H), 7.67 (td, *J* = 7.7, 1.8 Hz, 1H), 7.50 (dt, *J* = 7.9, 1.1 Hz, 1H), 7.42 (dd, *J* = 7.6, 1.6 Hz, 1H), 7.27 – 7.19 (m, 2H), 6.68 – 6.59 (m, 2H), 4.71 (s, 1H), 3.26 (q, *J* = 7.2 Hz, 2H), 1.31 (t, *J* = 7.2 Hz, 3H); ^{13}C NMR (100 MHz, $CDCl_3$) δ 150.0, 149.4, 143.7, 136.1, 132.8, 130.7, 127.0, 122.5, 116.0, 109.5, 106.0, 94.4, 86.5, 37.9, 14.7; MS (APCI): *m/z* 223.1 $[M+H]^+$

Synthesis of 4-ethynylisoquinoline (S7″)

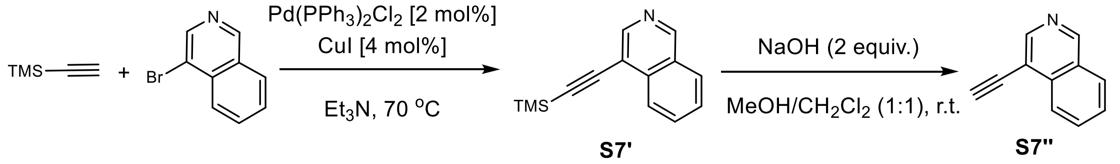

4-((trimethylsilyl)ethynyl)isoquinoline (S7′)

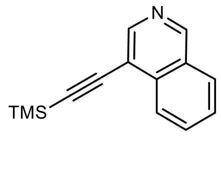

In a three neck round bottom flask, 4-bromoisoquinoline (1.4 mmol, 1.0 equiv.), $Pd(PPh_3)_2Cl_2$ (2 mol%) and CuI (4 mol%) were added. The flask was flushed with nitrogen and Et_3N (5 mL) was added into the flask while stirring at 30 °C, followed by trimethylsilylacetylene (1.68 mmol, 1.2 equiv.). After 5 h at 70 °C, the reaction mixture was quenched with water, extracted with CH_2Cl_2, dried with anhydrous Na_2SO_4 and filtered. The solvent was removed under reduced pressure and the crude product was purified by flash chromatography. 88% yield; yellow oil; ^{1}H NMR (400 MHz, $CDCl_3$) δ 9.17 (s, 1H), 8.70 (s, 1H), 8.22 (ddt, *J* = 8.3, 1.7, 0.9 Hz, 1H), 7.95 (ddd, *J* = 8.1, 2.4, 1.1 Hz, 1H), 7.77 (dddd, *J* = 8.2, 6.9, 2.0, 1.2 Hz, 1H), 7.62 (tdd, *J* = 8.1, 2.4, 1.2 Hz, 1H), 0.34 (d, *J* = 0.9 Hz, 9H). The structure was further confirmed by spectral comparison with literature data.[37]

4-ethynylisoquinoline (S7″)

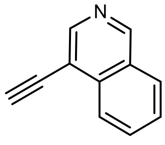

To a stirred solution of 4-((trimethylsilyl)ethynyl)isoquinoline **S7′** (1.1 mmol, 1.0 equiv.) in anhydrous $MeOH/CH_2Cl_2$ (5/5 mL), was added sodium hydroxide (2.2 mmol, 2.0 equiv.) under nitrogen atmosphere. After 2 h, the reaction mixture was quenched with water; extracted with CH_2Cl_2, dried with Na_2SO_4 and filtered. The solvent was removed under reduced pressure and the residue was purified by flash chromatography. 85% yield; light brown solid; ^{1}H NMR (400 MHz, $CDCl_3$) δ 9.10 (d, *J* = 4.2 Hz, 1H), 8.66 (d, *J* = 2.8 Hz, 1H), 8.12 (dd, *J* = 8.6, 4.4 Hz, 1H), 7.83 (t, *J* = 6.9 Hz, 1H), 7.73 – 7.58 (m, 1H), 7.58 – 7.43 (m, 1H), 3.52 (s, 1H). The structure was further confirmed by spectral comparison with literature data.[37]

2-(isoquinolin-4-ylethynyl)aniline (S7‴)

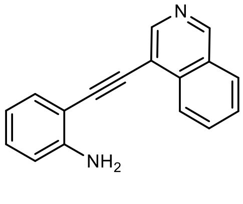

Prepared according to the ***General Procedure C*** using $Pd(PPh_3)_2Cl_2$ (2 mol%), CuI (4 mol%), 2-iodoaniline (0.9 mmol, 1.0 equiv.), and 4-ethynylisoquinoline **S7″** (1.2 equiv.) in Et_3N/THF (5 mL) at 30 °C. 79% yield; brown solid; ^{1}H NMR (400 MHz, $CDCl_3$) δ 9.18 (s, 1H), 8.76 (s, 1H), 8.30 (dt, *J* = 8.4, 0.9 Hz, 1H), 7.97 (dt, *J* = 8.2, 1.0 Hz, 1H), 7.78 (ddd, *J* = 8.3, 6.9, 1.3 Hz, 1H), 7.64 (ddd, *J* = 8.1, 6.9, 1.1 Hz, 1H), 7.48 (dd, *J* = 7.9, 1.6 Hz, 1H), 7.23 – 7.13 (m, 1H), 6.82 – 6.70 (m, 2H), 4.41 (s, 2H); ^{13}C NMR (100 MHz, $CDCl_3$) δ 151.9, 148.0, 146.2, 135.3, 132.4, 131.2, 130.3, 128.0, 127.9, 127.8, 125.0, 118.1, 116.1, 114.6, 107.4, 93.4, 89.6; MS (APCI): *m/z* 245.1 $[M+H]^+$

N-ethyl-2-(isoquinolin-4-ylethynyl)aniline (S7)

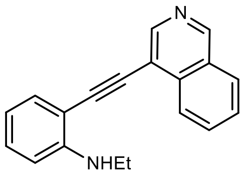

Prepared according to the ***General Procedure D*** using 2-(isoquinolin-4-ylethynyl)aniline **S7‴** (0.35 mmol, 1.0 equiv.) and iodoethane (0.9 equiv.). 75% yield; brown oil; ^{1}H NMR (400 MHz, $CDCl_3$) δ 9.20 (s, 1H), 8.76 (s, 1H), 8.33 (dd, *J* = 8.3, 1.1 Hz, 1H), 8.02 (d, *J* = 8.1 Hz, 1H), 7.81 (ddd, *J* = 8.3, 6.8, 1.3 Hz, 1H), 7.68 (ddd, *J* = 8.1, 6.8, 1.1 Hz, 1H), 7.49 (dd, *J* = 7.6, 1.6 Hz, 1H), 7.31 – 7.26 (m, 1H), 6.76 – 6.61 (m, 2H), 4.65 (s, 1H), 3.29 (p, *J* = 6.4, 5.7 Hz, 2H), 1.36 (t, *J* = 7.1 Hz, 3H); ^{13}C NMR (100 MHz, $CDCl_3$) δ 151.8, 149.1, 146.2, 135.4, 132.3, 131.1, 130.6, 128.0, 127.88, 127.85, 125.0, 116.3, 116.1, 109.7, 106.8, 93.7, 90.0, 38.1, 14.8; MS (APCI): *m/z* 273.2 $[M+H]^+$

2-(hex-1-yn-1-yl)aniline (S8)

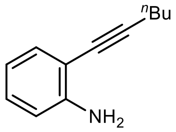

Prepared according to the ***General Procedure C*** using $Pd(PPh_3)_2Cl_2$ (2 mol%), CuI (1 mol%), 2-iodoaniline (0.5 mmol, 1.0 equiv.), and 1-Hexyne (1.2 equiv.) in Et_3N (1 mL, 0.5 M) at room temperature, 83% yield; brown liquid; ^{1}H NMR (400 MHz, $CDCl_3$) δ 7.24 (dd, *J* = 7.6, 1.5 Hz, 1H), 7.10 – 7.04 (m, 1H), 6.70 – 6.61 (m, 2H), 4.15 (s, 1H), 2.47 (t, *J* = 7.0 Hz, 2H), 1.66 – 1.56 (m, 2H), 1.55 – 1.41 (m, 2H), 0.95 (t, *J* = 7.3 Hz, 3H); The structure was further confirmed by spectral comparison with literature data.[38]

2-(naphthalen-2-ylethynyl)aniline (S9′)

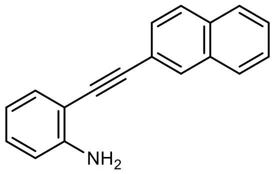

Prepared according to the ***General Procedure C*** using $Pd(PPh_3)_2Cl_2$ (2 mol%), CuI (4 mol%), 2-iodoaniline (0.27 mmol, 1.0 equiv.), and 2-ethynylnaphthalene (1.2 equiv.) in Et_3N (0.68 mL,0.4 M) at room temperature, 96% yield; white solid; ^{1}H NMR (400 MHz, $CDCl_3$) δ 8.05 (s, 1H), 7.85 –7.80 (m, 3H), 7.58 (dd, *J* = 8.5, 1.6 Hz, 1H), 7.53

– 7.48 (m, 2H), 7.45 – 7.38 (m, 1H), 7.16 (ddd, J = 8.2, 7.4, 1.6 Hz, 1H), 6.79 – 6.70 (m, 2H), 4.33 (s, 2H); The structure was further confirmed by spectral comparison with literature data.[39]

N-ethyl-2-(naphthalen-2-ylethynyl)aniline (S9)

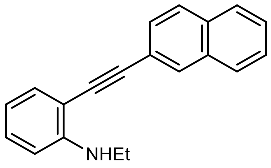

Prepared according to the ***General Procedure D*** using 2-(naphthalen-2-ylethynyl)aniline **S9′** (0.25 mmol, 1.0 equiv.). 84% Yield; ^{1}H NMR (400 MHz, $CDCl_3$) δ 8.03 (s, 1H), 7.84 – 7.74 (m, 3H), 7.56 (dd, J = 8.5, 1.6 Hz, 1H), 7.52 – 7.46 (m, 2H), 7.41 (dd, J = 7.6, 1.5 Hz, 1H), 7.27 – 7.18 (m, 1H), 6.70 – 6.60 (m, 2H), 4.63 (s, 1H), 3.32 – 3.20 (m, 2H), 1.34 (t, J = 7.1 Hz, 3H); ^{13}C NMR (100 MHz, $CDCl_3$) δ 149.0, 133.0, 132.7, 132.2, 131.0, 130.1, 128.3, 128.0, 127.8, 127.7, 126.6, 126.6, 120.7, 116.2, 109.5, 107.2, 95.4, 86.5, 38.1, 14.8; MS (APCI): m/z 272.0 $[M+H]^+$

Part 2 : Synthesis of ester and amide for enyne substrates

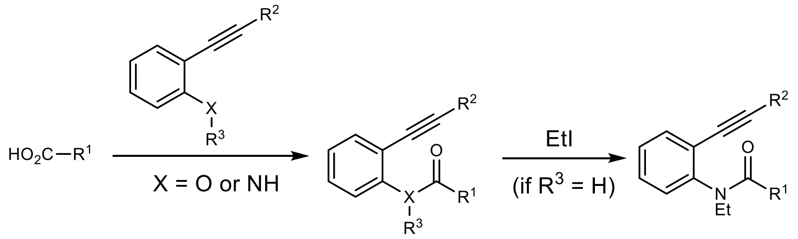

General Procedure E (for ester and amide synthesis via acid chloride)

To a suspension of the corresponding acid (1.2 equiv.), DMF (3 mol%) in dry CH_2Cl_2 (0.5 M) was added dropwise oxalyl chloride (1.1 equiv., per acid) at 0°C. The reaction mixture was stirred at room temperature for 1h. Then, the solvent was removed under reduced pressure. The residue was dissolved in dry THF (0.3 M) and slowly added dropwise to a solution of the appropriate phenol or aniline (1.0 equiv.) and Et_3N (1.2 equiv.) at 0°C. The mixture was stirred at room temperature until TLC indicated complete consumption of the starting material. The reaction mixture was diluted with water and extracted with CH_2Cl_2. The combined organic layer was dried over Na_2SO_4, filtered and concentrated. The crude material was purified by flash chromatography on silica gel.

General Procedure F (for amide synthesis via mixed anhydride)

To a solution of the corresponding acid (1.0 equiv.), and Et_3N (1.2 equiv.) was dissolved in THF (0.15 M) under nitrogen atmosphere and cooled to 0°C. Isobutyl chloroformate (1.0 equiv.) was added dropwise, and the mixture

was stirred for 30 min at 0°C. The appropriate amine (1.0 equiv.) in solvent was added dropwise at 0°C, and the reaction mixture was stirred at room temperature. The solvent was evaporated under reduced pressure. The crude material was purified by flash chromatography on silica gel.

General Procedure G **(for ethylation of amide)**

To solution of the corresponding amide (1.0 equiv.) in THF (0.3 M) was added sodium hydride (1.5 equiv.) at 0°C. The reaction mixture was stirred at room temperature for 1 hour. Then iodoethane (2.9 equiv.) was added dropwise. The reaction mixture was monitored by TLC. The reaction mixture was washed with a saturated solution of NH_4Cl and extracted with EtOAc. The combined organic layer was dried over Na_2SO_4, filtered and concentrated. The crude material was purified by flash chromatography on silica gel.

2-(*p*-tolylethynyl)phenyl (*E*)-3-(*p*-tolyl)acrylate (4a)

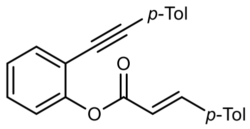

Prepared according to the ***General Procedure E*** using 4-methylcinnamic acid (1.2 equiv.) and 2-(*p*-tolylethynyl)phenol **S1** (1.2 mmol, 1.0 equiv.), 94% yield; yellow solid; ^{1}H NMR (400 MHz, $CDCl_3$) δ 7.94 (d, *J* = 16.0 Hz, 1H), 7.59 (dd, *J* = 7.7, 1.6 Hz, 1H), 7.49 (d, *J* = 8.1 Hz, 2H), 7.37 (td, *J* = 7.8, 1.6 Hz, 1H), 7.32 (d, *J* = 8.2 Hz, 2H), 7.29 – 7.18 (m, 4H), 7.04 (d, *J* = 7.9 Hz, 2H), 6.67 (d, *J* = 16.0 Hz, 1H), 2.41 (s, 3H), 2.31 (s, 3H); ^{13}C NMR (100 MHz, $CDCl_3$) δ 165.1, 151.8, 147.0, 141.3, 138.7, 132.9, 131.6, 131.6, 129.9, 129.3, 129.2, 128.5, 125.9, 122.5, 120.0, 117.8, 116.1, 94.8, 84.0, 21.7, 21.6; MS (APCI): *m/z* 353.3 $[M+H]^+$

2-((trimethylsilyl)ethynyl)phenyl (*E*)-3-(*p*-tolyl)acrylate (4b)

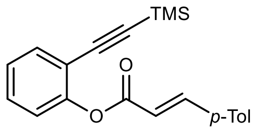

Prepared according to the ***General Procedure E*** using 4-methylcinnamic acid (1.2 equiv.) and 2-((trimethylsilyl)ethynyl)phenol **S2** (0.14 mmol, 1.0 equiv.), 72% yield; white solid; ^{1}H NMR (400 MHz, $CDCl_3$) δ 7.90 (d, *J* = 16.0 Hz, 1H), 7.52 (dd, *J* = 7.7, 1.7 Hz, 1H), 7.49 (d, *J* = 8.1 Hz, 2H), 7.37 (td, *J* = 7.8, 1.7 Hz, 1H), 7.24 (d, *J* = 8.1 Hz, 2H), 7.21 (dd, *J* = 7.6, 1.1 Hz, 1H), 7.04 (d, *J* = 7.9 Hz, 2H), 7.17 (dd, *J* = 8.1, 1.1 Hz, 1H), 6.62 (d, *J* = 16.0 Hz, 1H), 2.40 (s, 3H), 0.15 (s, 9H); ^{13}C NMR (100 MHz, $CDCl_3$) δ 164.7, 152.3, 146.7, 141.1, 133.0, 131.5, 129.7, 129.6, 128.2, 125.7, 122.3, 117.4, 116.0, 99.9, 99.7, 21.5, -0.2; MS (APCI): *m/z* 335.2 $[M+H]^+$

(*E*)-3-(*p*-tolyl)-*N*-(2-(*p*-tolylethynyl)phenyl)acrylamide (4′c)

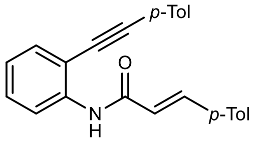

Prepared according to the ***General Procedure E*** using 4-methylcinnamic acid (1.2 equiv.), and 2-(*p*-tolylethynyl)aniline **S3** (0.5 mmol, 1.0 equiv.). 82% yield; yellow solid; ^{1}H NMR (400 MHz, $CDCl_3$) δ 8.57 (d, *J* = 8.1 Hz, 1H), 8.18 (s, 1H), 7.73 (d, *J* = 15.6 Hz, 1H), 7.51 (d, *J* = 7.8 Hz, 1H), 7.48 (d, *J* = 7.9 Hz, 2H), 7.44 (d, *J* = 7.8 Hz, 2H), 7.38 (t, *J* = 7.7 Hz, 1H), 7.21 (t, *J* = 8.5 Hz, 4H), 7.08 (t, *J* = 7.5 Hz, 1H), 6.51 (d, *J* = 15.6 Hz, 1H), 2.43 (s, 3H), 2.38 (s, 3H); ^{13}C NMR (100 MHz, $CDCl_3$) δ 164.0, 142.4, 140.4, 139.3, 131.7, 131.6, 131.5, 129.6, 129.6, 129.4, 128.0, 123.4, 119.9, 119.5, 119.5, 119.3, 96.8, 83.8, 21.6, 21.5; MS (APCI): *m/z* 352.3 $[M+H]^+$

(*E*)-*N*-ethyl-3-(*p*-tolyl)-*N*-(2-(*p*-tolylethynyl)phenyl)acrylamide (4c)

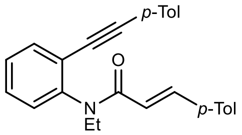

Prepared according to the ***General Procedure G*** using (*E*)-3-(*p*-tolyl)-*N*-(2-(*p*-tolylethynyl)phenyl)acrylamide **4′c** (0.2 mmol, 1.0 equiv.). >99% yield; white solid; ^{1}H NMR (400 MHz, $CDCl_3$): δ 7.65 (d, *J* = 15.4 Hz, 1H), 7.64 – 7.62 (m, 1H), 7.42 – 7.37 (m, 2H), 7.36 (d, *J* = 8.1 Hz, 2H), 7.25 – 7.23 (m, 1H), 7.18 (d, *J* = 8.1 Hz, 2H), 7.11 (d, *J* = 8.1 Hz, 2H), 7.05 (d, *J* = 8.0 Hz, 2H), 6.18 (d, *J* = 15.5 Hz, 1H), 4.08 (dq, *J* = 14.3, 7.2 Hz, 1H), 3.85 (dq, *J* = 14.2, 7.2 Hz, 1H), 2.34 (s, 3H), 2.29 (s, 3H), 1.21 (t, *J* = 7.2 Hz, 3H); ^{13}C NMR (100 MHz, $CDCl_3$) δ 166.0, 143.2, 141.5, 139.5, 138.9, 132.9, 132.7, 131.5, 129.6, 129.3, 129.1, 129.0, 127.9, 127.8, 123.9, 119.6, 118.3, 95.3, 85.1, 44.0, 21.5, 21.3, 13.1; MS (APCI): *m/z* 380.3 $[M+H]^+$

synthesis of (*E*)-3-(2-bromophenyl)acryloyl chloride (4″d)

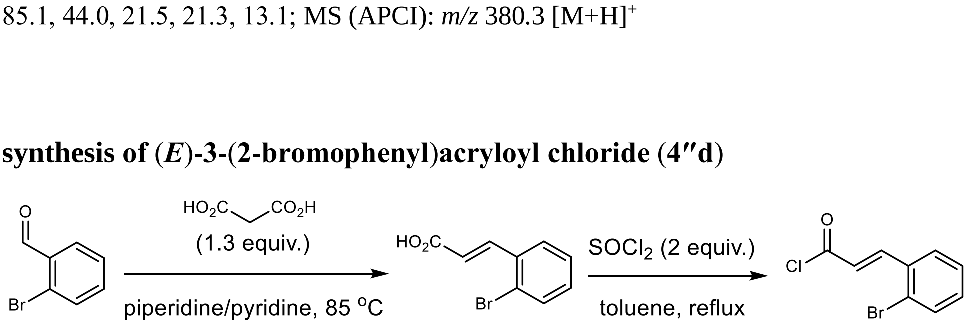

4'd **4"d**

(*E*)-3-(2-bromophenyl)acrylic acid (4′d)

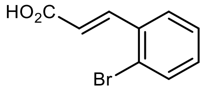

To a stirred solution of malonic acid (2.16 mmol, 1.3 equiv.) in pyridine (0.5 mL), and piperidine (0.08 mL), 2-bromobenzaldehyde (1.66 mmol, 1.0 equiv.) was added slowly at 85°C, the resulting mixture was stirred for 18 h. After cooling to r.t., reaction was neutralized with 10% HCl under ice water, observing crystallization of white solid, which was filtered and washed with cool water and dried to yield desired product. 90% yield; white crystalline solid; ^{1}H NMR (400 MHz, DMSO-d$_6$) δ 7.85 – 7.76 (m, 2H), 7.60 (dd, *J* = 8.0, 1.3 Hz, 1H), 7.35 (ddd, *J* = 8.5, 7.5, 1.4 Hz, 1H), 7.26 (td, *J* = 7.7, 1.7 Hz, 1H), 6.51 (d, *J* = 15.9 Hz, 1H). The structure was further confirmed by spectral comparison with literature data.[40]

(*E*)-3-(2-bromophenyl)acryloyl chloride (4″d)

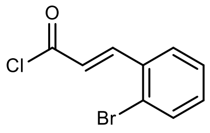

To a stirred solution of (*E*)-3-(2-bromophenyl)acrylic acid **4′d** (1.29 mmol, 1.0 equiv.) in anhydrous toluene (3 mL) was added thionyl chloride (2.58 mmol, 2.0 equiv.) in dropwise manner under nitrogen atmosphere. The reaction mixture was heated to reflux for 5 h, until the consumption of all starting materials. It was then cooled to r.t., excess thionyl chloride was evaporated in rotary and the crude residue was used directly for the next step without further purification. 91% crude yield; white solid.

(*E*)-3-(2-bromophenyl)-*N*-ethyl-*N*-(2-(phenylethynyl)phenyl)acrylamide (4d)

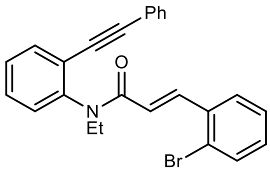

Prepared according to the ***General Procedure E*** using (*E*)-3-(2-bromophenyl)acryloyl chloride **4″d** prepared by above procedure (1.2 equiv.), Et_3N (2.0 equiv.) and *N*-ethyl-2-(phenylethynyl)aniline **S5** (0.5 mmol, 1.0 equiv.) in CH_2Cl_2 (5 mL, 0.1 M). 82% yield; brown solid; ^{1}H NMR (400 MHz, $CDCl_3$) δ 8.01 (d, *J* = 15.4 Hz, 1H), 7.66 – 7.61 (m, 1H), 7.55 – 7.46 (m, 3H), 7.44 – 7.35 (m, 2H), 7.35 – 7.25 (m, 4H), 7.20 (dd, *J* = 7.7, 1.8 Hz, 1H), 7.10 (dtd, *J* = 24.0, 7.4, 1.6 Hz, 2H), 6.21 (d, *J* = 15.4 Hz, 1H), 4.06 – 3.89 (m, 2H), 1.23 (t, *J* = 7.2 Hz, 3H); ^{13}C NMR (100 MHz, $CDCl_3$) δ 165.3, 143.1, 140.1, 135.7, 133.1, 132.9, 131.7, 130.2, 129.4, 129.3, 128.7, 128.4, 128.0, 127.8, 127.3, 125.0, 123.8, 122.5, 122.4, 95.2, 85.7, 44.4, 13.0; MS (APCI): *m/z* 430.1 $[M+H]^+$

Synthesis of *N*-phenyl-2-(*p*-tolylethynyl)aniline (4″e)

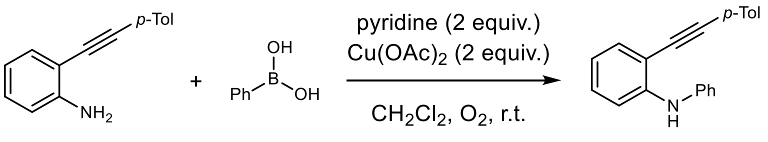

S3 **4'e** **4"e**

Phenylboronic acid (4′e)

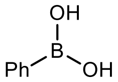

To make Grignard reagent first, put oven dried magnesium (1.2 equiv.) into flask, and dissolved in at least amount of THF (1 M). Using heat-gun, heated up the solution until it was boiling. Quickly added 1 drop of 1,2-dibromoethane and watch if the bubble is shown on surface of magnesium. Then, slowly added about 20% of solution of bromobenzene dissolved in THF then, slowly added rest of the solution and then warmed to 50 °C until nearly all magnesium was dissolved. The Grignard solution was added to another solution of trimethylbormate in Et_2O at -78 °C under inert atmosphere. The mixture was stirred for 30 min. and cool bath was removed then stirred overnight at room temperature. Then, the boronic ester was hydrolyzed by 1 N HCl solution, followed by 1 h stirring. The reaction mixture was extracted with Et_2O, the combined organic layers dried over Na_2SO_4 and evaporated to dryness *in vacuo*, and without purification, use crude residue can be converted directly into next step. 92% yield; white solid; ^{1}H NMR (400 MHz, $CDCl_3$) δ 8.26 – 8.24 (m, 2H), 7.63 – 7.59 (m, 1H), 7.54 – 7.50 (m, 2H); The structure was further confirmed by spectral comparison with literature data.[41]

***N*-phenyl-2-(*p*-tolylethynyl)aniline (4″e)**

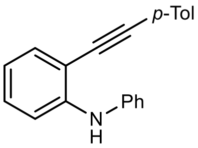

2-(*p*-tolylethynyl)aniline **S3** was dissolved in CH_2Cl_2 at room temperature. Phenylboronic acid **4′e** (2.0 equiv.), pyridine (2.0 equiv.) and copper diacetate (2.0 equiv.) were added and the reaction mixture was stirred at room temperature under O_2 atmosphere (balloon) for overnight. The reaction mixture was partitioned between aqueous copper sulfate and CH_2Cl_2, dried over Na_2SO_4 and concentrated under reduced pressure. The crude was purified by flash chromatography on silica gel. 81% yield; yellow liquid; ^{1}H NMR (400 MHz, $CDCl_3$) δ 7.47 – 7.41 (m, 3H), 7.35 – 7.31 (m, 2H), 7.26 – 7.16 (m, 6H), 7.05 – 7.00 (m, 1H), 6.83 – 6.80 (m, 1H), 6.52 (s, 1H), 2.37 (s, 3H); The structure was further confirmed by spectral comparison with literature data.[42]

(*E*)-*N*-phenyl-3-(*p*-tolyl)-*N*-(2-(*p*-tolylethynyl)phenyl)acrylamide (4e)

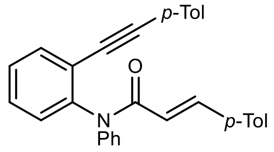

Prepared according to the ***General Procedure E*** using 4-methylcinnamic acid (1.5 equiv.), *N*-phenyl-2-(*p*-tolylethynyl)aniline **4″e** (1.5 mmol, 1.0 equiv.) and K_2CO_3 (3.0 equiv.) instead of Et_3N in CH_2Cl_2 (3 mL, 0.5 M) and additional DMAP (0.6 equiv.). 69% yield; white solid; ^1H NMR (400 MHz, $CDCl_3$) δ 7.76 (d, *J* = 15.5 Hz, 1H), 7.64 – 7.57 (m, 1H), 7.40 – 7.26 (m, 9H), 7.22 (d, *J* = 8.0 Hz, 2H), 7.18 (d, *J* = 7.4 Hz, 1H), 7.07 (d, *J* = 7.9 Hz, 2H), 7.04 (d, *J* = 7.9 Hz, 2H), 6.45 (d, *J* = 15.5 Hz, 1H), 2.30 (s, 3H), 2.29 (s, 3H).; ^{13}C NMR (100 MHz, $CDCl_3$) δ 166.3, 144.1, 142.7, 142.6, 139.8, 138.6, 133.1, 132.7, 131.5, 129.5, 129.3, 129.1, 129.0, 128.8, 127.9, 127.6, 126.8, 126.2, 123.9, 119.8, 118.9, 85.6, 77.2, 21.4, 21.2.; MS (APCI): *m/z* 428.9 $[M+H]^+$

(*E*)-*N*-ethyl-*N*-(2-(pyridin-2-ylethynyl)phenyl)-3-(*p*-tolyl)acrylamide (4f):

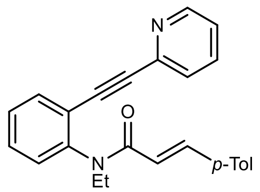

Prepared according to the ***General Procedure E*** using 4-methylcinnamic acid (2.0 equiv.), *N*-ethyl-2-(pyridin-2-ylethynyl)aniline **S6** (0.5 mmol, 1.0 equiv.) and Et_3N (2.0 equiv.) in CH_2Cl_2 (5 mL). 83% yield; brown oil; ^1H NMR (400 MHz, $CDCl_3$) δ 8.57 (ddd, *J* = 4.9, 1.9, 0.9 Hz, 1H), 7.73 (dd, *J* = 7.5, 1.8 Hz, 1H), 7.70 – 7.62 (m, 2H), 7.50 – 7.38 (m, 3H), 7.29 – 7.26 (m, 1H), 7.22 (ddd, *J* = 7.6, 4.9, 1.2 Hz, 1H), 7.19 – 7.14 (m, 2H), 7.05 (d, *J* = 7.9 Hz, 2H), 6.18 (d, *J* = 15.4 Hz, 1H), 4.08 (dq, *J* = 14.3, 7.2 Hz, 1H), 3.87 (dq, *J* = 14.3, 7.2 Hz, 1H), 2.29 (s, 3H), 1.21 (t, *J* = 7.1 Hz, 3H); ^{13}C NMR (100 MHz, $CDCl_3$) δ 166.0, 150.0, 143.7, 142.9, 141.7, 139.7, 136.3, 133.6, 132.4, 130.1, 129.6, 129.3, 128.1, 127.8, 127.8, 123.1, 122.7, 118.0, 93.8, 85.4, 44.2, 21.4, 13.1; MS (APCI): *m/z* 367.2 $[M+H]^+$

(*E*)-*N*-ethyl-*N*-(2-(isoquinolin-4-ylethynyl)phenyl)-3-(*p*-tolyl)acrylamide (4g):

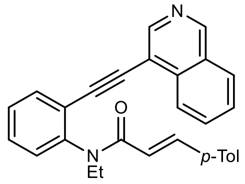

Prepared according to the ***General Procedure E*** using 4-methylcinnamic acid (2.0 equiv.), *N*-ethyl-2-(isoquinolin-4-ylethynyl)aniline **S7** (0.5 mmol, 1.0 equiv.) and Et_3N (2.0 equiv.) in CH_2Cl_2 (5 mL). 78% yield; brown oil; ^1H NMR (400 MHz, $CDCl_3$) δ 9.17 (s, 1H), 8.71 (s, 1H), 8.21 (dd, *J* = 8.4, 1.0 Hz, 1H), 7.96 (dt, *J* = 8.1, 1.0 Hz,

1H), 7.82 – 7.60 (m, 4H), 7.54 – 7.42 (m, 2H), 7.35 – 7.29 (m, 1H), 7.19 (d, *J* = 8.1 Hz, 2H), 7.05 (d, *J* = 7.9 Hz, 2H), 6.26 (d, *J* = 15.5 Hz, 1H), 4.15 (dq, *J* = 14.4, 7.2 Hz, 1H), 3.91 (dq, *J* = 14.2, 7.2 Hz, 1H), 2.28 (s, 3H), 1.23 (t, *J* = 7.2 Hz, 3H); ^{13}C NMR (100 MHz, $CDCl_3$) δ 165.8, 152.3, 146.7, 143.2, 142.0, 139.7, 135.4, 133.5, 132.5, 131.4, 129.9, 129.8, 129.3, 128.1, 128.0, 127.80, 127.79, 125.1, 123.4, 118.0, 92.8, 89.9, 44.1, 21.3, 13.2; MS (APCI): *m/z* 417.3 $[M+H]^+$

(*E*)-*N*-(2-(hex-1-yn-1-yl)phenyl)-3-(*p*-tolyl)acrylamide (4′h)

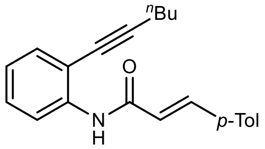

Prepared according to the ***General Procedure E*** using 4-methylcinnamic acid (1.5 equiv.), and 2-(hex-1-yn-1-yl)aniline **S8** (0.4 mmol, 1.0 equiv.) and additional DMAP (0.2 equiv.) in CH_2Cl_2 (2 mL, 0.2 M). 50% yield; white solid; ^{1}H NMR (400 MHz, $CDCl_3$) δ 8.52 (d, *J* = 8.4 Hz, 1H), 8.12 (s, 1H), 7.73 (d, *J* = 15.5 Hz, 1H), 7.46 (d, *J* = 8.1 Hz, 2H), 7.38 (dd, *J* = 7.7, 1.5 Hz, 1H), 7.32 (ddd, *J* = 8.7, 7.6, 1.6 Hz, 1H), 7.21 (d, *J* = 7.9 Hz, 2H), 7.02 (td, *J* = 7.6, 1.1 Hz, 1H), 6.49 (d, *J* = 15.5 Hz, 1H), 2.56 (t, *J* = 7.0 Hz, 2H), 2.39 (s, 3H), 1.74 – 1.64 (m, 2H), 1.63 – 1.54 (m, 2H), 1.00 (t, *J* = 7.3 Hz, 3H); ^{13}C NMR (100 MHz, $CDCl_3$) δ 163.9, 142.3, 140.4, 139.0, 131.8, 131.5, 129.6, 128.9, 127.9, 123.2, 119.9, 119.2, 112.7, 98.0, 76.1, 30.8, 22.2, 21.4, 19.3, 13.7; MS (APCI): *m/z* 318.5 $[M+H]^+$

(*E*)-*N*-ethyl-*N*-(2-(hex-1-yn-1-yl)phenyl)-3-(*p*-tolyl)acrylamide (4h)

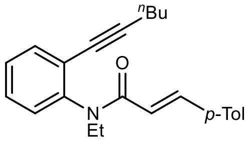

Prepared according to the ***General Procedure G*** using (*E*)-*N*-(2-(hex-1-yn-1-yl)phenyl)-3-(*p*-tolyl)acrylamide **4′h** (0.19 mmol, 1.0 equiv.). 99% yield; ^{1}H NMR (400 MHz, $CDCl_3$) δ 7.63 (d, *J* = 15.4 Hz, 1H), 7.54 – 7.47 (m, 1H), 7.36 – 7.28 (m, 2H), 7.23 – 7.14 (m, 3H), 7.06 (d, *J* = 7.9 Hz, 2H), 6.13 (d, *J* = 15.6 Hz, 1H), 4.10 – 3.99 (dq, *J* = 14.1, 7.1 Hz, 1H), 3.73 (dq, *J* = 14.1, 7.1 Hz, 1H), 2.34 (t, *J* = 7.0 Hz, 2H), 2.30 (s, 3H), 1.56 – 1.45 (m, 2H), 1.44 – 1.33 (m, 2H), 1.16 (t, *J* = 7.2 Hz, 3H), 0.87 (t, *J* = 7.2 Hz, 3H); ^{13}C NMR (100 MHz, $CDCl_3$) δ 165.8, 143.1, 141.2, 139.4, 133.2, 132.7, 129.6, 129.3, 128.4, 127.8, 127.7, 124.3, 118.3, 96.5, 76.9, 43.7, 30.5, 21.9, 21.3, 19.2, 13.5, 13.0; MS (APCI): *m/z* 346.5 [M+H]+

(*E*)-3-(pyridin-2-yl)acrylic acid (4′i)

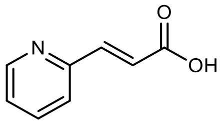

To a solution of pyridine-2-carbaldehyde (1.0 mmol. 1.0 equiv.), malonic acid (1.5 equiv.), and piperidine (10.0 M) in pyridine (1.0 M) was refluxed for 1 h. The reaction mixture was poured into ice and a solution of 1N HCl was added dropwise. The resulting solid was collected by filtration, and washed with water to afford pure product. 61% yield; yellow solid; ^{1}H NMR (400 MHz, DMSO-d$_6$) δ 12.54 (s, 1H), 8.64 (dd, 4.8, 0.9 Hz, 1H), 7.86 (td, 7.7, 1.8 Hz, 1H), 7.72 (d, 7.8 Hz, 1H), 7.59 (d, 15.7 Hz, 1H), 7.40 (ddd, 7.6, 4.8, 1.1 Hz, 1H), 6.82 (d, 15.7 Hz, 1H); The structure was further confirmed by spectral comparison with literature data.[43]

(*E*)-*N*-ethyl-3-(pyridin-2-yl)-*N*-(2-(*p*-tolylethynyl)phenyl)acrylamide (4i)

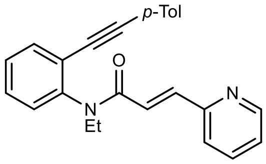

Prepared according to the ***General Procedure F*** using (*E*)-3-(pyridin-2-yl)acrylic acid **4′i** (1.0 equiv.), and *N*-ethyl-2-(*p*-tolylethynyl)aniline **S4** (0.6 mmol, 1.0 equiv.). 58% yield; yellow solid; ^{1}H NMR (400 MHz, $CDCl_3$) δ 8.50 – 8.46 (m, 1H), 7.68 (d, J = 15.2 Hz, 1H), 7.64 – 7.55 (m, 2H), 7.42 – 7.34 (m, 4H), 7.29 – 7.20 (m, 2H), 7.16 – 7.08 (m, 3H), 6.78 (d, J = 15.2 Hz, 1H), 4.14 – 4.03 (m, 1H), 3.93 – 3.81 (m, 1H), 2.34 (s, 3H), 1.20 (t, J = 7.2 Hz, 3H); ^{13}C NMR (100 MHz, $CDCl_3$) δ 165.8, 153.9, 149.9, 143.0, 140.7, 139.0, 136.5, 133.0, 131.6, 129.6, 129.26, 129.25, 128.1, 124.2, 123.9, 123.5, 123.3, 119.7, 95.4, 85.4, 44.4, 21.6, 13.1; MS (APCI): *m/z* 367.2 $[M+H]^+$

(*E*)-3-(furan-2-yl)acrylic acid (4′j)

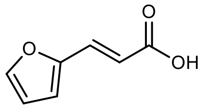

To a solution of 2-furaldehyde (0.5 mmol. 1.0 equiv.), malonic acid (1.5 equiv.), and piperidine (10.0 M) in pyridine (1.0 M) was refluxed for 1 h. The reaction mixture was poured into ice and a solution of 1N HCl was added dropwise. The resulting solid was collected by filtration, and washed with water to afford pure product. 92% yield; brown solid; ^{1}H NMR (400 MHz, DMSO-d$_6$) δ 12.36 (s, 1H), 7.83 (s, 1H), 7.39 (d, J = 15.8 Hz, 1H), 6.92 (d, J = 3.4 Hz, 1H), 6.62 (dd, J = 3.4, 1.8 Hz, 1H), 6.16 (d, J = 15.8 Hz, 1H); The structure was further confirmed by spectral comparison with literature data.[44]

(*E*)-*N*-ethyl-3-(furan-2-yl)-*N*-(2-(*p*-tolylethynyl)phenyl)acrylamide (4j)

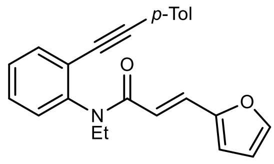

Prepared according to the ***General Procedure E*** using (*E*)-3-(furan-2-yl)acrylic acid **4′j** (1.2 equiv.), and *N*-ethyl-2-(*p*-tolylethynyl)aniline **S4** (0.25 mmol, 1.0 equiv.), 91% yield; dark oil; ^{1}H NMR (400 MHz, $CDCl_3$) δ 7.65 – 7.61 (m, 1H), 7.44 (d, *J* = 15.2 Hz, 1H), 7.41 – 7.34 (m, 4H), 7.29 – 7.21 (m, 2H), 7.12 (d, *J* = 7.8 Hz, 2H), 6.43 (d, *J* = 3.4 Hz, 1H), 6.34 (dd, *J* = 3.4, 1.8 Hz, 1H), 6.12 (d, *J* = 15.2 Hz, 1H), 4.14 – 4.00 (m, 1H), 3.88 – 3.78 (m, 1H), 2.34 (s, 3H), 1.20 (t, *J* = 7.2 Hz, 3H); ^{13}C NMR (100 MHz, $CDCl_3$) δ 165.8, 151.7, 143.6, 143.0, 138.8, 132.8, 131.5, 129.5, 129.1, 129.0, 128.4, 127.9, 123.9, 119.6, 116.9, 113.5, 111.9, 95.2, 85.2, 44.0, 21.5, 13.0; MS (APCI): *m/z* 356.3 $[M+H]^+$

Synthesis of Methyl (*Z*)-4-(ethyl(2-(*p*-tolylethynyl)phenyl)amino)-4-oxobut-2-enoate (4k)

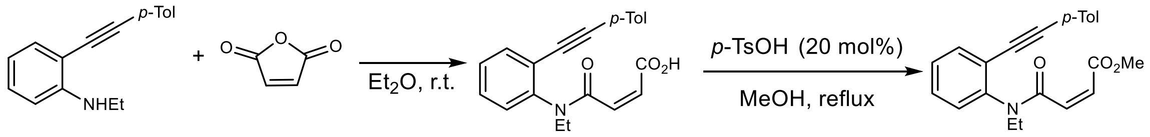

S4 **4'k** **4k**

(*Z*)-4-(ethyl(2-(*p*-tolylethynyl)phenyl)amino)-4-oxobut-2-enoic acid (4′k)

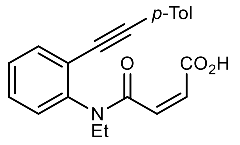

To a solution of maleic anhydride (1.0 equiv.) in Et_2O was added a solution of *N*-ethyl-2-(*p*-tolylethynyl)aniline **S4** (0.2 mmol, 1.0 equiv, 0.8 M) in Et_2O. The reaction mixture was stirred at room temperature for 6h. The solvent was evaporated under reduced pressure. The crude material was purified by flash chromatography on silica gel. 73% yield; white solid; ^{1}H NMR (400 MHz, $CDCl_3$) δ 7.65 (dd, *J* = 5.9, 3.4 Hz, 1H), 7.45 (dd, *J* = 5.8, 3.4 Hz, 2H), 7.34 (d, *J* = 8.1 Hz, 2H), 7.25 (dd, *J* = 5.7, 3.6 Hz, 1H), 7.17 (d, *J* = 7.9 Hz, 2H), 6.18 (d, *J* = 13.0 Hz, 1H), 6.11 (d, *J* = 13.2 Hz, 1H), 3.94 (q, *J* = 7.2 Hz, 1H), 2.37 (s, 3H), 1.25 (t, *J* = 7.2 Hz, 3H); ^{13}C NMR (100 MHz, $CDCl_3$) δ 166.3, 164.9, 140.9, 139.7, 136.2, 133.2, 131.4, 129.6, 129.4, 129.3, 128.5, 128.1, 123.4, 118.6, 96.6, 83.8, 45.7, 21.6, 12.3; MS (APCI): *m/z* 334.2 $[M+H]^+$

Methyl (*Z*)-4-(ethyl(2-(*p*-tolylethynyl)phenyl)amino)-4-oxobut-2-enoate (4k)

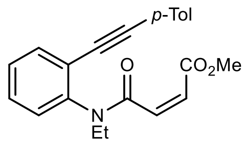

To a solution of (*Z*)-4-(ethyl(2-(*p*-tolylethynyl)phenyl)amino)-4-oxobut-2-enoic acid **4′k** (0.2 mmol, 1.0 equiv.), and *p*-toluenesulfonic acid monohydrate (0.2 equiv.) in MeOH (0.1 M) was refluxed for 3h. The solvent was evaporated under reduced pressure. The solvent was evaporated under reduced pressure. The crude material was purified by flash chromatography on silica gel. 99% yield; white solid; ^{1}H NMR (400 MHz, $CDCl_3$) δ 7.59 – 7.52 (m, 1H), 7.41 (d, *J* = 8.1 Hz, 2H), 7.37 – 7.24 (m, 3H), 7.15 (d, *J* = 7.9 Hz, 2H), 6.39 (d, *J* = 11.9 Hz, 1H), 5.72 (d, *J* = 11.9 Hz, 1H), 4.03 – 3.84 (m, 2H), 3.74 (s, 3H), 2.37 (s, 3H), 1.23 (t, *J* = 7.2 Hz, 3H); ^{13}C NMR (100 MHz, $CDCl_3$) δ 165.7, 165.6, 142.4, 139.1, 137.2, 132.6, 131.6, 129.2, 128.99, 128.96, 128.2, 123.7, 123.6, 119.3, 95.2, 84.9, 51.8, 43.5, 21.5, 12.8; MS (APCI): *m/z* 348.3 $[M+H]^+$

N^1-ethyl-*N*^{4},*N*^{4}-diisopropyl-*N*^{1}-(2-(*p*-tolylethynyl)phenyl)maleamide (4l)

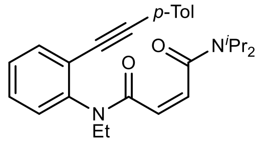

Prepared according to the ***General Procedure F*** using (*Z*)-4-(ethyl(2-(*p*-tolylethynyl)phenyl)amino)-4-oxobut-2-enoic acid **4′k** (0.2 mmol, 1.0 equiv.), and DIPA (1.2 equiv.). 78% yield; white solid; ^{1}H NMR (400 MHz, $CDCl_3$) δ 7.60 – 7.56 (m, 1H), 7.38 (d, *J* = 8.0 Hz, 2H), 7.36 – 7.32 (m, 3H), 7.14 (d, *J* = 7.8 Hz, 2H), 6.09 (d, *J* = 11.9 Hz, 1H), 5.75 (d, *J* = 11.9 Hz, 1H), 4.14 – 3.98 (m, 1H), 3.97 – 3.85 (m, 1H), 3.83 – 3.69 (m, 1H), 3.56 – 3.44 (m, 1H), 2.36 (s, 3H), 1.52 (d, *J* = 6.8 Hz, 3H), 1.45 (d, *J* = 6.9 Hz, 3H), 1.18 – 1.12 (m, 6H), 1.09 (d, *J* = 6.7 Hz, 3H); ^{13}C NMR (100 MHz, $CDCl_3$) δ 166.9, 164.7, 142.8, 139.0, 134.4, 132.8, 131.6, 130.3, 129.1, 129.1, 127.9, 124.9, 123.1, 119.5, 95.1, 85.2, 50.1, 45.4, 43.3, 21.6, 20.8, 20.5, 20.2, 12.9; MS (APCI): *m/z* 417.4 $[M+H]^+$

Synthesis of 3-phenyl-*N*-(2-(*p*-tolylethynyl)phenyl)but-2-enamide (4‴m)

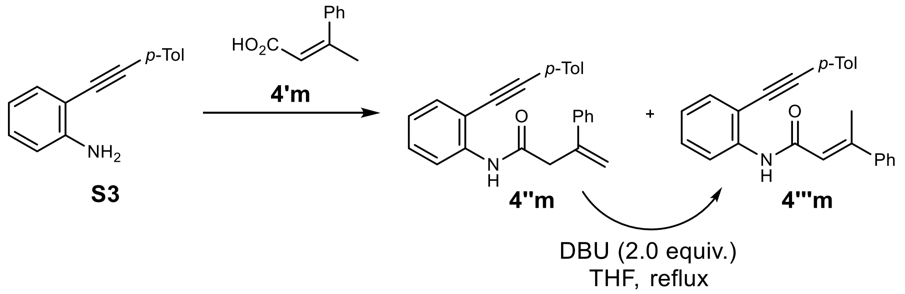

(*Z*)-3-phenylbut-2-enoic acid (4′m)

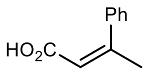

To suspension of CuI (2.0 equiv.) of dry diethyl ether (1.7 mL, 0.3 M) was added dropwise methyllithium solution (3.1 M in diethoxymethane, 4.0 equiv.) at -20°C. The suspension was stirred at -20°C while the solid dissolved. To the solution, 3-phenylpropynoic acid (0.5 mmol, 1.0 equiv.) was added at -78°C. The reaction mixture was stirred at -20°C for 3 hours. The reaction mixture was poured into 1 N aqueous HCl solution and extracted with CH_2Cl_2. The combined organic layer was dried over Na_2SO_4, filtered and concentrated. The crude material was purified by flash chromatography. 92%; white solid; ^{1}H NMR (400 MHz, $CDCl_3$) δ 7.36 – 7.29 (m, 3H), 7.22 – 7.18 (m, 2 H), 5.90 (d, J = 1.4 Hz, 1H), 2.19 (d, J = 1.4 Hz, 3H); The structure was further confirmed by spectral comparison with literature data.[45]

3-phenyl-*N*-(2-(*p*-tolylethynyl)phenyl)but-3-enamide (4″m)

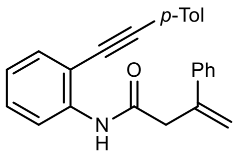

Prepared according to the ***General Procedure E*** using (*Z*)-3-phenylbut-2-enoic acid **4′m** (1.5 equiv.), and 2-(*p*-tolylethynyl)aniline **S3** (0.1 mmol, 1.0 equiv.) in CH_2Cl_2 (0.2 mL, 0.5 M) and additional DMAP (0.2 equiv.). 75% yield; ^{1}H NMR (400 MHz, $CDCl_3$) δ 8.63 (s, 1H), 8.46 (d, J = 8.3 Hz, 1H), 7.45 – 7.38 (m, 3H), 7.35 – 7.24 (m, 5H), 7.21 – 7.12 (m, 2H), 7.03 (td, J = 7.6, 1.2 Hz, 1H), 5.58 (d, J = 0.9 Hz, 1H), 5.37 (d, J = 0.9 Hz, 1H), 3.67 (d, J = 0.9 Hz, 1H), 2.41 (s, 3H). ^{13}C NMR (100 MHz, $CDCl_3$) δ 168.4, 141.9, 139.1, 138.7, 138.5, 131.6, 131.4, 129.5, 129.2, 128.6, 128.3, 125.7, 123.4, 119.3, 118.9, 117.9, 112.1, 96.3, 83.3, 45.8, 21.6; MS (APCI): *m/z* 352.8 $[M+H]^+$

(*E*)-3-phenyl-*N*-(2-(*p*-tolylethynyl)phenyl)but-2-enamide (4‴m)

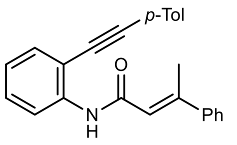

To a solution of 3-phenyl-*N*-(2-(*p*-tolylethynyl)phenyl)but-3-enamide **4″m** (0.11 mmol, 1.0 equiv.) in THF (0.28 mL, 0.4 M) was added DBU (2.0 equiv.). The reaction mixture was refluxed for 2 hours. The reaction mixture was washed with a saturated solution of NH_4Cl and extracted with EtOAc. The combined organic layer was dried over Na_2SO_4, filtered and concentrated. The crude material was purified by flash chromatography on silica gel. 63% yield; yellow solid; ^{1}H NMR (400 MHz, $CDCl_3$) δ 8.54 (d, J = 8.4 Hz, 1H), 8.11 (s, 1H), 7.53 – 7.44 (m, 3H), 7.44 – 7.33 (m, 5H), 7.17 (d, J = 7.8 Hz, 2H), 7.07 (t, J = 7.6 Hz, 1H), 6.19 (s, 1H), 2.65 (s, 3H), 2.38 (s,

3H); ^{13}C NMR (100 MHz, $CDCl_3$) δ 164.7, 153.4, 142.6, 139.2, 139.1, 131.7, 131.4, 129.5, 129.3, 128.8, 128.6, 126.3, 123.3, 120.1, 119.3, 119.3, 112.2, 96.8, 83.9, 21.5, 18.1; MS (APCI): *m/z* 352.8 $[M+H]^+$

(*E*)-*N*-ethyl-3-phenyl-*N*-(2-(*p*-tolylethynyl)phenyl)but-2-enamide (4m)

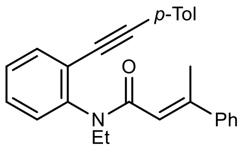

Prepared according to the ***General Procedure G*** using 3-phenyl-*N*-(2-(*p*-tolylethynyl)phenyl)but-2-enamide **4'''m** (0.06 mmol, 1.0 equiv.). 67% yield; ^{1}H NMR (400 MHz, $CDCl_3$) δ 7.59 (dd, *J* = 7.3, 1.9 Hz, 1H), 7.39 (d, *J* = 8.1 Hz, 2H), 7.37 – 7.30 (m, 2H), 7.25 – 7.19 (m, 4H), 7.14 (d, *J* = 7.9 Hz, 2H), 7.11 – 7.08 (m ,2H), 5.85 (d, *J* = 1.2 Hz, 1H), 4.04 – 3.83 (m, 2H), 2.42 (d, *J* = 1.1 Hz, 3H), 2.35 (s, 3H), 1.22 (t, *J* = 7.2 Hz, 3H); ^{13}C NMR (100 MHz, $CDCl_3$) δ 167.2, 148.7, 143.6, 143.0, 138.8, 132.8, 131.6, 129.1, 129.1, 129.0, 128.2, 127.9, 127.7, 126.1, 123.9, 120.3, 119.7, 94.8, 85.4, 43.6, 21.5, 18.1, 13.2; MS (APCI): *m/z* 380.9 $[M+H]^+$

(*E*)-*N*-ethyl-2-(*p*-tolyl)-*N*-(2-(*p*-tolylethynyl)phenyl)ethene-1-sulfonamide (4n)

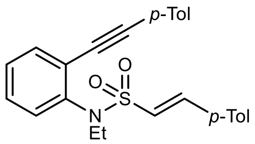

Sulfuryl chloride (2.0 equiv., per styrene) was added dropwise to stirred anhydrous DMF (2.0 M) at 0°C under nitrogen atmosphere. The reaction mixture was heated to room temperature and stirred for 0.5h. 4-Methylstyrene (1.2 equiv.) was added dropwise, and the reaction mixture was stirred for 1h at 80°C. The mixture was filtered through a short pad of silica gel. The filtrate was concentrated under reduced pressure. The resulting sulfonyl chloride was dissolved in CH_2Cl_2 and slowly added dropwise to a solution of *N*-ethyl-2-(*p*-tolylethynyl)aniline **S4** (0.2 mmol, 1.0 equiv.) and pyridine (1.5 equiv.) in CH_2Cl_2 (0.33 M) at 0°C. The reaction mixture was stirred at 0°C for overnight. The solvent was evaporated under reduced pressure. The crude material was purified by flash chromatography on silica gel. 73% yield; yellow solid; ^{1}H NMR (400 MHz, $CDCl_3$) δ 7.55 (dd, *J* = 7.2, 2.0 Hz, 1H), 7.45 (dd, *J* = 7.7, 1.5 Hz, 1H), 7.39 – 7.30 (m, 3H), 7.26 – 7.24 (m, 2H), 7.15 (d, *J* = 8.1 Hz, 2H), 7.02 – 6.96 (m, 4H), 6.85 (d, *J* = 15.4 Hz, 1H), 3.86 (q, *J* = 7.1 Hz, 2H), 2.33 (s, 3H), 2.31 (s, 3H), 1.20 (t, *J* = 7.1 Hz, 3H); ^{13}C NMR (100 MHz, $CDCl_3$) δ 140.8, 140.6, 139.5, 138.8, 133.3, 133.1, 131.3, 130.1, 129.4, 129.1, 128.9, 128.3, 128.2, 124.4, 123.9, 119.4, 95.0, 86.2, 45.4, 21.5, 21.4, 14.9; MS (APCI): *m/z* 416.3 $[M+H]^+$

1-((trimethylsilyl)ethynyl)cyclohexyl (*E*)-3-(*p*-tolyl)acrylate (4o)

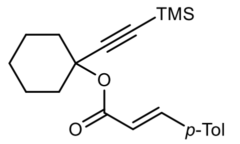

To a suspension of 4-methylcinnamic acid (1.2 equiv.) and the 1-((trimethylsilyl)ethynyl)cyclohexan-1-ol **1p** (0.3 mmol, 1.0 equiv.) in dry CH_2Cl_2 (0.2 M) was added 4-(dimethylamino)pyridine (0.6 equiv.). The solution was cooled to 0 °C and *N,N'*-dicyclohexyl carbodiimide (1.5 equiv.) was added. The reaction mixture was refluxed 24 hours. The precipitated *N,N*-dicyclohexylurea was separated by filtration, and the filtrate was concentrated. The crude material was purified by flash chromatography. 65% yield; white solid; ^{1}H NMR (400 MHz, $CDCl_3$) δ 7.62 (d, *J* = 16.0 Hz, 1H), 7.41 (d, *J* = 7.8 Hz, 2H), 7.18 (d, *J* = 7.7 Hz, 2H), 6.36 (d, *J* = 16.0 Hz, 1H), 2.37 (s, 3H), 2.24 – 2.10 (m, 2H), 2.00 – 1.86 (m, 2H), 1.72 – 1.47 (m, 5H), 1.34 (dp, *J* = 14.0, 7.3, 6.9 Hz, 1H), 0.17 (s, 9H); ^{13}C NMR (100 MHz, $CDCl_3$) δ 165.0, 144.2, 140.4, 131.8, 129.5, 128.0, 118.2, 105.4, 90.8, 75.7, 37.2, 25.2, 22.7, 21.4, -0.0; MS (APCI): *m/z* 341.8 $[M+H]^+$

Synthesis of (*E*)-*N*-phenyl-3-(*p*-tolyl)-*N*-(1-((trimethylsilyl)ethynyl)cyclohexyl)acrylamide (4p)

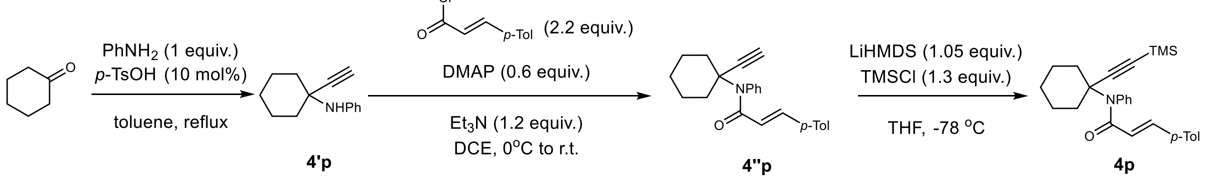

N-(1-ethynylcyclohexyl)aniline (4′p)

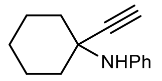

A solution of aniline (0.5 mmol, 1.0 equiv.) and cyclohexanone (1.5 equiv.) in dry toluene (0.5 mL, 1 M) was added *p*-toluenesulfonic acid (0.1 equiv.). The reaction mixture was stirred at 110°C for 24 hours. The mixture was taken up in hexane and filtered through a pad of Celite®. The solvent was then removed under vacuum to obtain *N*-phenyl cyclohexanimine, which was used in the next step without purification. To mixture of *N*-phenyl cyclohexanimine (1.0 equiv.) and Ethynyltrimethylsilane (1.5 equiv.) in DMSO (1.7 mL, 0.3 M) was added potassium tert-butoxide (0.5 equiv.). The reaction mixture was stirred at 40°C to 20 min. The mixture was washed with saturated aqueous Na_2CO_3 and extracted with EtOAc. The combined organic layer was dried over Na_2SO_4, filtered and concentrated. The crude material was purified by flash chromatography. 45% yield; ^{1}H NMR (400 MHz, $CDCl_3$) δ 7.18 (dd, *J* = 8.6, 7.3 Hz, 2H), 6.95 (dd, *J* = 8.6, 1.1 Hz, 2H), 6.77 (tt, *J* = 7.4, 1.1 Hz, 1H), 3.64 (s, 1H), 2.45 (s, 1H), 2.19 (d, *J* = 12.1 Hz, 2H), 1.77 – 1.49 (m, 7H), 1.35 – 1.21 (m, 1H); The structure was further confirmed by spectral comparison with literature data.[46]

(*E*)-*N*-(1-ethynylcyclohexyl)-*N*-phenyl-3-(*p*-tolyl)acrylamide (4″p)

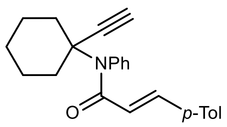

To a suspension of 4-methylcinnamic acid (2.2 equiv.), DMF (3 mol%) in dry CH_2Cl_2 (0.5 M) was added dropwise oxalyl chloride (1.1 equiv., per acid) at 0°C. The reaction mixture was stirred at room temperature for 1h. Then, the solvent was removed under reduced pressure. The residue was dissolved in dry DCE and slowly added dropwise to a solution of *N*-(1-ethynylcyclohexyl) aniline **4′p** (0.21 mmol, 1.0 equiv.), Et_3N (1.2 equiv.) and DMAP (0.6 equiv.) at 0°C. The mixture was stirred at room temperature until TLC indicated complete consumption of the starting material. The reaction mixture was diluted with water and extracted with EtOAc. The combined organic layer was dried over Na_2SO_4, filtered and concentrated. The crude material was purified by flash chromatography on silica gel. 83% yield; yellow solid; ^{1}H NMR (400 MHz, $CDCl_3$) δ 7.60 (d, *J* = 15.5 Hz, 1H), 7.45 – 7.37 (m, 3H), 7.29 – 7.21 (m, 2H), 7.10 (d, *J* = 8.1 Hz, 2H), 7.03 (d, *J* = 8.0 Hz, 2H), 5.96 (d, *J* = 15.5 Hz, 1H), 2.56 (s, 1H), 2.43 – 2.30 (m, 2H), 2.29 (s, 3H), 1.77 (q, *J* = 13.3 Hz, 2H), 1.66 – 1.61 (m, 3 H), 1.56 – 1.44 (m, 2 H), 1.16 – 1.02 (m, 1H); ^{13}C NMR (100 MHz, $CDCl_3$) δ 166.3, 141.3, 140.1, 139.5, 132.5, 131.0, 129.3, 129.1, 128.5, 127.7, 120.1, 85.1, 73.4, 59.0, 36.9, 25.3, 23.3, 21.3; MS (APCI): *m/z* 344.1 $[M+H]^+$

(*E*)-*N*-phenyl-3-(*p*-tolyl)-*N*-(1-((trimethylsilyl)ethynyl)cyclohexyl)acrylamide (4p)

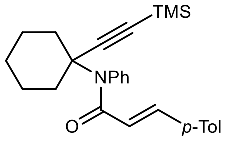

To a solution of ((*E*)-*N*-(1-ethynylcyclohexyl)-*N*-phenyl-3-(*p*-tolyl)acrylamide **4″p** (0.15 mmol, 1.0 equiv.) in THF (0.2 mL, 0.8 M) was add lithium bis(trimethylsilyl)amide solution (1 M in THF, 1.05.0 equiv.) at -78°C under nitrogen. The reaction mixture was stirred at -78°C for 1 hour. then Chlorotrimethylsilane (1.3 equiv.) was added dropwise, and the solution stirred for 3 hours at -78°C. The reaction mixture was washed with a saturated solution of NH_4Cl and extracted with CH_2Cl_2. The combined organic layer was dried over Na_2SO_4, filtered and concentrated. The crude material was purified by flash chromatography on silica gel. 83% yield; white solid; ^{1}H NMR (400 MHz, $CDCl_3$) δ 7.53 (d, *J* = 15.5 Hz, 1H), 7.42 – 7.35 (m, 3H), 7.25 – 7.18 (m, 2H), 7.10 (d, *J* = 8.1 Hz, 2H), 7.04 (d, *J* = 8.0 Hz, 2H), 6.01 (d, *J* = 15.4 Hz, 1H), 2.29 (s, 3H), 2.24 – 2.09 (m, 4H), 1.82 – 1.52 (m, 5H), 1.27 – 1.10 (m, 1H), 0.06 (s, 9H). ^{13}C NMR (100 MHz, $CDCl_3$) δ 166.2, 141.4, 140.7, 139.6, 132.8, 131.1, 129.4, 128.9, 128.3, 127.8, 121.0, 106.8, 92.3, 62.0, 36.0, 25.3, 23.9, 21.5, 0.1; MS (APCI): *m/z* 416.3 $[M+H]^+$

Synthesis of (*E*)-*N*-ethyl-3-(*p*-tolyl)-*N*-(3-(*p*-tolyl)prop-2-yn-1-yl)acrylamide (4q)

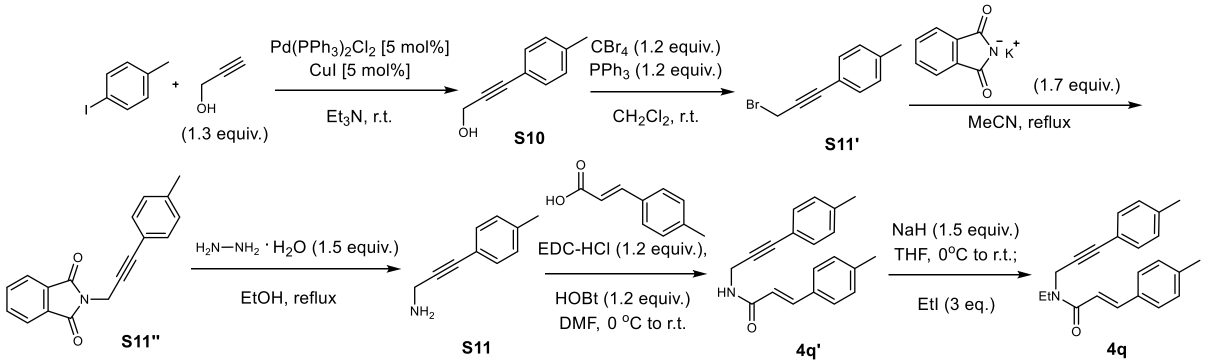

3-(*p*-tolyl)prop-2-yn-1-ol (S10)

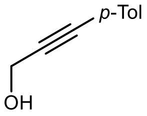

To a suspension of $Pd(PPh_3)_2Cl_2$ (5 mol%), CuI (5 mol%), and 4-iodotoluene (6 mmol, 1.0 equiv.) in degassed Et_3N (30 mL, 0.2 M) was added dropwise the corresponding alkyne (1.3 equiv.) under nitrogen atmosphere. The reaction mixture was stirred for 3 hours at room temperature. The reaction mixture was washed with a saturated solution of NH_4Cl and extracted with CH_2Cl_2. The combined organic layer was dried over Na_2SO_4, filtered and concentrated. The crude material was purified by flash chromatography on silica gel. 99% yield; yellow solid; ^1H NMR (400 MHz, $CDCl_3$) δ 7.33 (d, J = 8.2 Hz, 2H), 7.12 (d, J = 8.2 Hz, 2H), 4.49 (d, J = 6.0 Hz, 2H), 2.35 (s, 3H), 1.71 – 1.63 (m, 1H)); The structure was further confirmed by spectral comparison with literature data.[47]

1-(3-bromoprop-1-yn-1-yl)-4-methylbenzene (S11′)

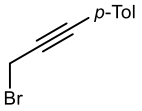

To a solution of 3-(*p*-tolyl)prop-2-yn-1-ol **S10** (0.5 mmol, 1.0 equiv.) and Tetrabromomethane (1.2 equiv.) in CH_2Cl_2 (1.7 mL, 0.3 M) was added triphenylphosphine (1.2 equiv.) at 0°C. The reaction mixture was stirred for 1 hour at 0°C. The solvent was then removed under vacuum. The crude material was purified by flash chromatography on silica gel. 96% yield; colorless oil; ^1H NMR (400 MHz, $CDCl_3$) δ 7.33 (d, J = 8.2 Hz, 2H), 7.12 (d, J = 7.8 Hz, 2H), 4.17 (s, 2H), 2.35 (s, 3H).; The structure was further confirmed by spectral comparison with literature data.[48]

2-(3-(*p*-tolyl)prop-2-yn-1-yl)isoindoline-1,3-dione (S11″)

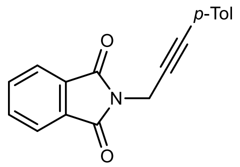

The mixture of 1-(3-bromoprop-1-yn-1-yl)-4-methylbenzene **S11′** (1.0 equiv.), potassium phthalimide (1.7 equiv.) in MeCN (0.2 M) at reflux was stirred overnight. After removed the solvent in vacuum, the residue was purified by flash column chromatography on silica gel. 89% yield; white solid; ^{1}H NMR (400 MHz, $CDCl_3$) δ 7.91 – 7.89 (dd, 2H), 7.75 – 7.73 (dd, 2H), 7.32 – 7.30 (d, 2H), 7.08 – 7.06 (d, 2H) 4.67 (s, 2H), 2.32 (s, 3H); The structure was further confirmed by spectral comparison with literature data.[49]

3-(*p*-tolyl)prop-2-yn-1-amine (S11)

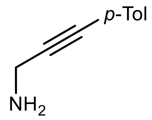

The mixture of 2-(3-(*p*-tolyl)prop-2-yn-1-yl)isoindoline-1,3-dione **S11″** (1.0 equiv.), hydrazine monohydrate (1.5 equiv.) and EtOH (0.25 M) was heated to reflux for overnight. Upon completion, the solution was further diluted in Et_2O and filtered to removed undesired insoluble byproducts. The crude reaction mixture was then concentrated and purified via flash column chromatography on silica gel. 89% yield; yellow oil; ^{1}H NMR (400 MHz, $CDCl_3$) δ 7.31 – 7.29 (d, 2H), 7.11 – 7.09 (d, 2H), 3.65 (br, 2H), 2.34 (s, 3H); The structure was further confirmed by spectral comparison with literature data.[49]

(*E*)-3-(*p*-tolyl)-*N*-(3-(*p*-tolyl)prop-2-yn-1-yl)acrylamide (4q′)

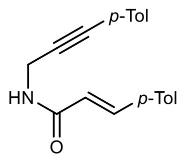

To a solution of 4-methylcinnamic acid (1.0 equiv.) in DMF (0.25 M) at 0 °C was added 3-(*p*-tolyl)prop-2-yn-1-amine **S11** (1.0 equiv.), and EDC-HCl (1.2 equiv.), HOBt (1.2 equiv.). The cooling bath was removed and the reaction stirred at room temperature. A saturated solution of $NaHCO_3$ was added and the crude product extracted with Et_2O. The combined organic layers were dried over Na_2SO_4 concentrated under reduced pressure and the product purified by flash chromatography on silica gel. 91% yield; white solid; ^{1}H NMR (400 MHz, $CDCl_3$) δ 7.66 – 7.62 (d, J = 15.6 Hz, 1H), 7.42 – 7.40 (d, J = 8.1 Hz, 2H), 7.33 – 7.31 (d, J = 8.2 Hz, 2H), 7.19 – 7.17 (d, J = 7.9 Hz, 2H), 7.13 – 7.10 (d, J = 8.4 Hz, 2H), 6.39 – 6.35(d, J = 15.6 Hz, 1H), 5.78 (br, 1H), 4.41 – 4.40 (d,

2H), 2.37 (s, 3H), 2.35 (s, 3H); ^{13}C NMR (100 MHz, $CDCl_3$) δ 165.6, 141.7, 140.1, 138.6, 131.9, 131.6, 129.5, 129.1, 127.8, 119.4, 118.9, 84.0, 83.7, 30.3, 21.5, 21.4; MS (APCI): *m/z* 290.8 $[M+H]^+$

(*E*)-*N*-ethyl-3-(*p*-tolyl)-*N*-(3-(*p*-tolyl)prop-2-yn-1-yl)acrylamide (4q)

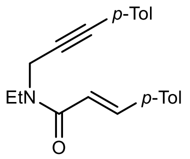

To a slurry of NaH (1.5 equiv.) in THF at 0 °C was added (*E*)-3-(*p*-tolyl)-*N*-(3-(*p*-tolyl)prop-2-yn-1-yl)acrylamide **4q′** (1.0 equiv.) dissolved in THF (0.2 M) dropwise. The reaction mixture was stirred at room temperature for 1 h, the iodoethane (3.0 equiv.) was added dropwise. The reaction mixture was monitored by TLC and quenched with a saturated solution of NH_4Cl upon completion then the solvent was removed under reduced pressure and ethyl acetate was added. The organic phase was washed with distilled water and dried with Na_2SO_4. The solvent was removed under reduced pressure. The crude product was purified by flash chromatography on silica gel. 40% yield; orange liquid; ^{1}H NMR (400 MHz, DMSO-d_6, 90 °C) δ 7.55 (d, *J* = 8.0 Hz, 2H), 7.50 (d, *J* = 15.4 Hz, 1H), 7.28 (d, *J* = 8.0 Hz, 2H), 7.20 (d, *J* = 7.9 Hz, 2H), 7.15 (d, *J* = 7.9 Hz, 2H), 7.06 (d, *J* = 15.3 Hz, 1H), 4.51 (s, 2H), 3.76 – 3.51 (m, 2H), 2.32 (s, 3H), 2.29 (s, 3H), 1.22 (t, *J* = 7.0 Hz, 3H); ^{13}C NMR (100 MHz, $CDCl_3$, 45 °C) δ 166.2, 142.9, 139.7, 138.3, 132.7, 131.6, 129.7, 129.4, 128.9, 127.7, 116.5, 83.9, 83.8, 41.8, 29.6, 21.3, 21.2; MS (APCI): *m/z* 318.9 $[M+H]^+$

Di-*tert*-butyl((3-(*p*-tolyl)prop-2-yn-1-yl)oxy)silyl (*E*)-3-(*p*-tolyl)acrylate (4r)

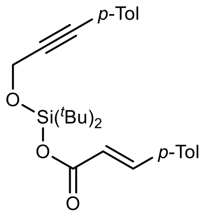

To a solution of $(^tBu)_2SiCl_2$ (0.5 mmol, 1.0 equiv.) in CH_2Cl_2 (2.5 mL, 0.2 M) was sequentially added 3-(*p*-tolyl)prop-2-yn-1-ol **S10** (1.0 equiv.), Et_3N (3.0 equiv.), imidazole (2.0 equiv.) and HOBt (0.5 equiv.) at 0 ºC. The solution was stirred at room temperature for 2 h. When TLC indicated the reaction was complete, 4-methylcinnamic acid (1.0 equiv.) was added at the same temperature. The solution was stirred for overnight. the reaction mixture was diluted in EtOAc and washed with brine. The organic layer was concentrated *in vacuo*. The resulting crude product was purified by flash column chromatography on silica gel to afford desired product. 77% yield; colorless oil; ^{1}H NMR (400 MHz, $CDCl_3$): δ 7.68 (d, *J* = 15.8 Hz, 1H), 7.38 (d, *J* = 8.2 Hz, 2H), 7.31 (d, *J* = 8.1 Hz, 2H), 7.16 (d, *J* = 8.0 Hz, 2H), 7.07 (d, *J* = 7.9 Hz, 2H), 6.41 (d, *J* = 15.9 Hz, 1H), 4.87 (s, 2H), 2.37 (s,

3H), 2.32 (s, 3H), 1.14 (s, 18H); ^{13}C NMR (100 MHz, $CDCl_3$) δ 165.5, 145.9, 140.9, 138.4, 131.73, 131.66, 129.7, 129.1, 128.4, 120.1, 118.6, 87.1, 85.1, 54.0, 27.5, 21.63, 21.58, 21.4; MS (APCI): *m/z* 449.7 $[M+H]^+$

Benzofuran-2-carboxylic acid (4′s)

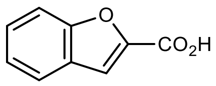

Benzofuran-2-carbaldehyde (0.3 mmol, 1.0 equiv.), NaH_2PO_4 (3.0 equiv.), and 2-methyl-2-butene (9.0 equiv.) were dissolved in acetone (0.2 M) and cooled to 0°C. A solution of sodium chloride (5.5 equiv.) in water (1 mL/mmol) was added dropwise, and the mixture was stirred at room temperature for 1h. The volatile components were evaporated under reduced pressure and the residue was added with 1N HCl. The resulting solid was collected by filtration, and washed with water to afford pure product. 88% yield; white solid; ^{1}H NMR (400 MHz, $CDCl_3$) δ 7.75 – 7.68 (m, 2H), 7.63 (d, 1H, J = 8.8 Hz), 7.50 (ddd, 1H, J = 8.5, 7.3, 1.2 Hz), 7.37 – 7.31 (m, 1H); The structure was further confirmed by spectral comparison with literature data.[50]

***N*-ethyl-*N*-(2-(*p*-tolylethynyl)phenyl)benzofuran-2-carboxamide (4s)**

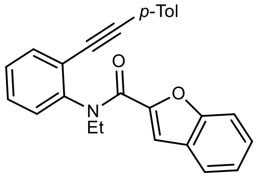

Prepared according to the ***General Procedure E*** using benzofuran-2-carboxylic acid **4′s** (1.2 equiv.), and *N*-ethyl-2-(*p*-tolylethynyl)aniline **S4** (0.2 mmol, 1.0 equiv.), 74% yield; white solid; ^{1}H NMR (400 MHz, $CDCl_3$) δ 7.58 – 7.65 (m, 1H), 7.44 – 7.35 (m, 5H), 7.35 – 7.30 (m, 2H), 7.29 – 7.22 (m, 1H), 6.32 (s, 1H), 4.18 – 3.92 (m, 2H), 2.35 (s, 3H), 1.29 (t, J = 7.2 Hz, 3H); ^{13}C NMR (100 MHz, $CDCl_3$) δ 160.0, 154.4, 143.6, 138.9, 132.8, 131.6, 129.1, 129.0, 129.0, 128.0, 127.1, 126.2, 123.8, 123.1, 122.2, 119.5, 111.9, 111.3, 95.2, 84.9, 45.4, 21.5, 12.7; MS (APCI): *m/z* 380.4 $[M+H]^+$

Synthesis of Benzo[b]thiophene-2-carboxylic acid (4″t)

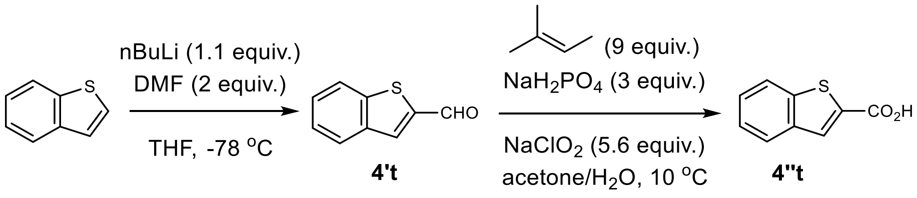

Benzo[b]thiophene-2-carbaldehyde (4′t)

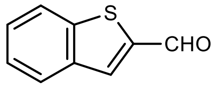

To a solution of benzothiophene (0.9 mmol, 1.0 equiv.) in anhydrous THF (8 mL) cooled at -78 °C was added dropwise *n*-butyllithium (2.5 M in hexane, 0.99 mmol, 1.1 equiv.). After 1 hour of stirring at -78 °C, DMF (1.8 mmol, 2.0 equiv.) was added dropwise. After 2h 30 min at -78 °C, the reaction mixture was quenched by addition of saturated aqueous NH_4Cl solution. The aqueous phase was extracted with EtOAc (3 x 10 mL). The organic layers were combined, dried over anhydrous Na_2SO_4, filtered and concentrated under reduced pressure. The crude product was purified by flash chromatography. 85% yield; colorless oil; ^{1}H NMR (400 MHz, Chloroform-d) = δ 10.11 (s, 1H), 8.03 (s, 1H), 7.97 – 7.86 (m, 2H), 7.47 (dddd, *J* = 26.7, 8.1, 7.1, 1.1 Hz, 2H). The structure was further confirmed by spectral comparison with literature data.[51]

Benzo[b]thiophene-2-carboxylic acid (4″t)

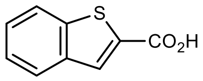

The aldehyde **4′t** (0.61 mmol, 1.0 equiv.), NaH_2PO_4 (1.83 mmol, 3.0 equiv.) and 2-methyl-2-butene (5.49 mmol, 9.0 equiv.) were dissolved in acetone (4 mL) and cooled to 10 °C. A solution of $NaClO_2$ (3.39 mmol, 5.55.0 equiv.) in water (1 M) was slowly added dropwise. After the reaction was stopped (TLC control), the volatile components were removed *in vacuo* and the residue was acidified with 1 N HCl (2 mL/mmol) to obtain white precipitate, which was collected by filtration and dried. The crude product was purified by recrystallization from (EtOH/Water = 1/1). 65% yield; white crystalline solid, ^{1}H NMR (400 MHz, DMSO-d$_6$) δ 13.25 (s, 1H), 8.09 (s, 1H), 8.06 – 7.96 (m, 2H), 7.54 – 7.39 (m, 2H). The structure was further confirmed by spectral comparison with literature data.[51]

***N*-ethyl-*N*-(2-(*p*-tolylethynyl)phenyl)benzo[b]thiophene-2-carboxamide (4t)**

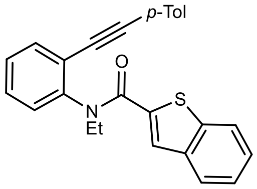

Prepared according to the ***General Procedure E*** using benzo[*b*]thiophene-2-carboxylic acid **4″t** (2.0 equiv.), *N*-ethyl-2-(*p*-tolylethynyl)aniline **S4** (0.49 mmol, 1.0 equiv.) and Et_3N (2.0 equiv.) in CH_2Cl_2 (5 mL). 88% yield; yellow solid; ^{1}H NMR (400 MHz, $CDCl_3$) δ 7.66 (d, *J* = 7.8 Hz, 1H), 7.56 (ddd, *J* = 8.1, 6.5, 2.0 Hz, 2H), 7.44 – 7.34 (m, 5H), 7.30 – 7.20 (m, 2H), 7.15 (d, *J* = 7.9 Hz, 2H), 7.06 (s, 1H), 4.05 (dtd, *J* = 27.4, 13.8, 7.0 Hz, 2H),

2.36 (s, 3H), 1.30 (t, J = 7.2 Hz, 3H); ^{13}C NMR (100 MHz, $CDCl_3$) δ 163.2, 143.6, 141.3, 139.0, 138.7, 138.5, 132.9, 131.6, 129.5, 129.2, 129.1, 128.3, 128.1, 125.7, 124.9, 124.4, 124.2, 122.1, 119.5, 95.4, 85.1, 46.0, 21.6, 12.7; MS (APCI): *m/z* 396.2 $[M+H]^+$

***N*-ethyl-*N*-(2-(naphthalen-2-ylethynyl)phenyl)benzo[b]thiophene-2-carboxamide (4u)**

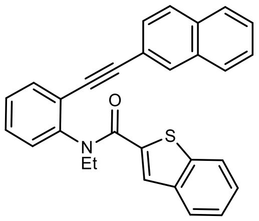

Prepared according to the ***General Procedure E*** using benzo[*b*]thiophene-2-carboxylic acid **4″t** (1.5 equiv.), and *N*-ethyl-2-(naphthalen-2-ylethynyl)aniline **S9** (0.22 mmol, 1.0 equiv.) in DCE (1.1 mL, 0.2 M) and additional DMAP (0.2 equiv.). 65% yield; white solid; ^{1}H NMR (400 MHz, $CDCl_3$) δ 8.05 (s, 1H), 7.89 – 7.75 (m, 3H), 7.67 (d, J = 7.8 Hz, 1H), 7.64 – 7.54 (m, 3H), 7.53 – 7.46 (m, 2H), 7.46 – 7.35 (m, 3H), 7.25 (m, 2H), 7.10 (s, 1H), 4.12 (qt, J = 13.0, 7.3 Hz, 1H), 1.35 (t, J = 7.2 Hz, 2H); ^{13}C NMR (100 MHz, $CDCl_3$) δ 163.3, 143.8, 141.3, 138.7, 138.5, 133.1, 133.0, 132.9, 131.7, 129.5, 129.4, 128.3, 128.3, 128.2, 128.1, 128.0, 127.8, 126.9, 126.6, 125.8, 124.9, 124.2, 124.2, 122.1, 119.9, 95.6, 86.1, 46.1, 12.7; MS (APCI): *m/z* 432.0 $[M+H]^+$

Synthesis of Extended π-Systems

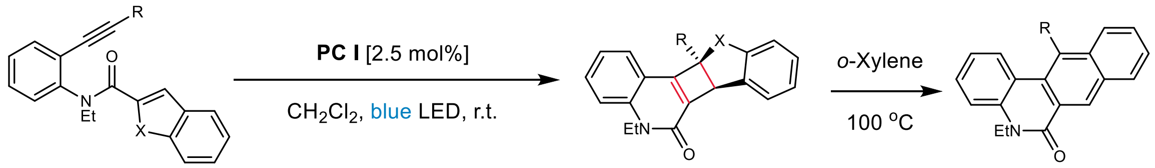

4s X = O, R = *p*-Tol
4t X = S, R = *p*-Tol
4u X = S, R = 2-Naphthalenyl

5s', 5t', 5u'

6t', 6u'

Step 1 : Enyne **4s, 4t** or **4u** (0.1 mmol, 1.0 equiv.), and photocatalyst $Ir[dF(CF_3)ppy]_2(dtbbpy)PF_6$ (2.5 mol%) were added to an oven-dried 4mL vial equipped with a stir bar. The combined materials were dissolved in CH_2Cl_2 (2 mL) under argon atmosphere in glovebox. The reaction mixture was then irradiated by 12 W blue LED lamp at room temperature (maintained with a cooling fan). After completion of the reaction as indicated by TLC, the solution was concentrated under reduced pressure. The residue was purified by flash chromatography on silica gel to give the desired product **5s′, 5t′** or **5u′**.

Step 2 : Solution of **5t′** or **5u′** in *o*-xylene was stirred at 100 °C. After completion of the reaction as indicated by TLC, the solution was concentrated under reduced pressure. The residue was purified by flash chromatography on silica gel to give the desired product **6t′**, **6u′**.

12-ethyl-5-(*p*-tolyl)-5a,12-dihydro-11*H*-benzofuro[2',3':1,4]cyclobuta[1,2-*c*]quinolin-11-one (5s′)

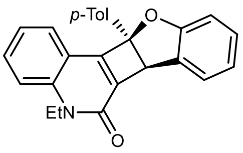

28 mg, 74% yield; white solid; m.p. 152 – 153 °C; ^{1}H NMR (400 MHz, $CDCl_3$) δ 7.58 – 7.49 (m, 3H), 7.43 (dd, J = 7.9, 5.7 Hz, 3H), 7.23 – 7.13 (m, 4H), 6.94 (t, J = 7.2 Hz, 2H), 4.89 (s, 1H), 4.36 (q, J = 7.1 Hz, 2H), 2.37 (s, 3H), 1.35 (t, J = 7.1 Hz, 3H); ^{13}C NMR (100 MHz, $CDCl_3$) δ 162.0, 157.5, 151.2, 142.0, 138.7, 137.3, 134.8, 130.6, 129.5, 129.1, 126.9, 126.1, 125.7, 125.3, 122.3, 121.6, 117.2, 115.6, 111.3, 93.4, 60.0, 37.7, 21.4, 13.2; HRMS *m/z* calculated for $[C_{26}H_{22}NO_2]^+$ ($[M+H]^+$): 380.1645, observed : 380.1645.

5-ethyl-11a-(*p*-tolyl)-6b,11a-dihydrobenzo[4',5']thieno[2',3':3,4]cyclobuta[1,2-c]quinolin-6(5*H*)-one (5t′)

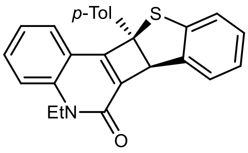

40 mg, >99% yield; white solid; m.p. 146 – 147 °C ^{1}H NMR (400 MHz, $CDCl_3$) δ 7.64 (dd, J = 7.8, 1.5 Hz, 1H), 7.61 – 7.55 (m, 2H), 7.46 (d, J = 8.2 Hz, 3H), 7.27 – 7.18 (m, 2H), 7.18 – 7.12 (m, 4H), 5.09 (s, 1H), 4.35 (qd, J = 7.0, 2.6 Hz, 2H), 2.35 (s, 3H), 1.34 (t, J = 7.1 Hz, 3H); ^{13}C NMR (100 MHz, $CDCl_3$) δ 157.0, 152.8, 142.1, 141.9, 138.0, 136.6, 134.2, 131.7, 130.4, 129.4, 128.4, 127.2, 126.1, 125.2, 125.0, 122.6, 122.1, 116.9, 115.7, 68.6, 66.9, 37.5, 21.1, 13.1.; HRMS *m/z* calculated for $[C_{26}H_{22}NOS]^+$ ($[M+H]^+$): 396.1417, observed 396.1421.

5-ethyl-12-(*p*-tolyl)benzo[*j*]phenanthridin-6(5*H*)-one (6t′)

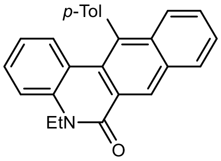

65% yield; ^{1}H NMR (400 MHz, $CDCl_3$) δ 9.28 (s, 1H), 8.12 (d, J = 8.0 Hz, 1H), 7.59 (d, J = 8.7 Hz, 1H), 7.54 (ddd, J = 8.1, 6.7, 1.3 Hz, 1H), 7.47 (ddd, J = 8.3, 6.7, 1.3 Hz, 1H), 7.41 (d, J = 7.9 Hz, 2H), 7.39 – 7.30 (m, 3H), 7.25 (d, J = 9.1 Hz, 2H), 6.78 (ddd, J = 8.4, 6.8, 1.4 Hz, 1H), 4.48 (q, J = 7.1 Hz, 2H), 2.55 (s, 3H), 1.47 (t, J = 7.1 Hz, 3H).; ^{13}C NMR (100 MHz, $CDCl_3$) δ 161.7, 137.8, 137.5, 137.3, 136.5, 135.7, 131.8, 130.31, 130.26,

130.1, 129.4, 129.3, 128.4, 128.0, 127.6, 126.9, 126.2, 124.5, 121.1, 120.9, 114.7, 38.2, 21.5, 12.7.; HRMS *m/z* calculated for $[C_{26}H_{22}NO]^+$ ($[M+H]^+$): 364.1696, observed 364.1697.

5-ethyl-11a-(naphthalen-2-yl)-6b,11a-dihydrobenzo[4',5']thieno[2',3':3,4]cyclobuta[1,2-c]quinolin-6(5*H*)-one (5u′)

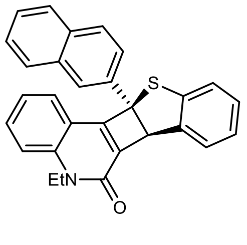

33 mg, 76% yield; white solid; m.p. 205 – 206 °C ^{1}H NMR (400 MHz, $CDCl_3$) δ 7.99 (m, 1H), 7.87 – 7.83 (m, 2H), 7.76 – 7.70 (m, 3H), 7.63 – 7.59 (m, 2H), 7.51 – 7.44 (m, 3H), 7.29 – 7.16 (m, 4H) 5.21 (s, 1H), 4.41 – 4.35 (m, 2H), 1.37 (t, *J* = 7.1 Hz, 3H); ^{13}C NMR (100 MHz, $CDCl_3$) δ 157.2, 152.9, 142.3, 142.2, 137.0, 134.3, 133.09, 133.05, 131.9, 130.7, 129.0, 128.7, 128.3, 127.8, 126.68, 126.67, 126.4, 126.0, 125.47, 125.45, 125.2, 122.8, 122.4, 117.1, 115.9, 68.6, 67.4, 37.7, 13.2; HRMS *m/z* calculated for $[C_{29}H_{22}NOS]^+$ ($[M+H]^+$): 432.1417, observed 432.1417.

5-ethyl-12-(naphthalen-2-yl)benzo[*j*]phenanthridin-6(5*H*)-one (6u′)

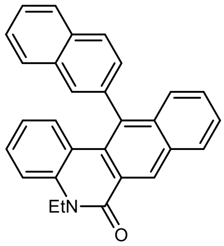

86% yield; ^{1}H NMR (400 MHz, $CDCl_3$) δ 9.33 (m, 1H), 8.15 (d, *J* = 8.2 Hz, 1H), 8.10 (d, *J* = 8.4 Hz, 1H), 8.03 (d, *J* = 8.0 Hz, 1H), 7.87 – 7.85 (m, 2H), 7.64 – 7.50 (m, 5H), 7.45 – 7.41 (m, 1H), 7.39 – 7.34 (m, 2H), 7.31 – 7.27 (m, 1H), 6.61 (ddd, *J* = 8.4, 7.0, 1.3 Hz, 1H), 4.50 (q, *J* = 7.1 Hz, 2H), 1.49 (t, *J* = 7.1 Hz, 3H); ^{13}C NMR (100 MHz, $CDCl_3$) δ 161.8, 138.6, 137.5, 136.3, 135.8, 134.1, 132.9, 132.0, 130.6, 129.61, 129.55, 129.5, 129.3, 128.9, 128.7, 128.4, 128.3, 128.2, 128.0, 127.0, 126.7, 126.6, 126.5, 124.7, 121.4, 120.8, 114.9, 38.4, 12.8; HRMS *m/z* calculated for $[C_{29}H_{22}NO]^+$ ($[M+H]^+$): 400.1696, observed 400.1696.

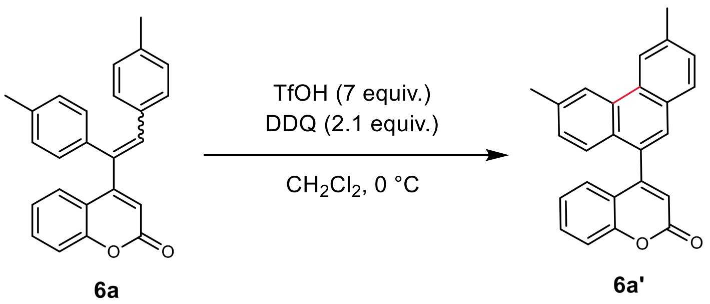

To a mixture of diene **6a** (0.2 mmol, 1.0 equiv.) and DDQ (2.1 equiv.) in dry CH_2Cl_2 (7 mL, 0.03 M) at 0°C was added trifluoromethanesulfonic acid (7.0 equiv.) dropwise. The reaction mixture was stirred at 0 °C for 3 hours. The mixture was quenched by saturated $NaHCO_3$ solution and extracted by CH_2Cl_2. The combined organic layer was dried over Na_2SO_4, filtered and concentrated. The residue was purified by flash chromatography on silica gel to give the desired product **6a′**. 43 mg, 61% yield; white solid; m.p. 199 – 200 °C; ^{1}H NMR (400 MHz, $CDCl_3$) δ 8.57 (s, 1H), 8.54 (s, 1H), 7.80 (d, *J* = 8.1 Hz, 1H), 7.64 (s, 1H), 7.55 – 7.42 (m, 4H), 7.32 (d, *J* = 8.3 Hz, 1H), 7.08 – 7.00 (m, 2H), 6.57 (s, 1H), 2.67 (s, 3H), 2.63 (s, 3H); ^{13}C NMR (100 MHz, $CDCl_3$) δ 160.9, 155.6, 153.7, 137.5, 136.9, 131.9, 130.6, 130.5, 130.2, 129.0, 128.9, 128.8, 128.7, 127.8, 127.6, 126.5, 126.2, 124.2, 122.9, 122.4, 120.1, 117.1, 116.7, 22.2, 22.0; HRMS *m/z* calculated for $[C_{25}H_{19}O_2]^+$ ($[M+H]^+$): 351.1380, observed 351.1380.

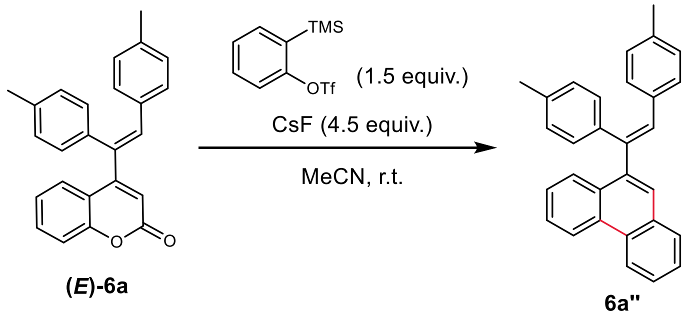

To a solution of diene **(*E*)-6a** (0.045 mmol, 1.0 equiv.) and 2-(trimethylsilyl)phenyl trifluoromethanesulfonate (1.5 equiv.) in THF (0.45 mL, 0.1 M) was added cesium fluoride (4.5 equiv.). The reaction mixture was stirred at room temperature for 18 hours. The reaction mixture was concentrated under vacuum. The residue was purified by flash chromatography on silica gel. 11 mg, 64% yield of **6a"**; White solid; ^{1}H NMR (400 MHz, $CDCl_3$) δ 8.70 (t, *J* = 8.4 Hz, 2H), 8.08 (d, *J* = 8.3 Hz, 1H), 7.89 (d, *J* = 7.7 Hz, 1H), 7.69 – 7.55 (m, 3H), 7.46 (ddq, *J* = 8.1, 6.9, 1.3 Hz, 1H), 7.21 (d, *J* = 8.1 Hz, 2H), 7.15 (d, *J* = 8.0 Hz, 2H), 7.04 (d, *J* = 7.7 Hz, 2H), 7.00 (d, *J* = 7.8 Hz, 2H), 6.83 (s, 1H), 2.34 (s, 3H), 2.30 (s, 3H); ^{13}C NMR (100 MHz, $CDCl_3$) δ 140.9, 140.8, 137.5, 137.0, 136.7, 134.6, 131.6, 131.2, 130.8, 130.7, 130.1, 129.5, 129.4, 129.0, 128.8, 128.6, 127.9, 127.2, 126.7, 126.5, 126.4, 126.2, 122.8, 122.5, 76.7, 21.3; HRMS *m/z* calculated for $[C_{30}H_{24}Na]^+$ ($[M+Na]^+$): 407.1770, observed 407.1770.

2-(trimethylsilyl)phenyl trifluoromethanesulfonate (S12)

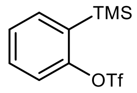

To a solution of 2-bromophenol (1.5 mmol, 1.0 equiv.) in THF (3 mL, 0.5 M) was added HMDS (2.0 equiv.) at room temperature. The reaction mixture was reflux for 3 hours. Then the solvent and remained HMDS was removed under vacuum to obtain 2-bromophenyl trimethylsilyl ether, which was used in the next step without purification. To a solution of 2-bromophenyl trimethylsilyl ether in THF (2.1 mL, 0.7 M) was added n-BuLi (2.0 equiv., 2.5 M in hexane) dropwise at -78 °C. After 20 min, Trifluoromethanesulfonic anhydride (2.0 equiv.) was added to the mixture, and stirred 1 hour at -78 °C. The mixture was quenched by saturated $NaHCO_3$ solution and extracted by EtOAc. The combined organic layer was dried over Na_2SO_4, filtered and concentrated. The residue was purified by flash chromatography on silica gel. 39% yield; colorless liquid; ^{1}H NMR (400 MHz, $CDCl_3$) δ 7.54 (dd, J = 7.5, 1.9 Hz, 1H), 7.47 – 7.39 (m, 1H), 7.37 – 7.31 (m, 2H), 0.37 (s, 9H); The structure was further confirmed by spectral comparison with literature data.[52]

Synthetic applications

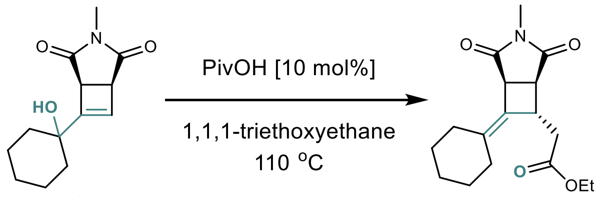

3qa **7**

To a solution of cyclobutene **3qa** (0.1 mmol, 1.0 equiv.) in triethyl orthoacetate (0.5 mL, 0.2 M) was added a catalytic quantity of trimethylacetic acid (0.1 equiv.) at room temperature. The reaction mixture was stirred at 110 °C for 24 hours. The reaction mixture was washed with saturated aqueous $NaHCO_3$ and extracted with CH_2Cl_2. The combined organic layer was dried over Na_2SO_4, filtered and concentrated. The residue was purified by flash chromatography on silica gel to give the desired product **7**. 18 mg, 60% yield; colorless liquid; ^{1}H NMR (400 MHz, $CDCl_3$) δ 4.16 (q, J = 7.1 Hz, 2H), 3.90 (d, J = 6.1 Hz, 1H), 3.34 (d, J = 3.5 Hz, 1H), 3.14 (dd, J = 6.1, 3.0 Hz, 1H), 2.97 (s, 3H), 2.74 (dd, J = 15.5, 4.2 Hz, 1H), 2.60 (dd, J = 15.5, 9.3 Hz, 1H), 2.21 (s, 2H), 2.00 – 1.85 (m, 2H), 1.49 (s, 6H), 1.27 (t, J = 7.5 Hz, 3H) ; ^{13}C NMR (100 MHz, $CDCl_3$) δ 178.0, 176.3, 170.9, 141.8, 121.9, 60.7, 45.4, 41.5, 41.0, 38.5, 30.0, 29.6, 27.5, 27.4, 26.0, 25.1, 14.2; HRMS *m/z* calculated for $[C_{17}H_{24}NO_4]^+$ ($[M+H]^+$): 306.1700, observed 306.1701.

Synthesis of 3-methyl-6,7-di-*p*-tolyl-3-azabicyclo[3.2.0]hept-6-en-2-one (8)

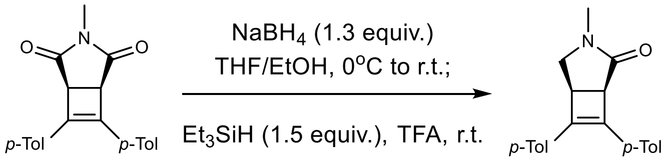

3aa **8**

To a solution of cyclobutene **3aa** (0.1 mmol, 1.0 equiv.) in dry THF/EtOH (0.3 mL/0.7 mL, 0.1 M) was slowly added $NaBH_4$ (1.3 equiv.) at 0 °C under nitrogen atmosphere and stirred the mixture overnight at room temperature. When TLC indicated the reaction was complete, the solution was adjusted to slightly acidic pH with glacial acetic acid (few drops), then concentrated *in vacuo*. The resulting mixture was diluted in EtOAc, washed with sat. $NaHCO_3$ solution and extracted with EtOAc. The combined organic layer was dried over Na_2SO_4, filtered and concentrated under reduced pressure. To the hydroxylactam prepared above was added triethylsilane (1.5 equiv.) followed by TFA (0.5 mL, 0.2 M). The reaction was stirred overnight at room temperature under nitrogen atmosphere. When TLC indicated the reaction was complete, the solution was concentrated under reduced pressure. The resulting crude product was purified by flash column chromatography on silica gel. 21 mg, 69% yield; white solid; m.p. 88 – 90 °C; ^{1}H NMR (400 MHz, $CDCl_3$) δ 7.73 (d, *J* = 8.1 Hz, 2H), 7.42 (d, *J* = 7.9 Hz, 2H), 7.17 (d, *J* = 7.9 Hz, 2H), 7.13 (d, *J* = 8.0 Hz, 2H), 3.72 (d, *J* = 4.2 Hz, 1H), 3.66 (ddd, *J* = 8.4, 4.4, 2.0 Hz, 1H), 3.49 (dd, *J* = 10.2, 8.4 Hz, 1H), 3.33 (dt, *J* = 10.2, 1.8 Hz, 1H), 2.80 (s, 3H), 2.37 (s, 3H), 2.34 (s, 3H); ^{13}C NMR (100 MHz, DMSO-d_6) δ 172.2, 140.9, 139.6, 138.0, 137.8, 131.4, 130.4, 129.4, 129.0, 126.4, 126.3, 49.0, 46.6, 34.0, 29.7, 21.0, 21.0; HRMS *m/z* calculated for $[C_{21}H_{22}NO]^+$ ($[M+H]^+$): 304.1696, observed : 304.1697

Synthesis of 3-methyl-6,7-diphenyl-3-azabicyclo[3.2.0]hept-6-ene (9)

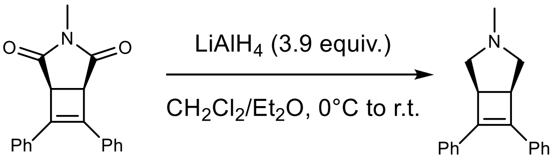

3ca **9**

To a slurry of $LiAlH_4$ (3.9 equiv.) in Et_2O (0.25 mL, 0.2 M) was added solution of cyclobutene **3ca** (0.05 mmol, 1.0 equiv.) in CH_2Cl_2 (0.5 mL, 0.1 M) at 0°C under nitrogen. The mixture was stirred at room temperature for 1 hour, and H_2O was carefully added at 0 °C. To reaction suspension was dried over Na_2SO_4, filtered and concentrated. The residue was purified by flash chromatography on silica gel to give the desired product. 9 mg, 69% yield; yellow oil; ^{1}H NMR (400 MHz, $CDCl_3$) δ 7.59 – 7.54 (m, 4H), 7.34 – 7.28 (m, 4H), 7.27 – 7.21 (m, 2H), 3.54 (d, *J* = 6.0 Hz, 2H), 3.14 (d, *J* = 9.9 Hz, 2H), 2.36 (s, 3H), 2.08 – 1.98 (m, 2H); ^{13}C NMR (100 MHz, $CDCl_3$) δ 139.0, 134.4, 128.4, 128.0, 126.5, 55.5, 44.1, 42.2.; HRMS *m/z* calculated for $[C_{19}H_{20}N]^+$ ($[M+H]^+$): 262.1590, observed 262.1592.

Supplementary Discussion

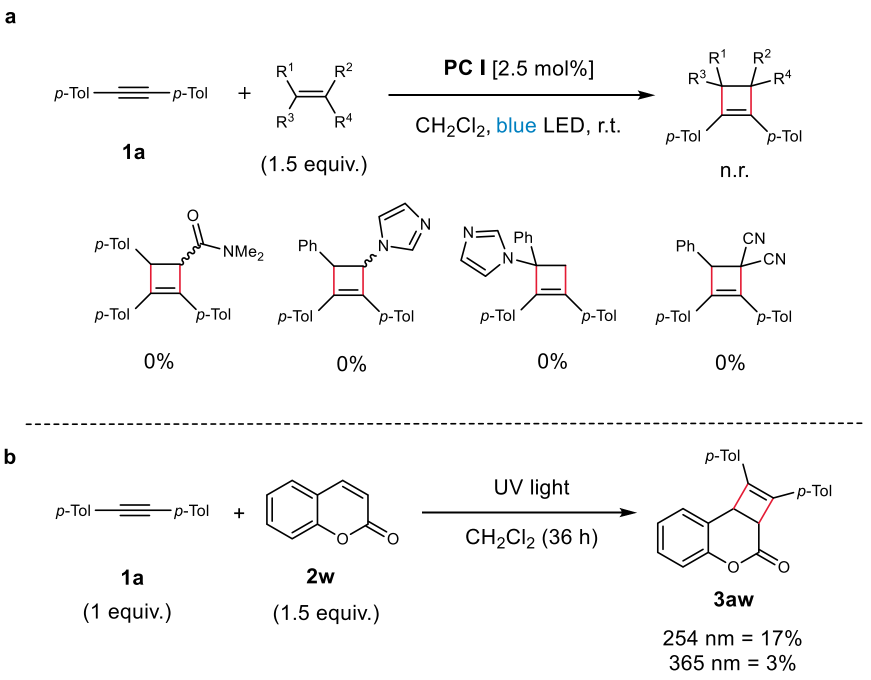

Supplementary Figure 1. a : Scope of failed substrates. **b :** Reactions under UV irradiation.

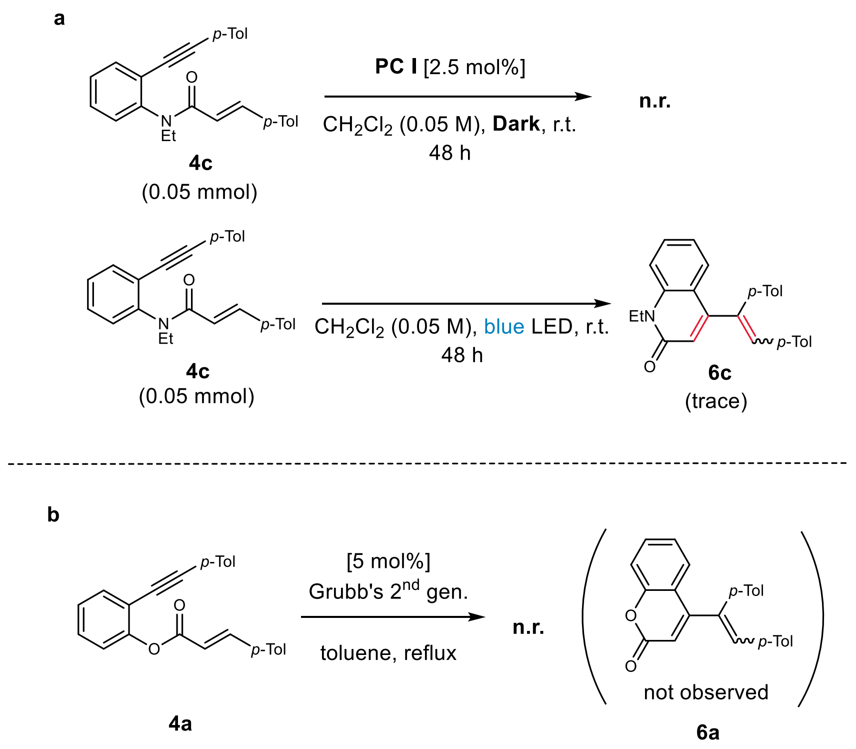

Supplementary Figure 2. a : Control experiments for intramolecular reaction of enyne. **b :** Enyne metathesis with Grubb's catalyst.

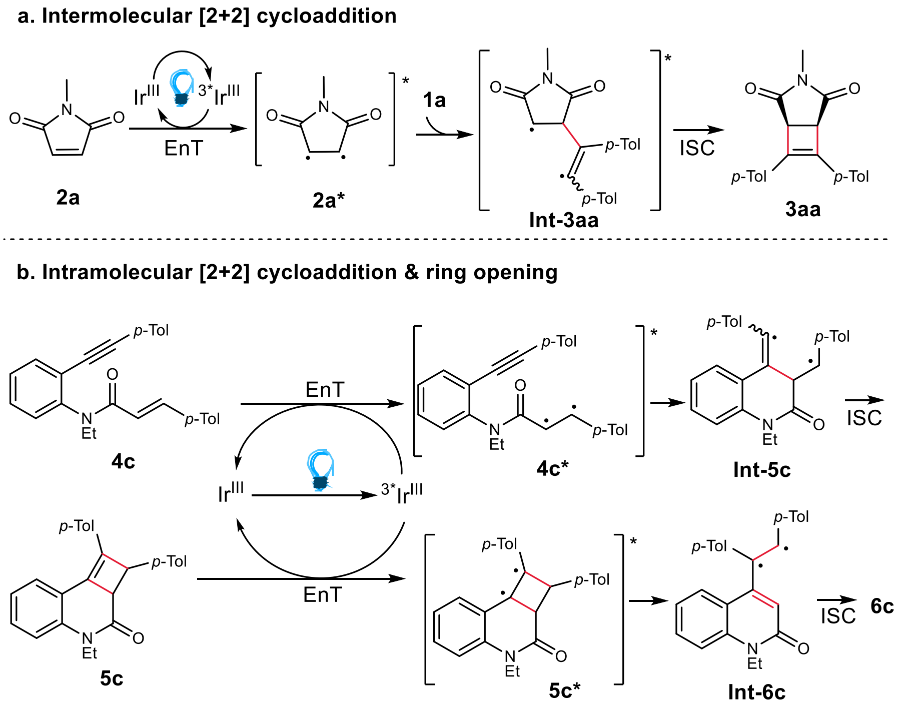

Supplementary Figure 3. Proposed mechanism. **a** Intermolecular [2+2] cycloaddition **b** Intramolecular [2+2] cycloaddition and ring opening

Effect of Triplet Quencher

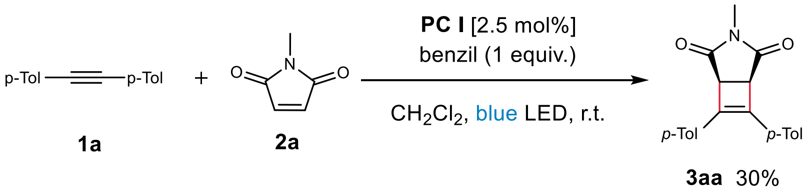

di(*p*-tolyl)acetylene **1a** (0.05 mmol, 1.0 equiv.), *N*-methylmaleimide **2a** (1.5 equiv.), photocatalyst Ir[dF(CF_3)ppy]$_2$(dtbbpy)PF_6 (2.5 mol%) and triplet quencher benzil (1.0 equiv.) were added to an oven-dried 4mL vial equipped with a stir bar. The combined materials were dissolved in CH_2Cl_2 (2 mL) under argon atmosphere in glovebox. The reaction mixture was then irradiated by 12 W blue LED lamp at room temperature (maintained with a cooling fan) for 4 hours. the solution was concentrated under reduced pressure. The yield was determined by ^{1}H NMR analysis ($CDCl_3$) of the crude reaction mixture using trichloroethylene as the internal standard; 30% yield.

Radical Clock Experiments

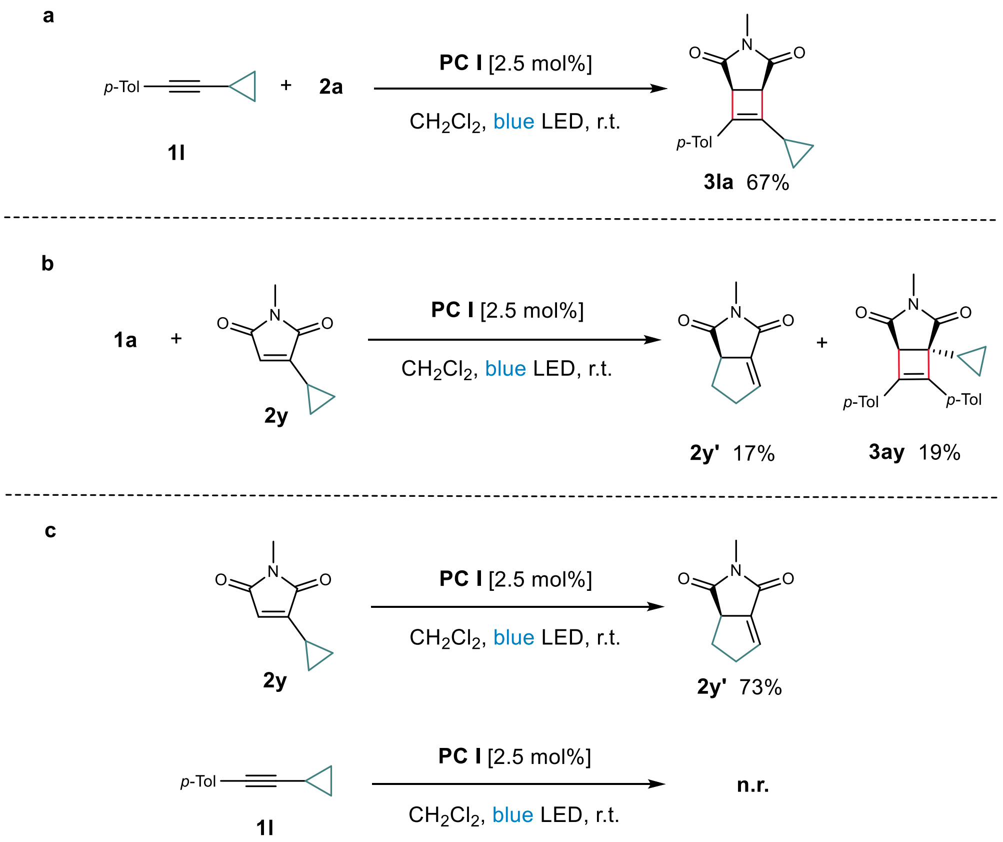

a)

See Supplementary Methods for experimental details.

b)

Di(*p*-tolyl)acetylene **1a** (0.1 mmol, 1.0 equiv.), 3-cyclopropyl-1-methyl-1H-pyrrole-2,5-dione **2y** (1.5 equiv.), and photocatalyst Ir[dF(CF$_3$)ppy]$_2$(dtbbpy)PF$_6$ (2.5 mol%) were added to an oven-dried 4 mL vial equipped with a stir bar. The combined materials were dissolved in CH_2Cl_2 (2 mL) under argon atmosphere in glovebox. The reaction mixture was then irradiated by 12 W blue LED lamp at room temperature (maintained with a cooling fan) for 48 hours. the solution was concentrated under reduced pressure. The residue was purified by flash chromatography on silica gel to give **3ay** (19% yield) and **2y′** (17% yield).

1-cyclopropyl-3-methyl-6,7-di-*p*-tolyl-3-azabicyclo[3.2.0]hept-6-ene-2,4-dione (3ay)

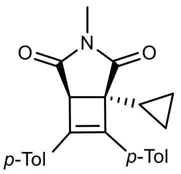

^1H NMR (400 MHz, $CDCl_3$) δ 7.65 (d, J = 8.2 Hz, 2H), 7.56 (d, J = 8.3 Hz, 2H), 7.18 (d, J = 8.0 Hz, 1H), 7.14 (d, J = 8.0 Hz, 1H), 3.49 (s, 1H), 2.97 (s, 3H), 2.37 (s, 3H), 2.34 (s, 3H), 1.59 – 1.51 (m, 1H), 0.68 – 0.60 (m, 1H), 0.59 – 0.50 (m, 2H), 0.37 – 0.25 (m, 1H); ^{13}C NMR (100 MHz, $CDCl_3$) δ 177.8, 175.0, 141.7, 139.3, 139.0, 137.7, 130.3, 129.9, 129.4, 129.4, 127.5, 126.8, 55.7, 47.3, 25.0, 21.63, 21.60, 10.6, 2.5, 1.5; HRMS *m/z* calculated for $[C_{24}H_{24}NO_2^+]^+$ ($[M+H]^+$): 358.1802, observed 358.1802.

2-methyl-4,5-dihydrocyclopenta[*c*]pyrrole-1,3(2*H*,3a*H*)-dione (2y′)

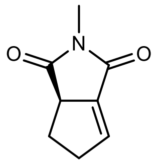

^1H NMR (400 MHz, $CDCl_3$) δ 6.66 (dt, J = 3.7, 2.5 Hz, 1H), 3.80 (td, J = 7.9, 3.8 Hz, 1H), 3.00 (s, 3H), 2.98 – 2.83 (m, 1H), 2.74 (ddd, J = 17.6, 8.6, 3.8 Hz, 1H), 2.46 (dt, J = 13.8, 7.0 Hz, 1H), 2.03 (dtd, J = 12.5, 10.8, 8.6 Hz, 1H); ^{13}C NMR (100 MHz, $CDCl_3$) δ 176.0, 165.1, 138.4, 136.3, 51.1, 37.4, 28.8, 24.6; HRMS *m/z* calculated for $[C_8H_{10}NO_2^+]^+$ ($[M+H]^+$): 152.0706, observed 152.0708.

c)

3-cyclopropyl-1-methyl-1H-pyrrole-2,5-dione **2y** (1.0 equiv.) and photocatalyst $Ir[dF(CF_3)ppy]_2(dtbbpy)PF_6$ (2.5 mol%) were added to an oven-dried 4mL vial equipped with a stir bar. The combined materials were dissolved in CH_2Cl_2 (2 mL) under argon atmosphere in glovebox. The reaction mixture was then irradiated by 12 W blue LED lamp at room temperature (maintained with a cooling fan) for 4 hours. the solution was concentrated under reduced pressure. The residue was purified by flash chromatography on silica gel to give **2y′** (73% yield).

1-(cyclopropylethynyl)-4-methylbenzene **1l** (0.1 mmol, 1.0 equiv.) and photocatalyst $Ir[dF(CF_3)ppy]_2(dtbbpy)PF_6$ (2.5 mol%) were added to an oven-dried 4mL vial equipped with a stir bar. The combined materials were dissolved in CH_2Cl_2 (2 mL) under argon atmosphere in glovebox. The reaction mixture was then irradiated by 12 W blue LED lamp at room temperature (maintained with a cooling fan) for 48 hours. the solution was concentrated under reduced pressure. The reaction wasn't processed. (determined by ^1H NMR analysis ($CDCl_3$) of the crude reaction mixture.)

Visible light irradiation on/off experiments.

Both reactions were conducted under irradiation of 12 W blue LED strip and Ar atmosphere. Light was switched off during the "off" periods. Ratio determined by ^1H NMR spectroscopic analysis.

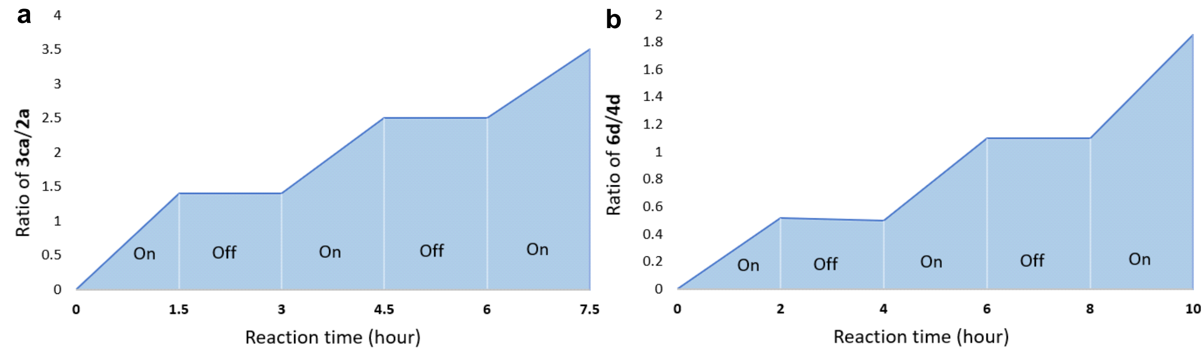

Supplementary Figure 4. Visible light irradiation on/off experiments. **a** : 0.025 mmol of **1c**, 1.5 equiv. of **2a** and 2.5 mol% of **PC I** in $CDCl_3$. **b** : 0.05 mmol of **4d** and 2.5 mol% of **PC I** in $CDCl_3$.

Quantum yield measurement and UV-Vis absorption spectra of substrates

Quantum yields were measured following a procedure by Yoon and coworkers.[53] The reaction yield can be converted to its quantum yield using the pre-determined photon flux of the system.

<u>Determination of the light intensity at 436 nm</u>

The effective photon flux of the used fluorometer was determined using standard ferrioxalate actinometry. A 0.15 M ferrioxalate solution was prepared by dissolving potassium ferrioxalate hydrate (221 mg, 0.45 mmol) in 0.05 M H_2SO_4 (3 mL). A buffer solution of 1,10-phenanthroline was prepared by dissolving 1,10-phenanthroline (10 mg, 0.055 mmol) and sodium acetate (2.25 g) in 0.5 M H_2SO_4 (10 mL). The ferrioxalate solution (0.4 mL) was added to cuvette and was irradiated at 436 nm for 90 s in the fluorometer. After irradiation, the phenanthroline solution (0.07 mL) was added to the cuvette and the mixture was allowed in the absence of light for 1 h to achieve full phenanthroline coordination to the ferrous ions. In addition, a non-irradiated sample was prepared similarly.

The absorbance of both samples was measured at 510 nm. From these values, conversion could be determined using Lambert-Beer's law :

$$\boldsymbol{n}(\mathbf{Fe^{2+}}) = \frac{V \cdot \boldsymbol{\Delta A}}{\boldsymbol{l} \cdot \boldsymbol{\varepsilon}} \quad (1)$$

Where V is the total volume (0.47 mL) of the solution after addition of phenanthroline, l is the optical path length of the cuvette (1.00 cm), ε is the molar absorptivity of the ferrioxalate actinometer (11,100 L mol^{-1} cm^{-1}) and ΔA is the absorbance difference between the irradiated at 436 nm and non-irradiated sample (1.305).

From this value, the photon flux Φ_q in the system can be calculated as :

$$\boldsymbol{\Phi_q} = \frac{\boldsymbol{n}(\mathbf{Fe}^{2+})}{\boldsymbol{\Phi_F} \cdot \boldsymbol{t} \cdot \boldsymbol{f}} \quad (2)$$

Where Φ_F is the quantum yield of the ferrioxalate system (1.01 for 0.15 M solution at λ_{ex} = 436 nm), t is the irradiation time (90.0 s) and f is the fraction of light absorbed at λ_{ex} = 436 nm (0.9987, See Supplementary Equation 3). The absorption fraction is calculated as :

$$f = 1\text{-}10^{-A} \quad (3)$$

Where A in Supplementary Equation 3 is the measured absorbance of ferrioxalate solution at 436 nm. It was measured to be 2.873.

The photon flux Φ_q was calculated (average of two experiments) to be 6.09×10^{-10} einstein s^{-1}.

Determination of quantum yield

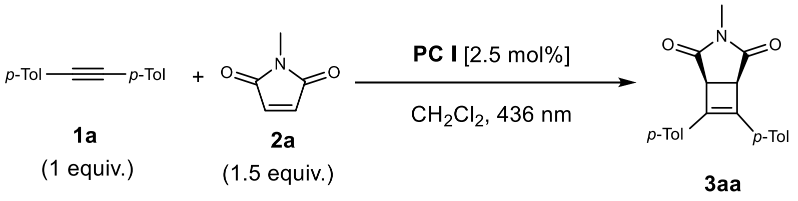

A cuvette was charged with alkyne **1a** (0.025 mmol, 1 equiv.), alkene **2a** (2 equiv.), Ir[dF(CF_3)ppy]$_2$(dtbbpy)PF_6 (2.5 mol%), and CH_2Cl_2 (0.5 mL, 0.05 M) in Ar charged glovebox. The sample was sealed with parafilm and irradiated (λ = 436 nm, slit width = 20.0 nm) for 7,200 s (2 h). After irradiation, the solvent was evaporated with a stream of nitrogen. The yield of product **3aa** formed was determined by ^{1}H NMR based on a trichloroethylene as internal standard. The quantum yield (average of two experiments) was determined using Supplementary Equation 4.

$$\boldsymbol{\Phi} = \frac{\boldsymbol{n(product)}}{\boldsymbol{\Phi_q} \cdot \boldsymbol{t} \cdot \boldsymbol{f}} \quad (4)$$

The absorbance of photocatalyst in CH_2Cl_2 was measured at the reaction concentration of 1.25×10^{-3} M. The absorbance at 436 nm is 1.91 indicating the fraction of light absorbed (f) is 0.9877.

The quantum yield Φ was calculated (The yields of product **3aa** are 15% and 16% in two experiments) to be 0.91.

UV-Vis absorption spectrum data

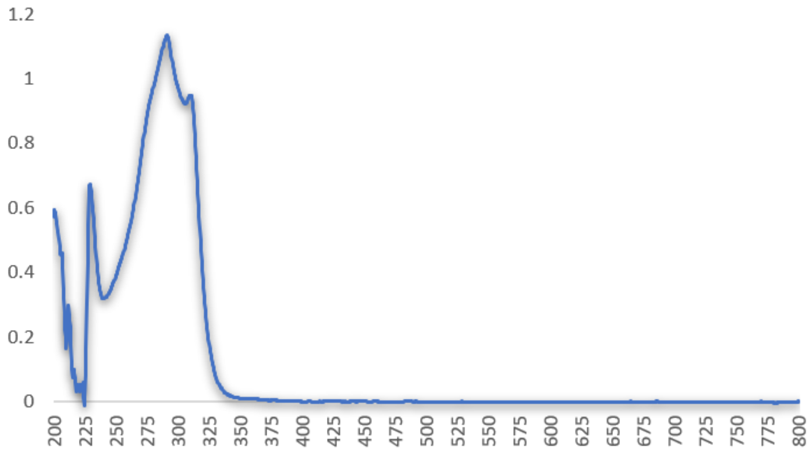

Supplementary Figure 5. UV-Vis absorption spectrum data of **4c**.

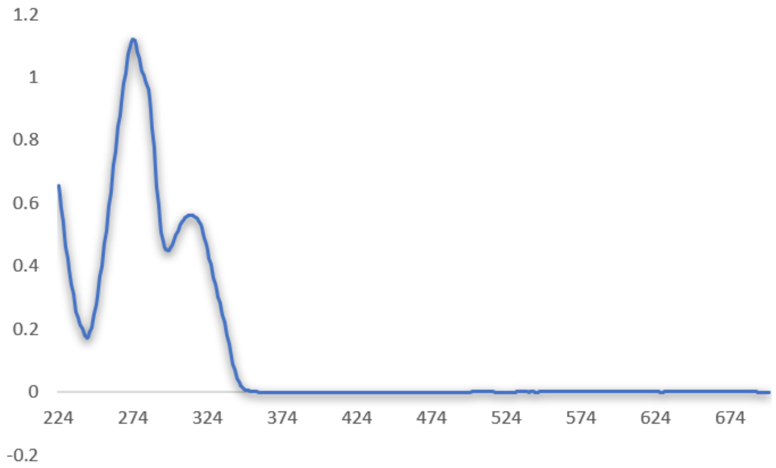

Supplementary Figure 6. UV-Vis absorption spectrum data of **2w**.

Stern-Volmer luminescence quenching experiments

Stern-Volmer luminescence quenching studies were carried out using a 0.1 mM solution of photocatalyst and variable concentrations of substrate in dry DCM at room temperature under an argon atmosphere. The samples were prepared in 0.5 mL quartz cuvettes inside an argon filled glove-box, and sealed with parafilm. The solutions were irradiated at 420 nm and the luminescence was measured at maximum emission wavelength of each photocatalyst. (I_0 = emission intensity of the photocatalyst in isolation at the specified wavelength; I = observed intensity as a function of the quencher concentration)

Photocatalyst (0.1 mM)		Quencher : 1a					
Cat.	Max emission wavelength	0 mM	2 mM	4 mM	6 mM	8 mM	10 mM
**PC I**	472 nm	1	3.0568	4.7348	7.1689	9.5936	12.1958
**PC IV**	505 nm	1	1.4928	2.2200	2.6175	3.2100	3.8640
**PC V**	527 nm	1	1.1203	1.3385	1.4381	1.6377	1.7515
**PC VI**	562 nm	1	1.0115	1.0763	1.0814	1.0106	1.0279

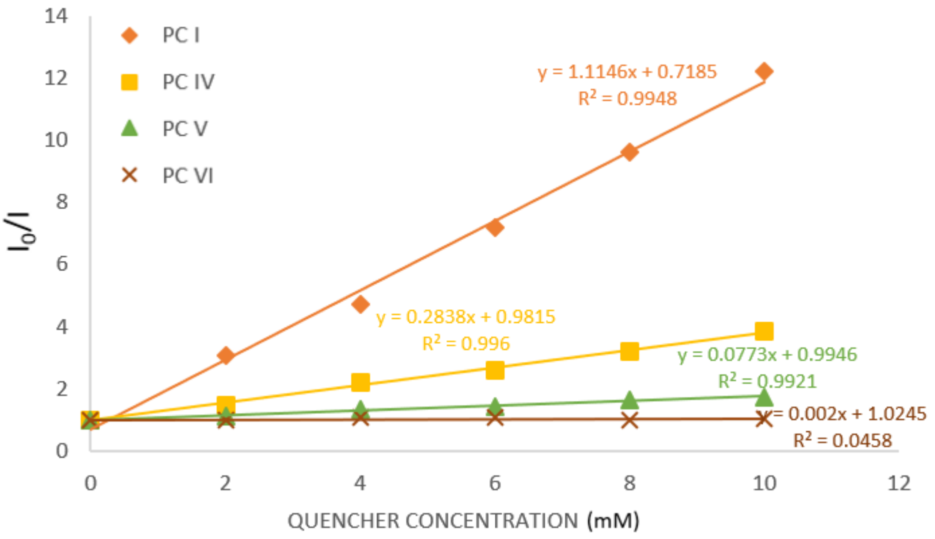

Supplementary Figure 7. Stern-Volmer plot using **1a** as a quencher.

Photocatalyst (0.1 mM)		Quencher : 2a					
Cat.	Max emission wavelength	0 mM	2 mM	4 mM	6 mM	8 mM	10 mM
**PC I**	472 nm	1	1.0900	1.6040	1.6821	2.0379	2.7735
**PC IV**	505 nm	1	1.0334	1.0874	1.2463	1.3397	1.4432
**PC V**	527 nm	1	0.9846	0.9468	1.1190	1.1403	1.1878
**PC VI**	562 nm	1	0.9530	0.8810	1.02276	0.9031	0.9291

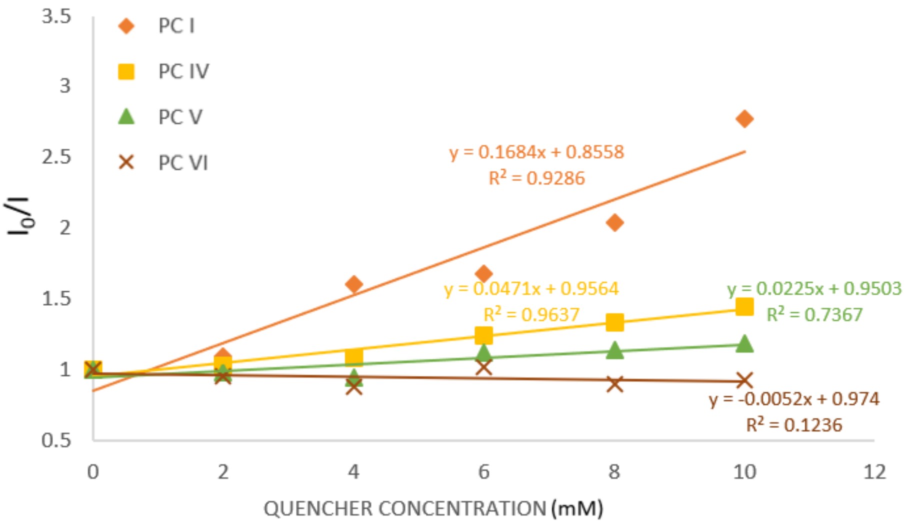

Supplementary Figure 8. Stern-Volmer plot using **2a** as a quencher.

Photocatalyst (0.1 mM)		Quencher : 4c					
Cat.	Max emission wavelength	0 mM	2 mM	4 mM	6 mM	8 mM	10 mM
**PC I**	472 nm	1	5.0653	10.3184	17.0076	22.4114	33.8915
**PC IV**	505 nm	1	1.6578	2.8213	4.2890	5.2490	5.9022
**PC V**	527 nm	1	1.2920	1.5187	1.6922	1.9659	2.2069
**PC VI**	562 nm	1	1.0597	1.1066	1.1730	1.2388	1.3084

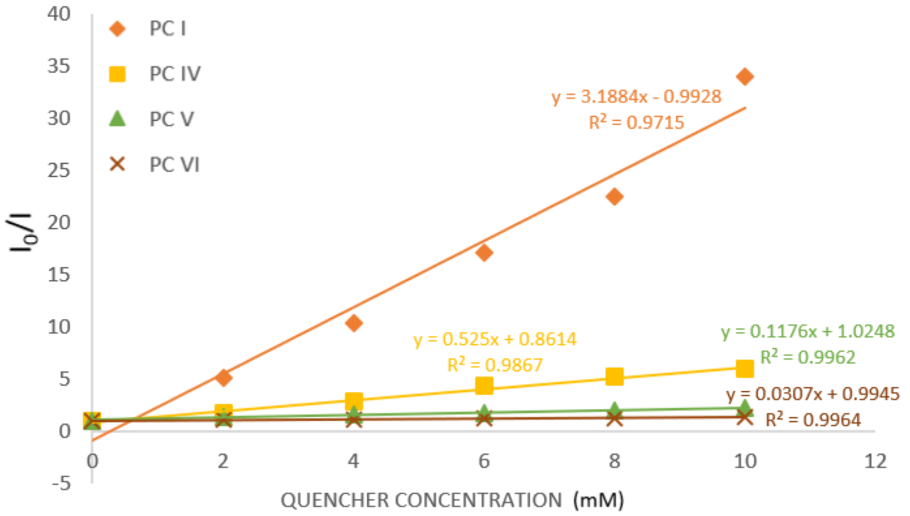

Supplementary Figure 9. Stern-Volmer plot using **4c** as a quencher.

Photocatalyst (0.1 mM)		Quencher : 1m					
Cat.	Max emission wavelength	0 mM	2 mM	4 mM	6 mM	8 mM	10 mM
**PC I**	472 nm	1	0.9692	0.9610	0.9834	0.9275	0.9778
**Photocatalyst (0.1 mM)**		**Quencher : 2a**					
Cat.	Max emission wavelength	0 mM	2 mM	4 mM	6 mM	8 mM	10 mM
**PC I**	472 nm	1	1.0900	1.6040	1.6821	2.0379	2.7735

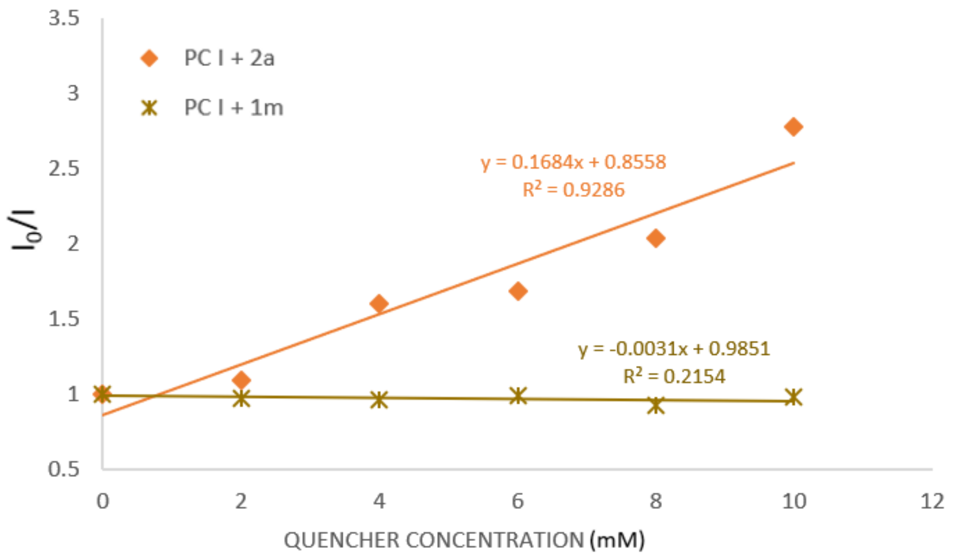

Supplementary Figure 10. Stern-Volmer plot using **1m** and **2a** as quenchers.

Electrochemical measurements with cyclic voltammogram

Electrochemical measurement :

Samples for electrochemical measurements were prepared with 0.03 mmol of substrate in anhydrous degassed 0.1 M Bu_4NPF_6 solution in MeCN (3 mL). The corresponding cyclic voltammograms were collected by a Potentiostat equipped with a glassy carbon working electrode, platinum wire counter electrode, 3 M KCl Ag/AgCl reference electrode, and a scan rate of 100 mV/s; Data were analyzed by subtracting the electrolyte solution background current prior to identifying the maximum current (Cp) and determining the potential ($E_{p/2}$) at half this value ($C_{p/2}$). The obtained value was referenced to Ag/AgCl and converted to SCE by subtracting 0.04 V.

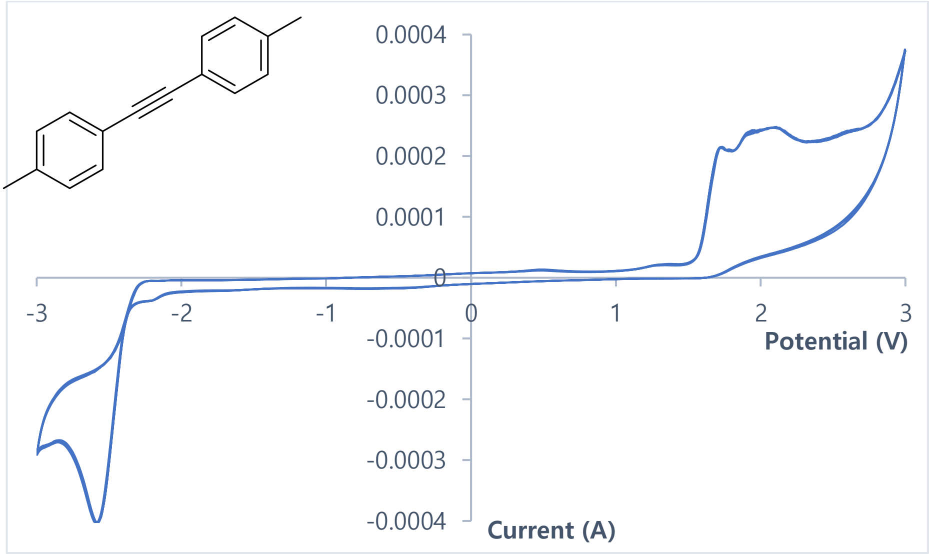

$E_{p/2}^{ox}$ = +1.59 V vs SCE, $E_{p/2}^{red}$ = -2.50 V vs SCE

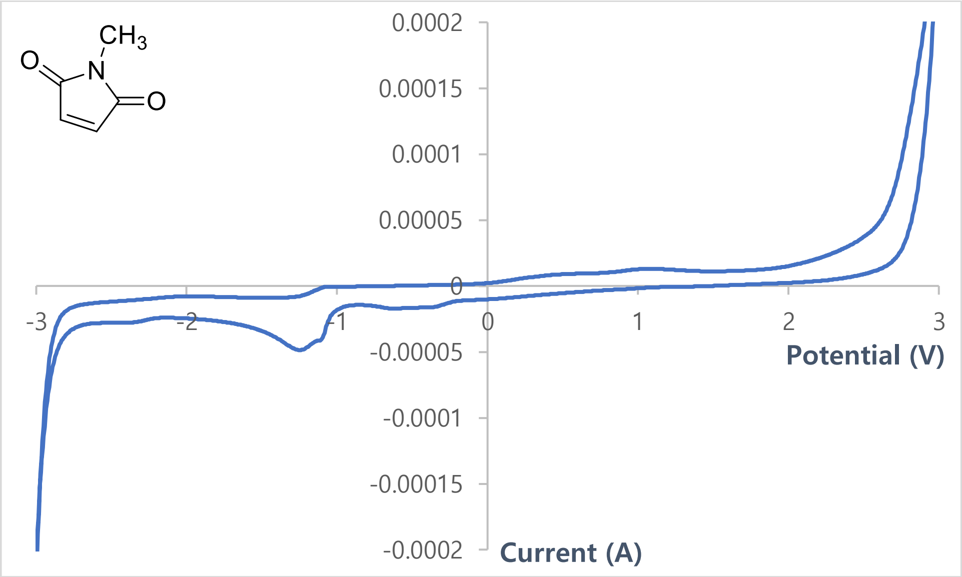

$E_{p/2}^{red}$ = -1.16 V vs SCE

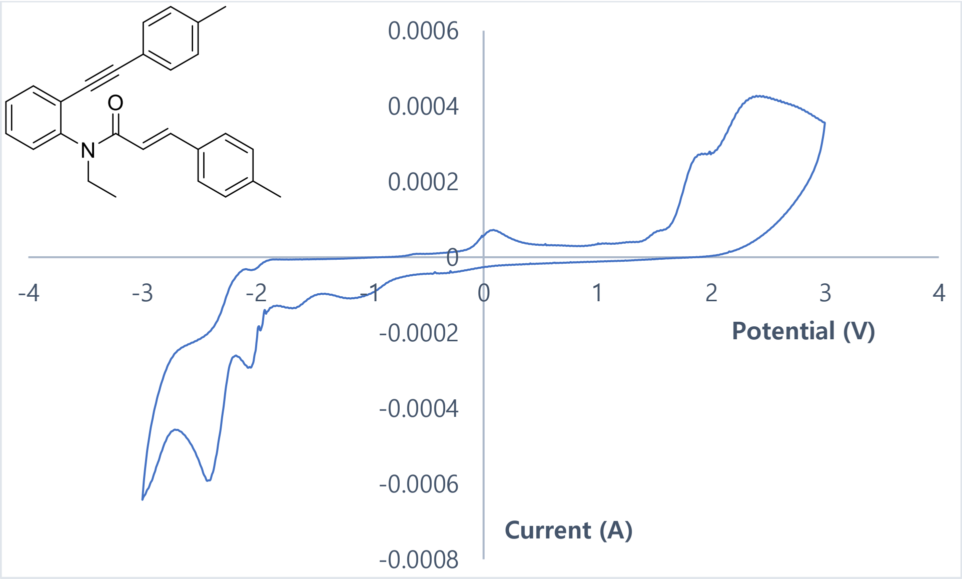

$E_{p/2}^{ox}$ = +1.82, +0.11 V vs SCE, $E_{p/2}^{red}$ = -2.13, -2.44 V vs SCE

Supplementary Figure 11. Cyclic voltammetry of **1a, 2a** and **4c**.

Computational details and references

Computational details

All DFT calculations were carried out in the Gaussian 09 software (Rev D.01)[54] using the M06 functional[55]. Geometry optimization was performed with the 6-311+g(d,p) basis set[56, 57] for H, C, N, O atoms, which were obtained from the EMSL Basis Set Exchange[58, 59]. Frequency calculations were performed for every optimized geometry with the same level of theory to obtain vibrational frequencies and thermochemical data at 298.15K. The SMD solvation model[60] with the solvent of dichloromethane (ε= 8.930) was used for all calculations. The transition states were identified by having one imaginary frequency, and intrinsic reaction coordinate[61, 62] (IRC) calculations were performed to connect transition states with corresponding intermediates. Each intermediate was verified as minima by having no imaginary frequency, and the geometries of intermediates with possibility of multiple conformations were optimized with several different starting geometries to find the lowest energy conformation. Triplet energies were calculated by the previously reported method[63-65] which is the difference between two free energies of singlet and triplet states. Unrestricted broken symmetry calculations were performed for the open-shelled singlet geometries. Open-shelled singlet geometries were confirmed by the S^2 values obtained in which the expected value for open-shelled singlet is around 1[66].

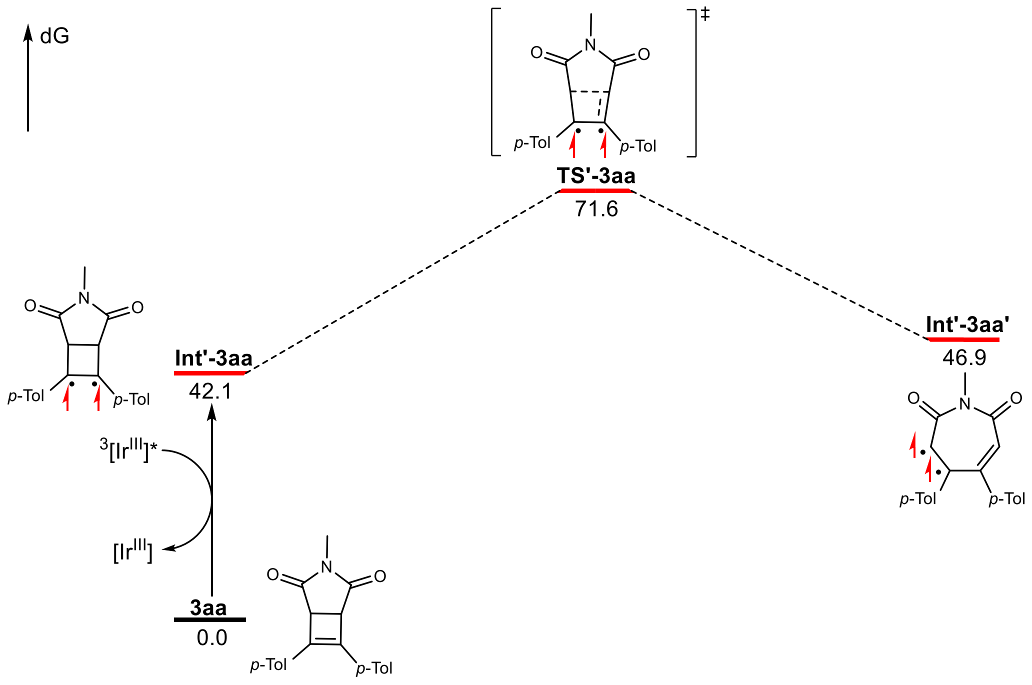

Supplementary Figure 12. DFT calculation : Ring-opening reaction of cyclobutene **3aa**.

Mulliken spin densities and images of spin density surfaces

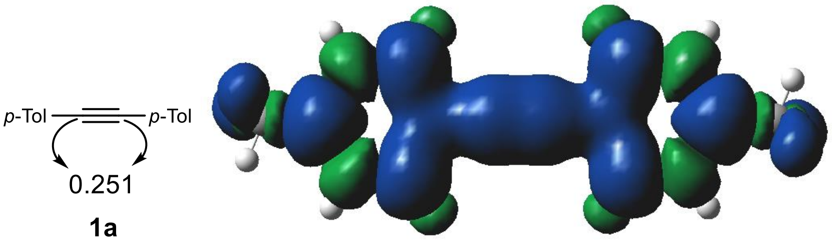

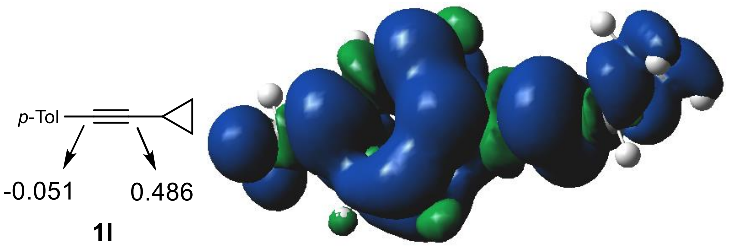

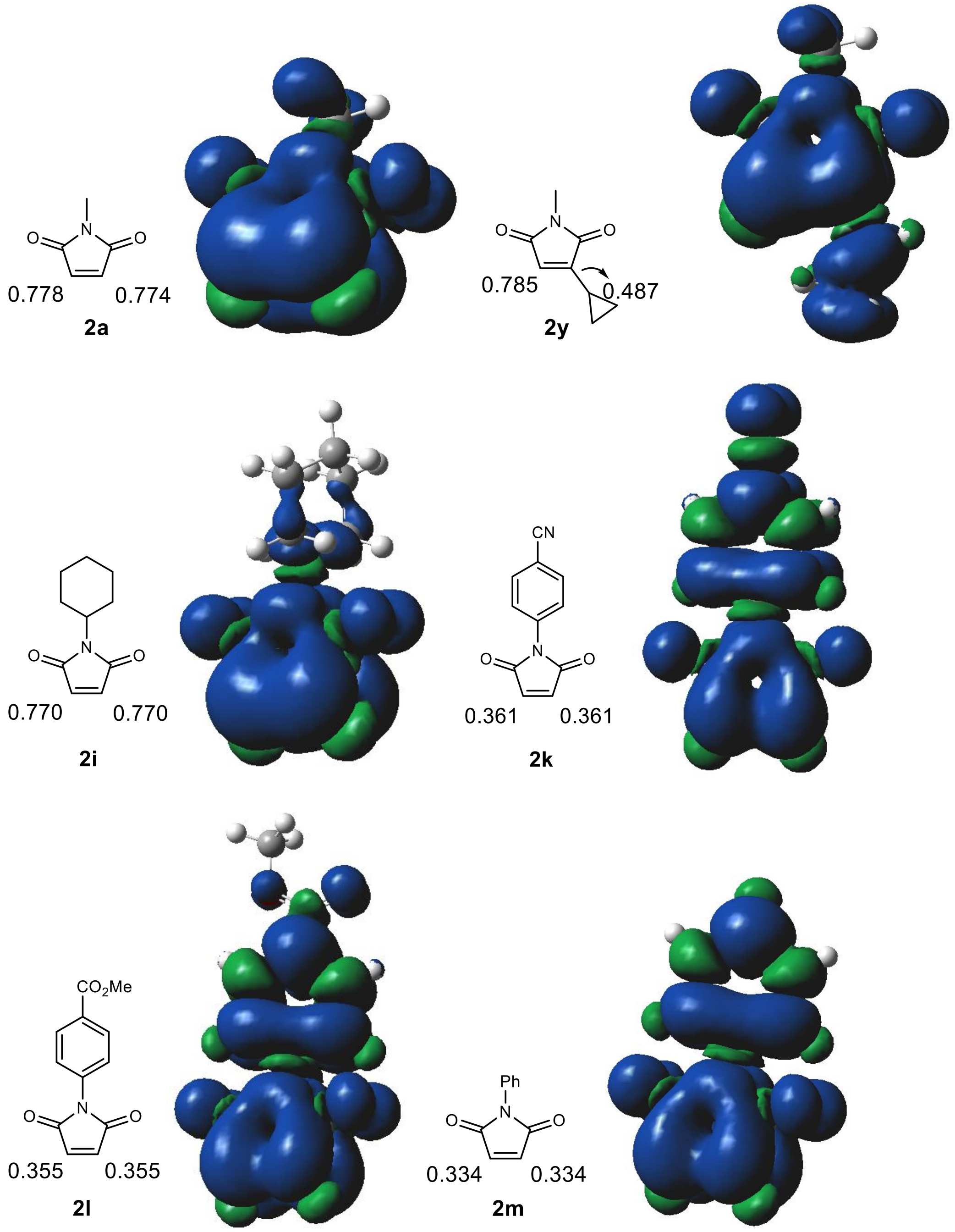
0.778
0.774
2a
0.785
0.487
2y
0.770
0.770
2i
CN
0.361
0.361
2k
CO_2Me
0.355
0.355
2l
Ph
0.334
0.334
2m

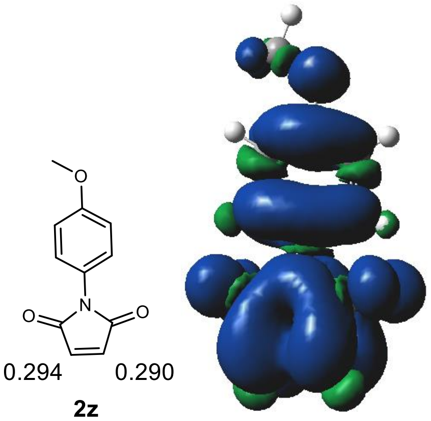

2z

Energies in hartree

Structure	Singlet State		Triplet State	
	E	G_{298}	E	G_{298}
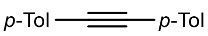	-617.768794	-617.567592	-617.671212	-617.477265
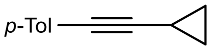	-464.182929	-464.024486	-464.072976	-463.922612
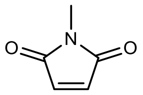	-398.623008	-398.559162	-398.529160	-398.470102
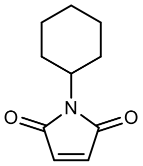	-593.889076	-593.709595	-593.794556	-593.620074
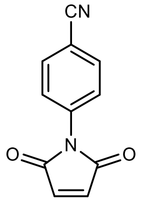	-682.471101	-682.363005	-682.380502	-682.277224
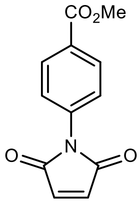	-818.086099	-817.939406	-817.99760	-817.853773
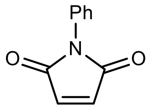	-590.258357	-590.146511	-590.173830	-590.066166

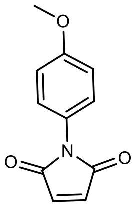	-704.753168	-704.612223	-704.680946	-704.542843
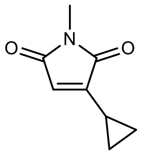	-515.275984	-515.155177	-515.193553	-515.076346

**Structure**	**E**	**G298**
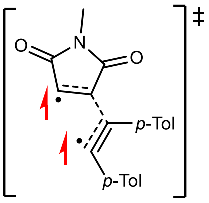	-1016.310304	-1016.026162
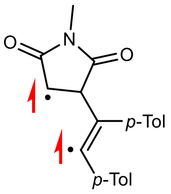	-1016.351762	-1016.064558
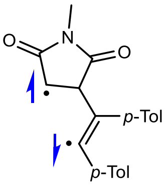	-1016.353003	-1016.064934
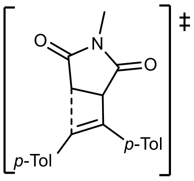	-1016.350217	-1016.065151
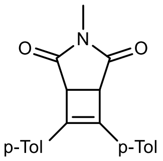	-1,016.445626	-1016.151950
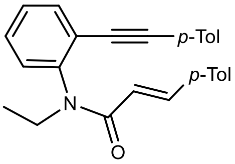	-1,173.307973	-1172.928150
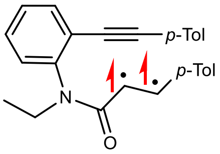	-1,173.230942	-1172.850034

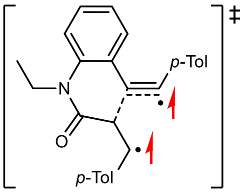	-1,173.218107	-1172.840691
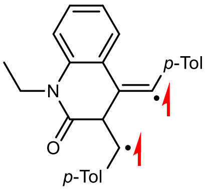	-1,173.264459	-1172.887191
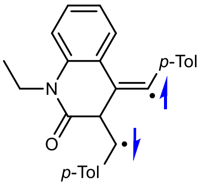	-1,173.264931	-1172.884844
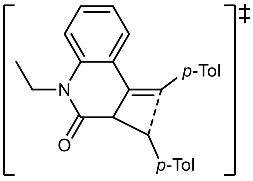	-1173.262014	-1172.883138
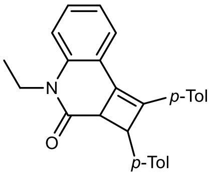	-1,173.340234	-1172.952621
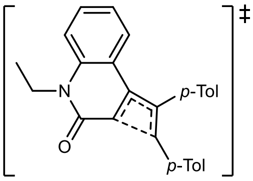	-1173.304587	-1172.918873
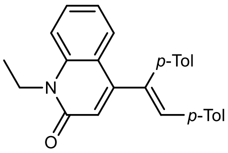	-1,173.36647	-1172.982681
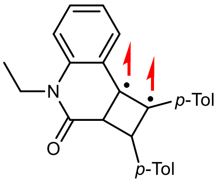	-1173.268737	-1172.887011
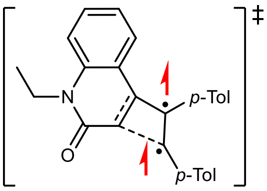	-1,173.243236	-1172.862334

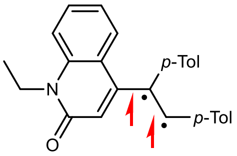	-1173.304252	-1172.923265
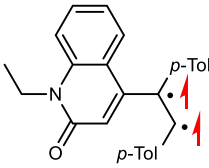	-1173.30499	-1172.923796
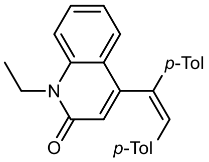	-1173.369365	-1172.985362
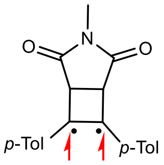	-1016.372528	-1016.084883
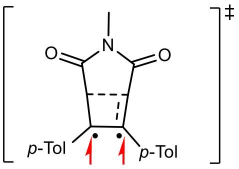	-1,016.325645	-1016.037862
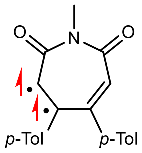	-1016.365684	-1016.077252

Confirmation of the open-shell singlet geometries

Structure	S^2	Open shell singlet molecular orbitals	
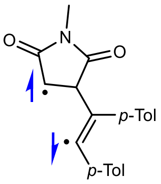	0.9436	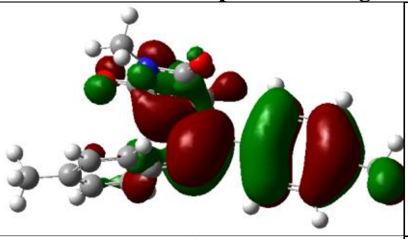	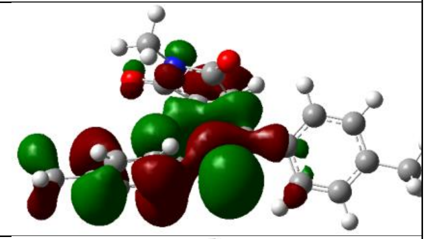
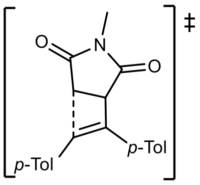	1.0426	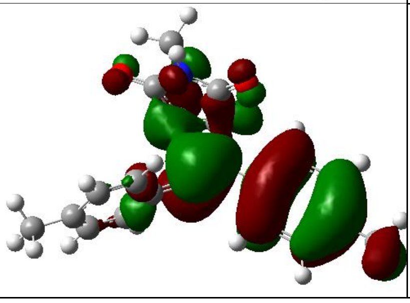	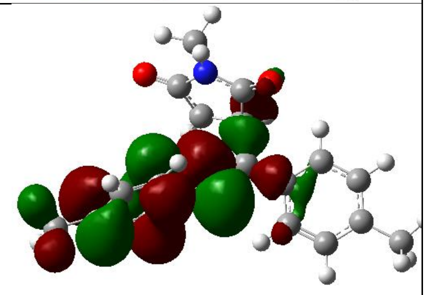

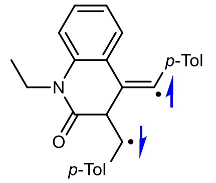	1.086<br>3	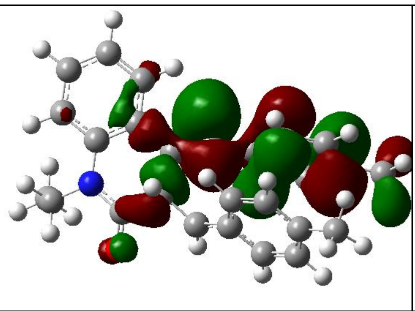	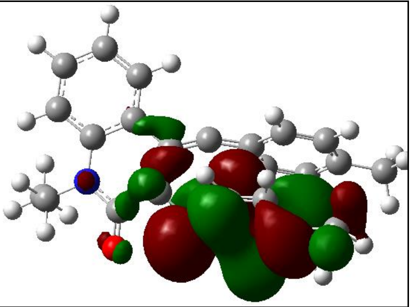
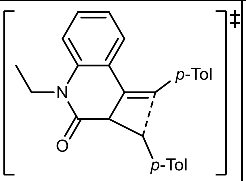	0.877<br>4	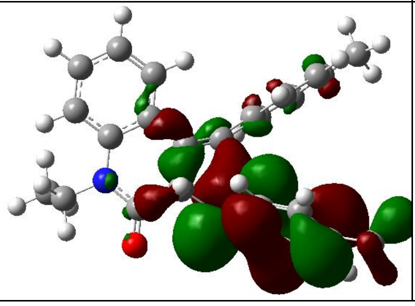	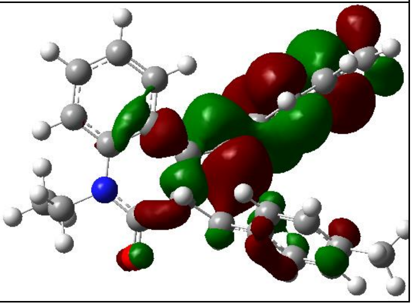

Stereochemical assignments by 1D NOE Experiments

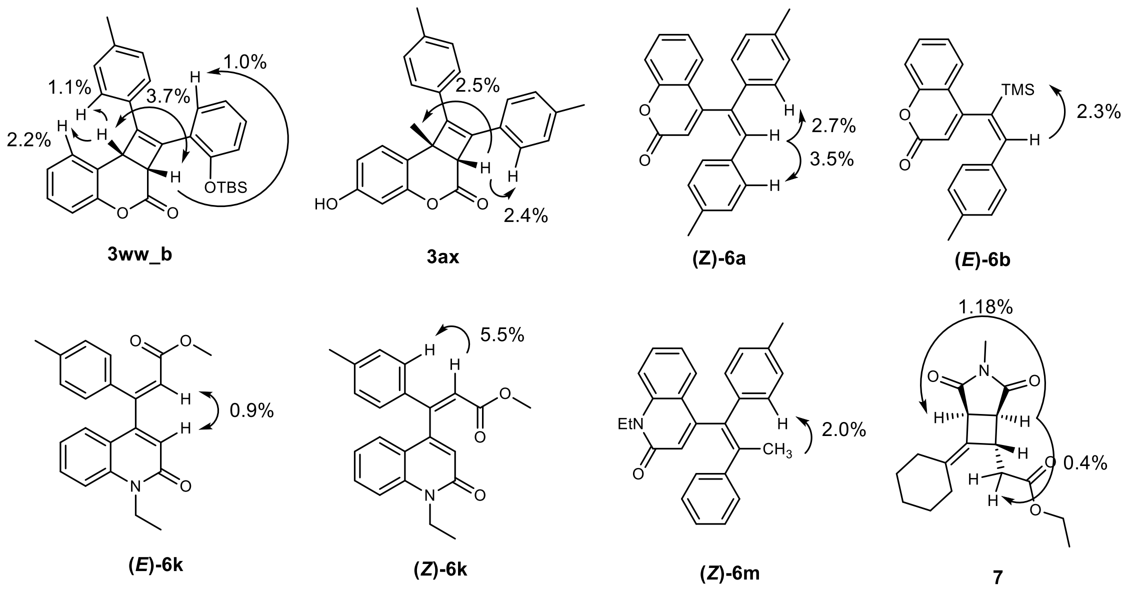

Supplementary Figure 13. Stereochemical Assignments by 1D NOE Experiments.

NMR spectra

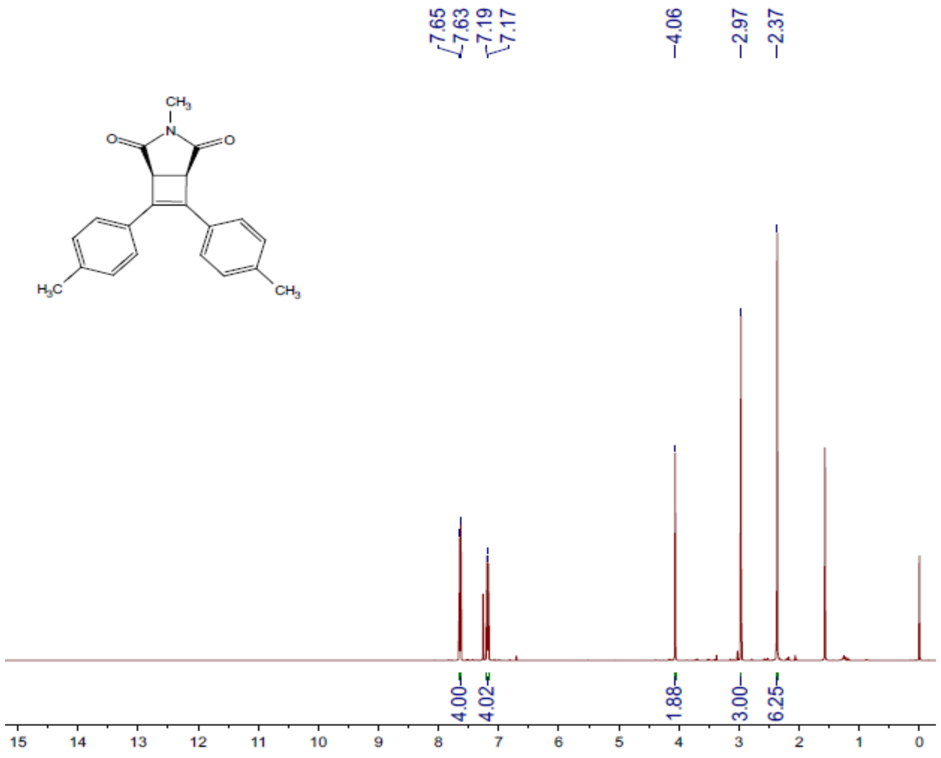

Supplementary Figure 14. ^{1}H NMR (400 MHz, $CDCl_3$) spectra of 3-methyl-6,7-di-*p*-tolyl-3-azabicyclo[3.2.0]hept-6-ene-2,4-dione (**3aa**)

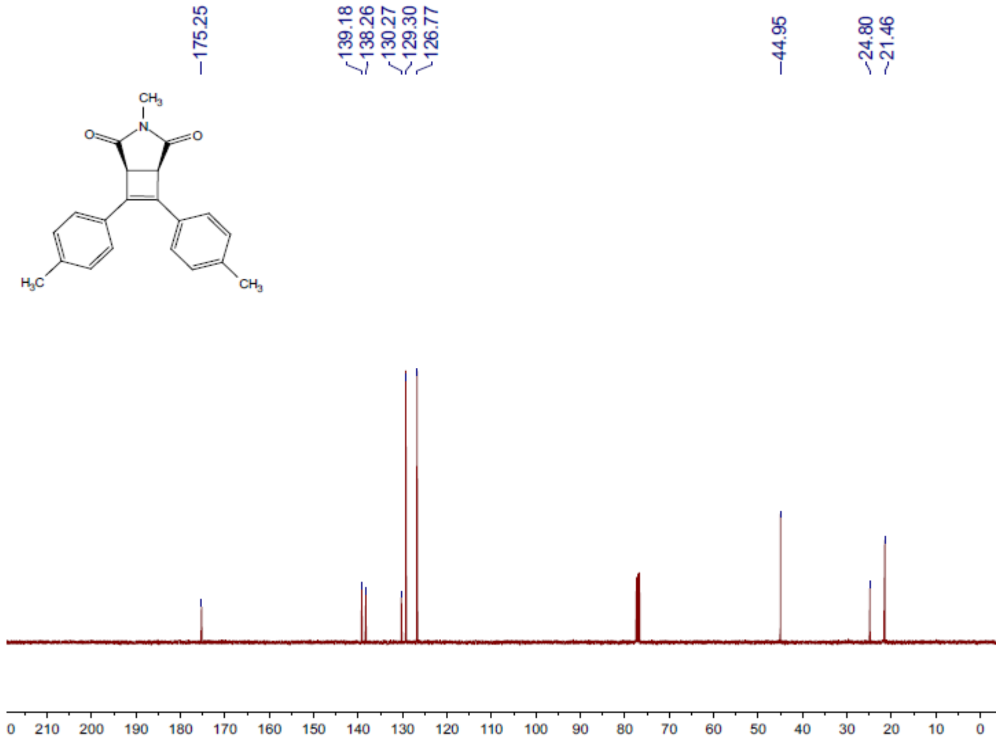

Supplementary Figure 15. ^{13}C NMR (100 MHz, $CDCl_3$) spectra of 3-methyl-6,7-di-*p*-tolyl-3-azabicyclo[3.2.0]hept-6-ene-2,4-dione (**3aa**)

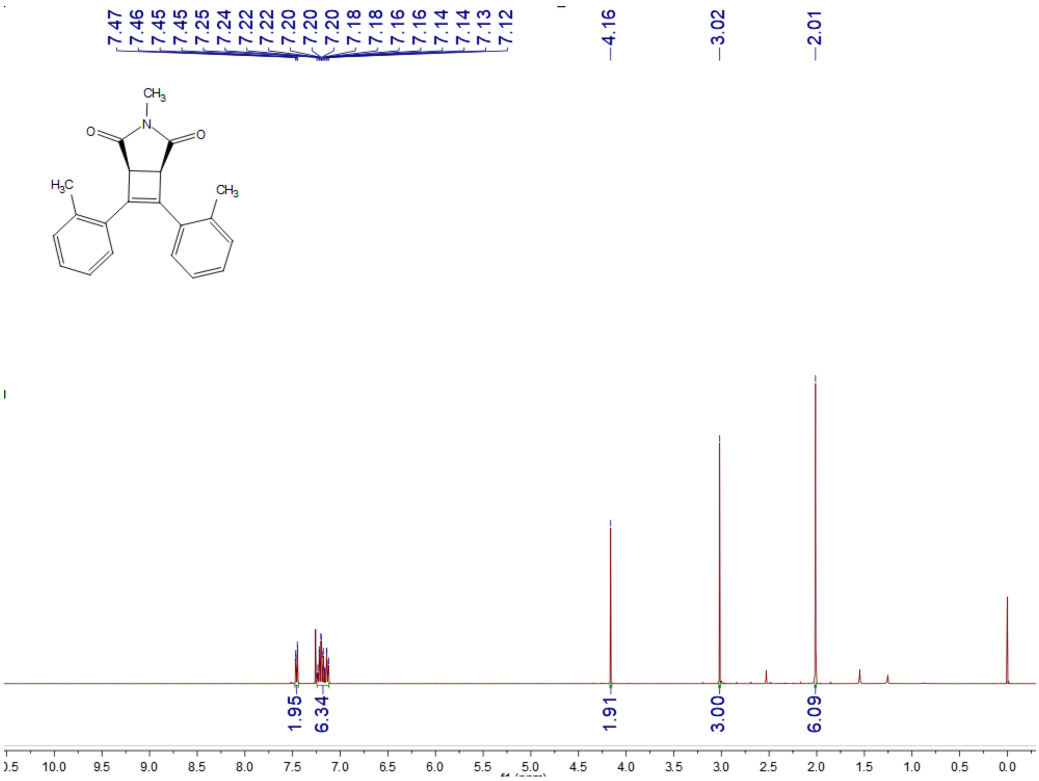

Supplementary Figure 16. ^{1}H NMR (400 MHz, $CDCl_3$) spectra of 3-methyl-6,7-di-*o*-tolyl-3-azabicyclo[3.2.0]hept-6-ene-2,4-dione (**3ba**)

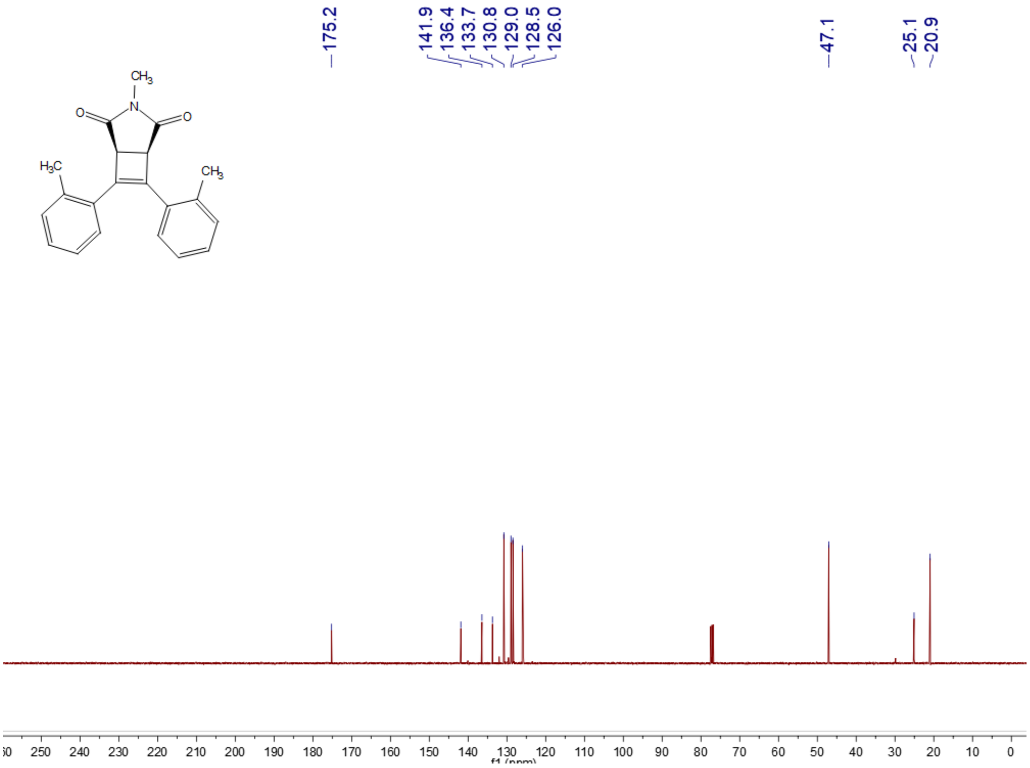

Supplementary Figure 17. ^{13}C NMR (100 MHz, $CDCl_3$) spectra of 3-methyl-6,7-di-*o*-tolyl-3-azabicyclo[3.2.0]hept-6-ene-2,4-dione (**3ba**)

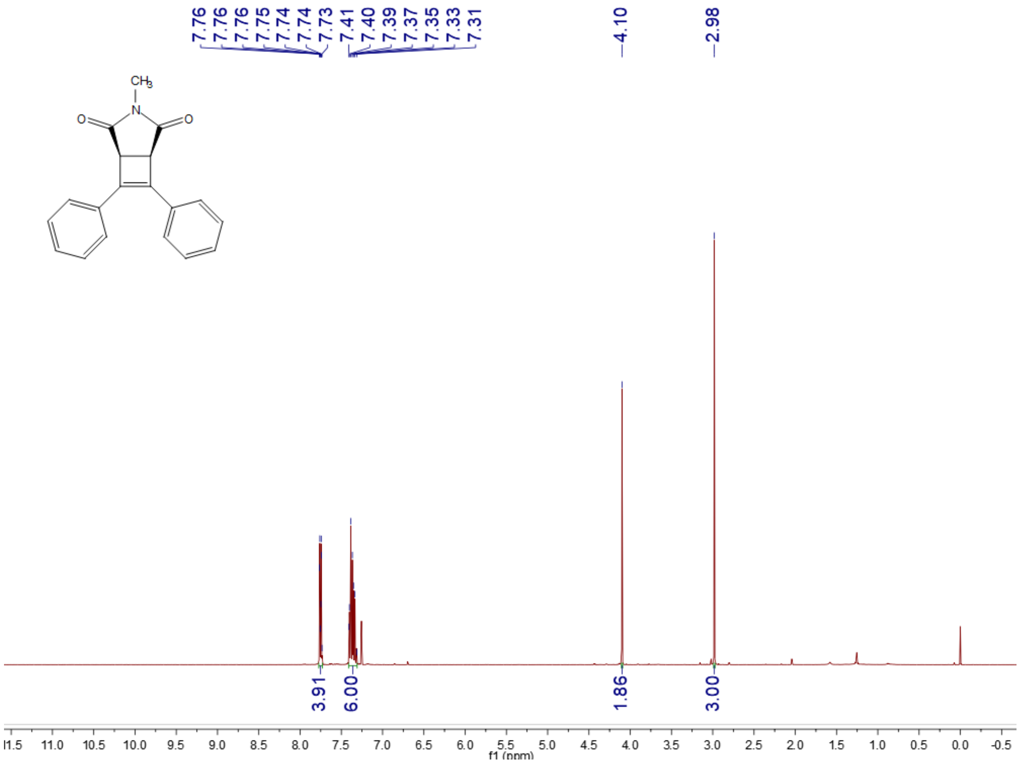

Supplementary Figure 18. ^{1}H NMR (400 MHz, $CDCl_3$) spectra of 3-methyl-6,7-diphenyl-3-azabicyclo[3.2.0]hept-6-ene-2,4-dione (**3ca**)

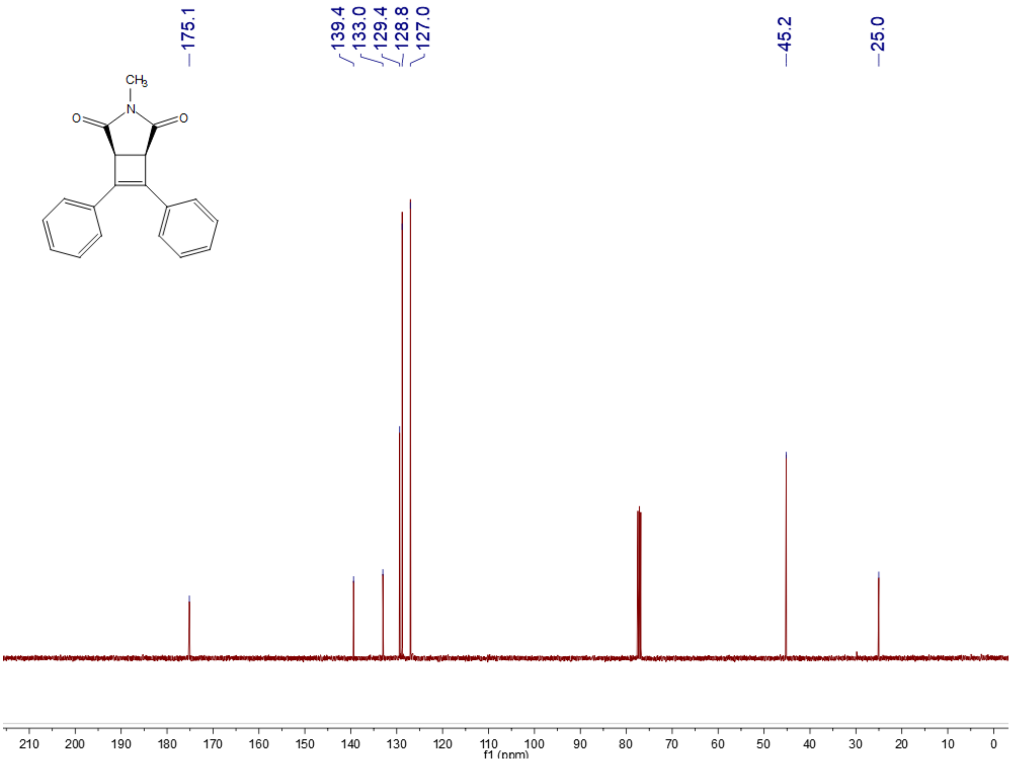

Supplementary Figure 19. ^{13}C NMR (100 MHz, $CDCl_3$) spectra of 3-methyl-6,7-diphenyl-3-azabicyclo[3.2.0]hept-6-ene-2,4-dione (**3ca**)

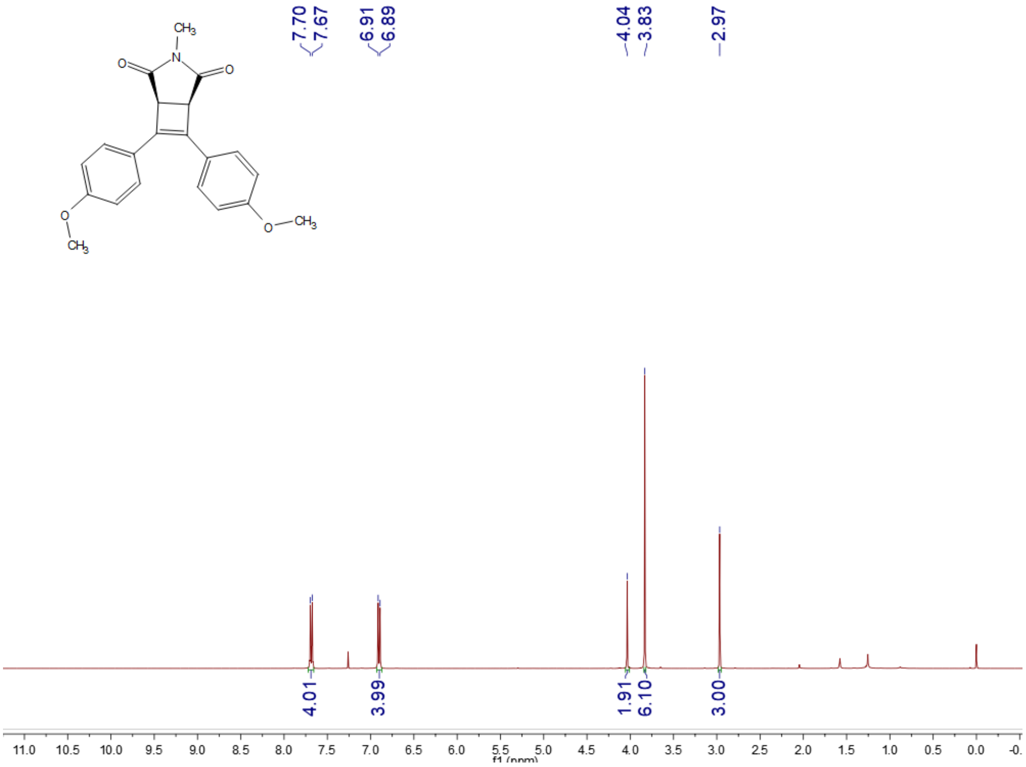

Supplementary Figure 20. ^{1}H NMR (400 MHz, $CDCl_3$) spectra of 6,7-bis(4-methoxyphenyl)-3-methyl-3-azabicyclo[3.2.0]hept-6-ene-2,4-dione (**3da**)

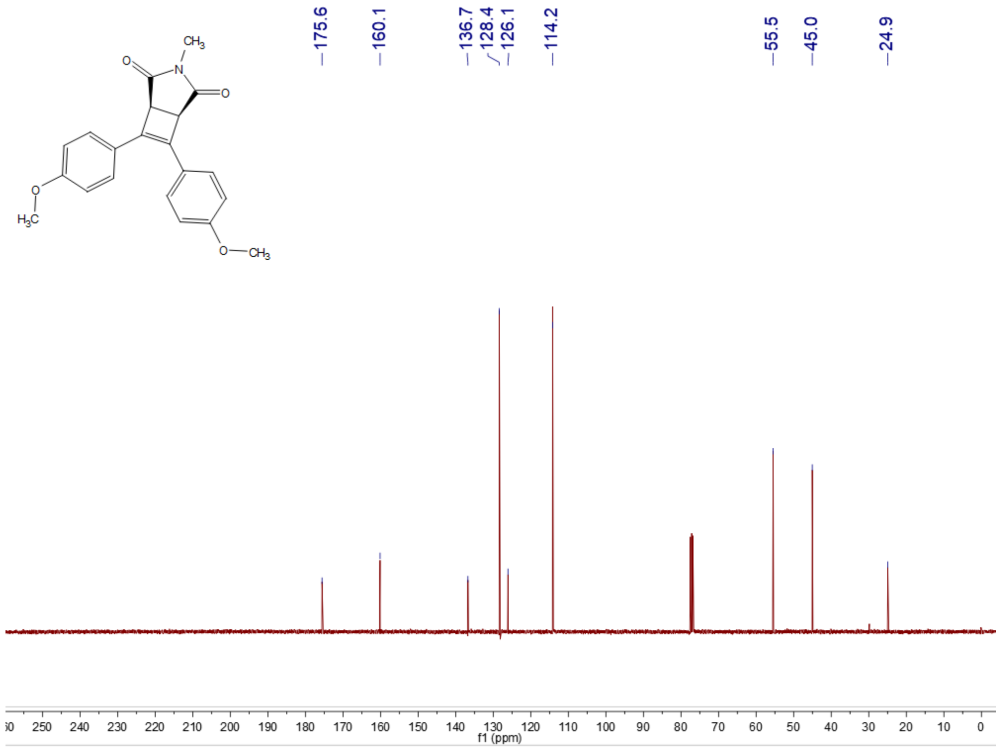

Supplementary Figure 21. ^{13}C NMR (100 MHz, $CDCl_3$) spectra of 6,7-bis(4-methoxyphenyl)-3-methyl-3-azabicyclo[3.2.0]hept-6-ene-2,4-dione (**3da**)

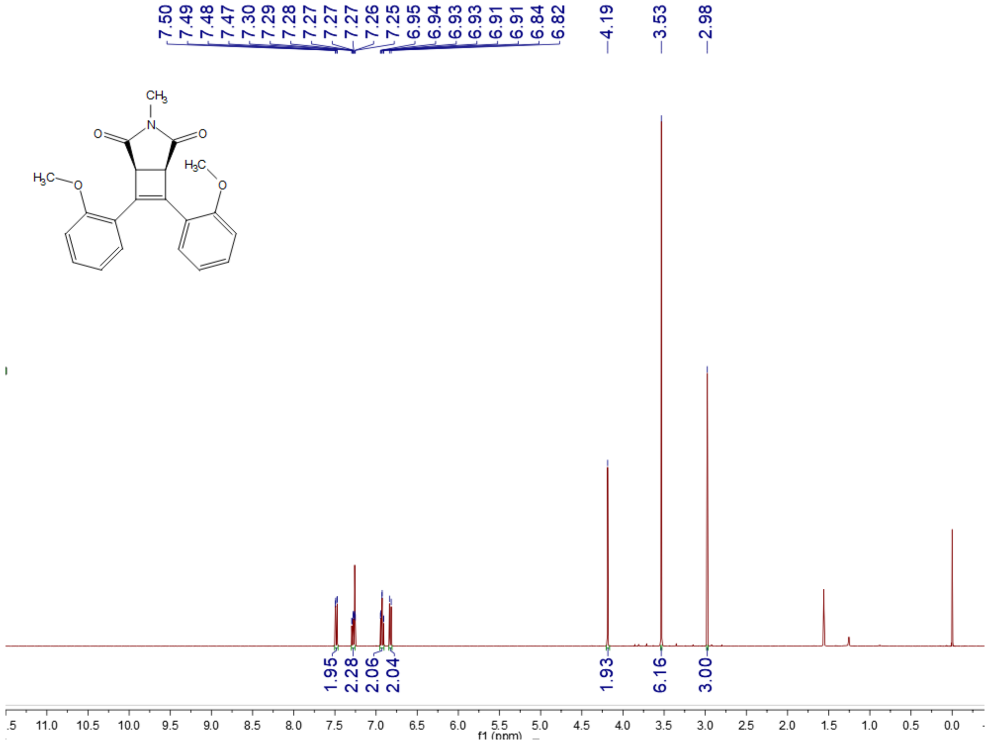

Supplementary Figure 22. ^{1}H NMR (400 MHz, $CDCl_3$) spectra of 6,7-bis(2-methoxyphenyl)-3-methyl-3-azabicyclo[3.2.0]hept-6-ene-2,4-dione (**3ea**)

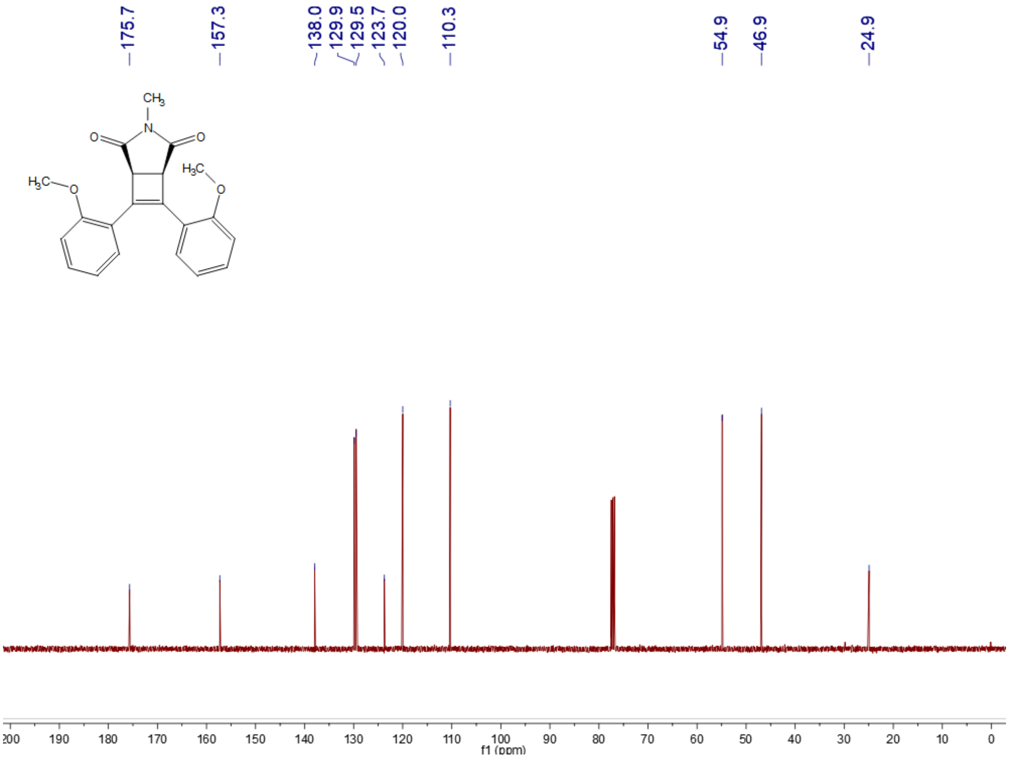

Supplementary Figure 23. ^{13}C NMR (100 MHz, $CDCl_3$) spectra of 6,7-bis(2-methoxyphenyl)-3-methyl-3-azabicyclo[3.2.0]hept-6-ene-2,4-dione (**3ea**)

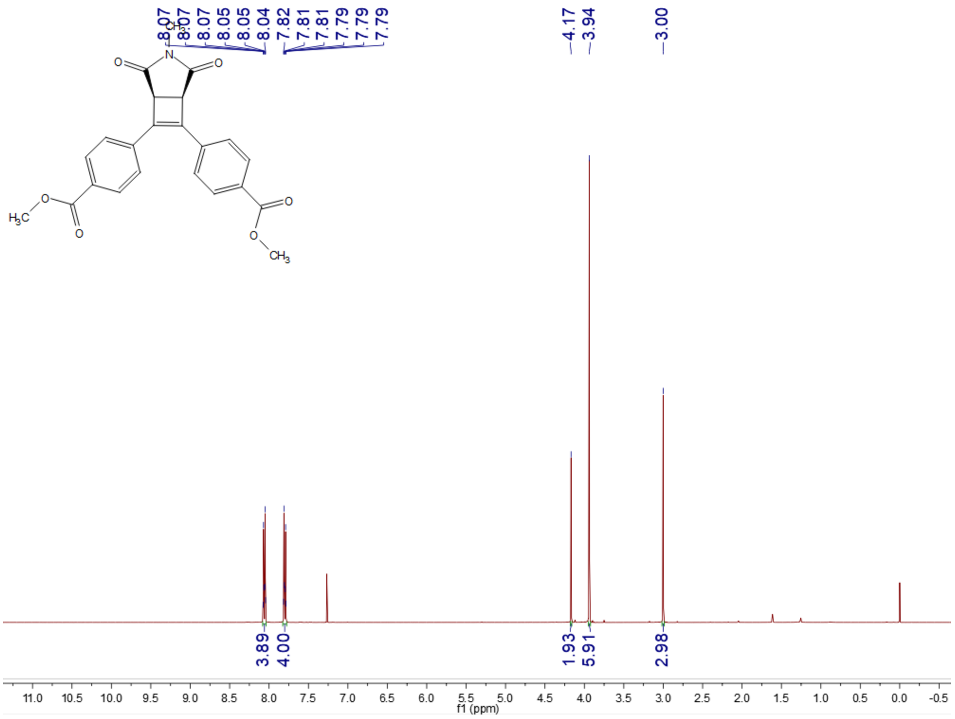

Supplementary Figure 24. ^{1}H NMR (400 MHz, $CDCl_3$) spectra of Dimethyl 4,4'-(3-methyl-2,4-dioxo-3-azabicyclo[3.2.0]hept-6-ene-6,7-diyl)dibenzoate (**3fa**)

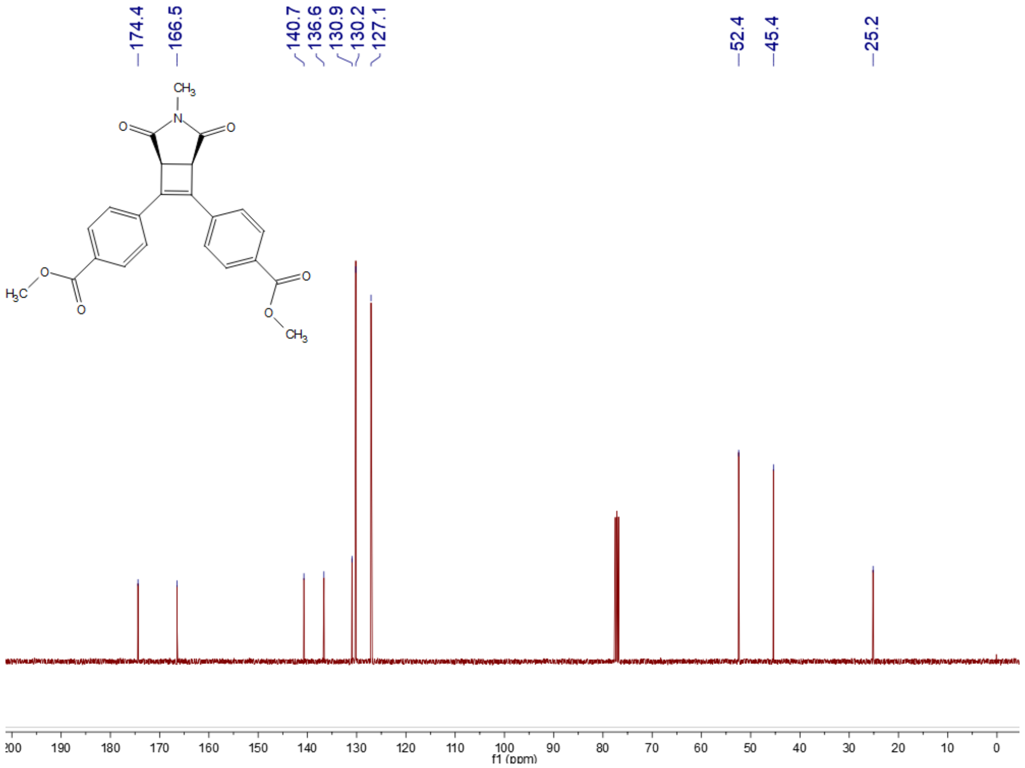

Supplementary Figure 25. ^{13}C NMR (100 MHz, $CDCl_3$) spectra of Dimethyl 4,4'-(3-methyl-2,4-dioxo-3-azabicyclo[3.2.0]hept-6-ene-6,7-diyl)dibenzoate (**3fa**)

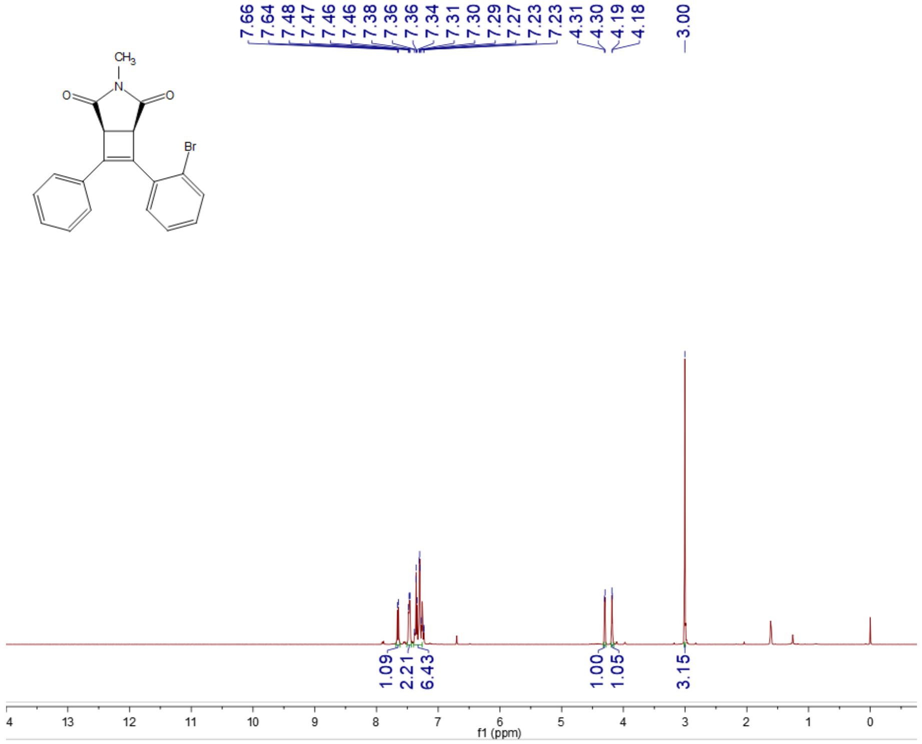

Supplementary Figure 26. ^{1}H NMR (400 MHz, $CDCl_3$) spectra of 6-(2-bromophenyl)-3-methyl-7-phenyl-3-azabicyclo[3.2.0]hept-6-ene-2,4-dione (**3ga**)

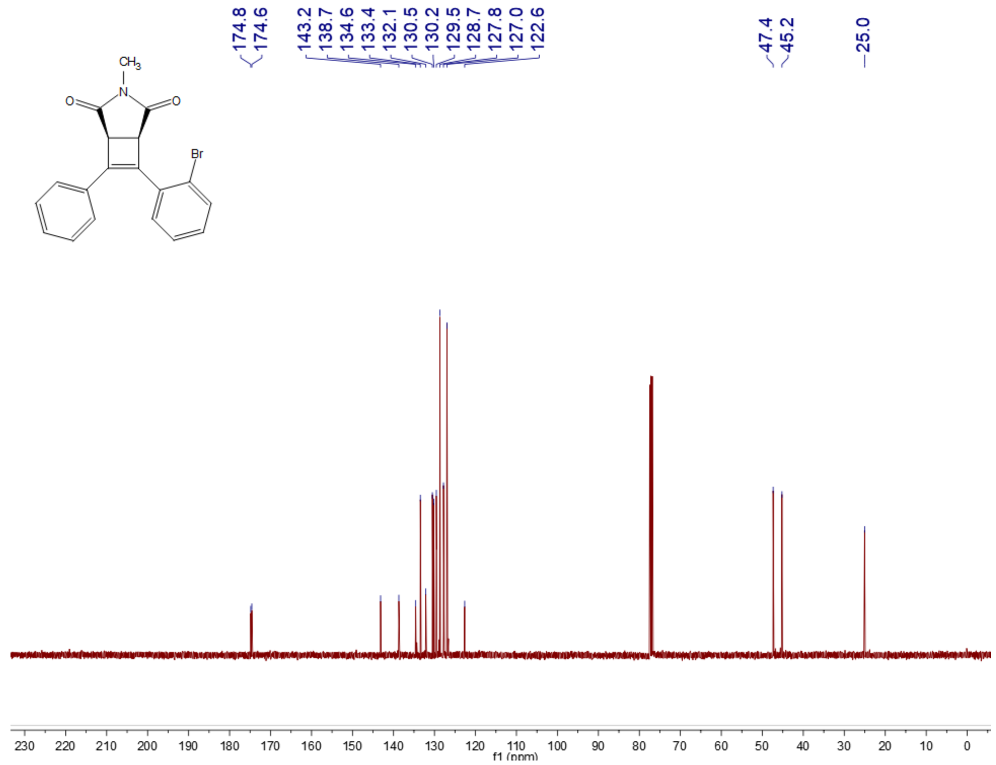

Supplementary Figure 27. ^{13}C NMR (100 MHz, $CDCl_3$) spectra of 6-(2-bromophenyl)-3-methyl-7-phenyl-3-azabicyclo[3.2.0]hept-6-ene-2,4-dione (**3ga**)

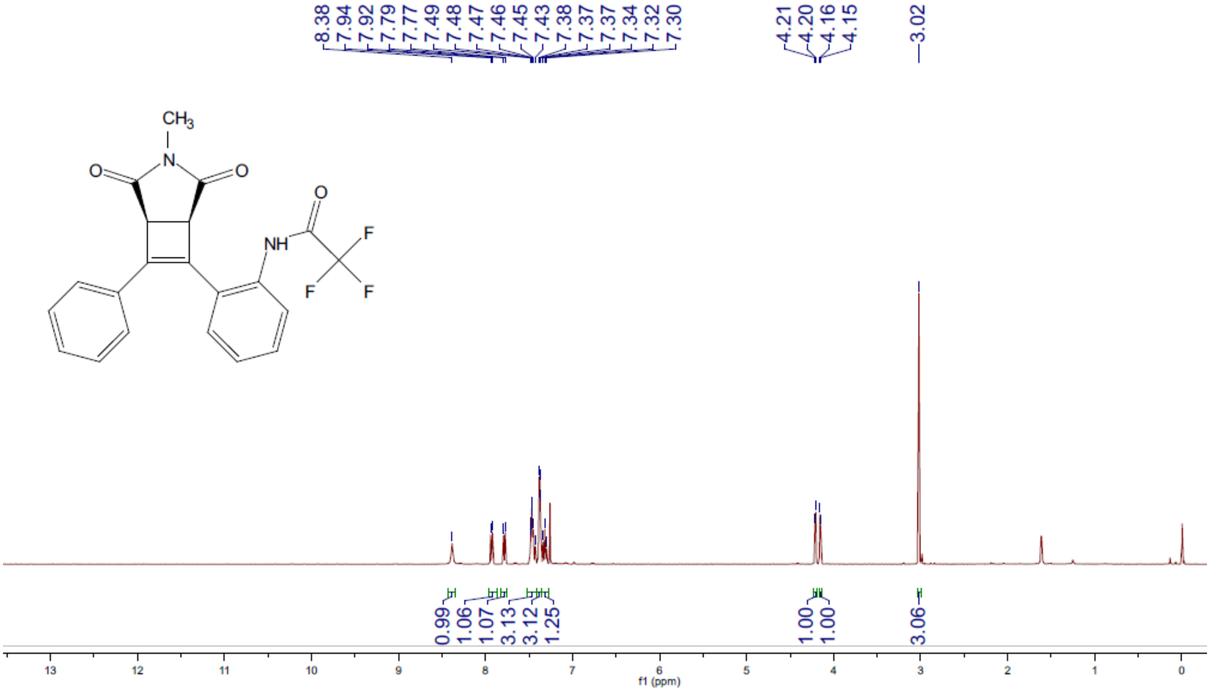

Supplementary Figure 28. ^{1}H NMR (400 MHz, $CDCl_3$) spectra of 3-methyl-6-phenyl-7-(2-((trifluoromethoxy)amino)phenyl)-3-azabicyclo[3.2.0]hept-6-ene-2,4-dione (**3ha**)

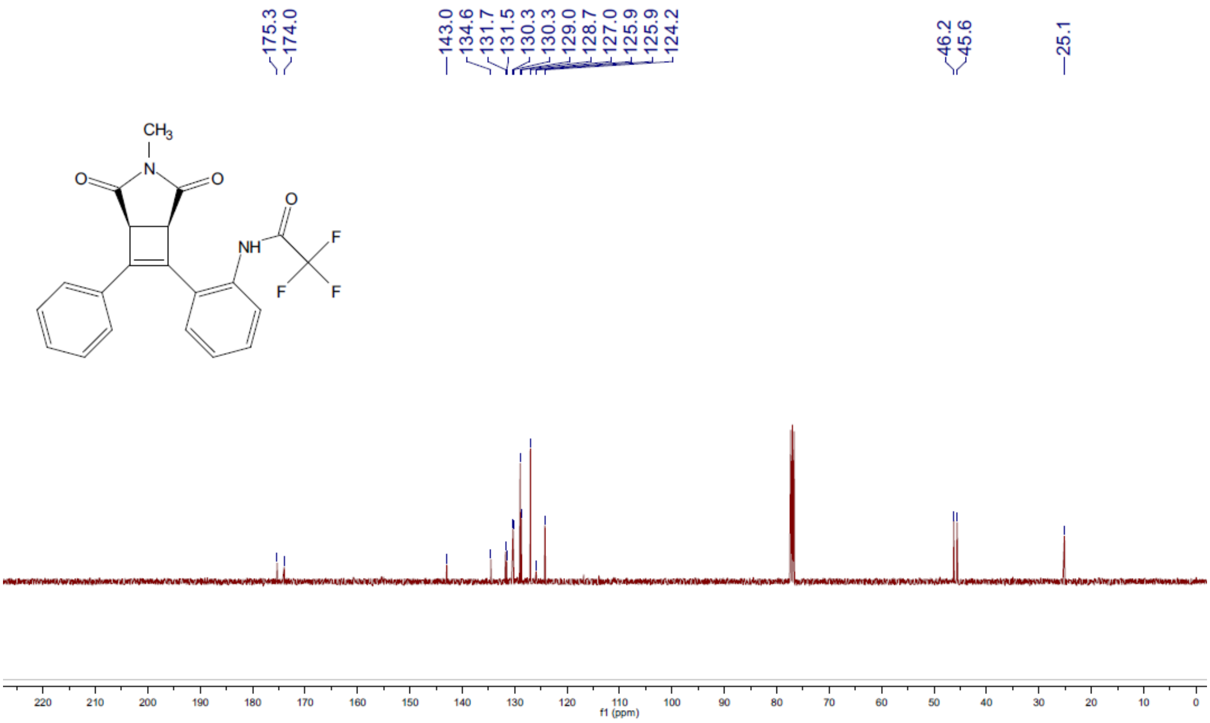

Supplementary Figure 29. ^{13}C NMR (100 MHz, $CDCl_3$) spectra of 3-methyl-6-phenyl-7-(2-((trifluoromethoxy)amino)phenyl)-3-azabicyclo[3.2.0]hept-6-ene-2,4-dione (**3ha**)

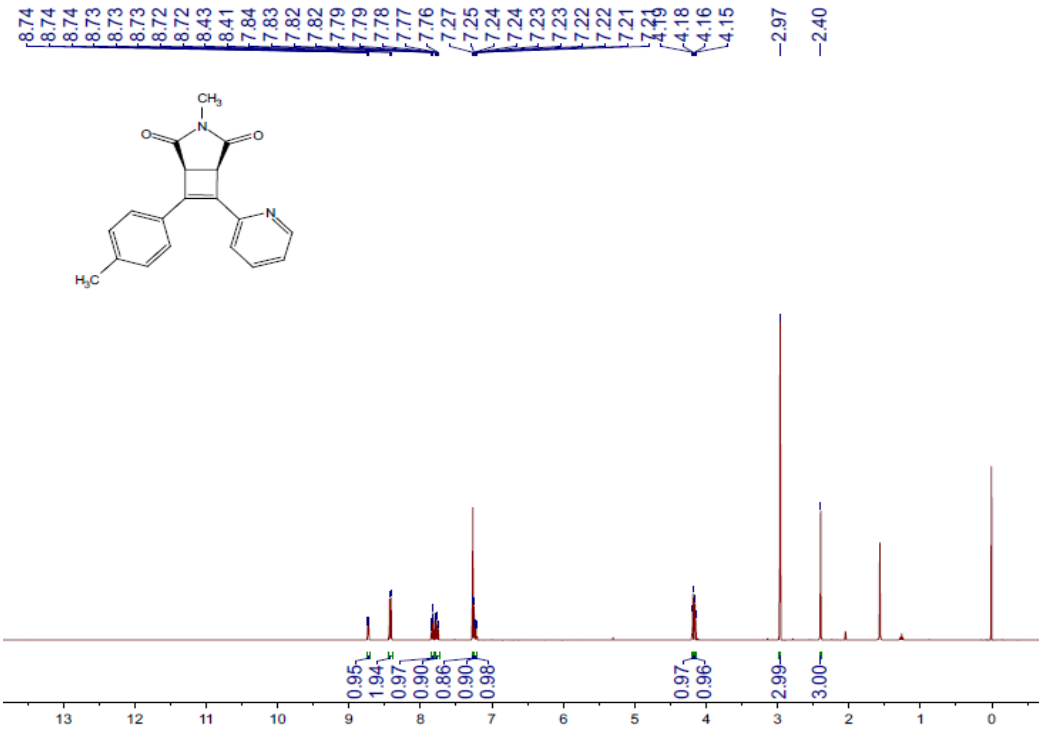

Supplementary Figure 30. ^{1}H NMR (400 MHz, $CDCl_3$) spectra of 3-methyl-6-(pyridin-2-yl)-7-(*p*-tolyl)-3-azabicyclo[3.2.0]hept-6-ene-2,4-dione (**3ia**)

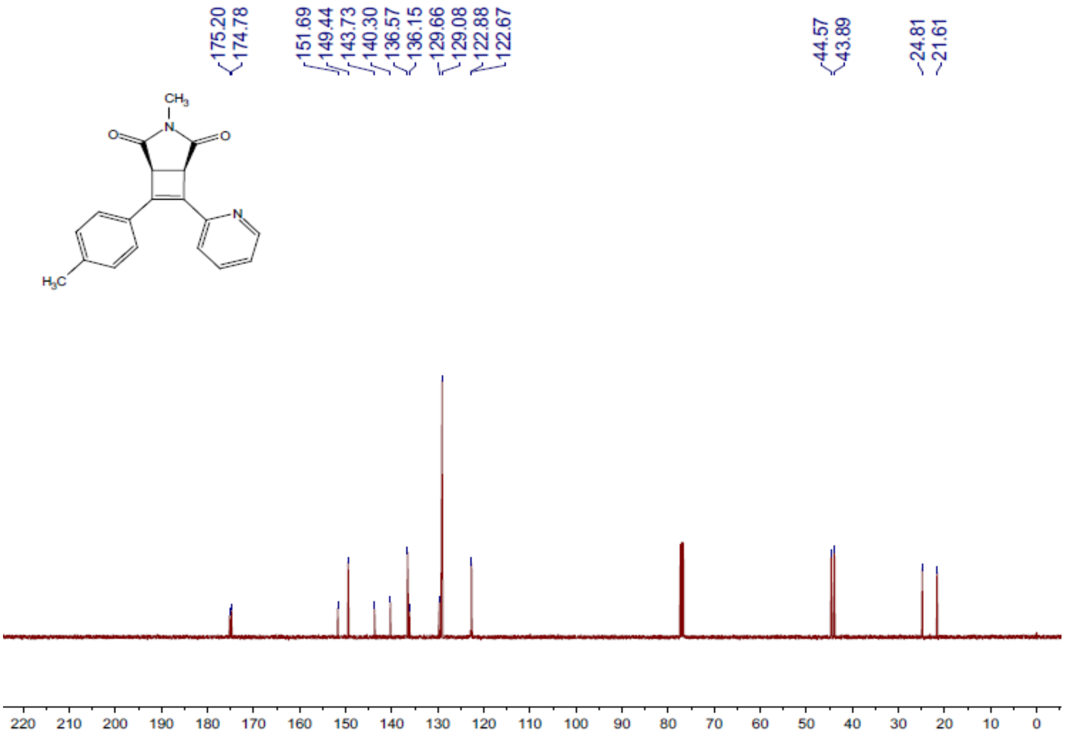

Supplementary Figure 31. ^{13}C NMR (100 MHz, $CDCl_3$) spectra of 3-methyl-6-(pyridin-2-yl)-7-(*p*-tolyl)-3-azabicyclo[3.2.0]hept-6-ene-2,4-dione (**3ia**)

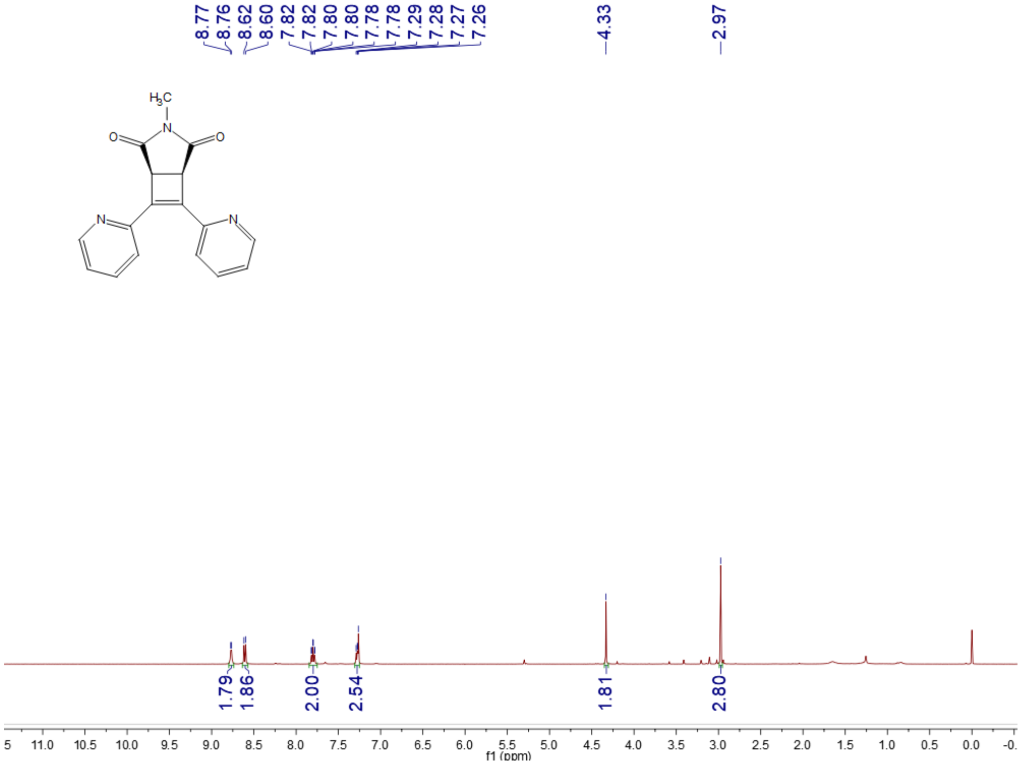

Supplementary Figure 32. ^{1}H NMR (400 MHz, $CDCl_3$) spectra of 3-methyl-6,7-di(pyridin-2-yl)-3-azabicyclo[3.2.0]hept-6-ene-2,4-dione (**3ja**)

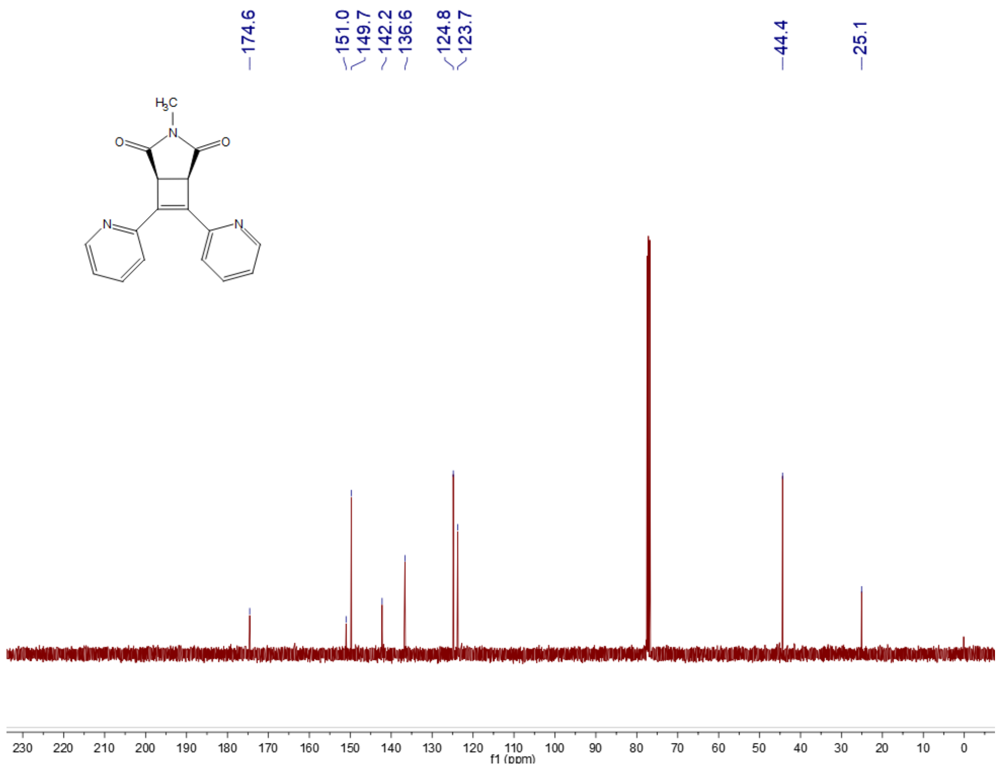

Supplementary Figure 33. ^{13}C NMR (100 MHz, $CDCl_3$) spectra of 3-methyl-6,7-di(pyridin-2-yl)-3-azabicyclo[3.2.0]hept-6-ene-2,4-dione (**3ja**)

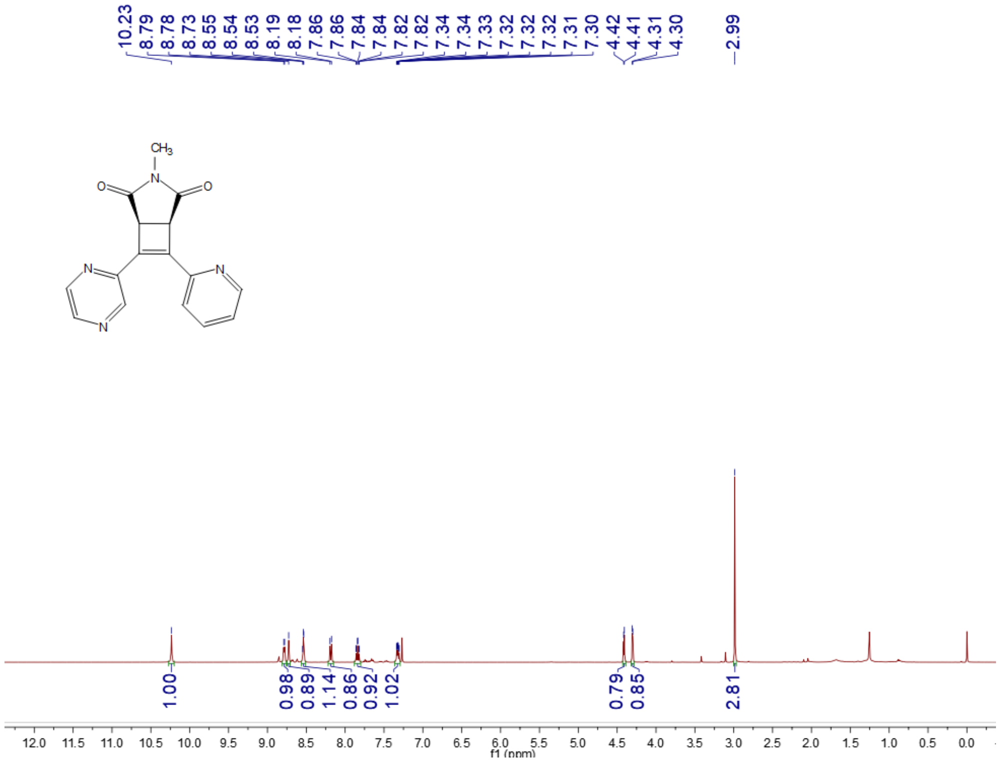

Supplementary Figure 34. ^{1}H NMR (400 MHz, $CDCl_3$) spectra of 3-methyl-6-(pyrazin-2-yl)-7-(pyridin-2-yl)-3-azabicyclo[3.2.0]hept-6-ene-2,4-dione (**3ka**)

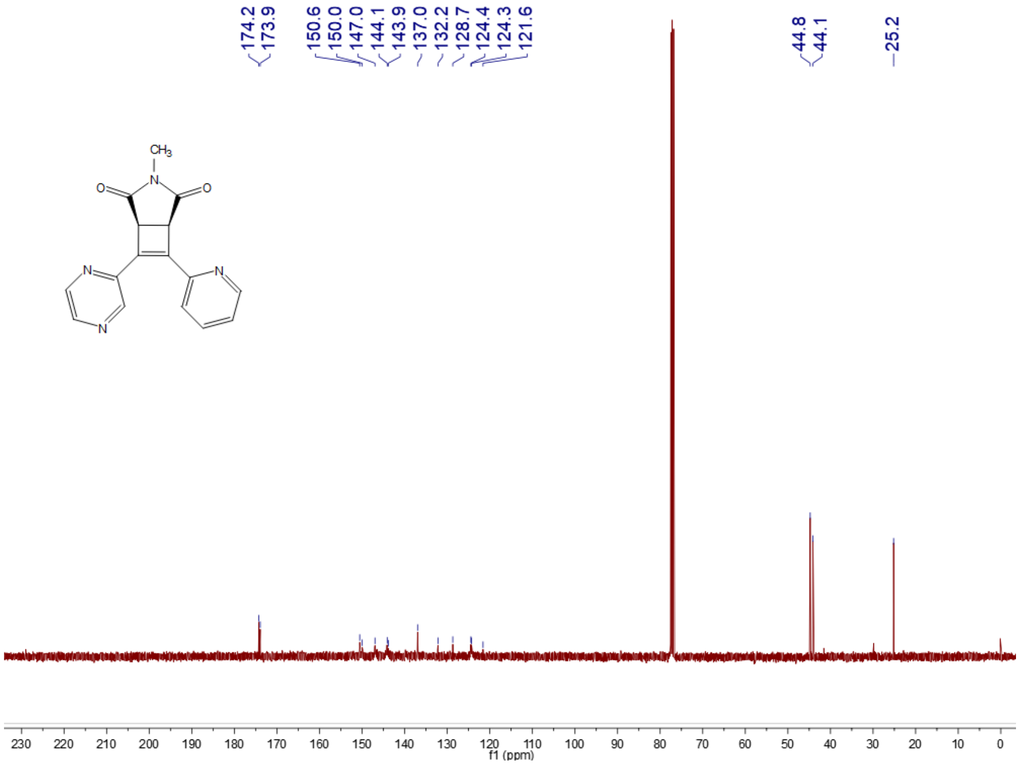

Supplementary Figure 35. ^{13}C NMR (100 MHz, $CDCl_3$) spectra of 3-methyl-6-(pyrazin-2-yl)-7-(pyridin-2-yl)-3-azabicyclo[3.2.0]hept-6-ene-2,4-dione (**3ka**)

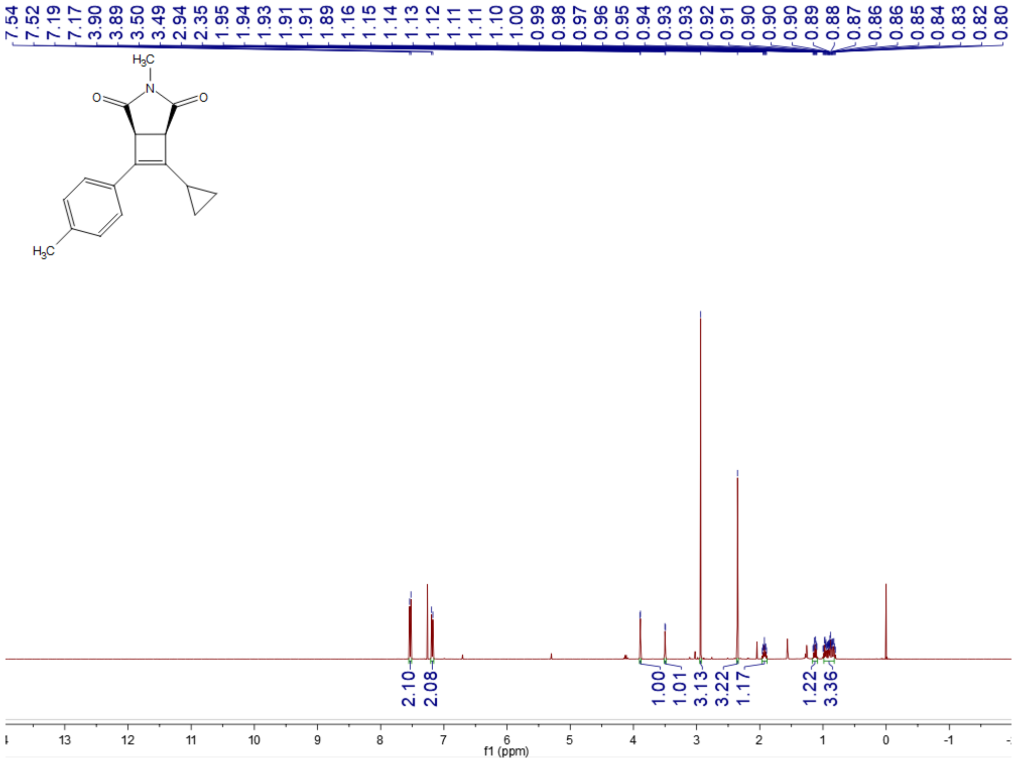

Supplementary Figure 36. ^{1}H NMR (400 MHz, $CDCl_3$) spectra of 6-cyclopropyl-3-methyl-7-(*p*-tolyl)-3-azabicyclo[3.2.0]hept-6-ene-2,4-dione (**3la**)

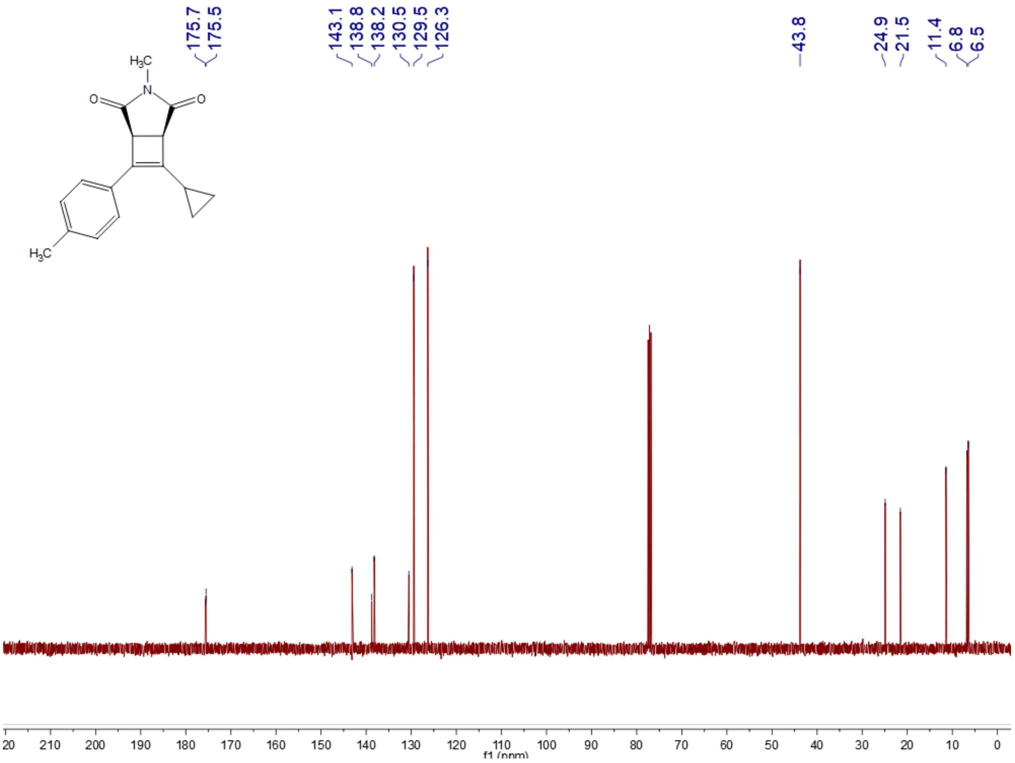

Supplementary Figure 37. ^{13}C NMR (100 MHz, $CDCl_3$) spectra of 6-cyclopropyl-3-methyl-7-(*p*-tolyl)-3-azabicyclo[3.2.0]hept-6-ene-2,4-dione (**3la**)

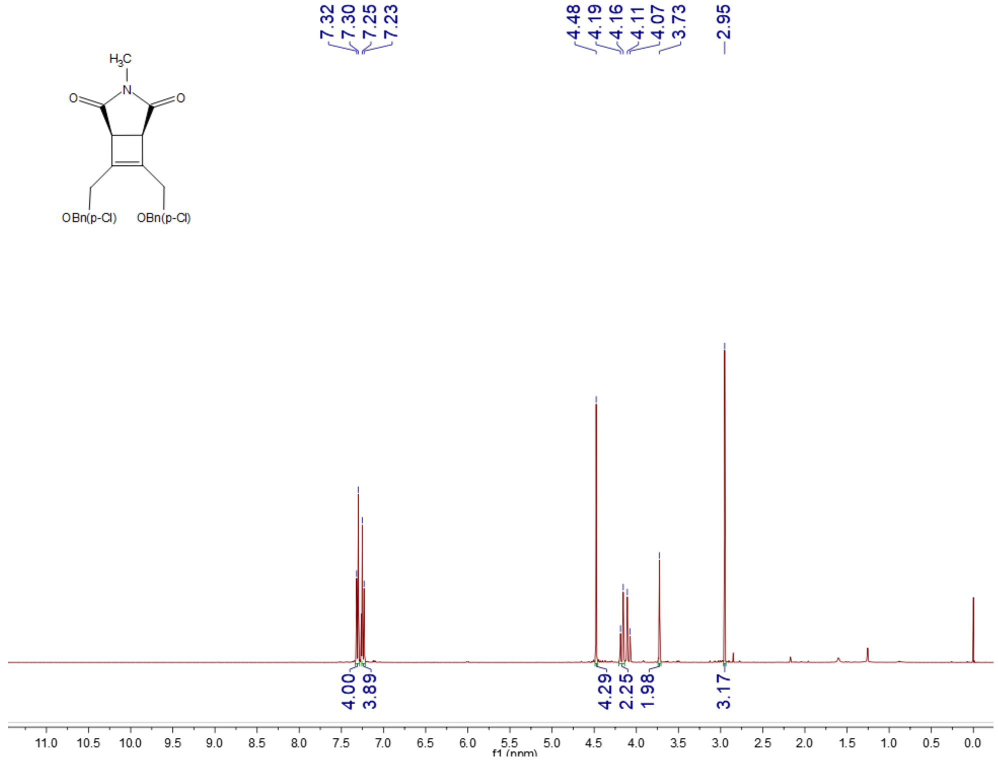

Supplementary Figure 38. ^{1}H NMR (400 MHz, $CDCl_3$) spectra of 6,7-bis(((4-chlorobenzyl)oxy)methyl)-3-methyl-3-azabicyclo[3.2.0]hept-6-ene-2,4-dione (**3ma**)

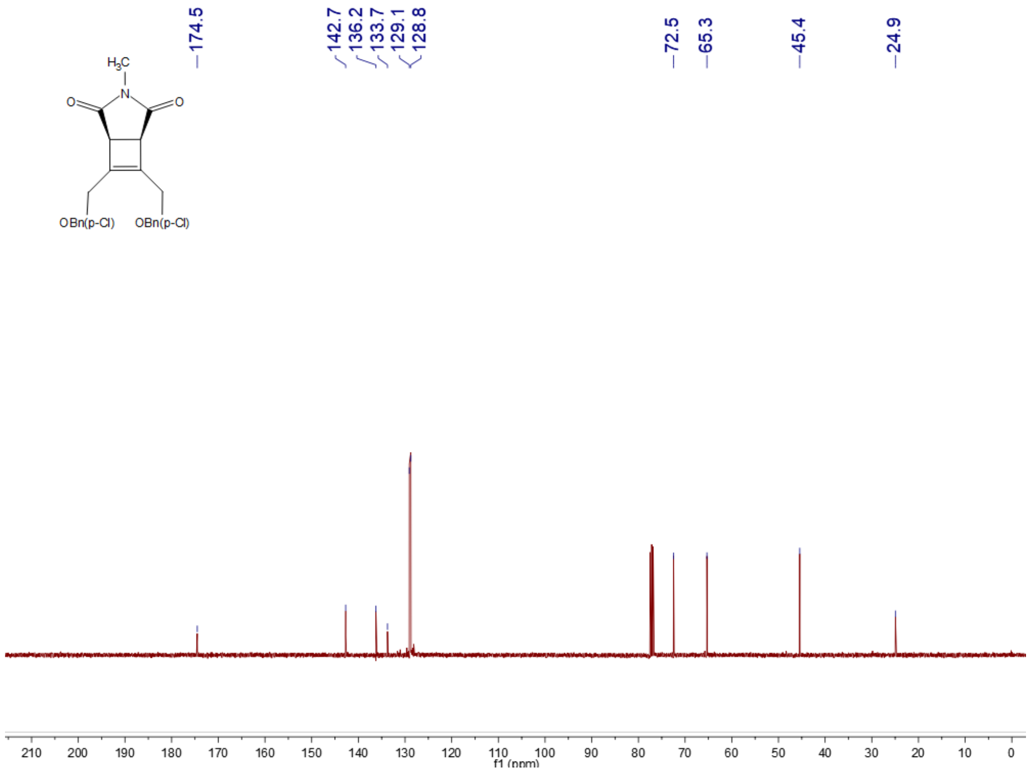

Supplementary Figure 39. ^{13}C NMR (100 MHz, $CDCl_3$) spectra of 6,7-bis(((4-chlorobenzyl)oxy)methyl)-3-methyl-3-azabicyclo[3.2.0]hept-6-ene-2,4-dione (**3ma**)

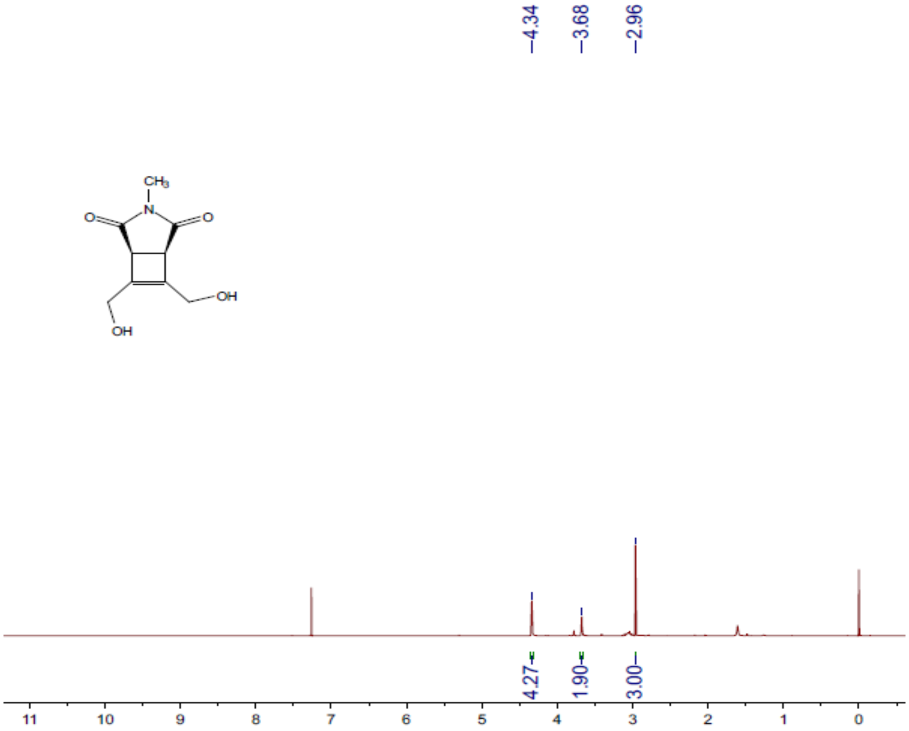

Supplementary Figure 40. ^{1}H NMR (400 MHz, $CDCl_3$) spectra of 6,7-bis(hydroxymethyl)-3-methyl-3-azabicyclo[3.2.0]hept-6-ene-2,4-dione (**3na**)

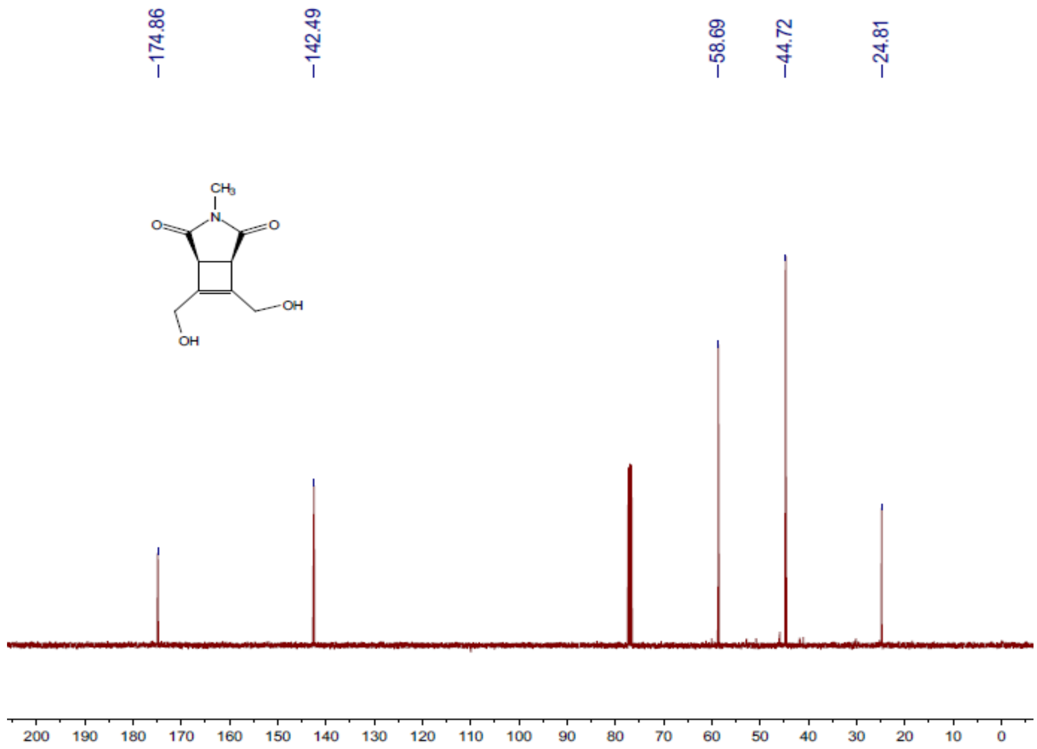

Supplementary Figure 41. ^{13}C NMR (100 MHz, $CDCl_3$) spectra of 6,7-bis(hydroxymethyl)-3-methyl-3-azabicyclo[3.2.0]hept-6-ene-2,4-dione (**3na**)

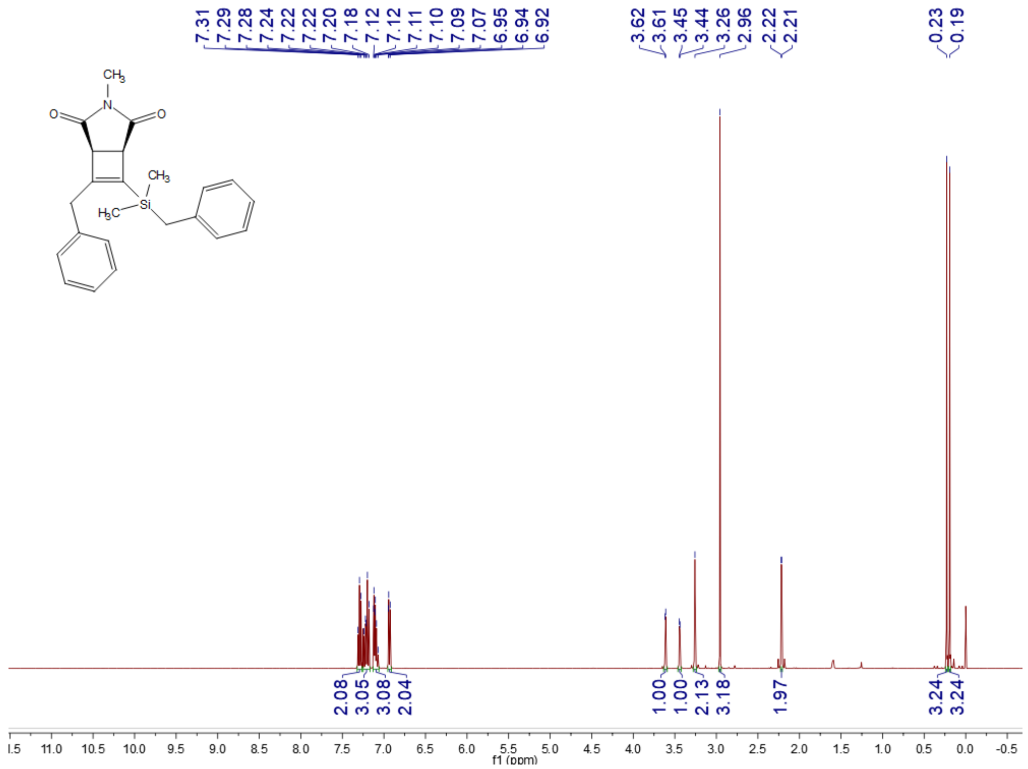

Supplementary Figure 42. ^{1}H NMR (400 MHz, $CDCl_3$) spectra of 6-benzyl-7-(benzyldimethylsilyl)-3-methyl-3-azabicyclo[3.2.0]hept-6-ene-2,4-dione (**3oa**)

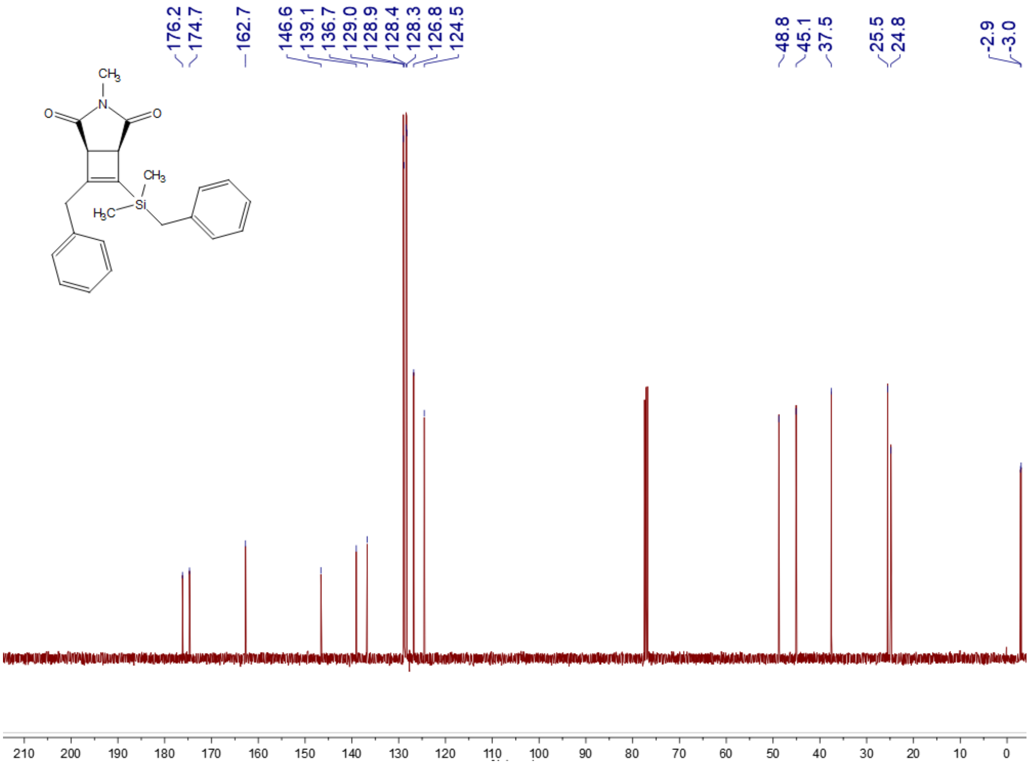

Supplementary Figure 43. ^{13}C NMR (100 MHz, $CDCl_3$) spectra of 6-benzyl-7-(benzyldimethylsilyl)-3-methyl-3-azabicyclo[3.2.0]hept-6-ene-2,4-dione (**3oa**)

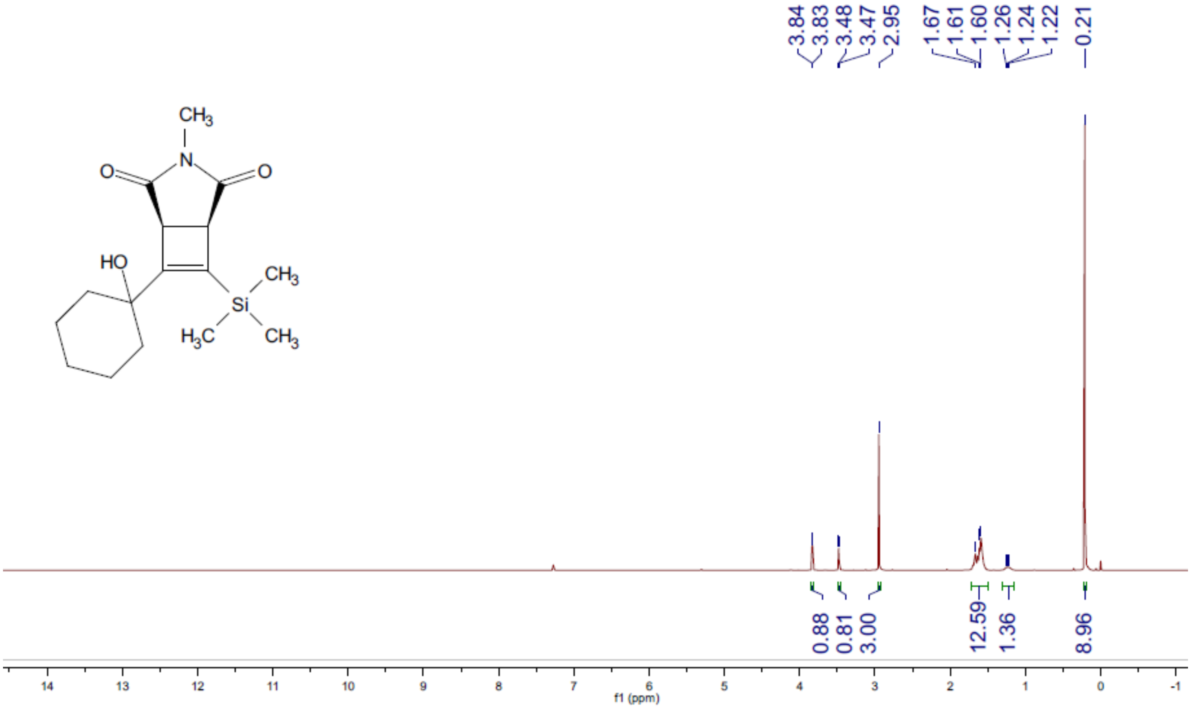

Supplementary Figure 44. ^{1}H NMR (400 MHz, $CDCl_3$) spectra of 6-(1-hydroxycyclohexyl)-3-methyl-7-(trimethylsilyl)-3-azabicyclo[3.2.0]hept-6-ene-2,4-dione (**3pa**)

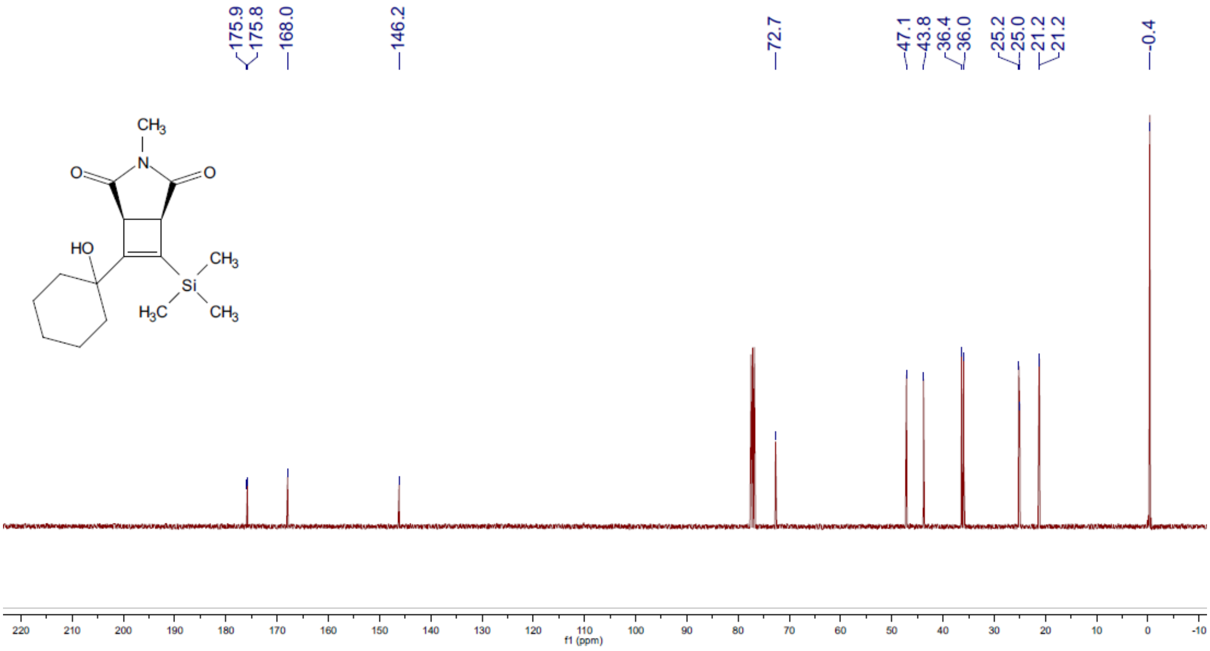

Supplementary Figure 45. ^{13}C NMR (100 MHz, $CDCl_3$) spectra of 6-(1-hydroxycyclohexyl)-3-methyl-7-(trimethylsilyl)-3-azabicyclo[3.2.0]hept-6-ene-2,4-dione (**3pa**)

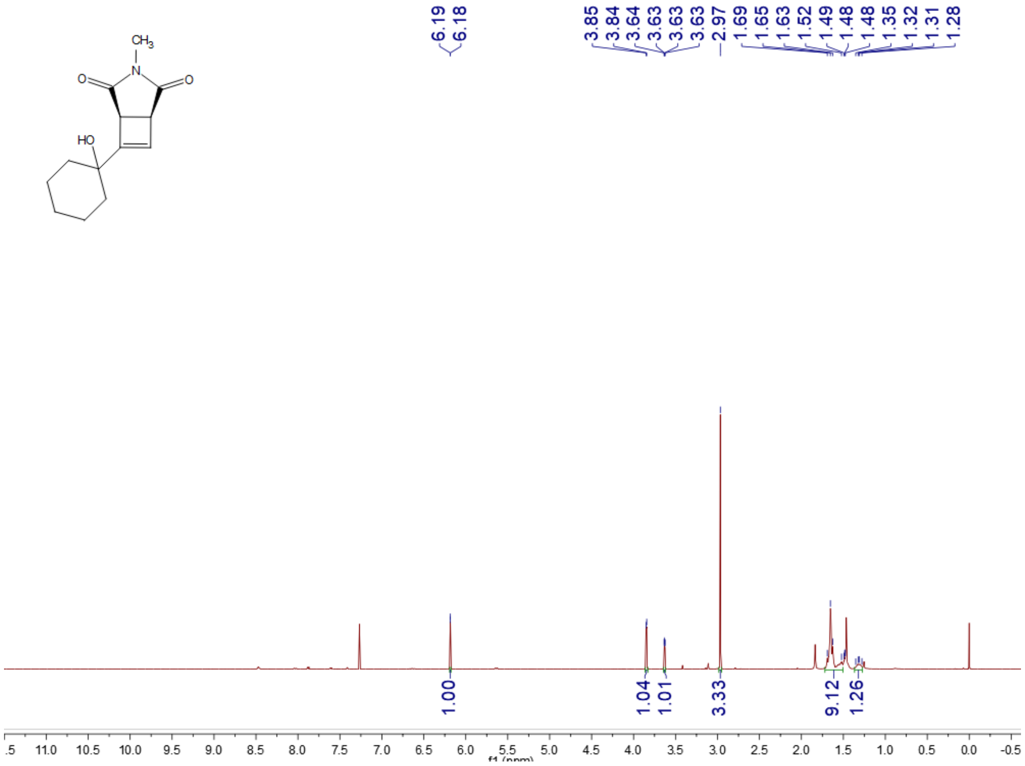

Supplementary Figure 46. ^{1}H NMR (400 MHz, $CDCl_3$) spectra of 6-(1-hydroxycyclohexyl)-3-methyl-3-azabicyclo[3.2.0]hept-6-ene-2,4-dione (**3qa**)

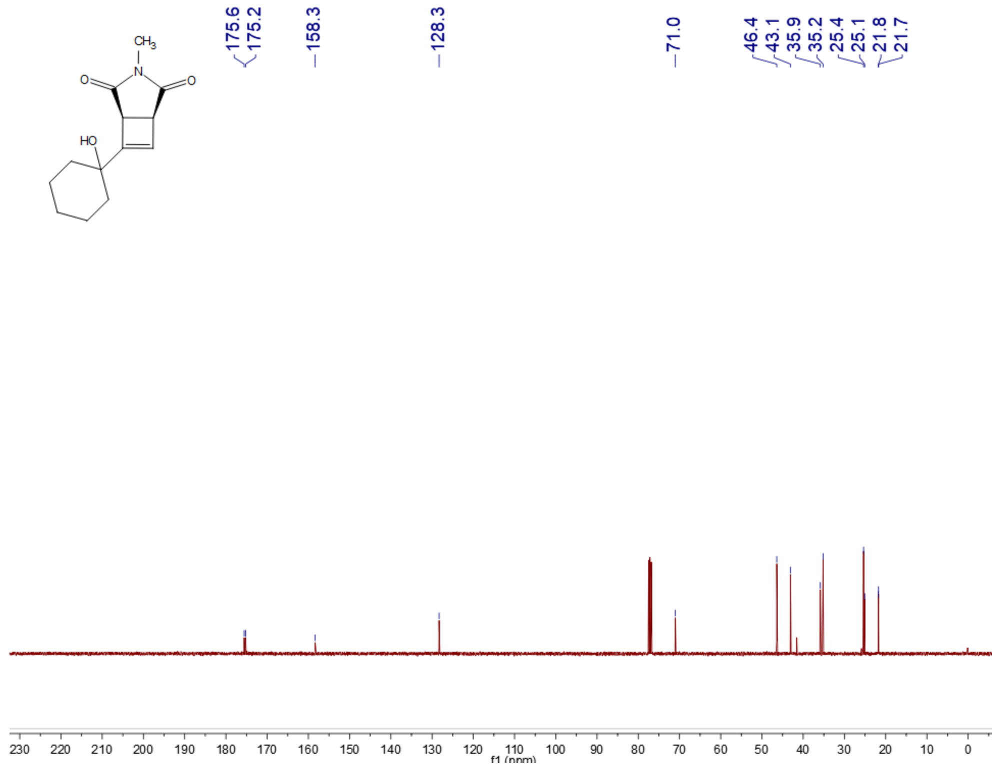

Supplementary Figure 47. ^{13}C NMR (100 MHz, $CDCl_3$) spectra of 6-(1-hydroxycyclohexyl)-3-methyl-3-azabicyclo[3.2.0]hept-6-ene-2,4-dione (**3qa**)

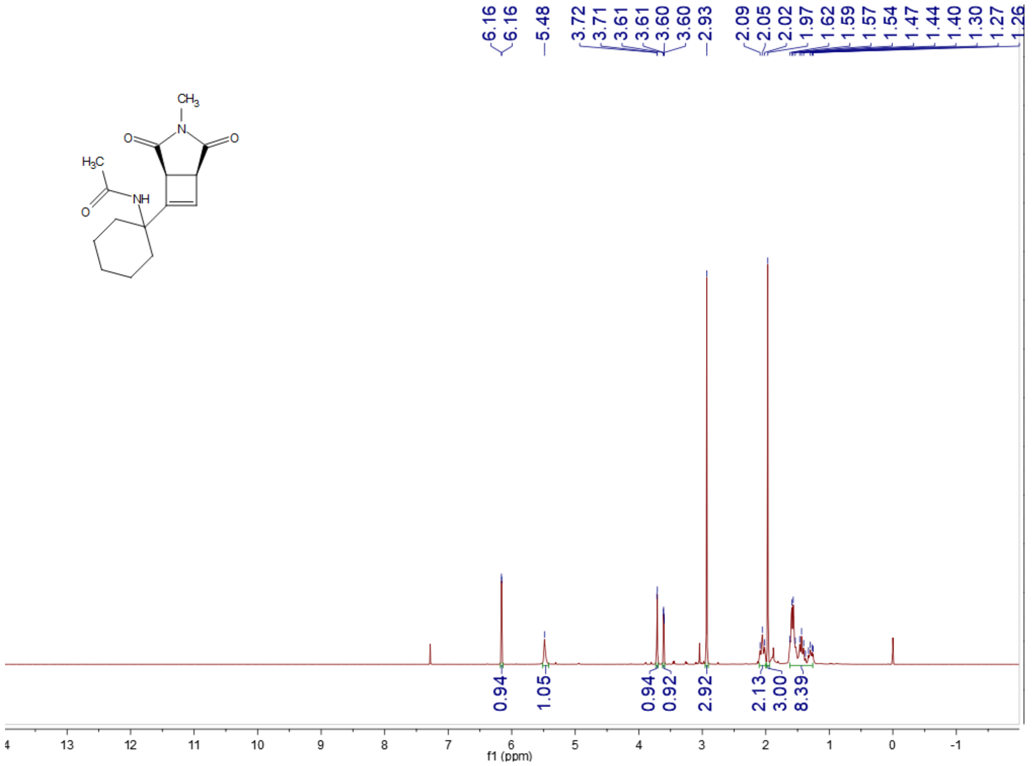

Supplementary Figure 48. ^{1}H NMR (400 MHz, $CDCl_3$) spectra of *N*-(1-(3-methyl-2,4-dioxo-3-azabicyclo[3.2.0]hept-6-en-6-yl)cyclohexyl)acetamide (**3ra**)

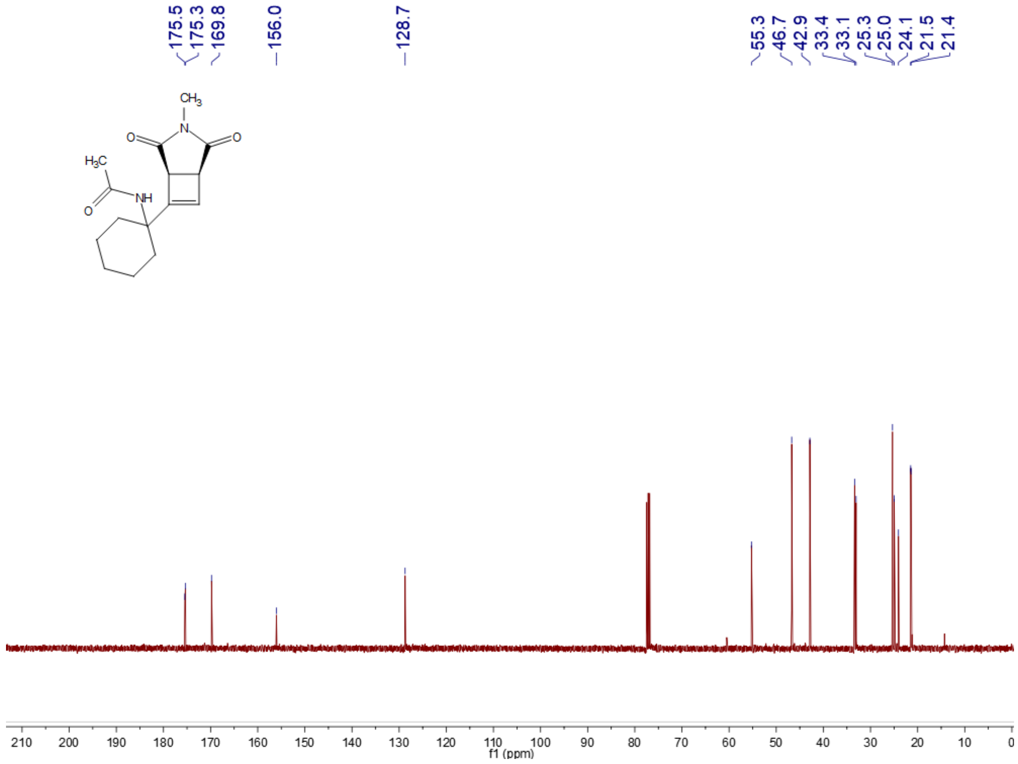

Supplementary Figure 49. ^{13}C NMR (100 MHz, $CDCl_3$) spectra of *N*-(1-(3-methyl-2,4-dioxo-3-azabicyclo[3.2.0]hept-6-en-6-yl)cyclohexyl)acetamide (**3ra**)

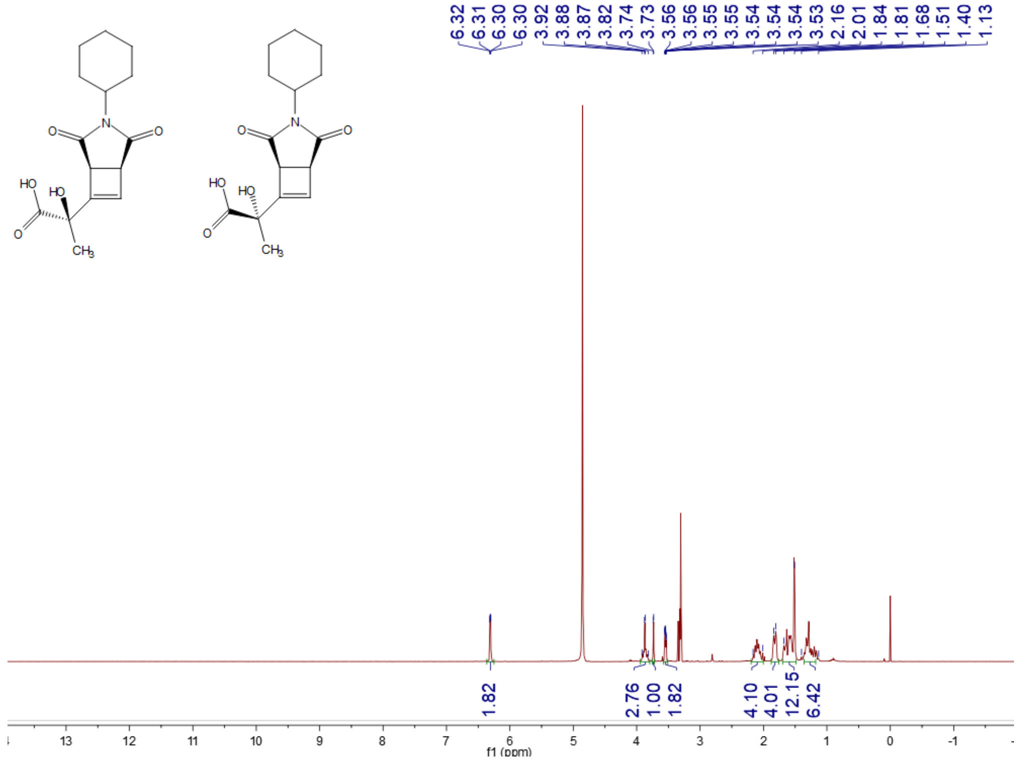

Supplementary Figure 50. ^{1}H NMR (400 MHz, CD_3OD) spectra of 2-(3-cyclohexyl-2,4-dioxo-3-azabicyclo[3.2.0]hept-6-en-6-yl)-2-hydroxypropanoic acid (**3si**)

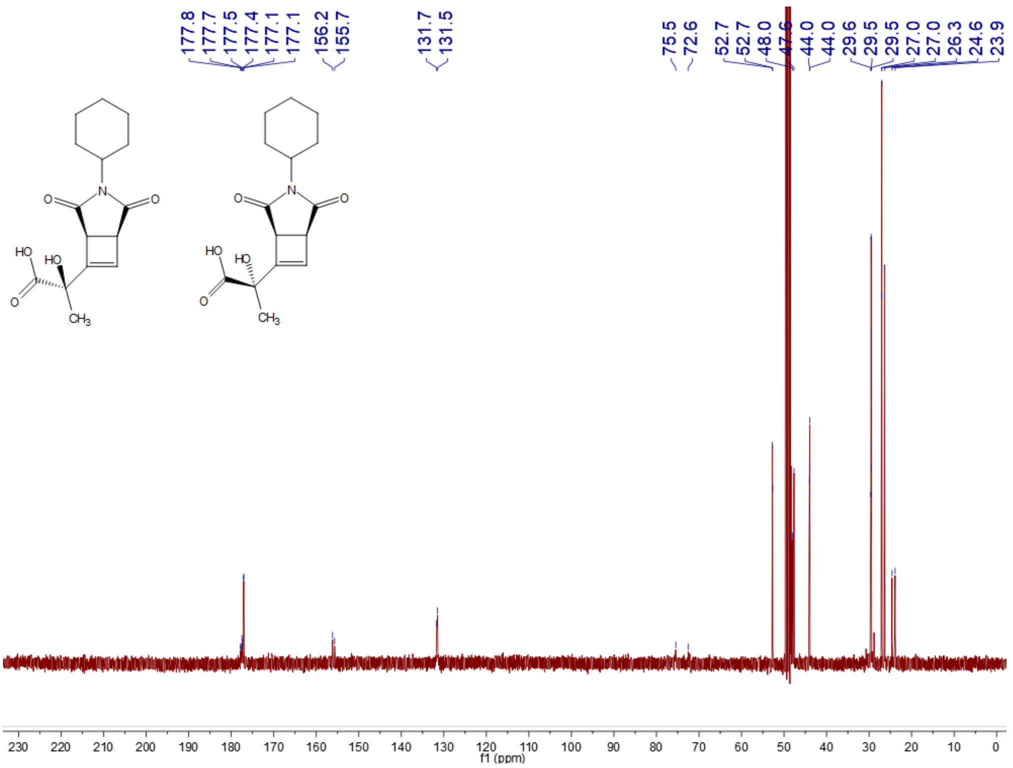

Supplementary Figure 51. ^{13}C NMR (100 MHz, CD_3OD) spectra of 2-(3-cyclohexyl-2,4-dioxo-3-azabicyclo[3.2.0]hept-6-en-6-yl)-2-hydroxypropanoic acid (**3si**)

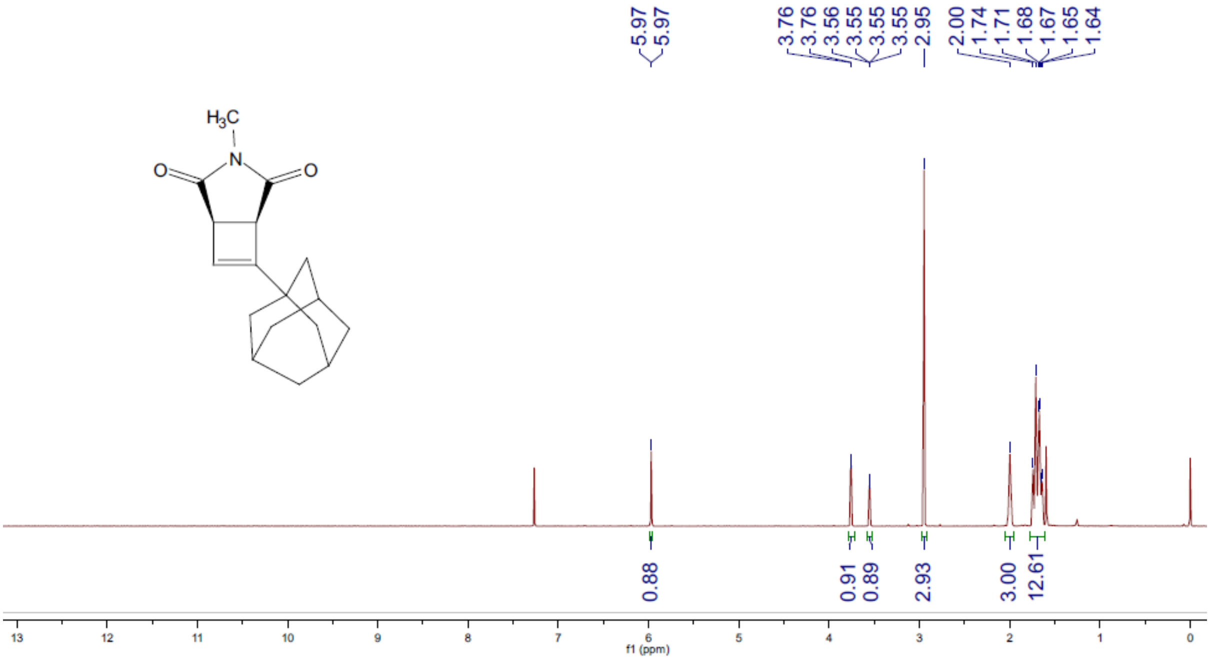

Supplementary Figure 52. ^{1}H NMR (400 MHz, $CDCl_3$) spectra of 6-((1*r*,3*r*,5*r*,7*r*)-adamantan-2-yl)-3-methyl-3-azabicyclo[3.2.0]hept-6-ene-2,4-dione (**3ta**)

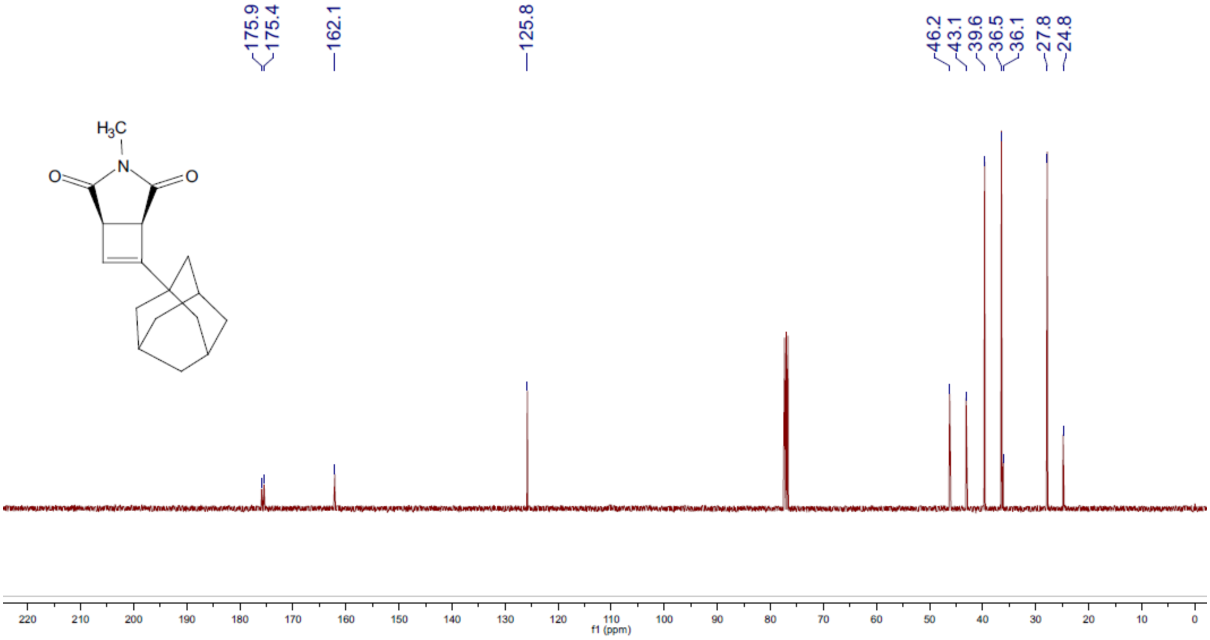

Supplementary Figure 53. ^{13}C NMR (100 MHz, $CDCl_3$) spectra of 6-((1*r*,3*r*,5*r*,7*r*)-adamantan-2-yl)-3-methyl-3-azabicyclo[3.2.0]hept-6-ene-2,4-dione (**3ta**)

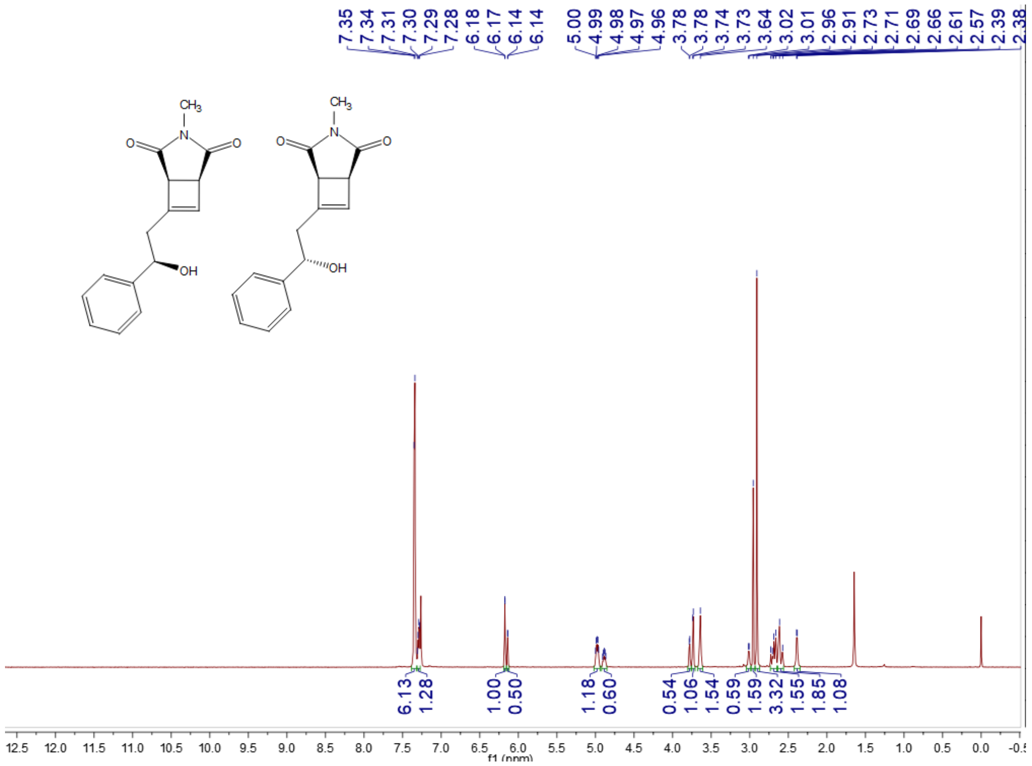

Supplementary Figure 54. ^{1}H NMR (400 MHz, $CDCl_3$) spectra of 6-(2-hydroxy-2-phenylethyl)-3-methyl-3-azabicyclo[3.2.0]hept-6-ene-2,4-dione (**3ua**)

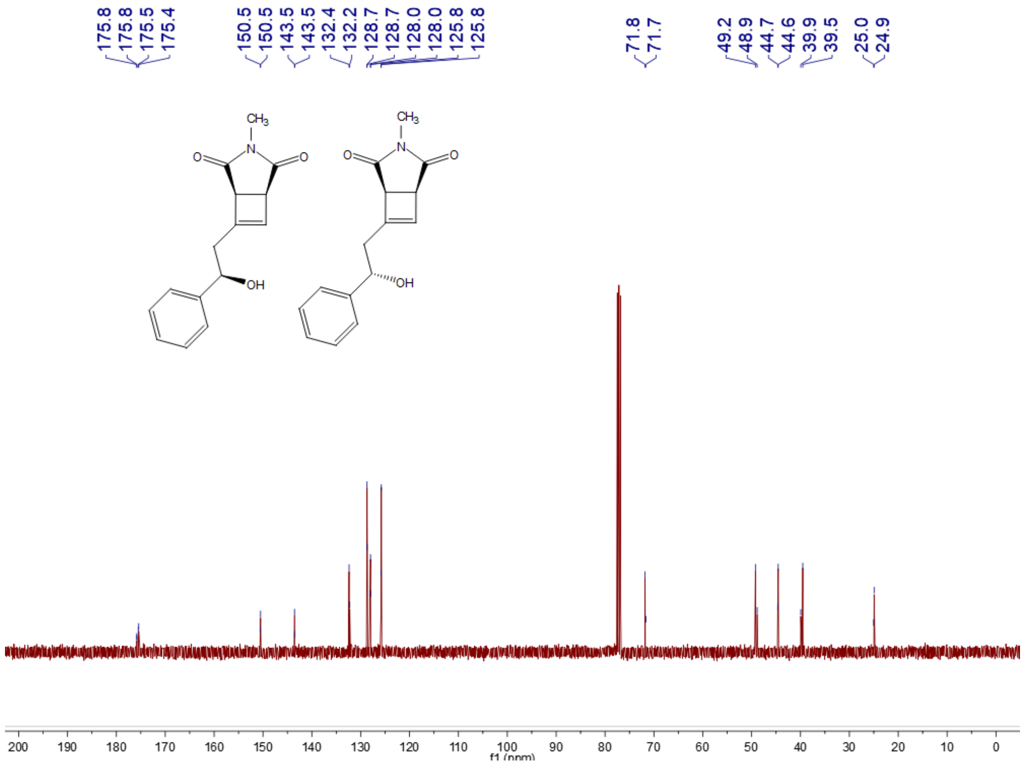

Supplementary Figure 55. ^{13}C NMR (100 MHz, $CDCl_3$) spectra of 6-(2-hydroxy-2-phenylethyl)-3-methyl-3-azabicyclo[3.2.0]hept-6-ene-2,4-dione (**3ua**)

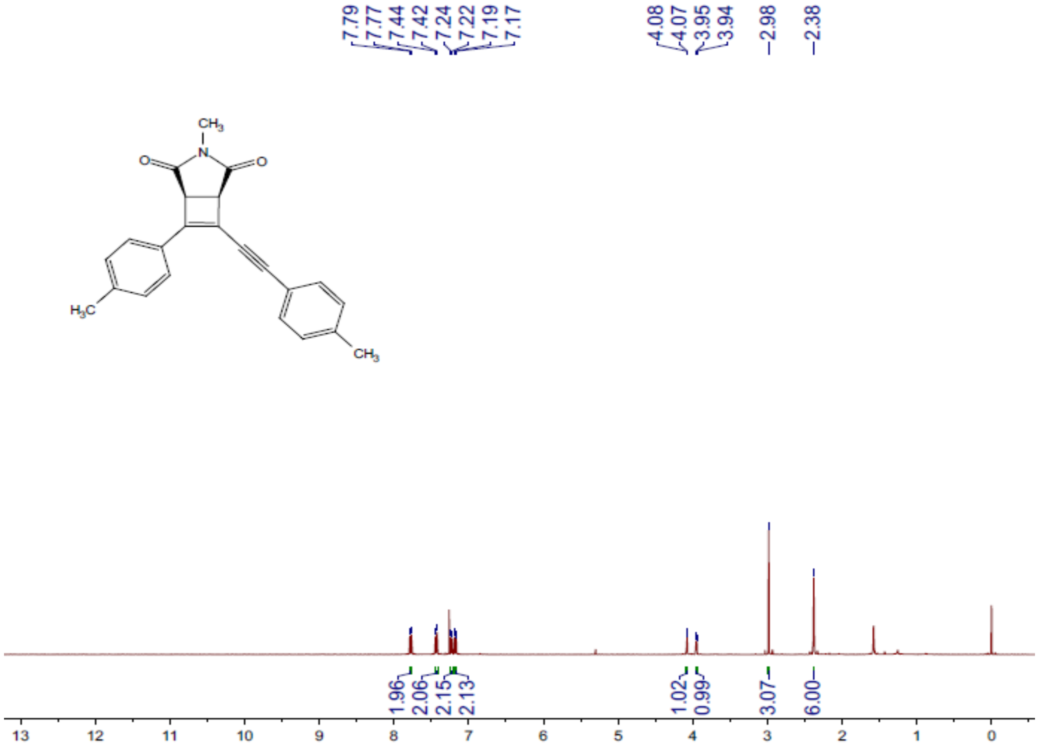

Supplementary Figure 56. ^{1}H NMR (400 MHz, $CDCl_3$) spectra of 3-methyl-6-(*p*-tolyl)-7-(*p*-tolylethynyl)-3-azabicyclo[3.2.0]hept-6-ene-2,4-dione (**3va**)

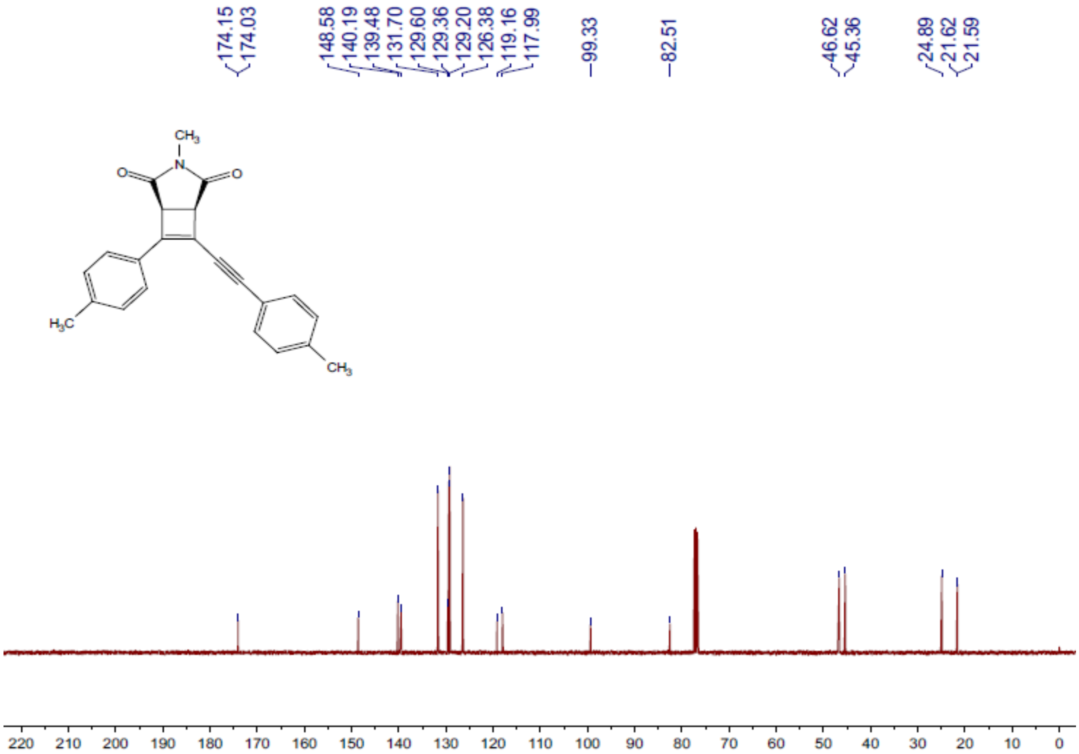

Supplementary Figure 57. ^{13}C NMR (100 MHz, $CDCl_3$) spectra of 3-methyl-6-(*p*-tolyl)-7-(*p*-tolylethynyl)-3-azabicyclo[3.2.0]hept-6-ene-2,4-dione (**3va**)

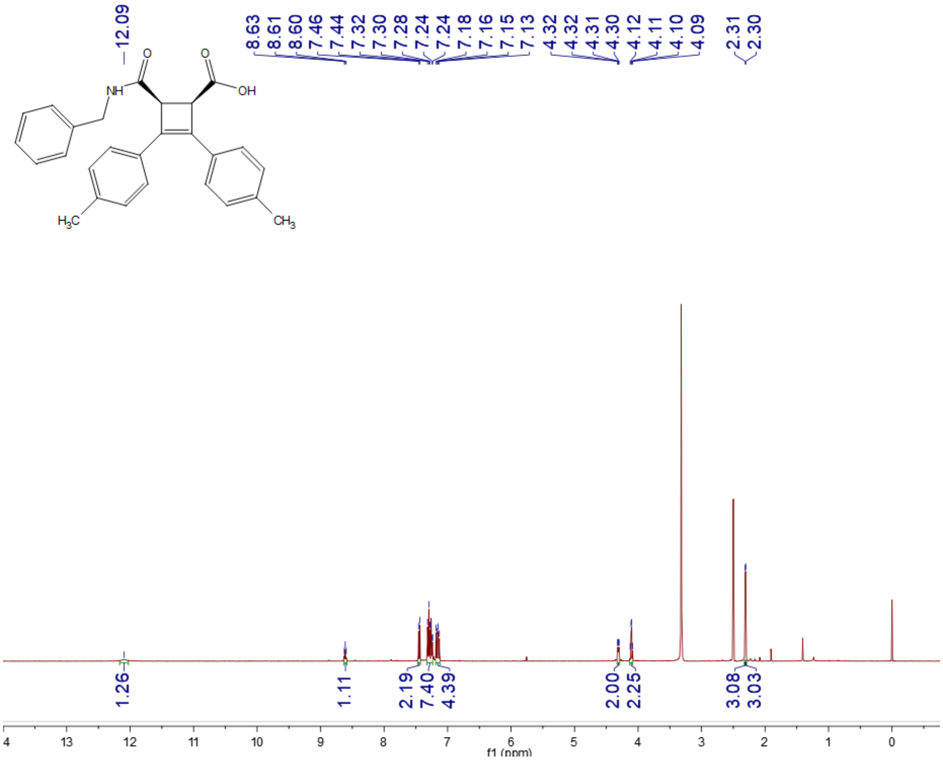

Supplementary Figure 58. ^{1}H NMR (400 MHz, DMSO-d_6) spectra of 4-(benzylcarbamoyl)-2,3-di-*p*-tolylcyclobut-2-ene-1-carboxylic acid (**3ab′**)

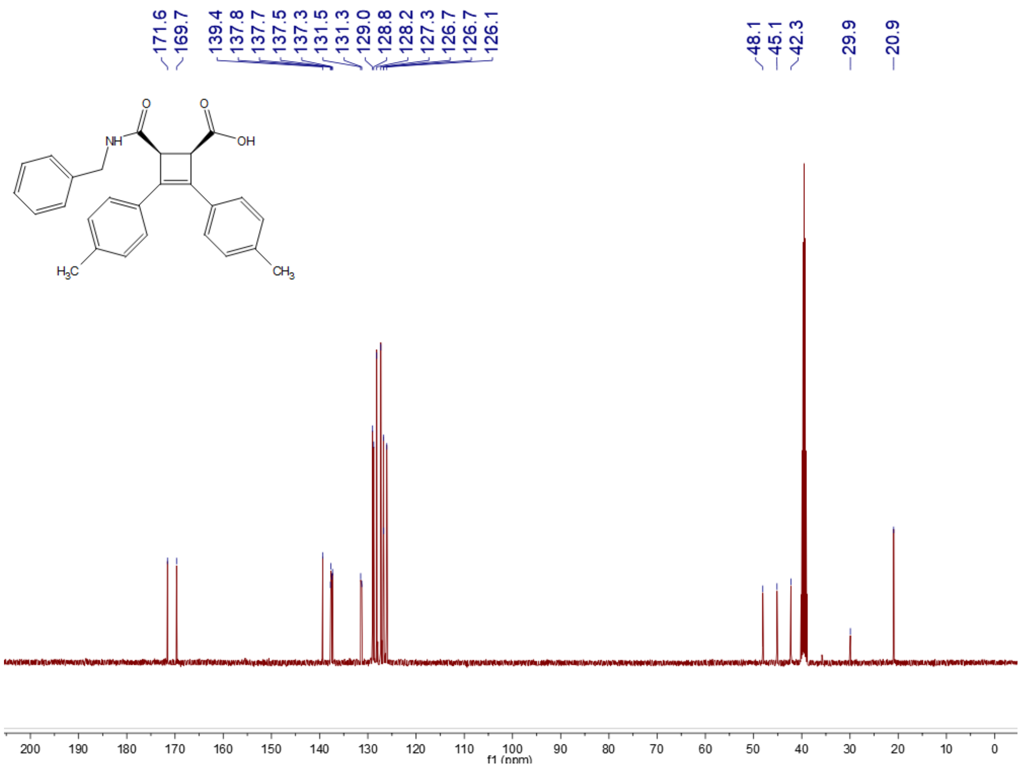

Supplementary Figure 59. ^{13}C NMR (100 MHz, DMSO-d_6) spectra of 4-(benzylcarbamoyl)-2,3-di-*p*-tolylcyclobut-2-ene-1-carboxylic acid (**3ab′**)

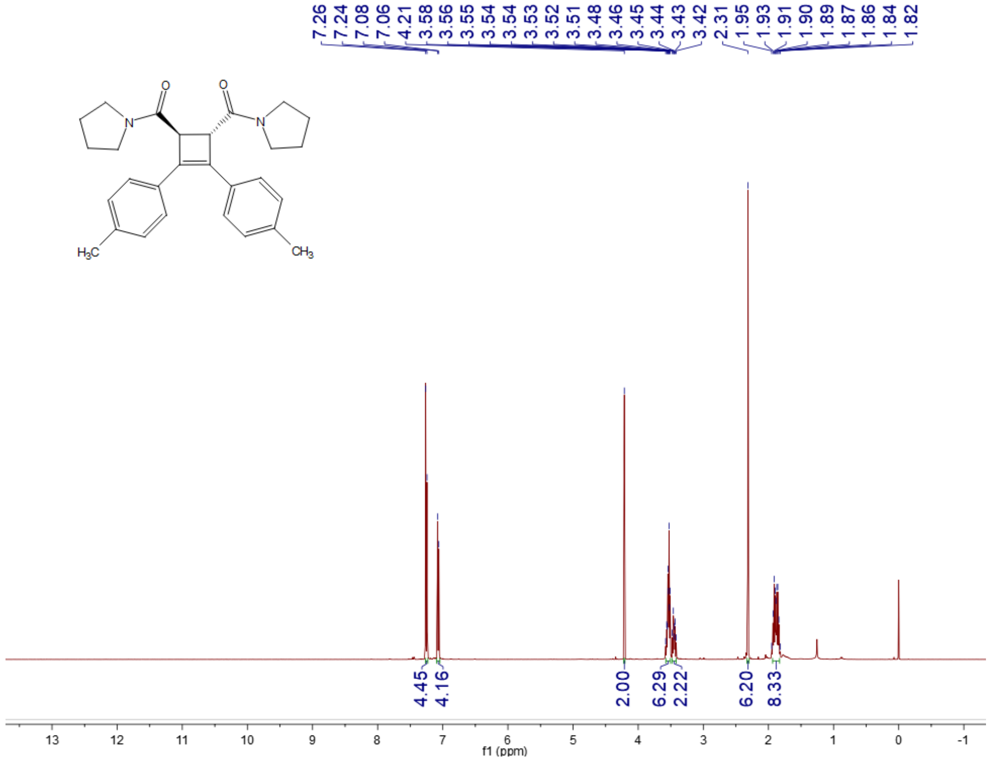

Supplementary Figure 60. ^{1}H NMR (400 MHz, $CDCl_3$) spectra of (3,4-di-*p*-tolylcyclobut-3-ene-1,2-diyl)bis(pyrrolidin-1-ylmethanone) (**3ac**)

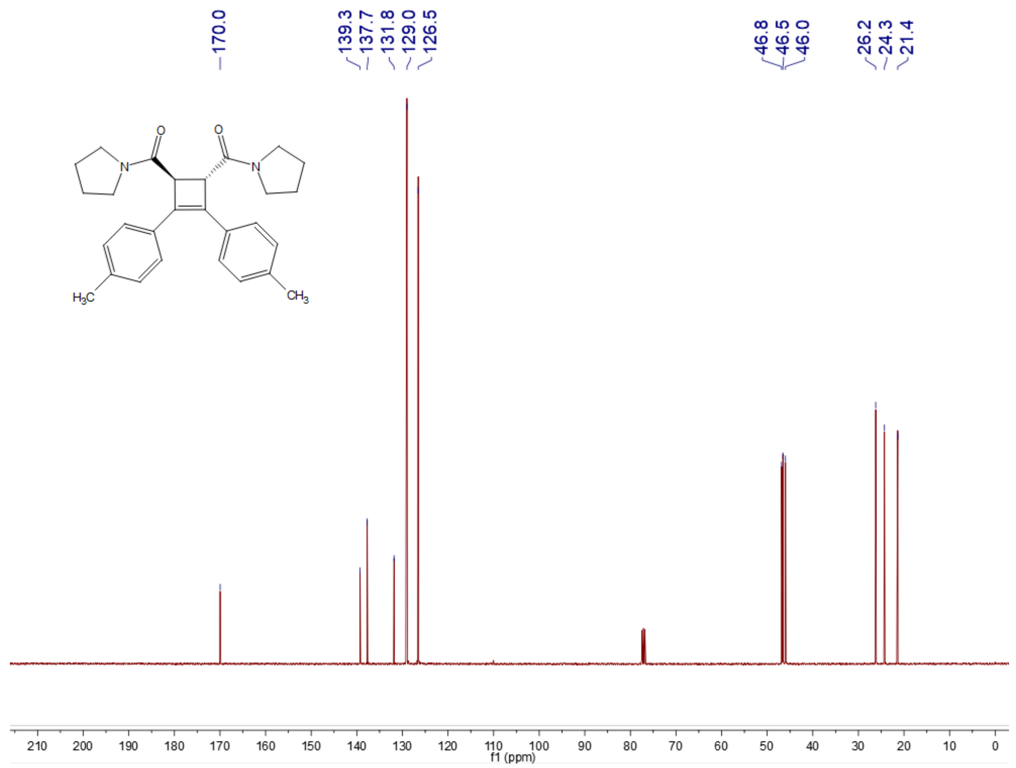

Supplementary Figure 61. ^{13}C NMR (100 MHz, $CDCl_3$) spectra of (3,4-di-*p*-tolylcyclobut-3-ene-1,2-diyl)bis(pyrrolidin-1-ylmethanone) (**3ac**)

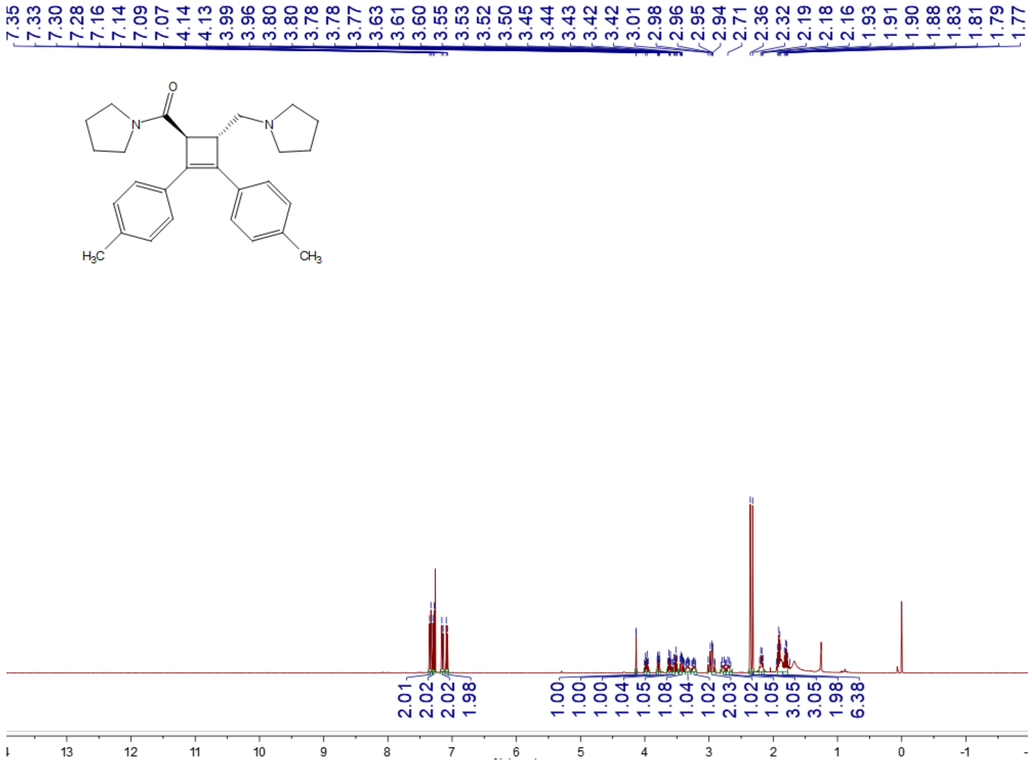

Supplementary Figure 62. ^{1}H NMR (400 MHz, $CDCl_3$) spectra of pyrrolidin-1-yl(4-(pyrrolidin-1-ylmethyl)-2,3-di-*p*-tolylcyclobut-2-en-1-yl)methanone (**3ac′**)

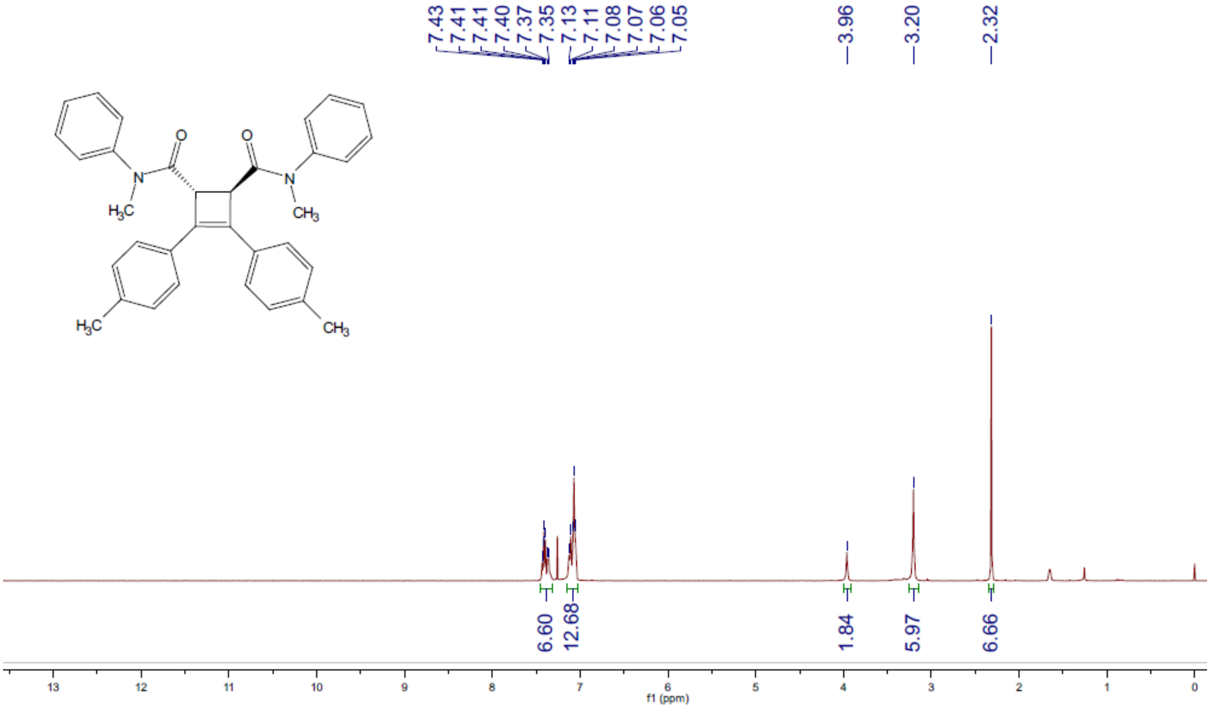

Supplementary Figure 63. ^{1}H NMR (400 MHz, CDCl$_3$) spectra of *N*^{1},*N*^{2}-dimethyl-*N*^{1},*N*^{2}-diphenyl-3,4-di-*p*-tolylcyclobut-3-ene-1,2-dicarboxamide (**3ad**)

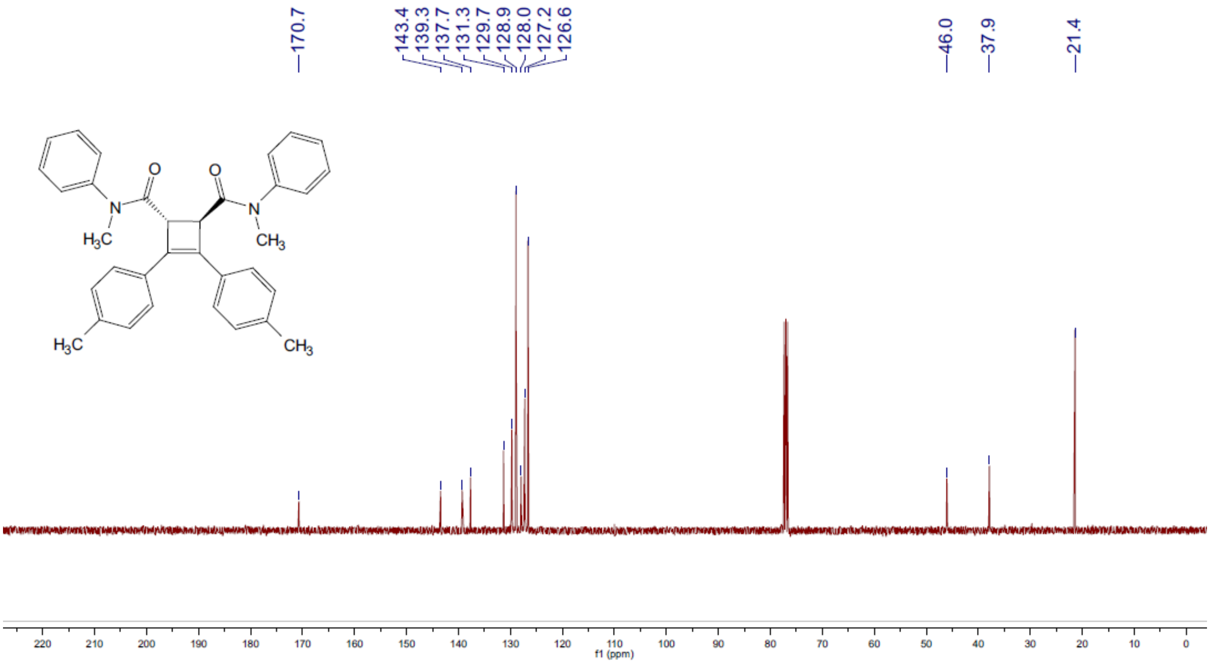

Supplementary Figure 64. ^{13}C NMR (100 MHz, CDCl$_3$) spectra of *N*^{1},*N*^{2}-dimethyl-*N*^{1},*N*^{2}-diphenyl-3,4-di-*p*-tolylcyclobut-3-ene-1,2-dicarboxamide (**3ad**)

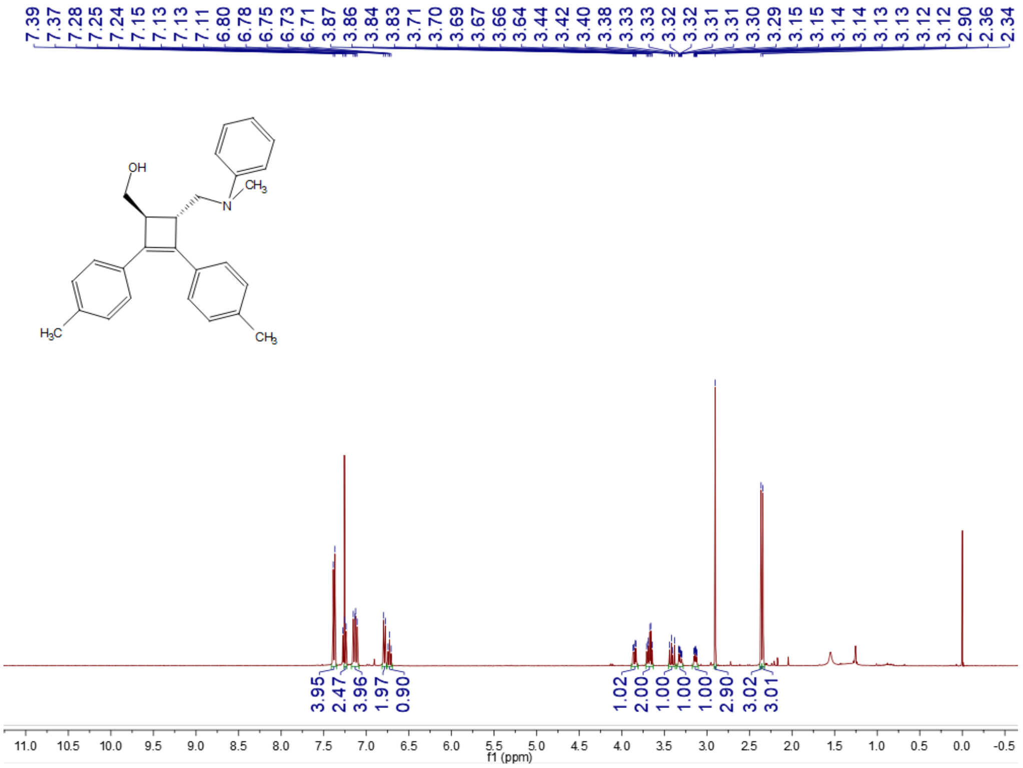

Supplementary Figure 65. ^{1}H NMR (400 MHz, $CDCl_3$) spectra of (4-((methyl(phenyl)amino)methyl)-2,3-di-*p*-tolylcyclobut-2-en-1-yl)methanol (**3ad′**)

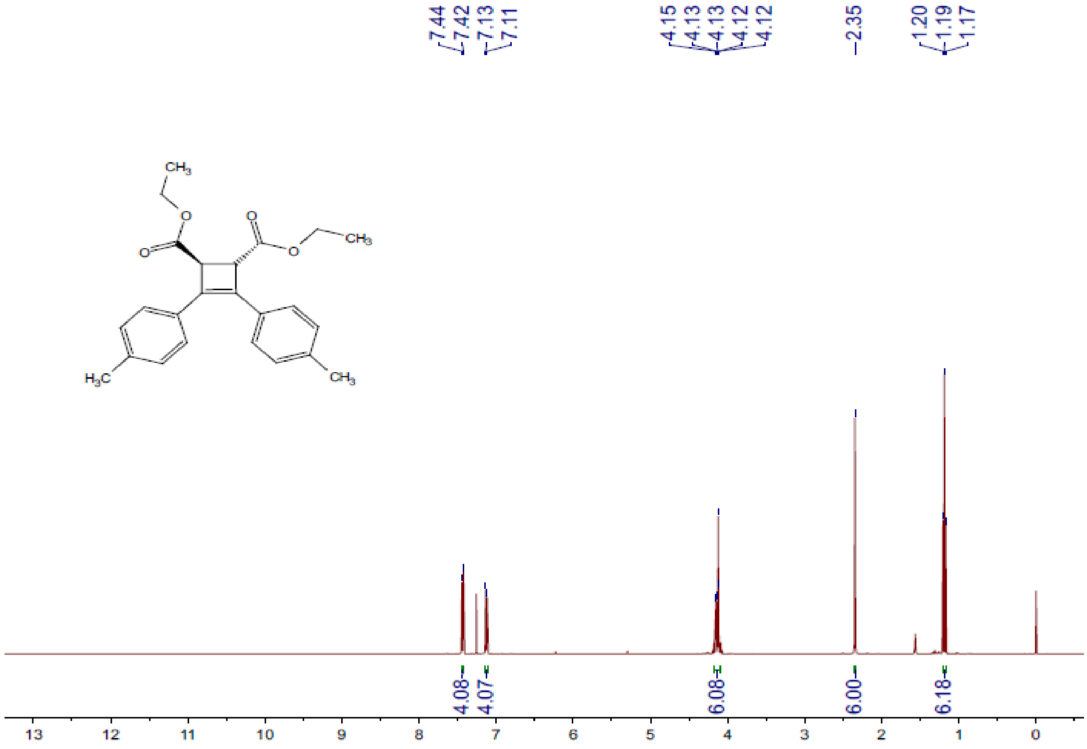

Supplementary Figure 66. ^{1}H NMR (400 MHz, $CDCl_3$) spectra of diethyl 3,4-di-*p*-tolylcyclobut-3-ene-1,2-dicarboxylate (**3ae**)

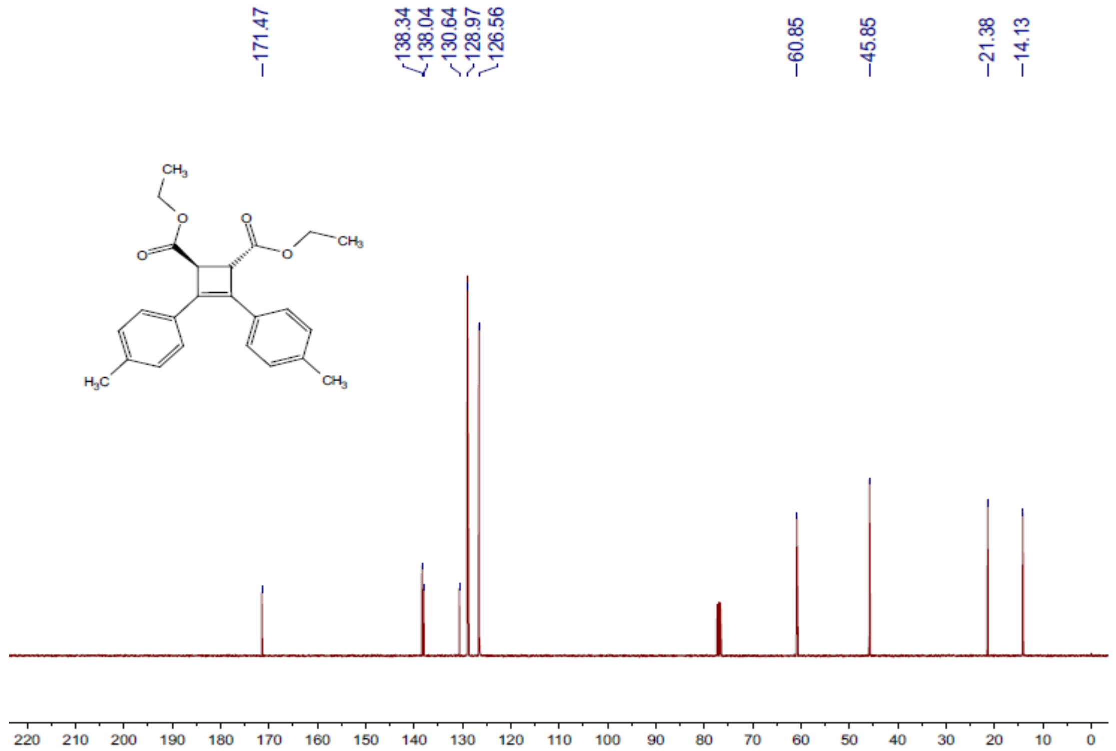

Supplementary Figure 67. ^{13}C NMR (100 MHz, $CDCl_3$) spectra of diethyl 3,4-di-*p*-tolylcyclobut-3-ene-1,2-dicarboxylate (**3ae**)

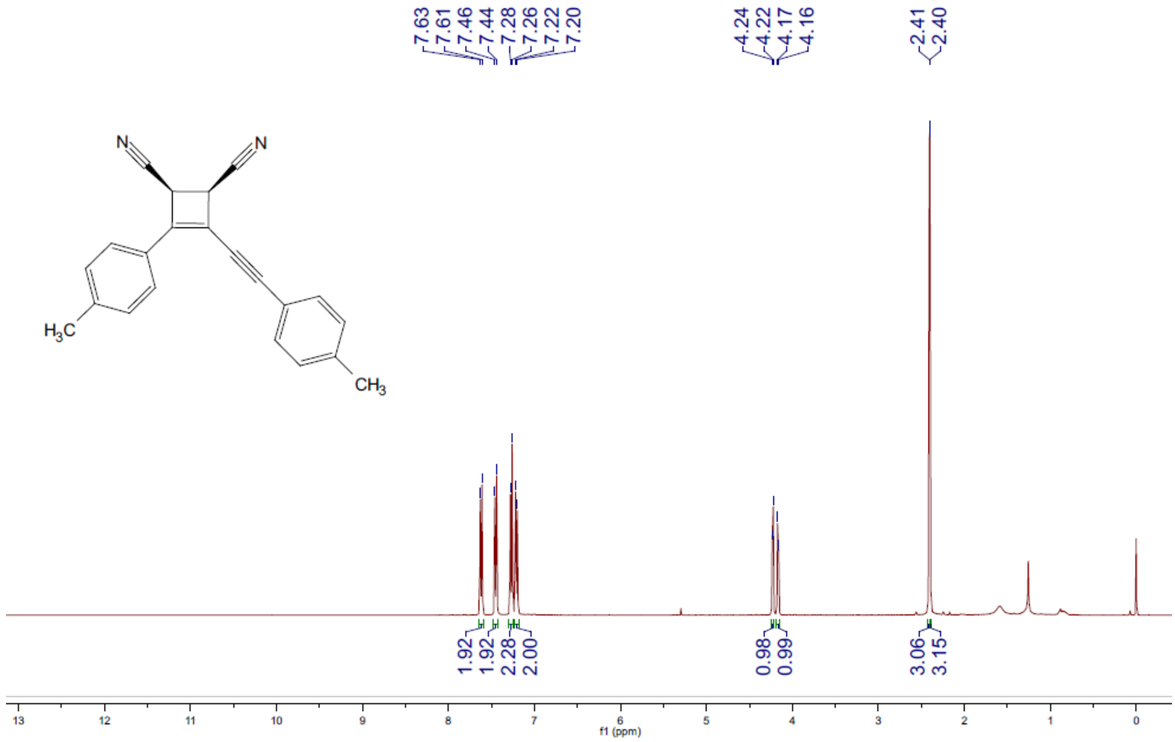

Supplementary Figure 68. ^{1}H NMR (400 MHz, $CDCl_3$) spectra of 3-(*p*-tolyl)-4-(*p*-tolylethynyl)cyclobut-3-ene-1,2-dicarbonitrile (***cis*-3vf**)

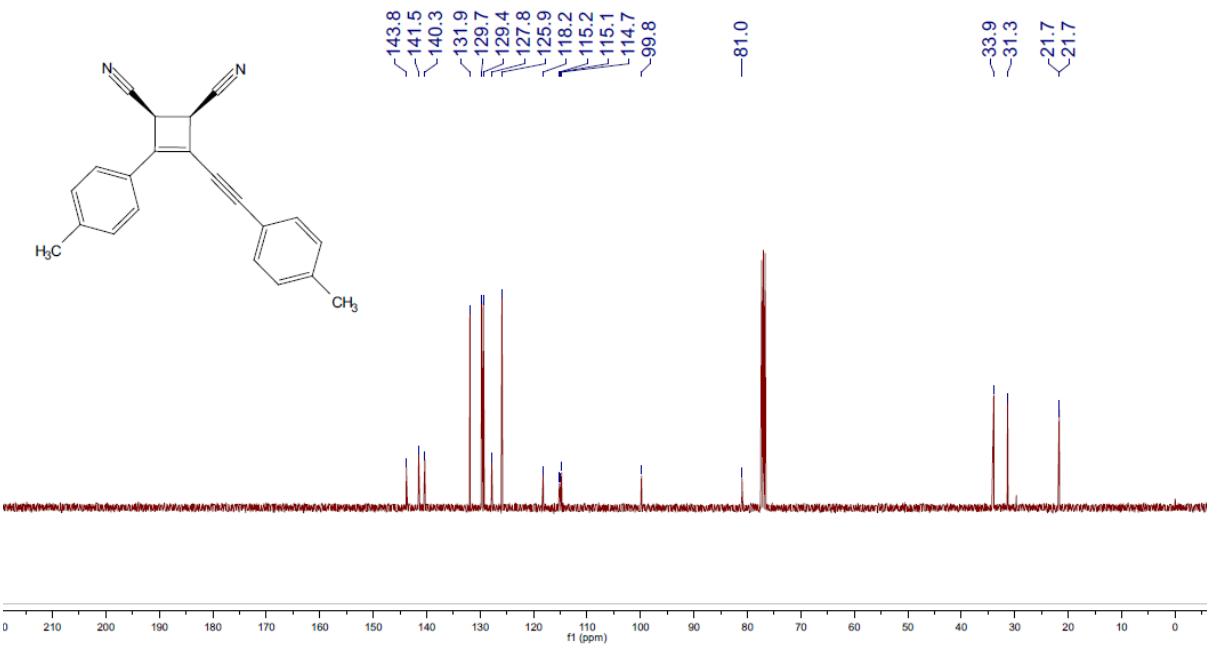

Supplementary Figure 69. ^{13}C NMR (100 MHz, $CDCl_3$) spectra of 3-(*p*-tolyl)-4-(*p*-tolylethynyl)cyclobut-3-ene-1,2-dicarbonitrile (***cis*-3vf**)

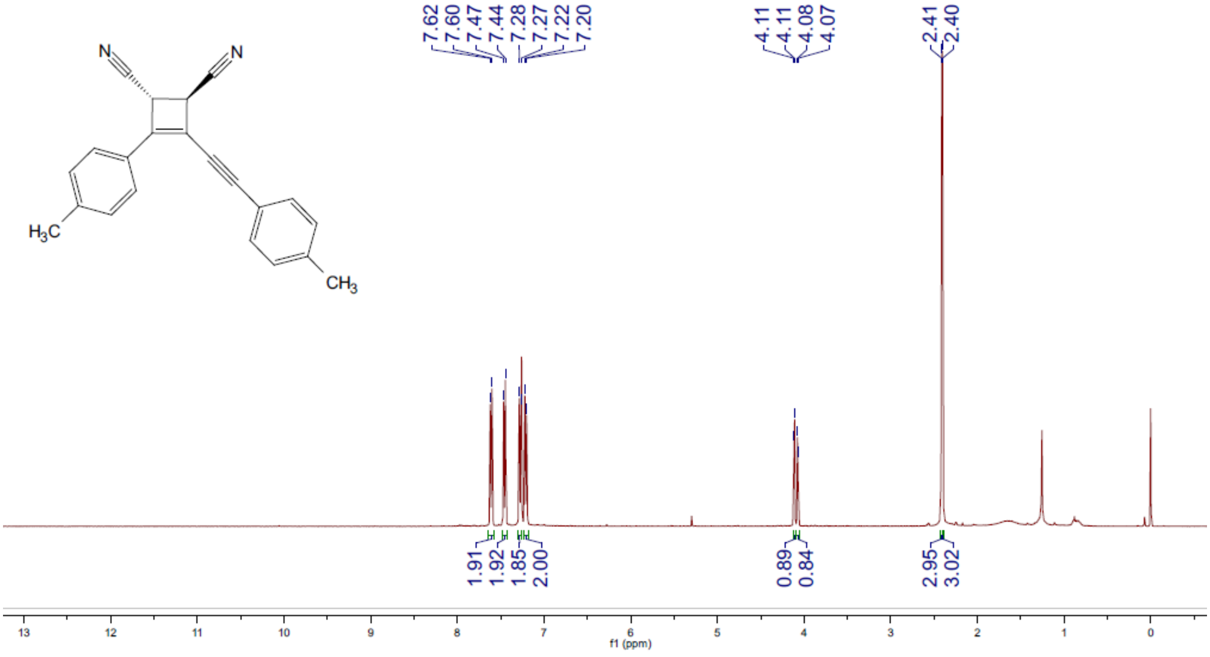

Supplementary Figure 70. ^{1}H NMR (400 MHz, $CDCl_3$) spectra of 3-(*p*-tolyl)-4-(*p*-tolylethynyl)cyclobut-3-ene-1,2-dicarbonitrile (***trans*-3vf**)

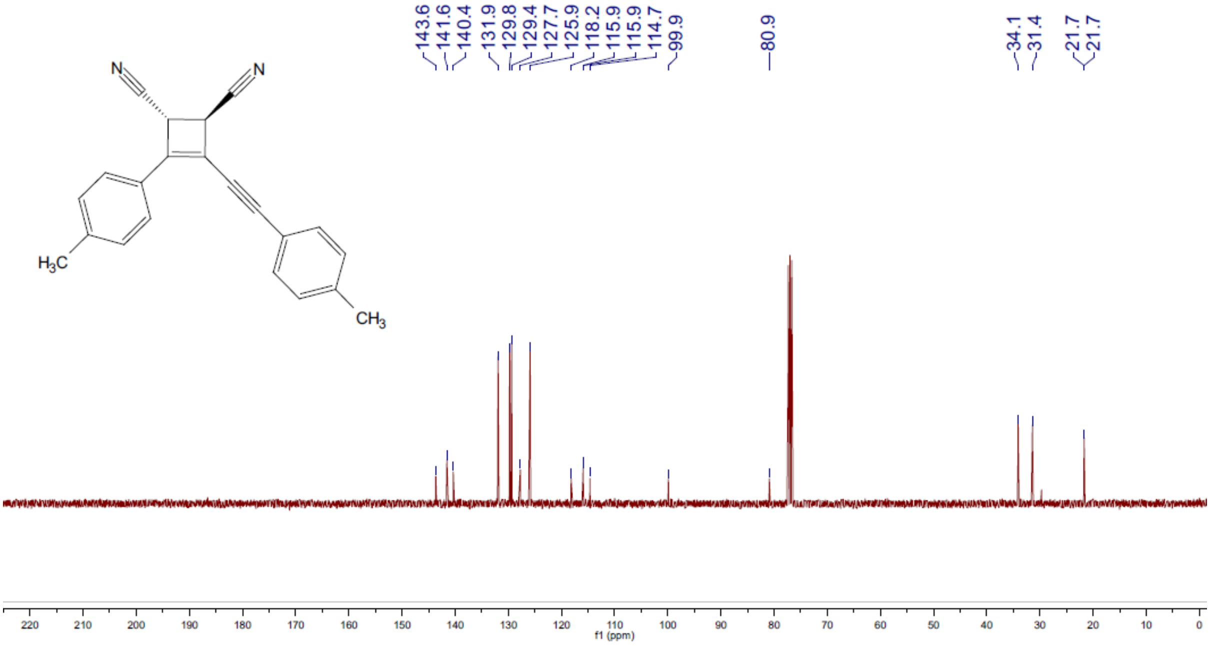

Supplementary Figure 71. ^{13}C NMR (100 MHz, $CDCl_3$) spectra of 3-(*p*-tolyl)-4-(*p*-tolylethynyl)cyclobut-3-ene-1,2-dicarbonitrile (***trans*-3vf**)

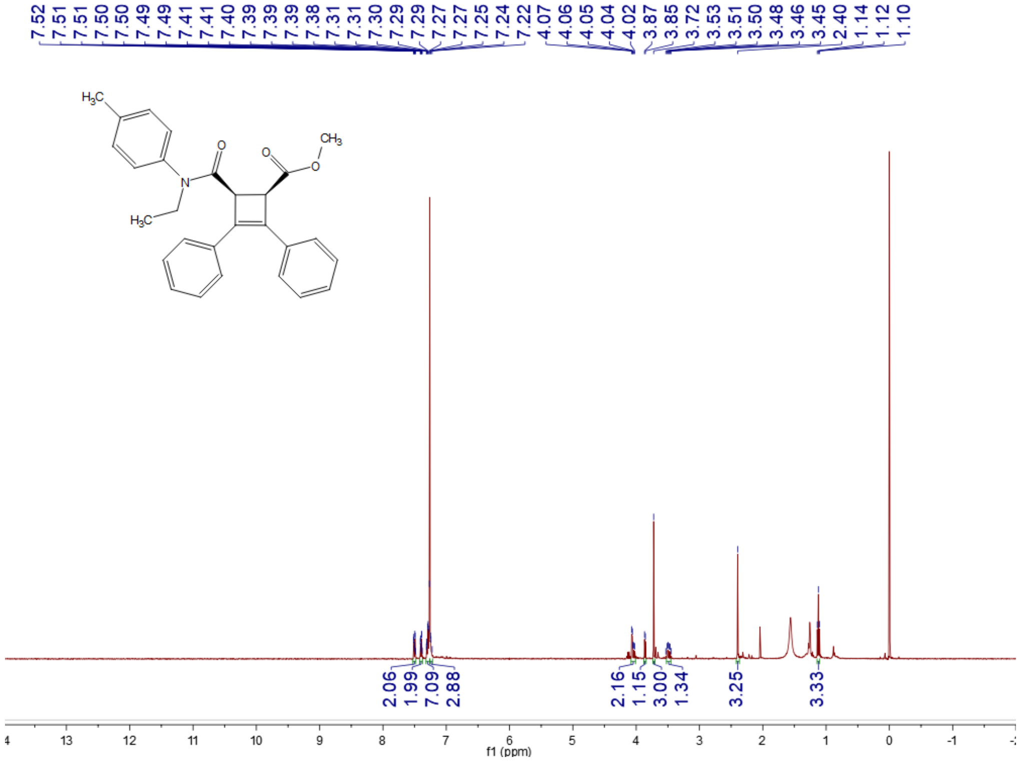

Supplementary Figure 72. ^{1}H NMR (400 MHz, $CDCl_3$) spectra of methyl 4-(ethyl(*p*-tolyl)carbamoyl)-2,3-diphenylcyclobut-2-ene-1-carboxylate (***cis*-3cg**)

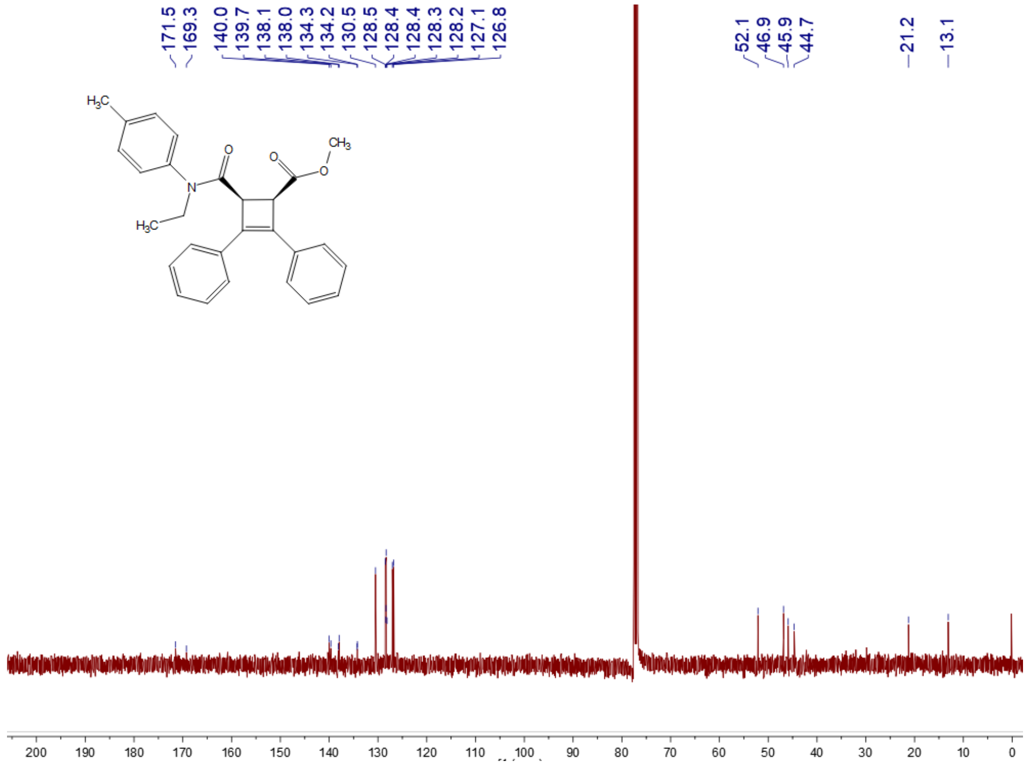

Supplementary Figure 73. ^{13}C NMR (100 MHz, $CDCl_3$) spectra of methyl 4-(ethyl(*p*-tolyl)carbamoyl)-2,3-diphenylcyclobut-2-ene-1-carboxylate (***cis*-3cg**)

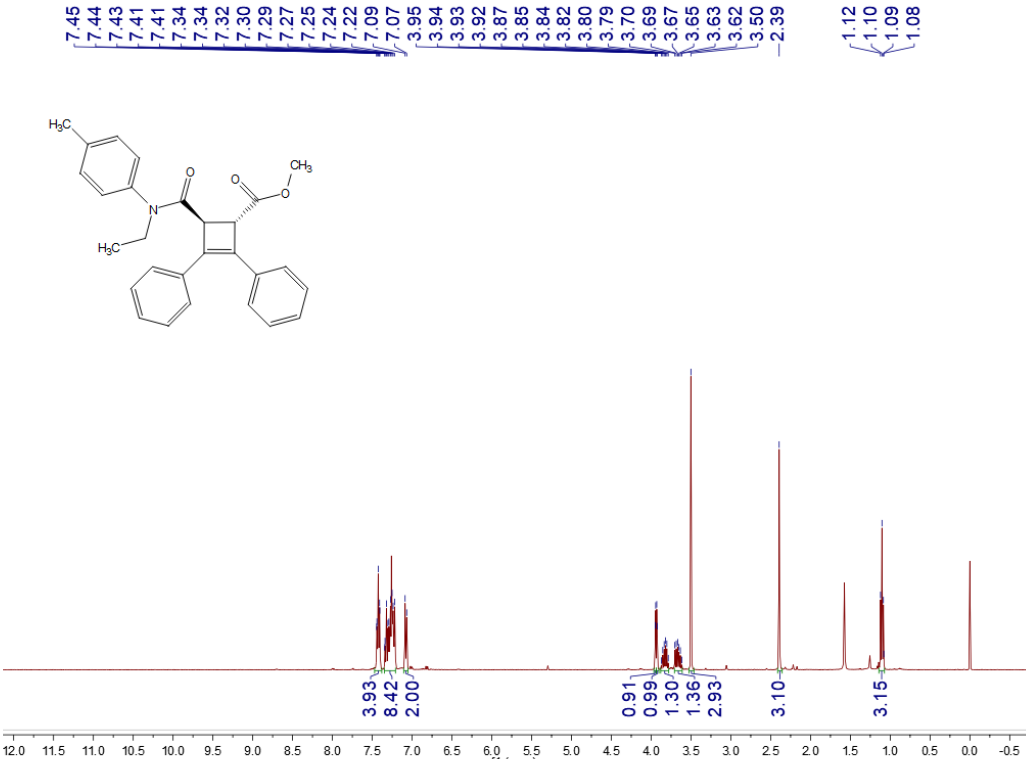

Supplementary Figure 74. ^{1}H NMR (400 MHz, $CDCl_3$) spectra of methyl 4-(ethyl(*p*-tolyl)carbamoyl)-2,3-diphenylcyclobut-2-ene-1-carboxylate (***trans*-3cg**)

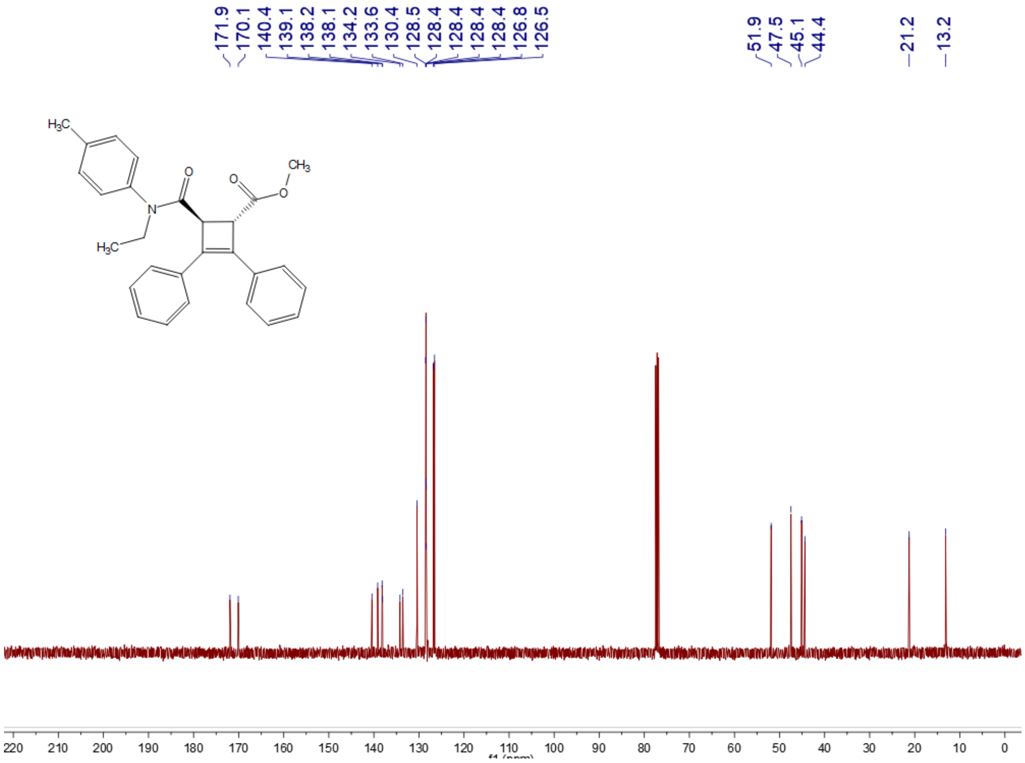

Supplementary Figure 75. ^{13}C NMR (100 MHz, $CDCl_3$) spectra of methyl 4-(ethyl(*p*-tolyl)carbamoyl)-2,3-diphenylcyclobut-2-ene-1-carboxylate (***trans*-3cg**)

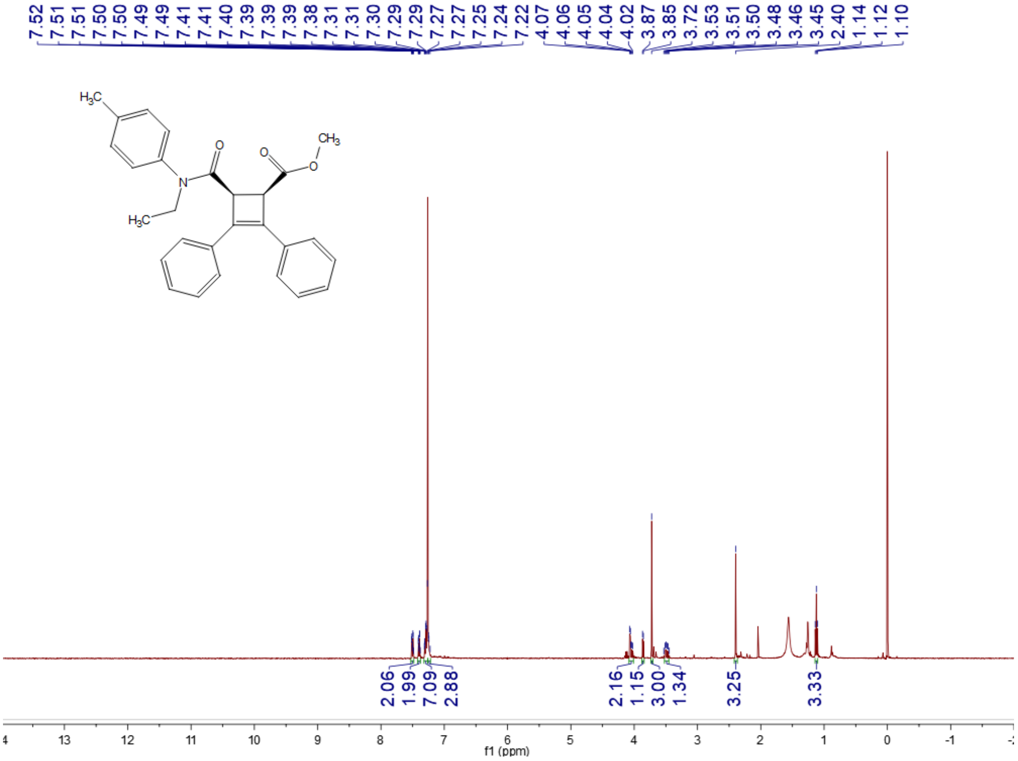

Supplementary Figure 76. ^{1}H NMR (400 MHz, $CDCl_3$) spectra of methyl 4-(ethyl(*p*-tolyl)carbamoyl)-2,3-diphenylcyclobut-2-ene-1-carboxylate (***cis*-3cg**)

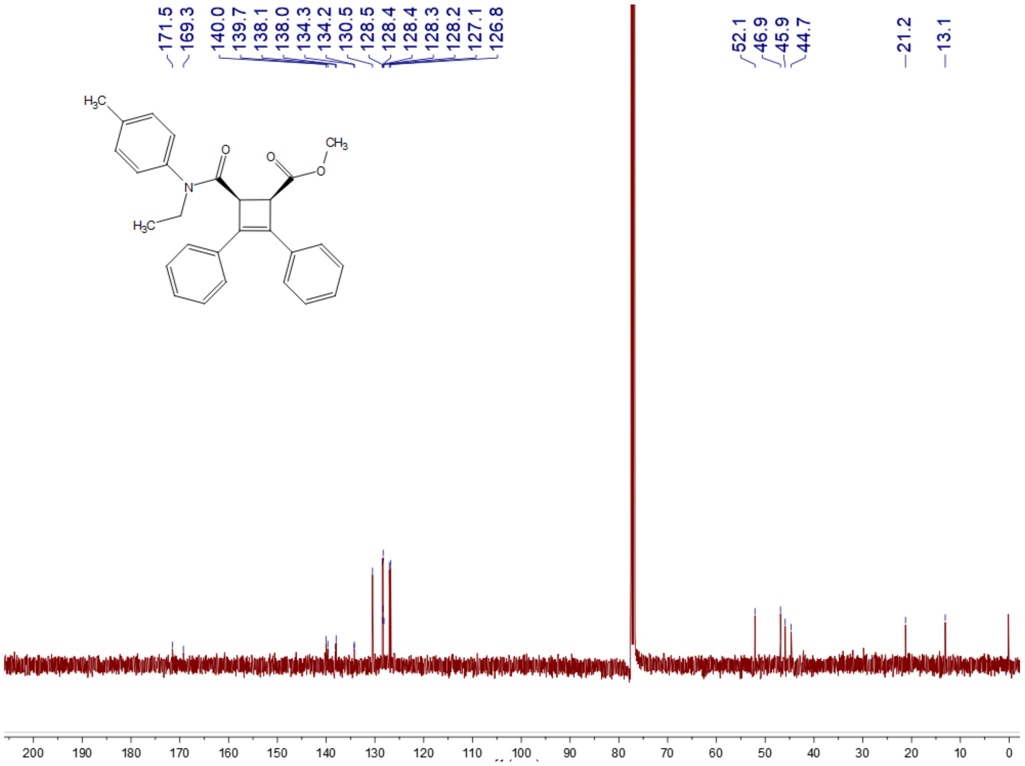

Supplementary Figure 77. ^{13}C NMR (100 MHz, $CDCl_3$) spectra of methyl 4-(ethyl(*p*-tolyl)carbamoyl)-2,3-diphenylcyclobut-2-ene-1-carboxylate (***cis*-3cg**)

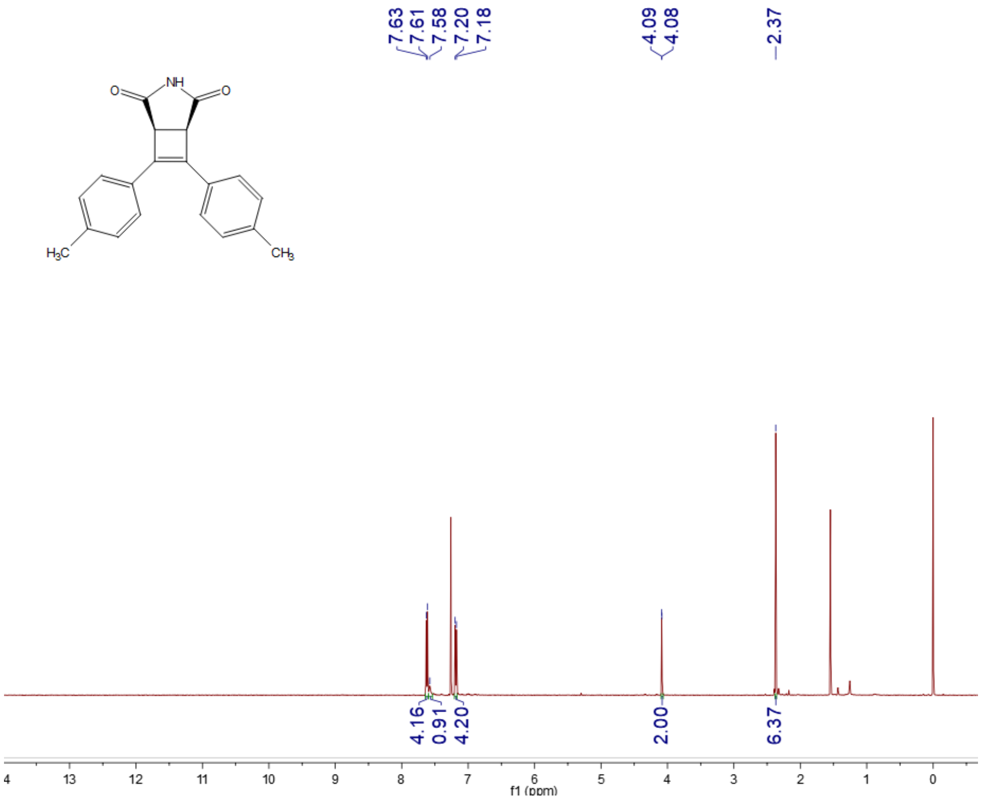

Supplementary Figure 78. ^{1}H NMR (400 MHz, $CDCl_3$) spectra of 6,7-di-*p*-tolyl-3-azabicyclo[3.2.0]hept-6-ene-2,4-dione (**3ah**)

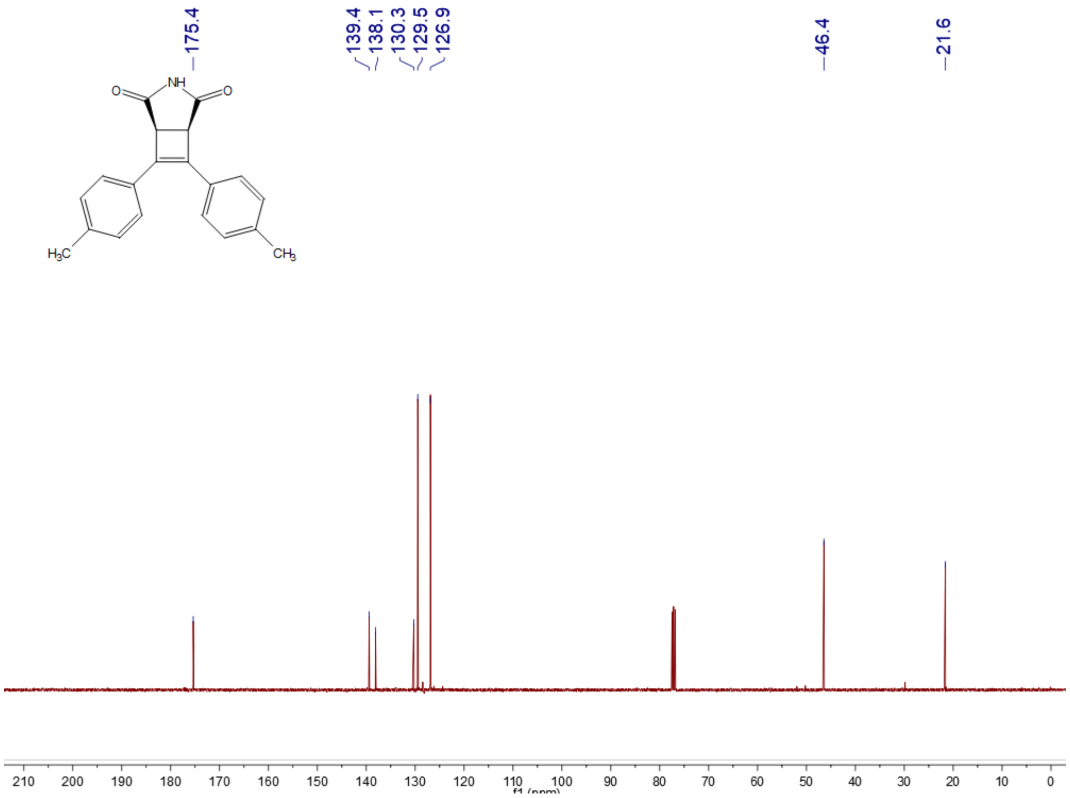

Supplementary Figure 79. ^{13}C NMR (100 MHz, $CDCl_3$) spectra of 6,7-di-*p*-tolyl-3-azabicyclo[3.2.0]hept-6-ene-2,4-dione (**3ah**)

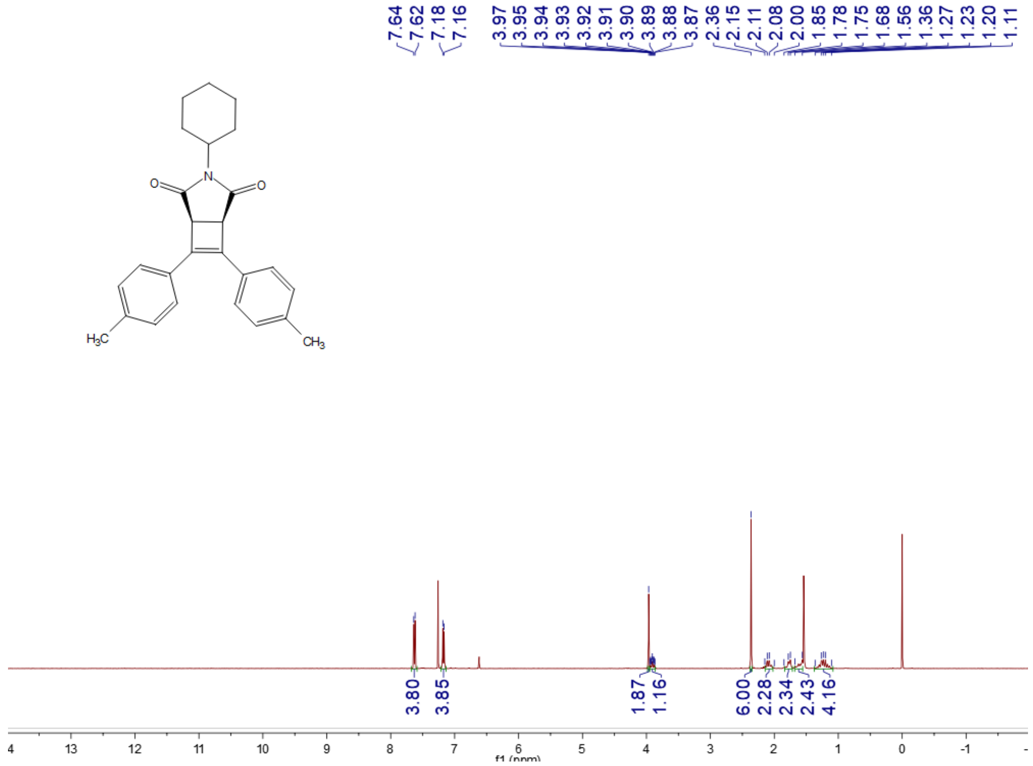

Supplementary Figure 80. ^{1}H NMR (400 MHz, $CDCl_3$) spectra of 3-cyclohexyl-6,7-di-*p*-tolyl-3-azabicyclo[3.2.0]hept-6-ene-2,4-dione (**3ai**)

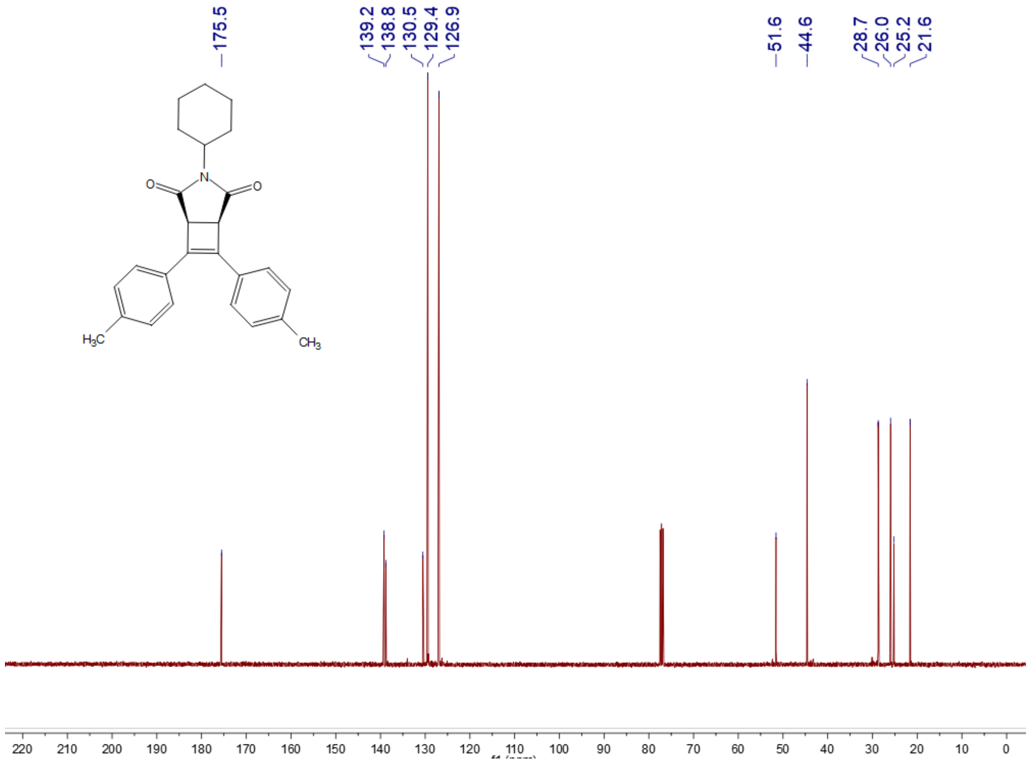

Supplementary Figure 81. ^{13}C NMR (100 MHz, $CDCl_3$) spectra of 3-cyclohexyl-6,7-di-*p*-tolyl-3-azabicyclo[3.2.0]hept-6-ene-2,4-dione (**3ai**)

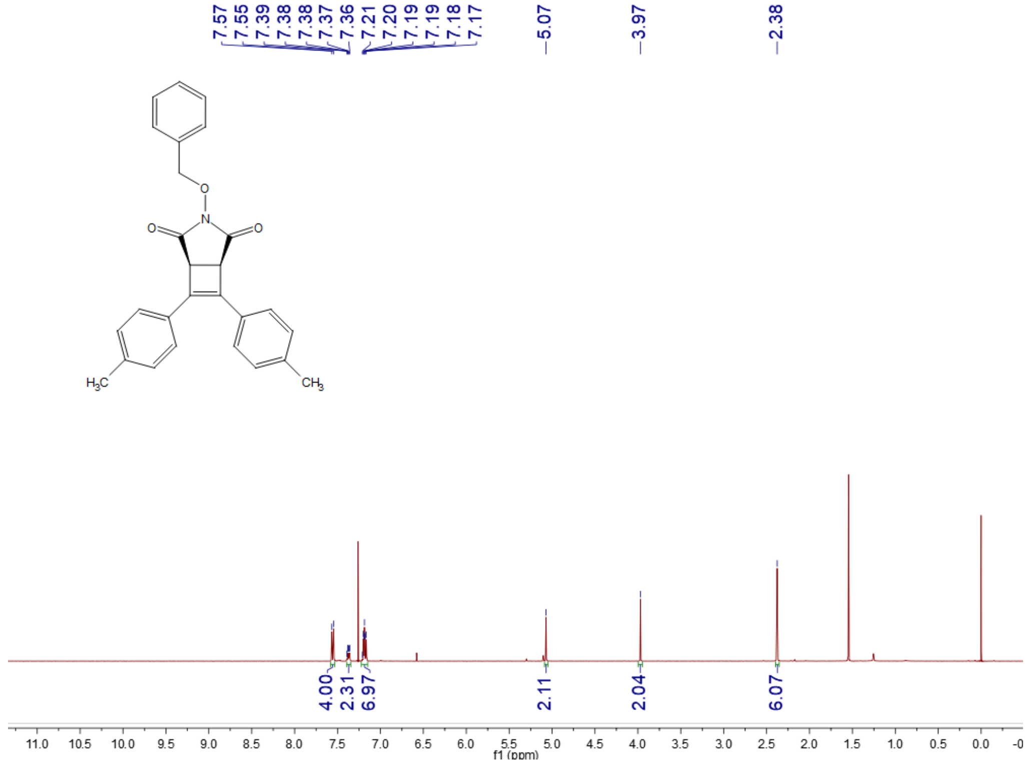

Supplementary Figure 82. ^{1}H NMR (400 MHz, $CDCl_3$) spectra of 3-(benzyloxy)-6,7-di-*p*-tolyl-3-azabicyclo[3.2.0]hept-6-ene-2,4-dione (**3aj**)

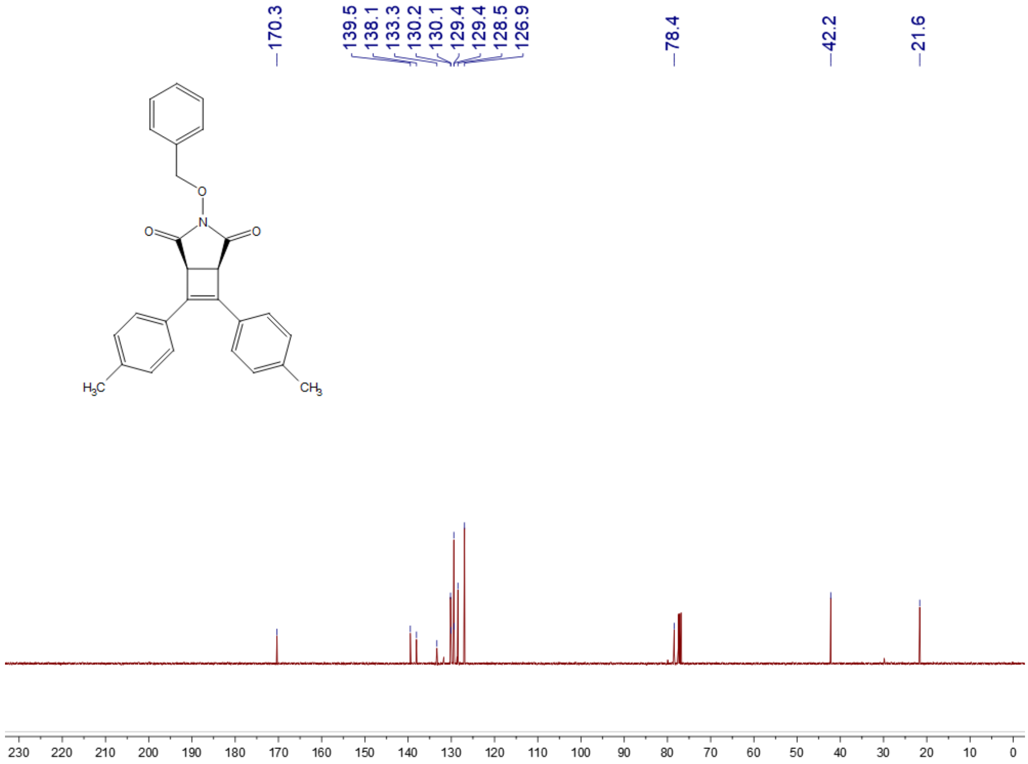

Supplementary Figure 83. ^{13}C NMR (100 MHz, $CDCl_3$) spectra of 3-(benzyloxy)-6,7-di-*p*-tolyl-3-azabicyclo[3.2.0]hept-6-ene-2,4-dione (**3aj**)

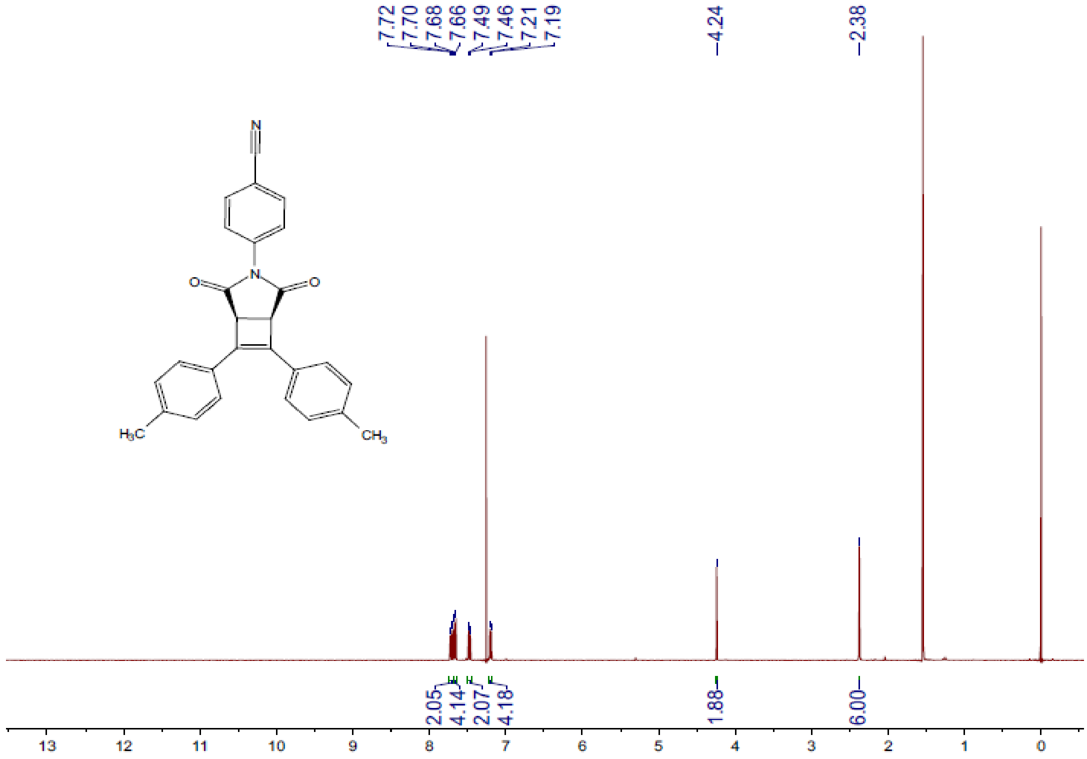

Supplementary Figure 84. ^{1}H NMR (400 MHz, $CDCl_3$) spectra 4-(2,4-dioxo-6,7-di-*p*-tolyl-3-azabicyclo[3.2.0]hept-6-en-3-yl)benzonitrile (**3ak**)

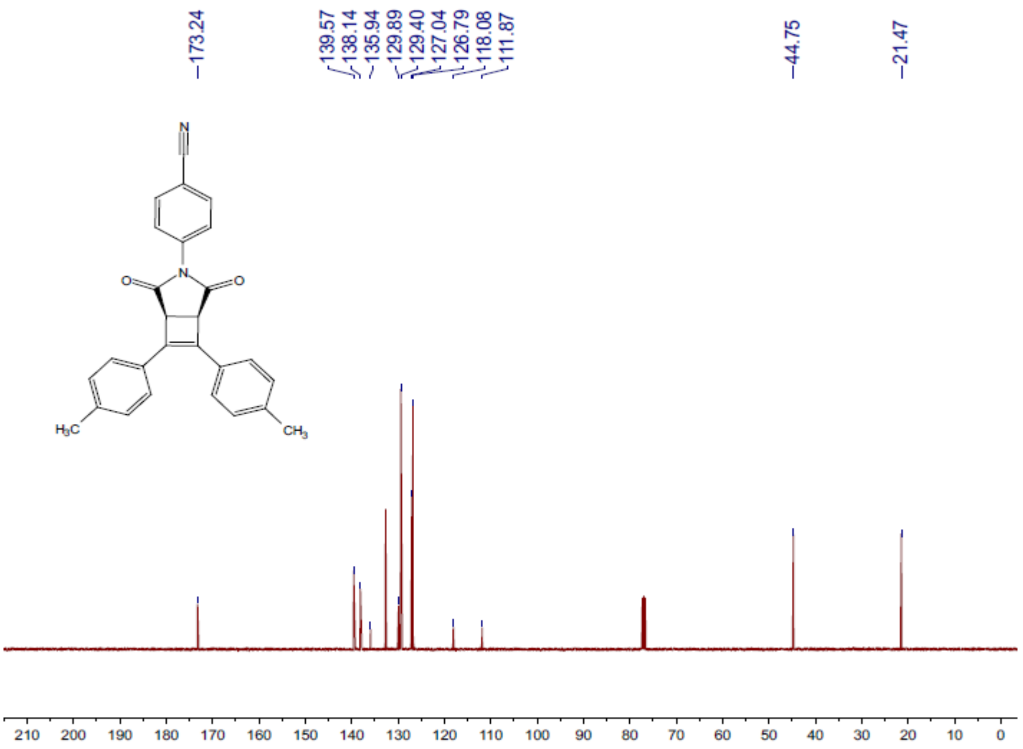

Supplementary Figure 85. ^{13}C NMR (100 MHz, $CDCl_3$) spectra of 4-(2,4-dioxo-6,7-di-*p*-tolyl-3-azabicyclo[3.2.0]hept-6-en-3-yl)benzonitrile (**3ak**)

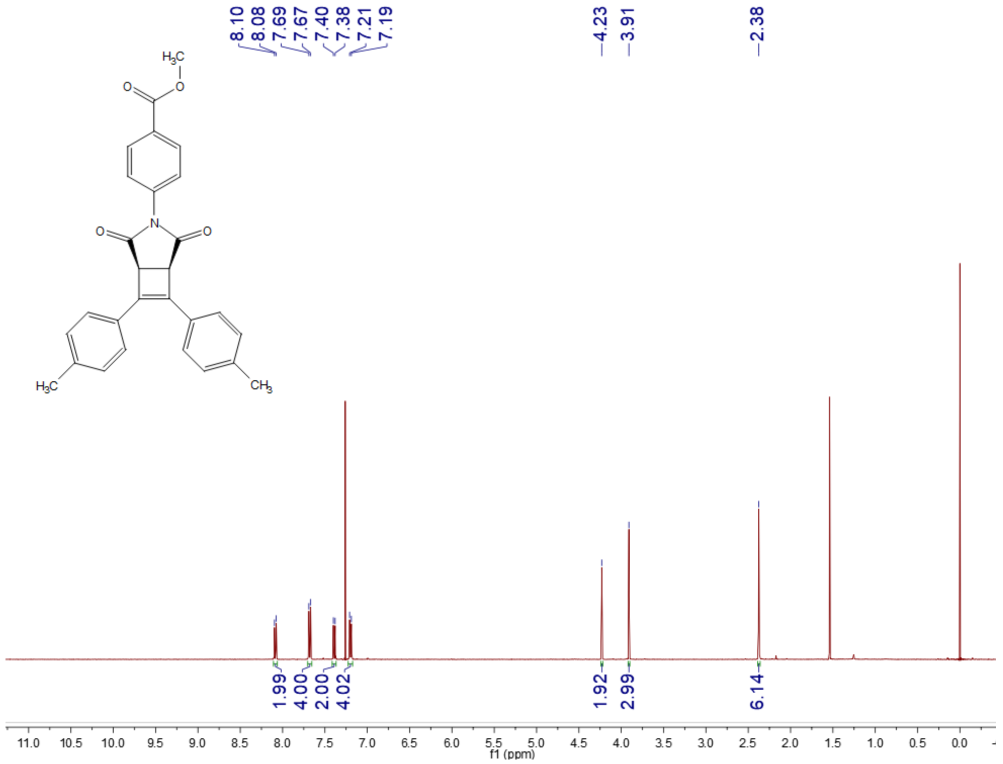

Supplementary Figure 86. ^{1}H NMR (400 MHz, $CDCl_3$) spectra of methyl 4-(2,4-dioxo-6,7-di-*p*-tolyl-3-azabicyclo[3.2.0]hept-6-en-3-yl)benzoate (**3al**)

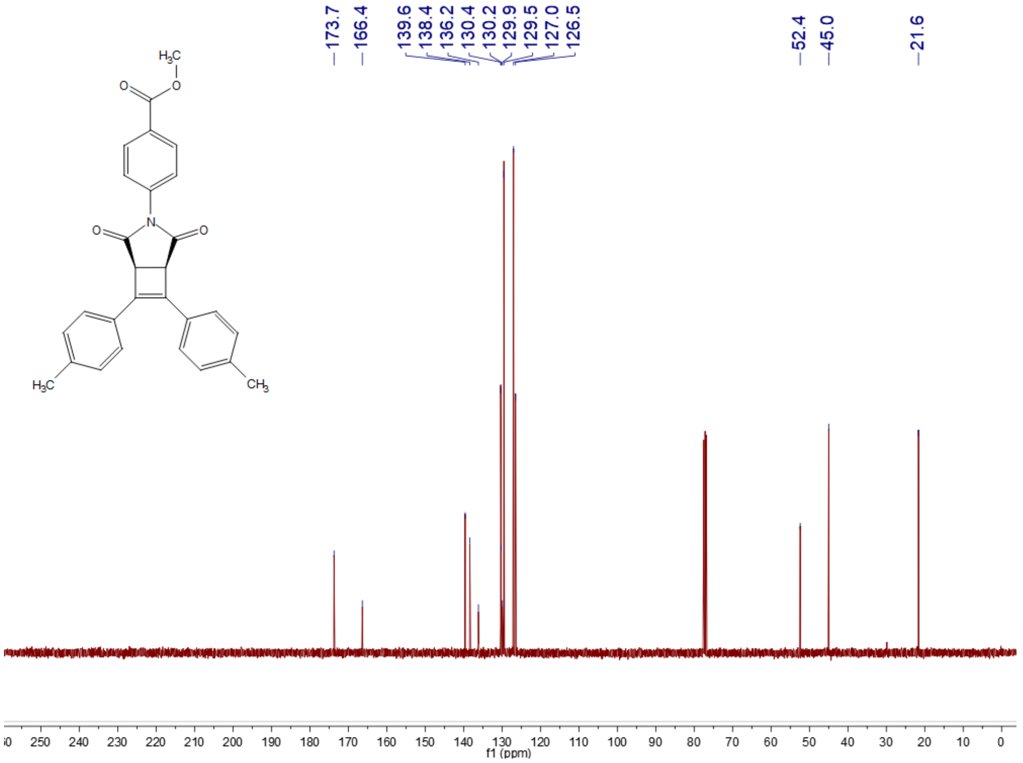

Supplementary Figure 87. ^{13}C NMR (100 MHz, $CDCl_3$) spectra of methyl 4-(2,4-dioxo-6,7-di-*p*-tolyl-3-azabicyclo[3.2.0]hept-6-en-3-yl)benzoate (**3al**)

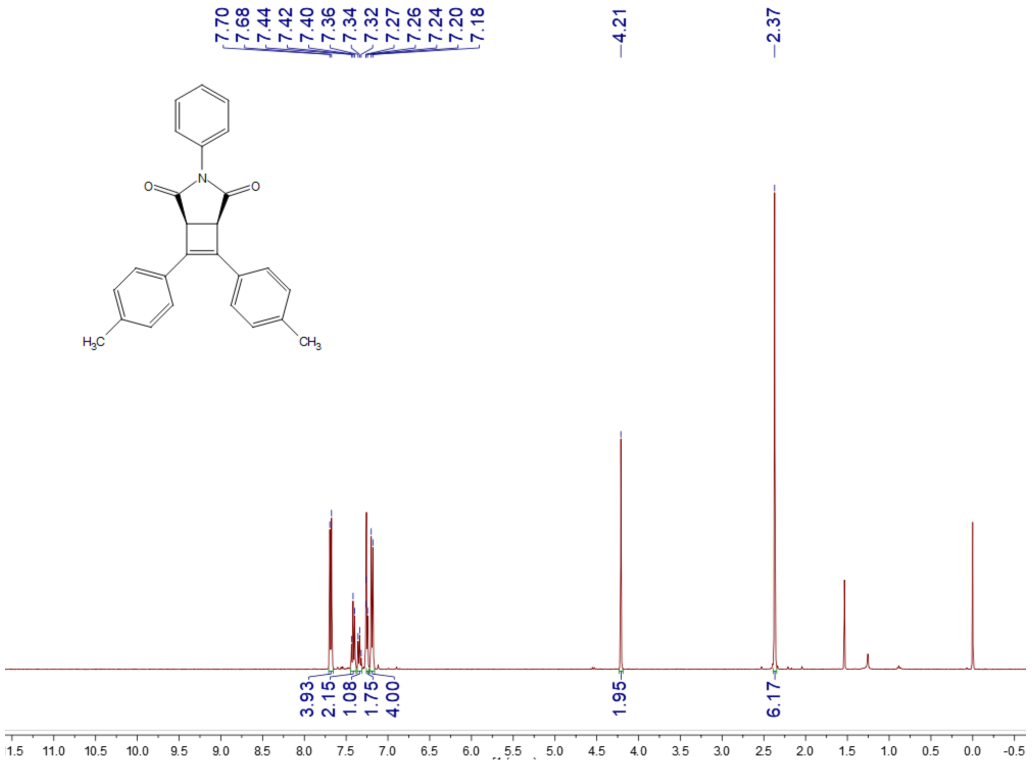

Supplementary Figure 88. ^{1}H NMR (400 MHz, $CDCl_3$) spectra of 3-phenyl-6,7-di-*p*-tolyl-3-azabicyclo[3.2.0]hept-6-ene-2,4-dione (**3am**)

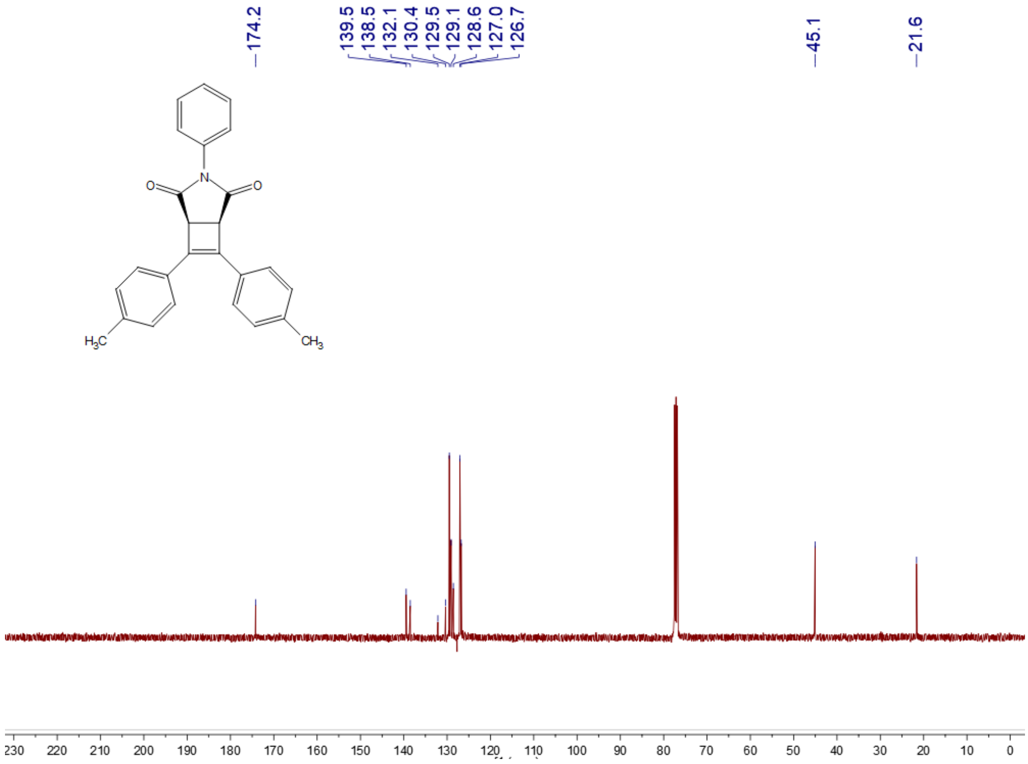

Supplementary Figure 89. ^{13}C NMR (100 MHz, $CDCl_3$) spectra of 3-phenyl-6,7-di-*p*-tolyl-3-azabicyclo[3.2.0]hept-6-ene-2,4-dione (**3am**)

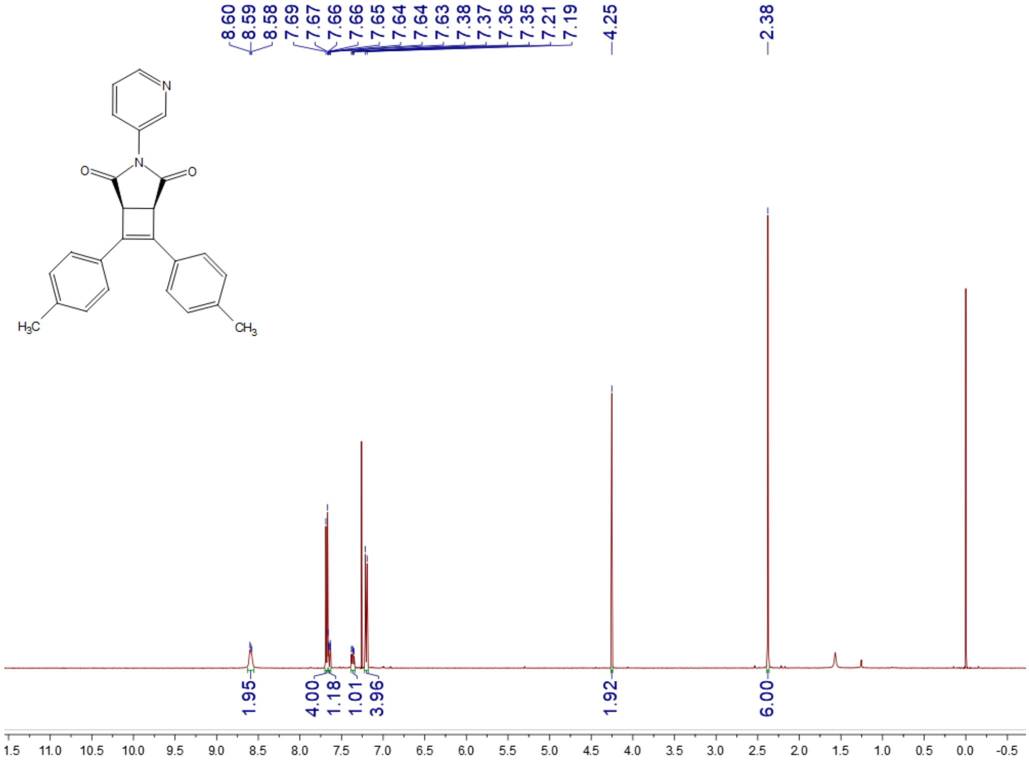

Supplementary Figure 90. ^{1}H NMR (400 MHz, $CDCl_3$) spectra of 3-(pyridin-3-yl)-6,7-di-*p*-tolyl-3-azabicyclo[3.2.0]hept-6-ene-2,4-dione (**3an**)

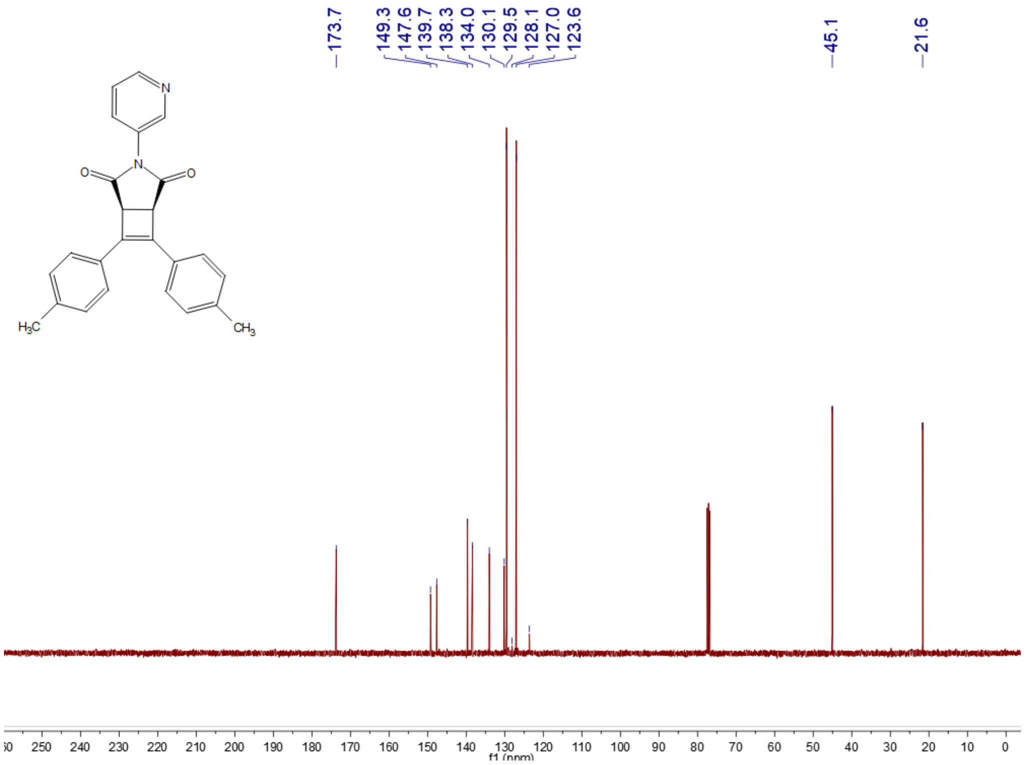

Supplementary Figure 91. ^{13}C NMR (100 MHz, $CDCl_3$) spectra of 3-(pyridin-3-yl)-6,7-di-*p*-tolyl-3-azabicyclo[3.2.0]hept-6-ene-2,4-dione (**3an**)

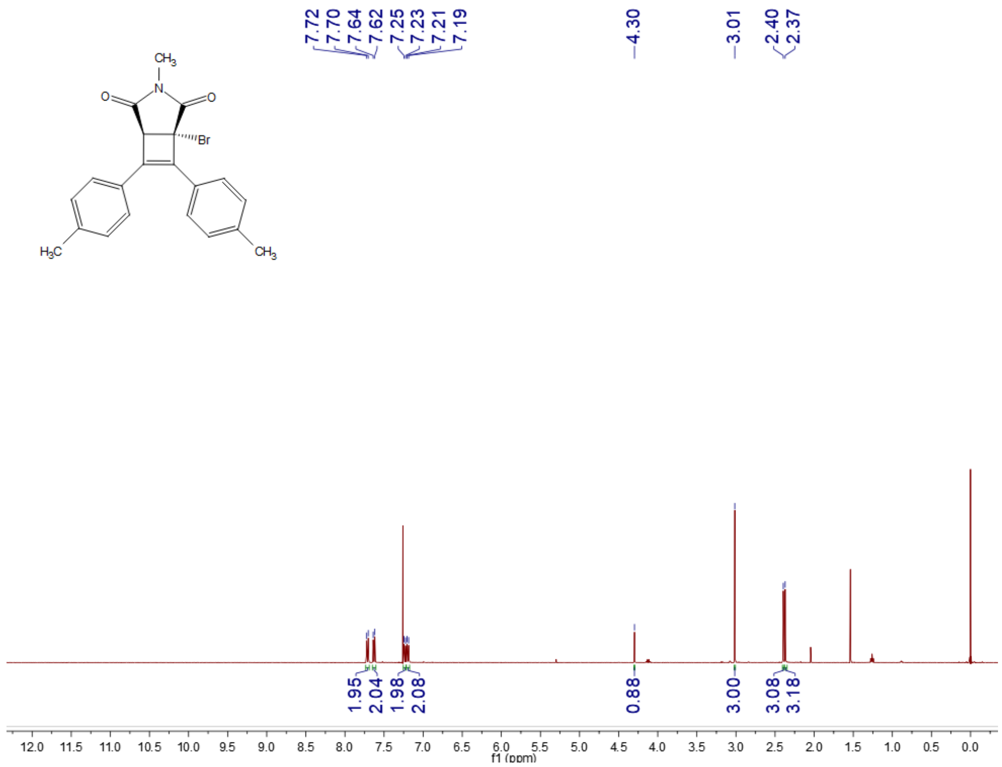

Supplementary Figure 92. ^{1}H NMR (400 MHz, $CDCl_3$) spectra of 1-bromo-3-methyl-6,7-di-*p*-tolyl-3-azabicyclo[3.2.0]hept-6-ene-2,4-dione (**3ao**)

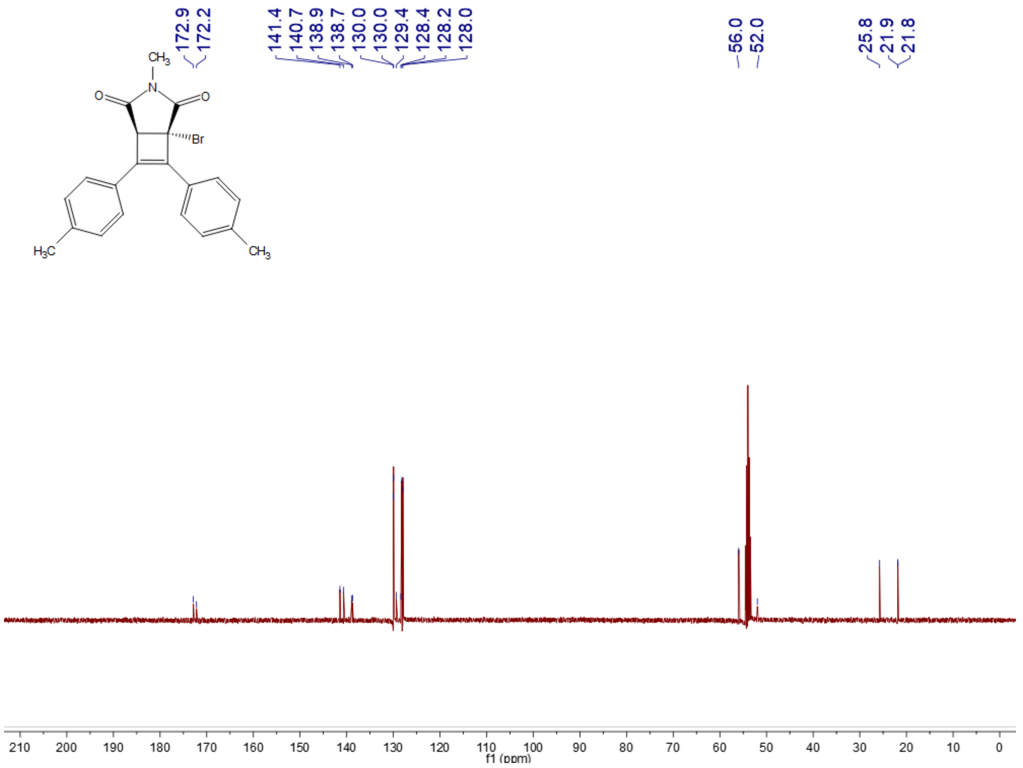

Supplementary Figure 93. ^{13}C NMR (100 MHz, CD_2Cl_2) spectra of 1-bromo-3-methyl-6,7-di-*p*-tolyl-3-azabicyclo[3.2.0]hept-6-ene-2,4-dione (**3ao**)

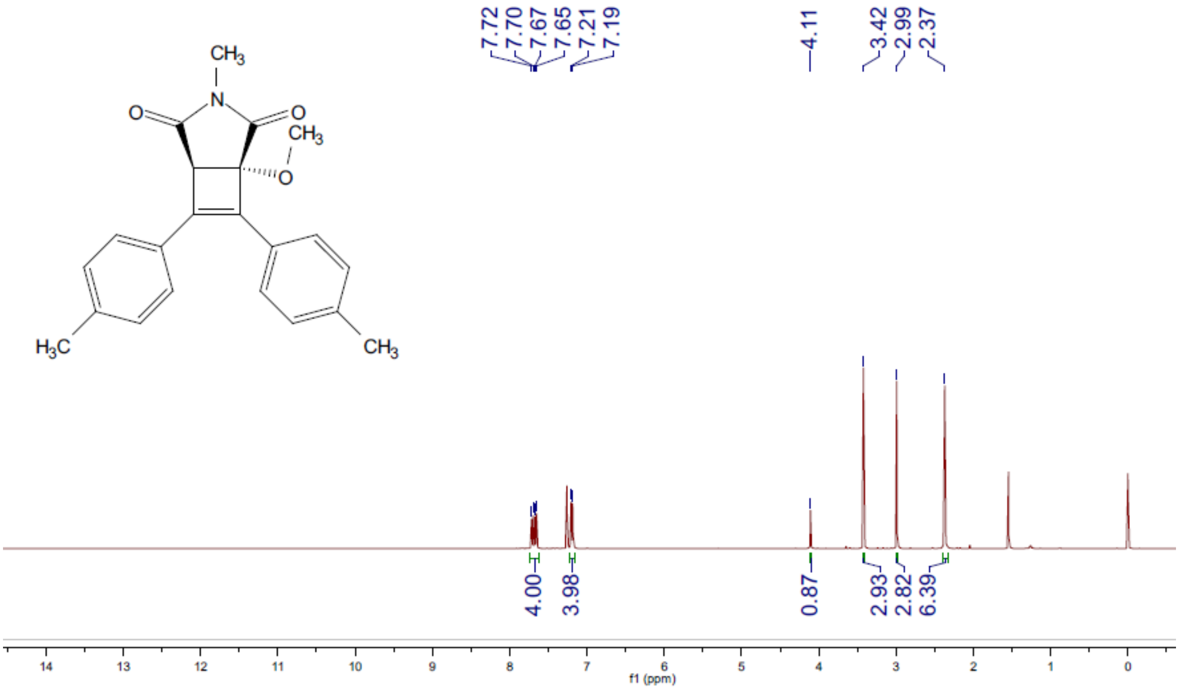

Supplementary Figure 94. ^{1}H NMR (400 MHz, $CDCl_3$) spectra of 1-methoxy-3-methyl-6,7-di-*p*-tolyl-3-azabicyclo[3.2.0]hept-6-ene-2,4-dione (**3ap**)

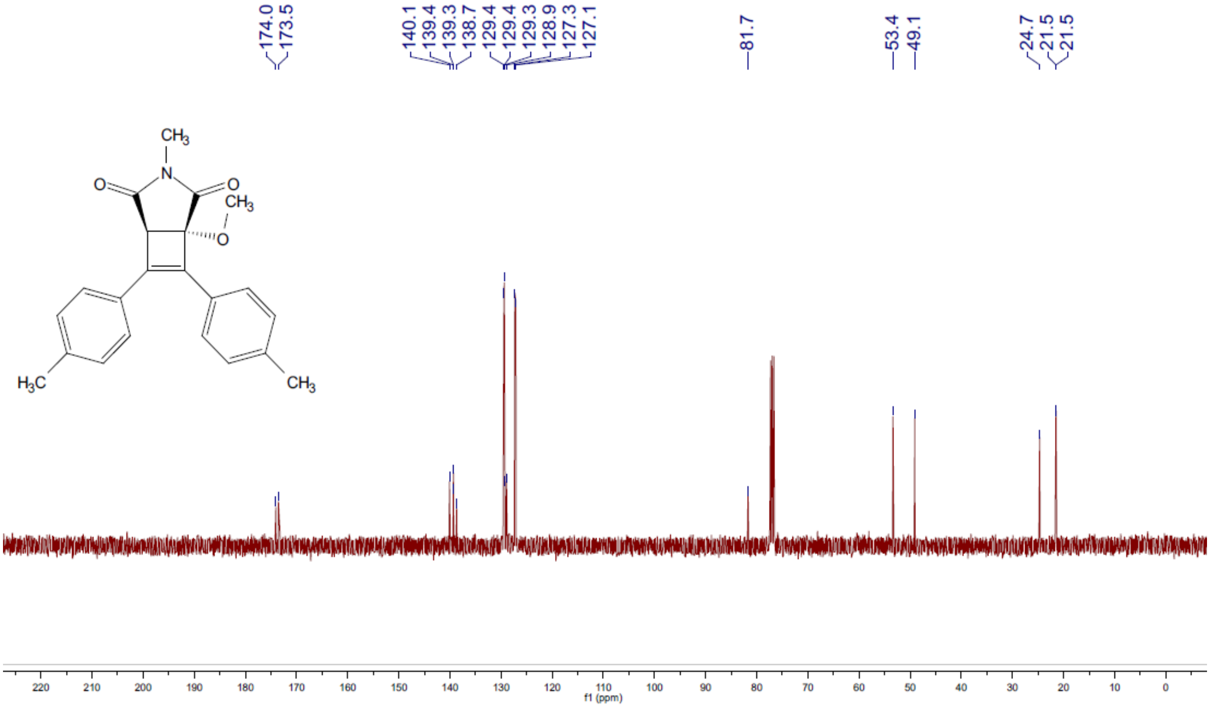

Supplementary Figure 95. ^{13}C NMR (100 MHz, $CDCl_3$) spectra of 1-methoxy-3-methyl-6,7-di-*p*-tolyl-3-azabicyclo[3.2.0]hept-6-ene-2,4-dione (**3ap**)

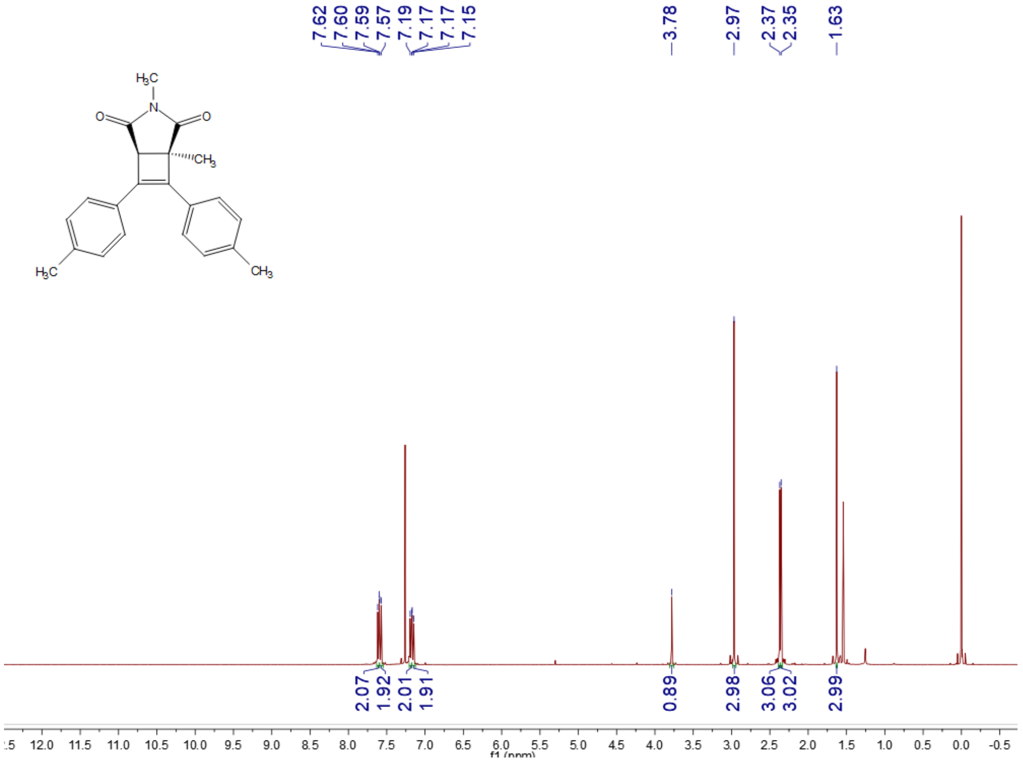

Supplementary Figure 96. ^{1}H NMR (400 MHz, $CDCl_3$) spectra of 1,3-dimethyl-6,7-di-*p*-tolyl-3-azabicyclo[3.2.0]hept-6-ene-2,4-dione (**3aq**)

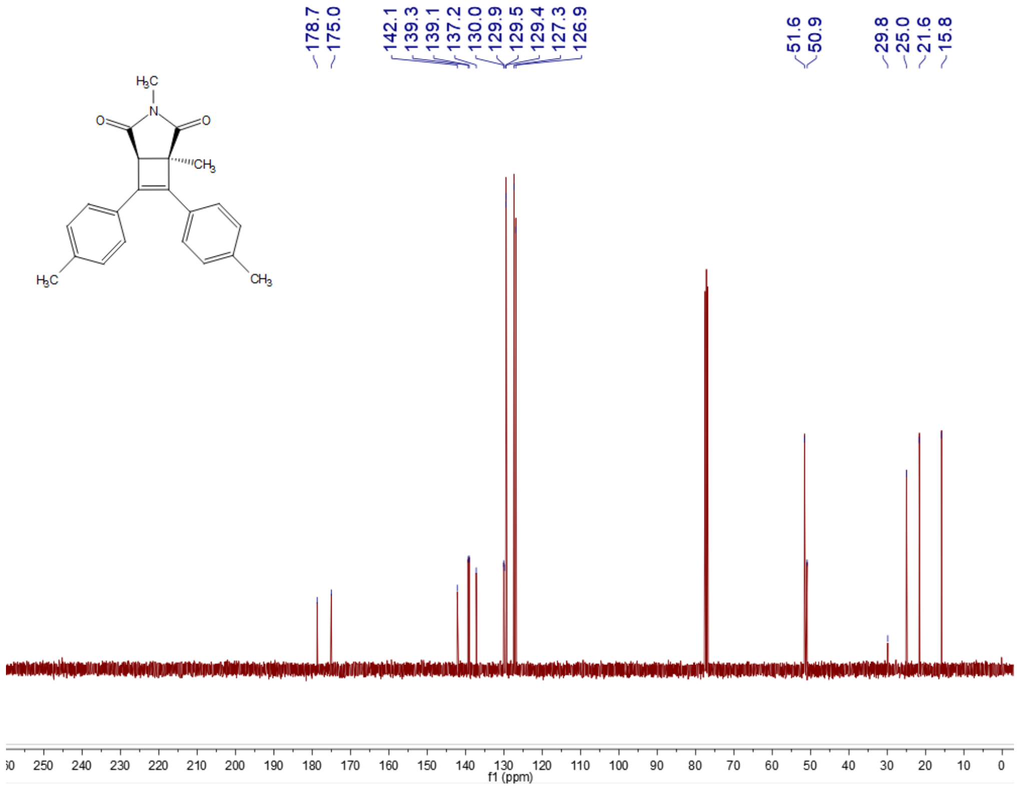

Supplementary Figure 97. ^{13}C NMR (100 MHz, $CDCl_3$) spectra of 1,3-dimethyl-6,7-di-*p*-tolyl-3-azabicyclo[3.2.0]hept-6-ene-2,4-dione (**3aq**)

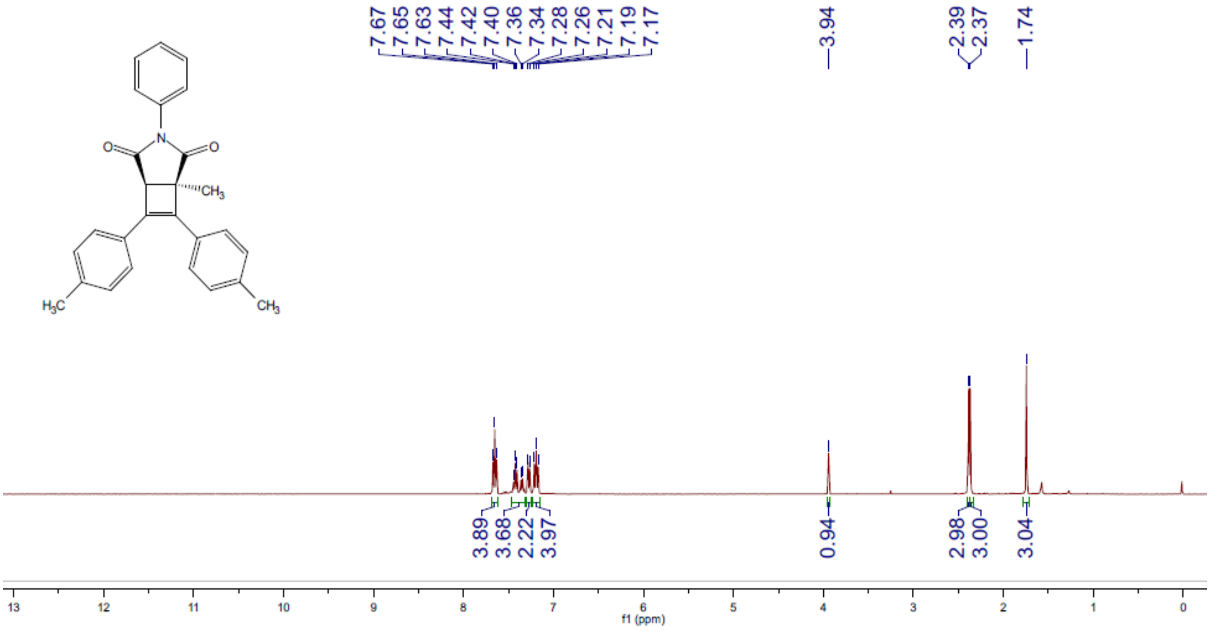

Supplementary Figure 98. ^{1}H NMR (400 MHz, $CDCl_3$) spectra of 1-methyl-3-phenyl-6,7-di-*p*-tolyl-3-azabicyclo[3.2.0]hept-6-ene-2,4-dione (**3ar**)

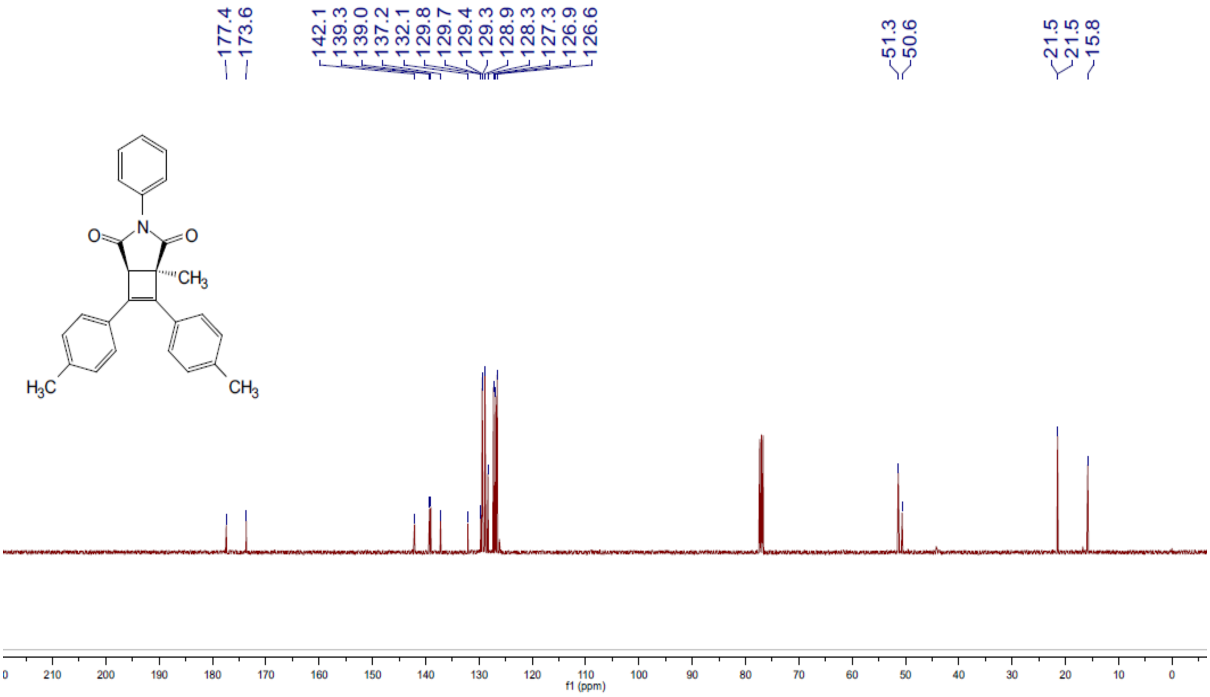

Supplementary Figure 99. ^{13}C NMR (100 MHz, $CDCl_3$) spectra of 1-methyl-3-phenyl-6,7-di-*p*-tolyl-3-azabicyclo[3.2.0]hept-6-ene-2,4-dione (**3ar**)

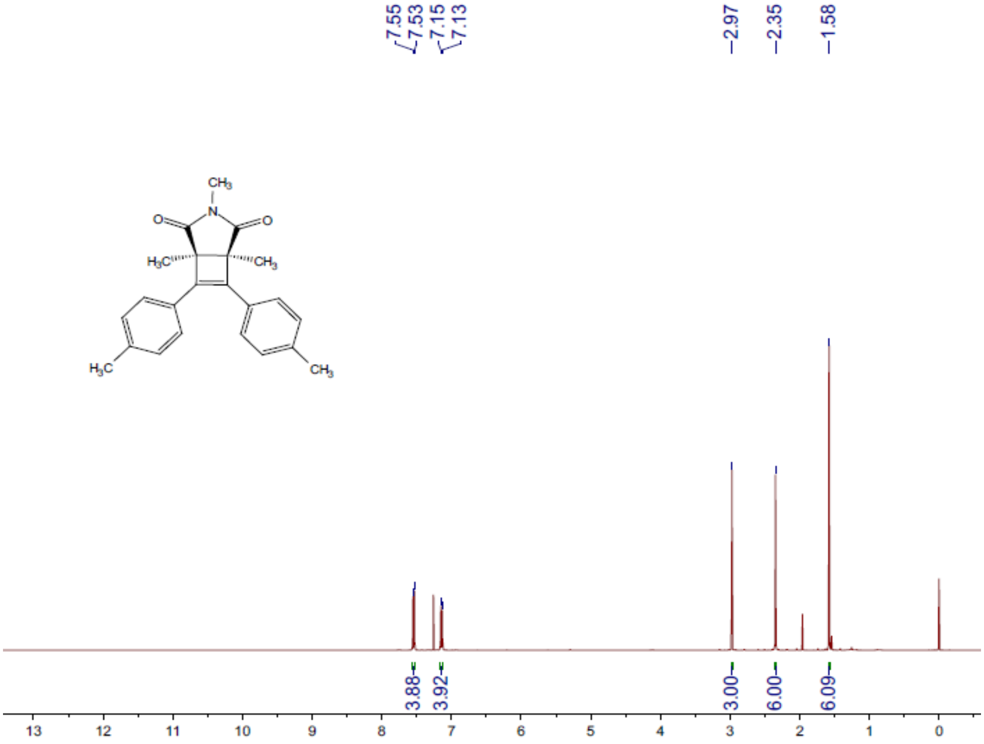

Supplementary Figure 100. ^{1}H NMR (400 MHz, $CDCl_3$) spectra of 1,3,5-trimethyl-6,7-di-*p*-tolyl-3-azabicyclo[3.2.0]hept-6-ene-2,4-dione (**3as**)

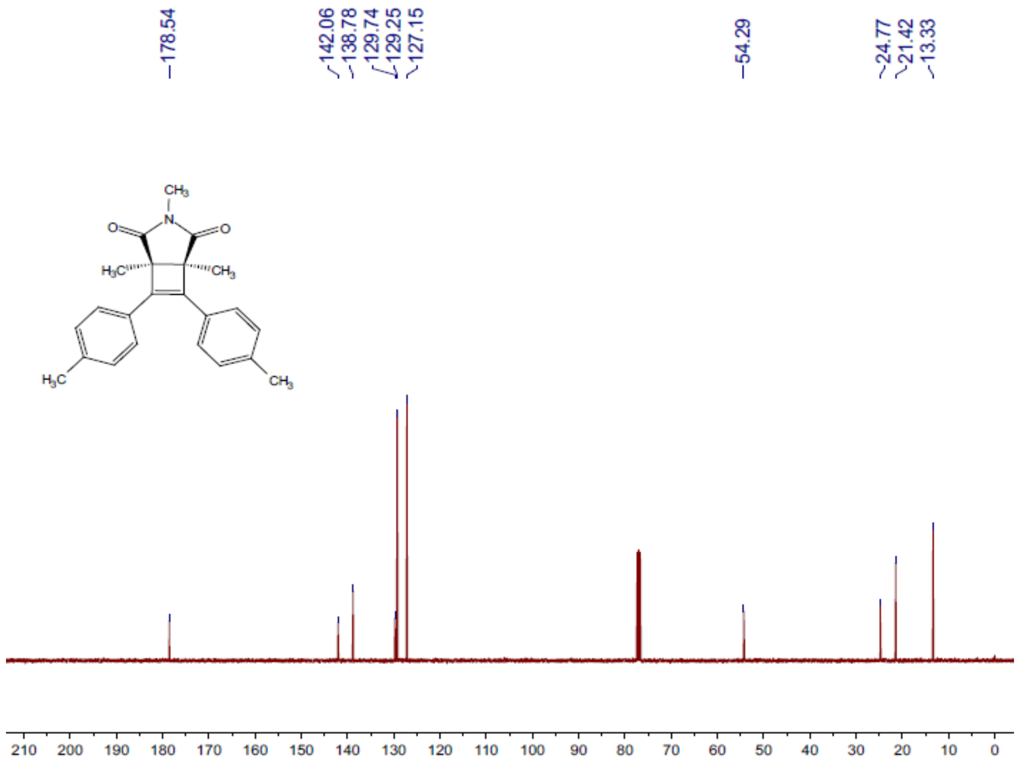

Supplementary Figure 101. ^{13}C NMR (100 MHz, $CDCl_3$) spectra of 1,3,5-trimethyl-6,7-di-*p*-tolyl-3-azabicyclo[3.2.0]hept-6-ene-2,4-dione (**3as**)

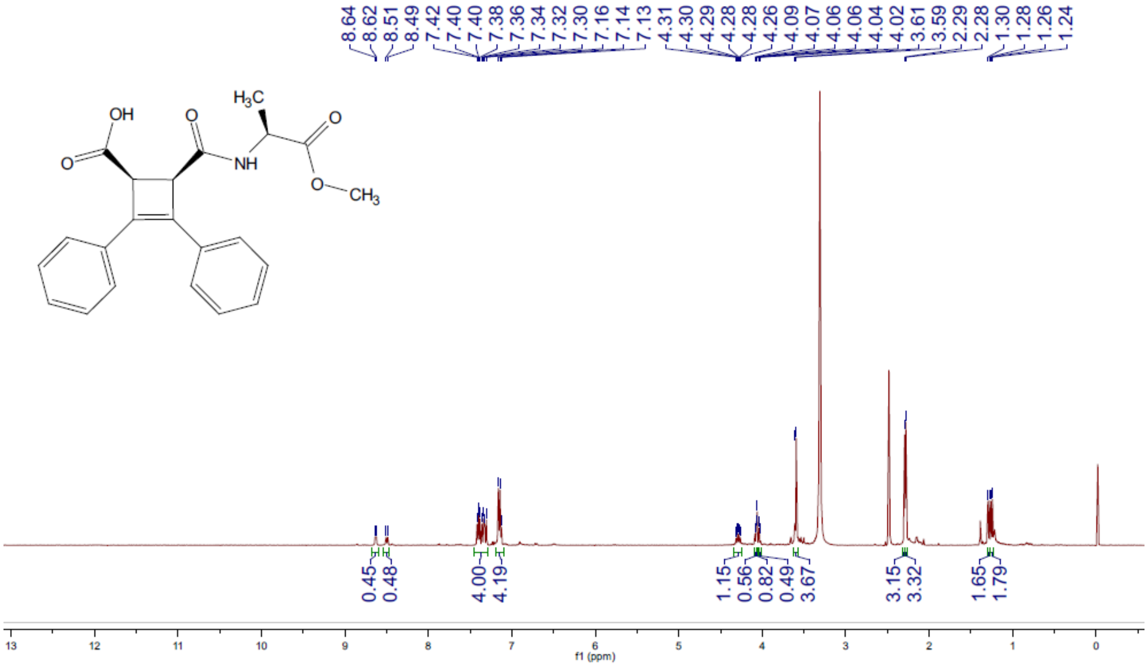

Supplementary Figure 102. ^{1}H NMR (400 MHz, $CDCl_3$) spectra of 4-(((*S*)-1-methoxy-1-oxopropan-2-yl)carbamoyl)-2,3-di-*p*-tolylcyclobut-2-ene-1-carboxylic acid (***cis*-3at**)

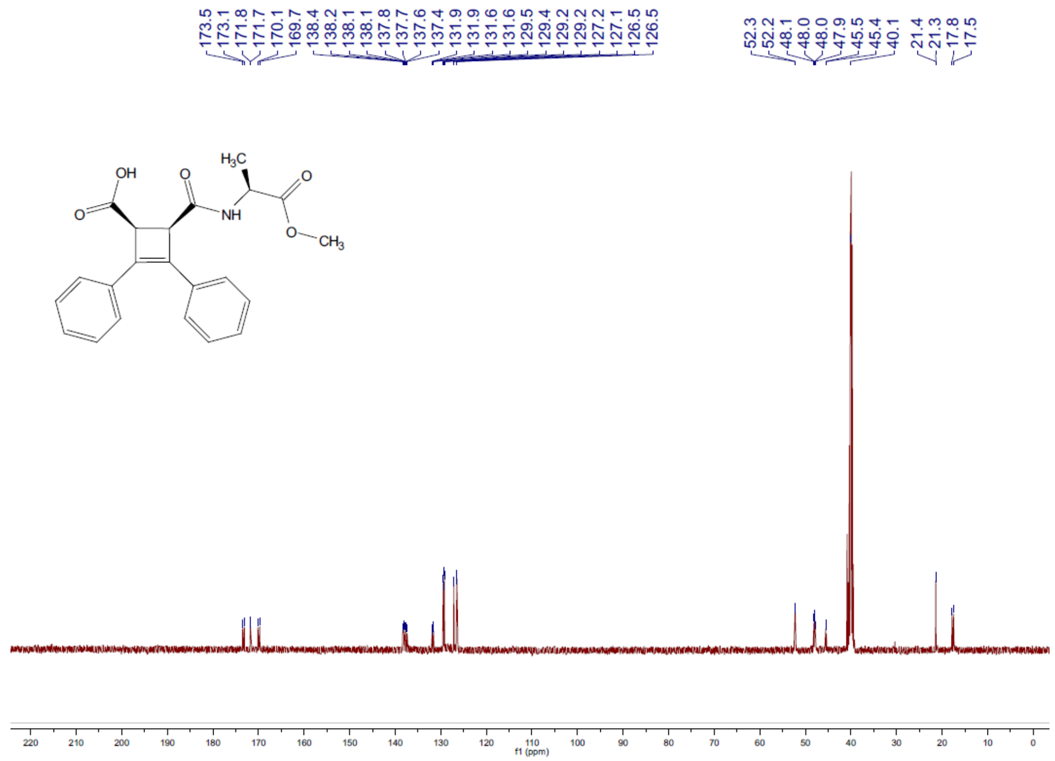

Supplementary Figure 103. ^{13}C NMR (100 MHz, $CDCl_3$) spectra of 4-(((*S*)-1-methoxy-1-oxopropan-2-yl)carbamoyl)-2,3-di-*p*-tolylcyclobut-2-ene-1-carboxylic acid (***cis*-3at**)

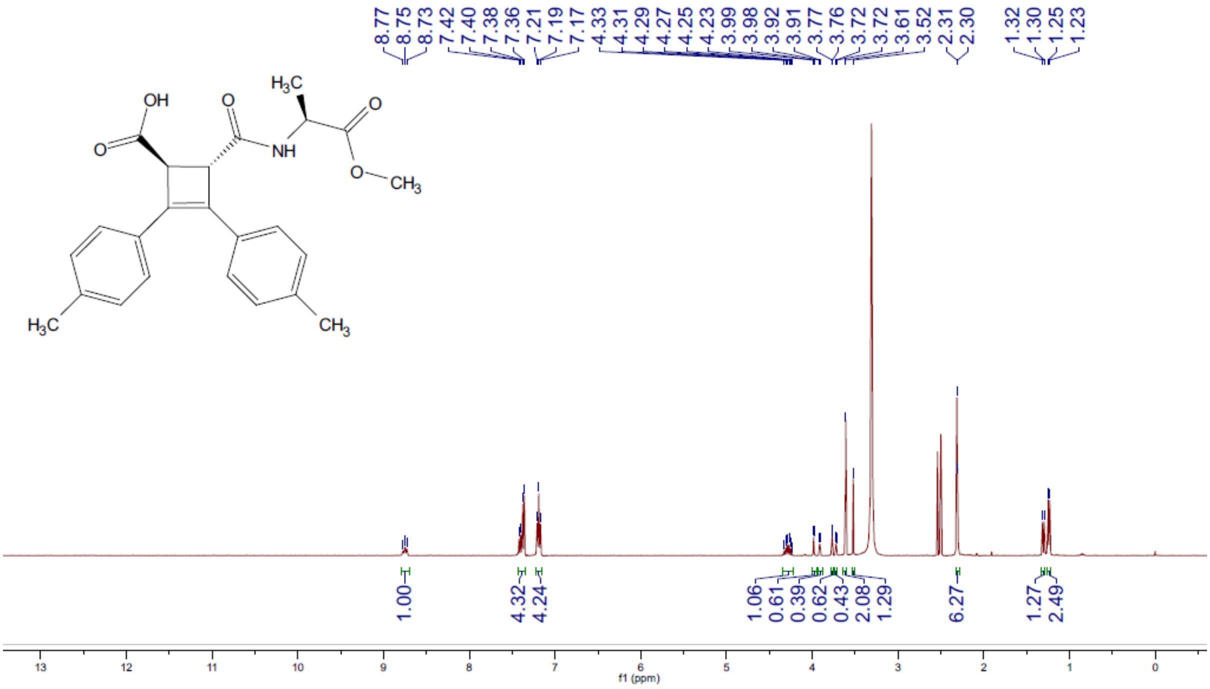

Supplementary Figure 104. ^1H NMR (400 MHz, $CDCl_3$) spectra of 4-(((*S*)-1-methoxy-1-oxopropan-2-yl)carbamoyl)-2,3-di-*p*-tolylcyclobut-2-ene-1-carboxylic acid (***trans*-3at**)

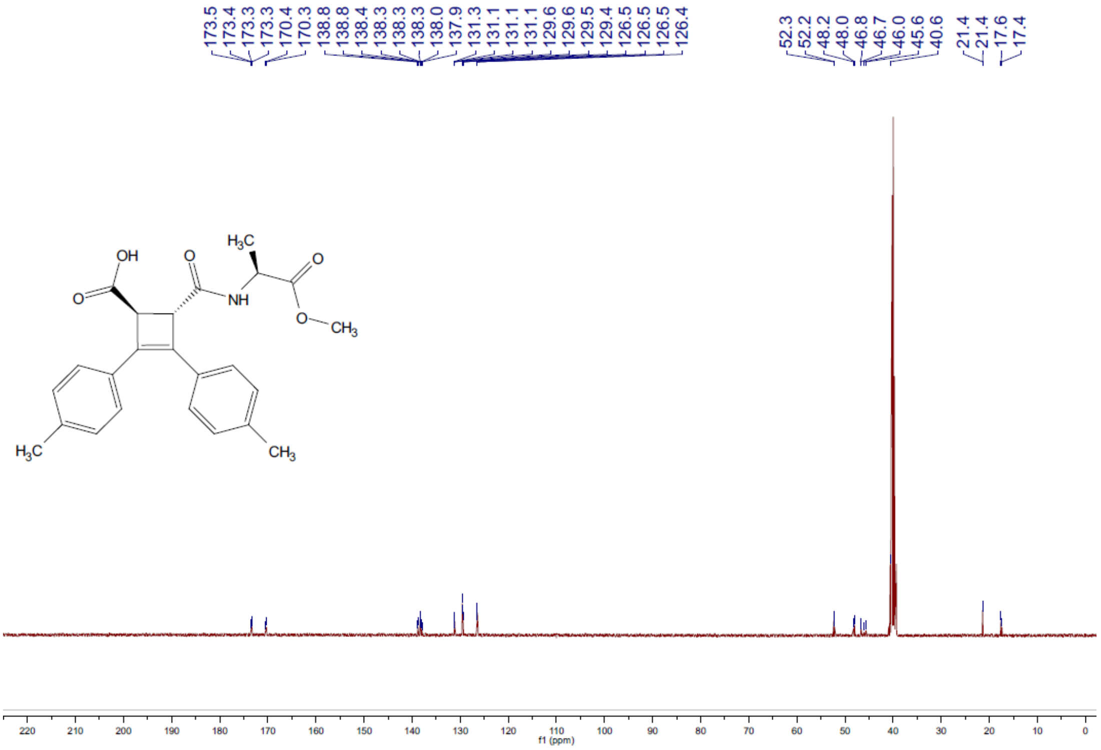

Supplementary Figure 105. ^{13}C NMR (100 MHz, $CDCl_3$) spectra of 4-(((*S*)-1-methoxy-1-oxopropan-2-yl)carbamoyl)-2,3-di-*p*-tolylcyclobut-2-ene-1-carboxylic acid (***trans*-3at**)

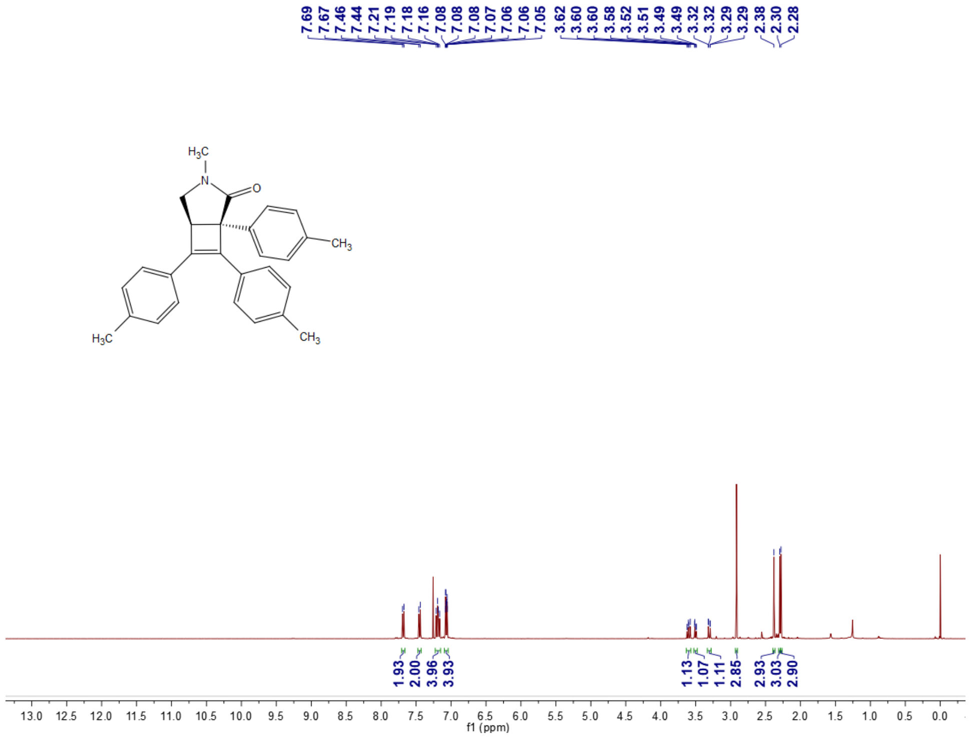

Supplementary Figure 106. ^{1}H NMR (400 MHz, $CDCl_3$) spectra of 3-methyl-1,6,7-tri-p-tolyl-3-azabicyclo[3.2.0]hept-6-en-2-one (**3av**)

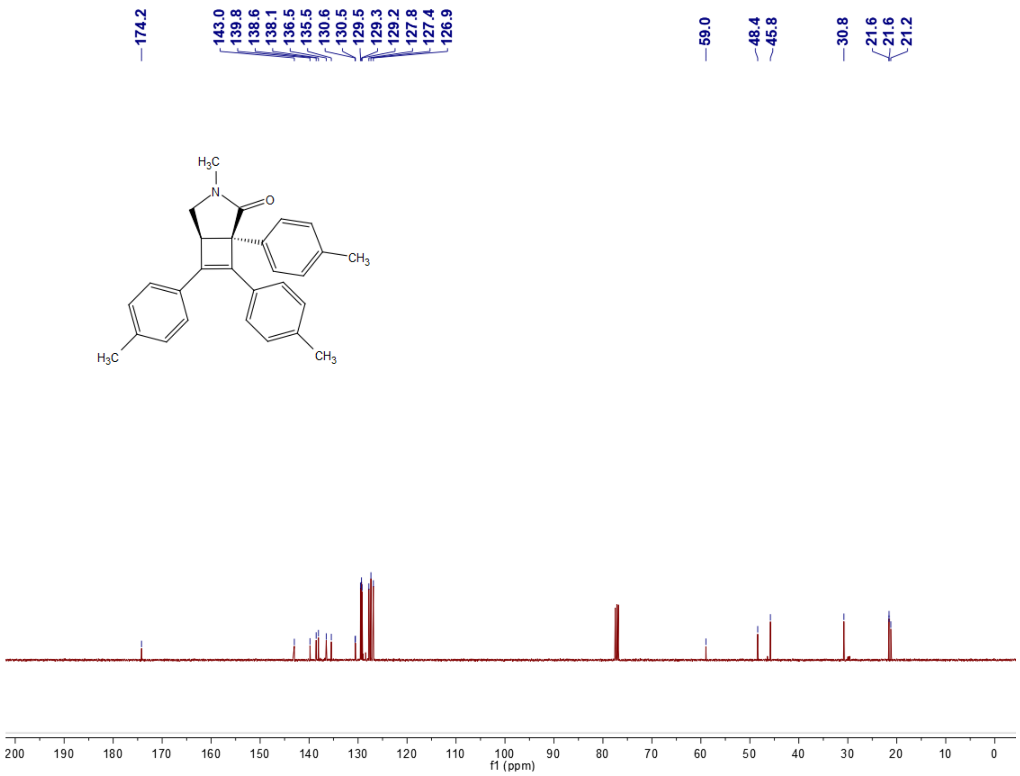

Supplementary Figure 107. ^{13}C NMR (100 MHz, $CDCl_3$) spectra of 3-methyl-1,6,7-tri-p-tolyl-3-azabicyclo[3.2.0]hept-6-en-2-one (**3av**)

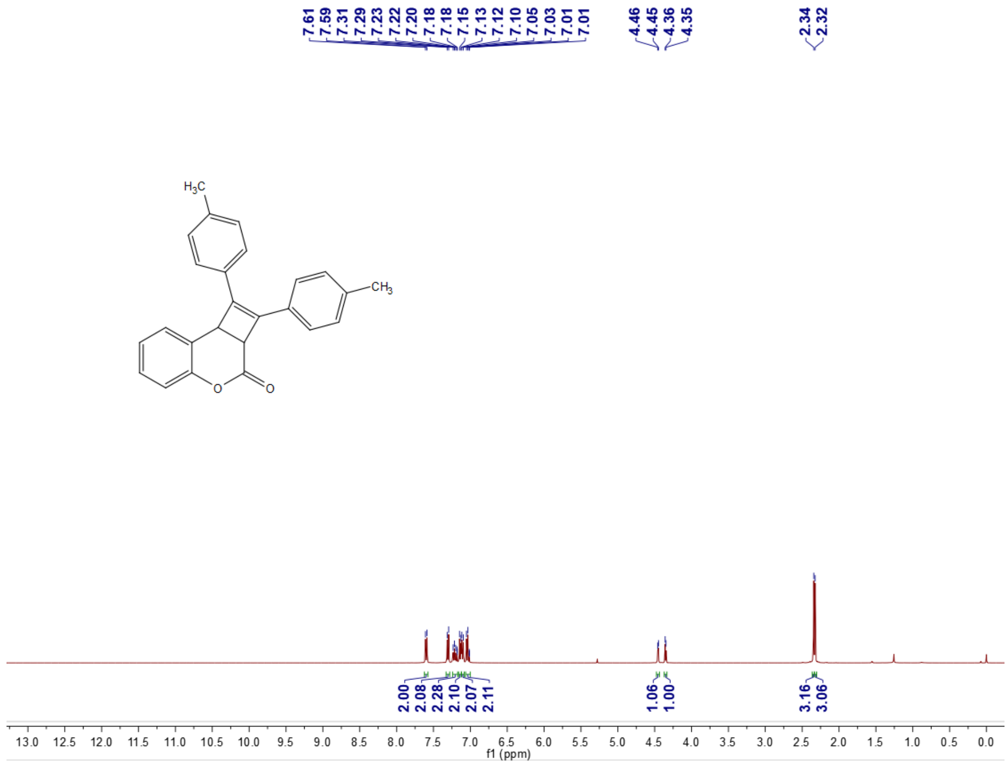

Supplementary Figure 108. ^{1}H NMR (400 MHz, $CDCl_3$) spectra of 1,2-di-*p*-tolyl-2a,8b-dihydro-3*H*-cyclobuta[*c*]chromen-3-one (**3aw**)

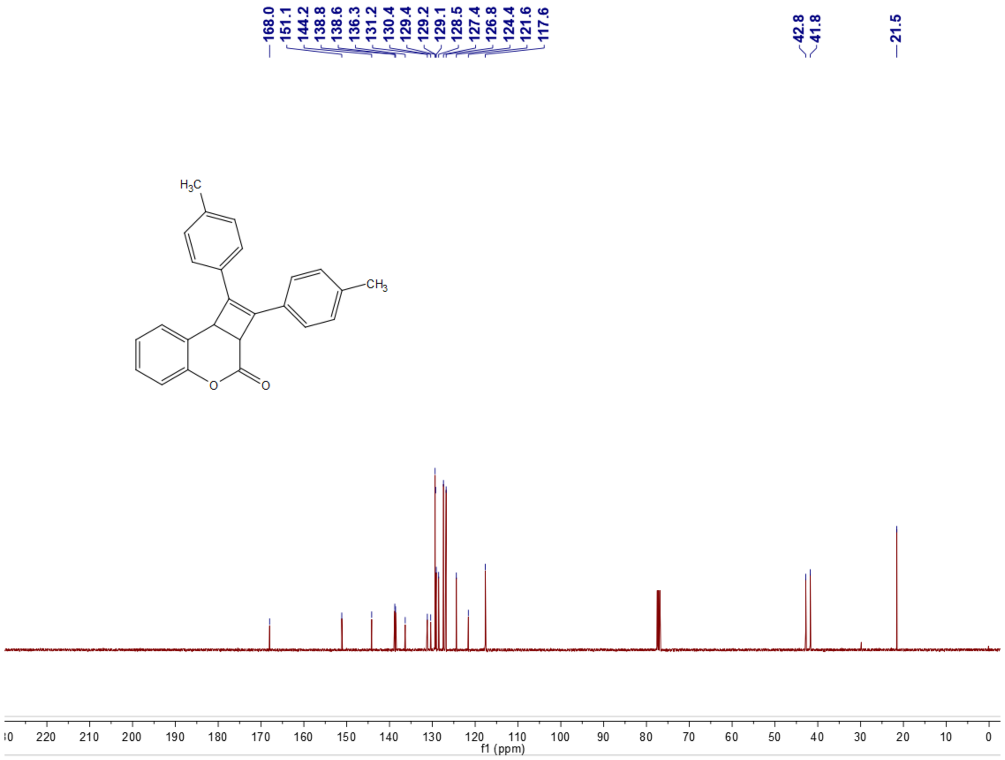

Supplementary Figure 109. ^{13}C NMR (100 MHz, $CDCl_3$) spectra of 1,2-di-*p*-tolyl-2a,8b-dihydro-3*H*-cyclobuta[*c*]chromen-3-one (**3aw**)

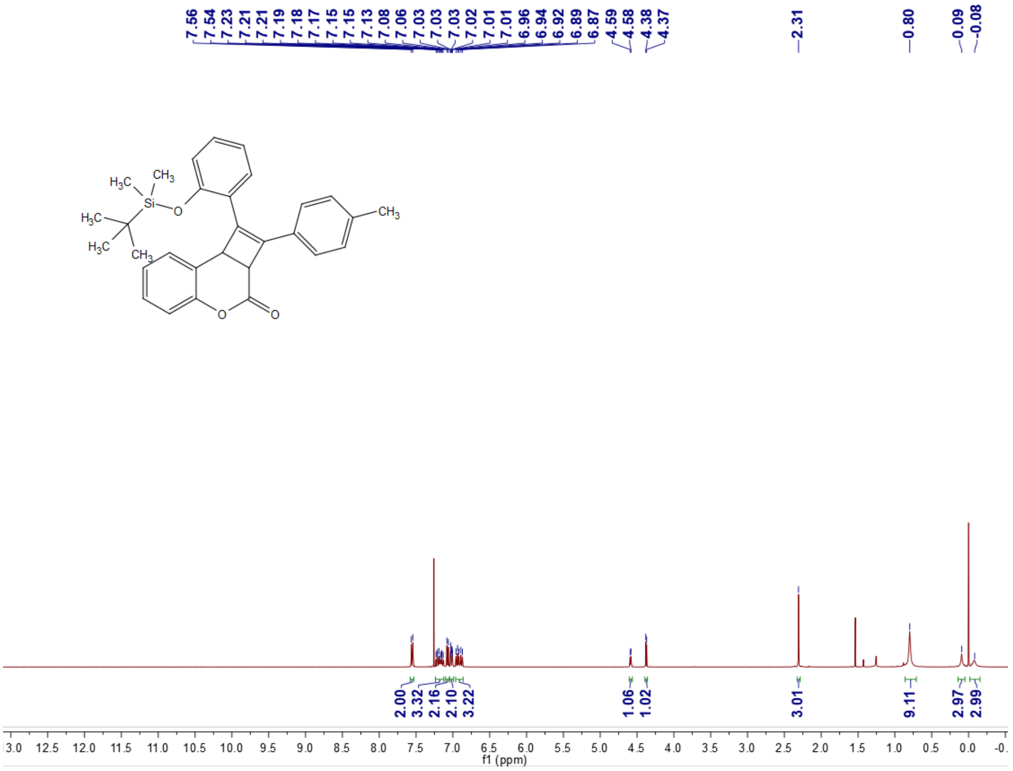

Supplementary Figure 110. ^{1}H NMR (400 MHz, $CDCl_3$) spectra of 1-(2-((*tert*-butyldimethylsilyl)oxy)phenyl)-2-(*p*-tolyl)-2a,8b-dihydro-3*H*-cyclobuta[*c*]chromen-3-one (**3ww_a**)

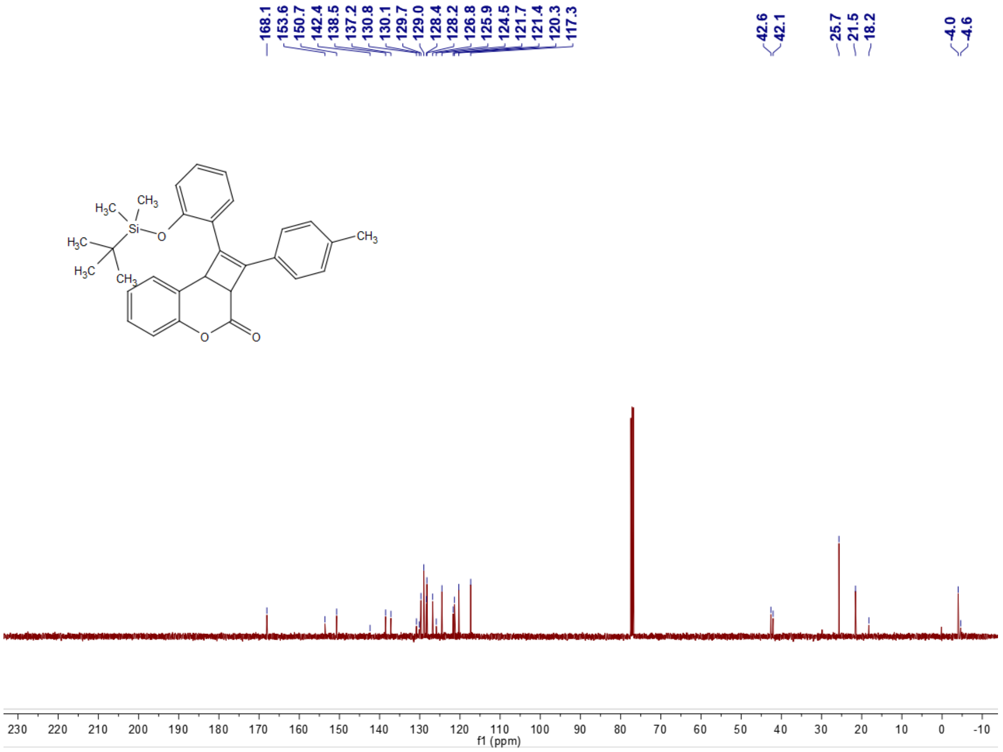

Supplementary Figure 111. ^{13}C NMR (100 MHz, $CDCl_3$) spectra of 1-(2-((*tert*-butyldimethylsilyl)oxy)phenyl)-2-(*p*-tolyl)-2a,8b-dihydro-3*H*-cyclobuta[*c*]chromen-3-one (**3ww_a**)

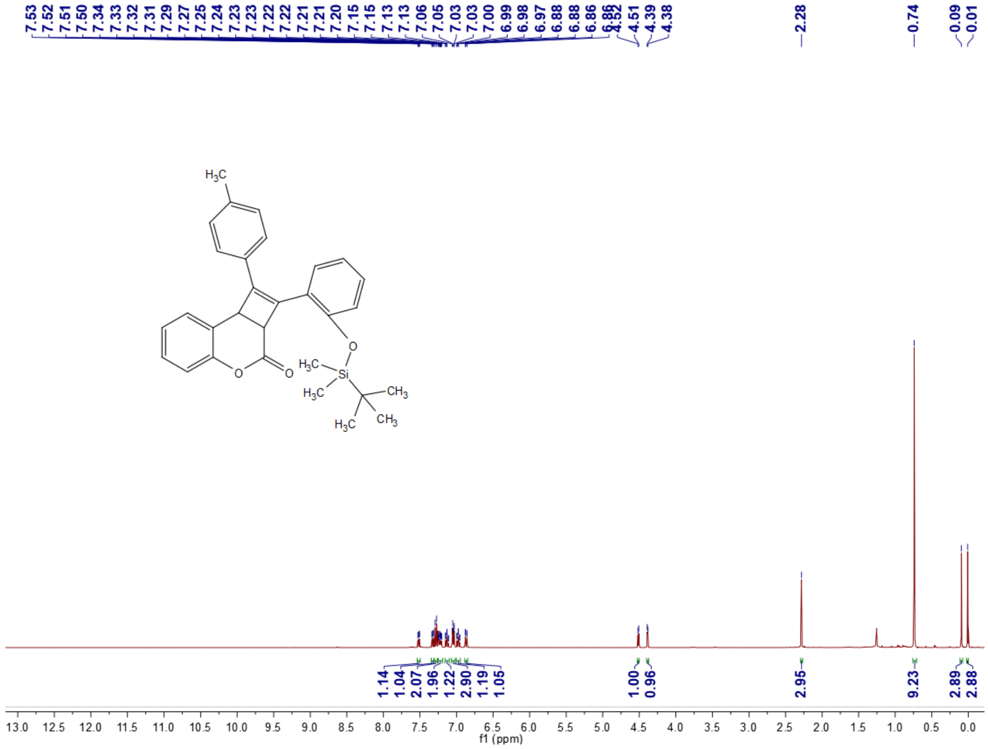

Supplementary Figure 112. ^{1}H NMR (400 MHz, $CDCl_3$) spectra of 2-(2-((*tert*-butyldimethylsilyl)oxy)phenyl)-1-(*p*-tolyl)-2a,8b-dihydro-3*H*-cyclobuta[*c*]chromen-3-one (**3ww_b**)

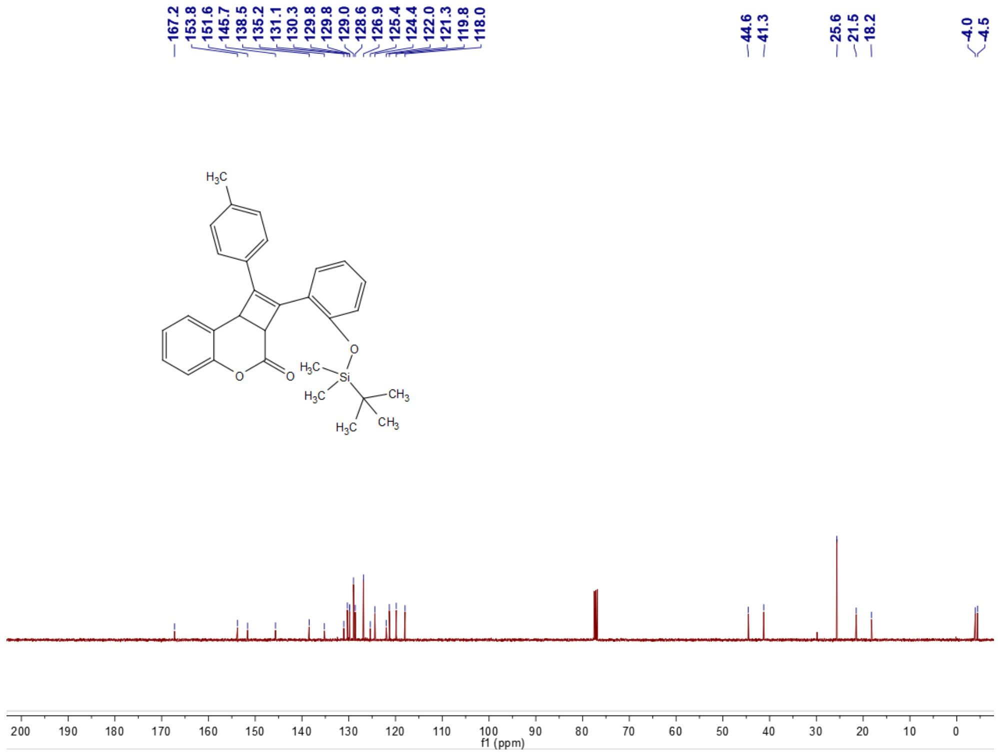

Supplementary Figure 113. ^{13}C NMR (100 MHz, $CDCl_3$) spectra of 2-(2-((*tert*-butyldimethylsilyl)oxy)phenyl)-1-(*p*-tolyl)-2a,8b-dihydro-3*H*-cyclobuta[*c*]chromen-3-one (**3ww_b**)

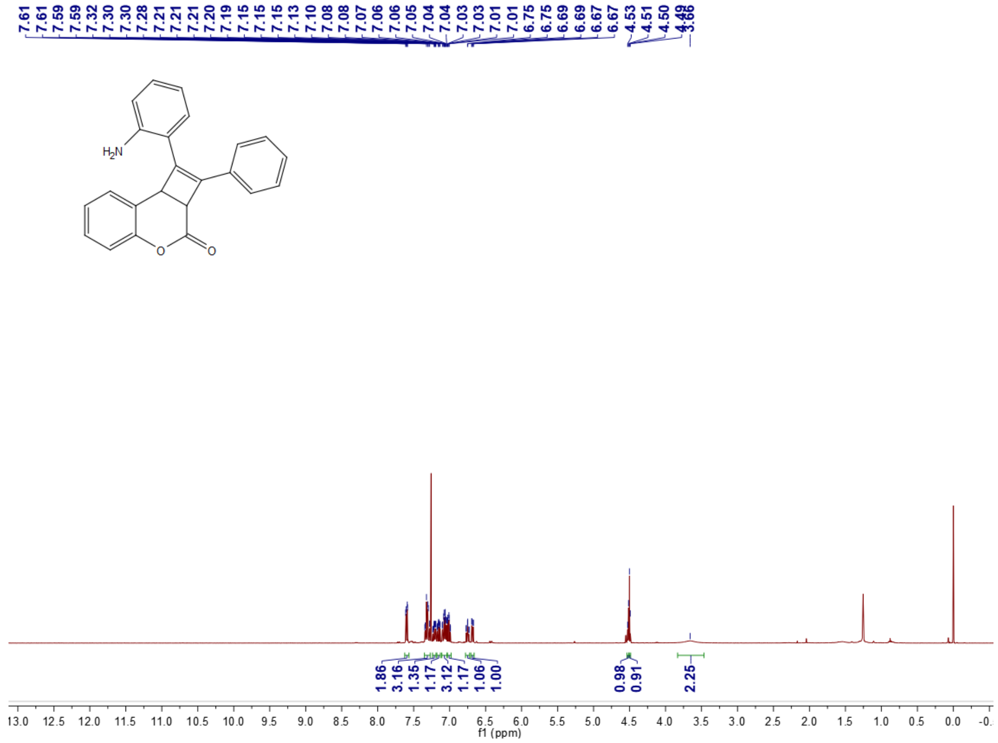

Supplementary Figure 114. ^{1}H NMR (400 MHz, $CDCl_3$) spectra of 1-(2-aminophenyl)-2-phenyl-2a,8b-dihydro-3*H*-cyclobuta[*c*]chromen-3-one (**3xw**)

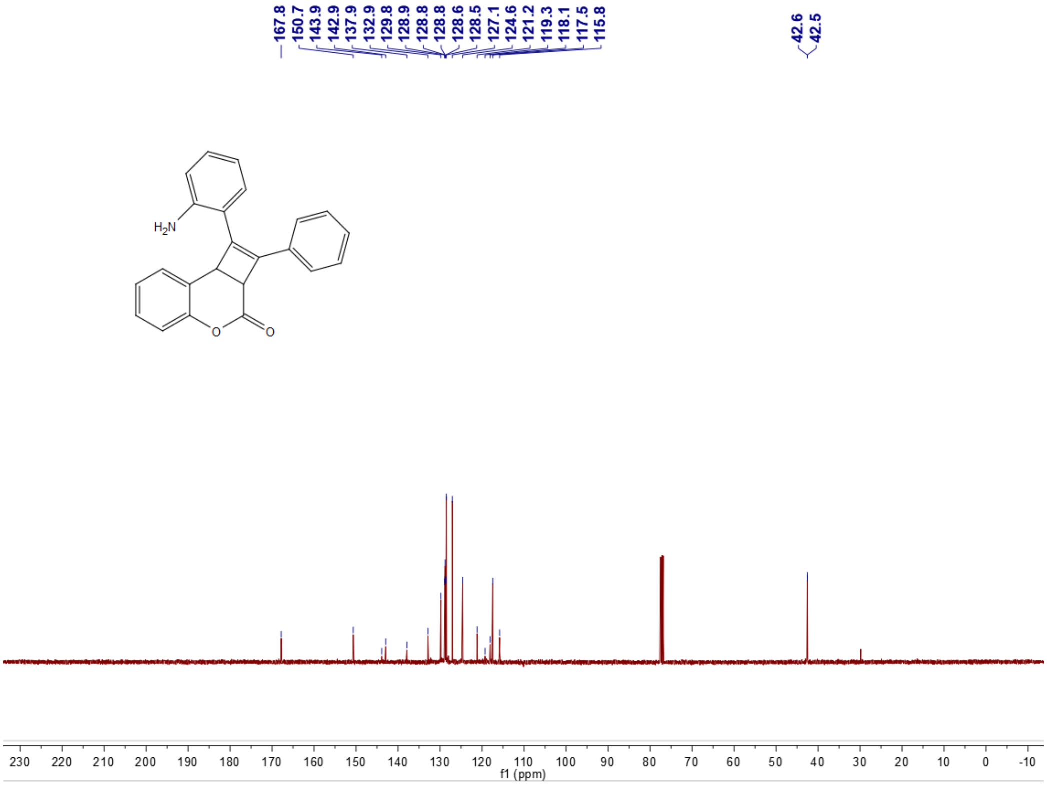

Supplementary Figure 115. ^{13}C NMR (100 MHz, $CDCl_3$) spectra of 1-(2-aminophenyl)-2-phenyl-2a,8b-dihydro-3*H*-cyclobuta[*c*]chromen-3-one (**3xw**)

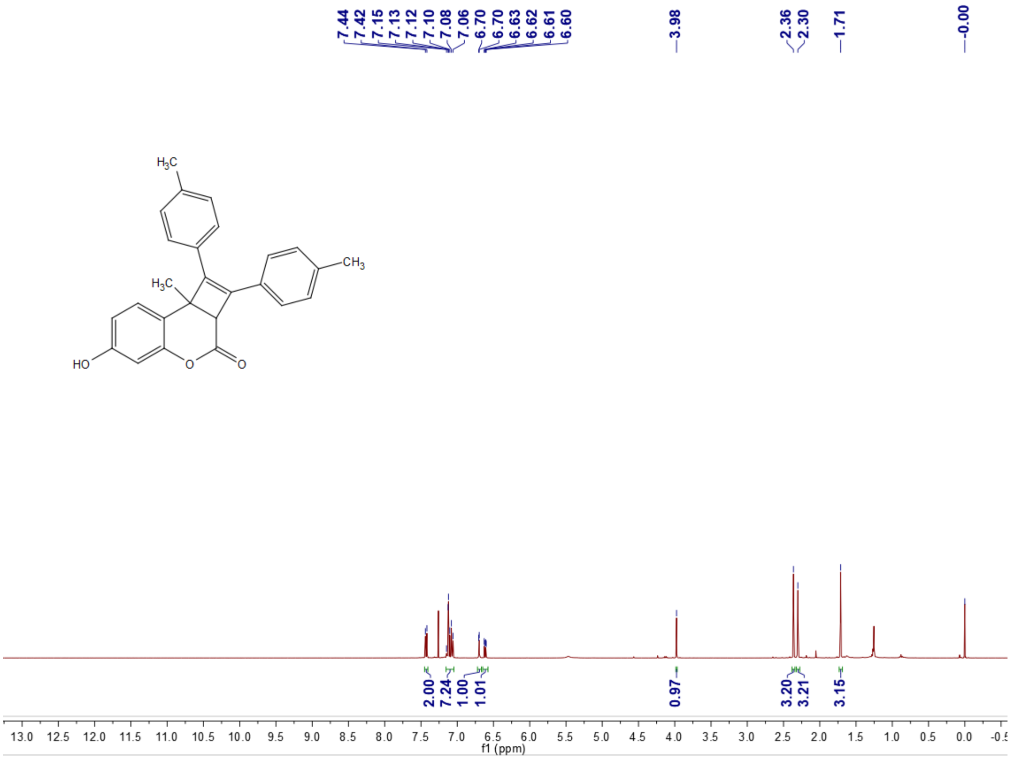

Supplementary Figure 116. ^{1}H NMR (400 MHz, $CDCl_3$) spectra of 6-hydroxy-8b-methyl-1,2-di-*p*-tolyl-2a,8b-dihydro-3*H*-cyclobuta[*c*]chromen-3-one (**3ax**)

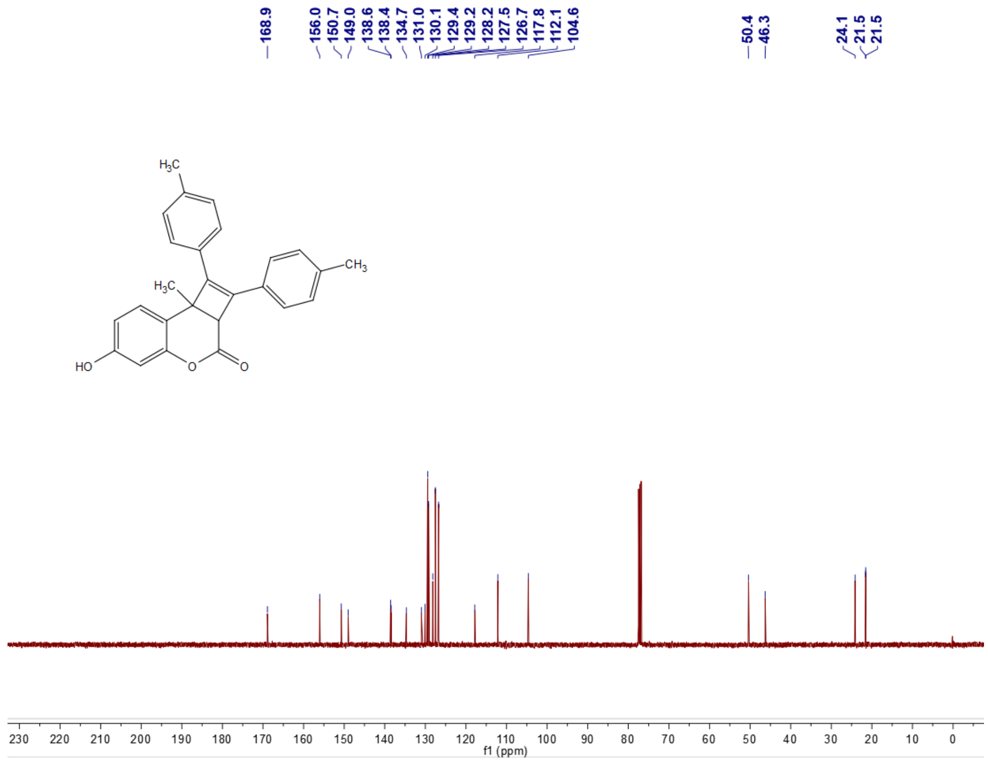

Supplementary Figure 117. ^{13}C NMR (100 MHz, CDCl$_3$) spectra of 6-hydroxy-8b-methyl-1,2-di-*p*-tolyl-2a,8b-dihydro-3*H*-cyclobuta[*c*]chromen-3-one (**3ax**)

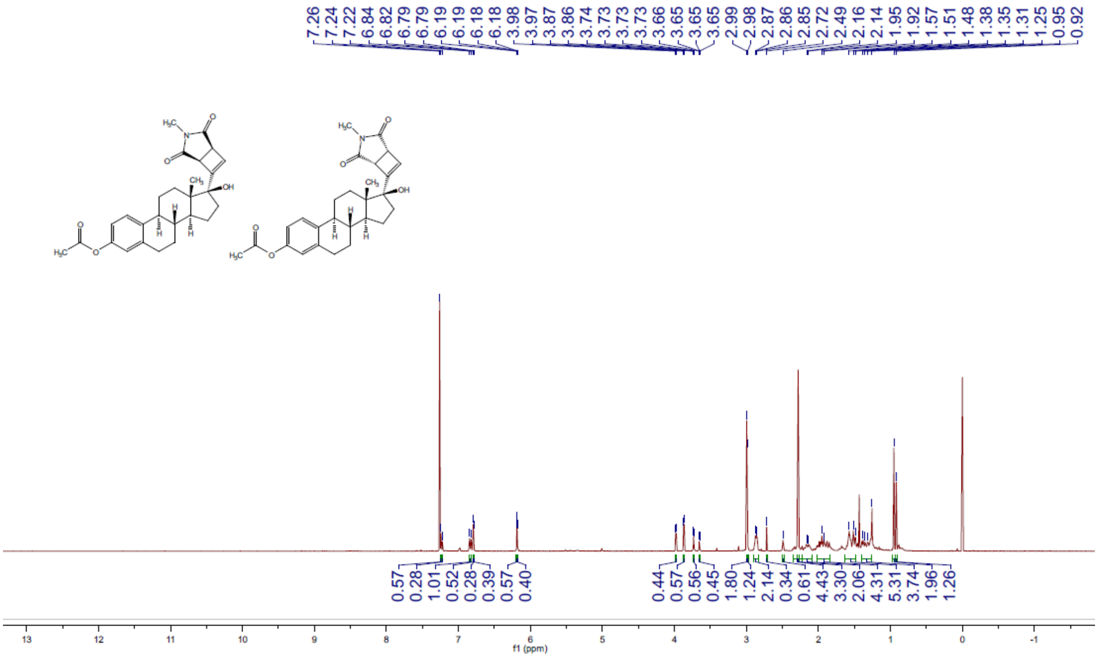

Supplementary Figure 118. ^{1}H NMR (400 MHz, CDCl$_3$) spectra of (8*R*,9*S*,13*S*,14*S*,17*S*)-17-hydroxy-13-methyl-17-(3-methyl-2,4-dioxo-3-azabicyclo[3.2.0]hept-6-en-6-yl)-7,8,9,11,12,13,14,15,16,17-decahydro-6*H*-cyclopenta[*a*]phenanthren-3-yl acetate (**3wa**)

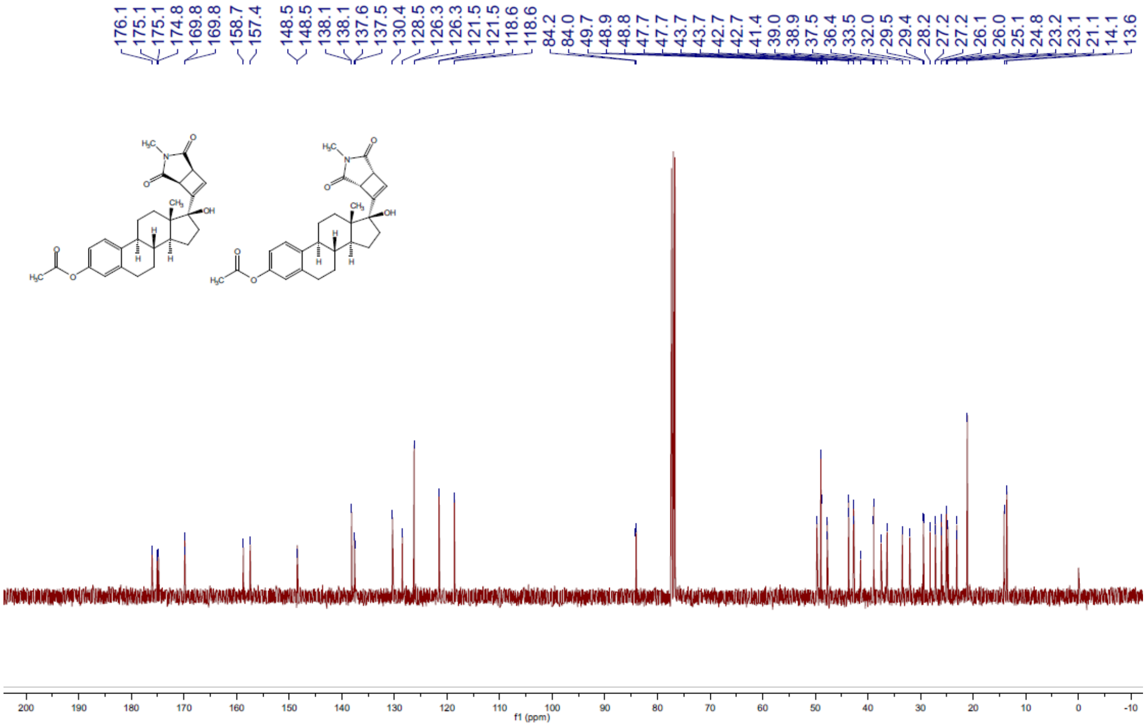

Supplementary Figure 119. ^{13}C NMR (100 MHz, $CDCl_3$) spectra of (8*R*,9*S*,13*S*,14*S*,17*S*)-17-hydroxy-13-methyl-17-(3-methyl-2,4-dioxo-3-azabicyclo[3.2.0]hept-6-en-6-yl)-7,8,9,11,12,13,14,15,16,17-decahydro-6*H*-cyclopenta[*a*]phenanthren-3-yl acetate (**3wa**)

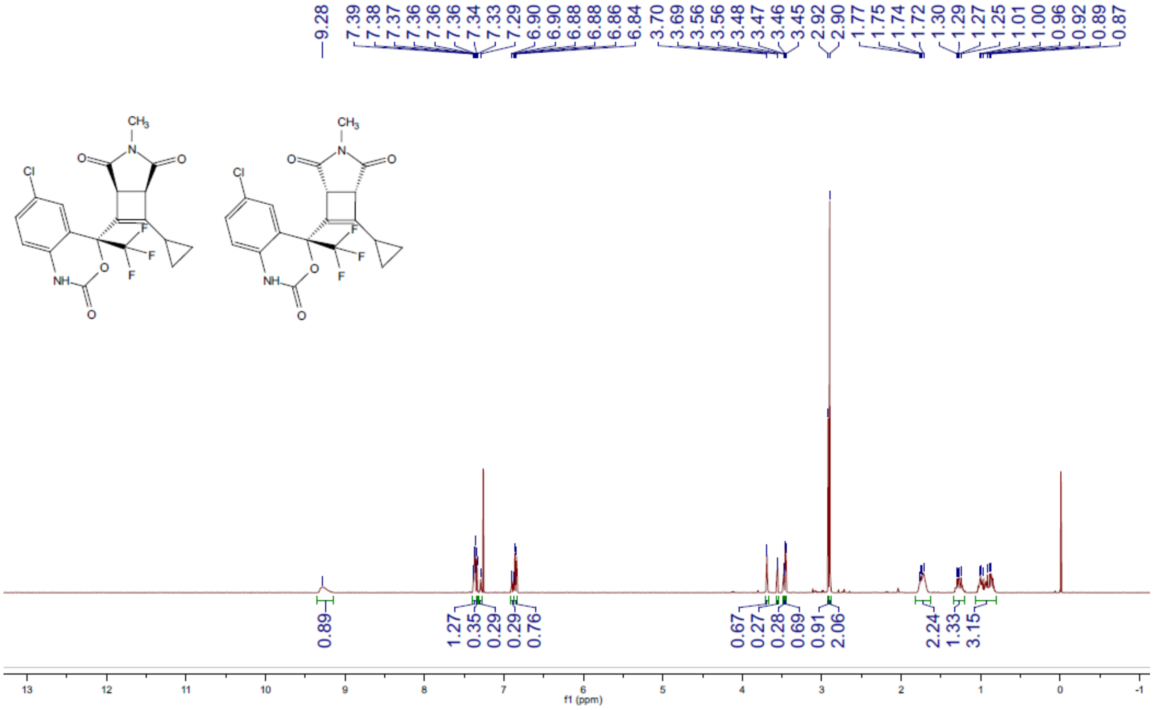

Supplementary Figure 120. ^{1}H NMR (400 MHz, $CDCl_3$) spectra of 6-(6-chloro-2-oxo-4-(trifluoromethyl)-1,4-dihydro-2*H*-benzo[*d*][1,3]oxazin-4-yl)-7-cyclopropyl-3-methyl-3-azabicyclo[3.2.0]hept-6-ene-2,4-dione (**3xa**)

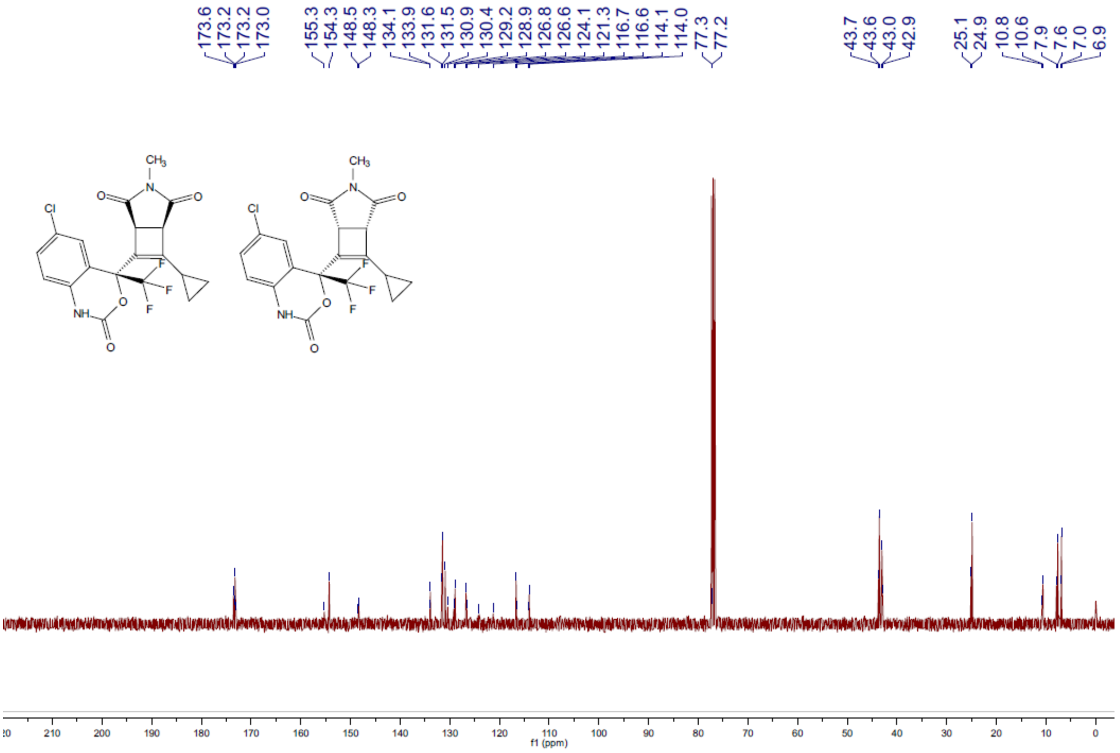

Supplementary Figure 121. ^{13}C NMR (100 MHz, $CDCl_3$) spectra of 6-(6-chloro-2-oxo-4-(trifluoromethyl)-1,4-dihydro-2*H*-benzo[*d*][1,3]oxazin-4-yl)-7-cyclopropyl-3-methyl-3-azabicyclo[3.2.0]hept-6-ene-2,4-dione (**3xa**)

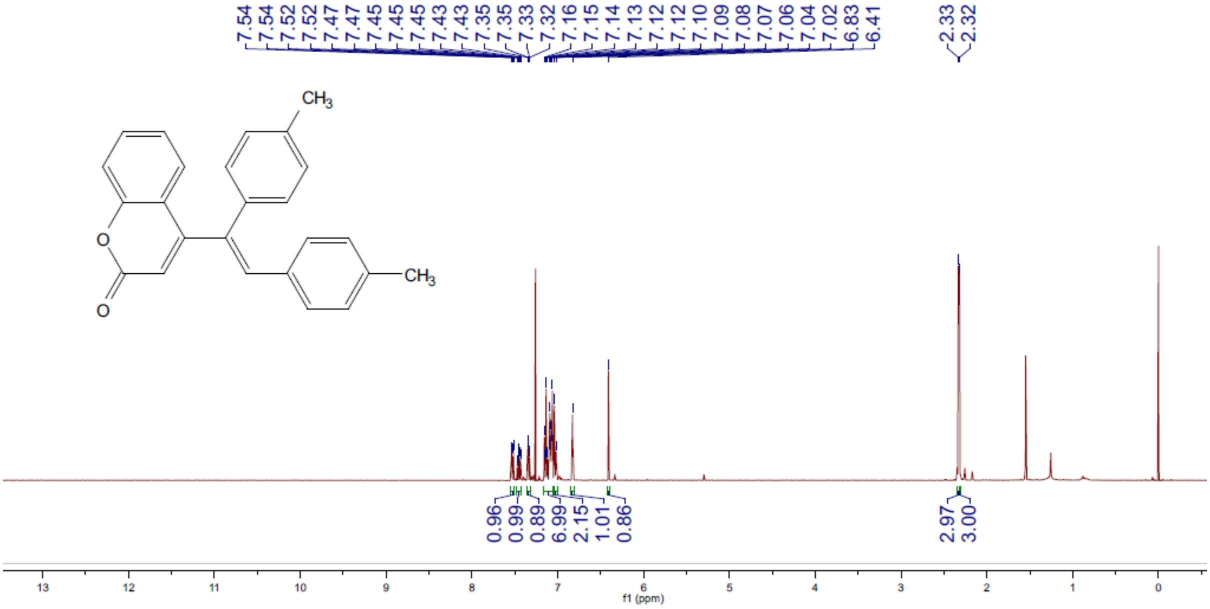

Supplementary Figure 122. ^{1}H NMR (400 MHz, $CDCl_3$) spectra of (*E*)-4-(1,2-di-*p*-tolylvinyl)-2*H*-chromen-2-one (**(*E*)-6a**)

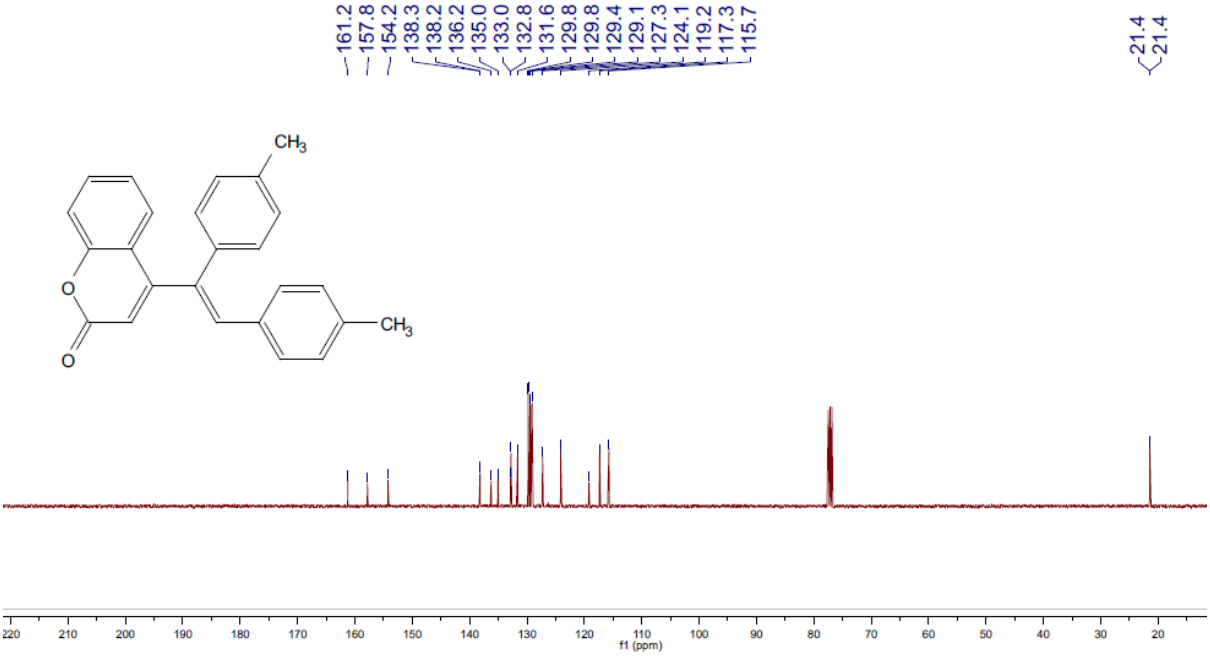

Supplementary Figure 123. ^{13}C NMR (100 MHz, $CDCl_3$) spectra of (*E*)-4-(1,2-di-*p*-tolylvinyl)-2*H*-chromen-2-one (**(*E*)-6a**)

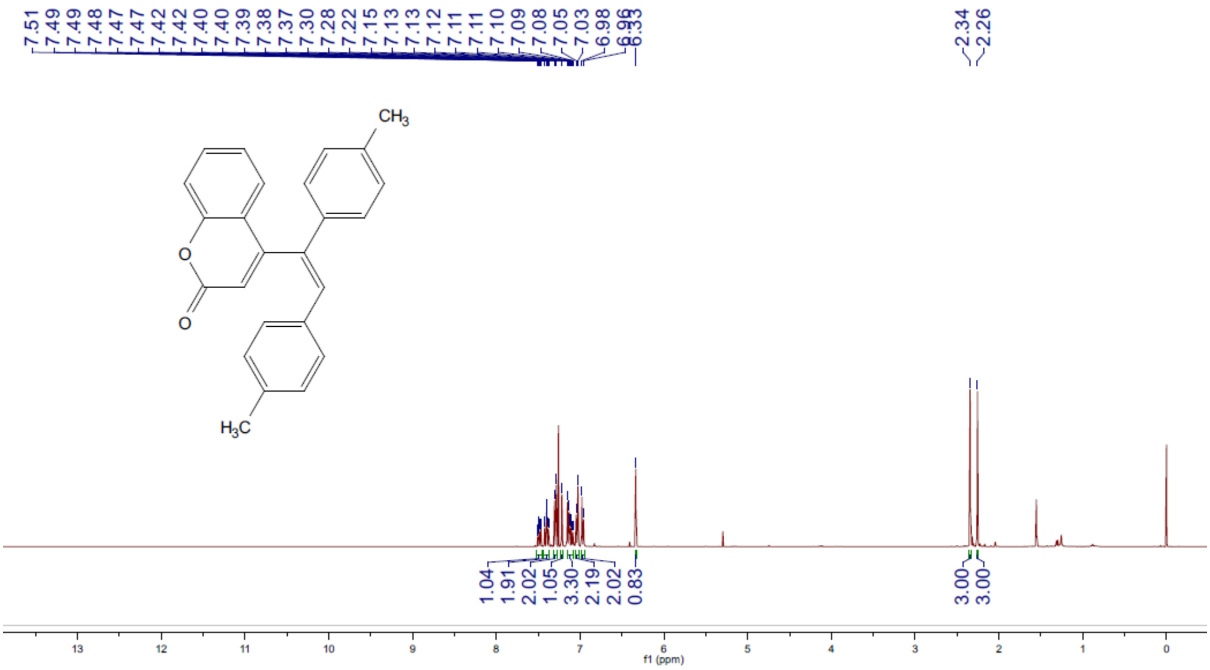

Supplementary Figure 124. ^{1}H NMR (400 MHz, $CDCl_3$) spectra of (*Z*)-4-(1,2-di-*p*-tolylvinyl)-2*H*-chromen-2-one (**(*Z*)-6a**)

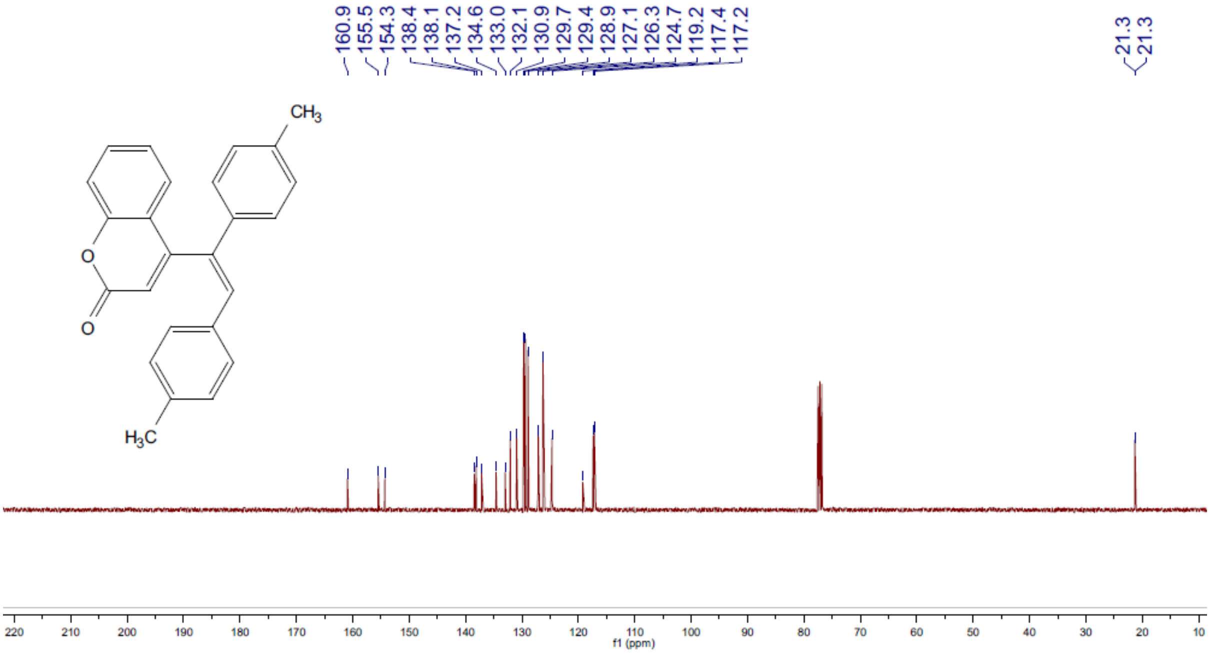

Supplementary Figure 125. ^{13}C NMR (100 MHz, $CDCl_3$) spectra of (*Z*)-4-(1,2-di-*p*-tolylvinyl)-2*H*-chromen-2-one (**(*Z*)-6a)**

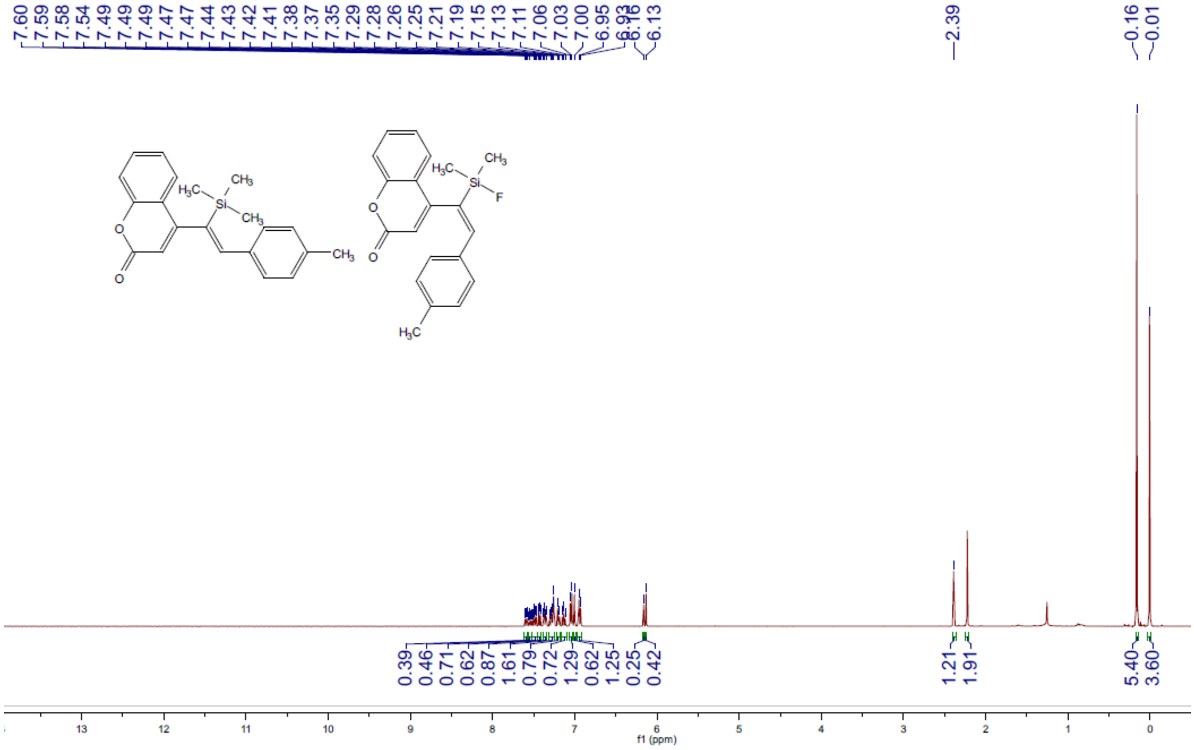

Supplementary Figure 126. ^{1}H NMR (400 MHz, $CDCl_3$) spectra of 4-(2-(*p*-tolyl)-1-(trimethylsilyl)vinyl)-2*H*-chromen-2-one (**6b**)

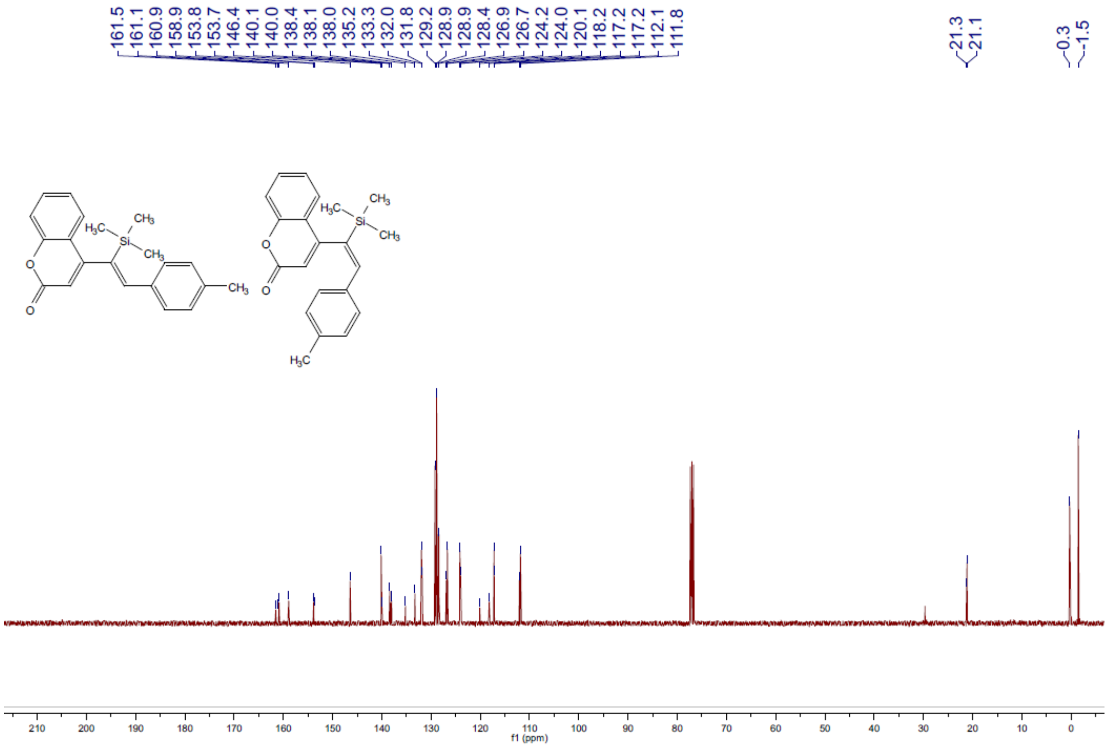

Supplementary Figure 127. ^{13}C NMR (100 MHz, $CDCl_3$) spectra of 4-(2-(*p*-tolyl)-1-(trimethylsilyl)vinyl)-2*H*-chromen-2-one (**6b**)

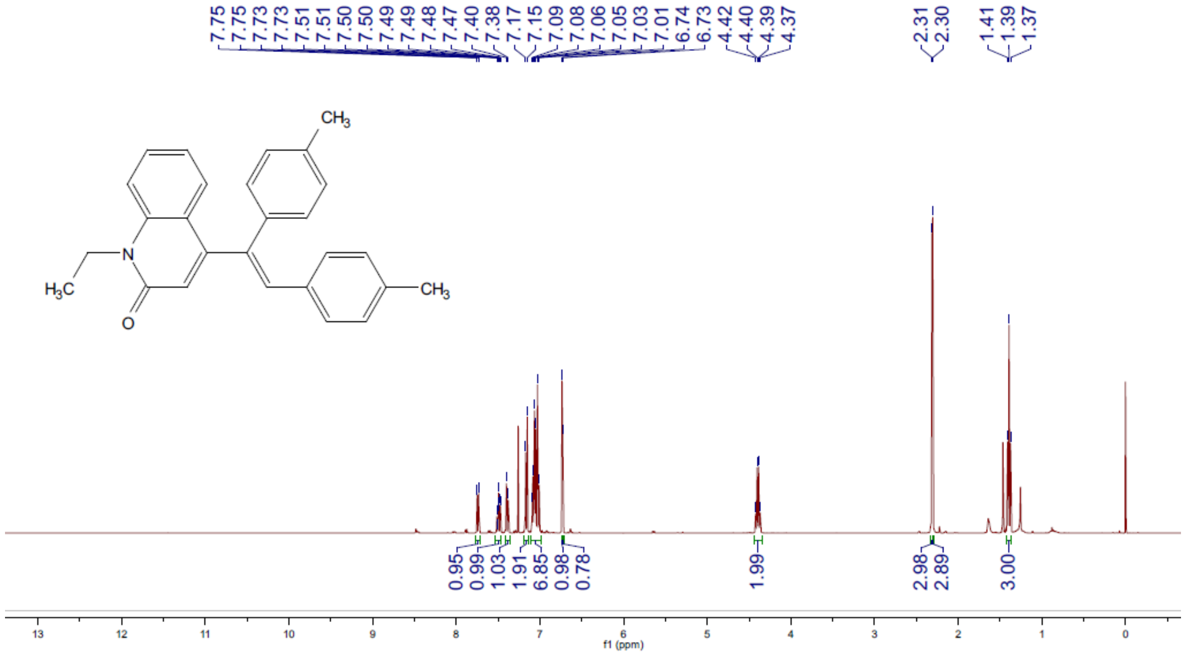

Supplementary Figure 128. ^{1}H NMR (400 MHz, $CDCl_3$) spectra of (*E*)-4-(1,2-di-*p*-tolylvinyl)-1-ethylquinolin-2(1*H*)-one (**(*E*)-6c**)

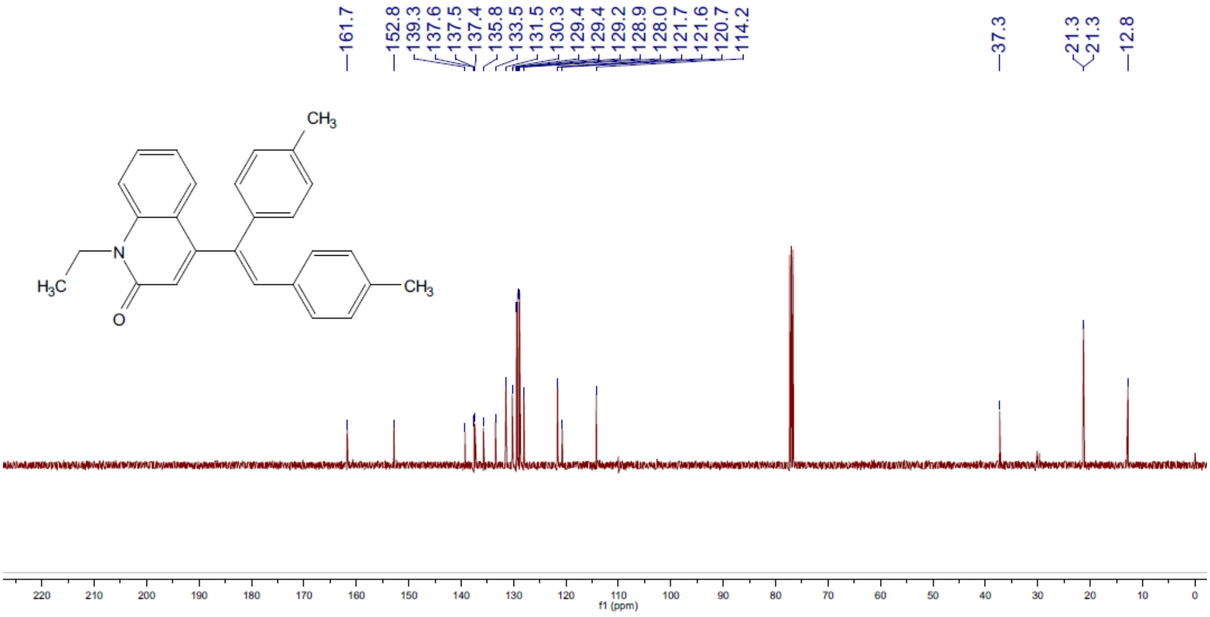

Supplementary Figure 129. ^{13}C NMR (100 MHz, $CDCl_3$) spectra of (*E*)-4-(1,2-di-*p*-tolylvinyl)-1-ethylquinolin-2(1*H*)-one (**(*E*)-6c**)

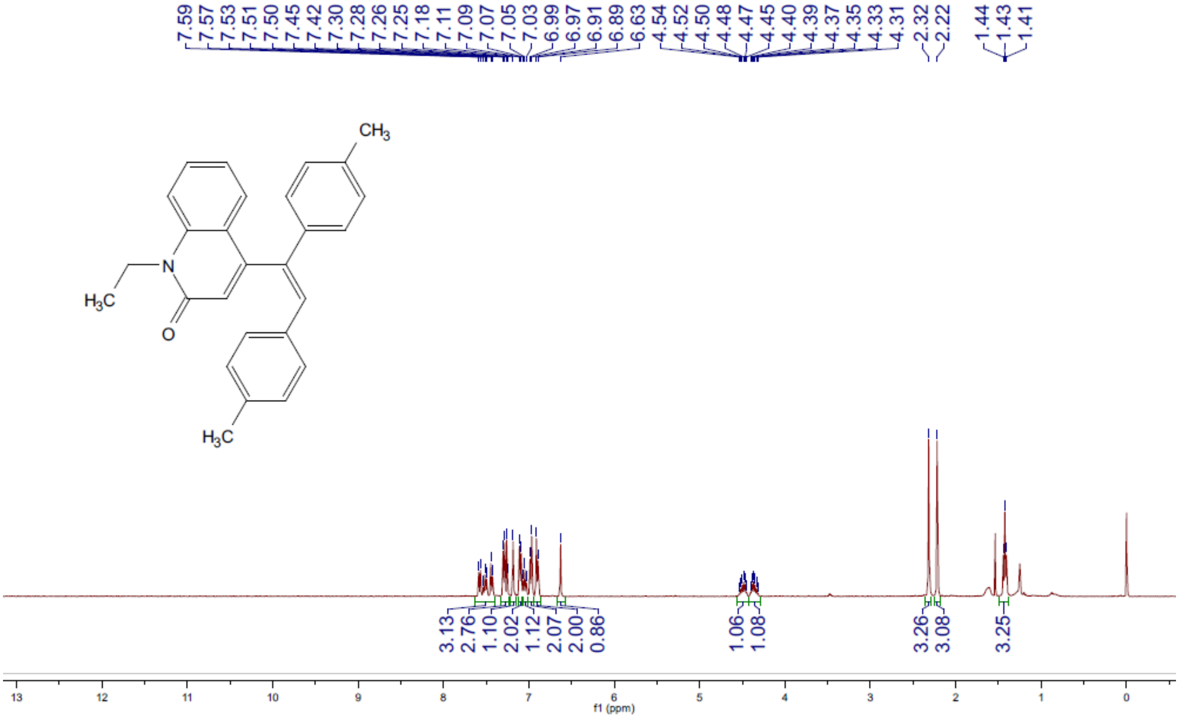

Supplementary Figure 130. ^{1}H NMR (400 MHz, $CDCl_3$) spectra of (*Z*)-4-(1,2-di-*p*-tolylvinyl)-1-ethylquinolin-2(1*H*)-one (**(*Z*)-6c**)

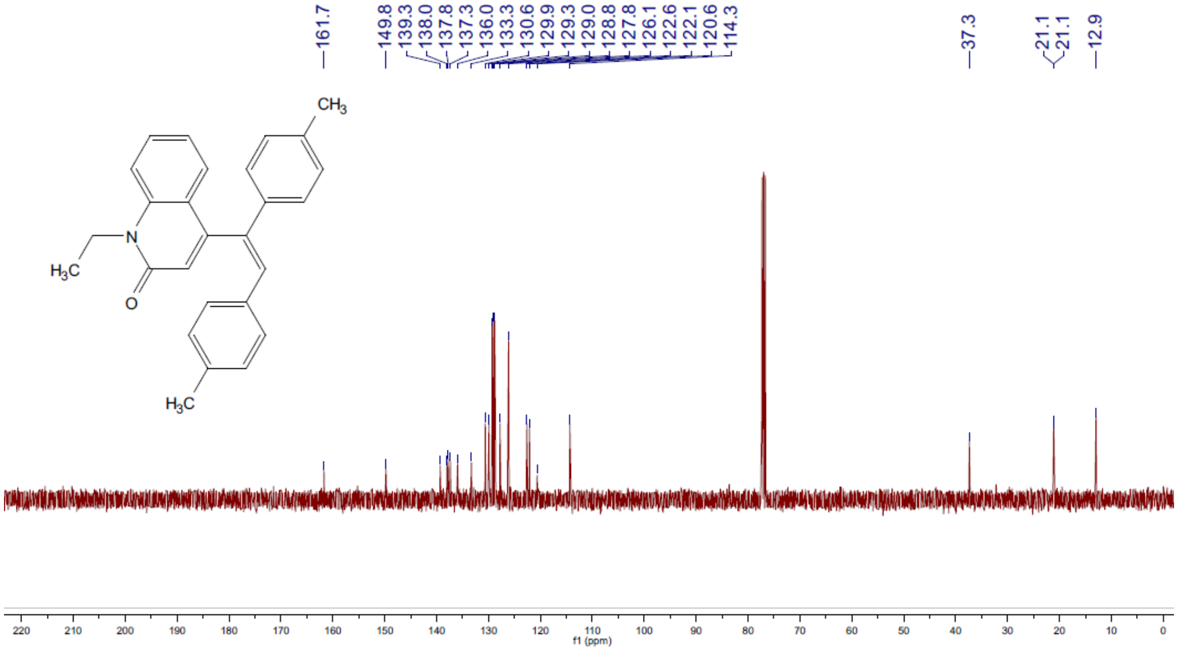

Supplementary Figure 131. ^{13}C NMR (100 MHz, $CDCl_3$) spectra of (*Z*)-4-(1,2-di-*p*-tolylvinyl)-1-ethylquinolin-2(1*H*)-one (**(*Z*)-6c**)

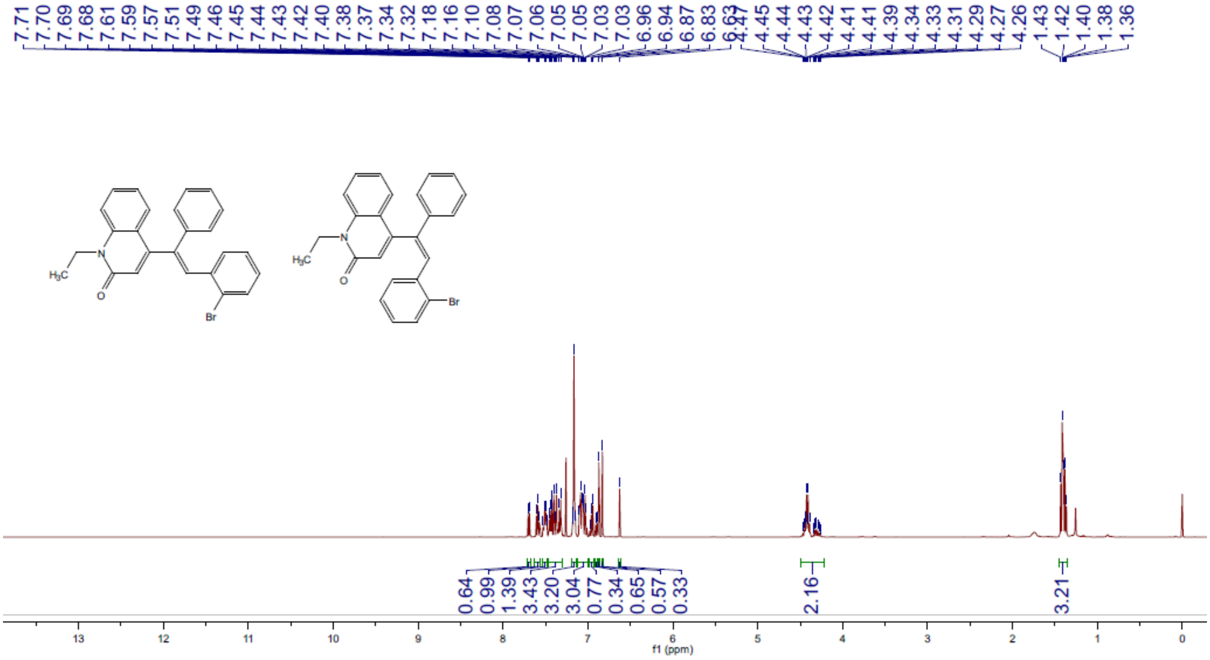

Supplementary Figure 132. ^{1}H NMR (400 MHz, $CDCl_3$) spectra of 4-(2-(2-bromophenyl)-1-phenylvinyl)-1-ethylquinolin-2(1*H*)-one (**6d**)

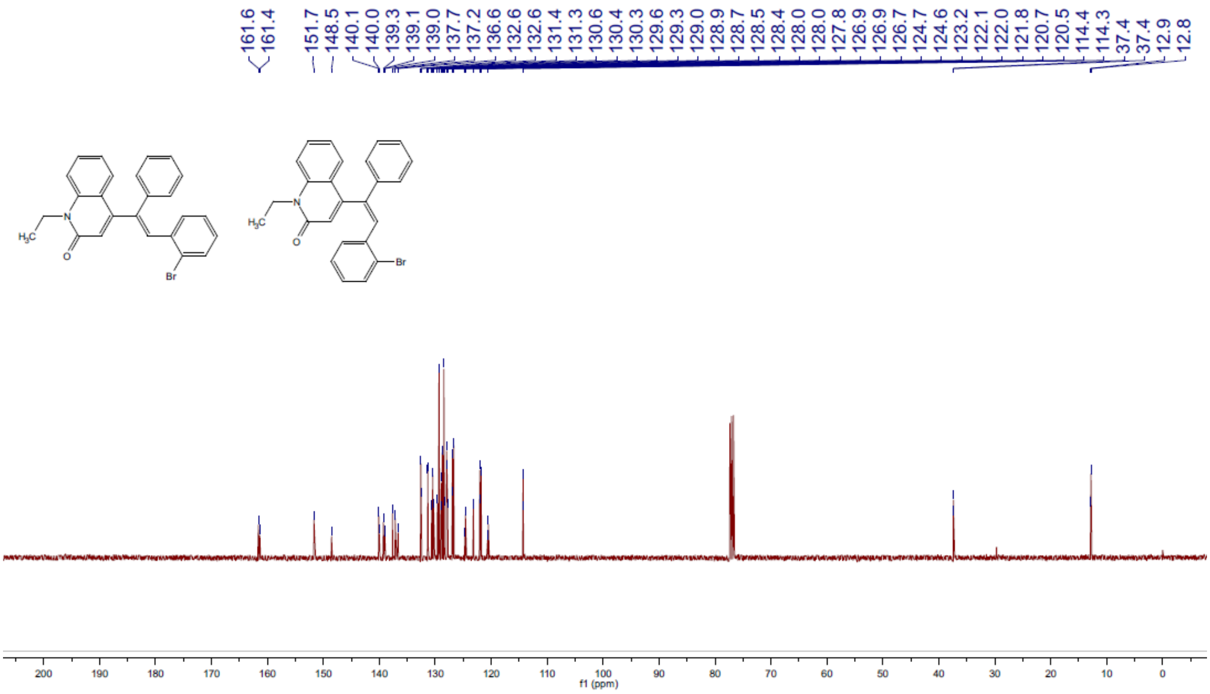

Supplementary Figure 133. ^{13}C NMR (100 MHz, $CDCl_3$) spectra of 4-(2-(2-bromophenyl)-1-phenylvinyl)-1-ethylquinolin-2(1*H*)-one (**6d**)

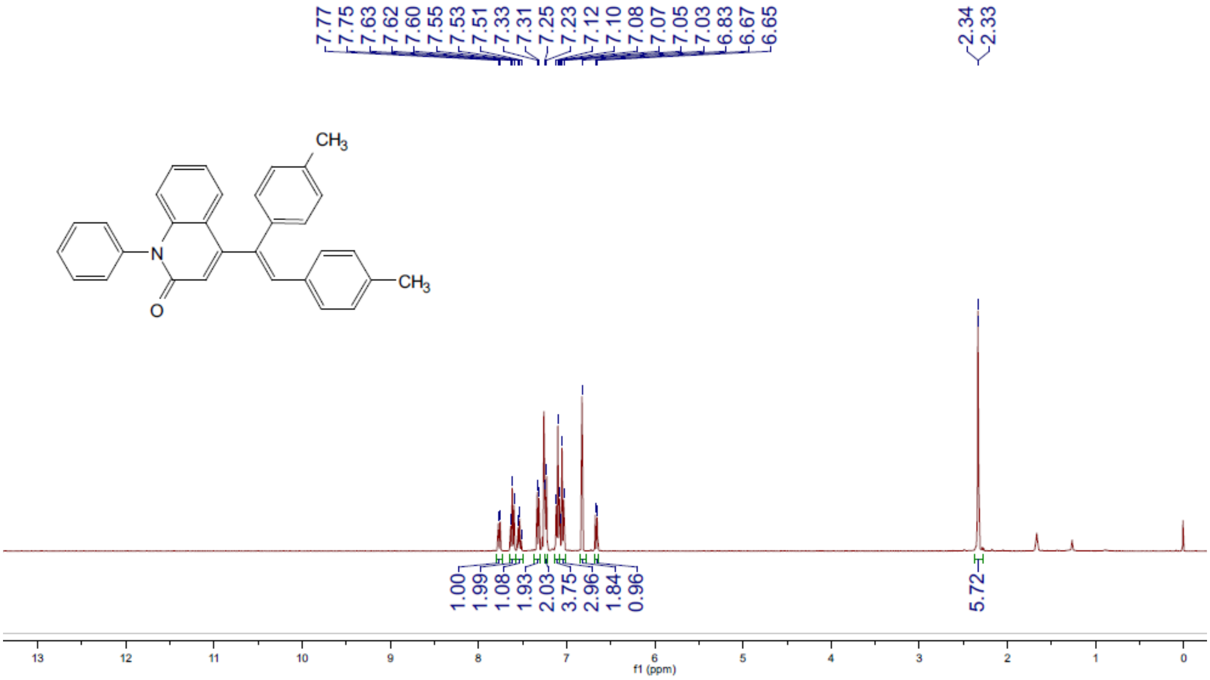

Supplementary Figure 134. ^{1}H NMR (400 MHz, $CDCl_3$) spectra of (*E*)-4-(1,2-di-*p*-tolylvinyl)-1-phenylquinolin-2(1H)-one (**(*E*)-6e**)

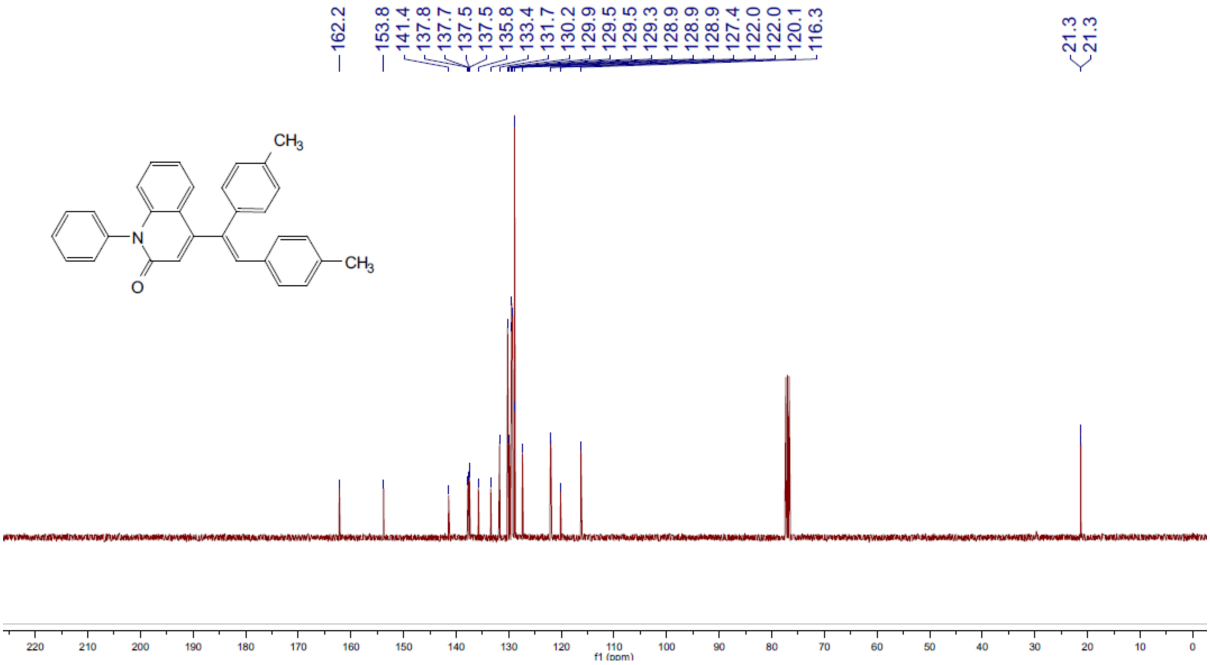

Supplementary Figure 135. ^{13}C NMR (100 MHz, $CDCl_3$) spectra of (*E*)-4-(1,2-di-*p*-tolylvinyl)-1-phenylquinolin-2(1H)-one (**(*E*)-6e)**

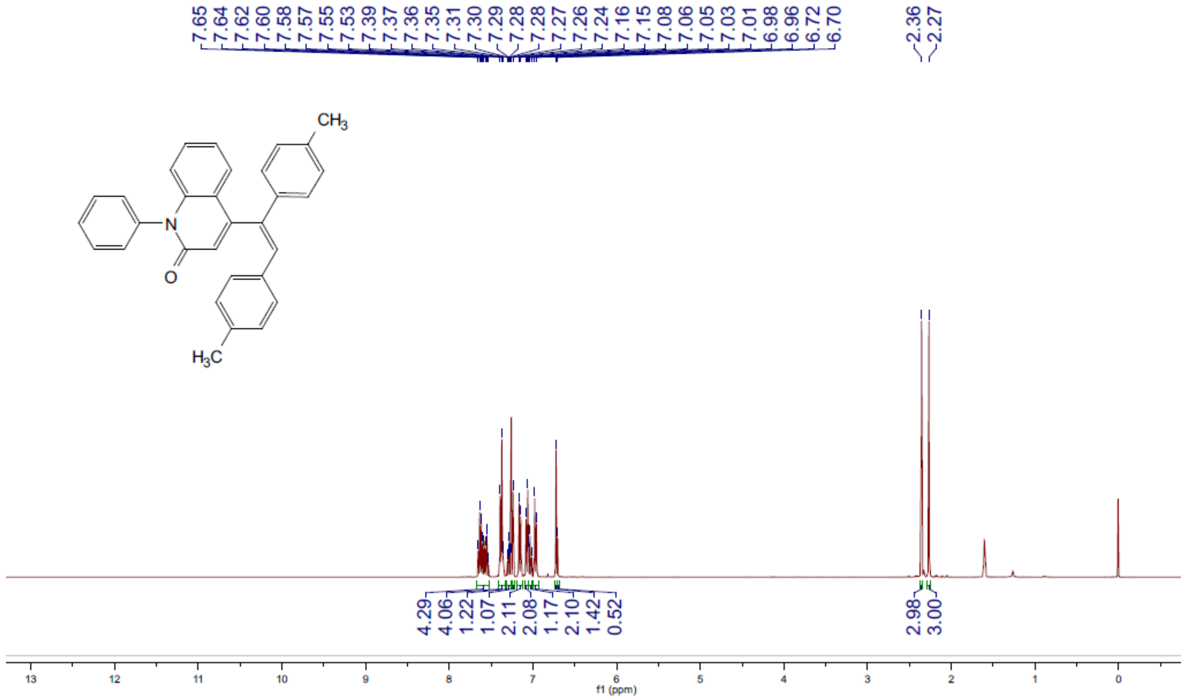

Supplementary Figure 136. ^{1}H NMR (400 MHz, $CDCl_3$) spectra of (*Z*)-4-(1,2-di-*p*-tolylvinyl)-1-phenylquinolin-2(1*H*)-one (**(*Z*)-6e**)

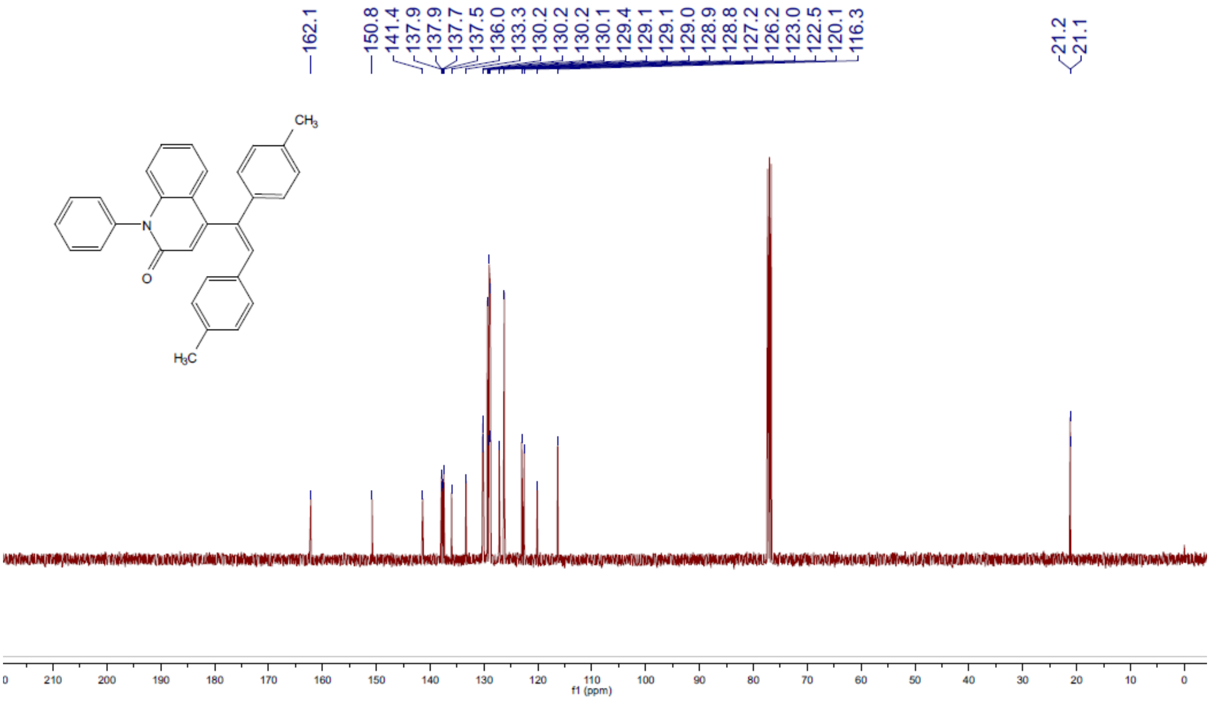

Supplementary Figure 137. ^{13}C NMR (100 MHz, $CDCl_3$) spectra of (*Z*)-4-(1,2-di-*p*-tolylvinyl)-1-phenylquinolin-2(1*H*)-one (**(*Z*)-6e**)

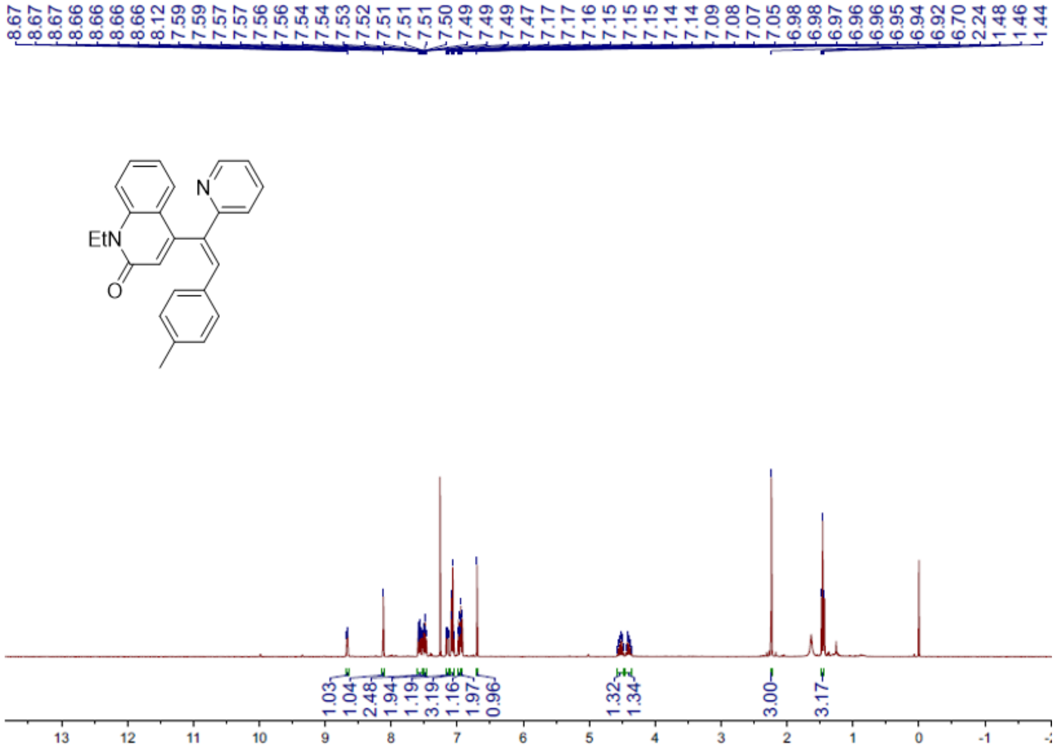

Supplementary Figure 138. ^{1}H NMR (400 MHz, $CDCl_3$) spectra of (*E*)-1-ethyl-4-(1-(pyridin-2-yl)-2-(*p*-tolyl)vinyl)quinolin-2(1*H*)-one (**(*E*)-6f)**

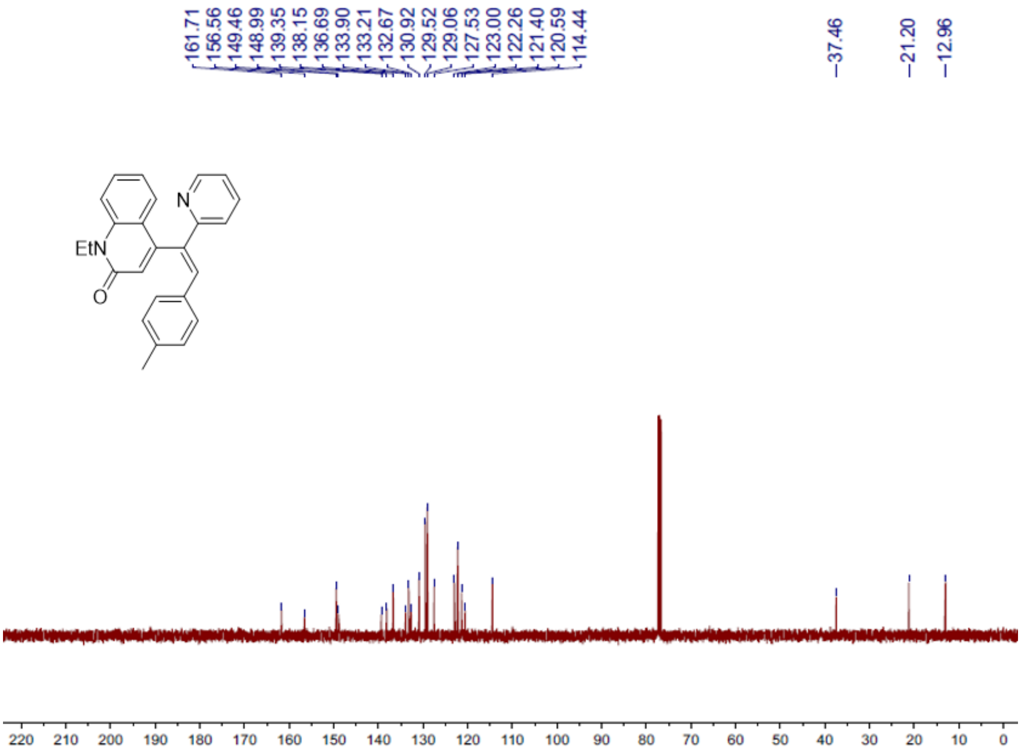

Supplementary Figure 139. ^{13}C NMR (100 MHz, $CDCl_3$) spectra of (*E*)-1-ethyl-4-(1-(pyridin-2-yl)-2-(*p*-tolyl)vinyl)quinolin-2(1H)-one (***(E)*-6f**)

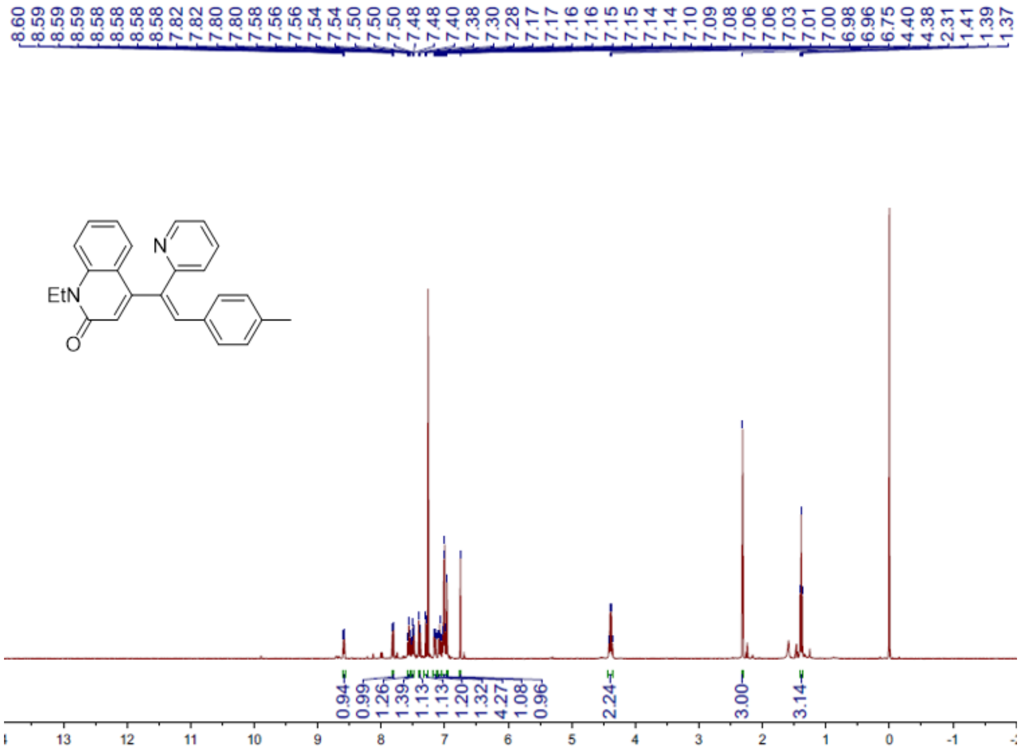

Supplementary Figure 140. ^{1}H NMR (400 MHz, $CDCl_3$) spectra of (*Z*)-1-ethyl-4-(1-(pyridin-2-yl)-2-(*p*-tolyl)vinyl)quinolin-2(1*H*)-one (***(Z)*-6f**)

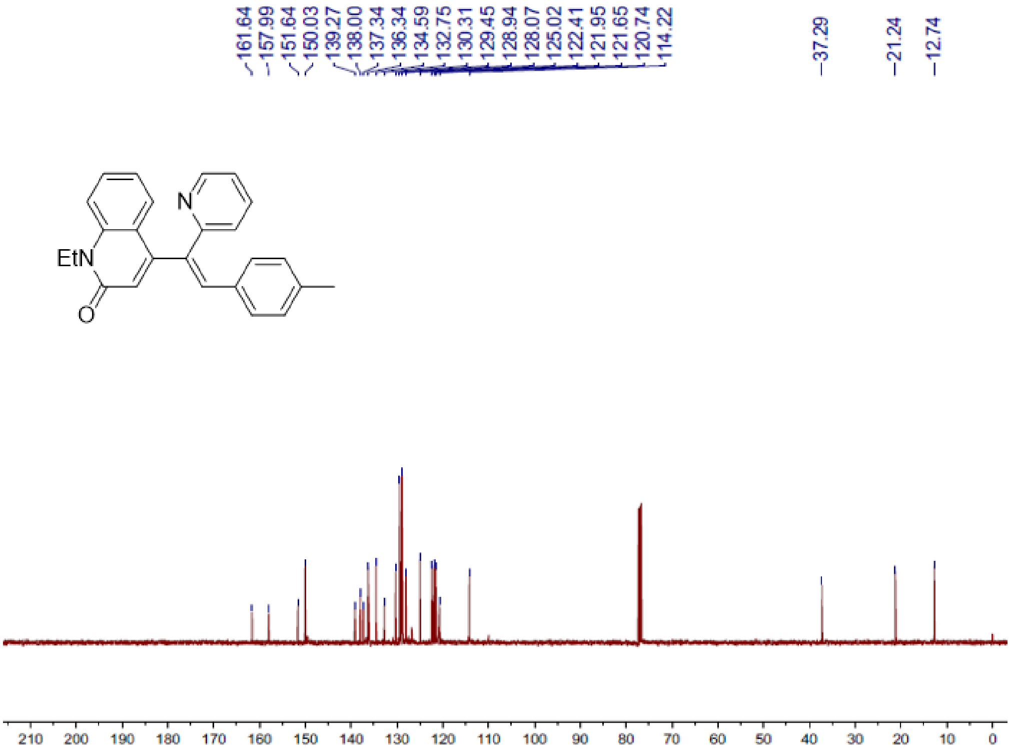

Supplementary Figure 141. ^{13}C NMR (100 MHz, $CDCl_3$) spectra of (*Z*)-1-ethyl-4-(1-(pyridin-2-yl)-2-(*p*-tolyl)vinyl)quinolin-2(1*H*)-one (**(*Z*)-6f)**

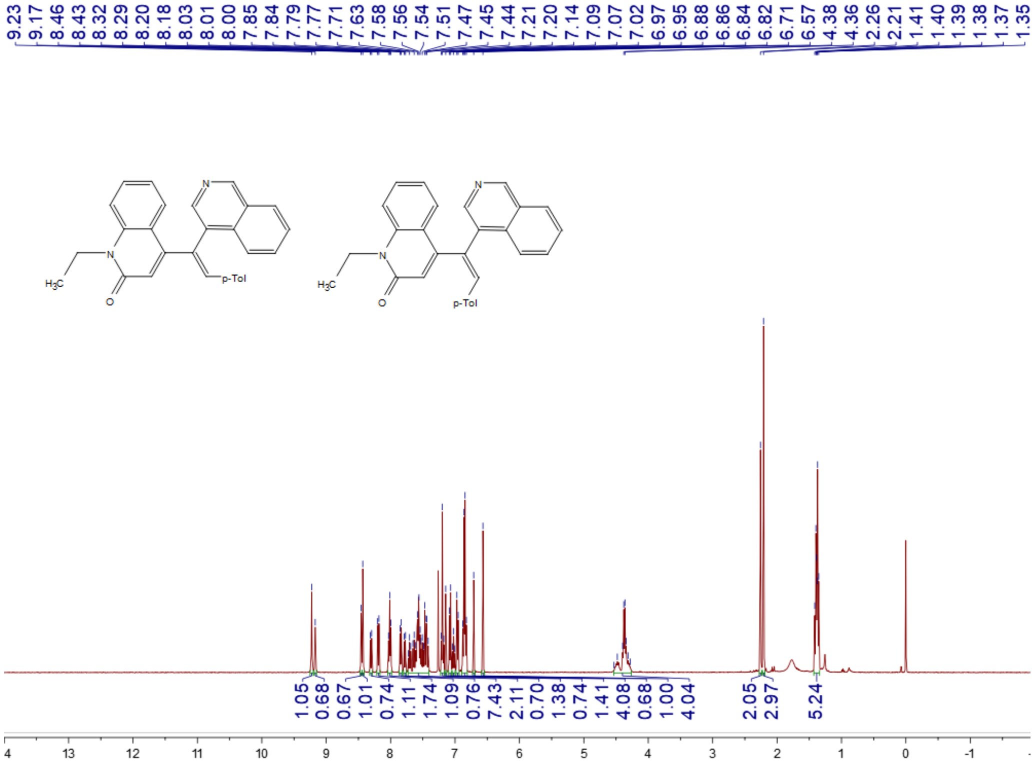

Supplementary Figure 142. ^{1}H NMR (400 MHz, $CDCl_3$) spectra of 1-ethyl-4-(1-(isoquinolin-4-yl)-2-(*p*-tolyl)vinyl)quinolin-2(1*H*)-one (**6g**)

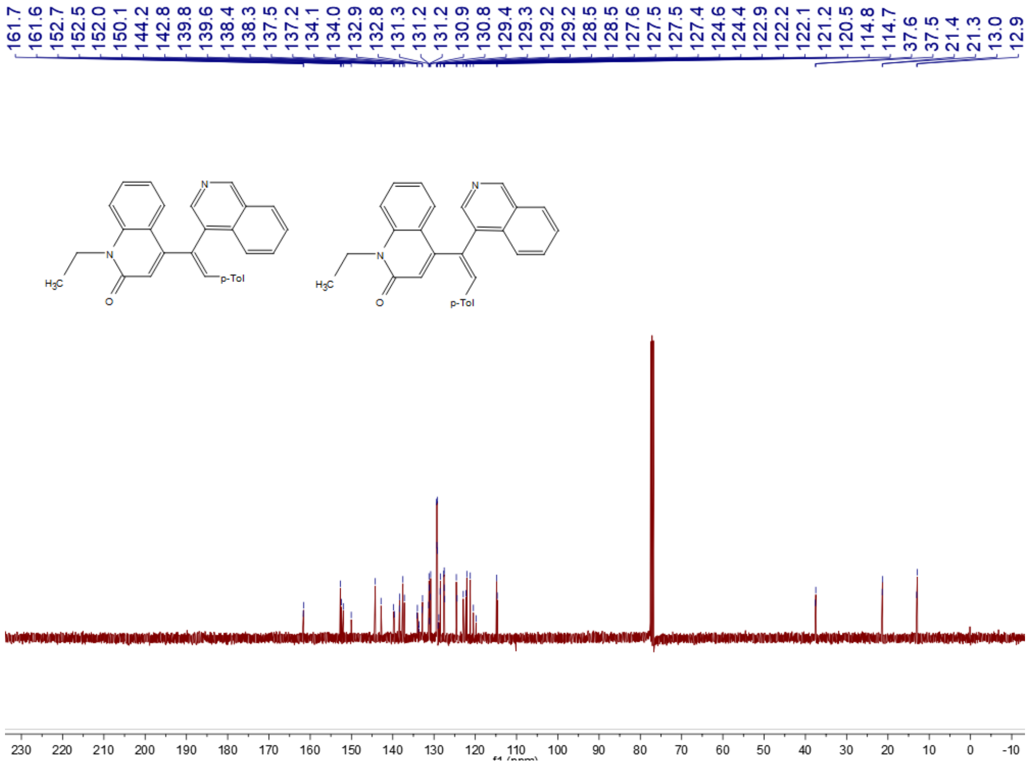

Supplementary Figure 143. ^{13}C NMR (100 MHz, $CDCl_3$) spectra of 1-ethyl-4-(1-(isoquinolin-4-yl)-2-(*p*-tolyl)vinyl)quinolin-2(1*H*)-one (**6g**)

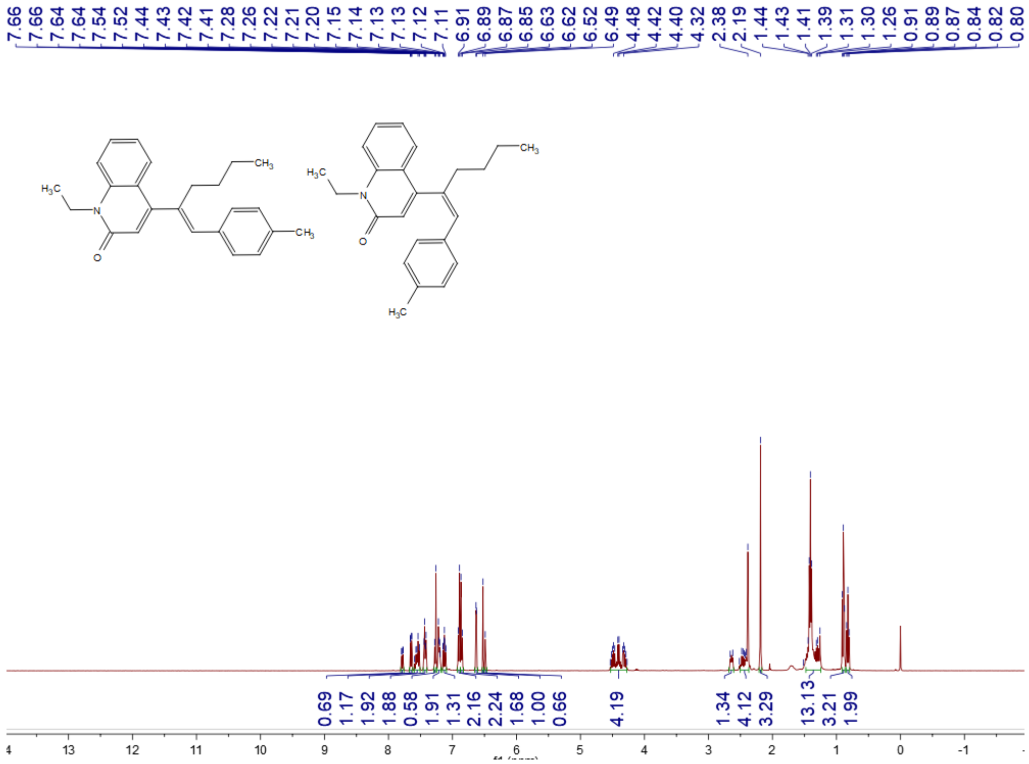

Supplementary Figure 144. ^{1}H NMR (400 MHz, $CDCl_3$) spectra of 1-ethyl-4-(1-(*p*-tolyl)hex-1-en-2-yl)quinolin-2(1*H*)-one (**6h**)

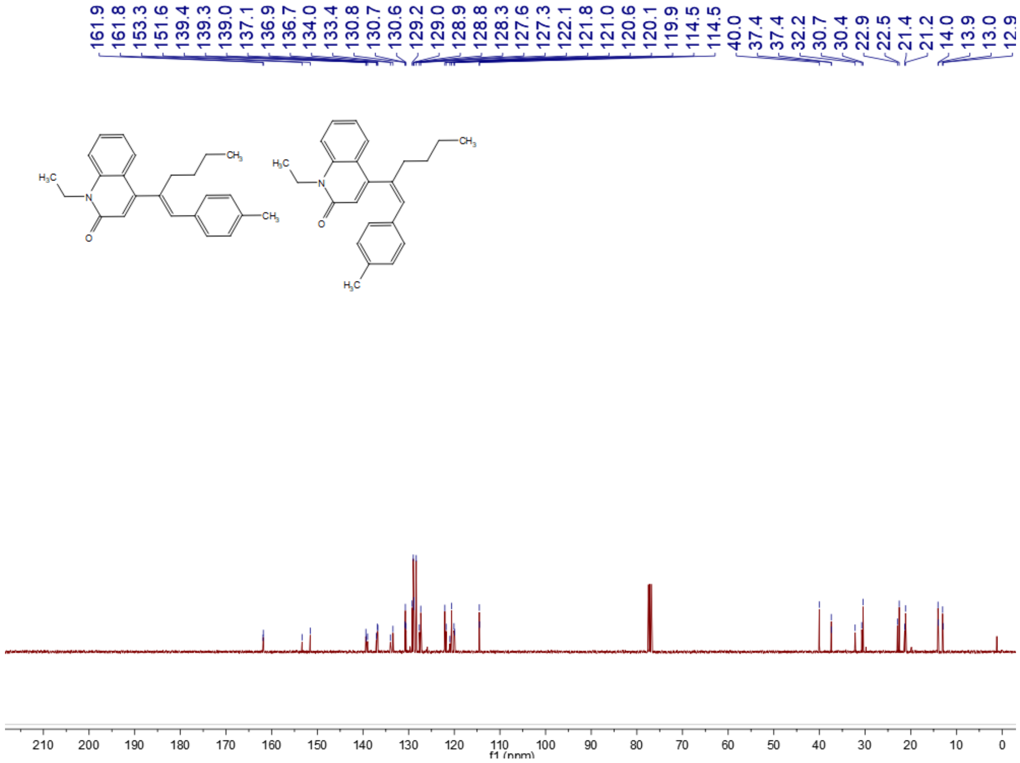

Supplementary Figure 145. ^{13}C NMR (100 MHz, $CDCl_3$) spectra of 1-ethyl-4-(1-(*p*-tolyl)hex-1-en-2-yl)quinolin-2(1*H*)-one (**6h**)

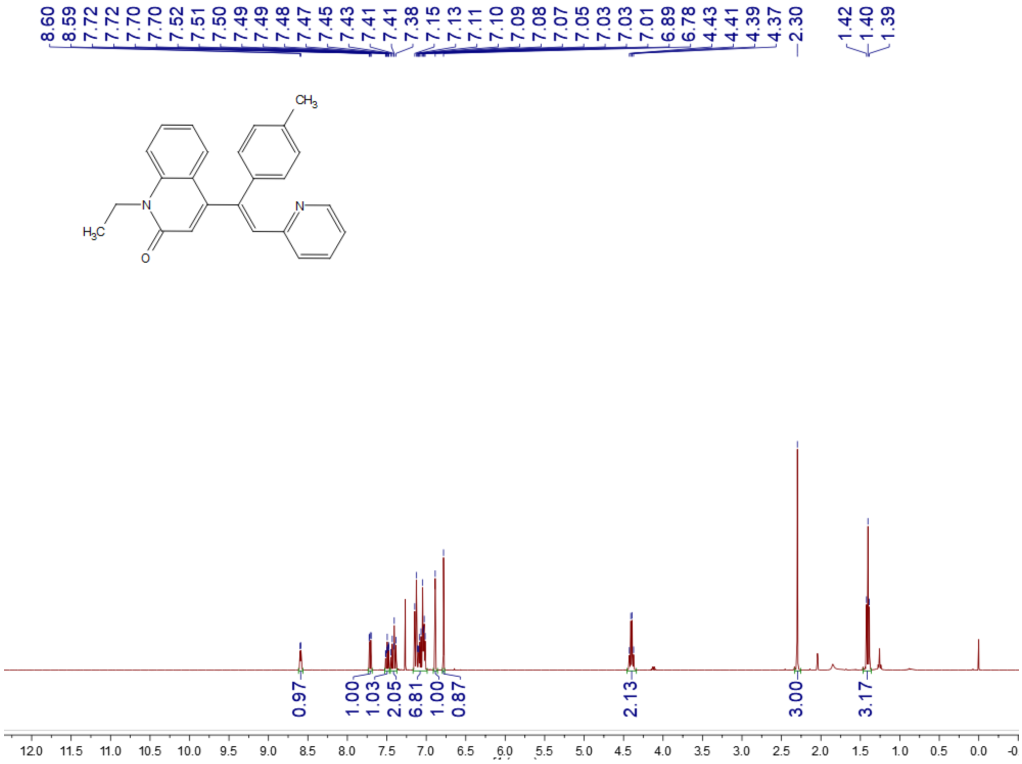

Supplementary Figure 146. ^{1}H NMR (400 MHz, $CDCl_3$) spectra of (*E*)-1-ethyl-4-(2-(pyridin-2-yl)-1-(*p*-tolyl)vinyl)quinolin-2(1H)-one (**(*E*)-6i**)

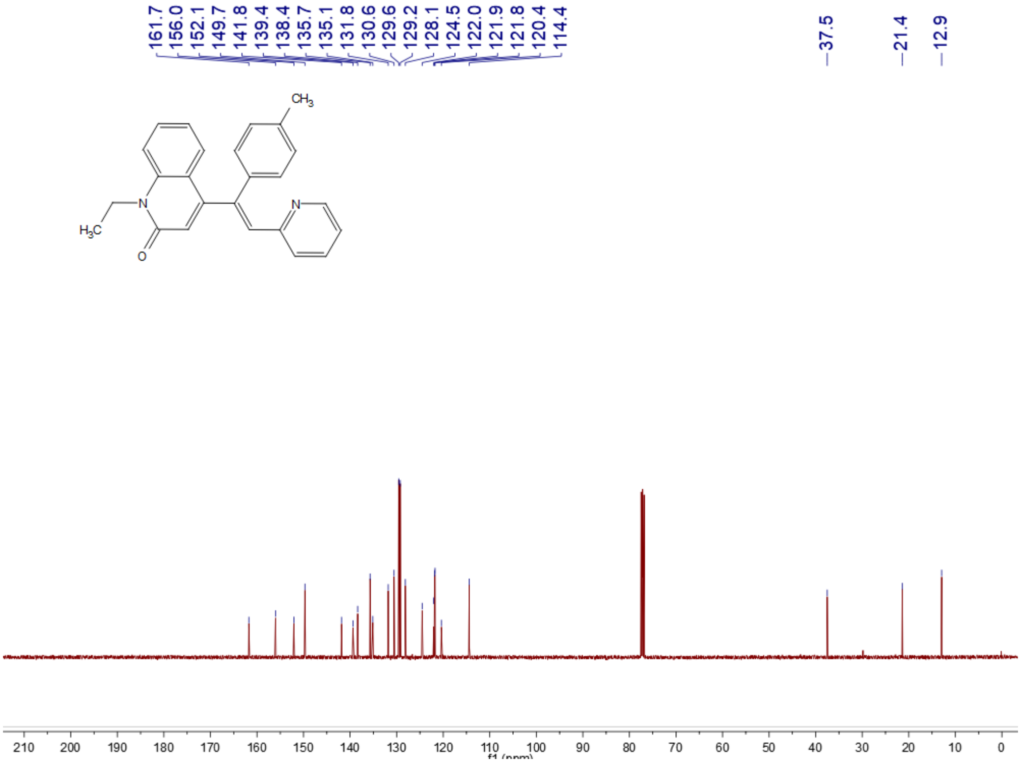

Supplementary Figure 147. ^{13}C NMR (100 MHz, $CDCl_3$) spectra of (*E*)-1-ethyl-4-(2-(pyridin-2-yl)-1-(*p*-tolyl)vinyl)quinolin-2(1H)-one (***(E)*-6i**)

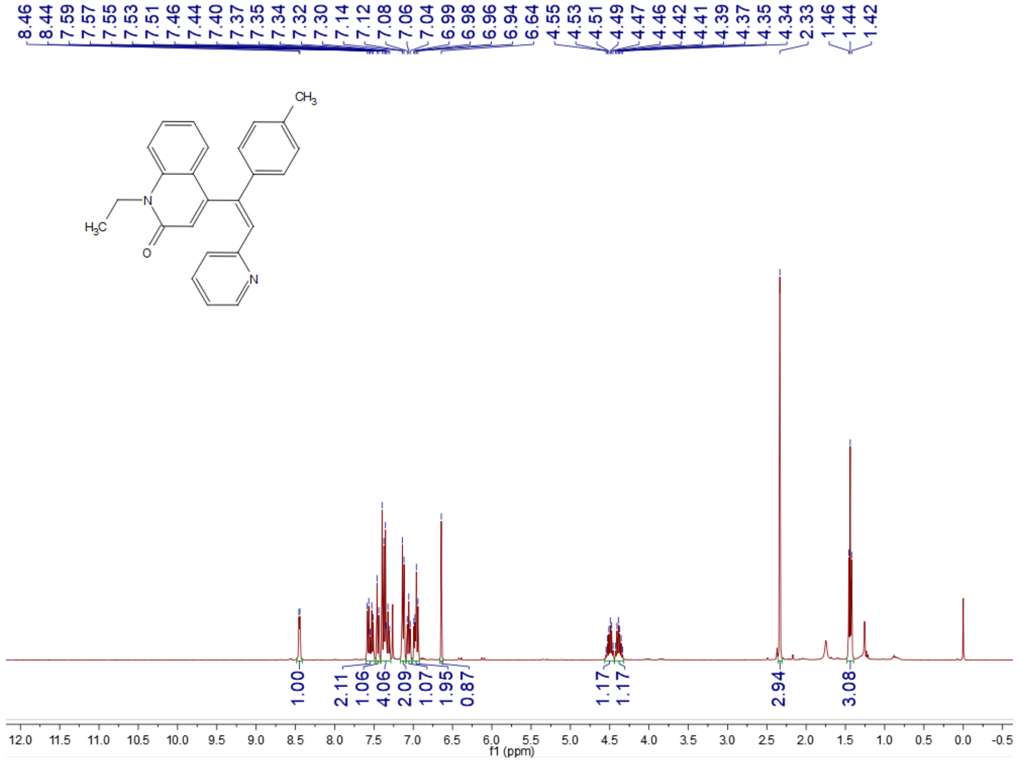

Supplementary Figure 148. ^1H NMR (400 MHz, $CDCl_3$) spectra of (*Z*)-1-ethyl-4-(2-(pyridin-2-yl)-1-(*p*-tolyl)vinyl)quinolin-2(1*H*)-one (***(Z)*-6i**)

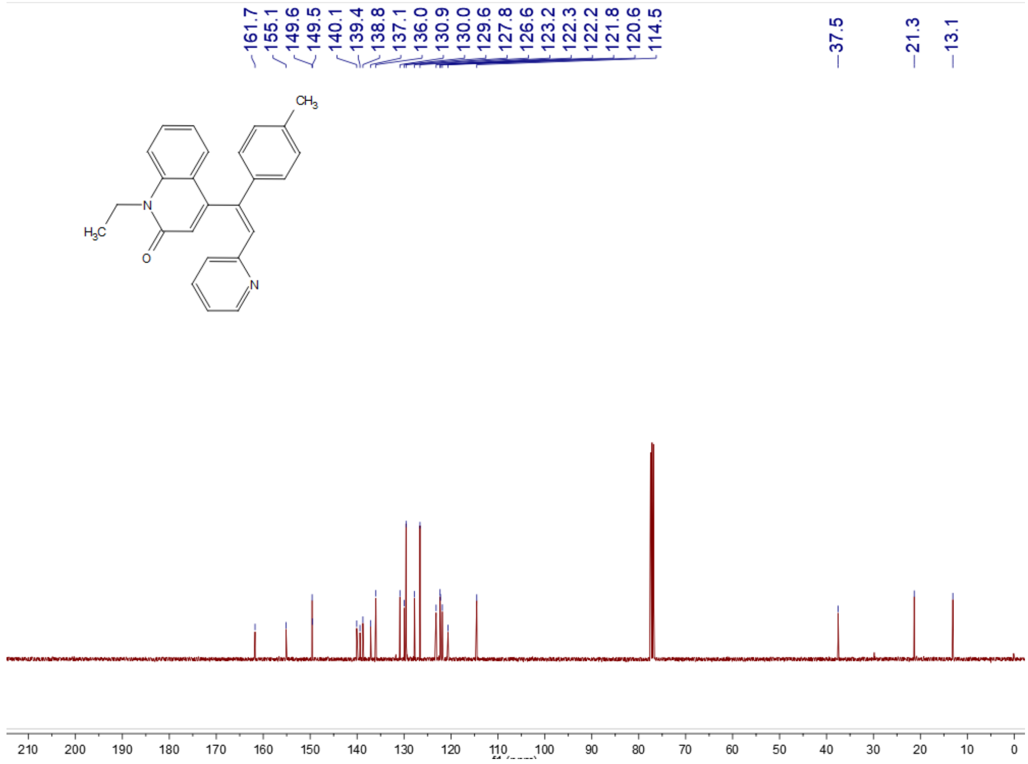

Supplementary Figure 149. ^{13}C NMR (100 MHz, $CDCl_3$) spectra of (*Z*)-1-ethyl-4-(2-(pyridin-2-yl)-1-(*p*-tolyl)vinyl)quinolin-2(1*H*)-one (**(*E*)-6i**)

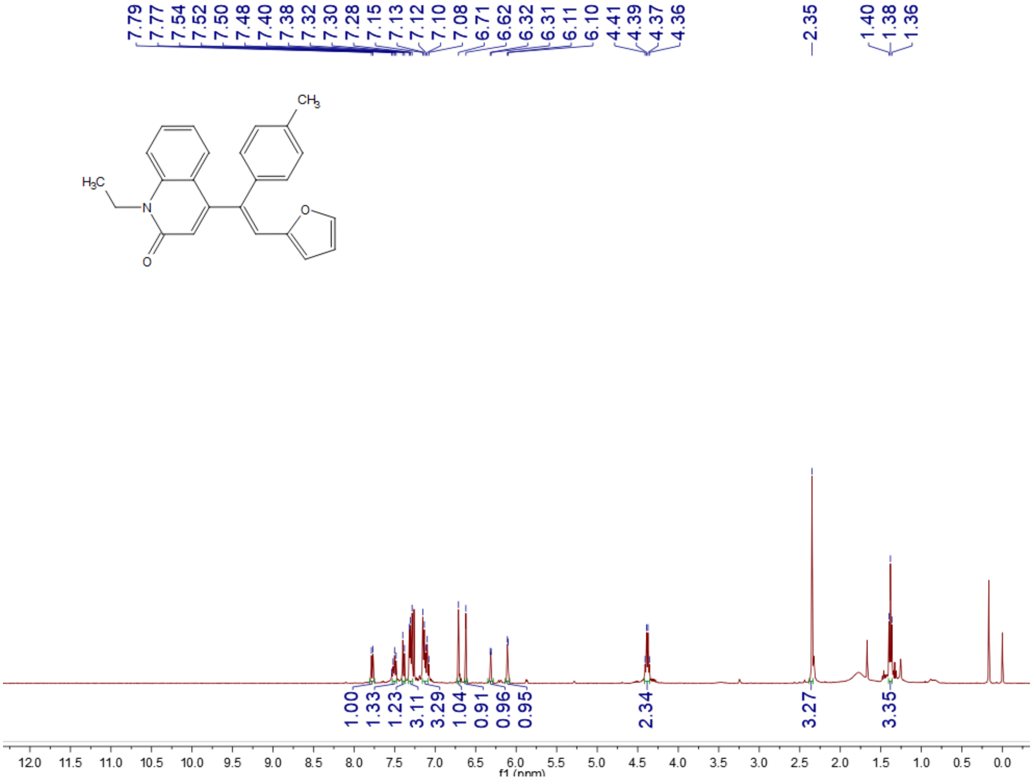

Supplementary Figure 150. ^{1}H NMR (400 MHz, $CDCl_3$) spectra of (*E*)-1-ethyl-4-(2-(furan-2-yl)-1-(*p*-tolyl)vinyl)quinolin-2(1H)-one (**(*E*)-6j**)

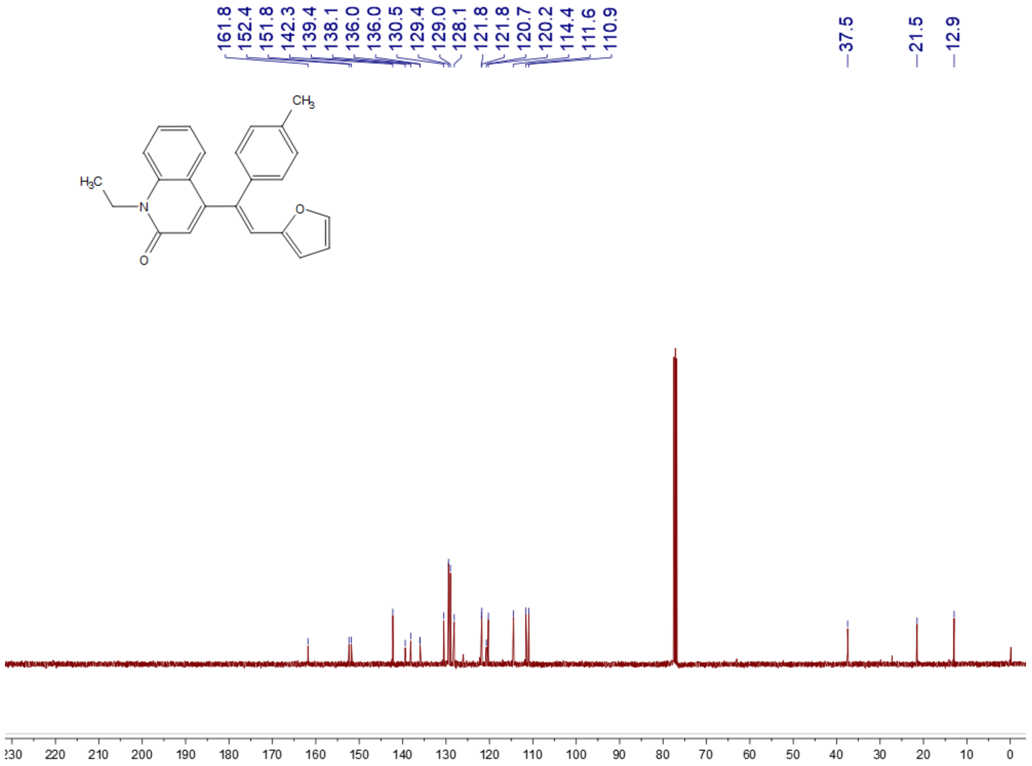

Supplementary Figure 151. ^{13}C NMR (100 MHz, $CDCl_3$) spectra of (*E*)-1-ethyl-4-(2-(furan-2-yl)-1-(*p*-tolyl)vinyl)quinolin-2(1H)-one (**(*E*)-6j**)

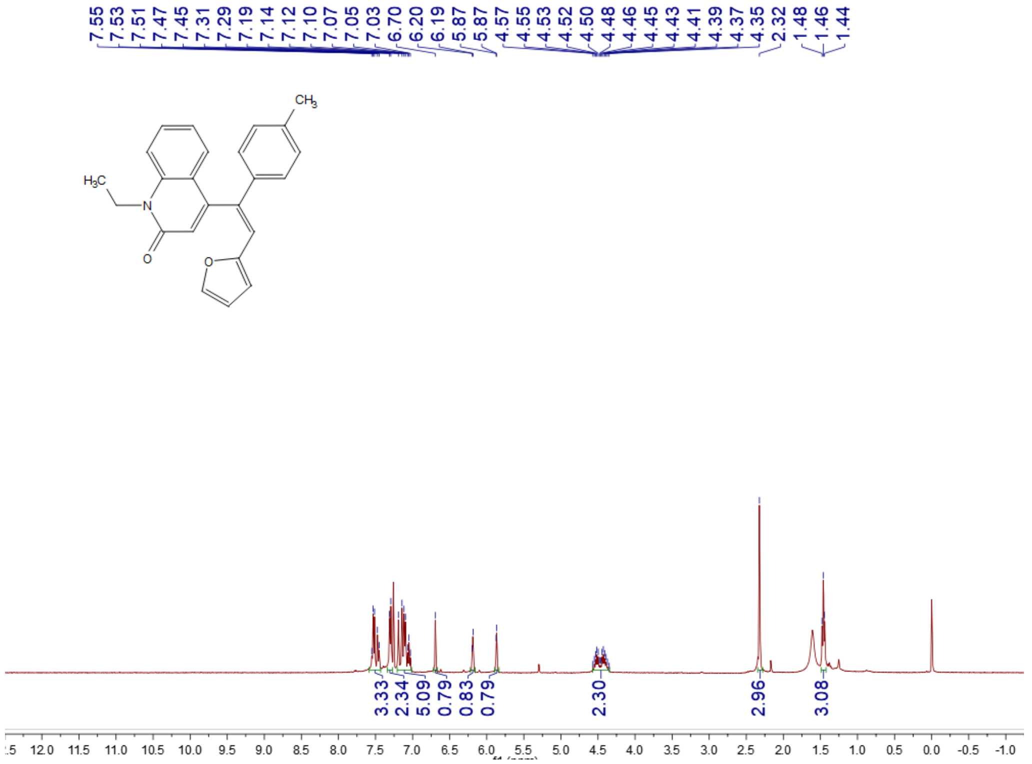

Supplementary Figure 152. ^{1}H NMR (400 MHz, $CDCl_3$) spectra of (*Z*)-1-ethyl-4-(2-(furan-2-yl)-1-(*p*-tolyl)vinyl)quinolin-2(1*H*)-one (**(*Z*)-6j**)

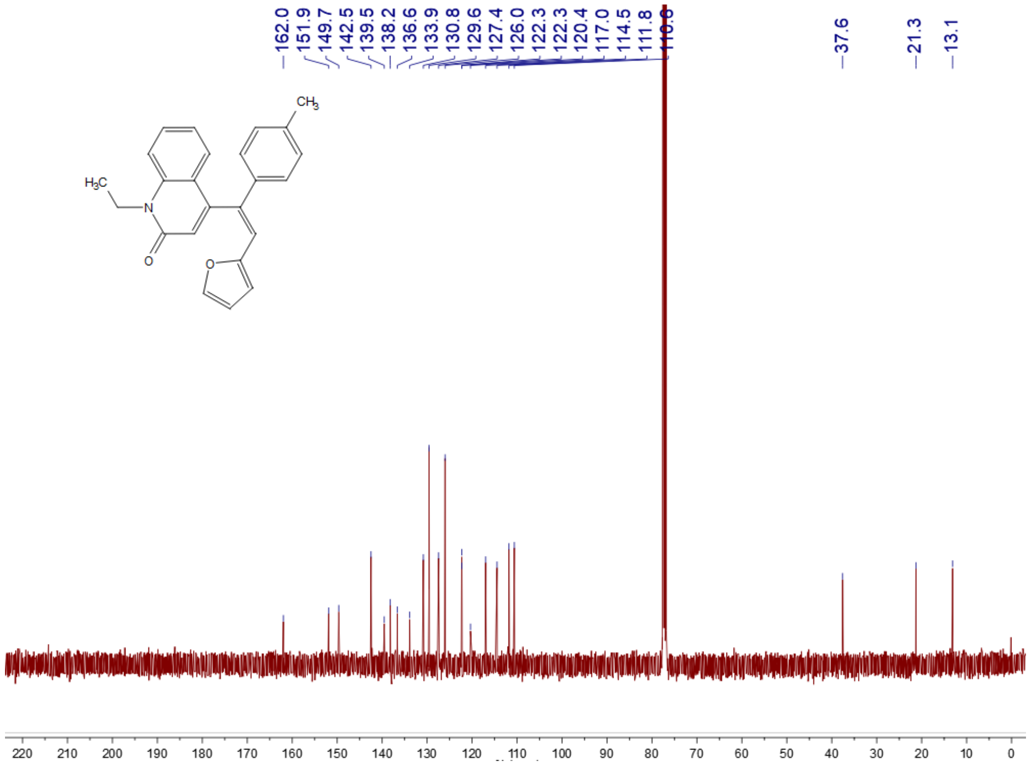

Supplementary Figure 153. ^{13}C NMR (100 MHz, $CDCl_3$) spectra of (*Z*)-1-ethyl-4-(2-(furan-2-yl)-1-(*p*-tolyl)vinyl)quinolin-2(1*H*)-one (**(*Z*)-6j**)

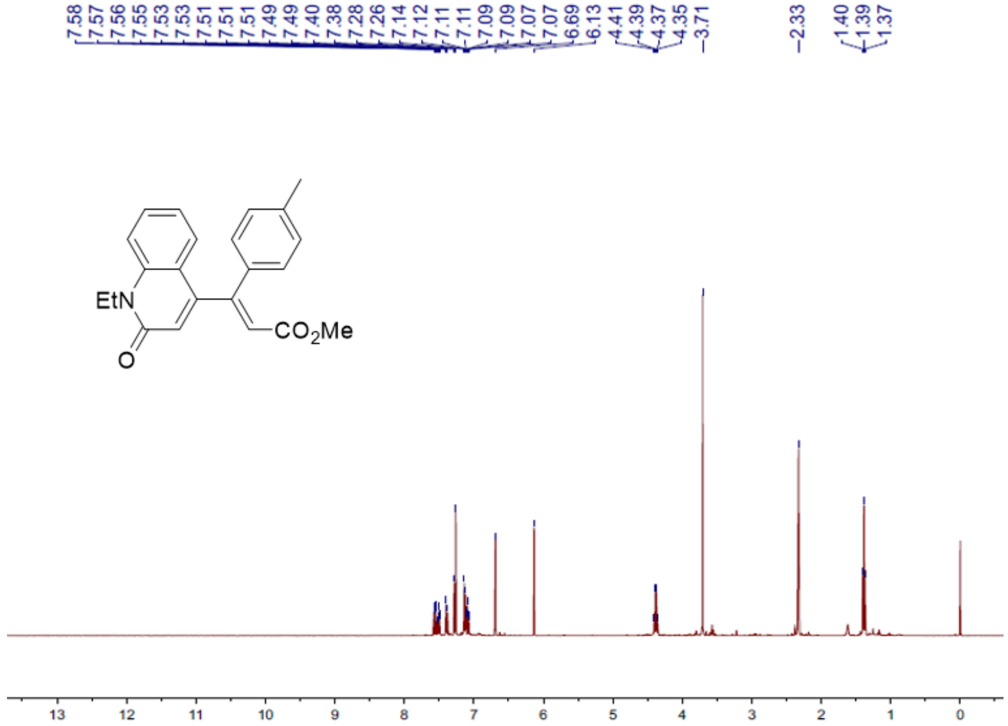

Supplementary Figure 154. ^{1}H NMR (400 MHz, $CDCl_3$) spectra of methyl (*E*)-3-(1-ethyl-2-oxo-1,2-dihydroquinolin-4-yl)-3-(*p*-tolyl)acrylate (**(*E*)-6k**)

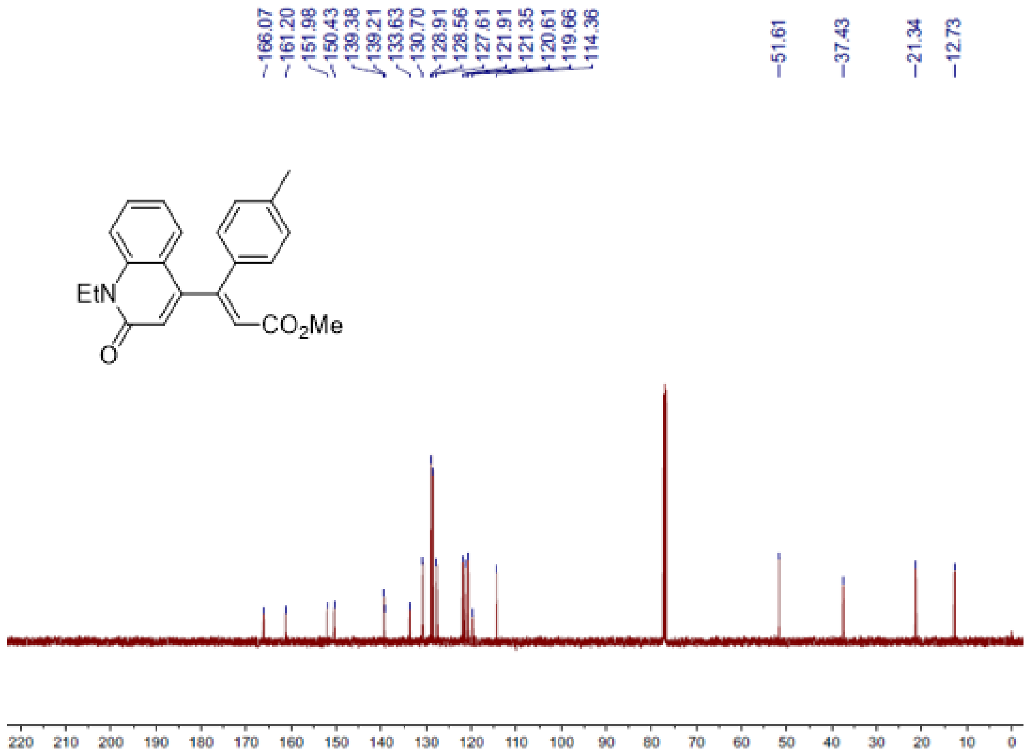

Supplementary Figure 155. ^{13}C NMR (100 MHz, $CDCl_3$) spectra of methyl (*E*)-3-(1-ethyl-2-oxo-1,2-dihydroquinolin-4-yl)-3-(*p*-tolyl)acrylate (**(*E*)-6k**)

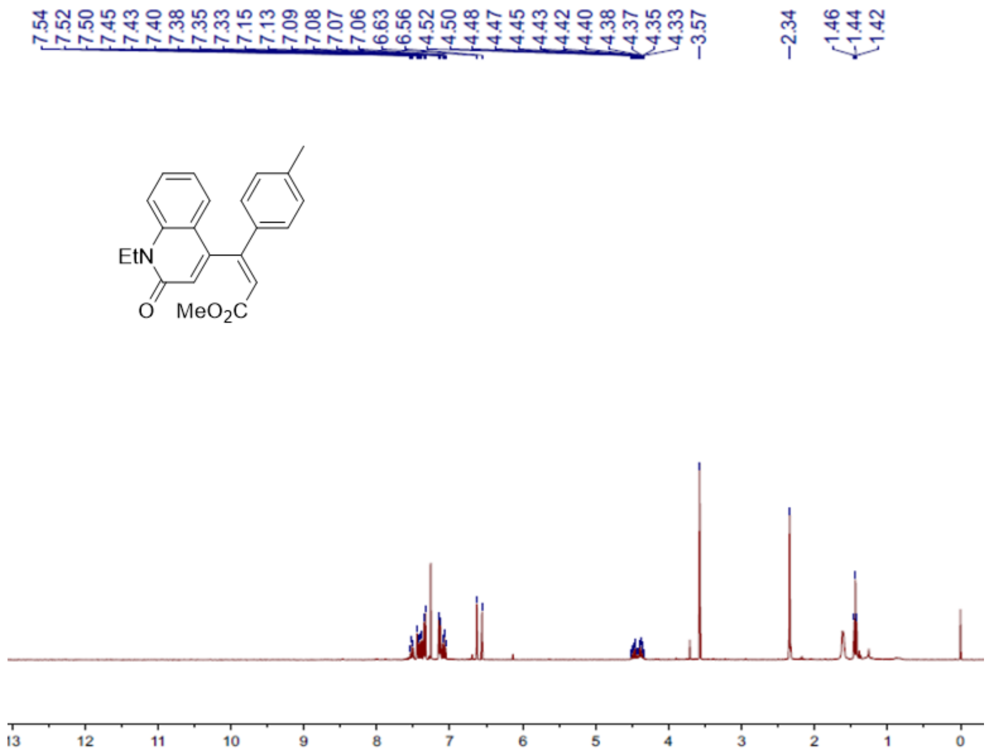

Supplementary Figure 156. ^{1}H NMR (400 MHz, $CDCl_3$) spectra of methyl (*Z*)-3-(1-ethyl-2-oxo-1,2-dihydroquinolin-4-yl)-3-(*p*-tolyl)acrylate (**(*Z*)-6k**)

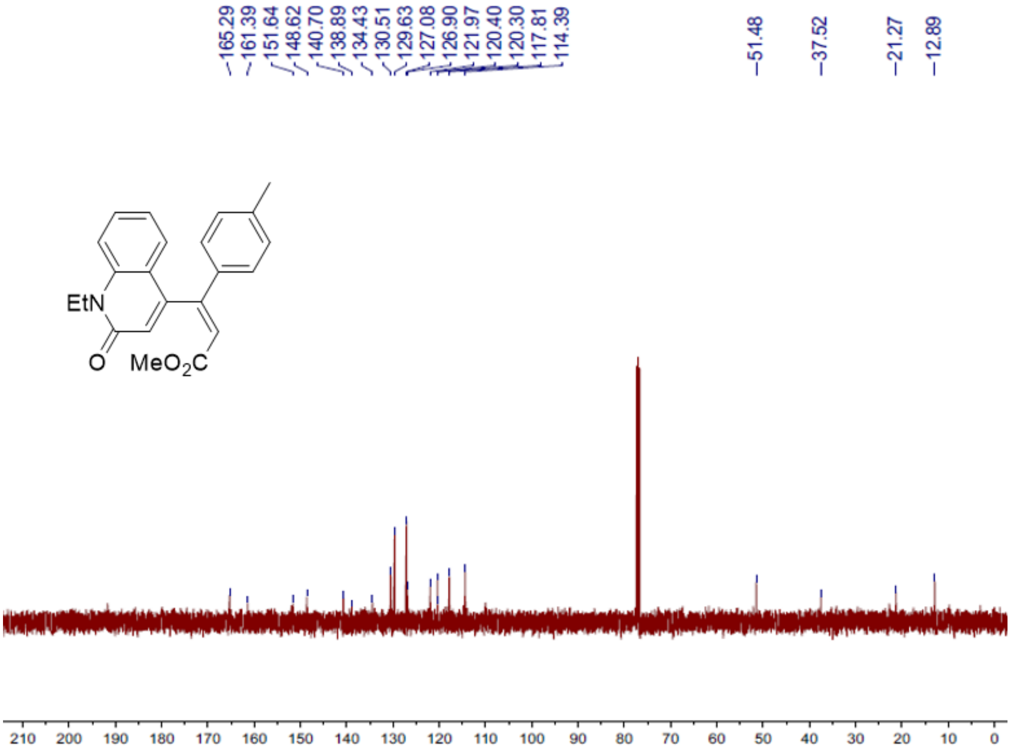

Supplementary Figure 157. ^{13}C NMR (100 MHz, $CDCl_3$) spectra of methyl (*Z*)-3-(1-ethyl-2-oxo-1,2-dihydroquinolin-4-yl)-3-(*p*-tolyl)acrylate (**(*Z*)-6k**)

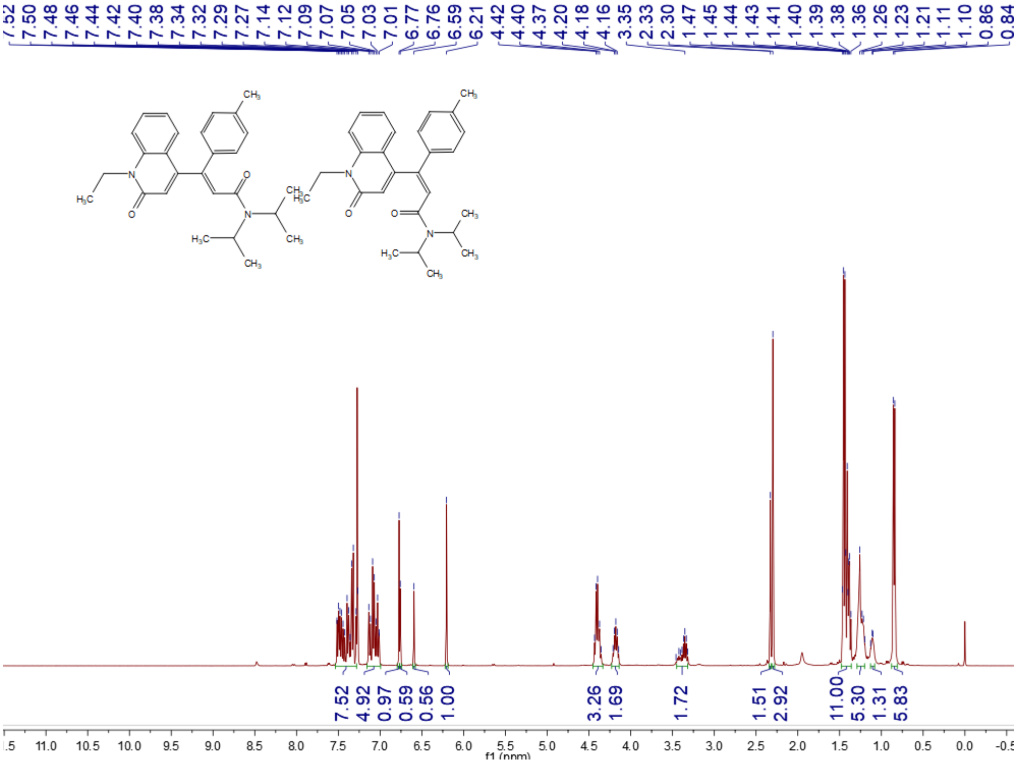

Supplementary Figure 158. ^{1}H NMR (400 MHz, $CDCl_3$) spectra of 3-(1-ethyl-2-oxo-1,2-dihydroquinolin-4-yl)-N,*N*-diisopropyl-3-(*p*-tolyl)acrylamide (**6i**)

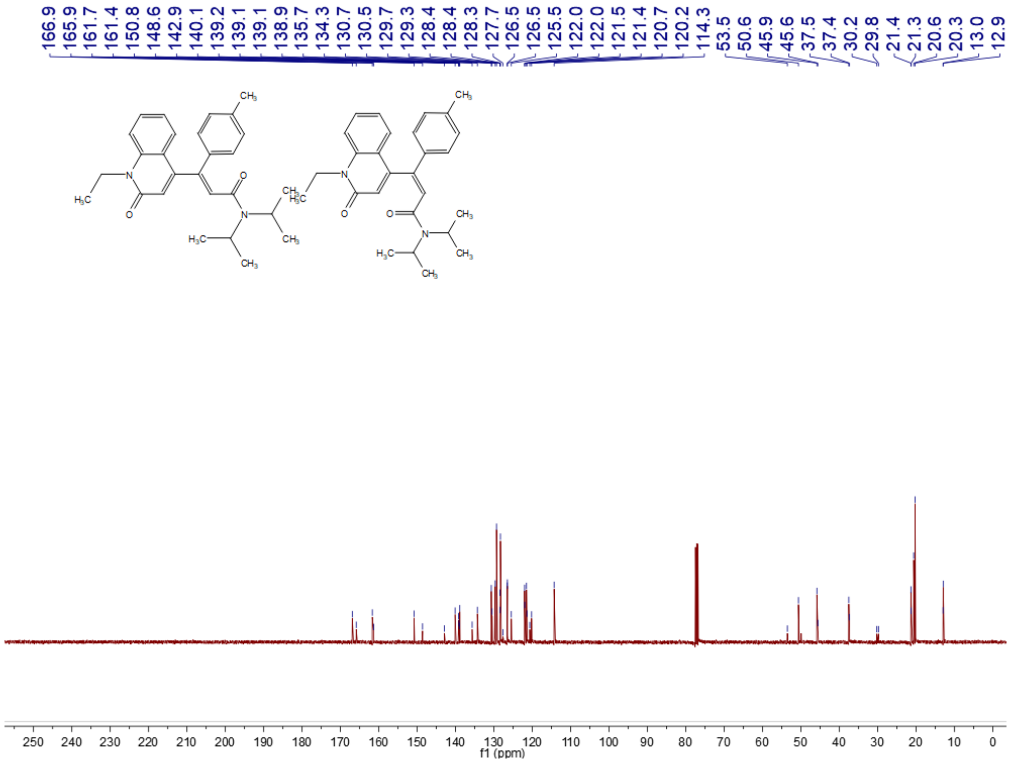

Supplementary Figure 159. ^{13}C NMR (100 MHz, $CDCl_3$) spectra of 3-(1-ethyl-2-oxo-1,2-dihydroquinolin-4-yl)-N,*N*-diisopropyl-3-(*p*-tolyl)acrylamide (**6i**)

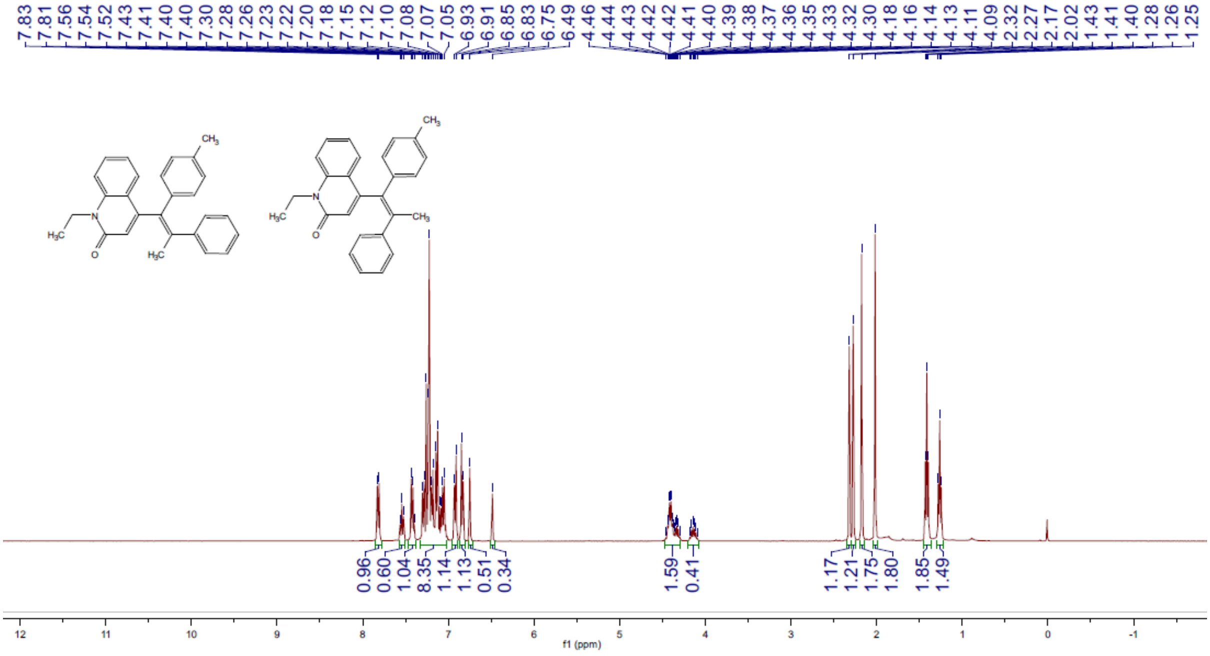

Supplementary Figure 160. ^{1}H NMR (400 MHz, $CDCl_3$) spectra of 1-ethyl-4-(2-phenyl-1-(*p*-tolyl)prop-1-en-1-yl)quinolin-2(1*H*)-one (**6m**)

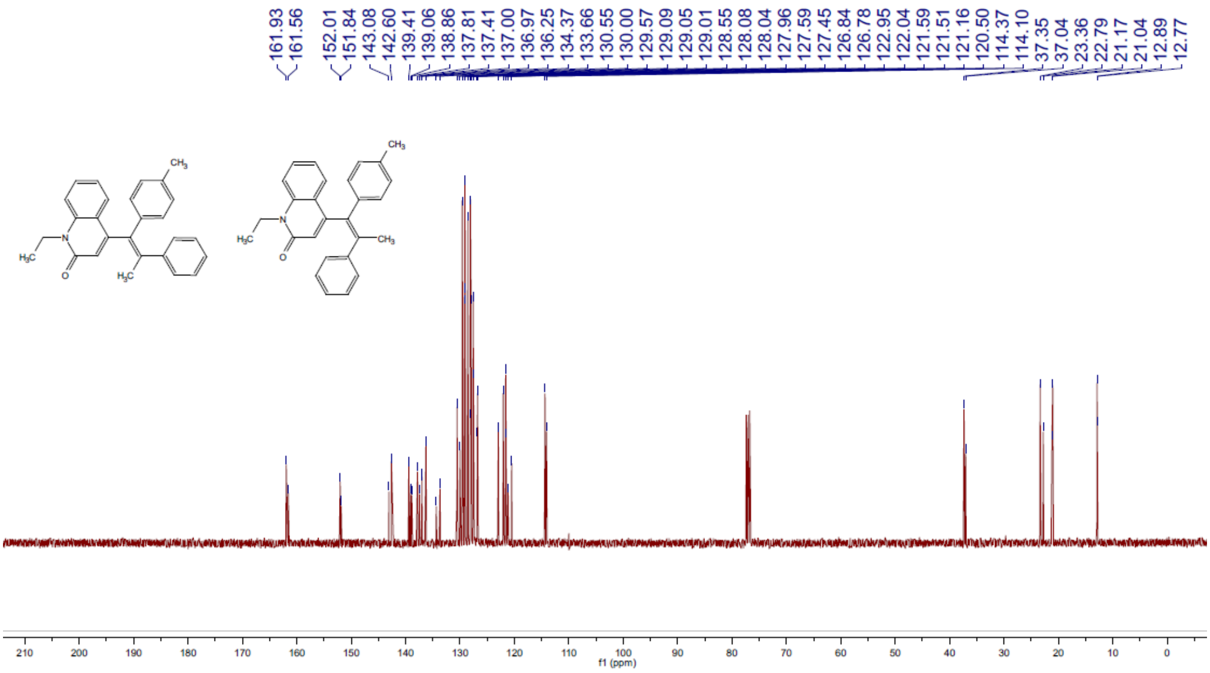

Supplementary Figure 161. ^{13}C NMR (100 MHz, $CDCl_3$) spectra of 1-ethyl-4-(2-phenyl-1-(*p*-tolyl)prop-1-en-1-yl)quinolin-2(1*H*)-one (**6m**)

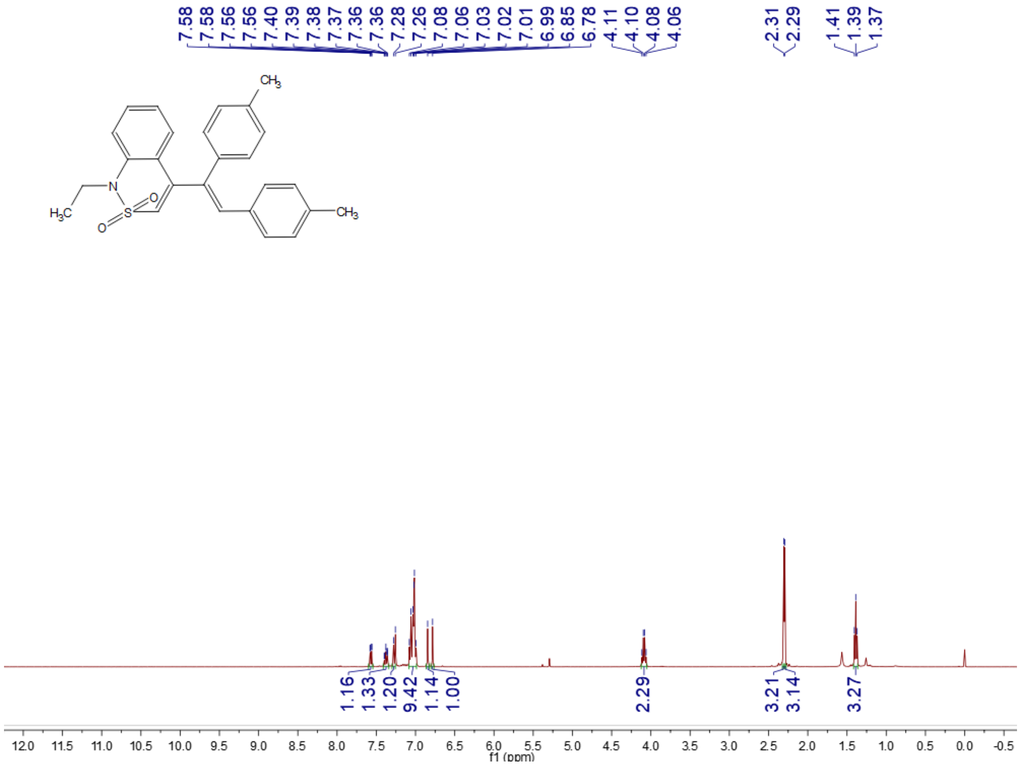

Supplementary Figure 162. ^{1}H NMR (400 MHz, $CDCl_3$) spectra of (*E*)-4-(1,2-di-*p*-tolylvinyl)-1-ethyl-1*H*-benzo[c][1,2]thiazine 2,2-dioxide (**(*E*)-6n)**

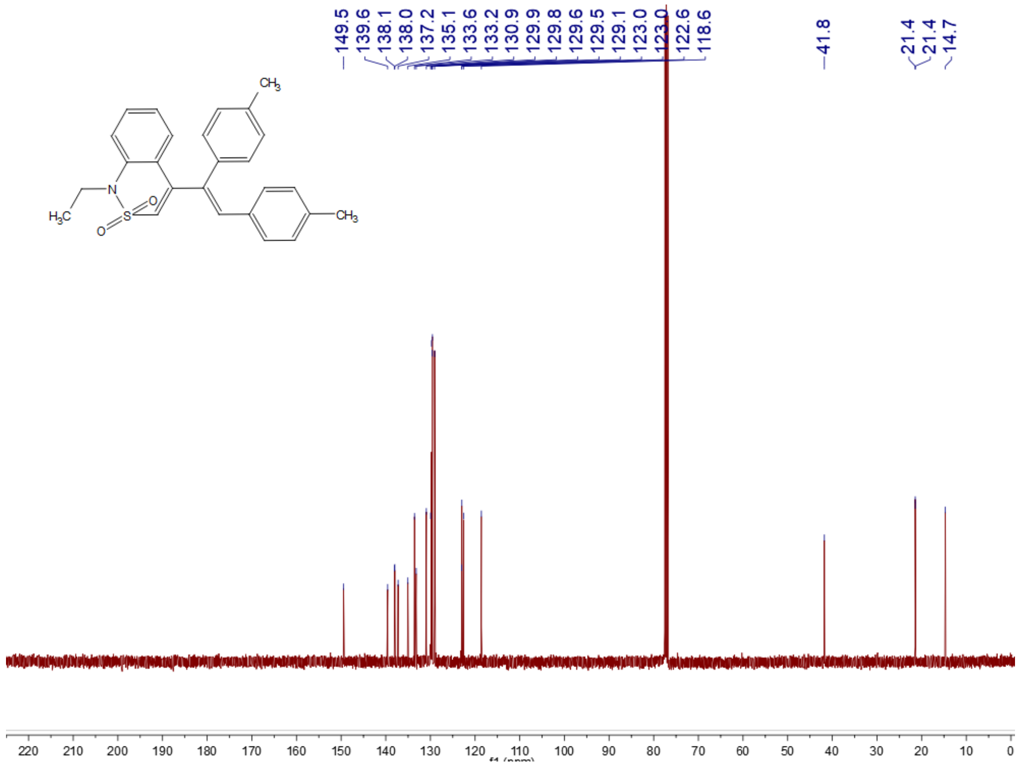

Supplementary Figure 163. ^{13}C NMR (100 MHz, $CDCl_3$) spectra of (*E*)-4-(1,2-di-*p*-tolylvinyl)-1-ethyl-1H-benzo[c][1,2]thiazine 2,2-dioxide (**(*E*)-6n)**

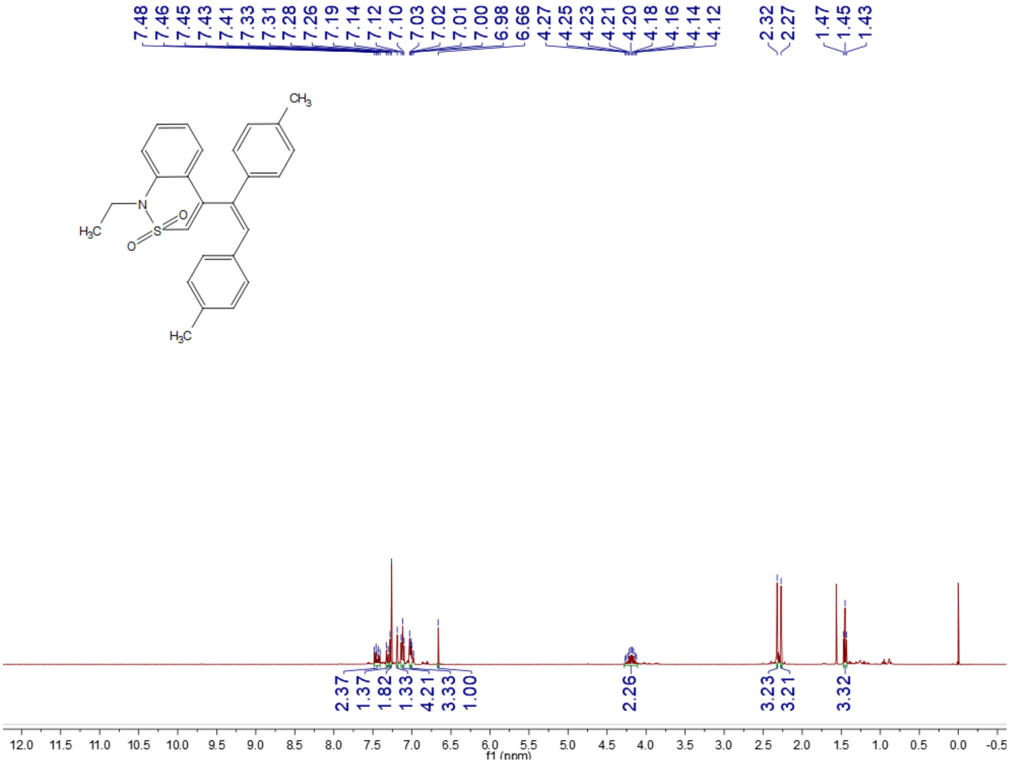

Supplementary Figure 164. ^{1}H NMR (400 MHz, $CDCl_3$) spectra of (*Z*)-4-(1,2-di-*p*-tolylvinyl)-1-ethyl-1H-benzo[*c*][1,2]thiazine 2,2-dioxide (**(*Z*)-6n**)

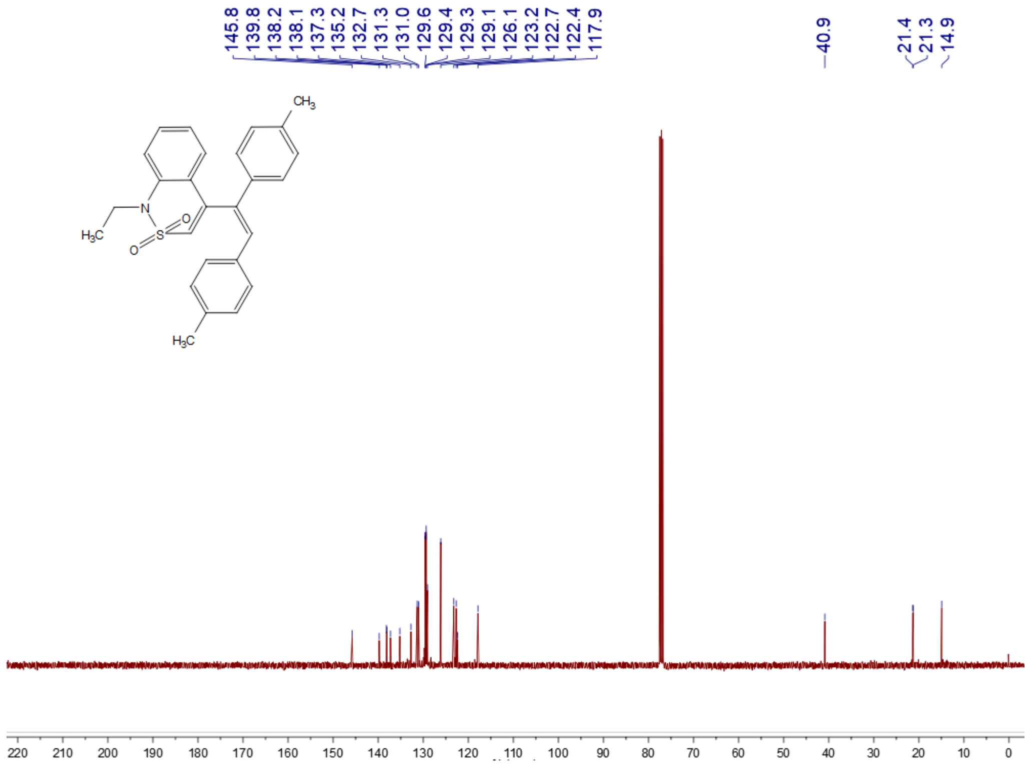

Supplementary Figure 165. ^{13}C NMR (100 MHz, $CDCl_3$) spectra of (*Z*)-4-(1,2-di-*p*-tolylvinyl)-1-ethyl-1*H*-benzo[*c*][1,2]thia*Z*ine 2,2-dioxide (**(*Z*)-6n**)

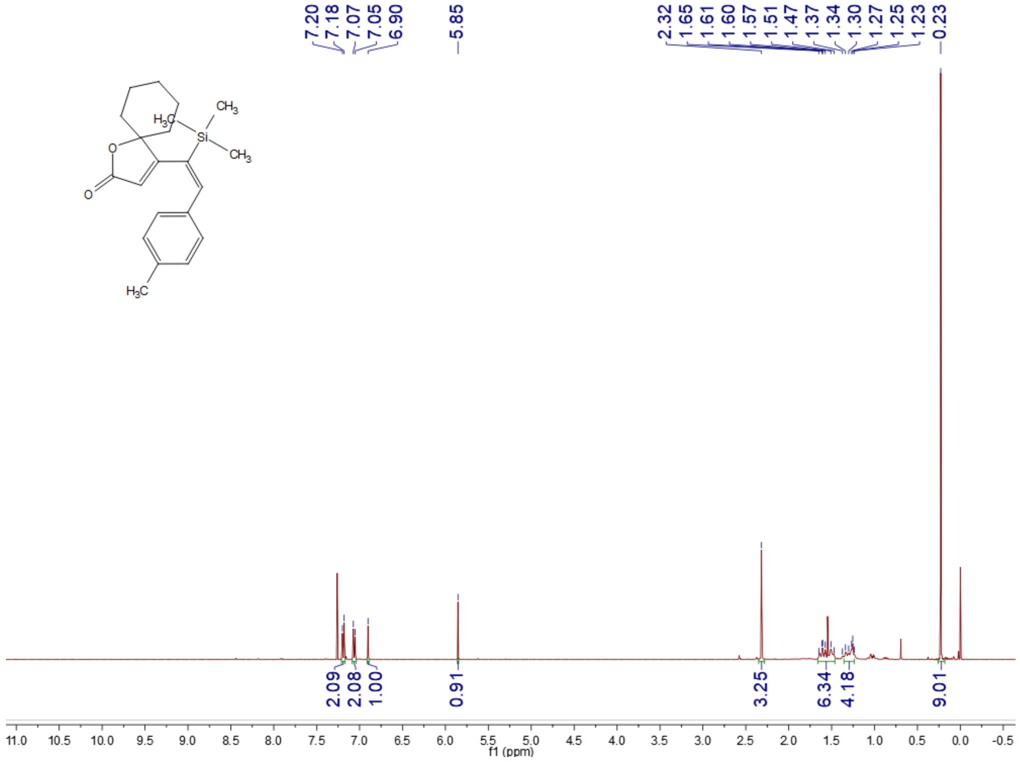

Supplementary Figure 166. ^{1}H NMR (400 MHz, $CDCl_3$) spectra of (*E*)-4-(2-(*p*-tolyl)-1-(trimethylsilyl)vinyl)-1-oxaspiro[4.5]dec-3-en-2-one (**(*E*)-6o**)

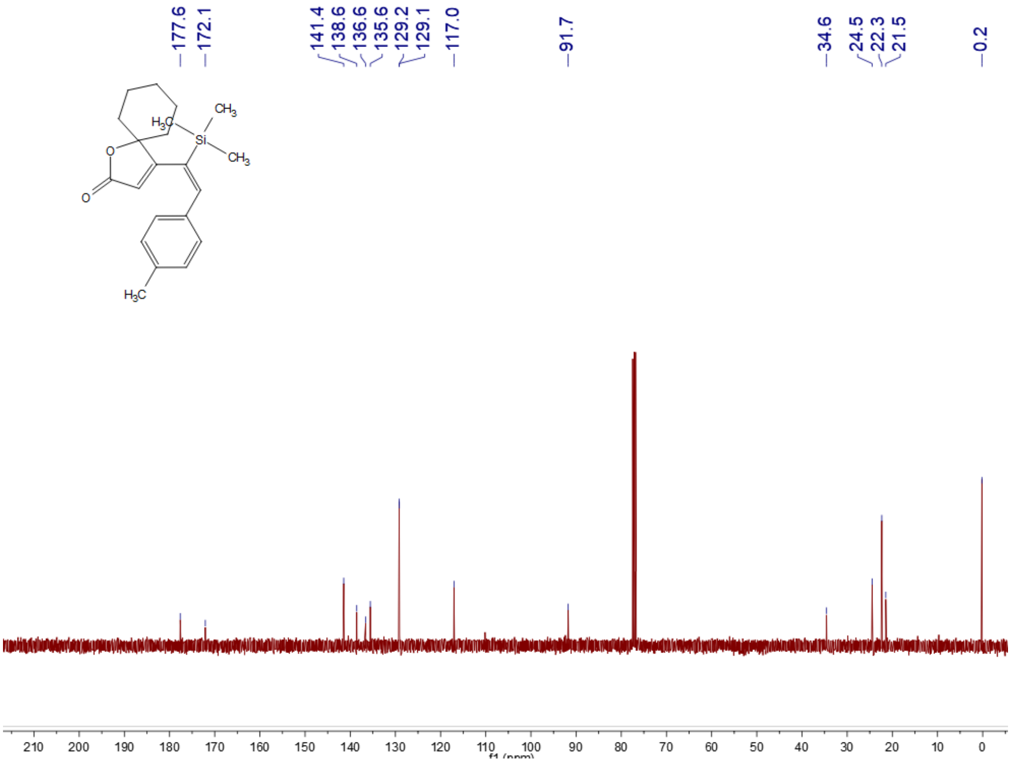

Supplementary Figure 167. ^{13}C NMR (100 MHz, $CDCl_3$) spectra of (*E*)-4-(2-(*p*-tolyl)-1-(trimethylsilyl)vinyl)-1-oxaspiro[4.5]dec-3-en-2-one (**(*E*)-6o**)

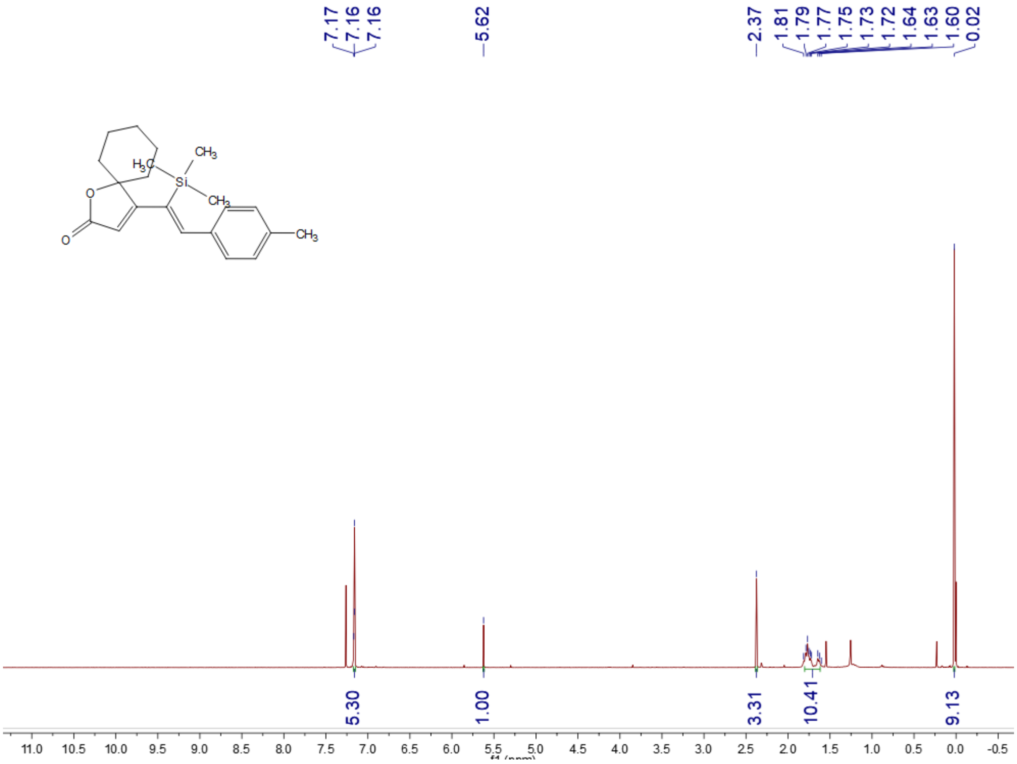

Supplementary Figure 168. ^{1}H NMR (400 MHz, $CDCl_3$) spectra of (*Z*)-4-(2-(*p*-tolyl)-1-(trimethylsilyl)vinyl)-1-oxaspiro[4.5]dec-3-en-2-one (**(*Z*)-6o**)

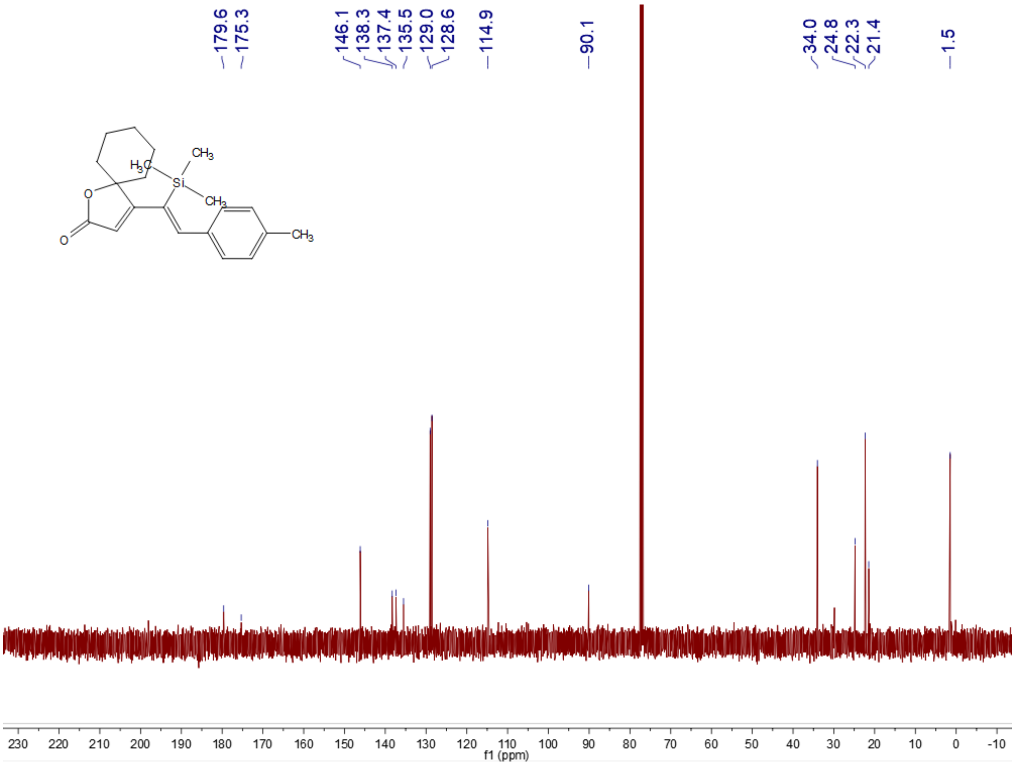

Supplementary Figure 169. ^{13}C NMR (100 MHz, $CDCl_3$) spectra of (*Z*)-4-(2-(*p*-tolyl)-1-(trimethylsilyl)vinyl)-1-oxaspiro[4.5]dec-3-en-2-one (**(*Z*)-6o**)

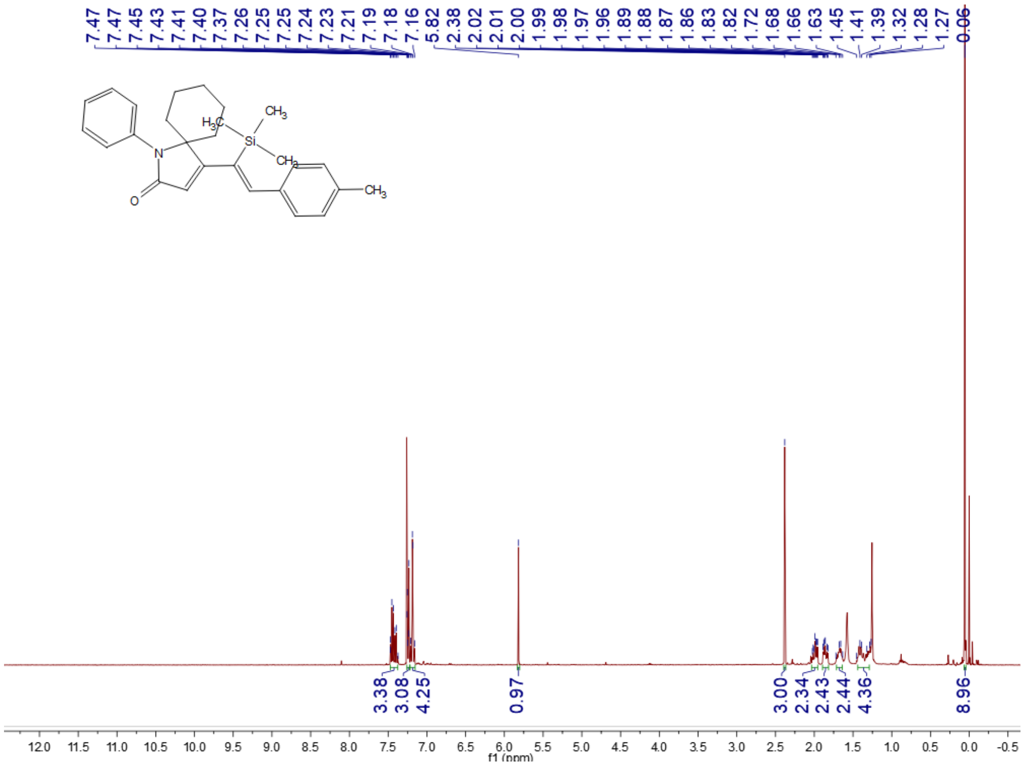

Supplementary Figure 170. ^{1}H NMR (400 MHz, $CDCl_3$) spectra of (*Z*)-1-phenyl-4-(2-(*p*-tolyl)-1-(trimethylsilyl)vinyl)-1-azaspiro[4.5]dec-3-en-2-one (**6p**)

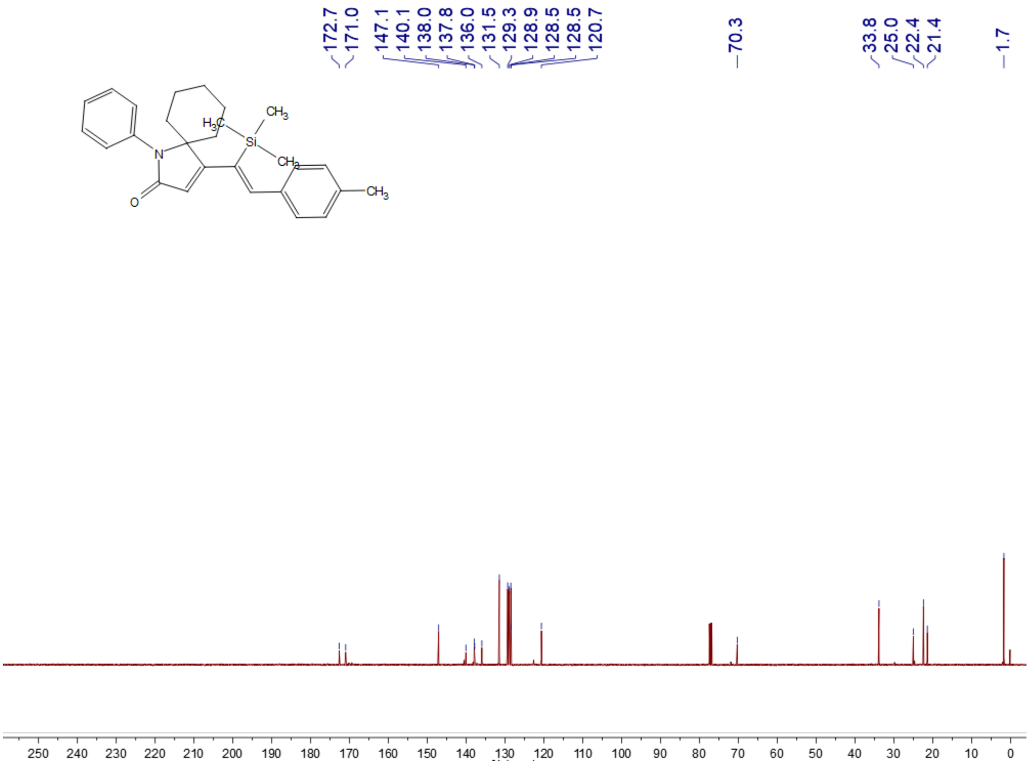

Supplementary Figure 171. ^{13}C NMR (100 MHz, $CDCl_3$) spectra of (*Z*)-1-phenyl-4-(2-(*p*-tolyl)-1-(trimethylsilyl)vinyl)-1-azaspiro[4.5]dec-3-en-2-one (**6p**)

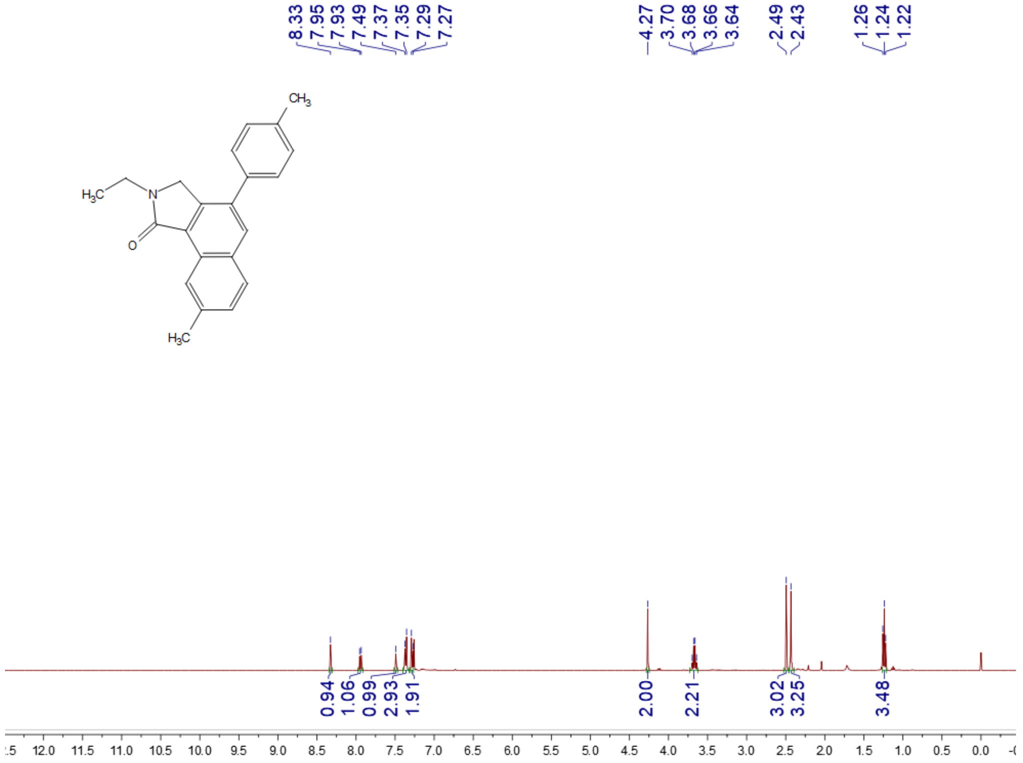

Supplementary Figure 172. ^{1}H NMR (400 MHz, $CDCl_3$) spectra of 2-ethyl-8-methyl-4-(*p*-tolyl)-2,3-dihydro-1*H*-benzo[*e*]isoindol-1-one (**6q′**)

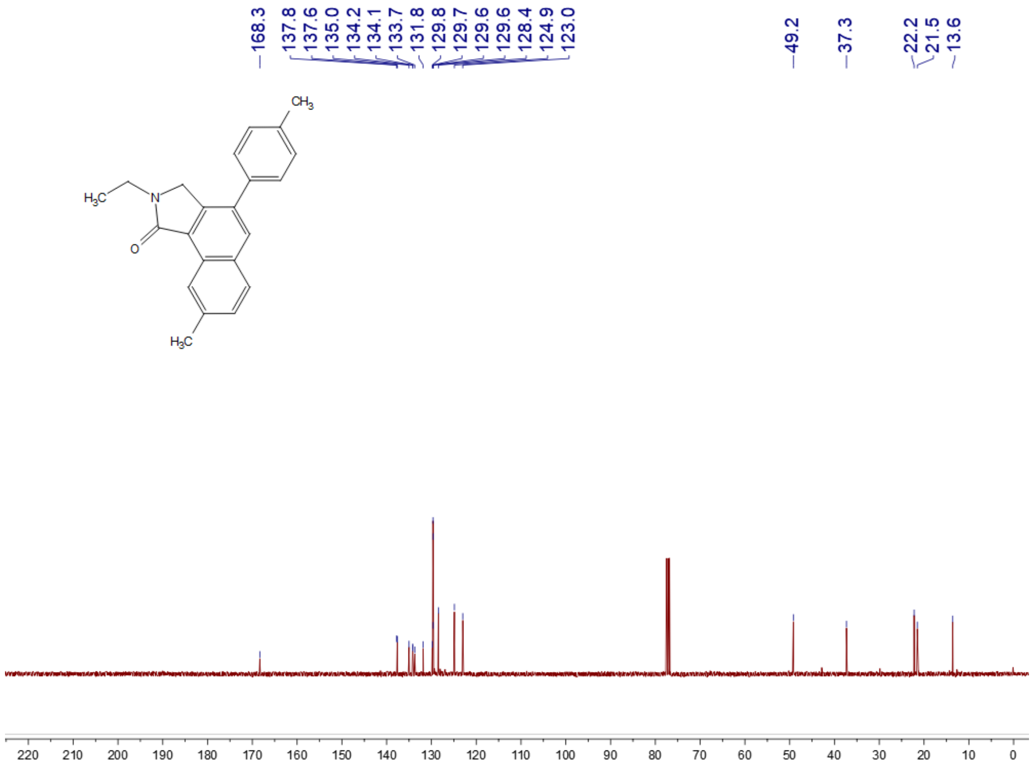

Supplementary Figure 173. ^{13}C NMR (100 MHz, $CDCl_3$) spectra of 2-ethyl-8-methyl-4-(*p*-tolyl)-2,3-dihydro-1*H*-benzo[*e*]isoindol-1-one (**6q′**)

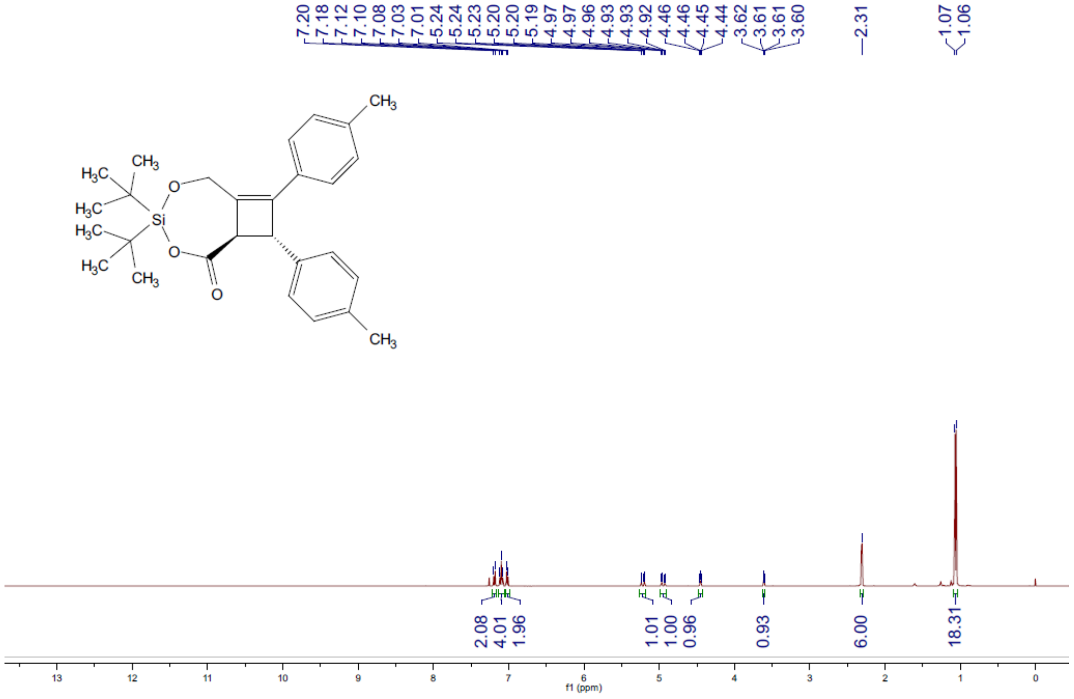

Supplementary Figure 174. ^{1}H NMR (400 MHz, $CDCl_3$) spectra of 4,4-di-*tert*-butyl-8,9-di-*p*-tolyl-3,5-dioxa-4-silabicyclo[5.2.0]no*N*-7-en-2-one (**5r**)

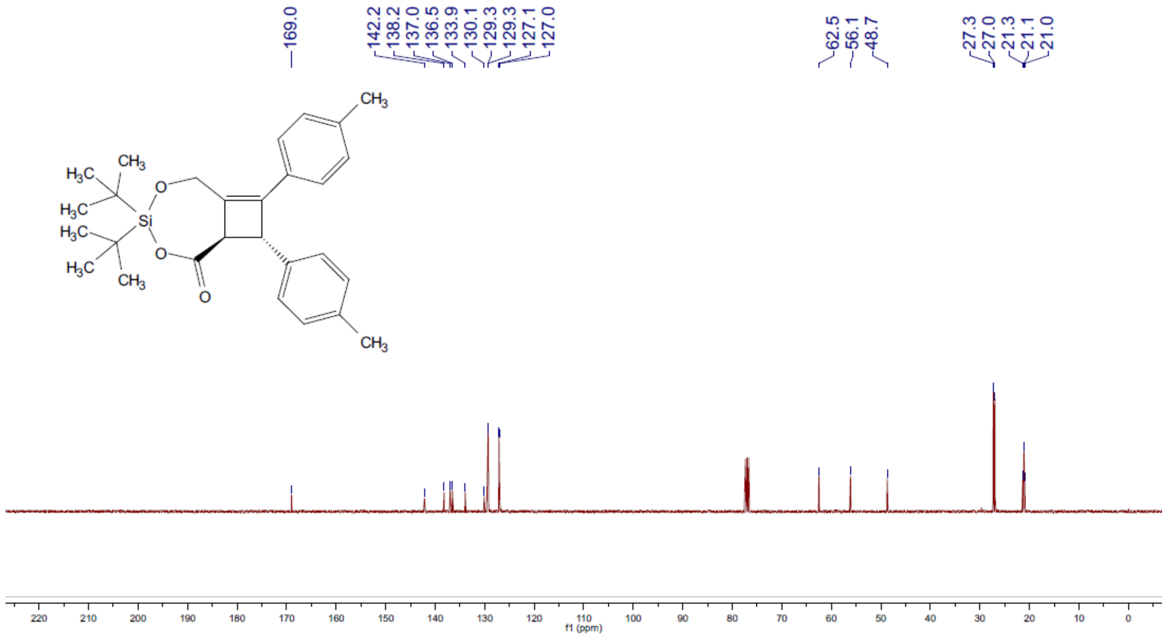

Supplementary Figure 175. ^{13}C NMR (100 MHz, $CDCl_3$) spectra of 4,4-di-*tert*-butyl-8,9-di-*p*-tolyl-3,5-dioxa-4-silabicyclo[5.2.0]no*N*-7-en-2-one (**5r**)

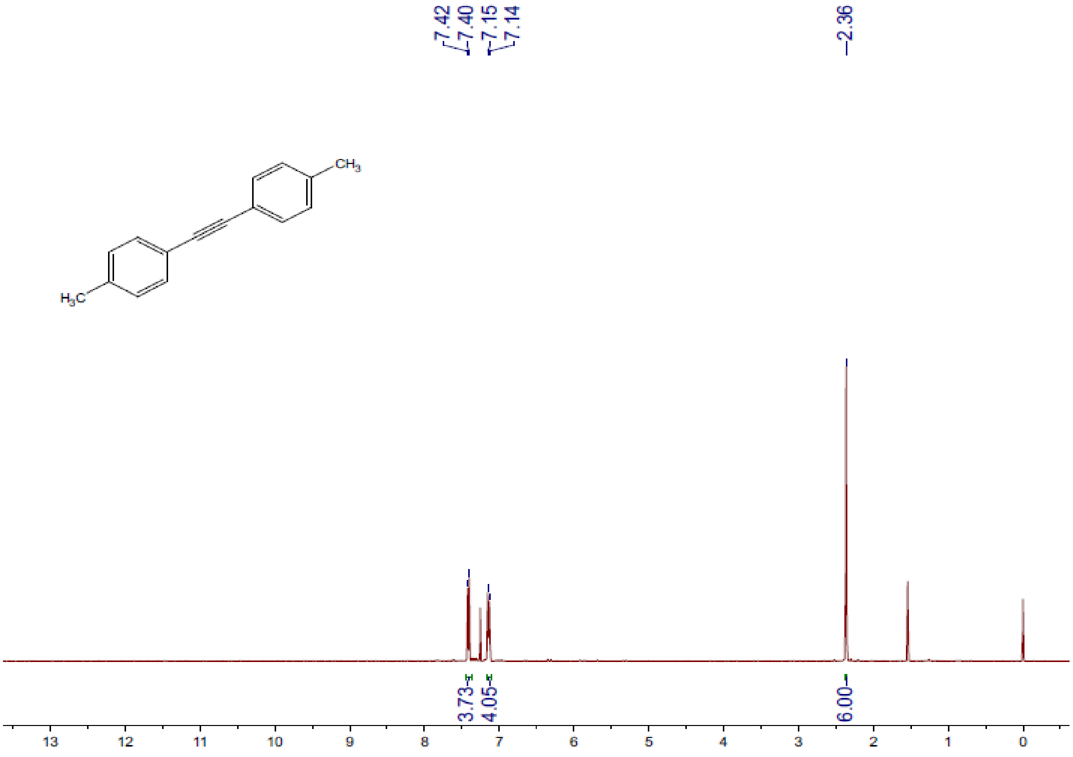

Supplementary Figure 176. ^{1}H NMR (400 MHz, $CDCl_3$) spectra of di(*p*-tolyl)acetylene (**1a**)

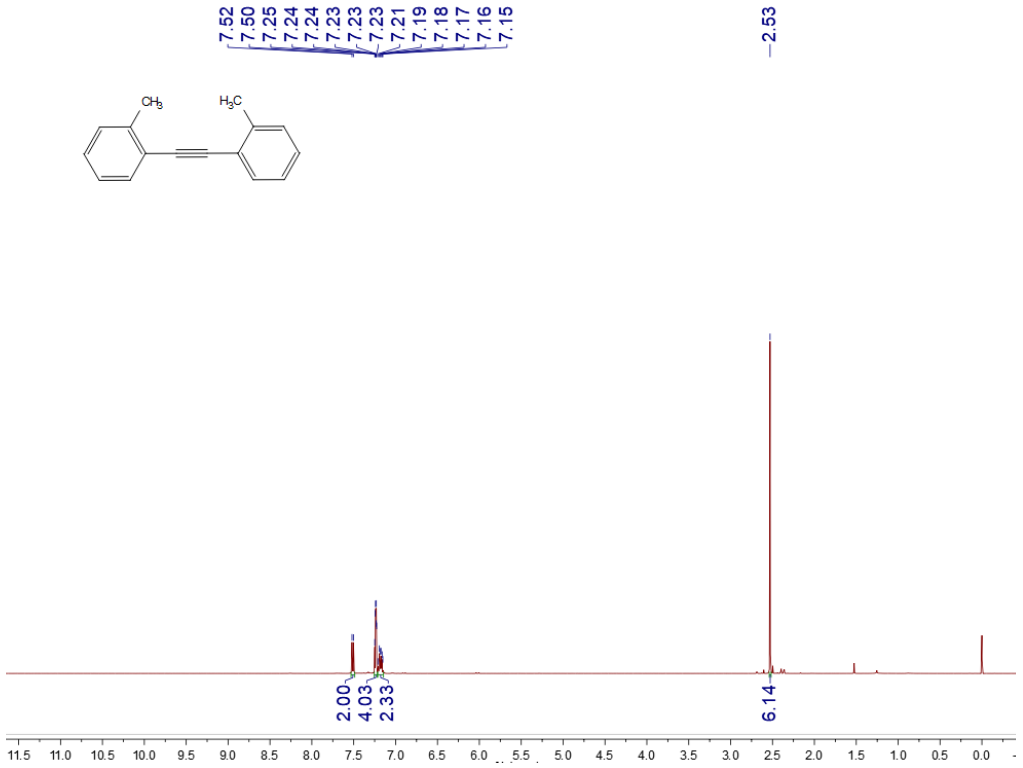

Supplementary Figure 177. ^{1}H NMR (400 MHz, $CDCl_3$) spectra of 1,2-di-o-tolylethyne (**1b**)

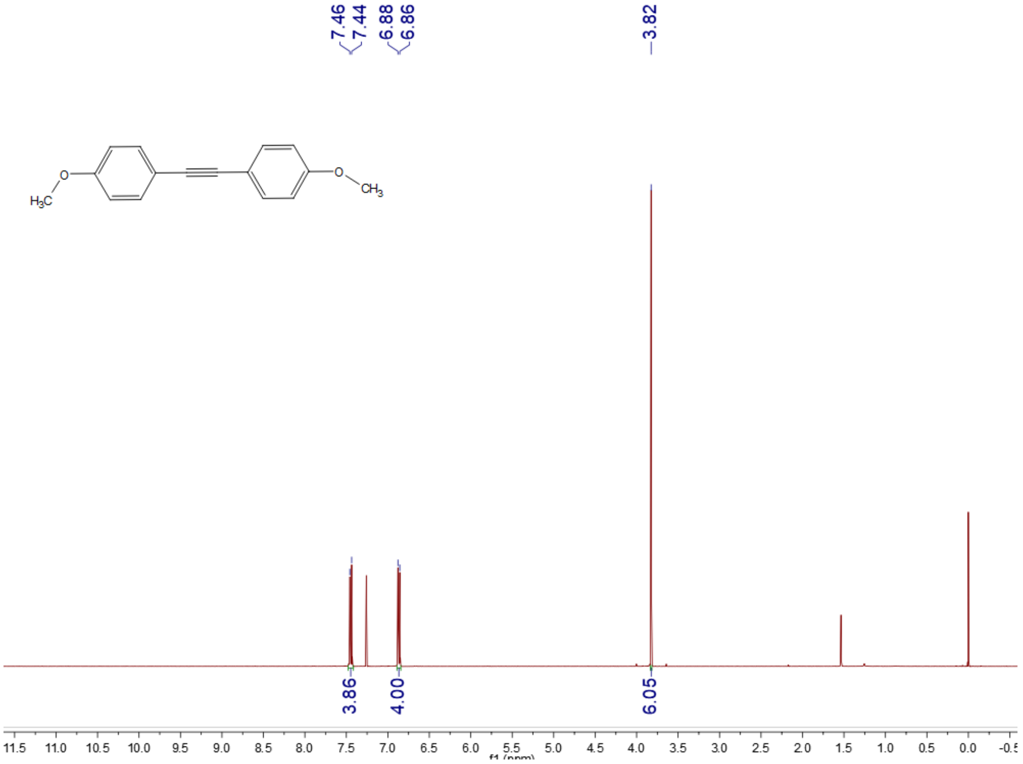

Supplementary Figure 178. ^{1}H NMR (400 MHz, $CDCl_3$) spectra of 1,2-bis(4-methoxyphenyl)ethyne (**1d**)

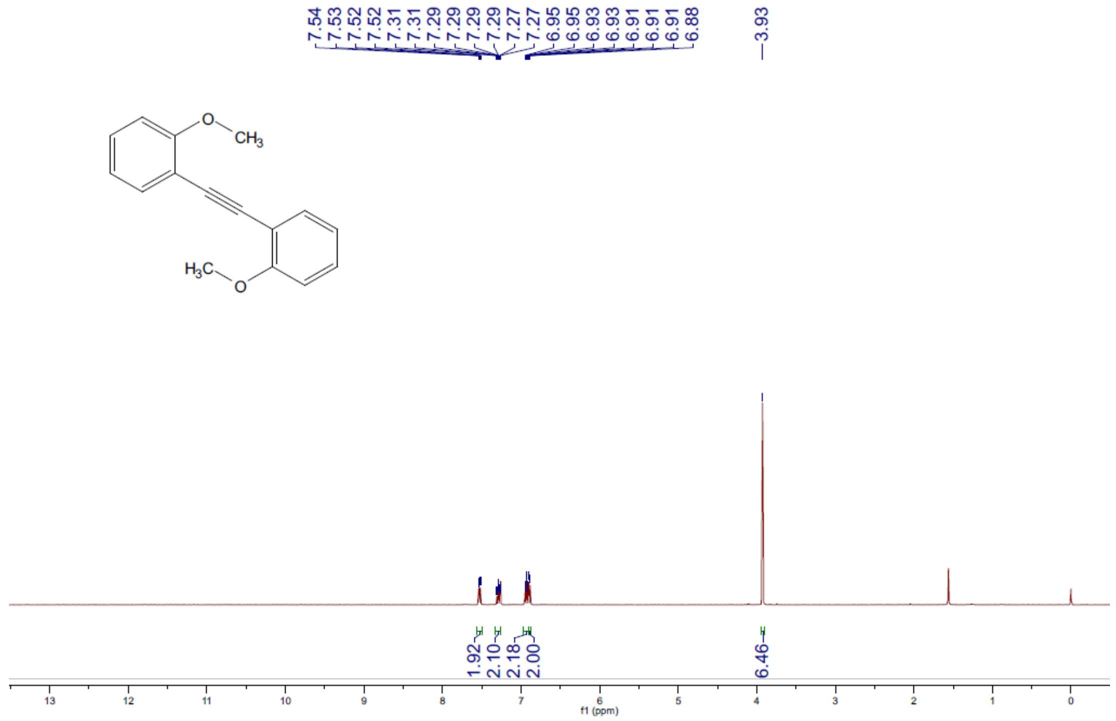

Supplementary Figure 179. ^{1}H NMR (400 MHz, $CDCl_3$) spectra of 1,2-bis(2-methoxyphenyl)ethyne (**1e**)

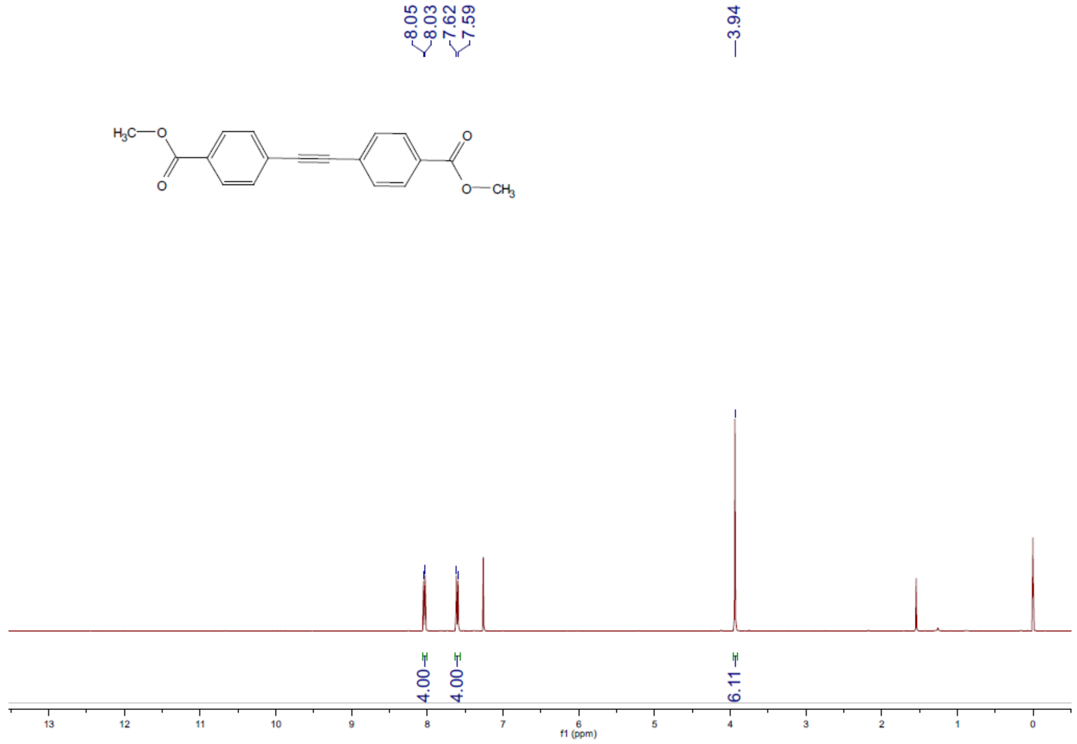

Supplementary Figure 180. ^{1}H NMR (400 MHz, $CDCl_3$) spectra of Dimethyl 4,4'-(ethyne-1,2-diyl)dibenzoate (**1f**)

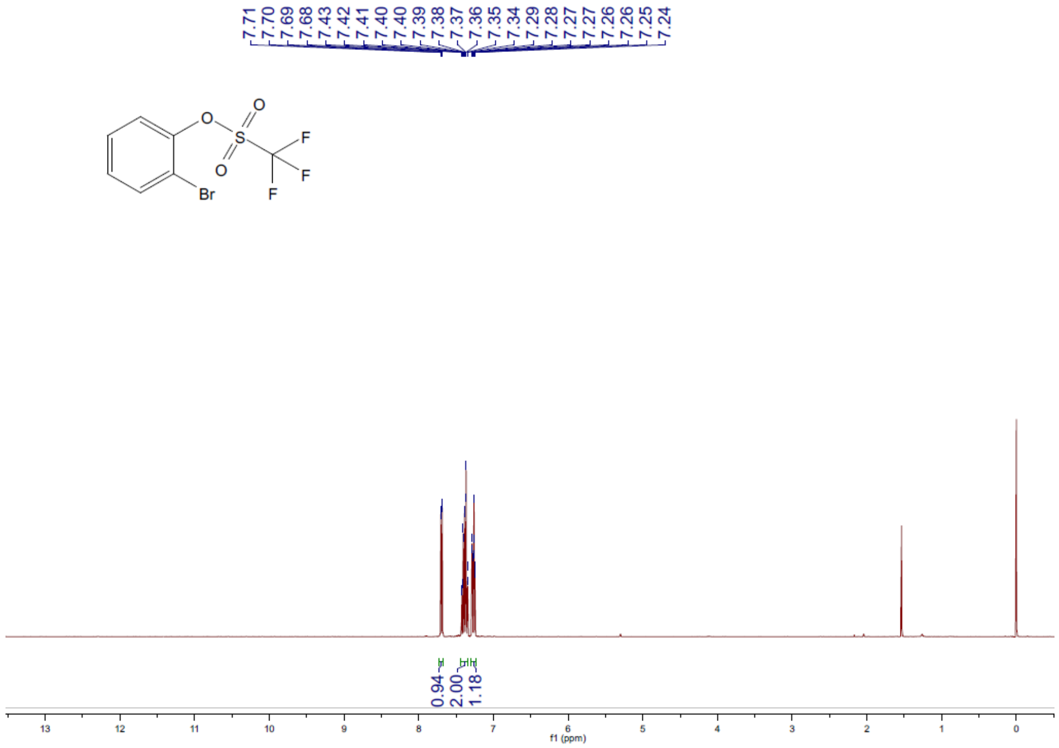

Supplementary Figure 181. ^{1}H NMR (400 MHz, $CDCl_3$) spectra of 2-bromophenyl trifluoromethanesulfonate (**1′g**)

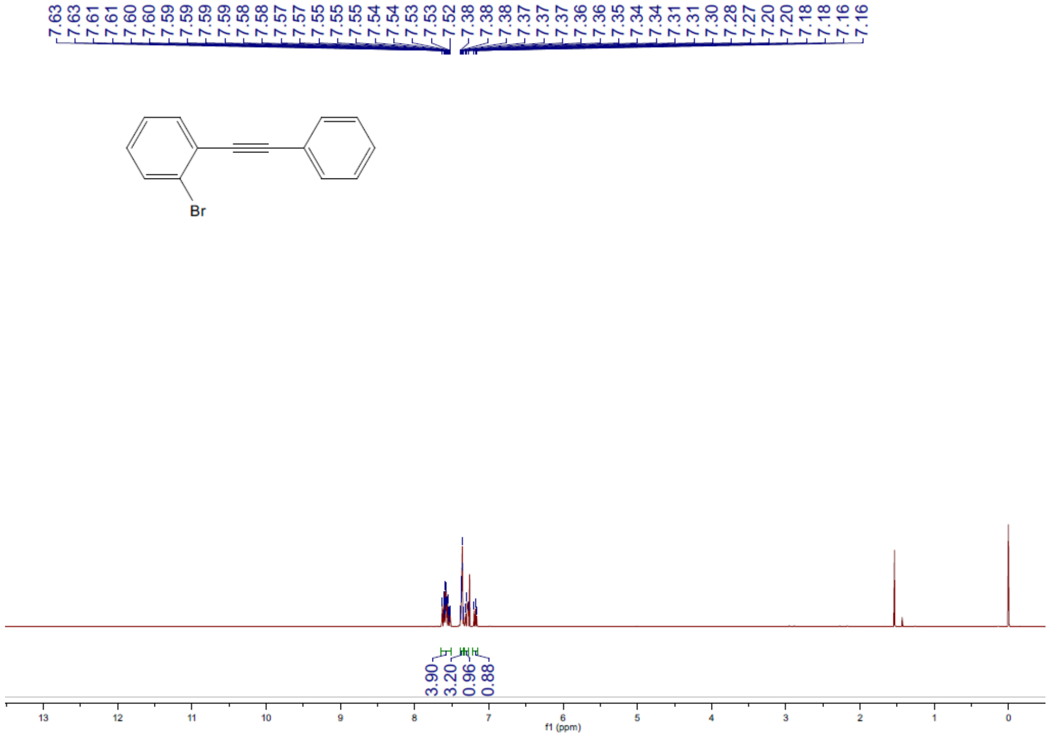

Supplementary Figure 182. ^{1}H NMR (400 MHz, $CDCl_3$) spectra of 1-bromo-2-(phenylethynyl)benzene (**1g**)

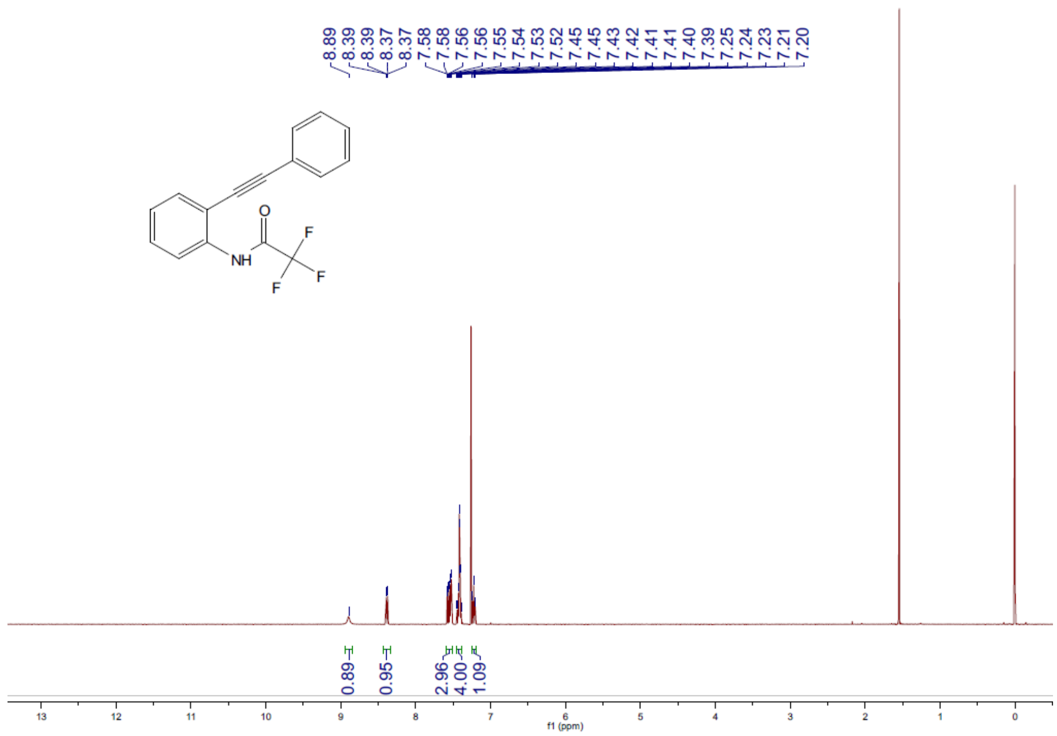

Supplementary Figure 183. ^{1}H NMR (400 MHz, $CDCl_3$) spectra of 2,2,2-trifluoro-*N*-(2-(phenylethynyl)phenyl)acetamide (**1h**)

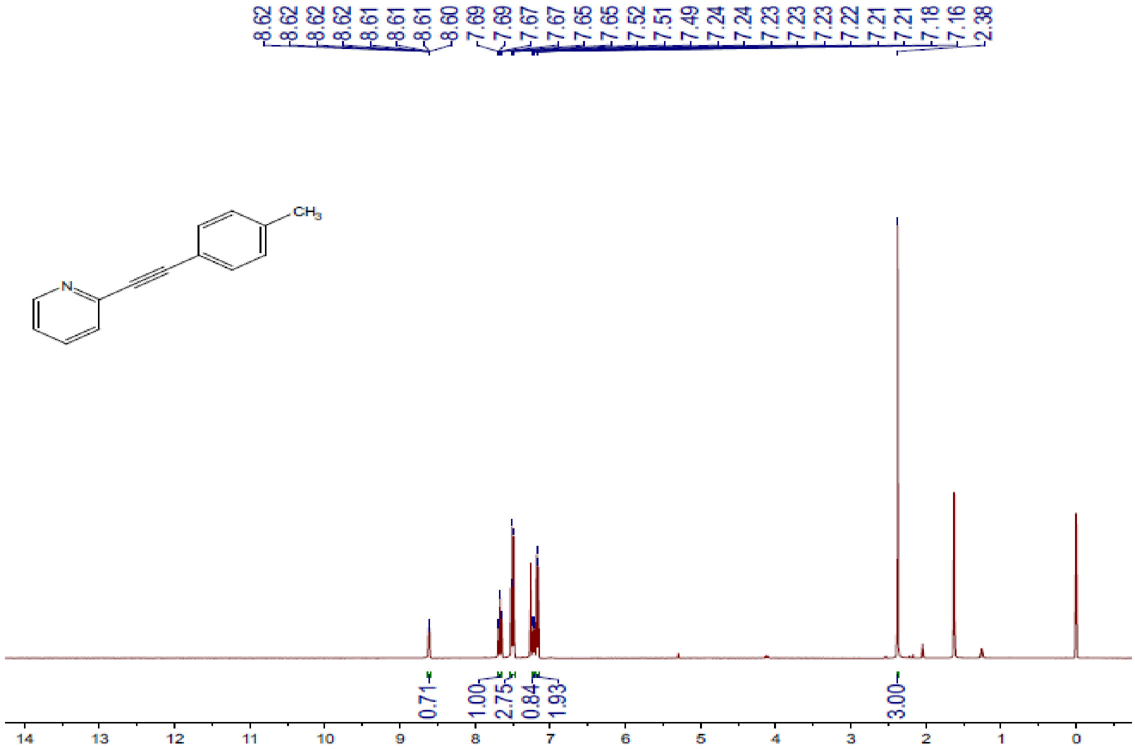

Supplementary Figure 184. ^{1}H NMR (400 MHz, $CDCl_3$) spectra of 2-(*p*-tolylethynyl)pyridine (**1i**)

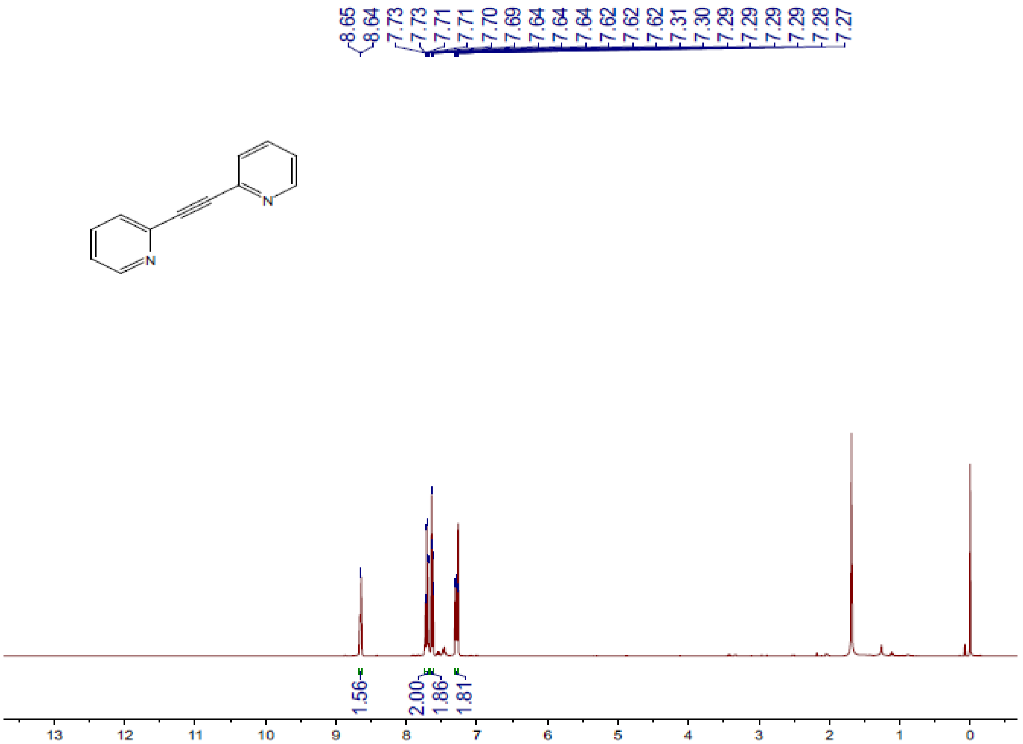

Supplementary Figure 185. ^{1}H NMR (400 MHz, $CDCl_3$) spectra of 1,2-di(pyridin-2-yl)ethyne (**1j**)

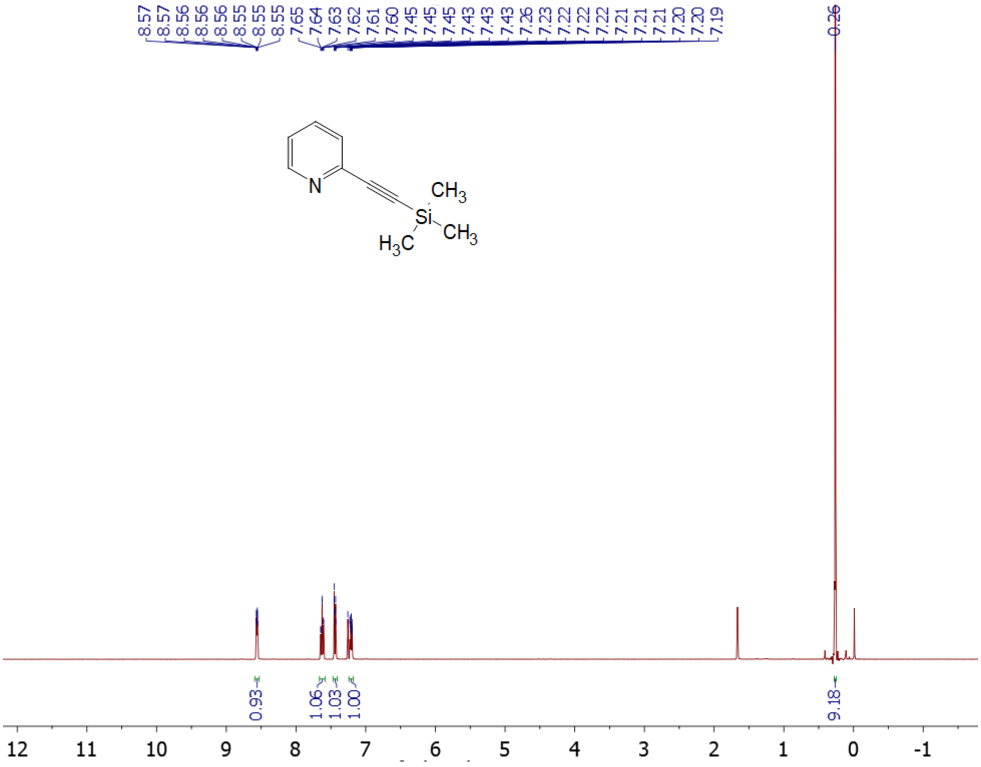

Supplementary Figure 186. ^{1}H NMR (400 MHz, $CDCl_3$) spectra of 2-((trimethylsilyl)ethynyl)pyridine (**1′k**)

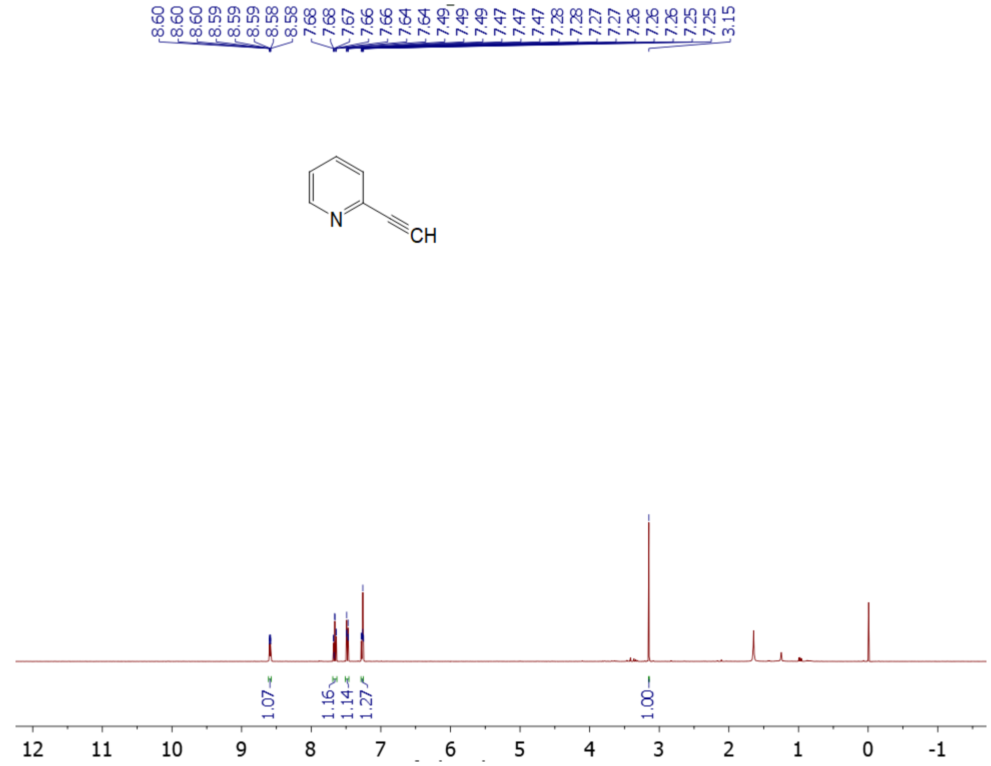

Supplementary Figure 187. ^{1}H NMR (400 MHz, $CDCl_3$) spectra of 2-ethynylpyridine (**1′′k**)

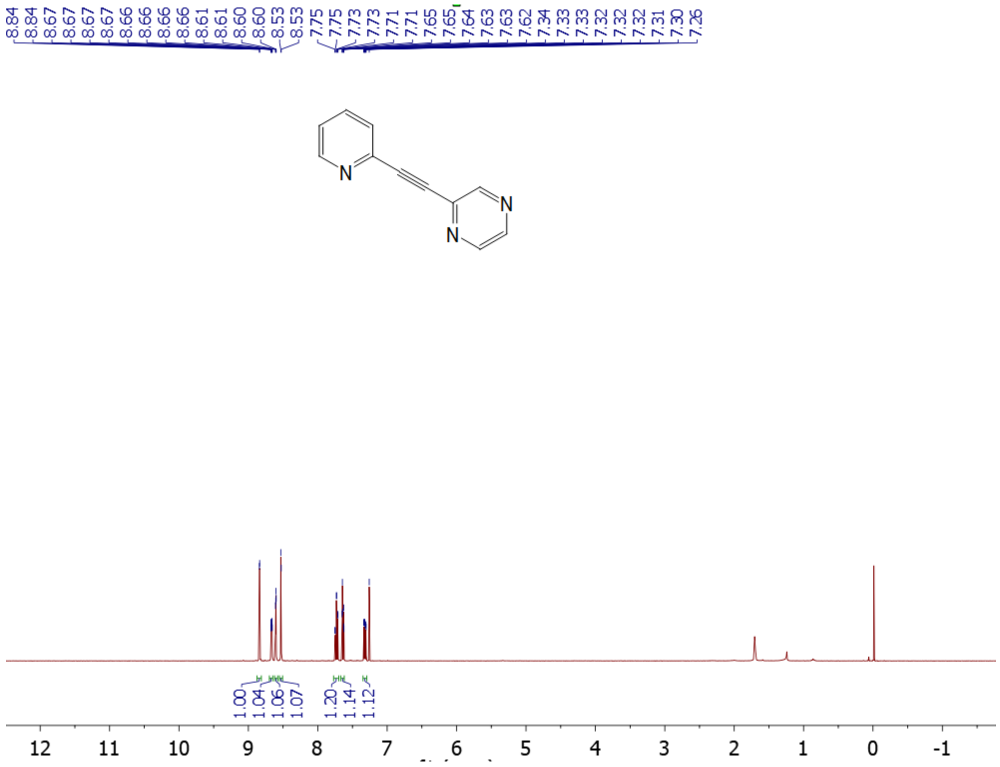

Supplementary Figure 188. ^{1}H NMR (400 MHz, $CDCl_3$) spectra of 2-(pyridin-2-ylethynyl)pyrazine (**1k**)

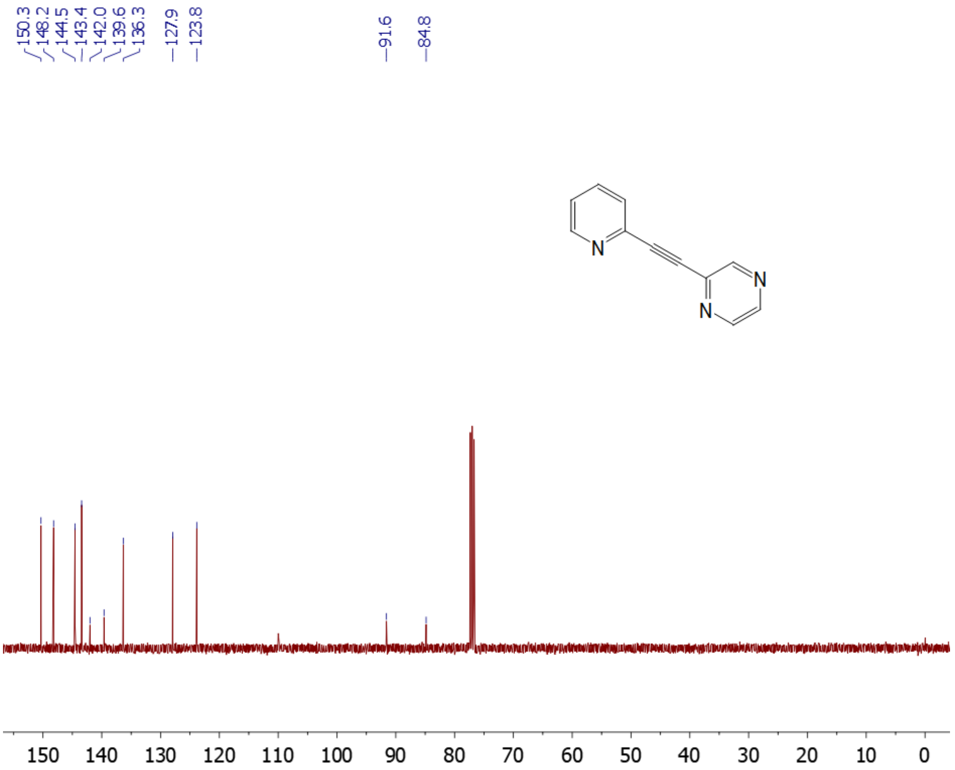

Supplementary Figure 189. ^{13}C NMR (100 MHz, $CDCl_3$) spectra of 2-(pyridin-2-ylethynyl)pyrazine (**1k**)

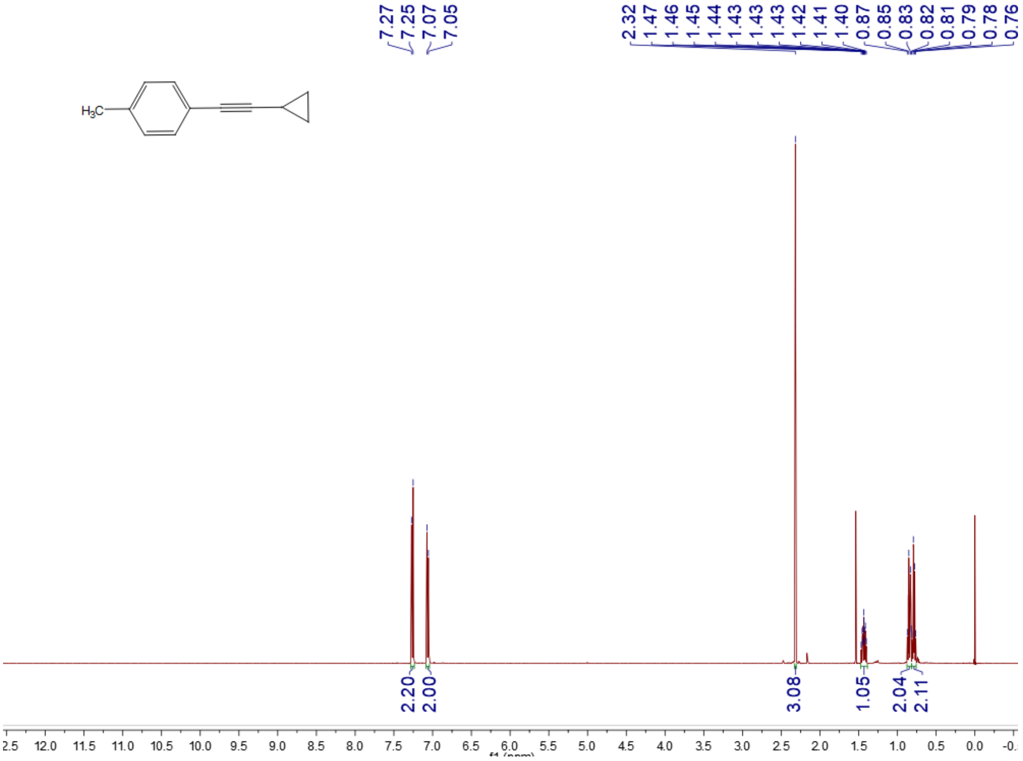

Supplementary Figure 190. ^{1}H NMR (400 MHz, $CDCl_3$) spectra of 1-(cyclopropylethynyl)-4-methylbenzene (**1l**)

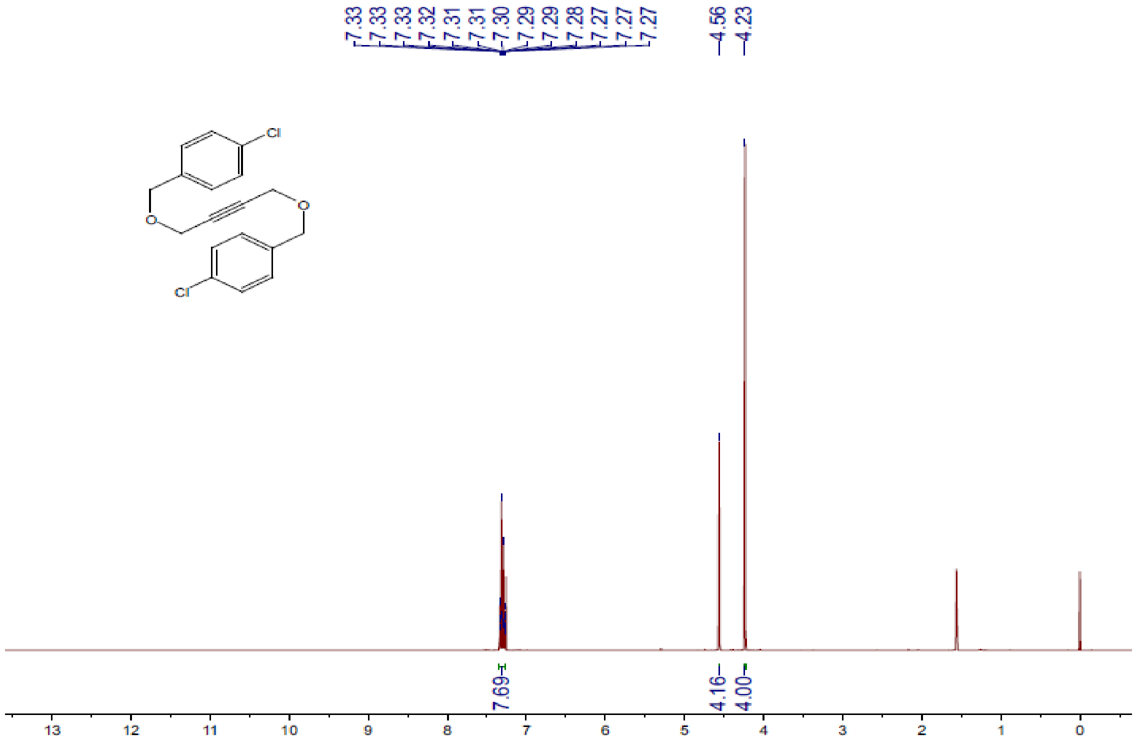

Supplementary Figure 191. ^{1}H NMR (400 MHz, $CDCl_3$) spectra of 1,4-bis((4-chlorobenzyl)oxy)but-2-yne (**1m**)

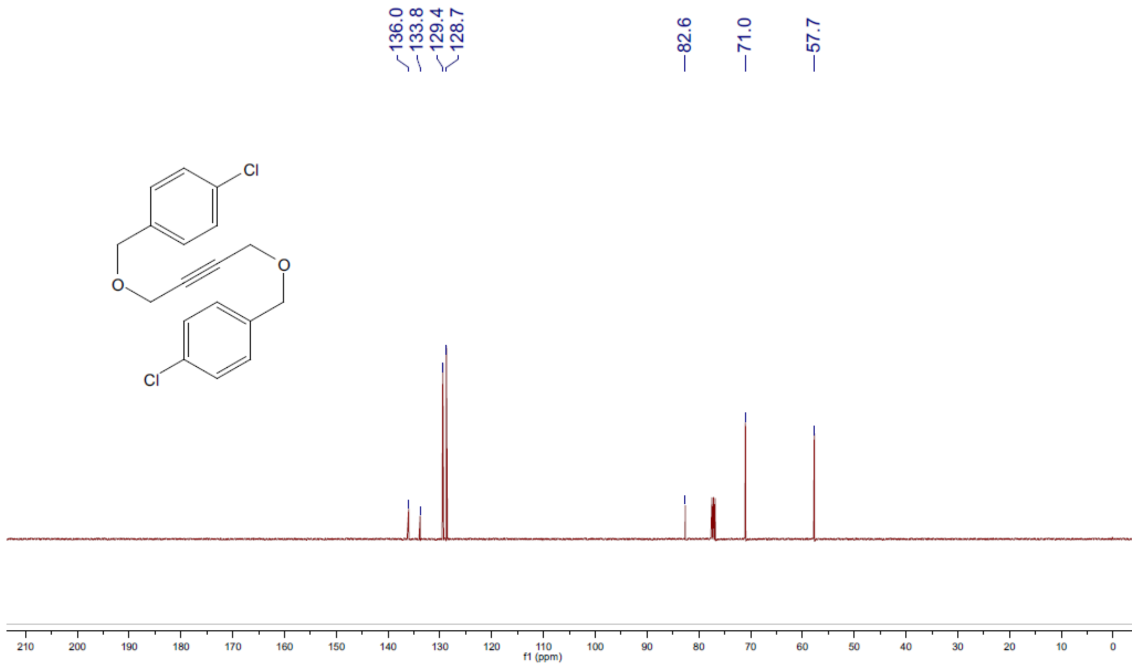

Supplementary Figure 192. ^{13}C NMR (100 MHz, $CDCl_3$) spectra of 1,4-bis((4-chlorobenzyl)oxy)but-2-yne (**1m**)

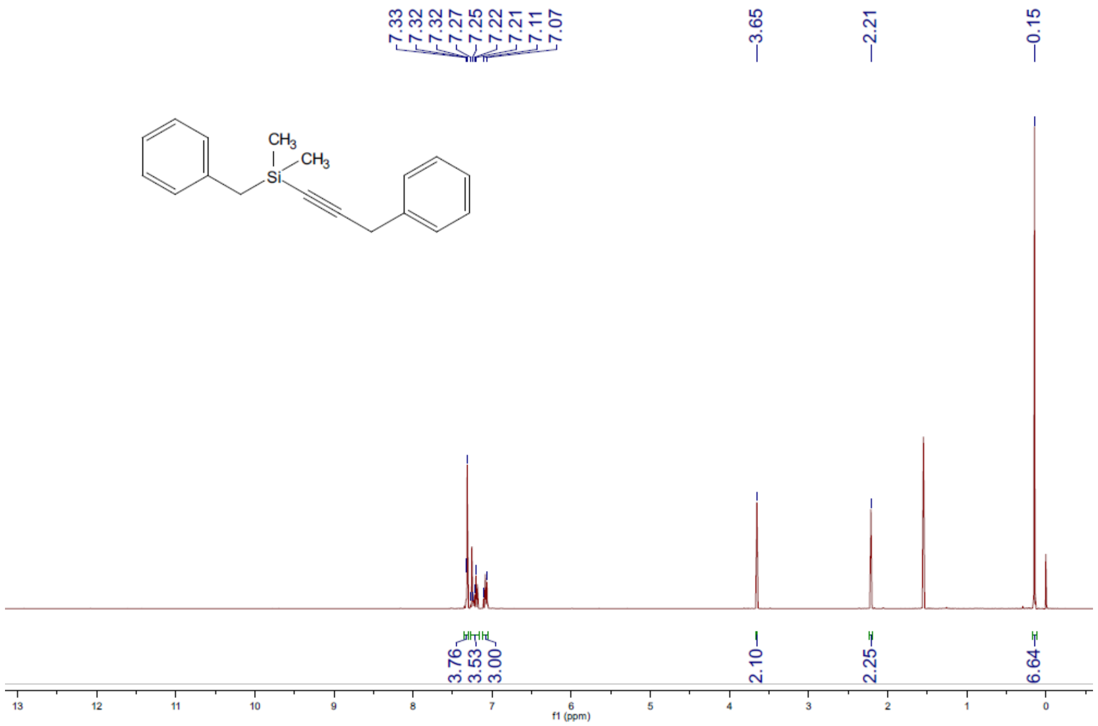

Supplementary Figure 193. ^{1}H NMR (400 MHz, $CDCl_3$) spectra of benzyldimethyl(3-phenylprop-1-yn-1-yl)silane (**1o**)

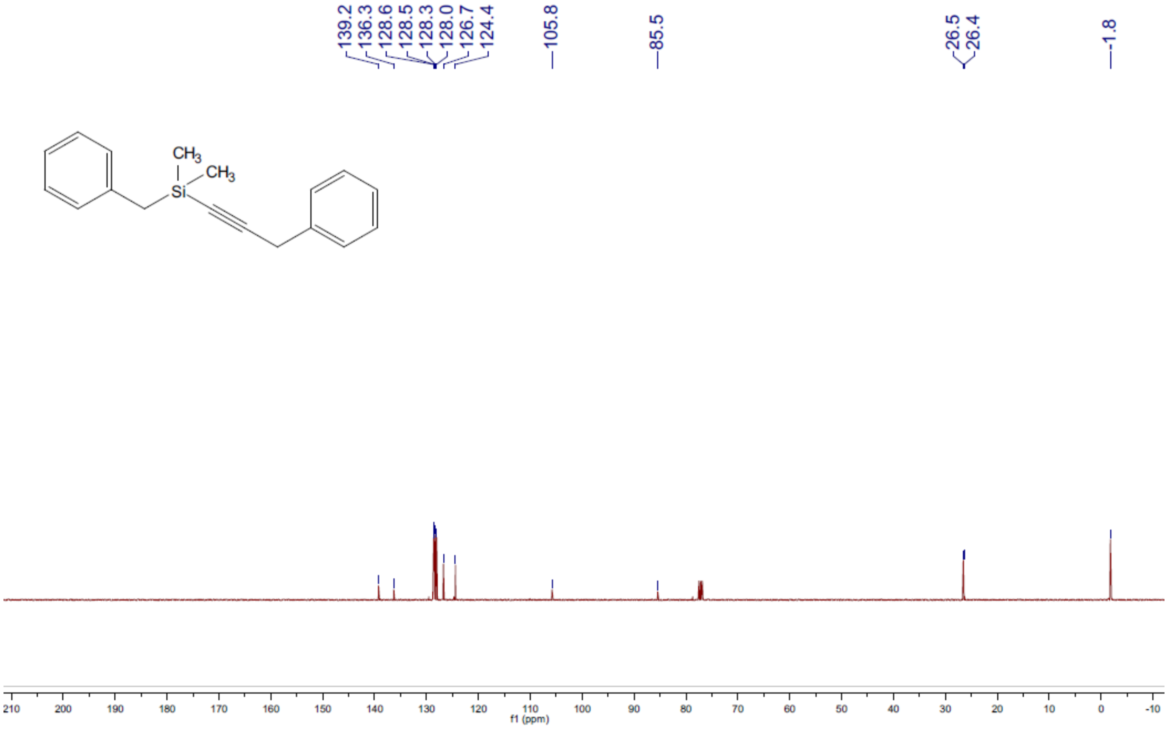

Supplementary Figure 194. ^{1}H NMR (400 MHz, $CDCl_3$) spectra of benzyldimethyl(3-phenylprop-1-yn-1-yl)silane (**1o**)

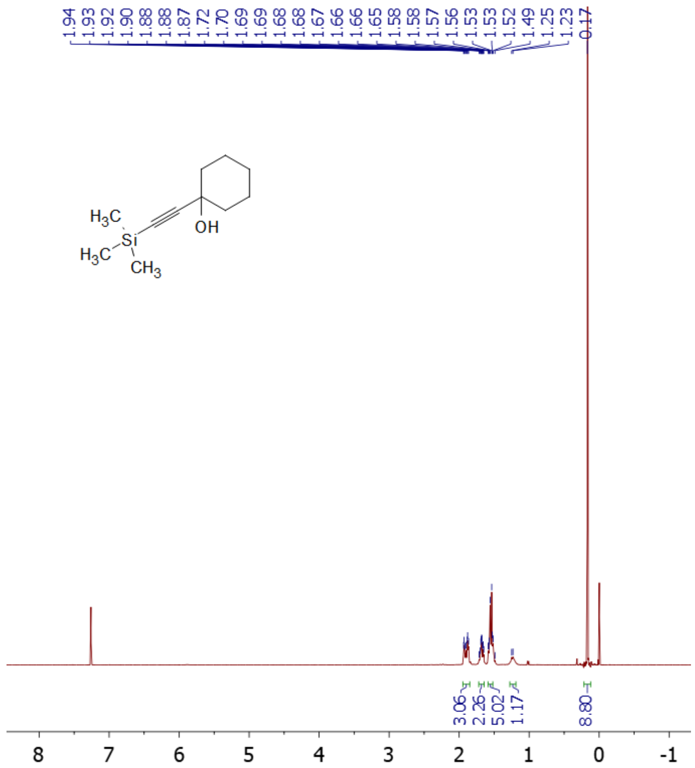

Supplementary Figure 195. ^{1}H NMR (400 MHz, $CDCl_3$) spectra of 1-((trimethylsilyl)ethynyl)cyclohexan-1-ol (**1p**)

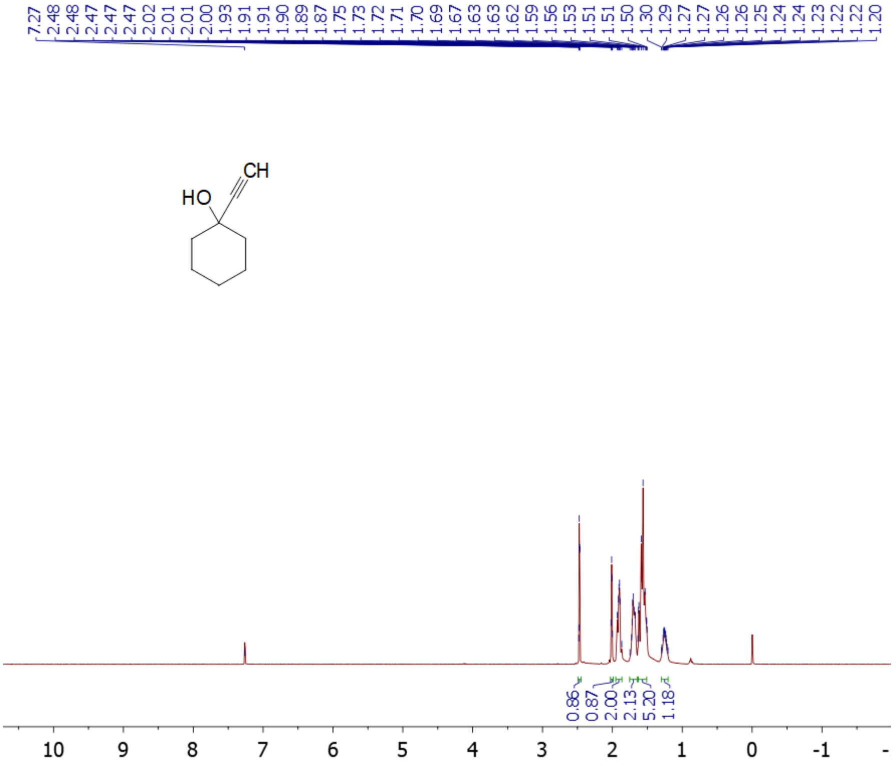

Supplementary Figure 196. ^{1}H NMR (400 MHz, $CDCl_3$) spectra of 1-ethynylcyclohexan-1-ol (**1q**)

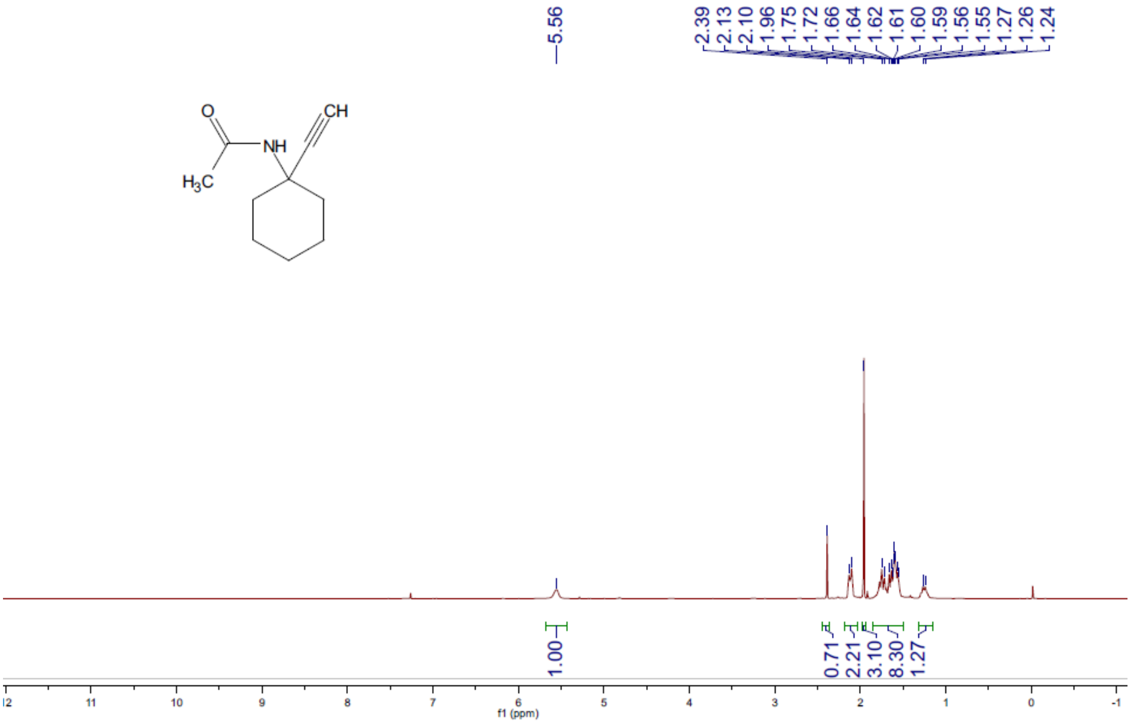

Supplementary Figure 197. ^{1}H NMR (400 MHz, $CDCl_3$) spectra of *N*-(1-ethynylcyclohexyl)acetamide (**1r**)

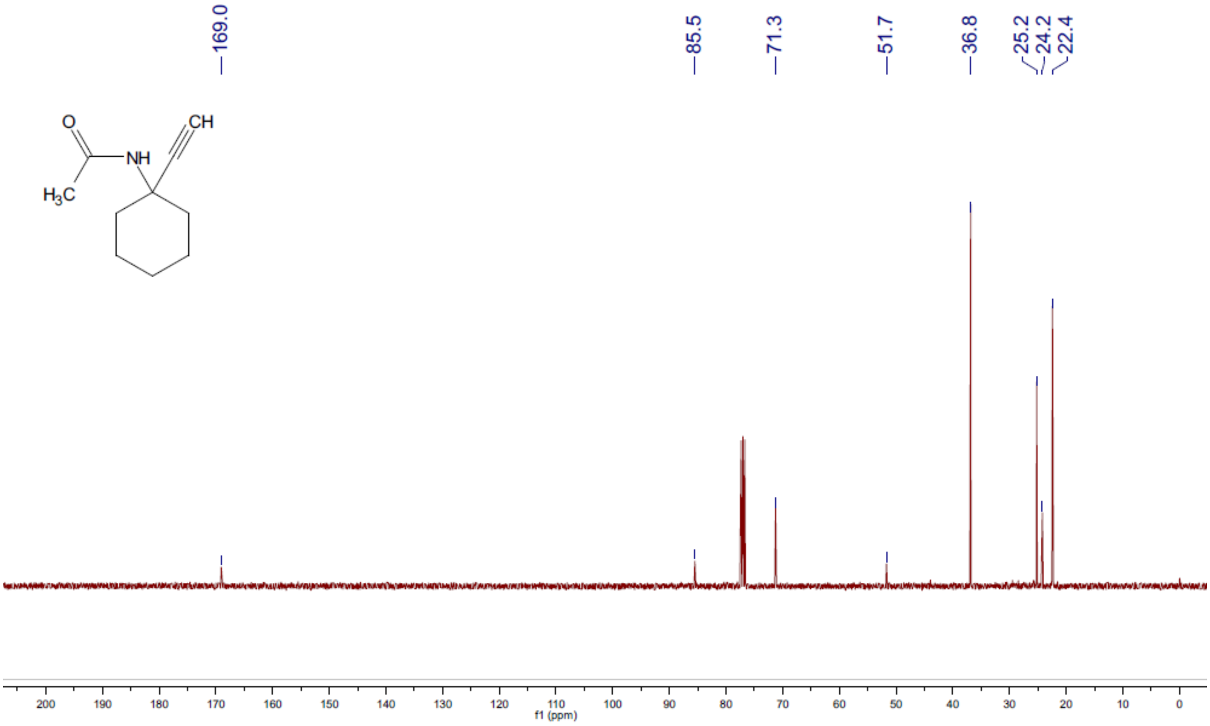

Supplementary Figure 198. ^{1}H NMR (400 MHz, $CDCl_3$) spectra of *N*-(1-ethynylcyclohexyl)acetamide (**1r**)

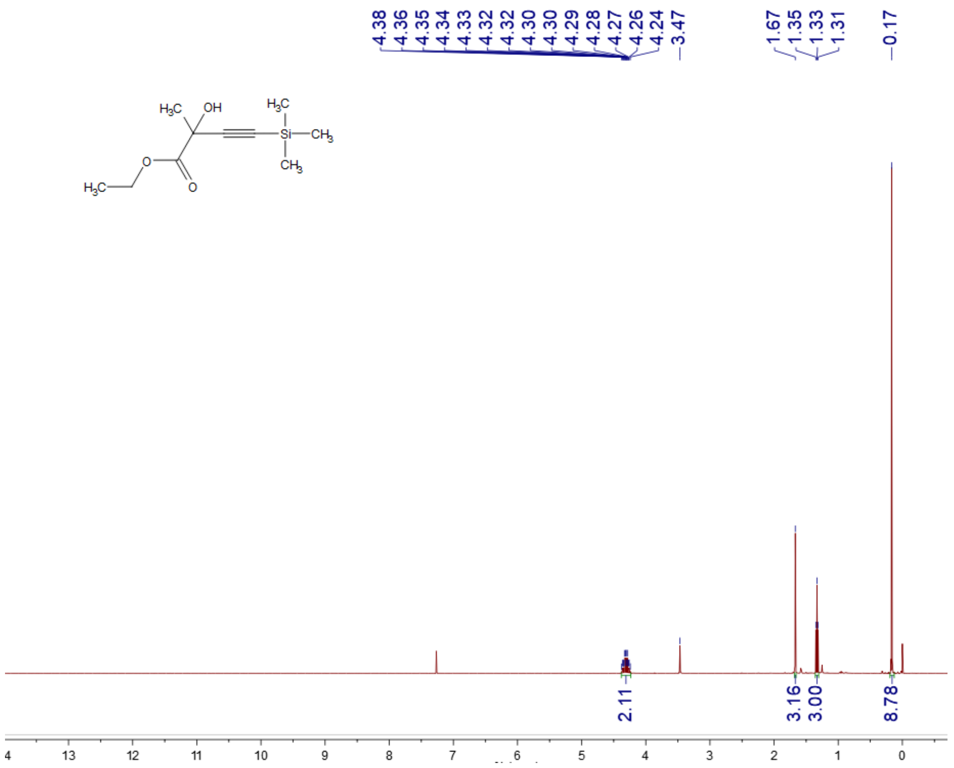

Supplementary Figure 199. ^{1}H NMR (400 MHz, $CDCl_3$) spectra of ethyl 2-hydroxy-2-methyl-4-(trimethylsilyl)but-3-ynoate (**1′s**)

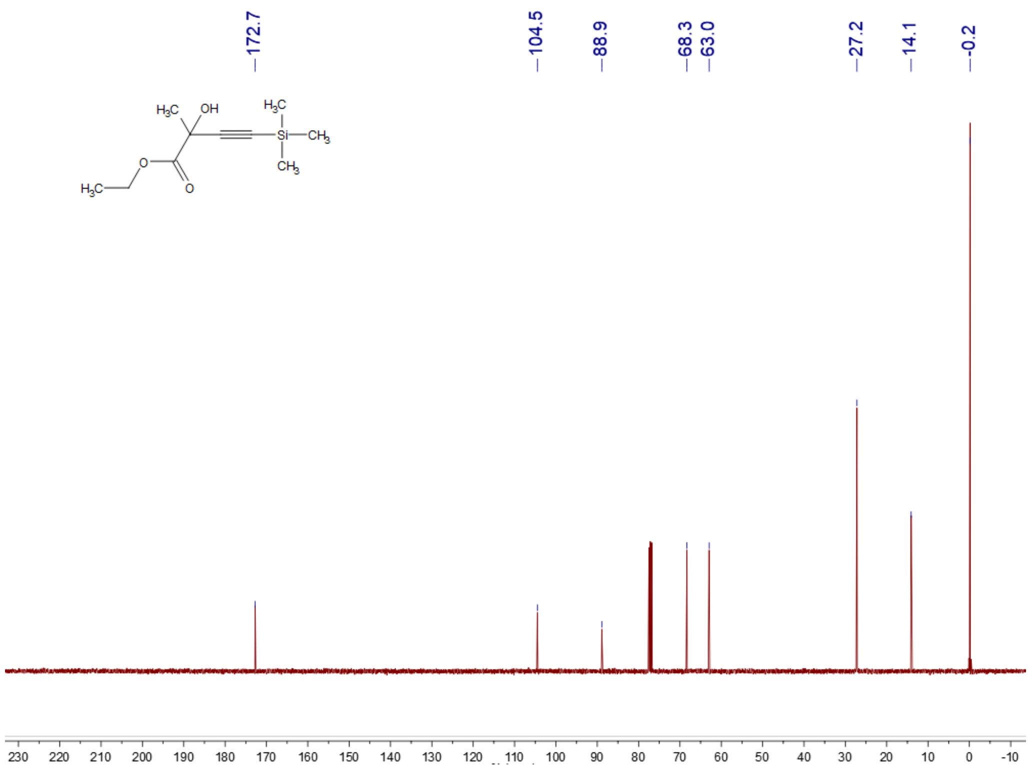

Supplementary Figure 200. ^{13}C NMR (100 MHz, $CDCl_3$) spectra of ethyl 2-hydroxy-2-methyl-4-(trimethylsilyl)but-3-ynoate (**1′s**)

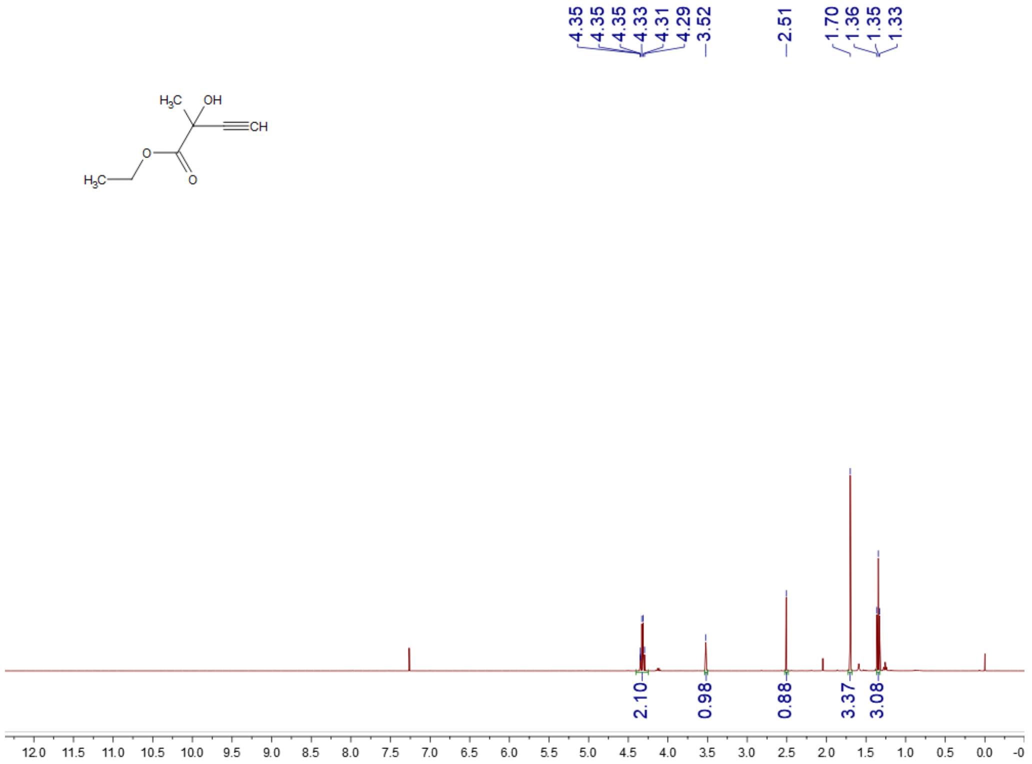

Supplementary Figure 201. ^{1}H NMR (400 MHz, $CDCl_3$) spectra of ethyl 2-hydroxy-2-methylbut-3-ynoate (**1″s**)

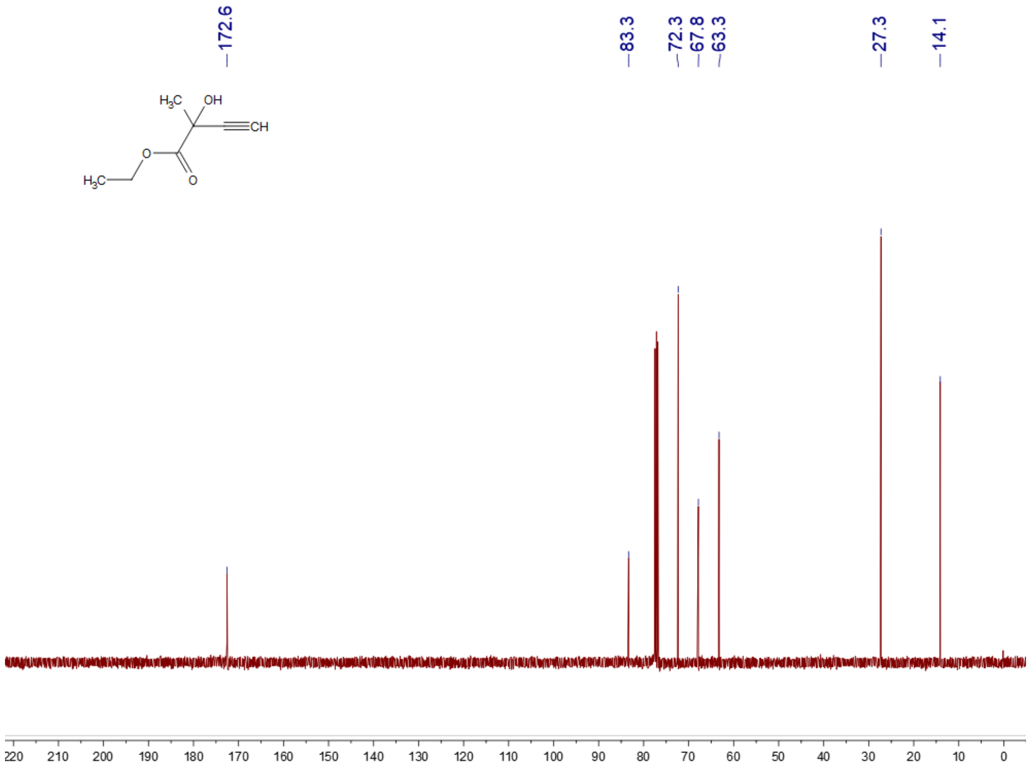

Supplementary Figure 202. ^{13}C NMR (100 MHz, $CDCl_3$) spectra of ethyl 2-hydroxy-2-methylbut-3-ynoate (**1″s**)

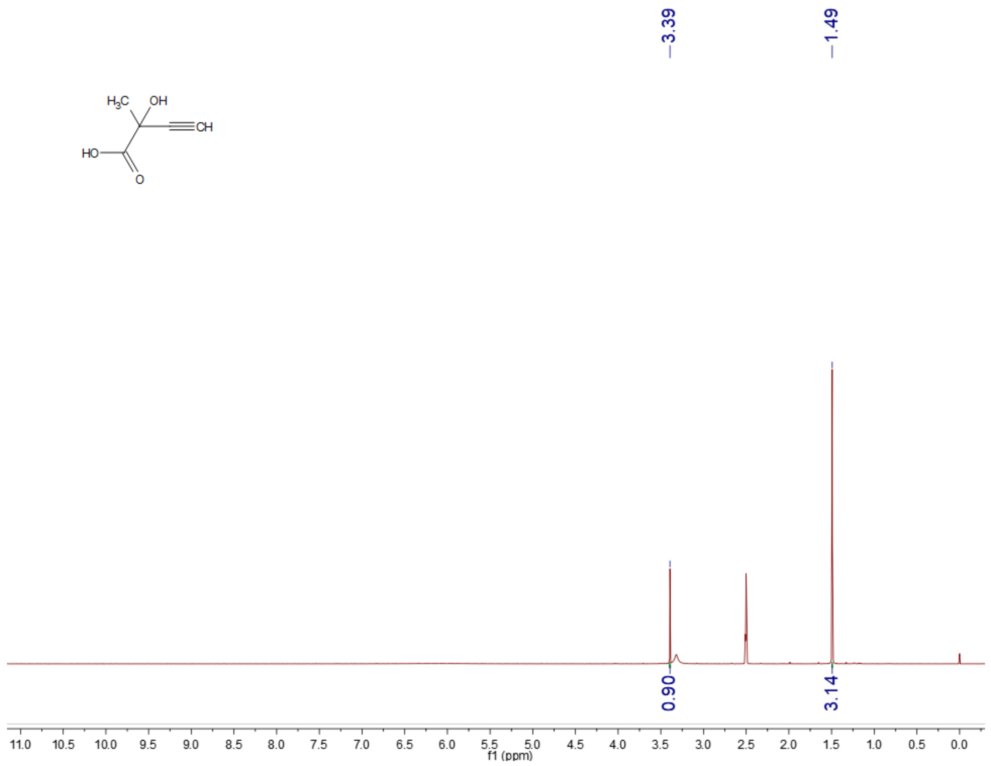

Supplementary Figure 203. ^{1}H NMR (400 MHz, DMSO-d$_6$) spectra of 2-hydroxy-2-methylbut-3-ynoic acid (**1s**)

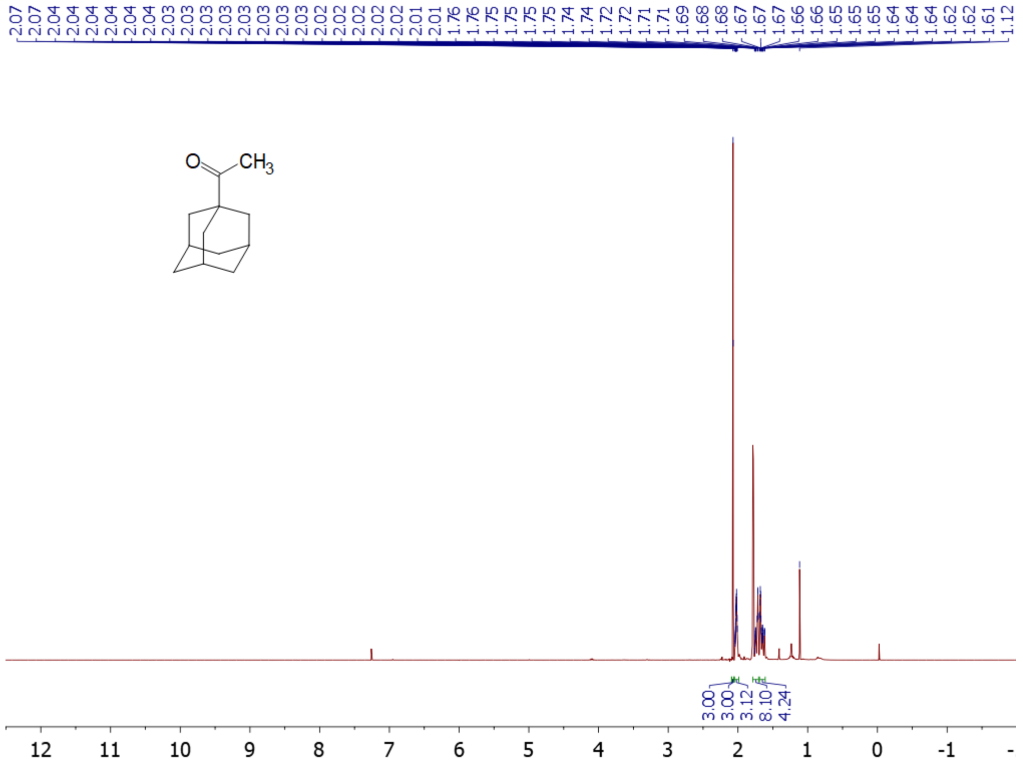

Supplementary Figure 204. ^{1}H NMR (400 MHz, CDCl$_3$) spectra of 1-((3*r*,5*r*,7*r*)-adamantan-1-yl)ethan-1-one (**1′t**)

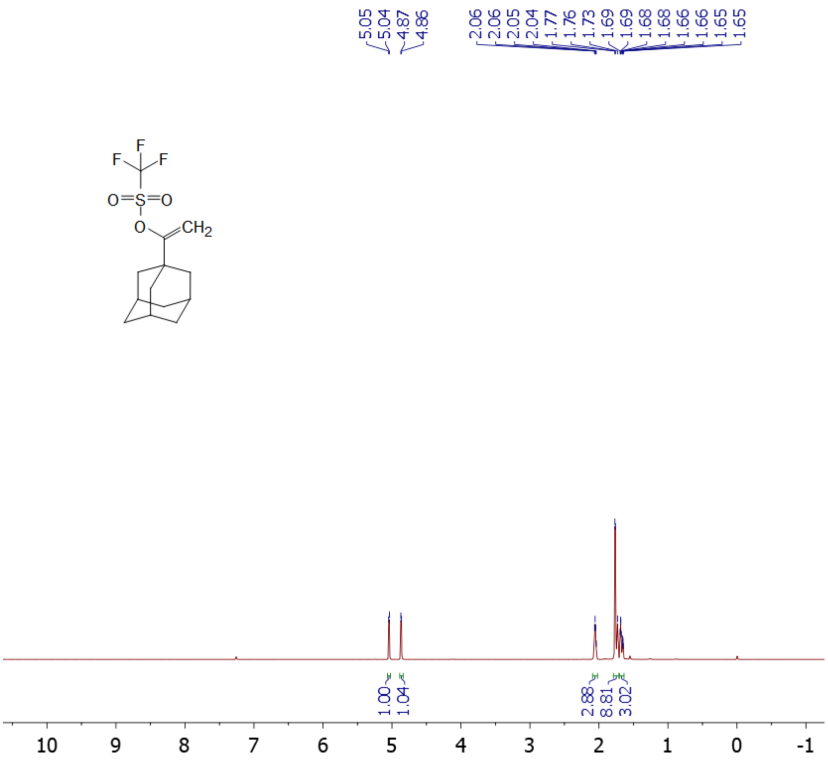

Supplementary Figure 205. ^{1}H NMR (400 MHz, $CDCl_3$) spectra of 1-((3*r*,5*r*,7*r*)-adamantan-1-yl)vinyl trifluoromethanesulfonate (**1′′t**)

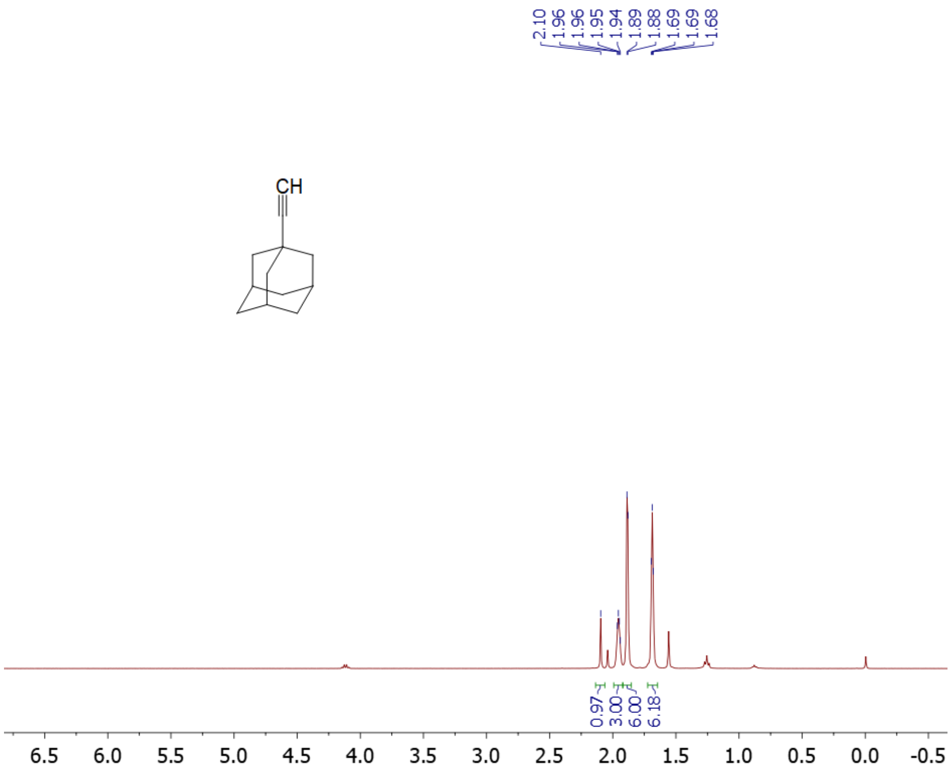

Supplementary Figure 206. ^{1}H NMR (400 MHz, $CDCl_3$) spectra of (3*r*,5*r*,7*r*)-1-ethynyladamantane (**1t**)

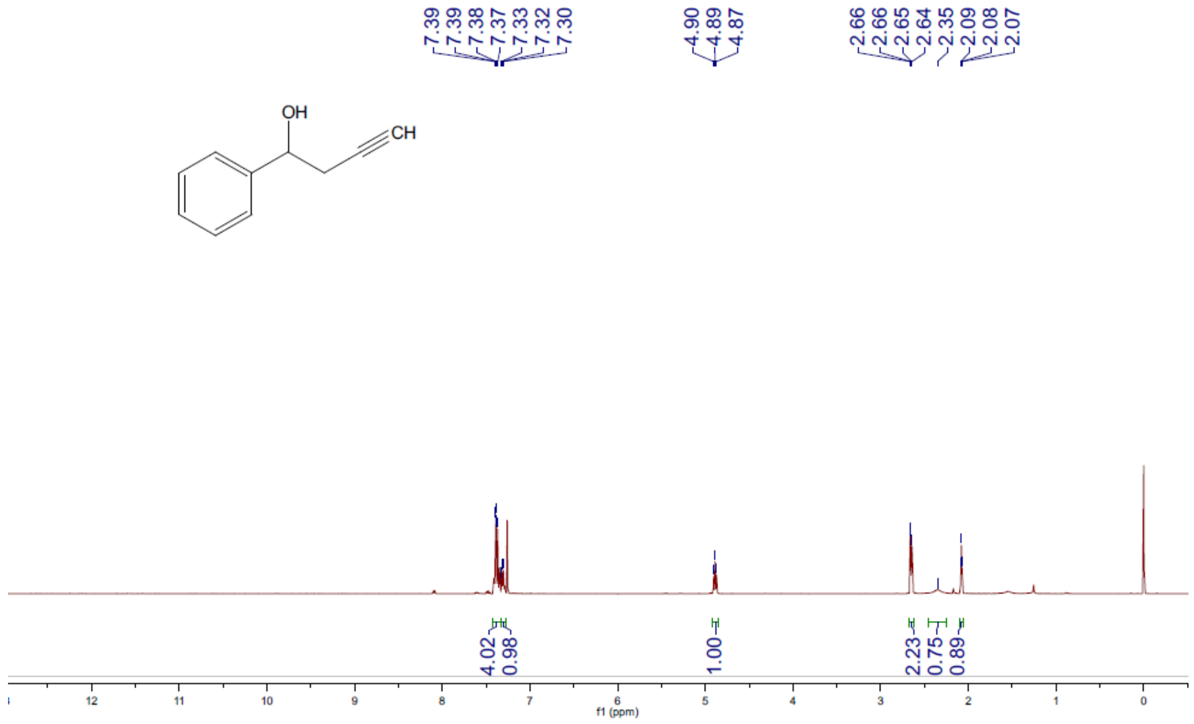

Supplementary Figure 207. ^{1}H NMR (400 MHz, $CDCl_3$) spectra of 1-phenylbut-3-yn-1-ol (**1u**)

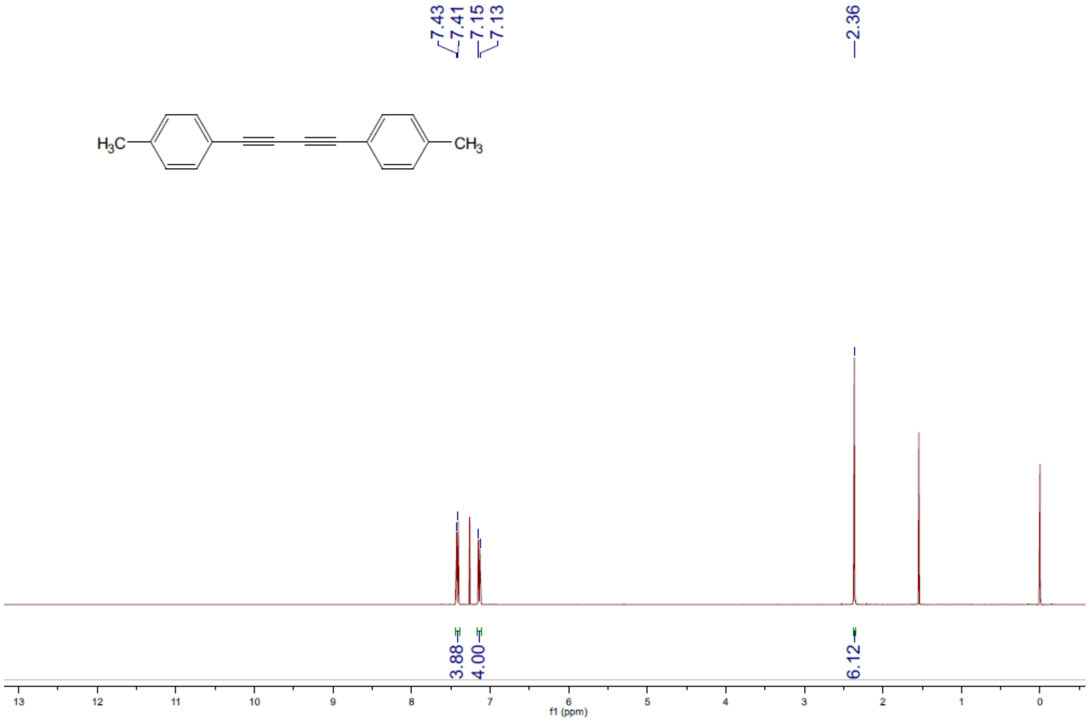

Supplementary Figure 208. ^{1}H NMR (400 MHz, $CDCl_3$) spectra of 1,4-di-*p*-tolylbuta-1,3-diyne (**1v**)

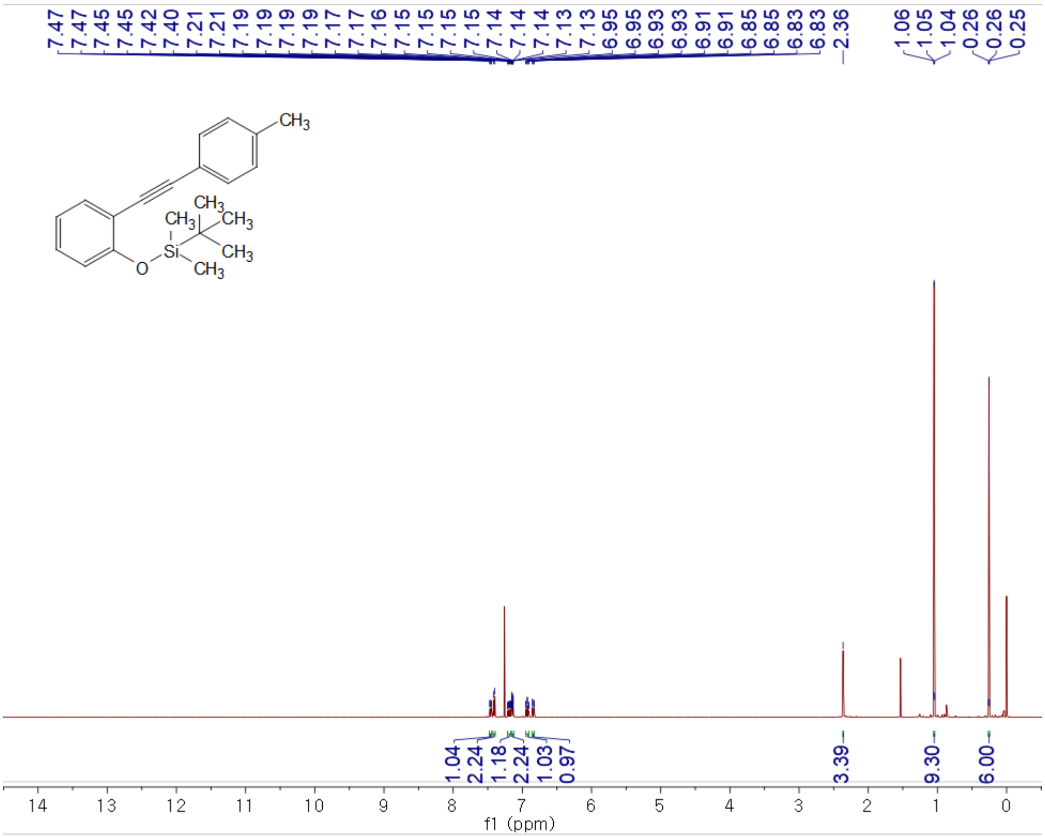

Supplementary Figure 209. ^{1}H NMR (400 MHz, $CDCl_3$) spectra of tert-butyldimethyl(2-(p-tolylethynyl)phenoxy)silane (**1w**)

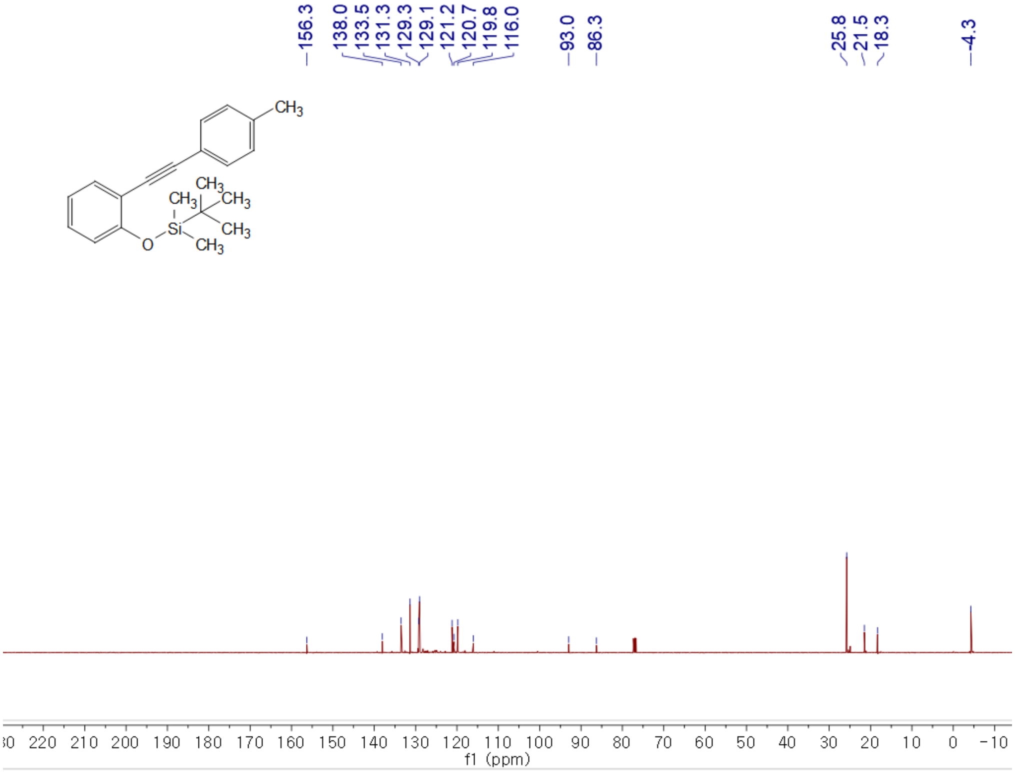

Supplementary Figure 210. ^{13}C NMR (100 MHz, $CDCl_3$) spectra of tert-butyldimethyl(2-(p-tolylethynyl)phenoxy)silane (**1w**)

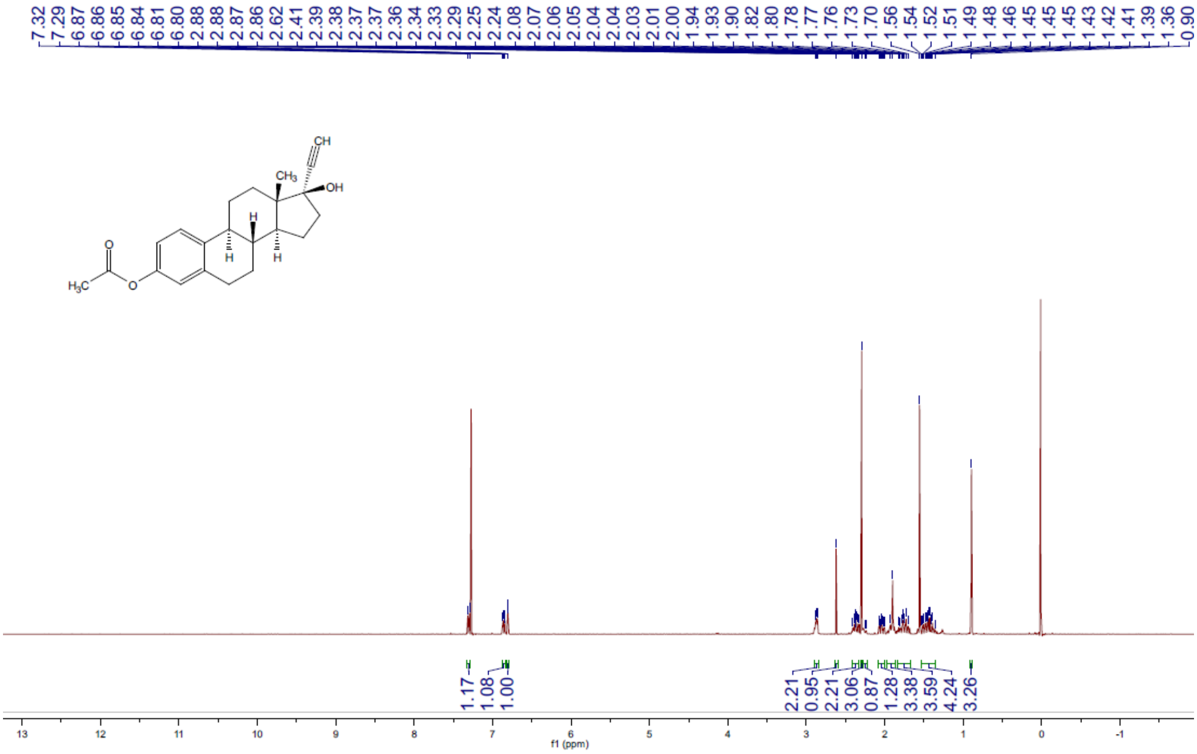

Supplementary Figure 211. ^{1}H NMR (400 MHz, $CDCl_3$) spectra of 17α-ethynylestradiol 3-acetate (**1y**)

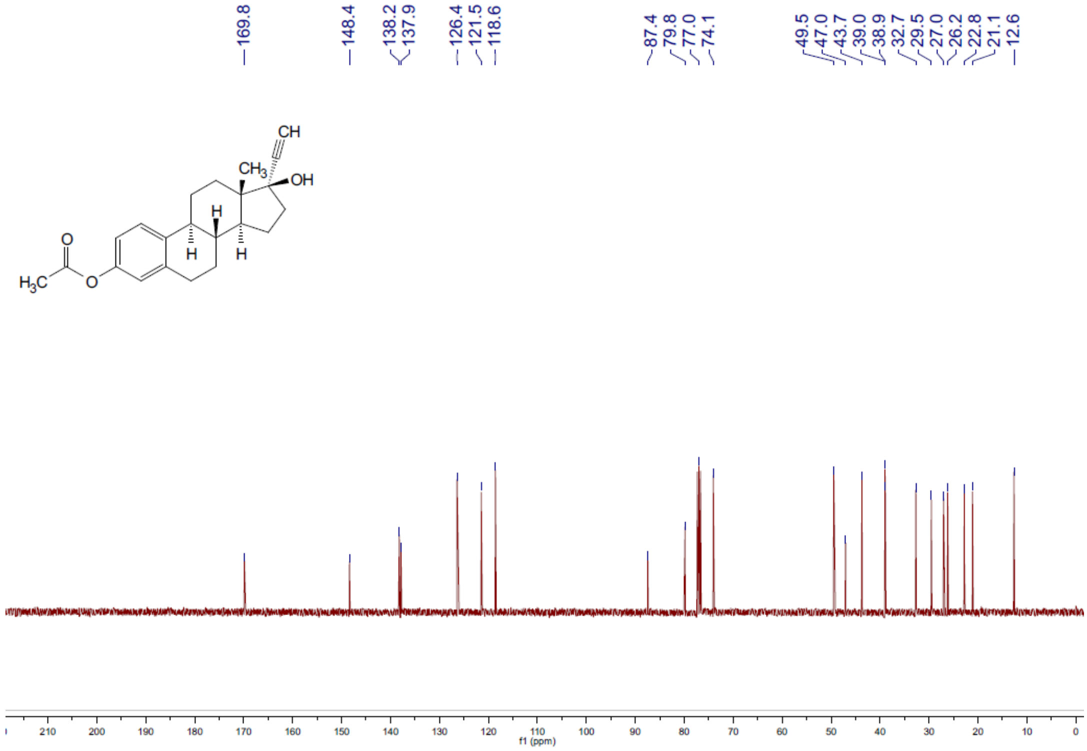

Supplementary Figure 212. ^{13}C NMR (100 MHz, $CDCl_3$) of 17α-ethynylestradiol 3-acetate (**1y**)

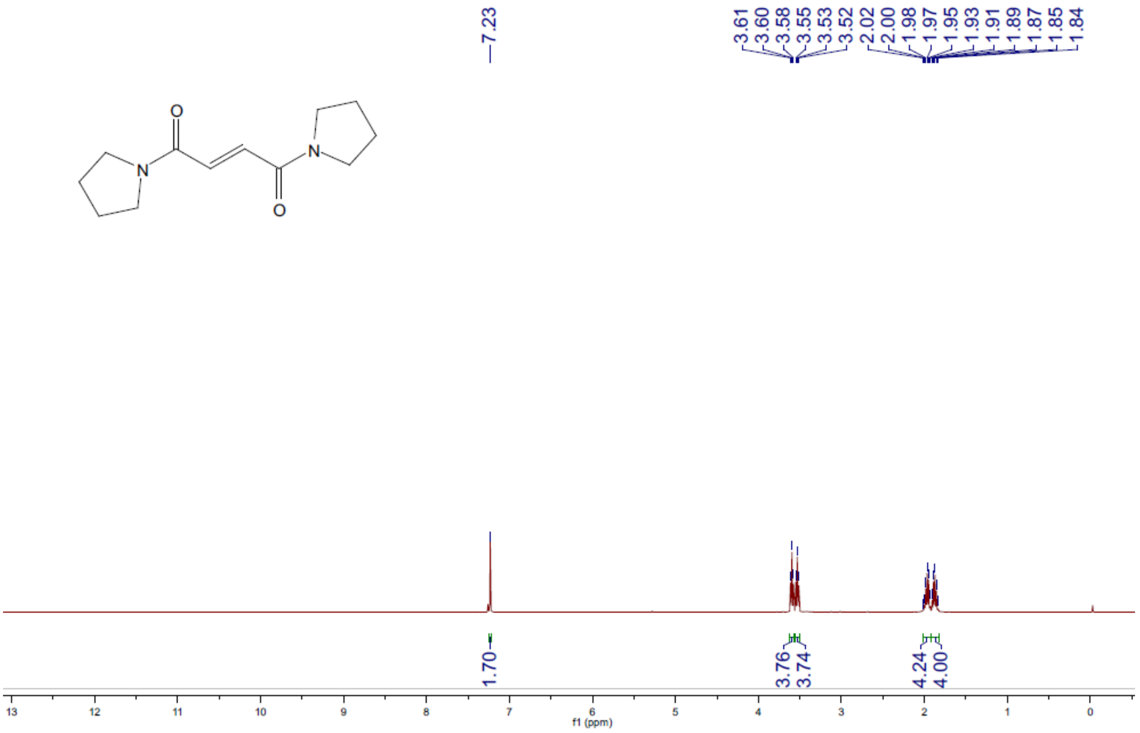

Supplementary Figure 213. ^{1}H NMR (400 MHz, $CDCl_3$) spectra of *(E)*-1,4-di(pyrrolidin-1-yl)but-2-ene-1,4-dione (**2c**)

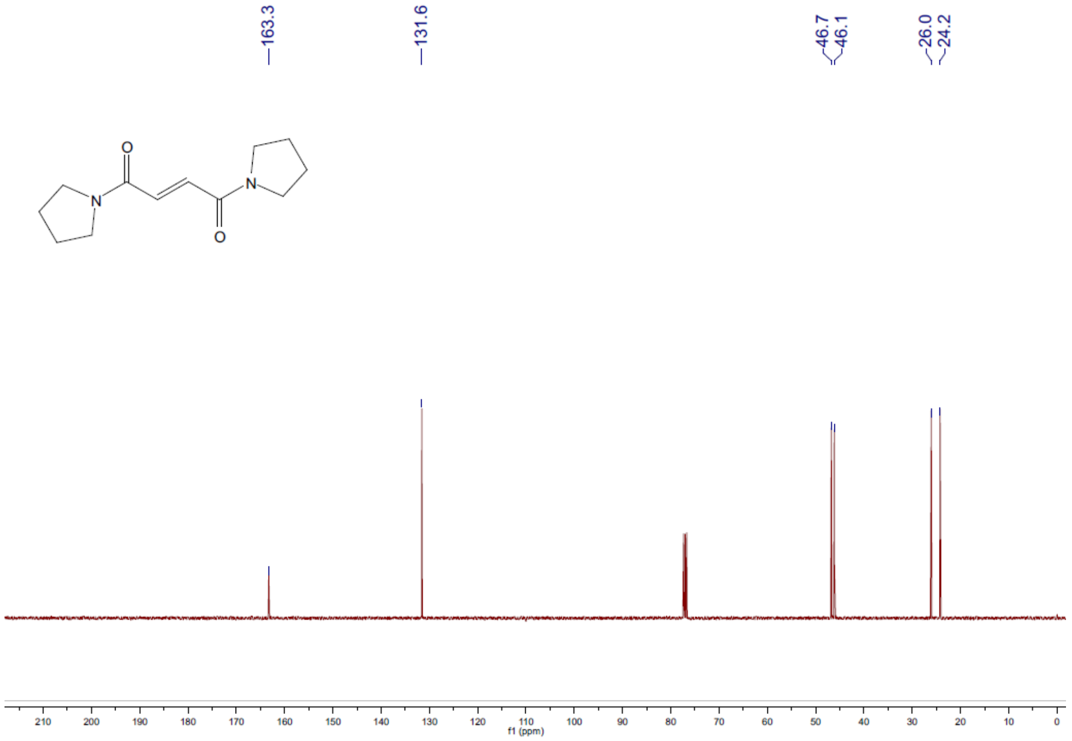

Supplementary Figure 214. ^{1}H NMR (400 MHz, $CDCl_3$) spectra of *(E)*-1,4-di(pyrrolidin-1-yl)but-2-

ene-1,4-dione (**2c**)

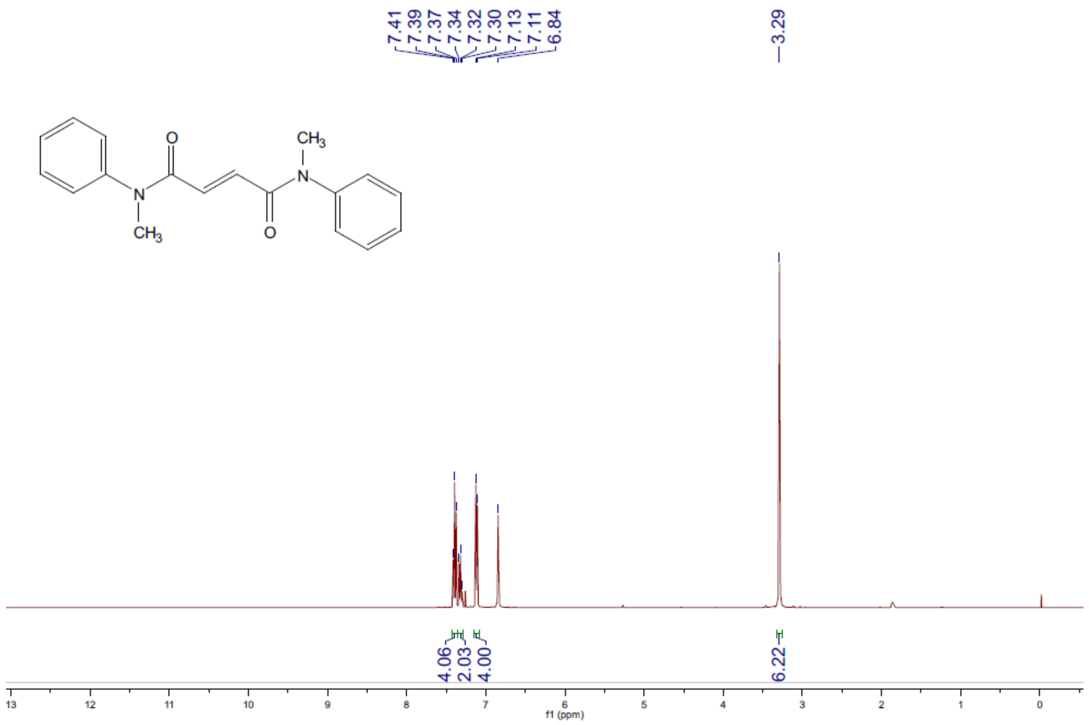

Supplementary Figure 215. ^{1}H NMR (400 MHz, $CDCl_3$) spectra of *N*[1],*N*[4]-dimethyl-*N*[1],*N*[4]-diphenylfumaramide (**2d**)

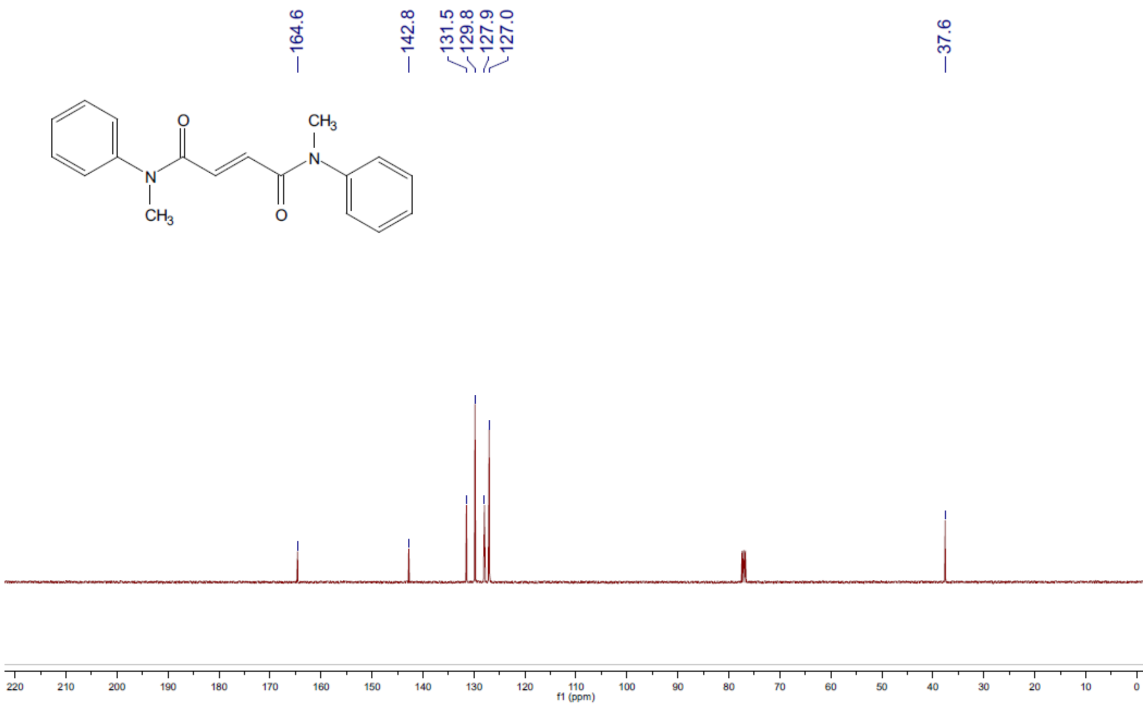

Supplementary Figure 216. ^{1}H NMR (400 MHz, $CDCl_3$) spectra of *N*[1],*N*[4]-dimethyl-*N*[1],*N*[4]-diphenylfumaramide (**2d**)

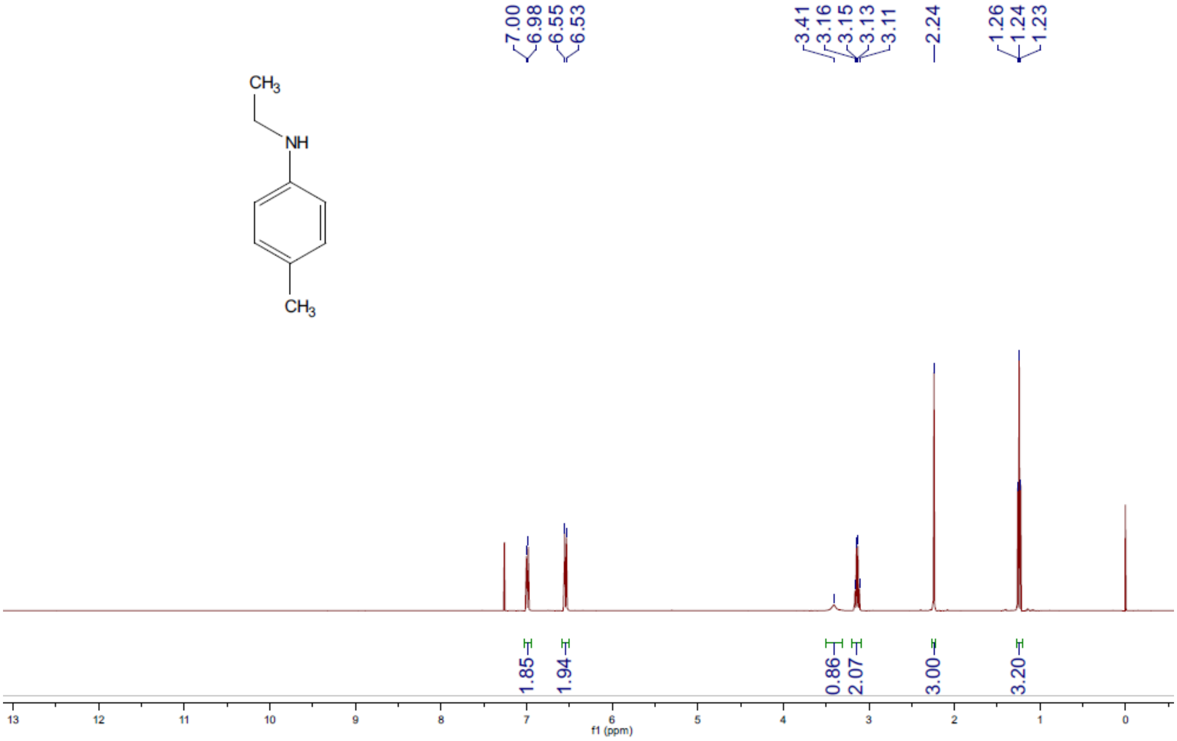

Supplementary Figure 217. ^{1}H NMR (400 MHz, $CDCl_3$) spectra of *N*-ethyl-4-methylaniline (**2′g**)

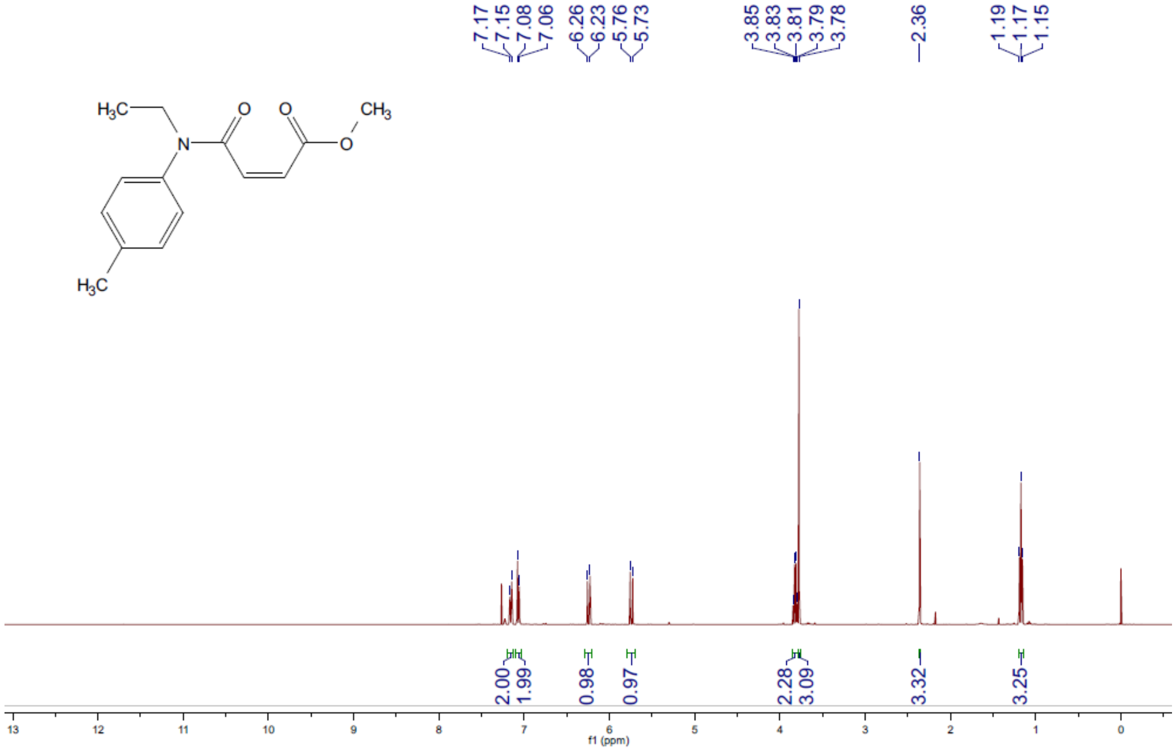

Supplementary Figure 218. ^{1}H NMR (400 MHz, $CDCl_3$) spectra of methyl (*Z*)-4-(ethyl(*p*-tolyl)amino)-4-oxobut-2-enoate (**2g**)

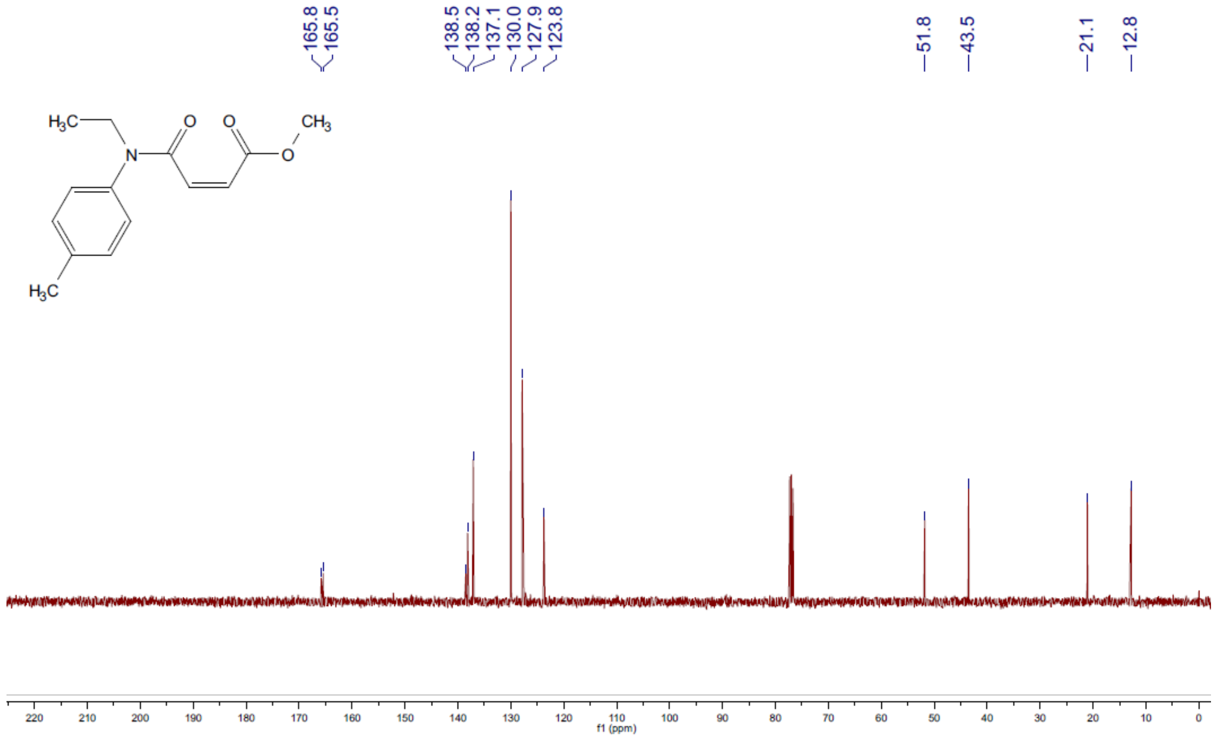

Supplementary Figure 219. ^{1}H NMR (400 MHz, $CDCl_3$) spectra of methyl (*Z*)-4-(ethyl(*p*-tolyl)amino)-4-oxobut-2-enoate (**2g**)

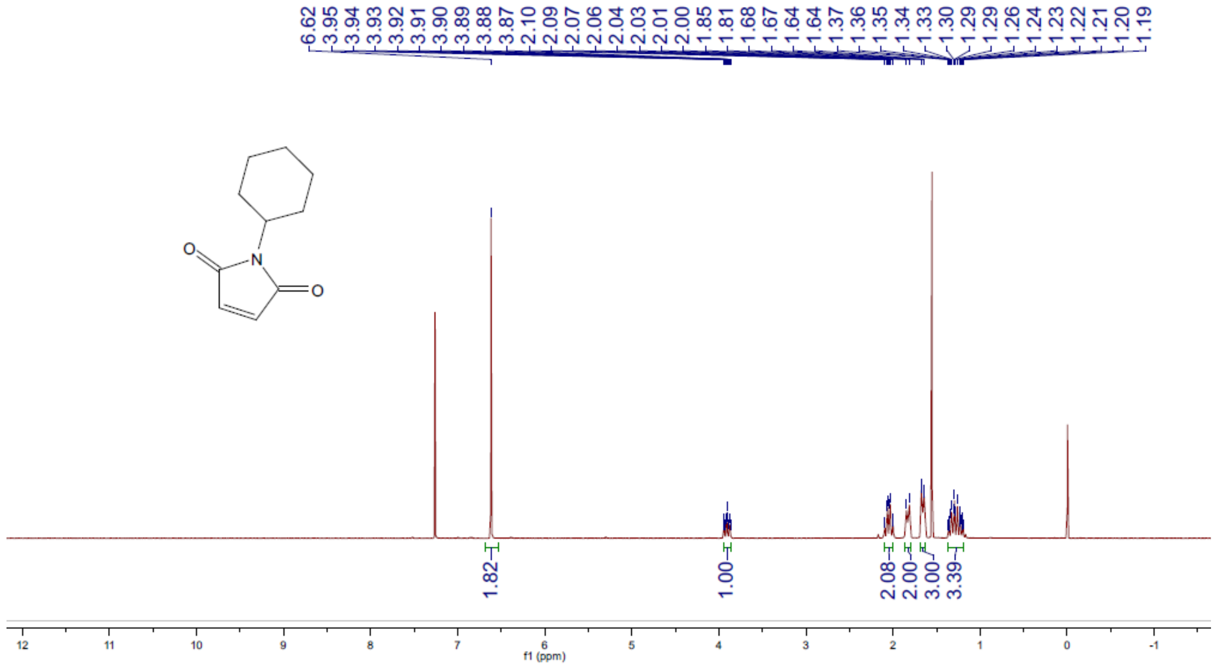

Supplementary Figure 220. ^{1}H NMR (400 MHz, $CDCl_3$) spectra of 1-cyclohexyl-1*H*-pyrrole-2,5-dione (**2i**)

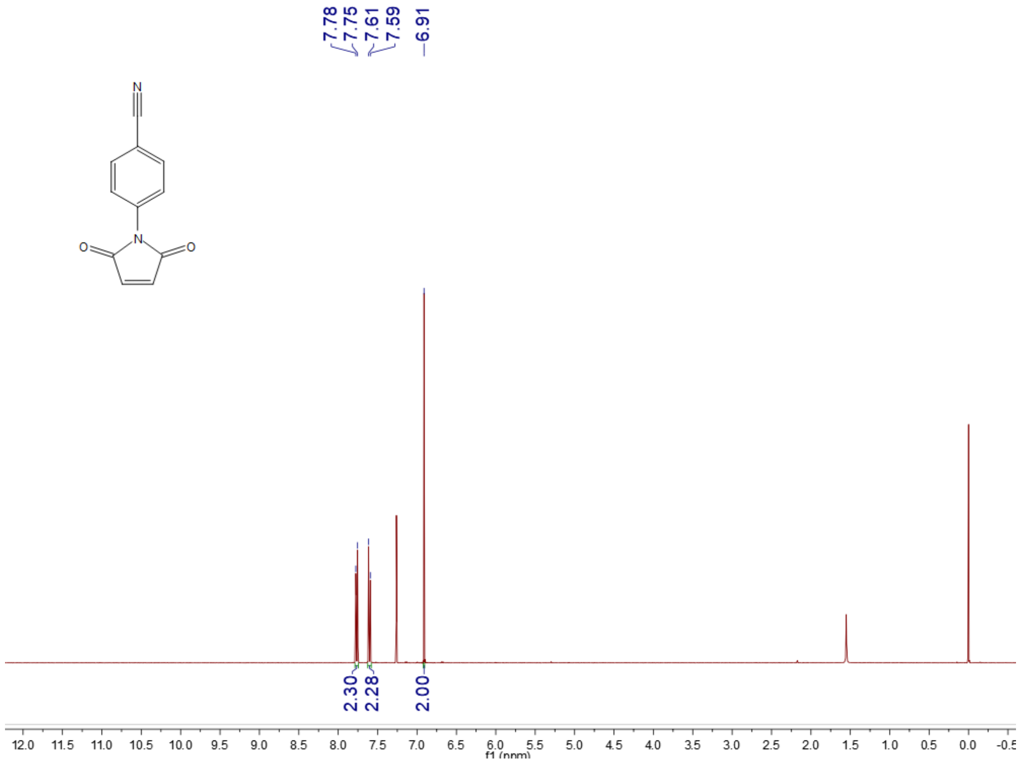

Supplementary Figure 221. ^{1}H NMR (400 MHz, $CDCl_3$) spectra of 4-(2,5-dioxo-2,5-dihydro-1*H*-pyrrol-1-yl)benzonitrile (**2k**)

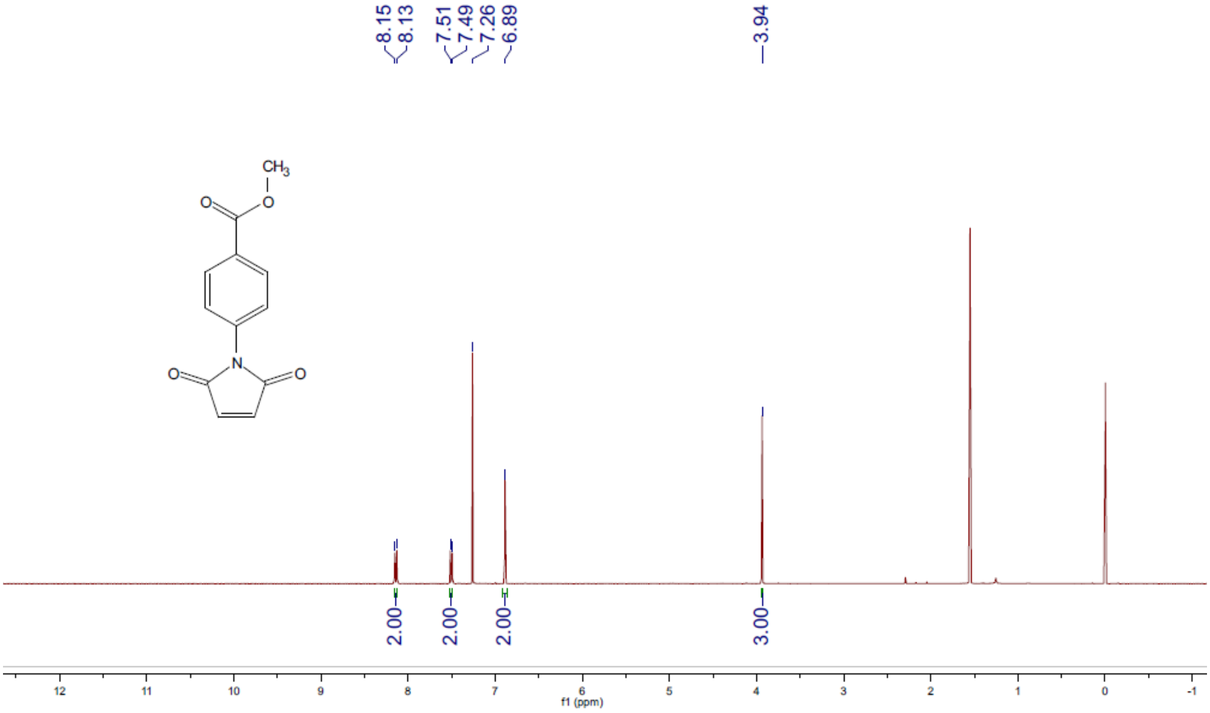

Supplementary Figure 222. ^{1}H NMR (400 MHz, $CDCl_3$) spectra of methyl 4-(2,5-dioxo-2,5-dihydro-1*H*-pyrrol-1-yl)benzoate (**2l**)

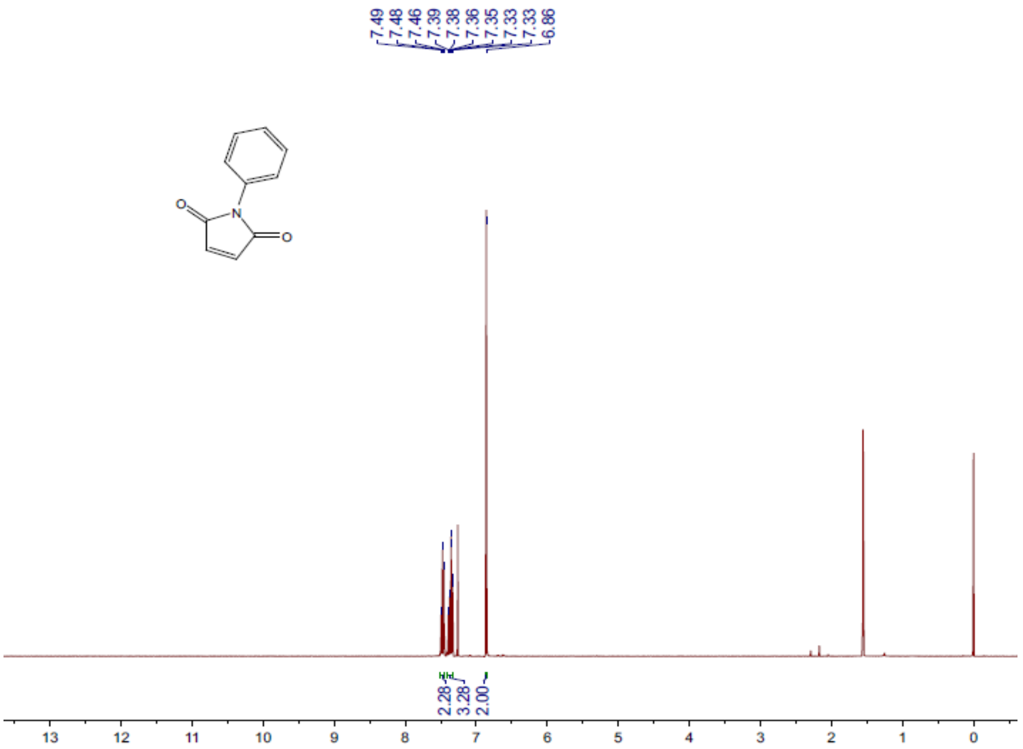

Supplementary Figure 223. ^{1}H NMR (400 MHz, $CDCl_3$) spectra of 1-phenyl-1*H*-pyrrole-2,5-dione (**2m**)

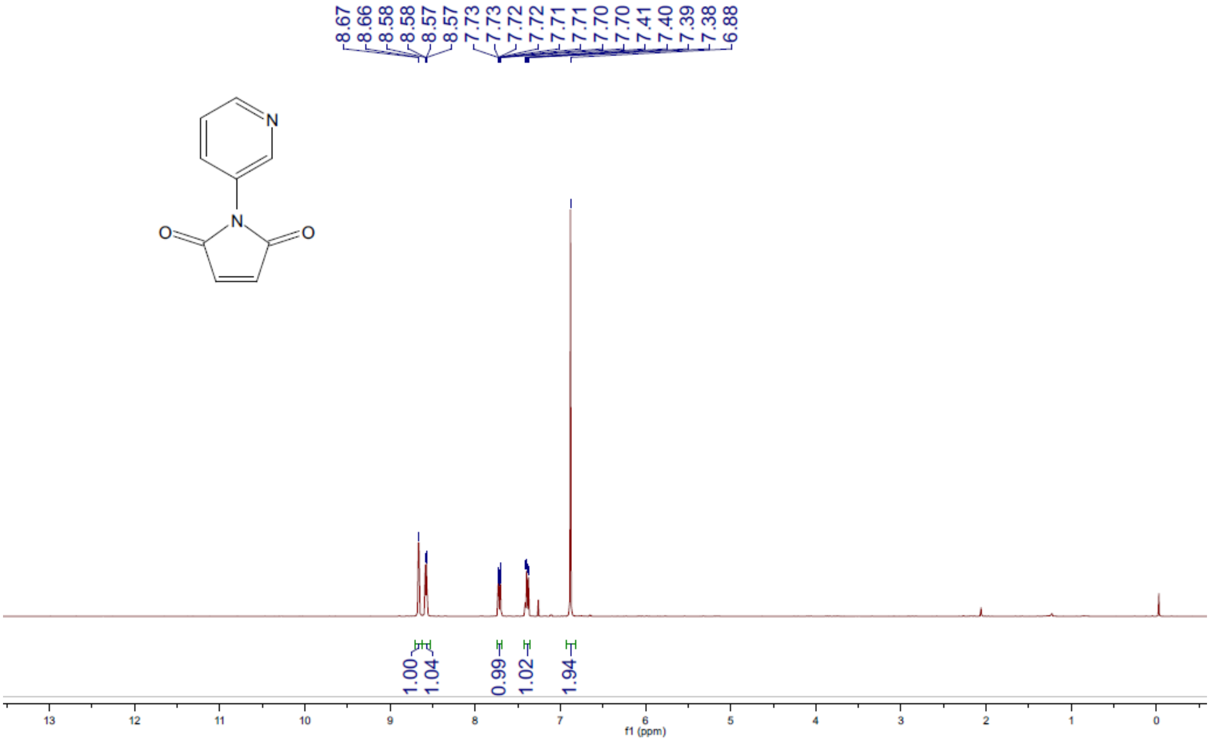

Supplementary Figure 224. ^{1}H NMR (400 MHz, $CDCl_3$) spectra of 1-(pyridin-3-yl)-1*H*-pyrrole-2,5-dione (**2n**)

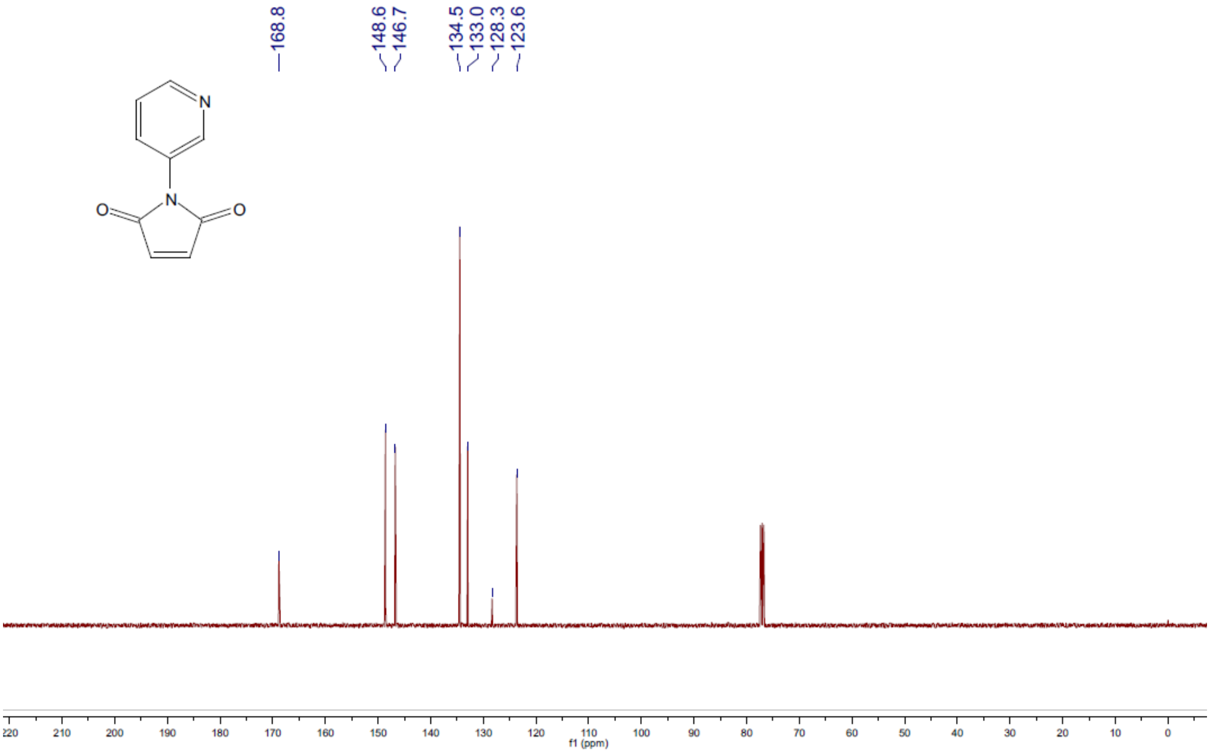

Supplementary Figure 225. ^{1}H NMR (400 MHz, $CDCl_3$) spectra of 1-(pyridin-3-yl)-1*H*-pyrrole-2,5-dione (**2n**)

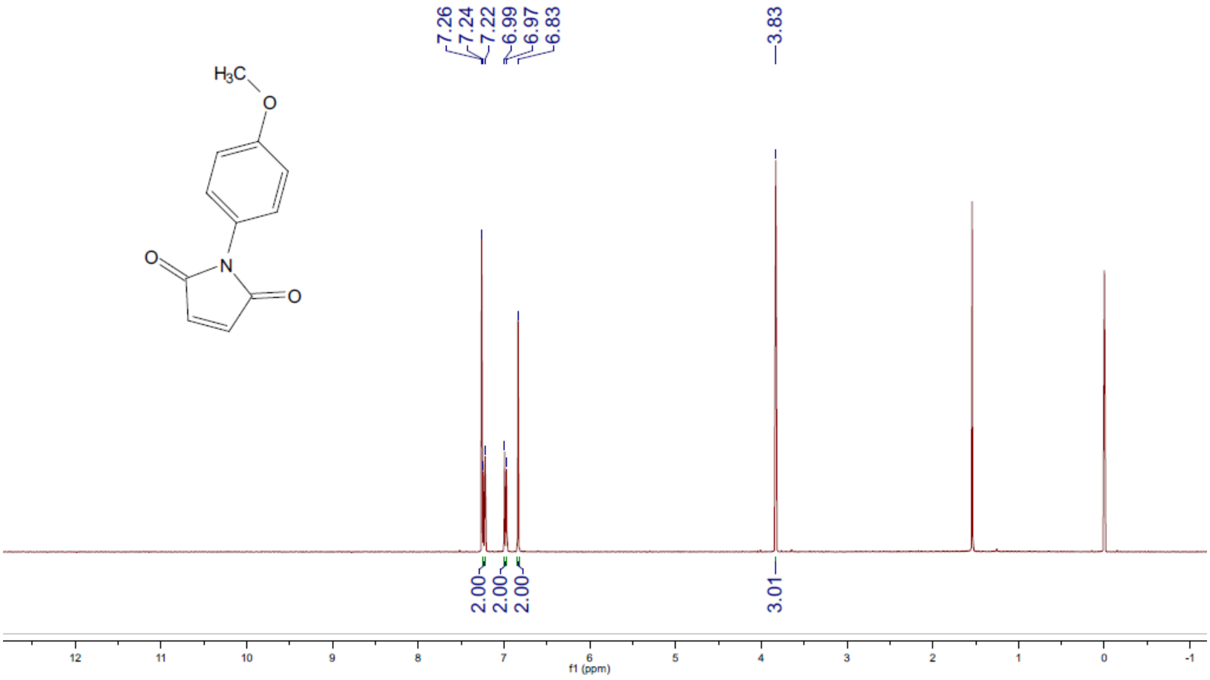

Supplementary Figure 226. ^{1}H NMR (400 MHz, $CDCl_3$) spectra of 1-(4-methoxyphenyl)-1*H*-pyrrole-2,5-dione (**2z**)

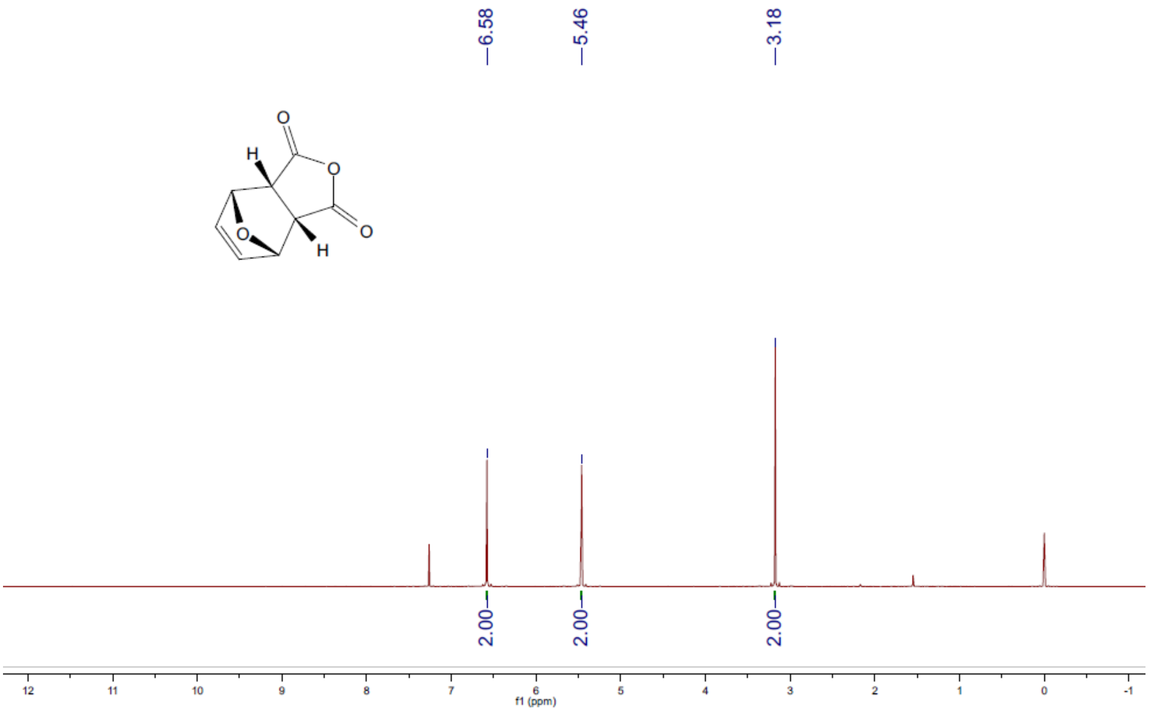

Supplementary Figure 227. ^{1}H NMR (400 MHz, $CDCl_3$) spectra of (3a*R*,4*R*,7*S*,7a*S*)-3a,4,7,7a-tetrahydro-4,7-epoxyisobenzofuran-1,3-dione (**2′j**)

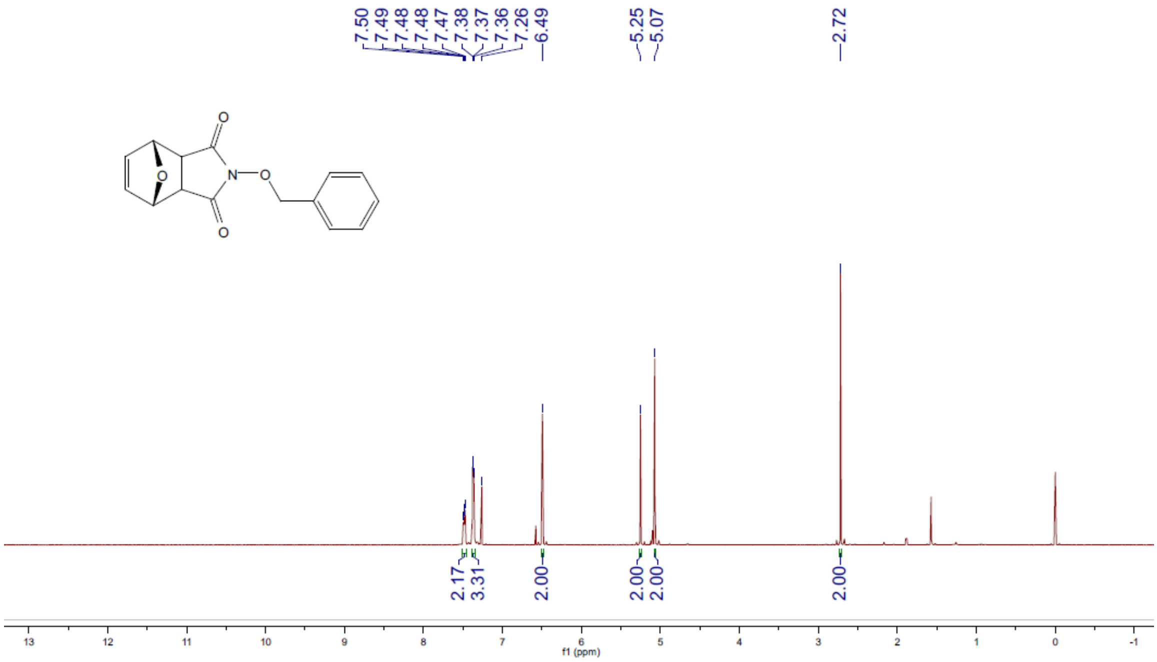

Supplementary Figure 228. ^{1}H NMR (400 MHz, $CDCl_3$) spectra of (3a*R*,4*R*,7*S*,7a*S*)-2-(benzyloxy)-3a,4,7,7a-tetrahydro-1*H*-4,7-epoxyisoindole-1,3(2*H*)-dione (**2″j**)

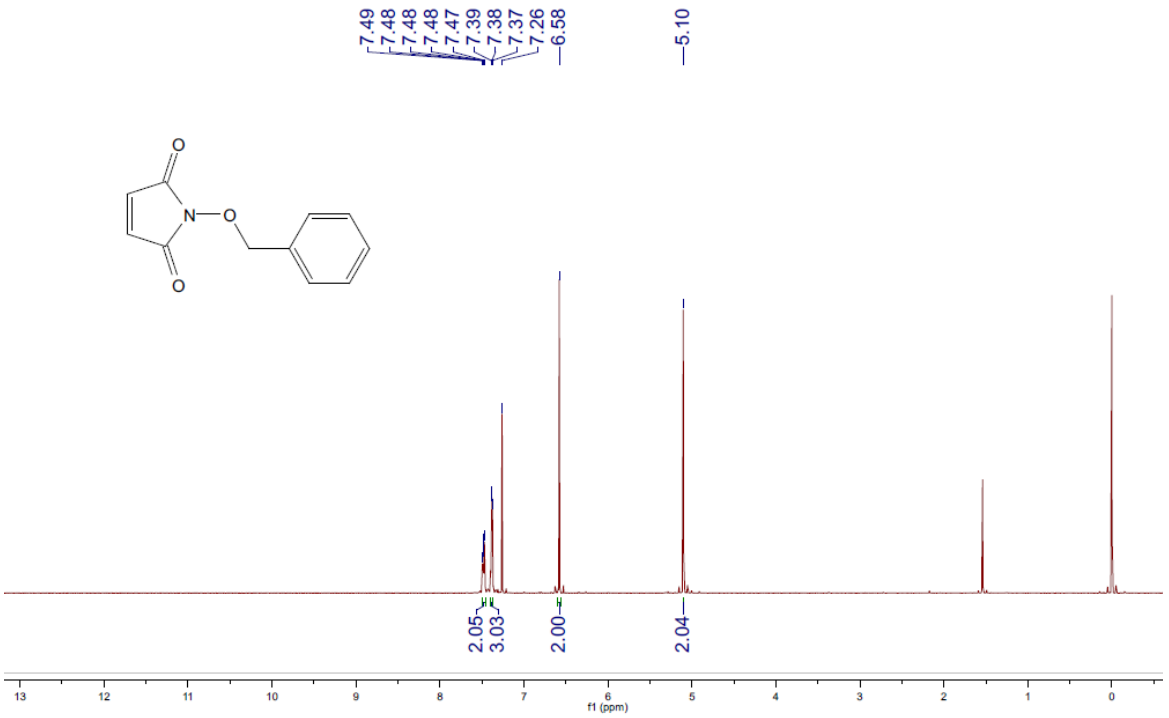

Supplementary Figure 229. ^{1}H NMR (400 MHz, $CDCl_3$) spectra of 1-(benzyloxy)-1*H*-pyrrole-2,5-dione (**2j**)

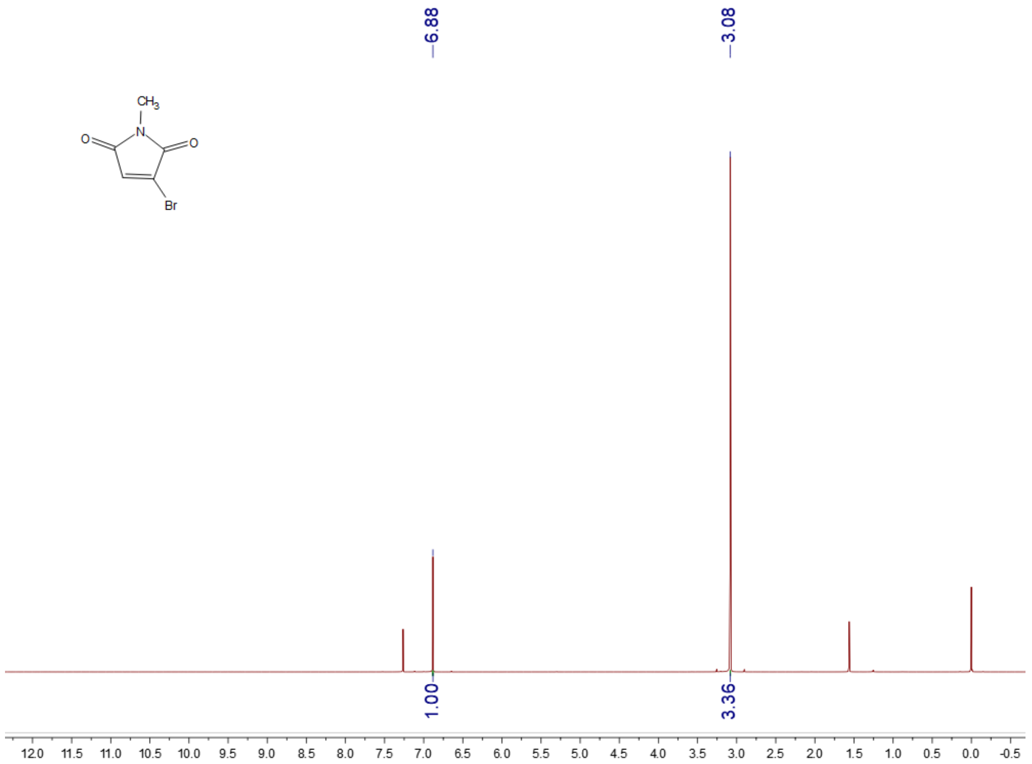

Supplementary Figure 230. ^{1}H NMR (400 MHz, $CDCl_3$) spectra of 3-bromo-1-methyl-1*H*-pyrrole-2,5-dione (**2o**)

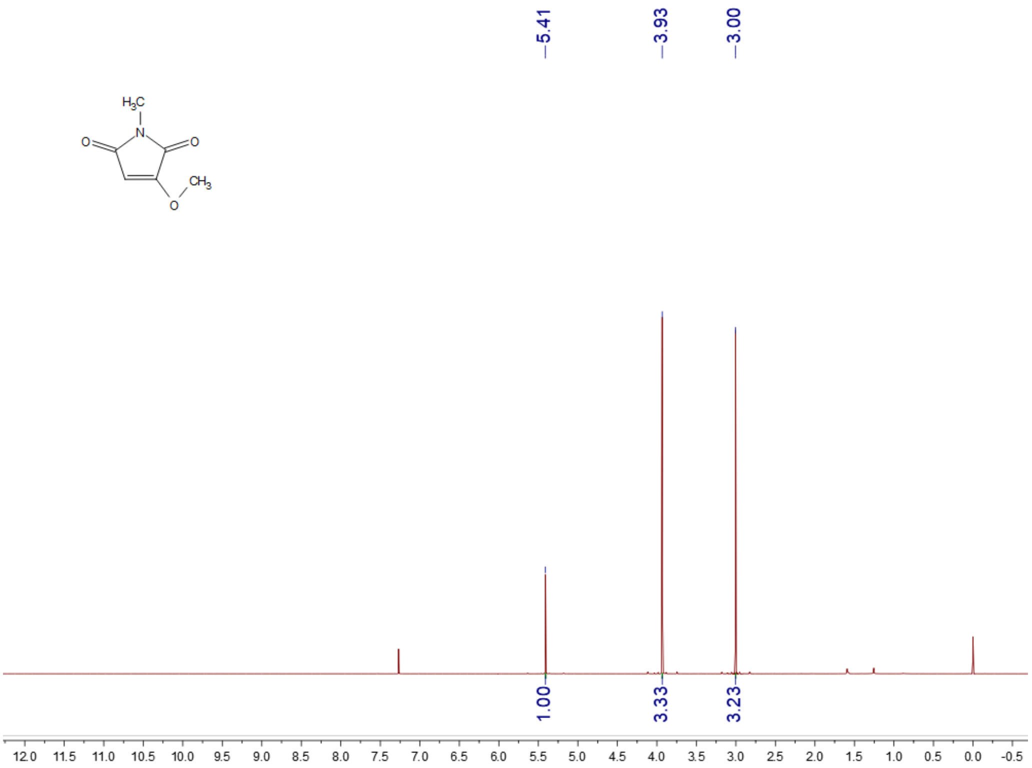

Supplementary Figure 231. ¹H NMR (400 MHz, $CDCl_3$) spectra of 3-methoxy-1-methyl-1*H*-pyrrole-2,5-dione (**2p**)

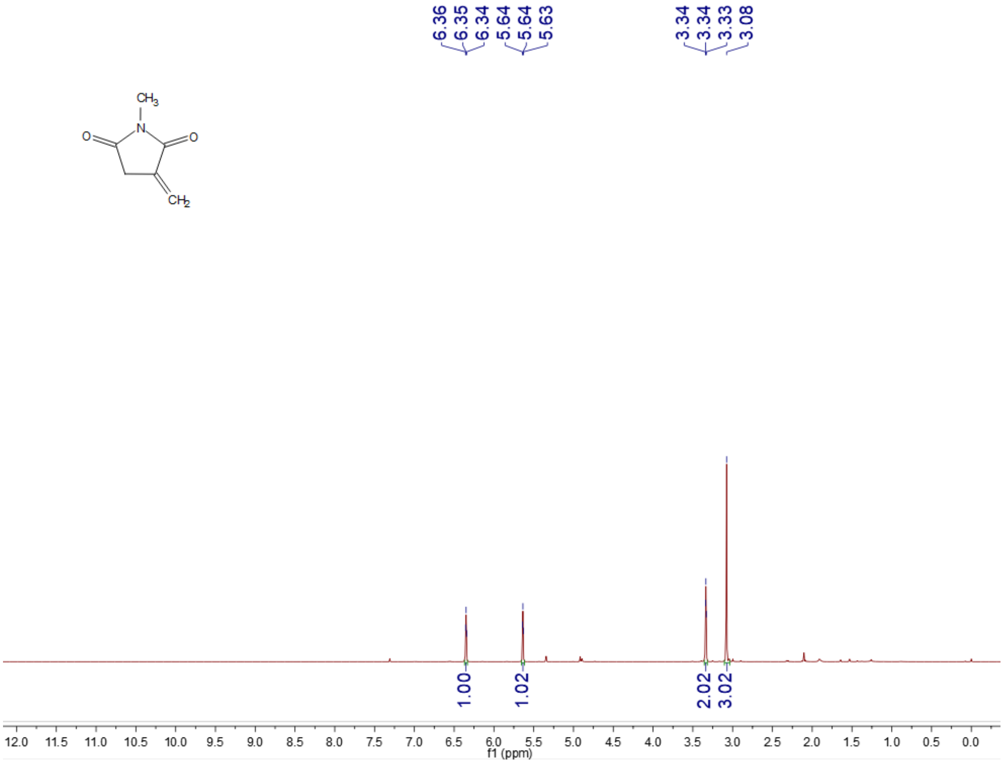

Supplementary Figure 232. ¹H NMR (400 MHz, $CDCl_3$) spectra of 1-methyl-3-methylenepyrrolidine-2,5-dione (**2′q**)

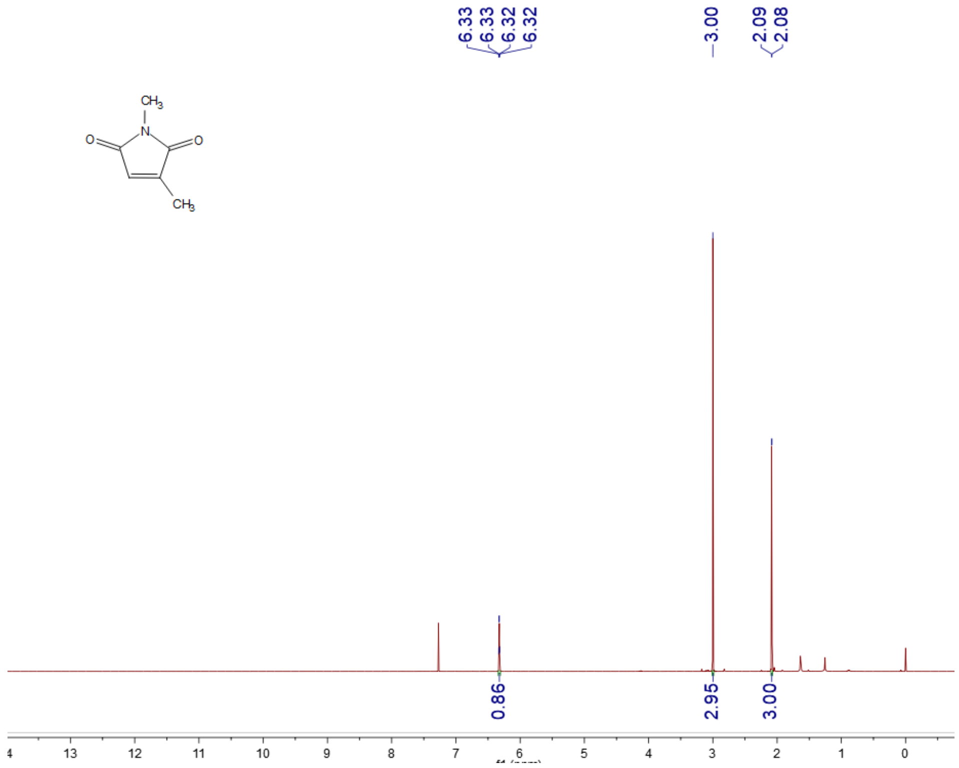

Supplementary Figure 233. ^{1}H NMR (400 MHz, $CDCl_3$) spectra of 1,3-dimethyl-1*H*-pyrrole-2,5-dione (**2q**)

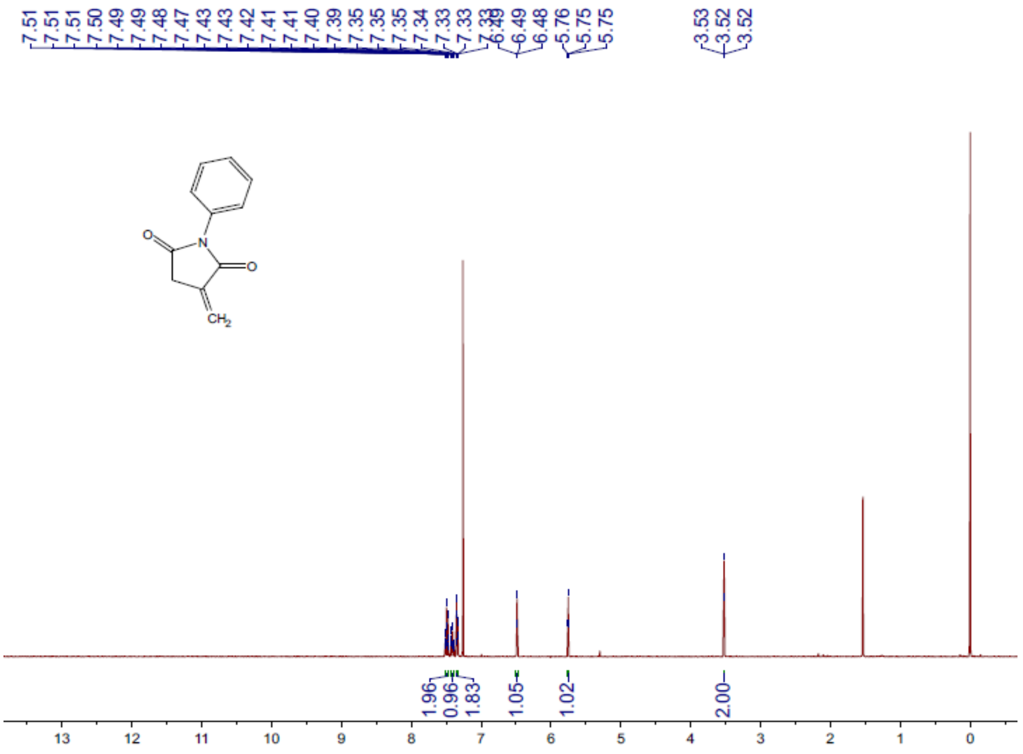

Supplementary Figure 234. ^{1}H NMR (400 MHz, $CDCl_3$) spectra of 3-methylene-1-phenylpyrrolidine-2,5-dione (**2′r**)

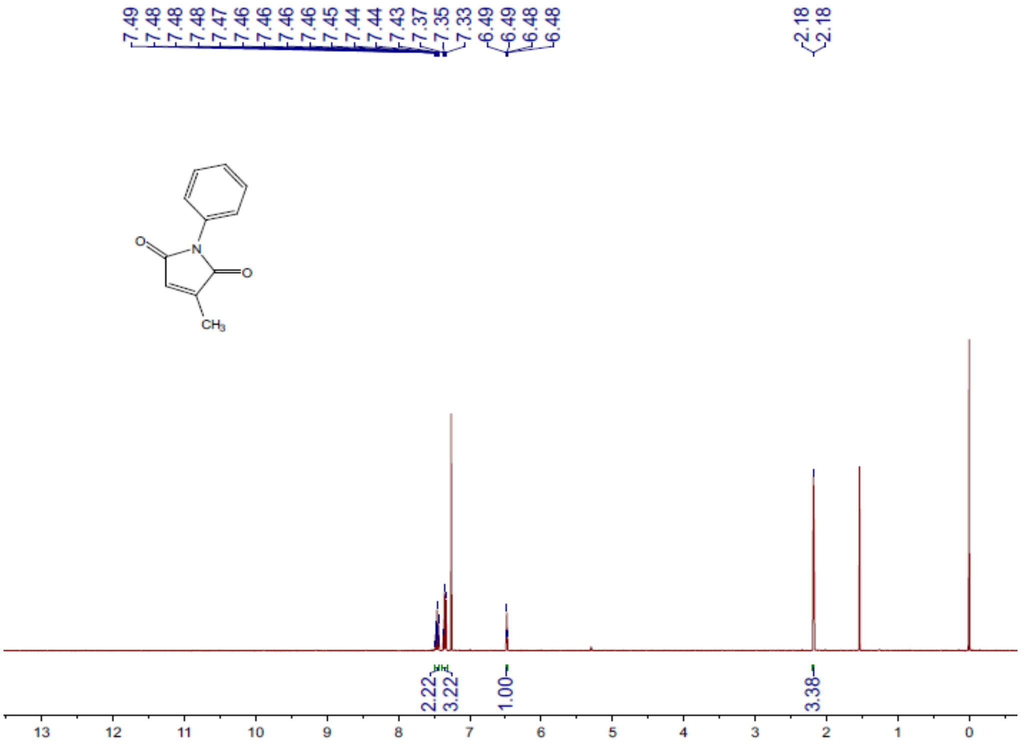

Supplementary Figure 235. ^{1}H NMR (400 MHz, $CDCl_3$) spectra of 3-methyl-1-phenyl-1*H*-pyrrole-2,5-dione (**2r**)

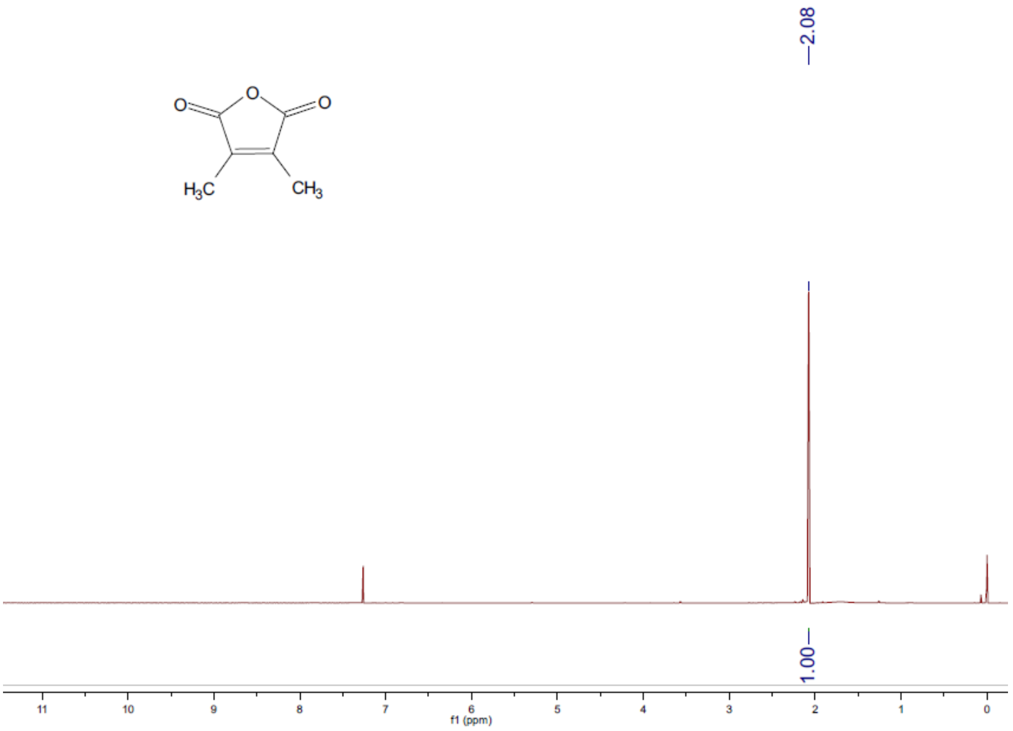

Supplementary Figure 236. ^{1}H NMR (400 MHz, $CDCl_3$) spectra of 3,4-dimethylfuran-2,5-dione (**2′s**)

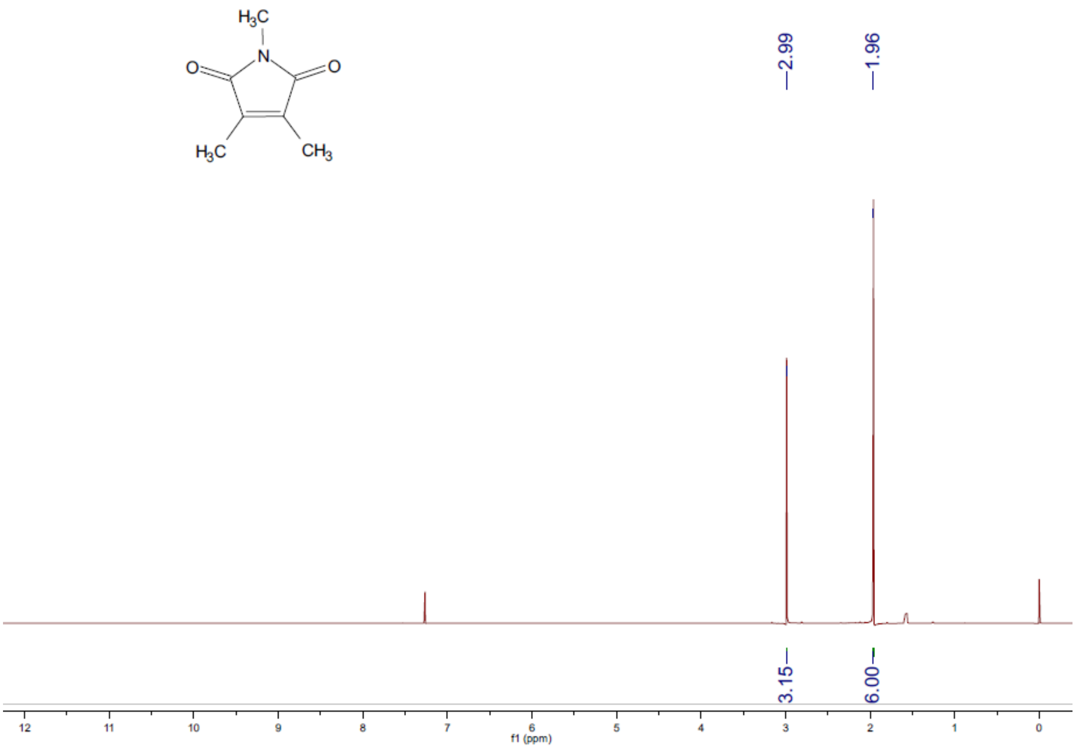

Supplementary Figure 237. ^{1}H NMR (400 MHz, $CDCl_3$) spectra of 1,3,4-trimethyl-1*H*-pyrrole-2,5-dione (**2s**)

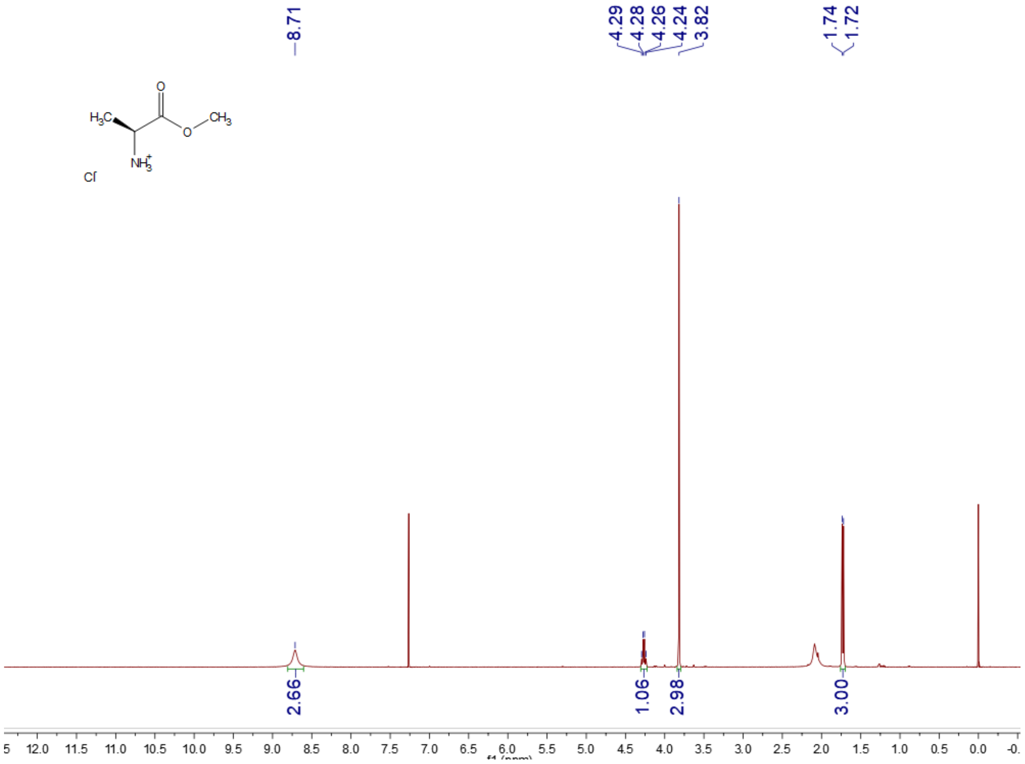

Supplementary Figure 238. ^{1}H NMR (400 MHz, $CDCl_3$) spectra of L-Alanine methyl ester hydrochloride (**2′t**)

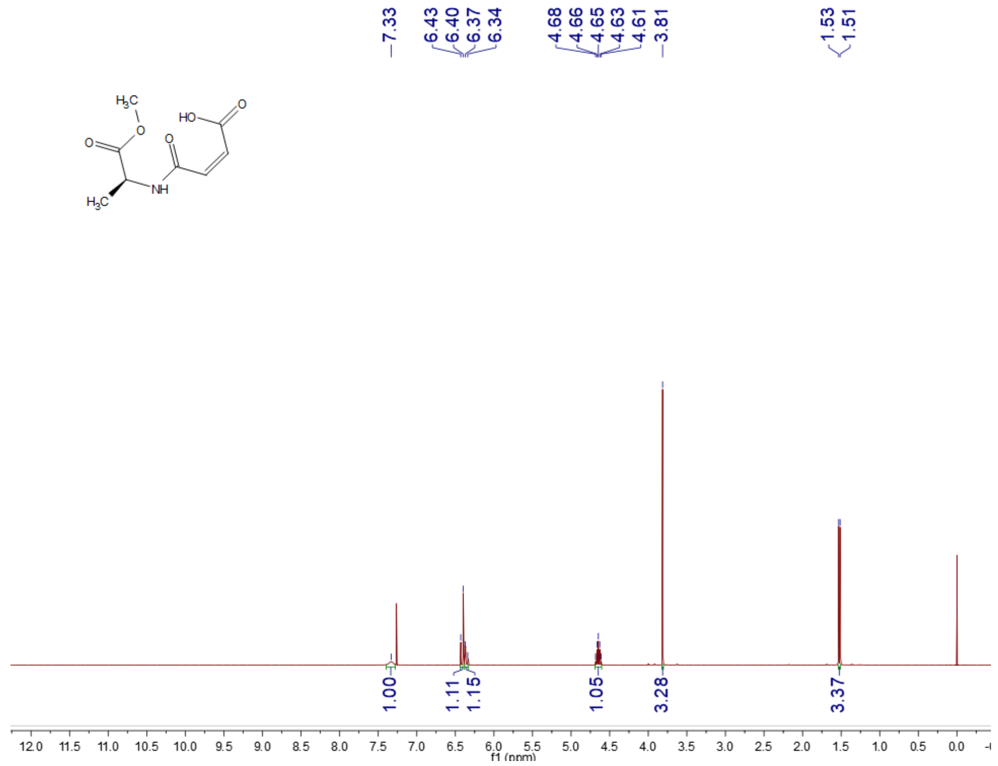

Supplementary Figure 239. ^{1}H NMR (400 MHz, $CDCl_3$) spectra of (S,*Z*)-4-((1-methoxy-1-oxopropan-2-yl)amino)-4-oxobut-2-enoic acid (**2t**)

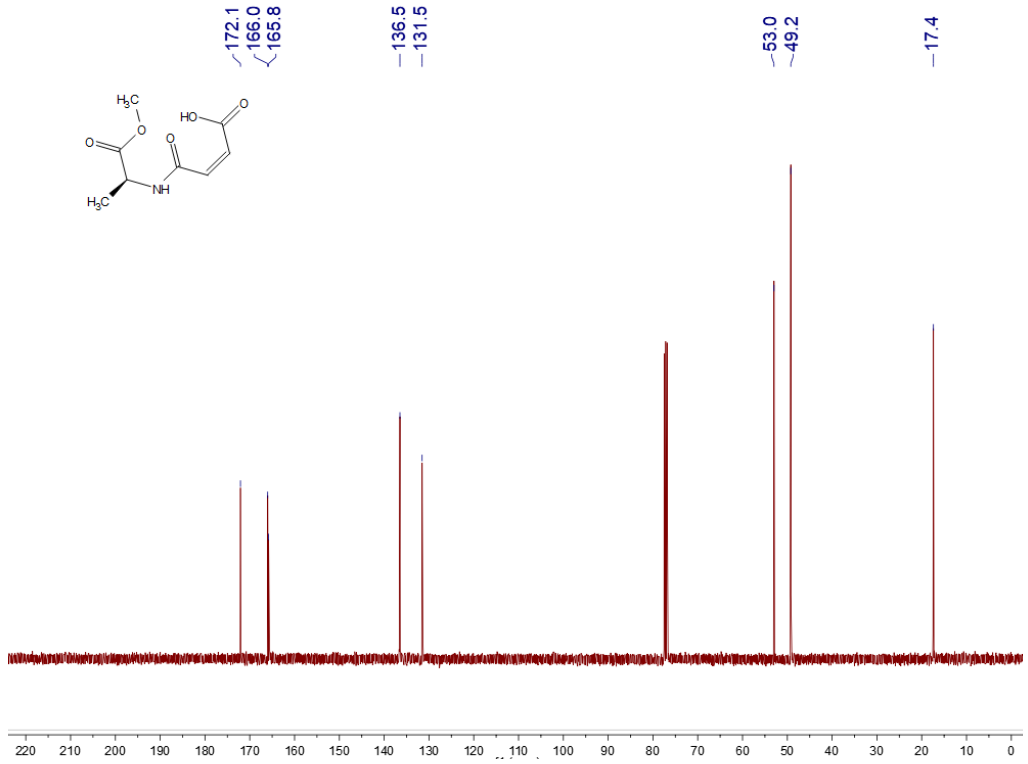

Supplementary Figure 240. ^{13}C NMR (100 MHz, $CDCl_3$) spectra of (S,*Z*)-4-((1-methoxy-1-oxopropan-2-yl)amino)-4-oxobut-2-enoic acid (**2t**)

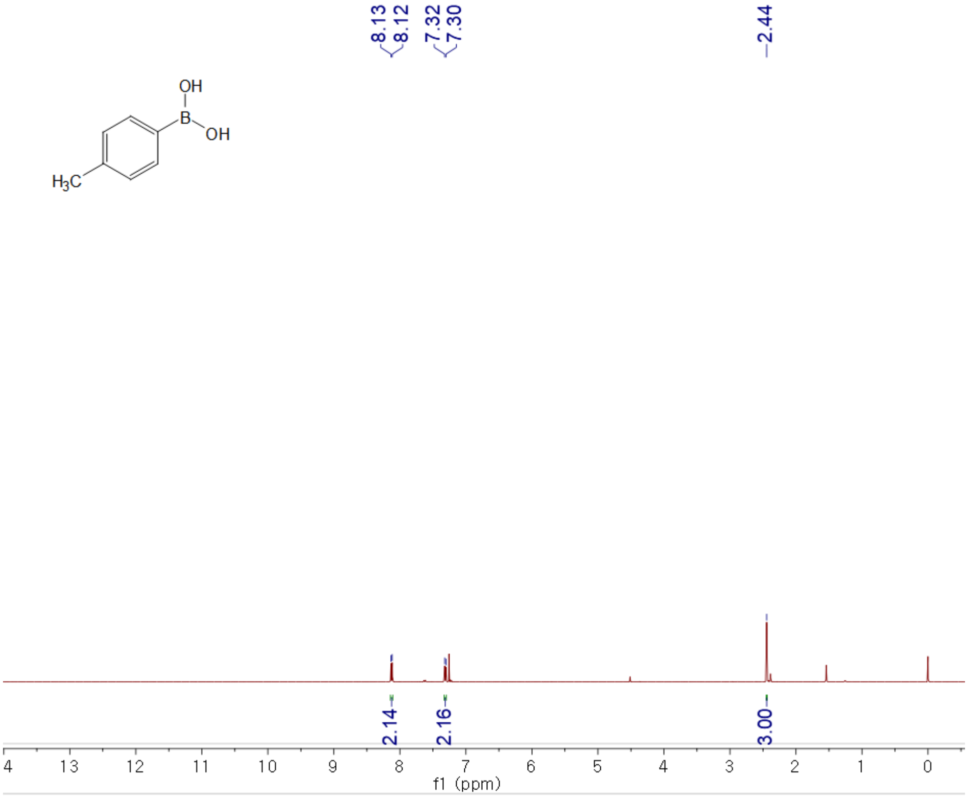

Supplementary Figure 241. ^{1}H NMR (400 MHz, $CDCl_3$) spectra of *p*-tolylboronic acid (**2′v**)

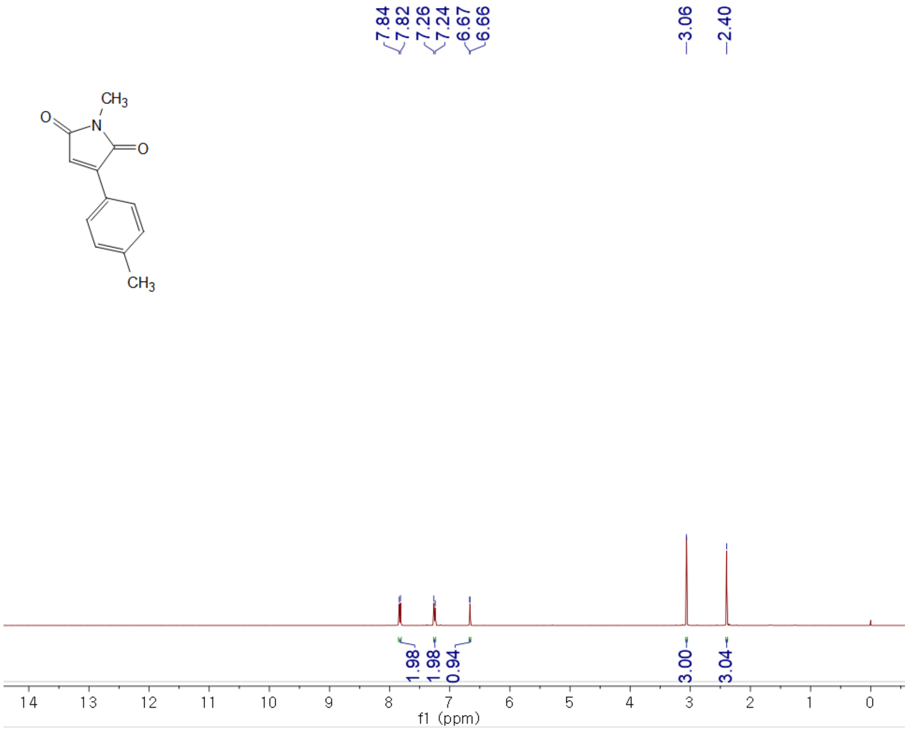

Supplementary Figure 242. ^{1}H NMR (400 MHz, $CDCl_3$) spectra of 1-methyl-3-(p-tolyl)-1H-pyrrole-2,5-dione (**2″v**)

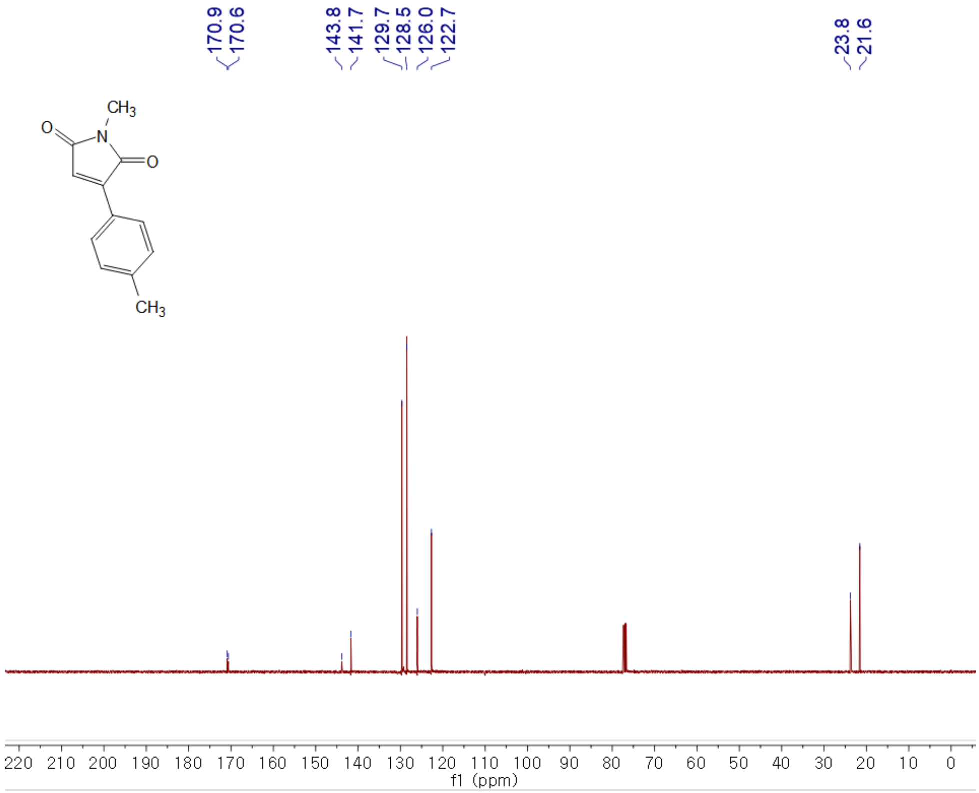

Supplementary Figure 243. ^{13}C NMR (100 MHz, $CDCl_3$) spectra of 1-methyl-3-(p-tolyl)-1H-pyrrole-2,5-dione (**2′′v**)

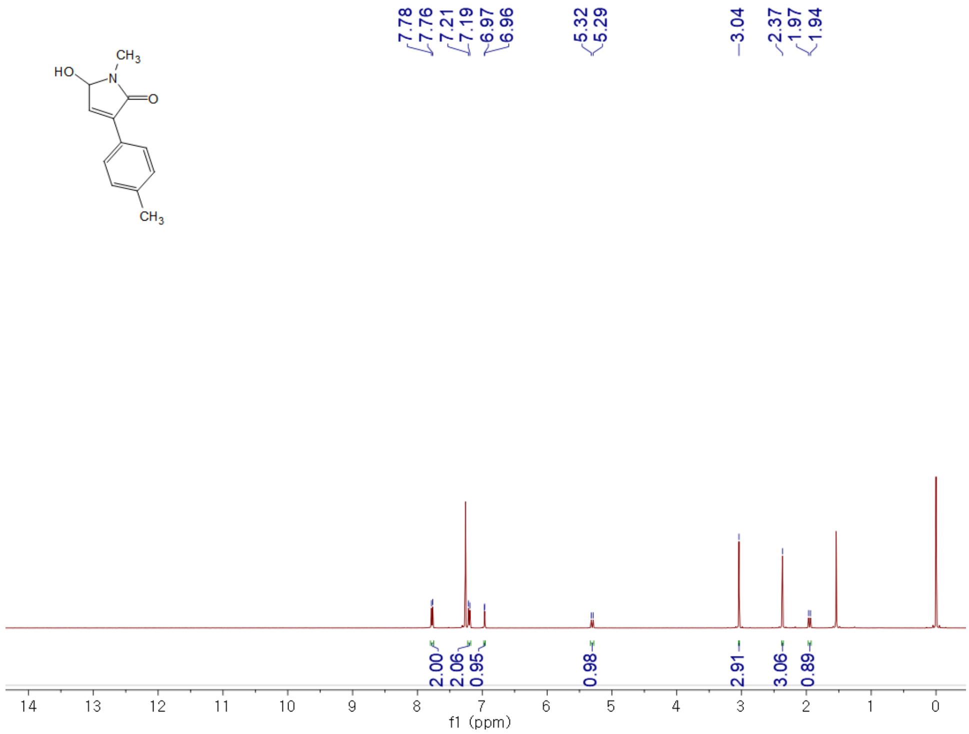

Supplementary Figure 244. ^{1}H NMR (400 MHz, $CDCl_3$) spectra of 5-hydroxy-1-methyl-3-(p-tolyl)-1,5-dihydro-2H-pyrrol-2-one (**2′′′v**)

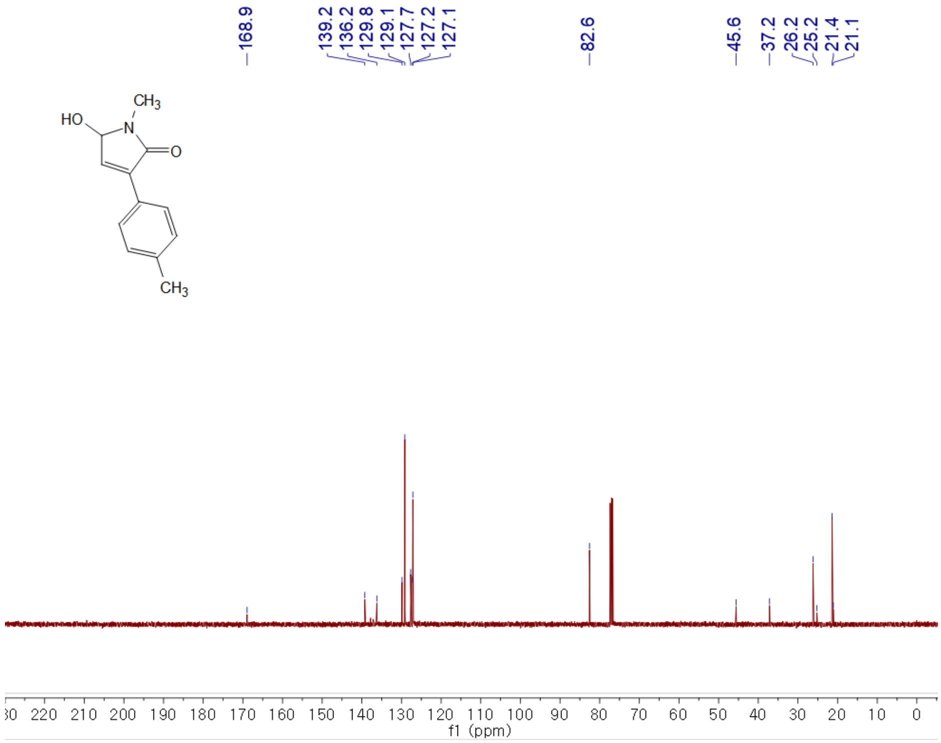

Supplementary Figure 245. ^{13}C NMR (100 MHz, $CDCl_3$) spectra of 5-hydroxy-1-methyl-3-(p-tolyl)-1,5-dihydro-2H-pyrrol-2-one (**2′′′v**)

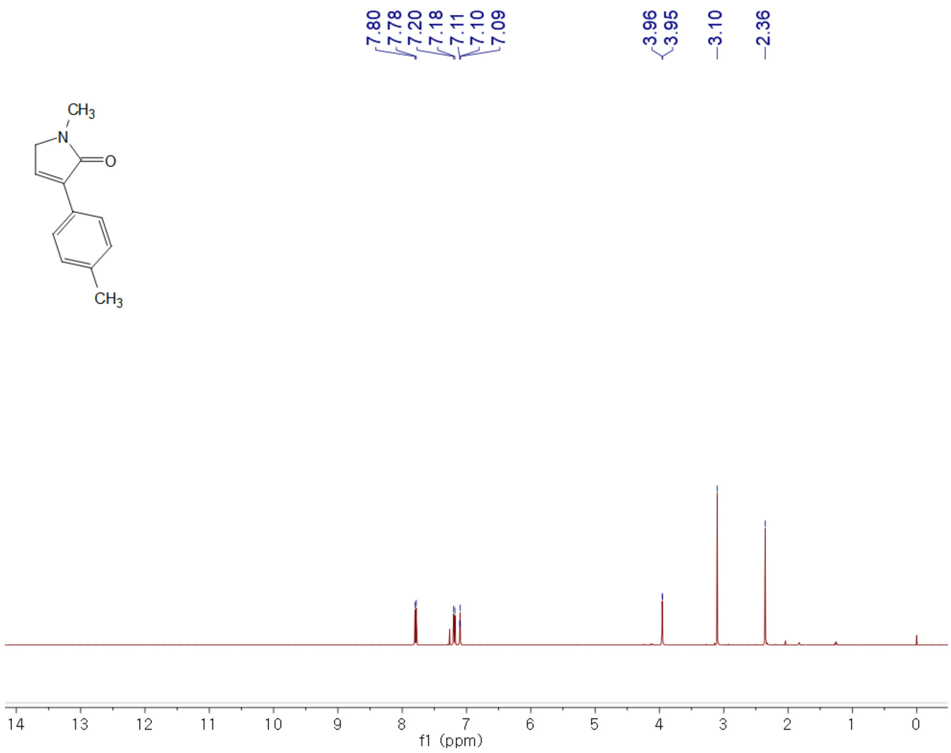

Supplementary Figure 246. ^{1}H NMR (400 MHz, $CDCl_3$) spectra of 1-methyl-3-(p-tolyl)-1,5-dihydro-2H-pyrrol-2-one (**2v**)

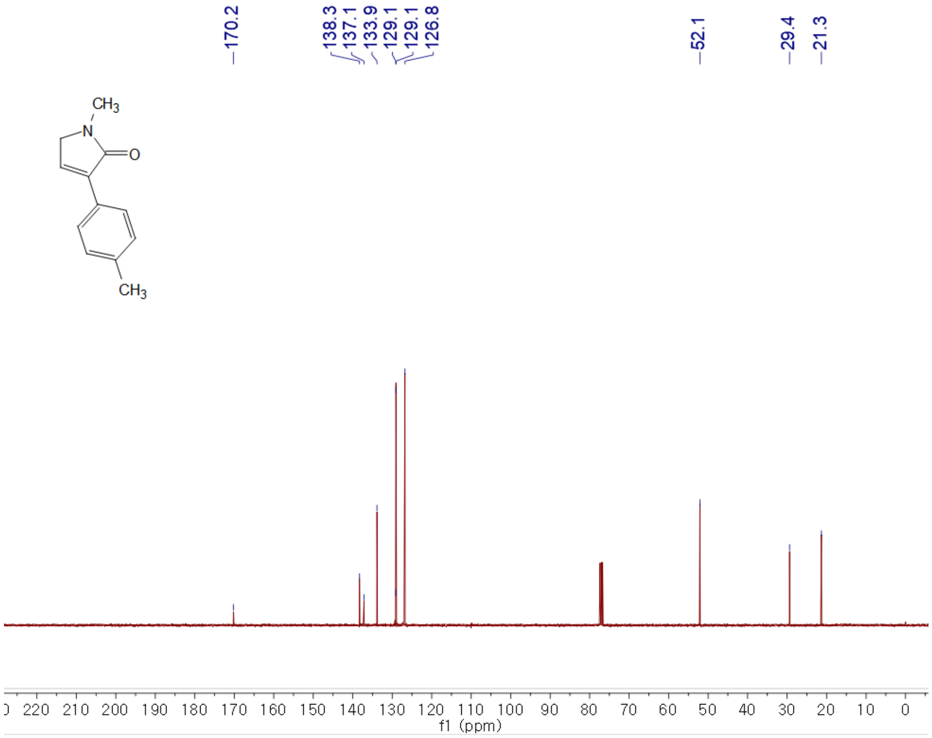

Supplementary Figure 247. ^{13}C NMR (100 MHz, $CDCl_3$) spectra of 1-methyl-3-(p-tolyl)-1,5-dihydro-2H-pyrrol-2-one (**2v**)

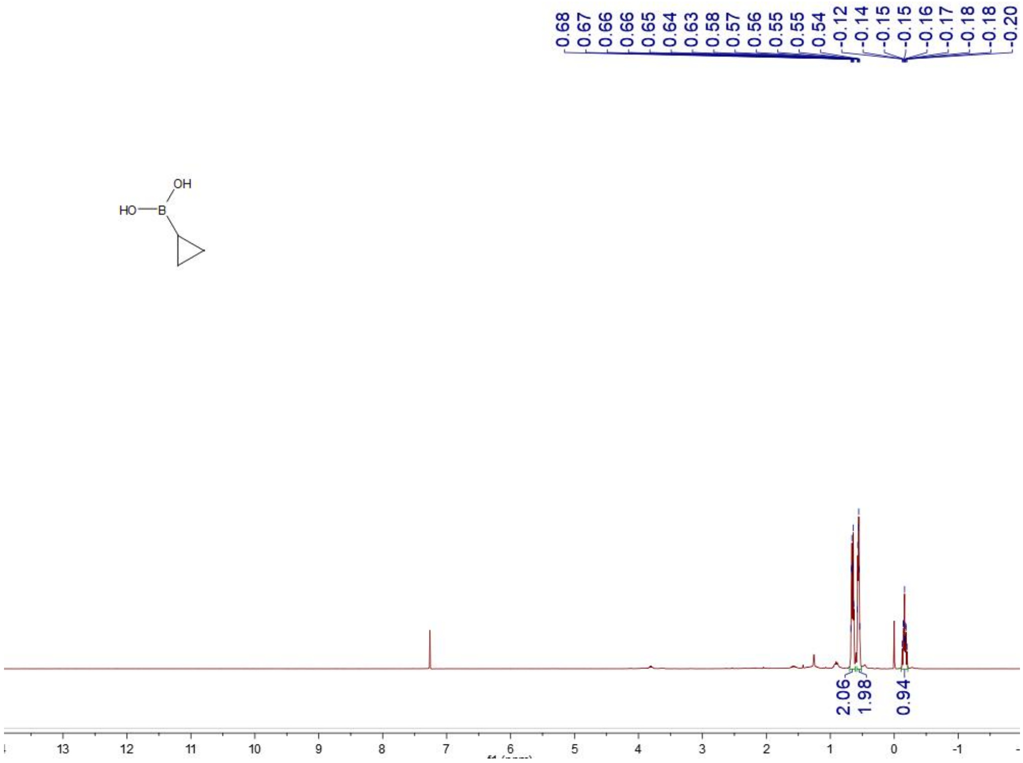

Supplementary Figure 248. ^{1}H NMR (400 MHz, $CDCl_3$) spectra of Cyclopropylboronic acid (**2′y**)

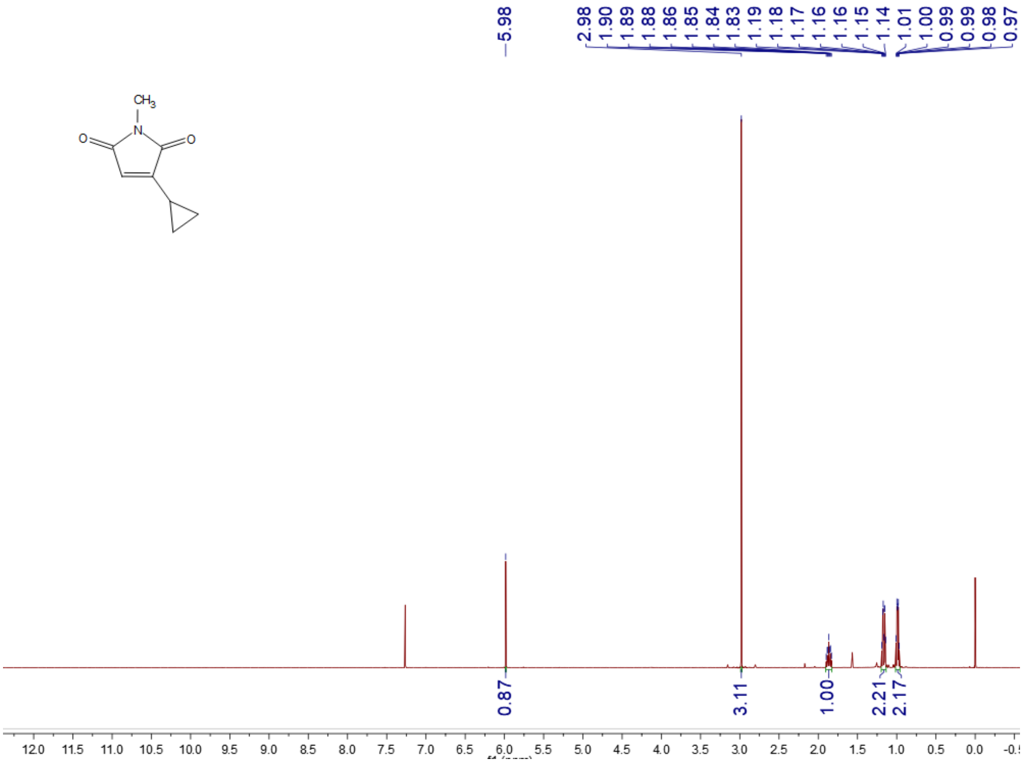

Supplementary Figure 249. ^{1}H NMR (400 MHz, $CDCl_3$) spectra of 3-cyclopropyl-1-methyl-1*H*-pyrrole-2,5-dione (**2y**)

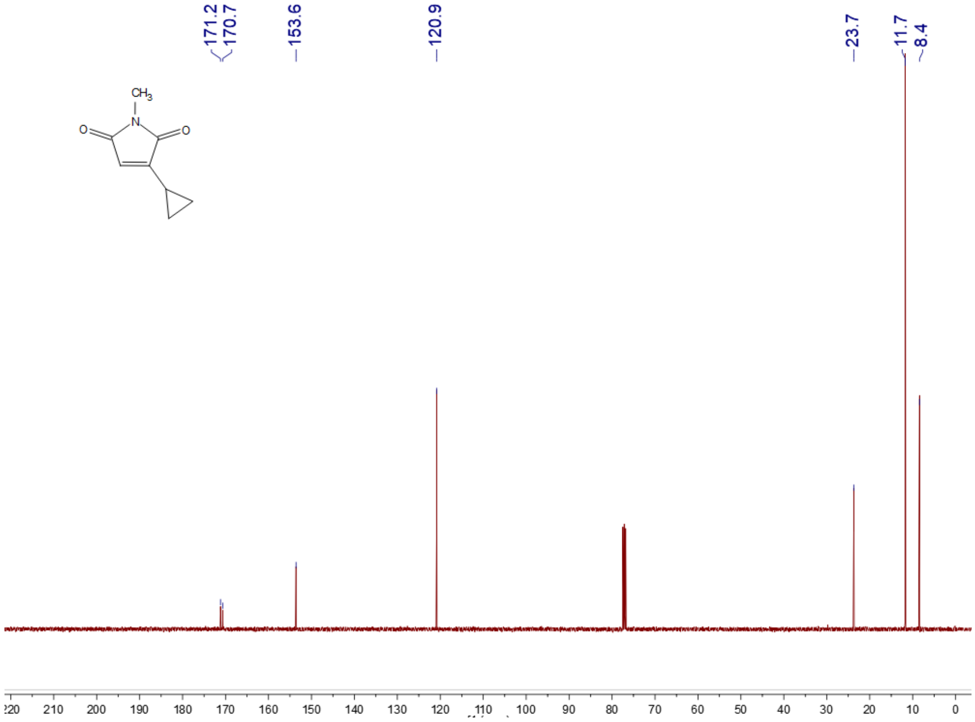

Supplementary Figure 250. ^{13}C NMR (100 MHz, $CDCl_3$) spectra of 3-cyclopropyl-1-methyl-1*H*-pyrrole-2,5-dione (**2y**)

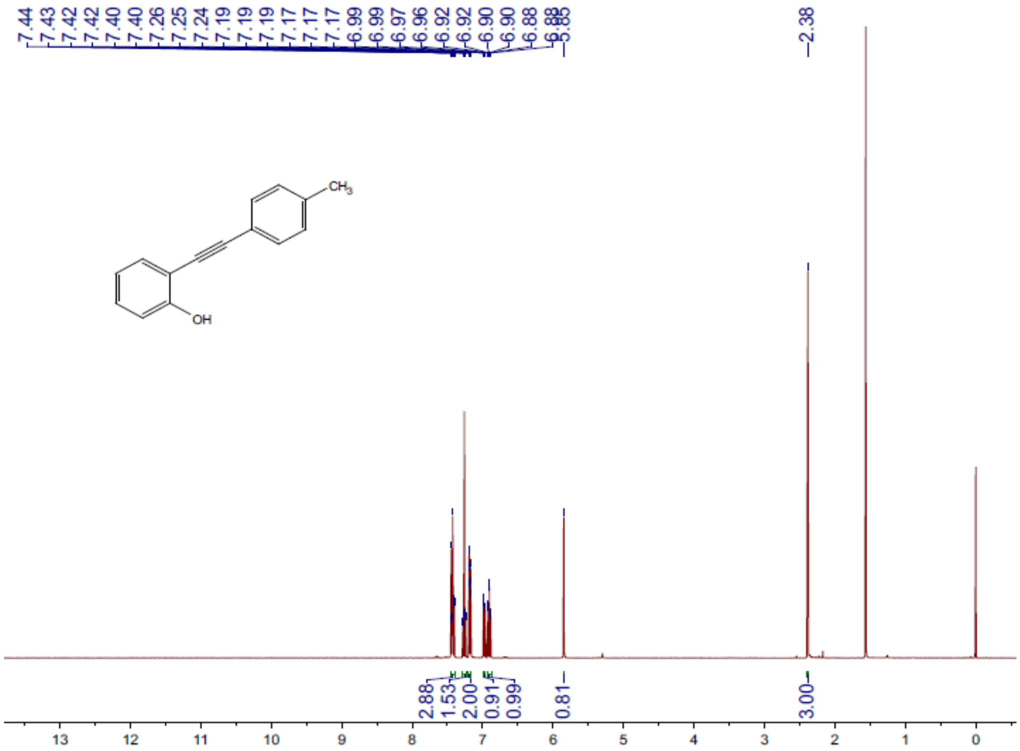

Supplementary Figure 251. ^{1}H NMR (400 MHz, $CDCl_3$) spectra of 2-(*p*-tolylethynyl)phenol (**S1**)

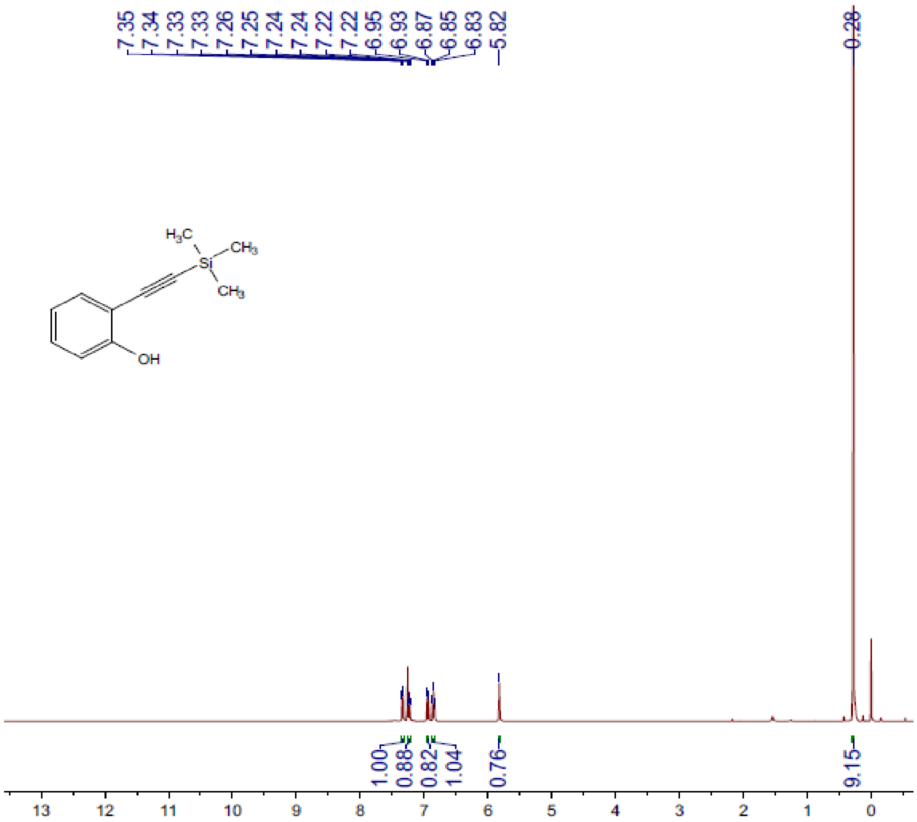

Supplementary Figure 252. ^{1}H NMR (400 MHz, $CDCl_3$) spectra of 2-((trimethylsilyl)ethynyl)phenol (**S2**)

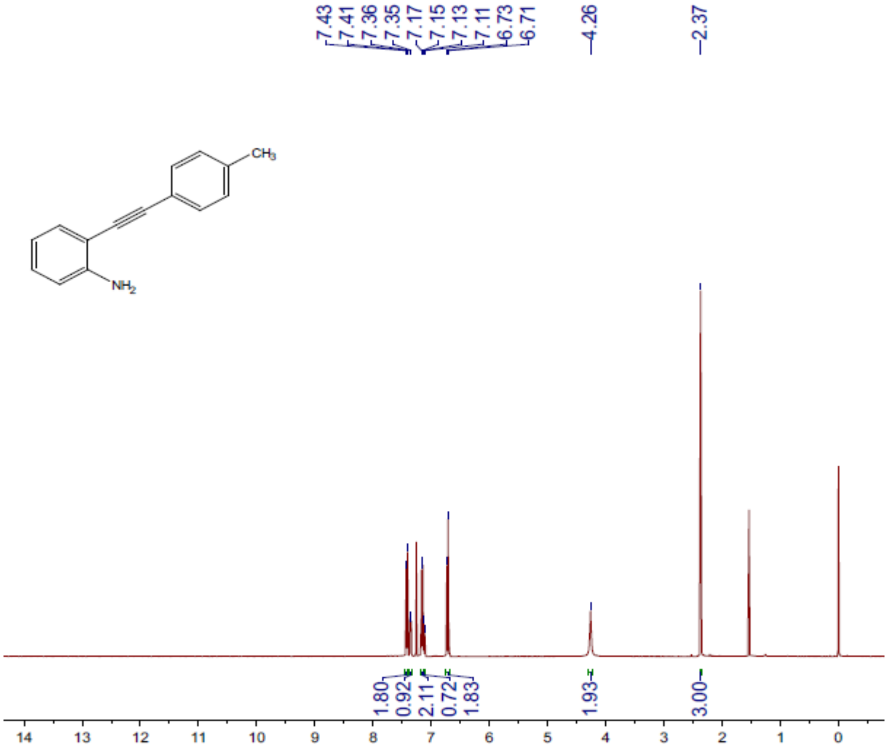

Supplementary Figure 253. ^{1}H NMR (400 MHz, $CDCl_3$) spectra of 2-(*p*-tolylethynyl)aniline (**S3**)

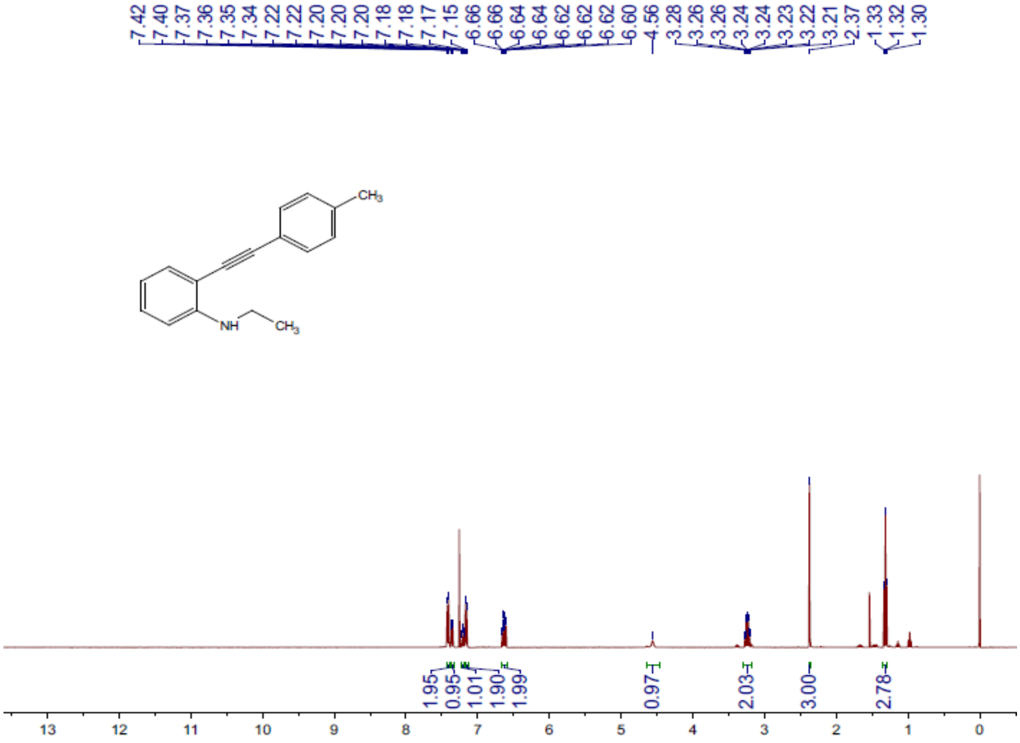

Supplementary Figure 254. ^{1}H NMR (400 MHz, $CDCl_3$) spectra of *N*-ethyl-2-(*p*-tolylethynyl)aniline (**S4**)

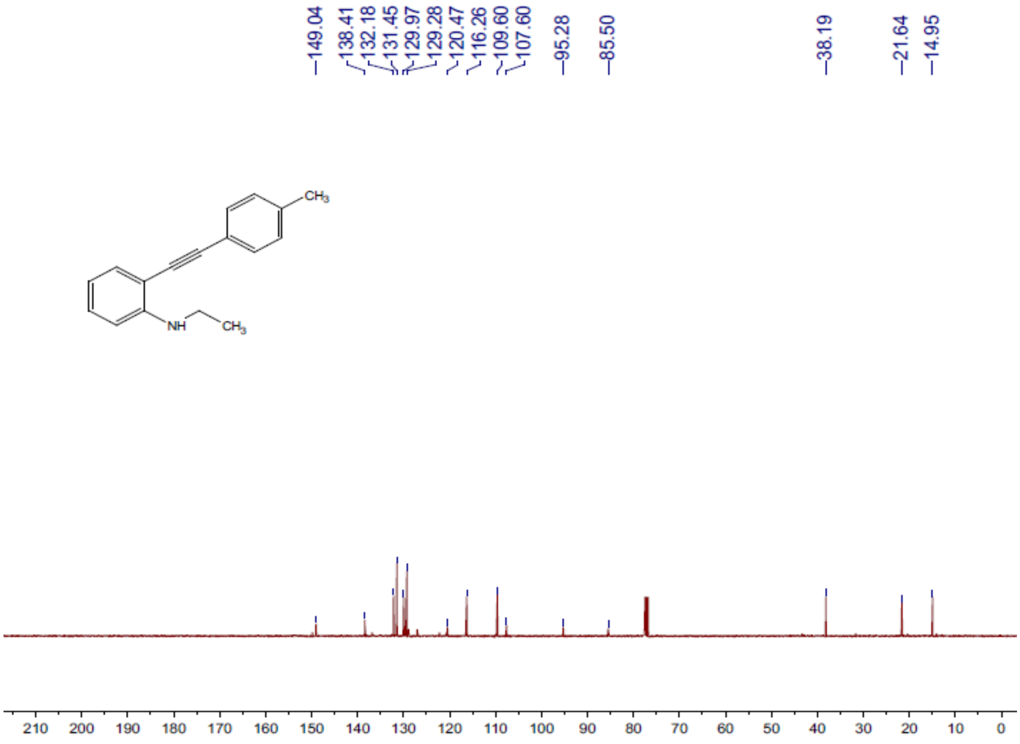

Supplementary Figure 255. ^{13}C NMR (100 MHz, $CDCl_3$) spectra of *N*-ethyl-2-(*p*-tolylethynyl)aniline (**S4**)

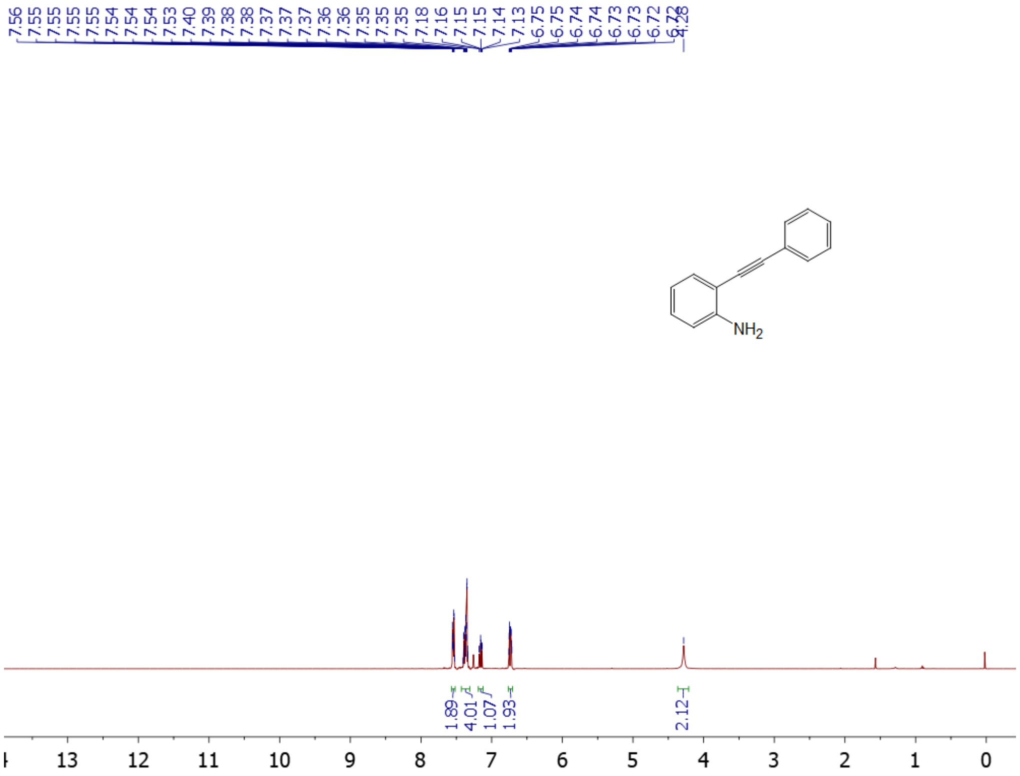

Supplementary Figure 256. ^{1}H NMR (400 MHz, $CDCl_3$) spectra of 2-(phenylethynyl)aniline (**1x**)

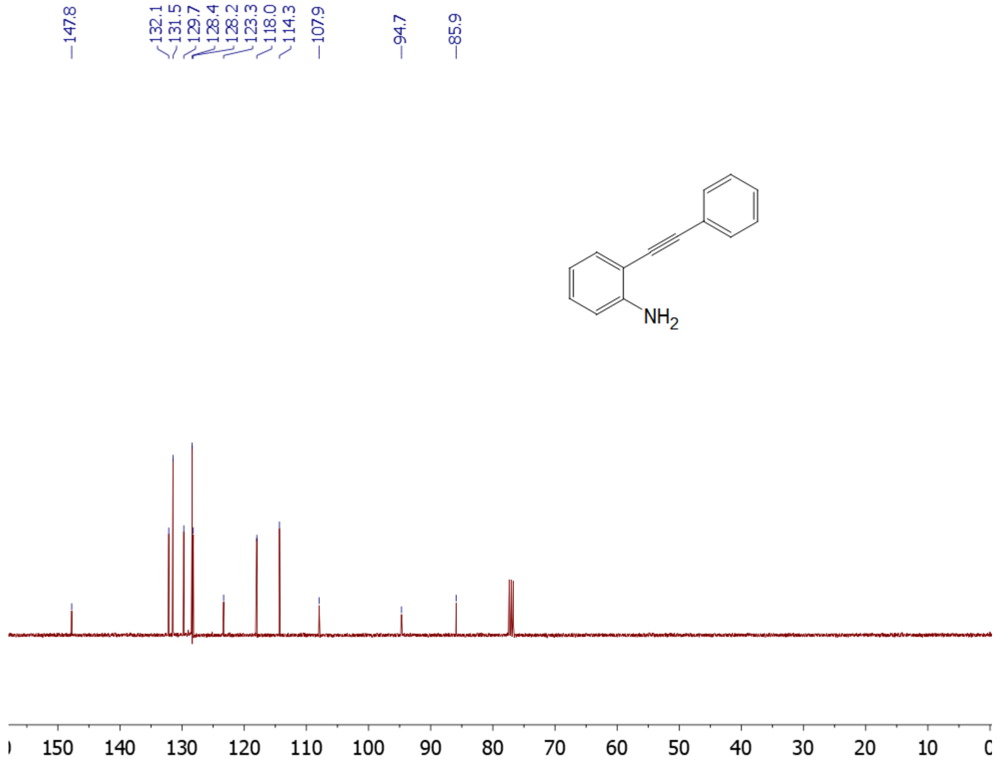

Supplementary Figure 257. ^{13}C NMR (100 MHz, $CDCl_3$) spectra of 2-(phenylethynyl)aniline (**1x**)

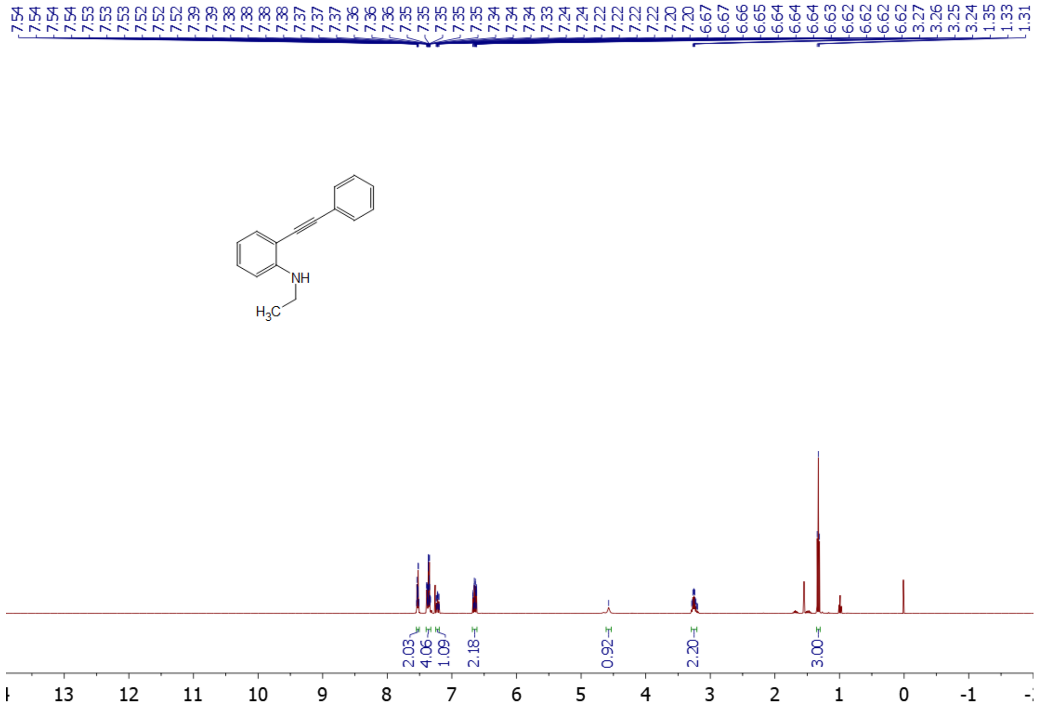

Supplementary Figure 258. ^{1}H NMR (400 MHz, $CDCl_3$) spectra of *N*-ethyl-2-(phenylethynyl)aniline (**S5**)

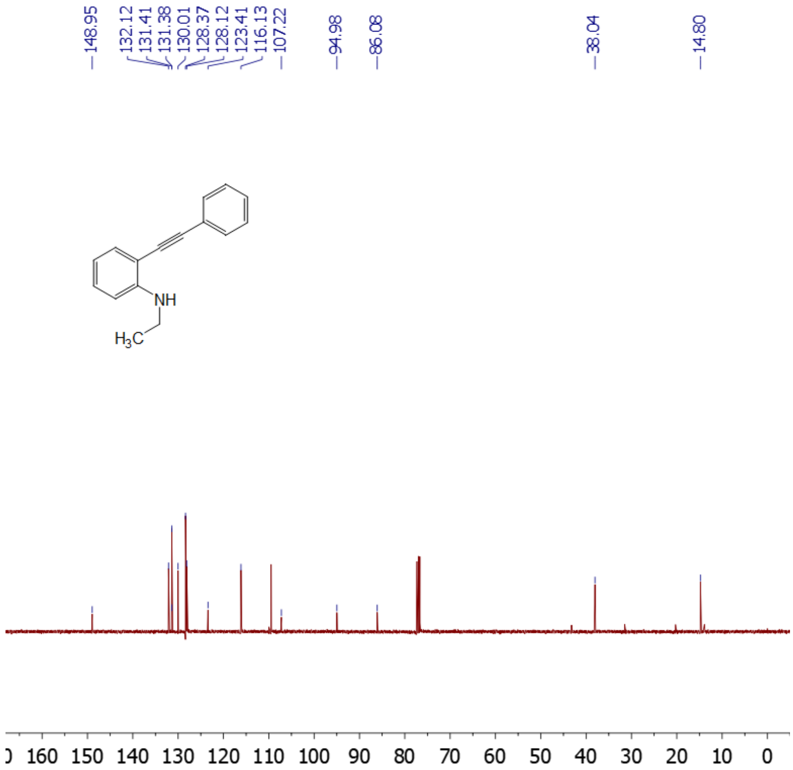

Supplementary Figure 259. ^{13}C NMR (100 MHz, $CDCl_3$) spectra of *N*-ethyl-2-(phenylethynyl)aniline (**S5**)

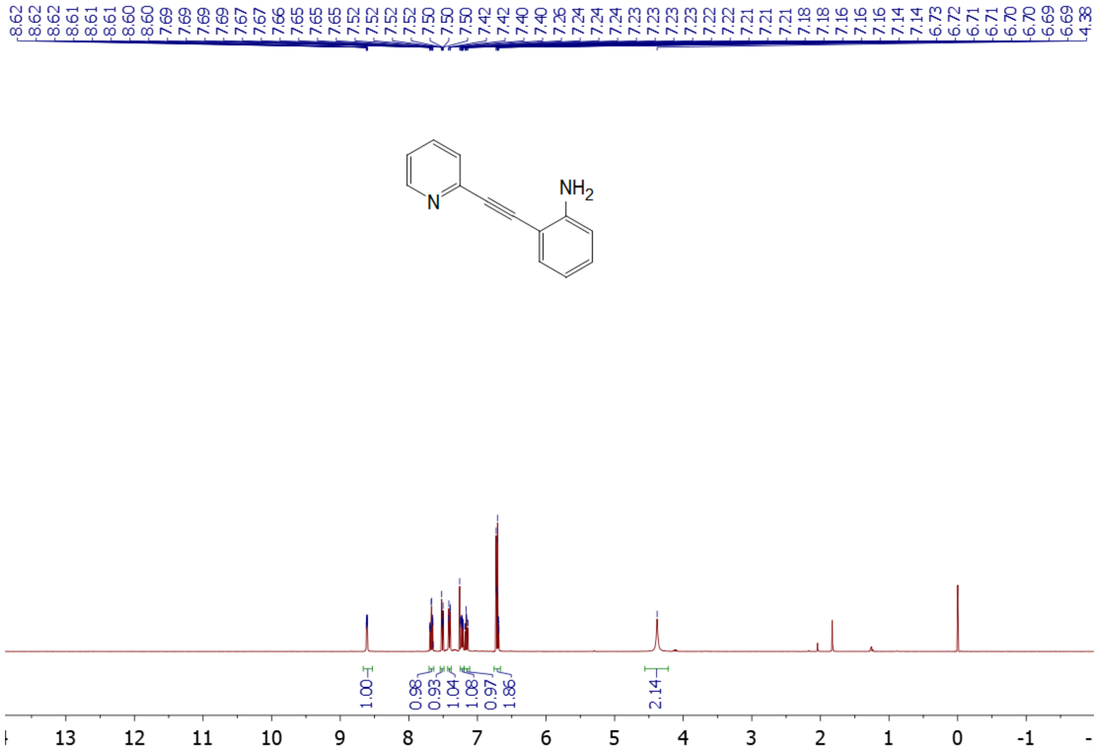

Supplementary Figure 260. ^{1}H NMR (400 MHz, $CDCl_3$) spectra of 2-(pyridin-2-ylethynyl)aniline (**S6′**)

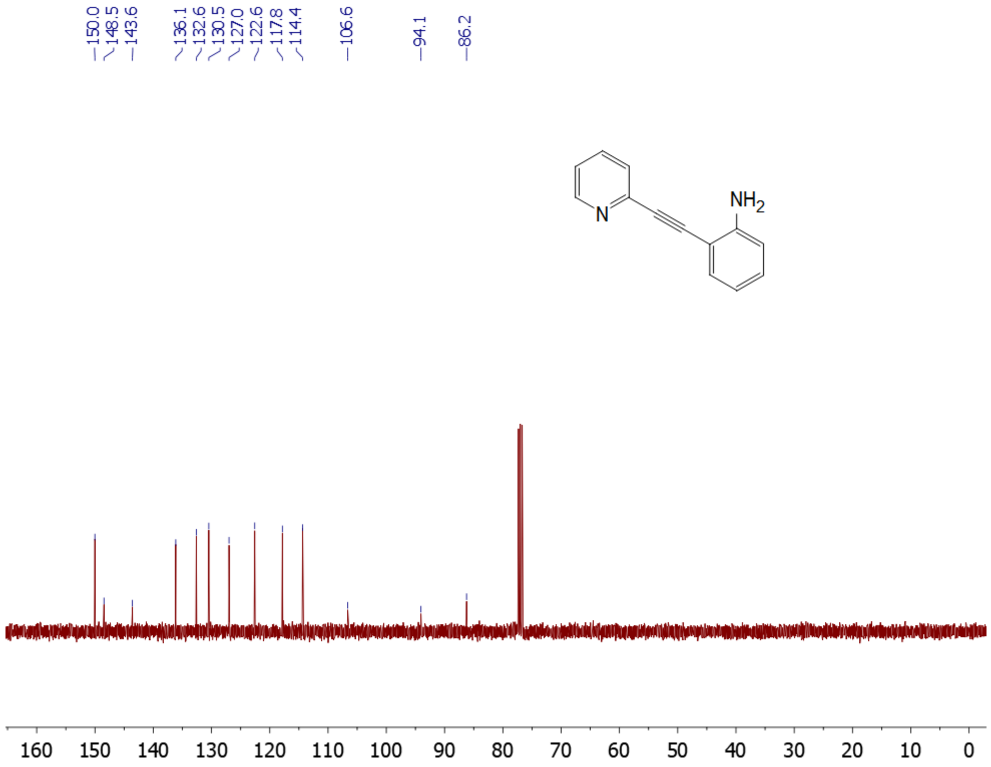

Supplementary Figure 261. ^{13}C NMR (100 MHz, $CDCl_3$) spectra of 2-(pyridin-2-ylethynyl)aniline (**S6′**)

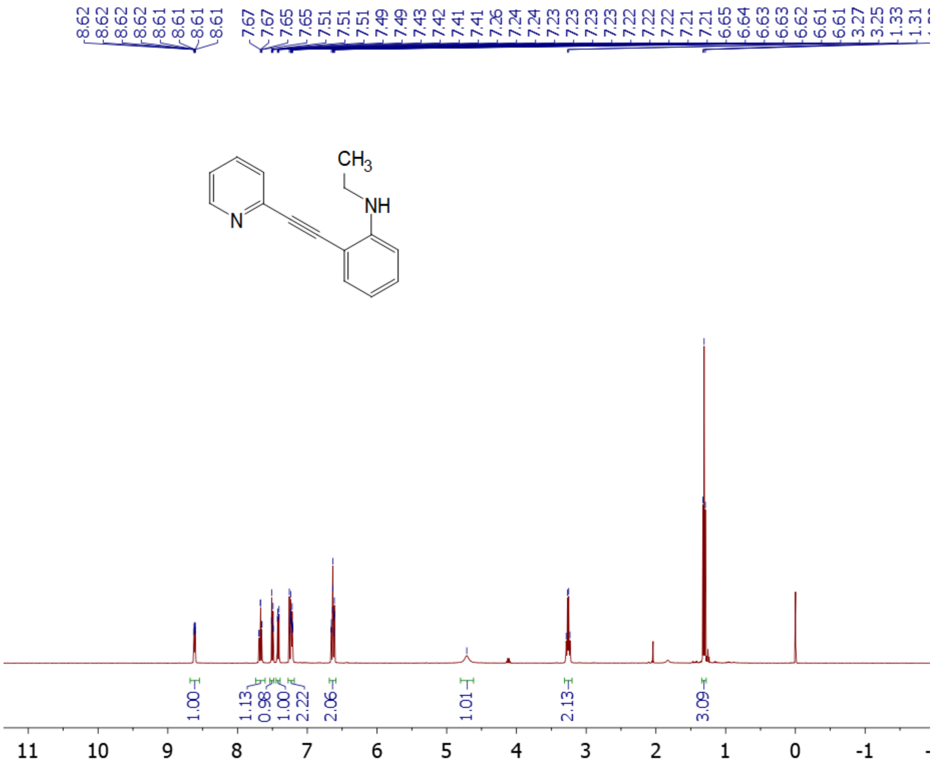

Supplementary Figure 262. ^{1}H NMR (400 MHz, $CDCl_3$) spectra of *N*-ethyl-2-(pyridin-2-ylethynyl)aniline (**S6**)

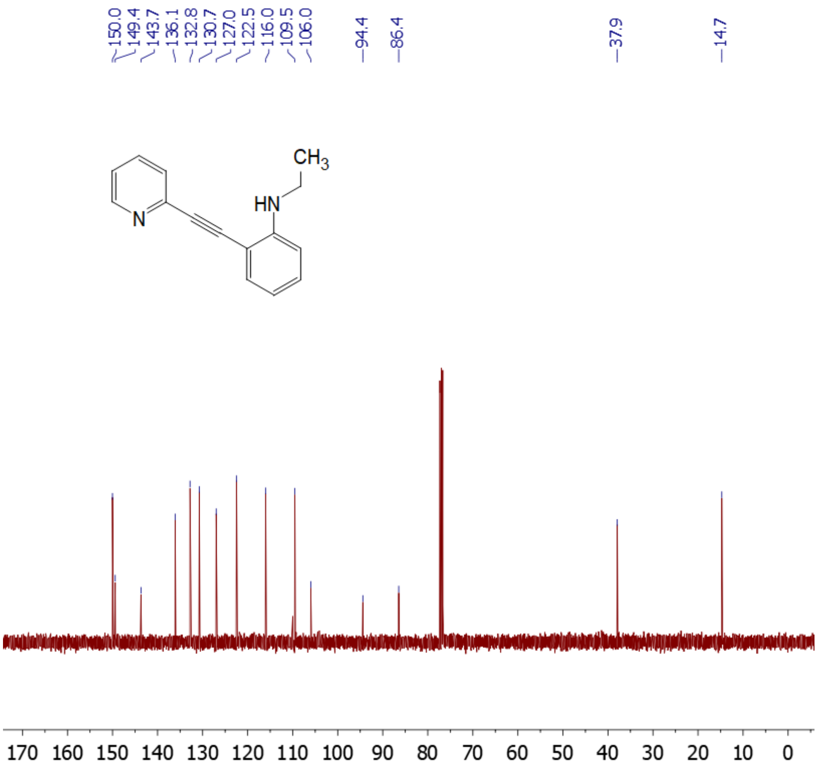

Supplementary Figure 263. ^{13}C NMR (100 MHz, $CDCl_3$) spectra of *N*-ethyl-2-(pyridin-2-ylethynyl)aniline (**S6**)

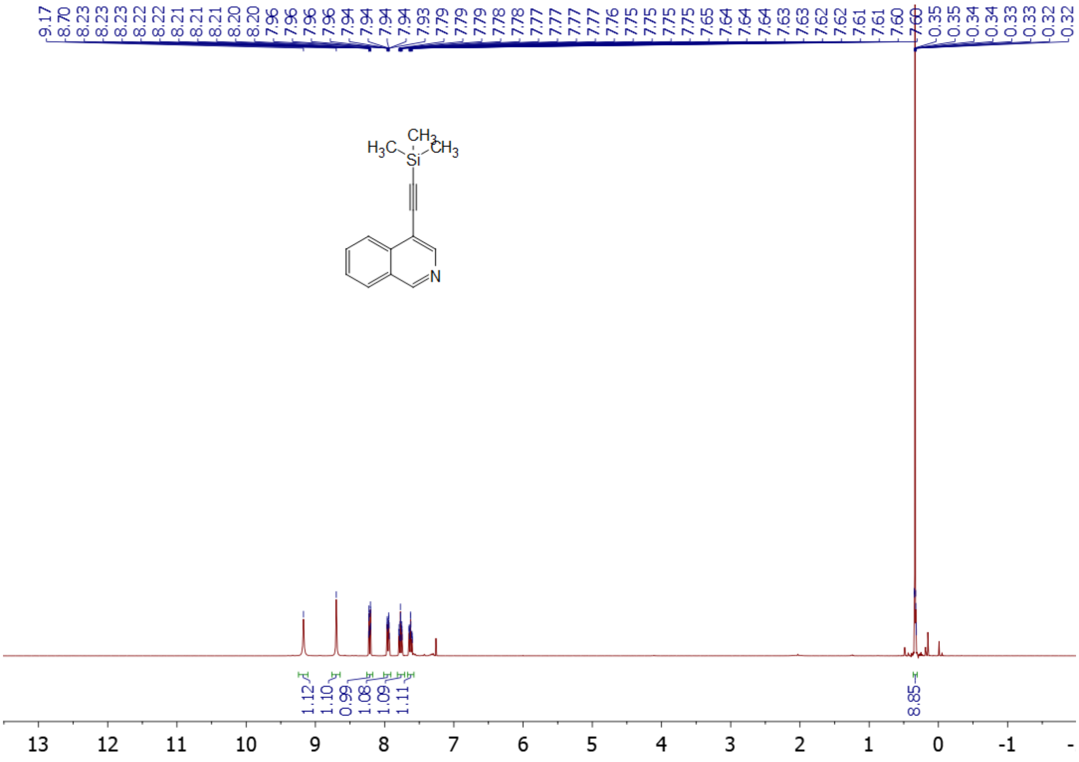

Supplementary Figure 264. ^{1}H NMR (400 MHz, $CDCl_3$) spectra of 4-((trimethylsilyl)ethynyl)isoquinoline (**S7′**)

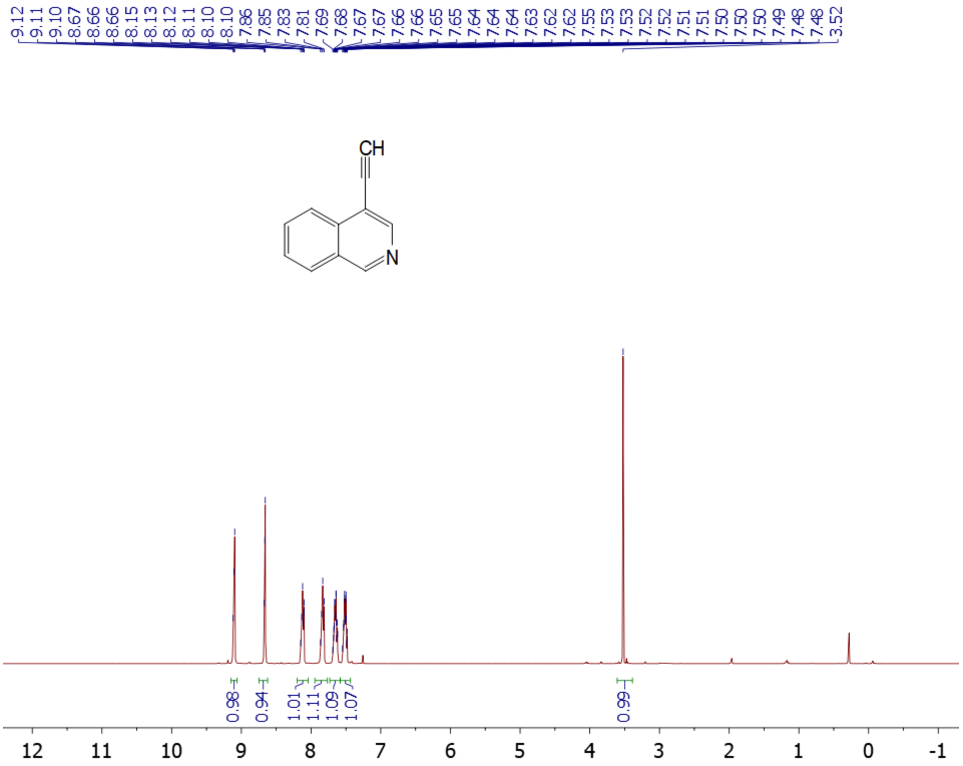

Supplementary Figure 265. ^{1}H NMR (400 MHz, $CDCl_3$) spectra of 4-ethynylisoquinoline (**S7′′**)

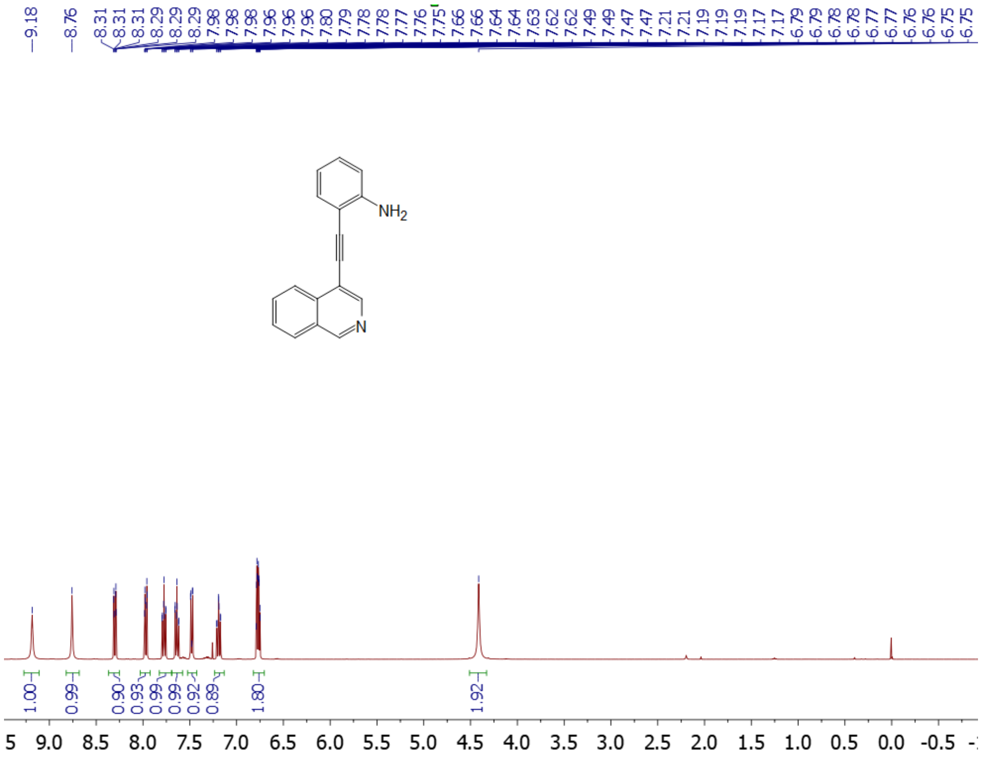

Supplementary Figure 266. ^{1}H NMR (400 MHz, $CDCl_3$) spectra of 2-(isoquinolin-4-ylethynyl)aniline (**S7′′′**)

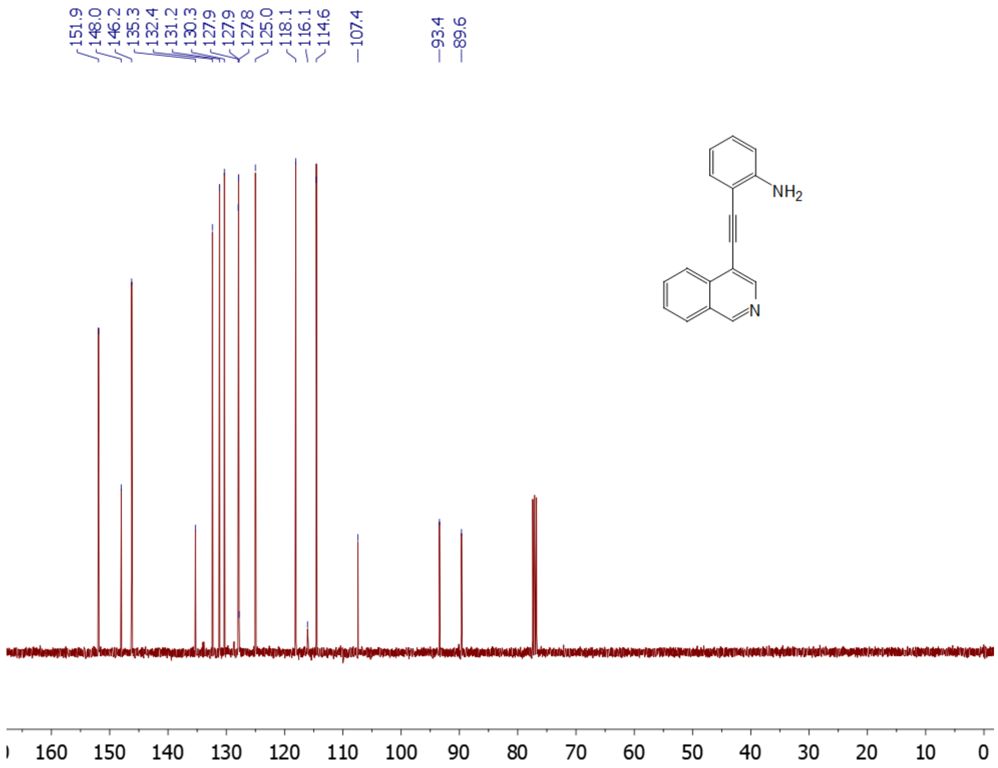

Supplementary Figure 267. ^{13}C NMR (100 MHz, $CDCl_3$) spectra of 2-(isoquinolin-4-ylethynyl)aniline (**S7′′′**)

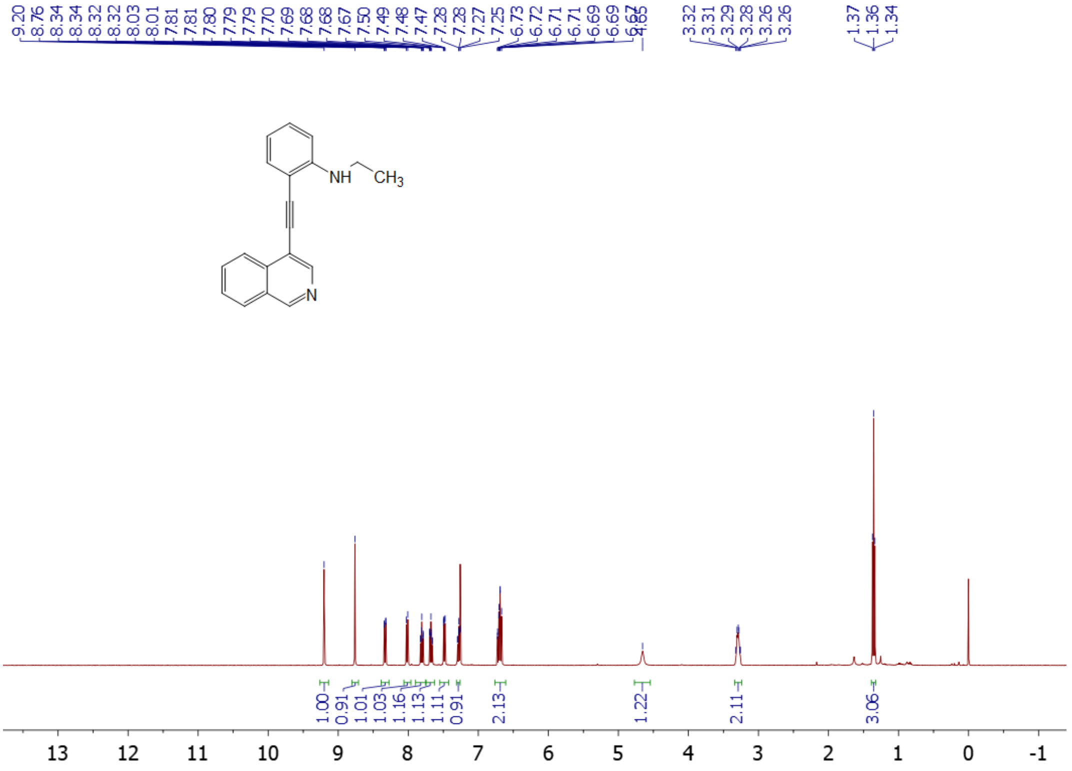

Supplementary Figure 268. ^{1}H NMR (400 MHz, $CDCl_3$) spectra of *N*-ethyl-2-(isoquinolin-4-ylethynyl)aniline (**S7**)

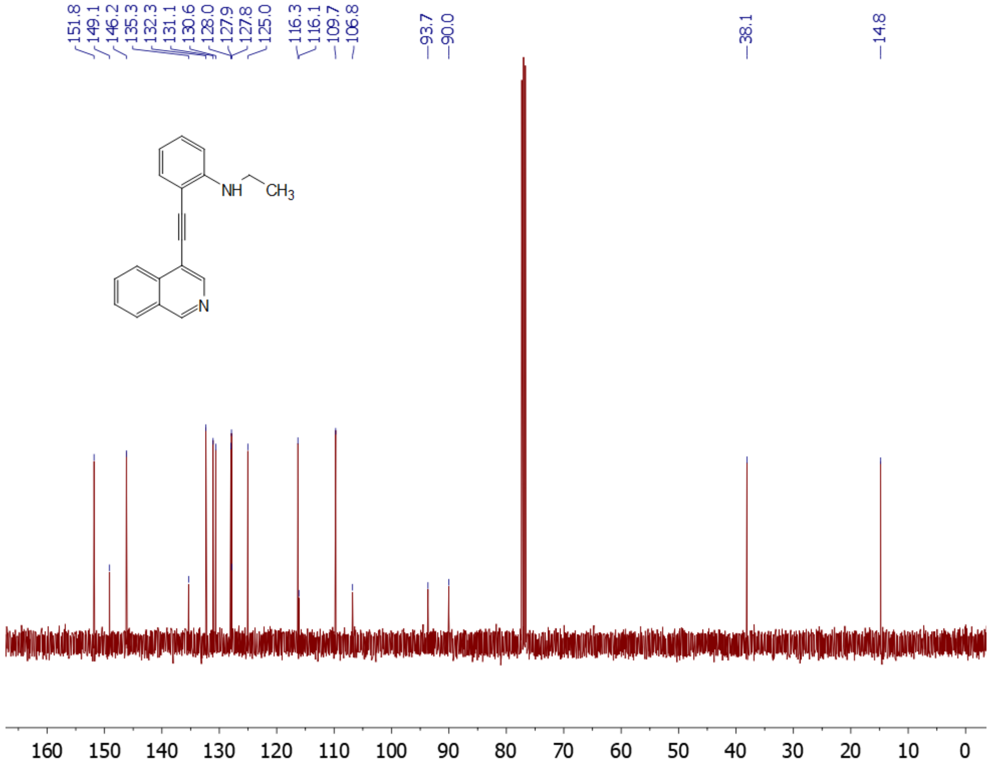

Supplementary Figure 269. ^{13}C NMR (100 MHz, $CDCl_3$) spectra of *N*-ethyl-2-(isoquinolin-4-ylethynyl)aniline (**S7**)

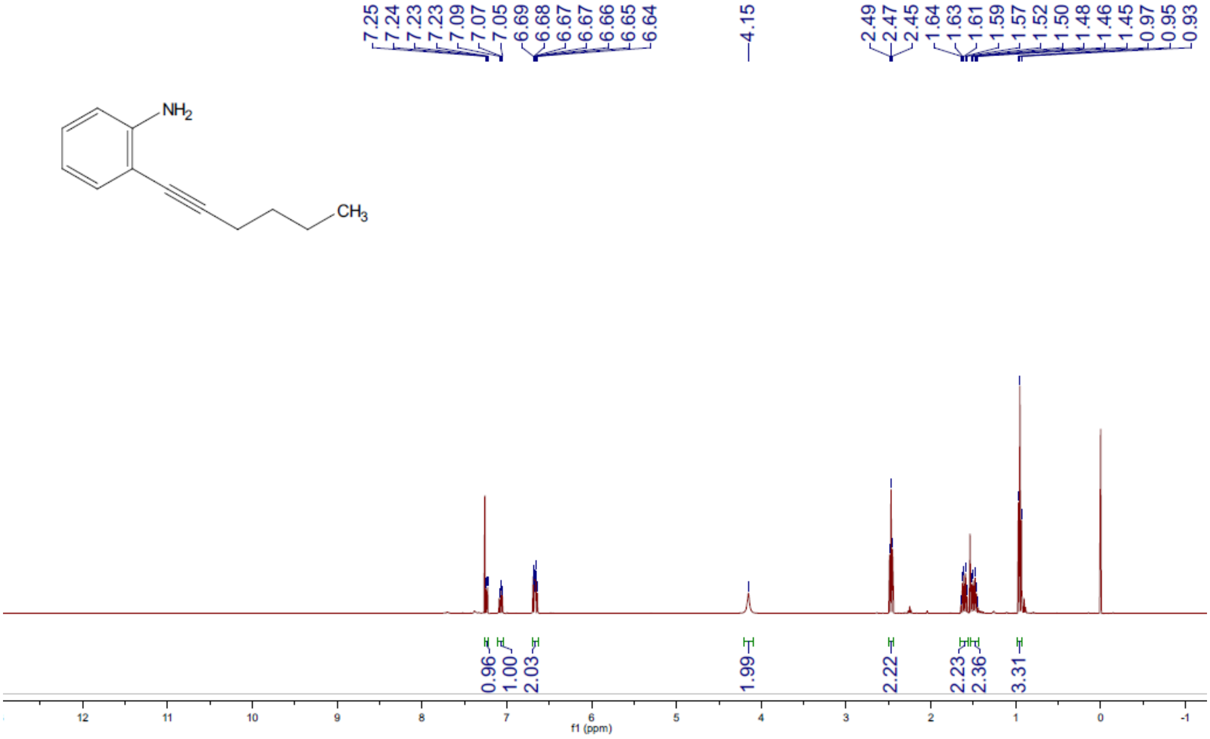

Supplementary Figure 270. ^{1}H NMR (400 MHz, $CDCl_3$) spectra of 2-(hex-1-yn-1-yl)aniline (**S8**)

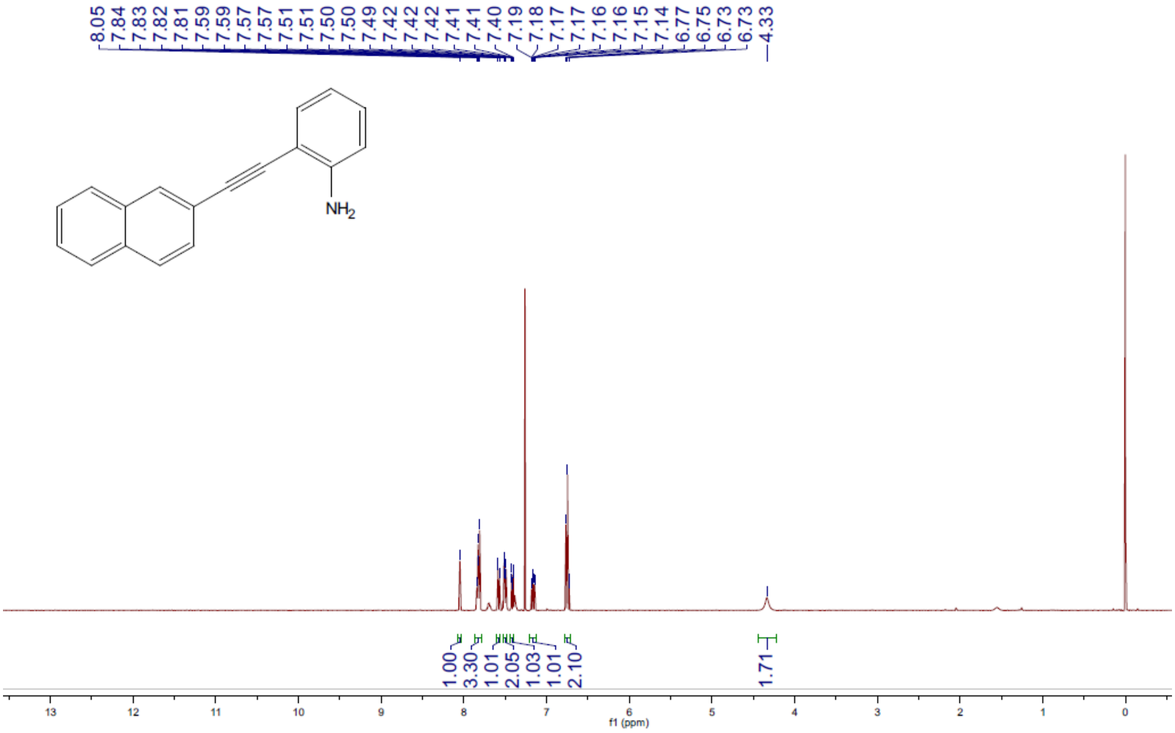

Supplementary Figure 271. ^{1}H NMR (400 MHz, $CDCl_3$) spectra of 2-(naphthalen-2-ylethynyl)aniline (**S9′**)

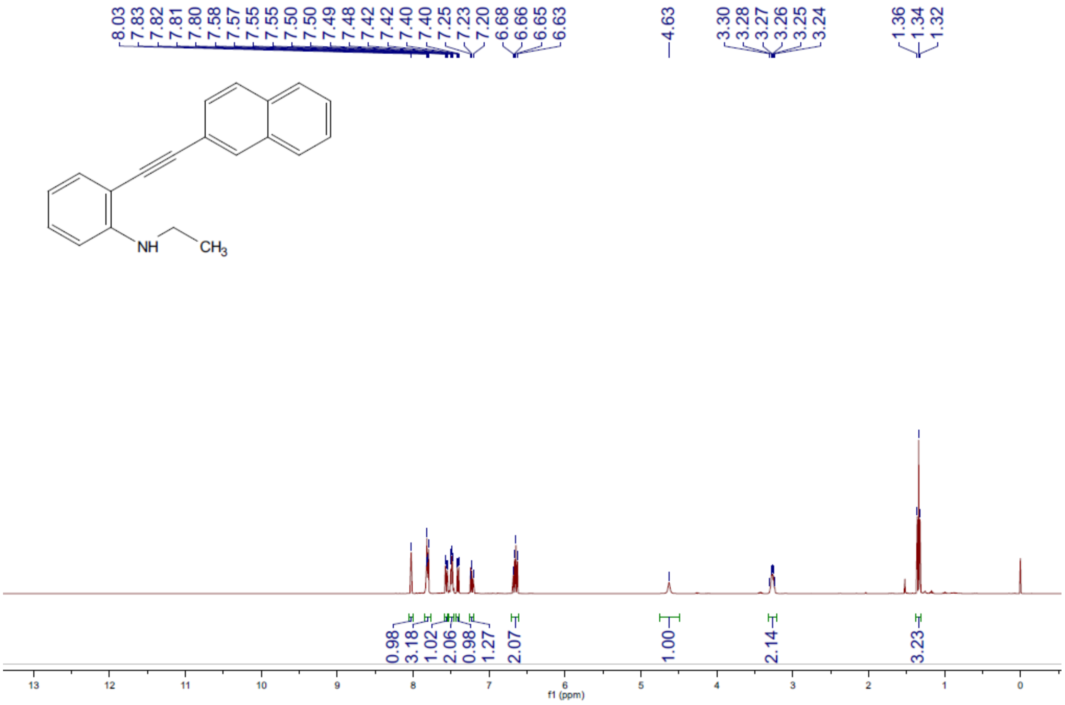

Supplementary Figure 272. ^{1}H NMR (400 MHz, $CDCl_3$) spectra of *N*-ethyl-2-(naphthalen-2-ylethynyl)aniline (**S9**)

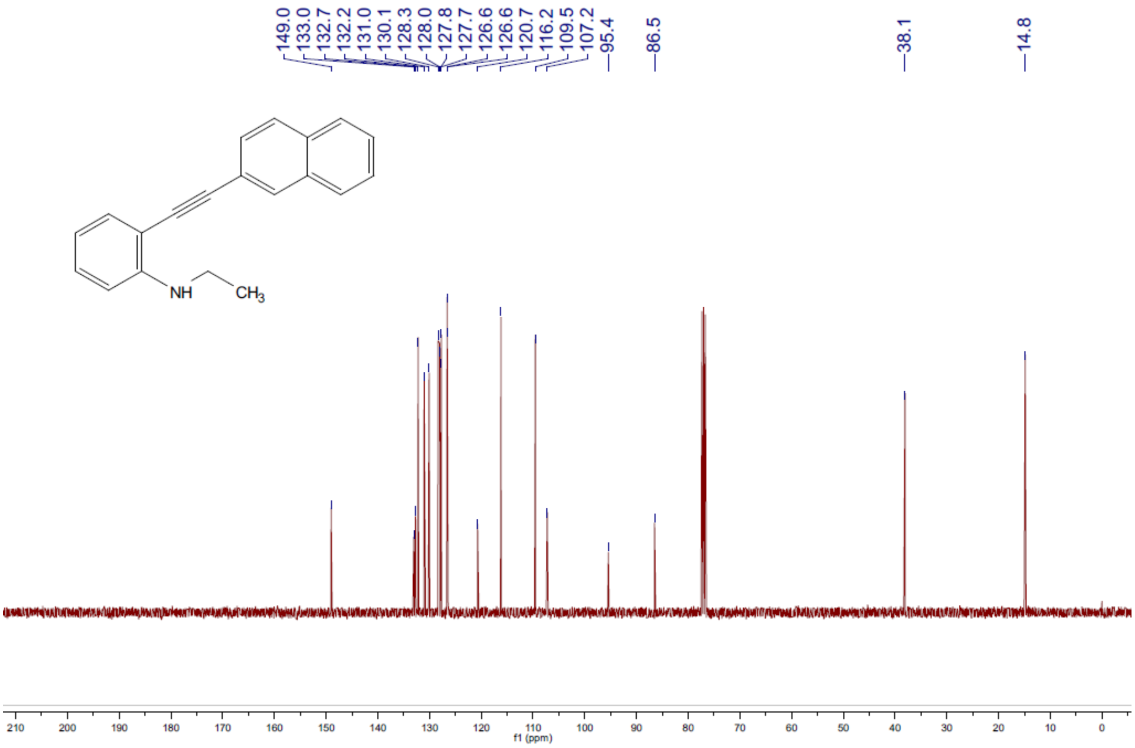

Supplementary Figure 273. ^{13}C NMR (100 MHz, $CDCl_3$) spectra of *N*-ethyl-2-(naphthalen-2-ylethynyl)aniline (**S9**)

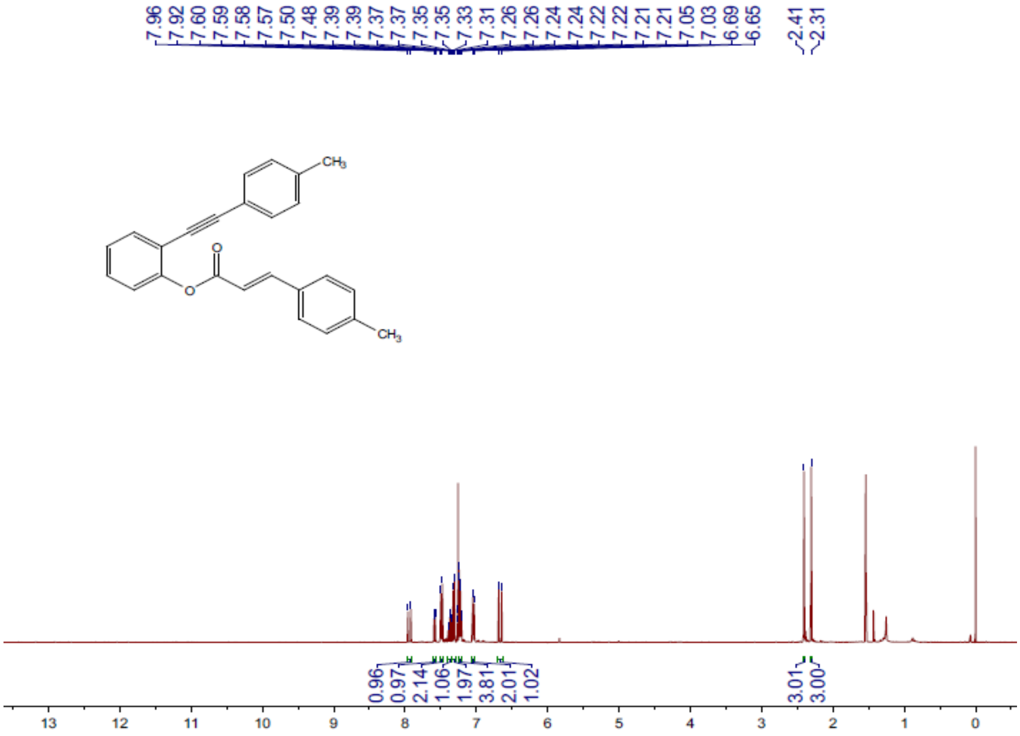

Supplementary Figure 274. ^{1}H NMR (400 MHz, $CDCl_3$) spectra of 2-(*p*-tolylethynyl)phenyl (*E*)-3-(*p*-tolyl)acrylate (**4a**)

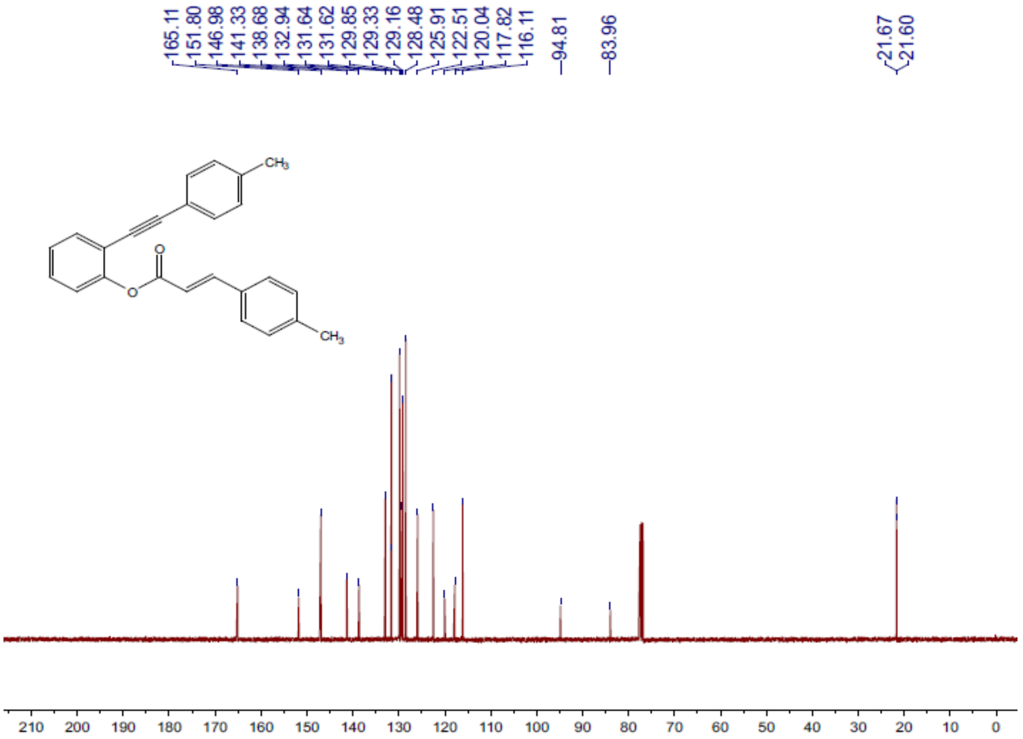

Supplementary Figure 275. ^{13}C NMR (100 MHz, $CDCl_3$) spectra of 2-(*p*-tolylethynyl)phenyl (*E*)-3-(*p*-tolyl)acrylate (**4a**)

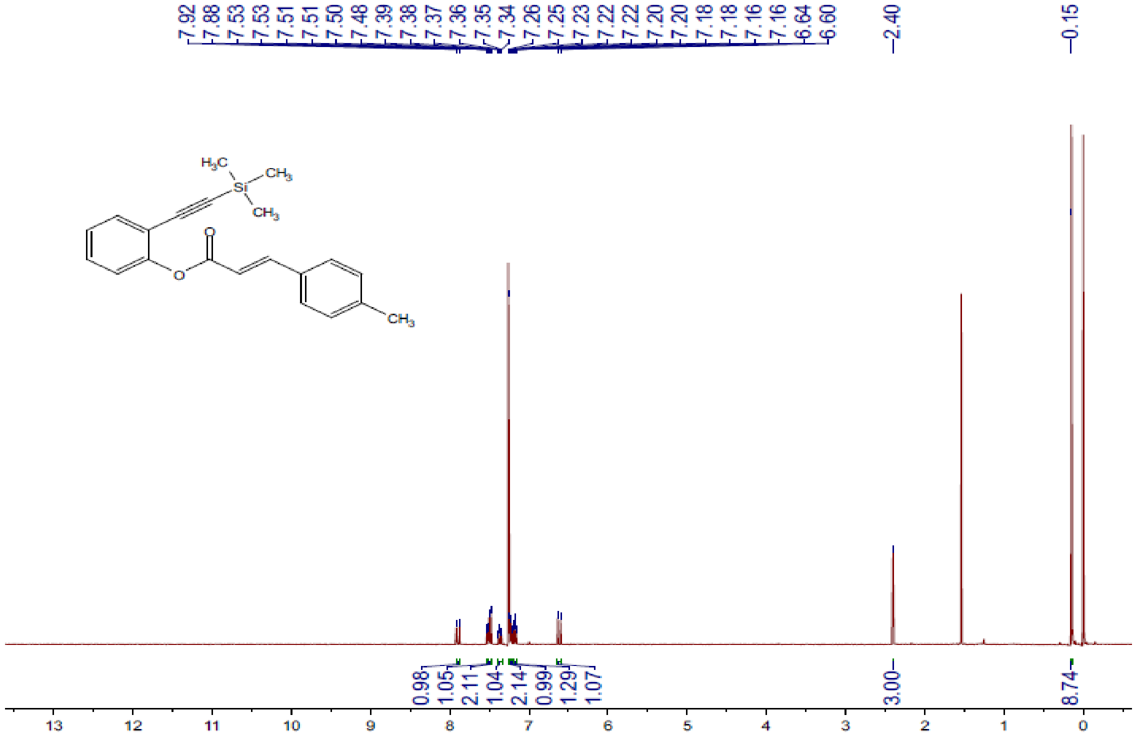

Supplementary Figure 276. ^{1}H NMR (400 MHz, $CDCl_3$) spectra of 2-((trimethylsilyl)ethynyl)phenyl (*E*)-3-(*p*-tolyl)acrylate (**4b**)

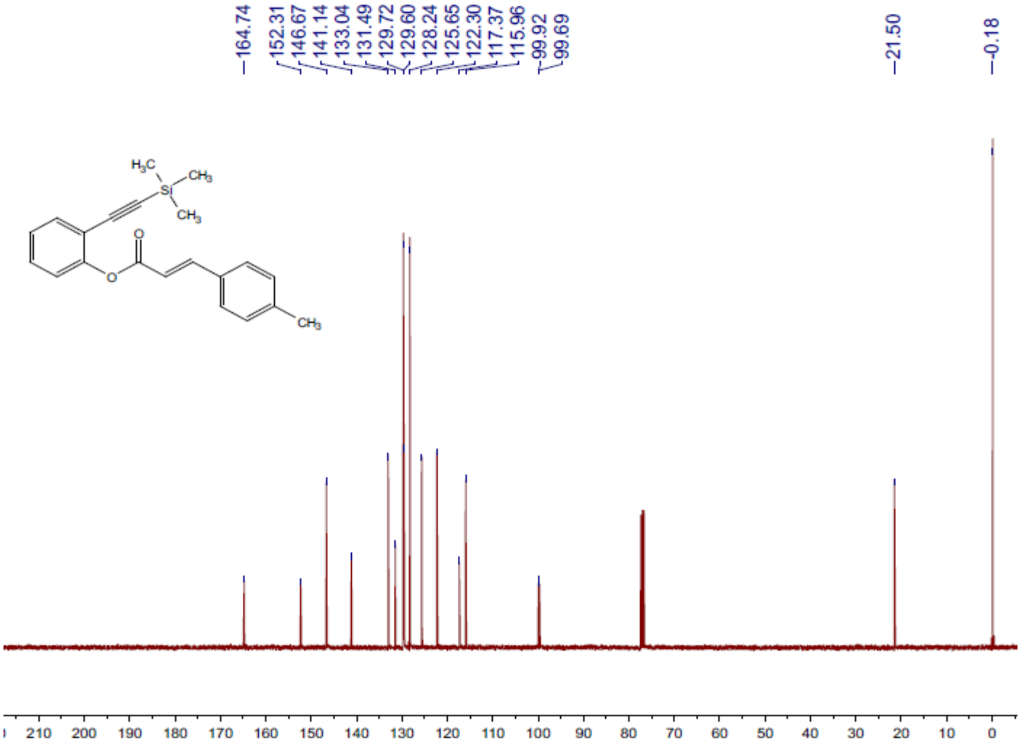

Supplementary Figure 277. ^{13}C NMR (100 MHz, $CDCl_3$) spectra of 2-((trimethylsilyl)ethynyl)phenyl (*E*)-3-(*p*-tolyl)acrylate (**4b**)

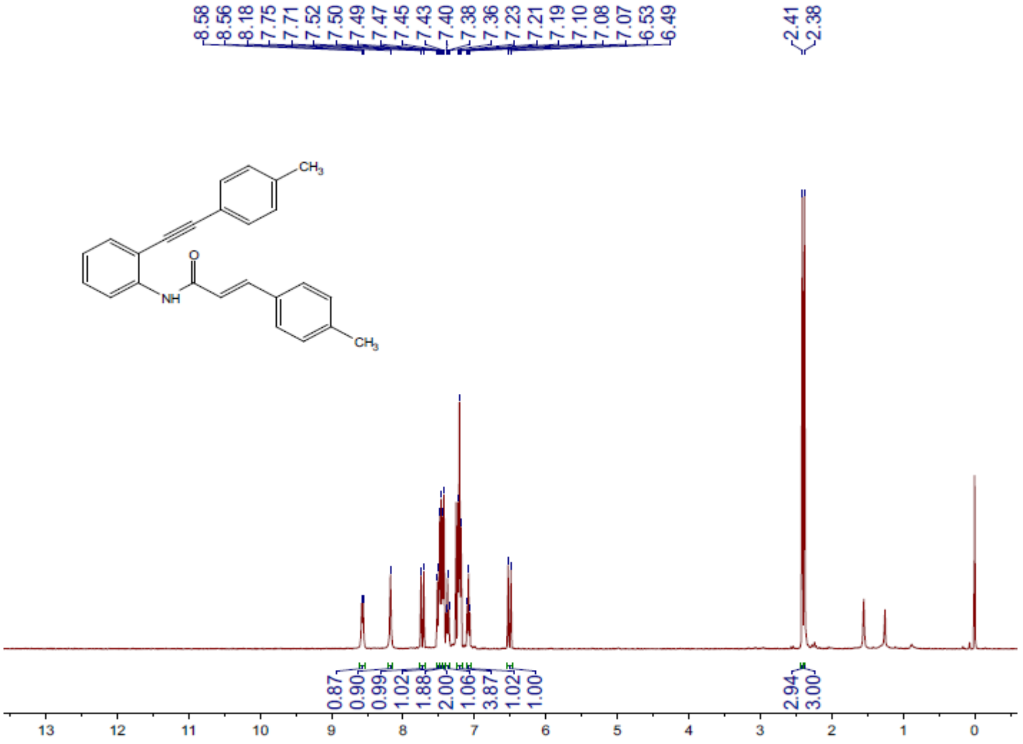

Supplementary Figure 278. ^{1}H NMR (400 MHz, $CDCl_3$) spectra of (*E*)-3-(*p*-tolyl)-*N*-(2-(*p*-tolylethynyl)phenyl)acrylamide (**4′c**)

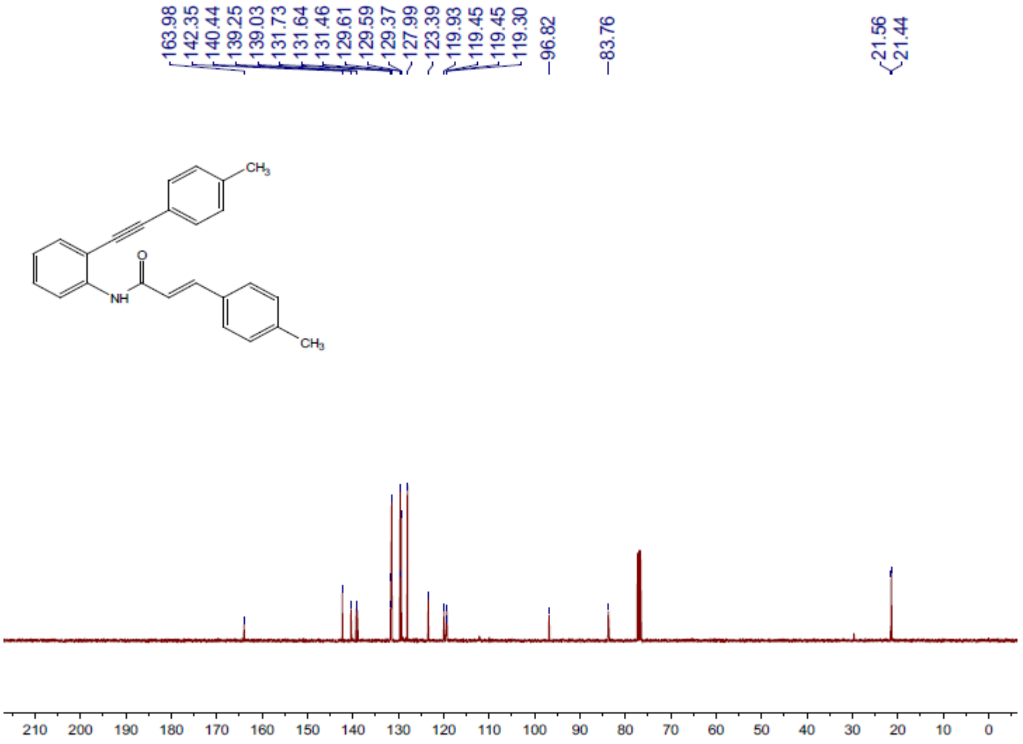

Supplementary Figure 279. ^{13}C NMR (100 MHz, $CDCl_3$) spectra of (*E*)-3-(*p*-tolyl)-*N*-(2-(*p*-tolylethynyl)phenyl)acrylamide (**4′c**)

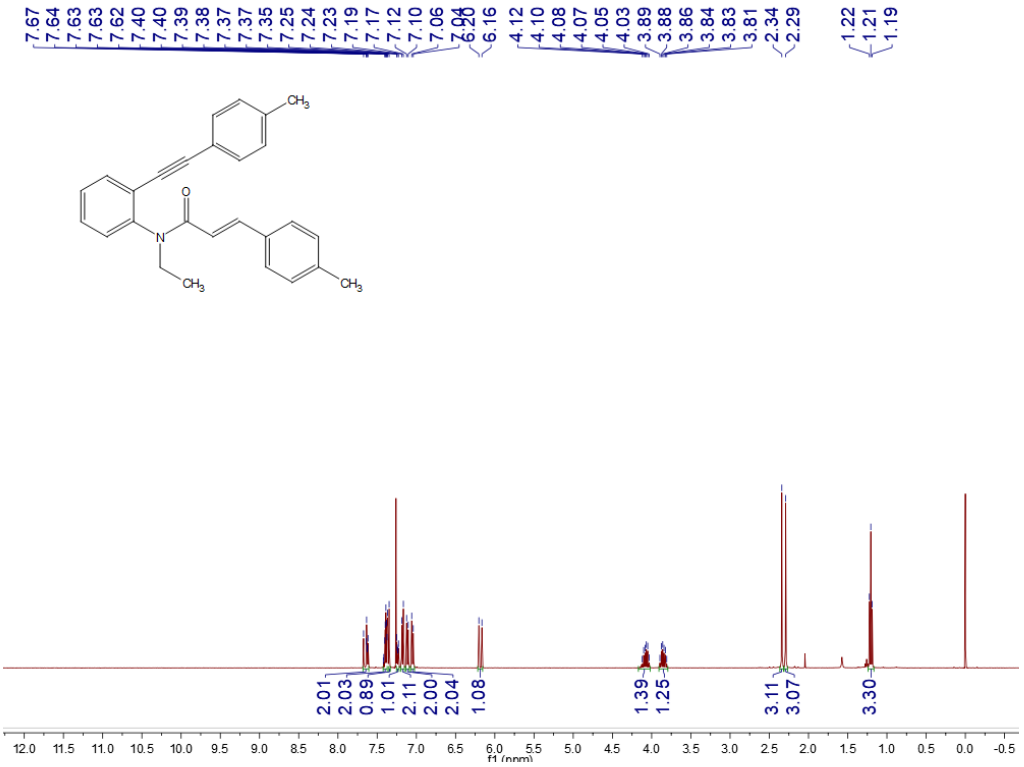

Supplementary Figure 280. ^{1}H NMR (400 MHz, $CDCl_3$) spectra of (*E*)-*N*-ethyl-3-(*p*-tolyl)-*N*-(2-(*p*-tolylethynyl)phenyl)acrylamide (**4c**)

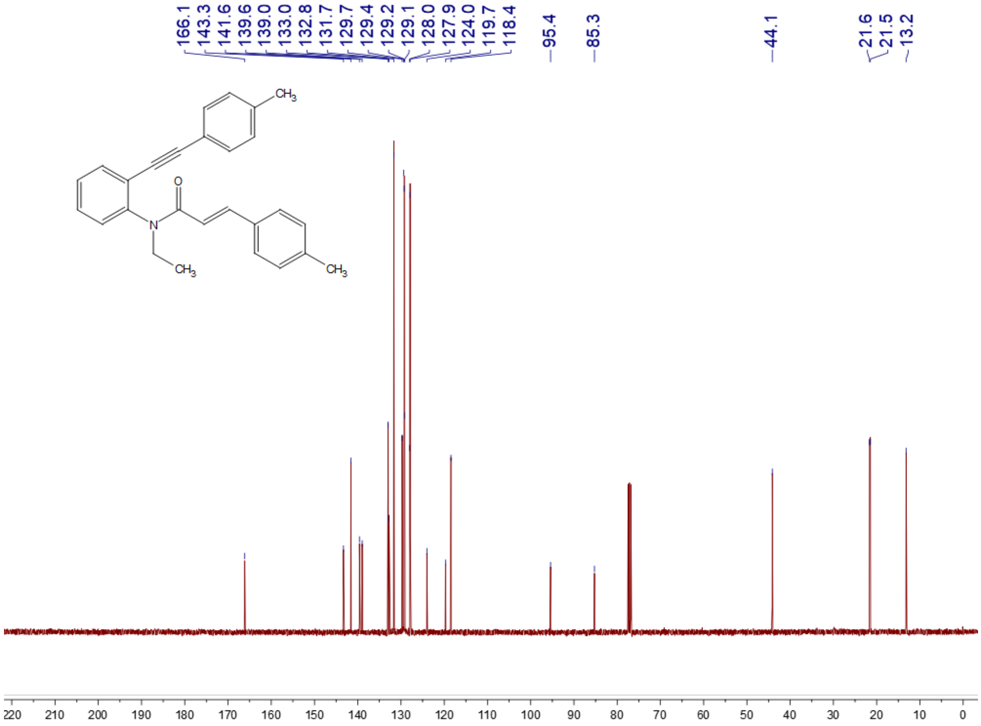

Supplementary Figure 281. ^{13}C NMR (100 MHz, $CDCl_3$) spectra of **(***E***)-***N*-ethyl**-**3**-(***p*-tolyl**)-***N*-(2**-(***p*-tolylethynyl**)**phenyl**)**acrylamide (**4c**)

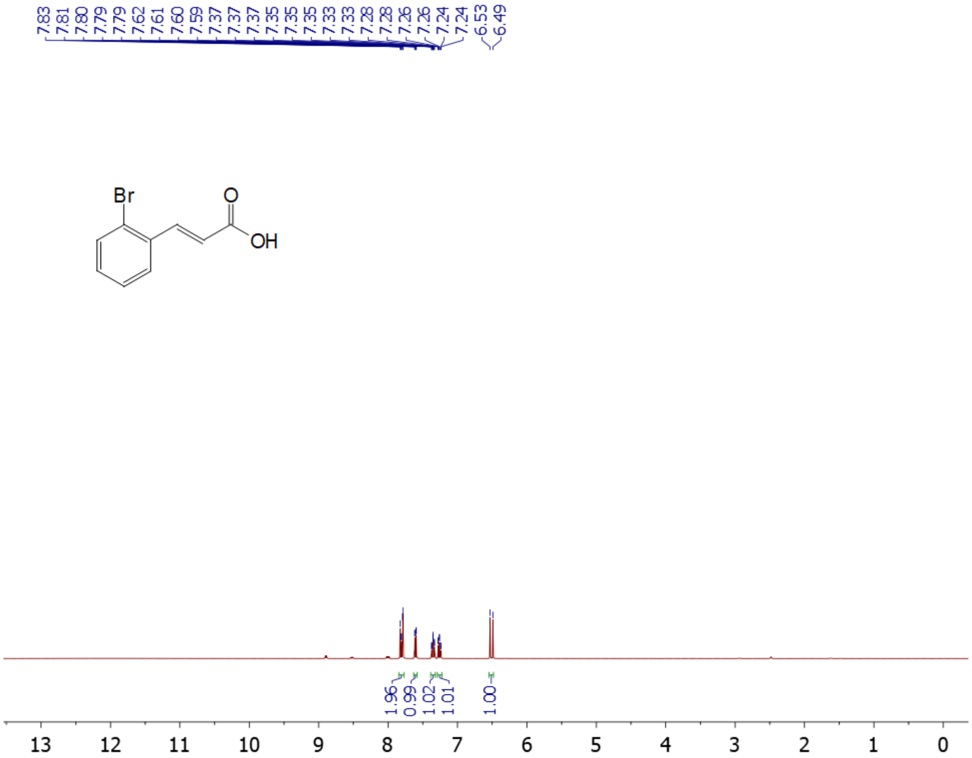

Supplementary Figure 282. ^{1}H NMR (400 MHz, DMSO-d$_6$) spectra of (*E*)-3-(2-bromophenyl)acrylic acid (**4′d**)

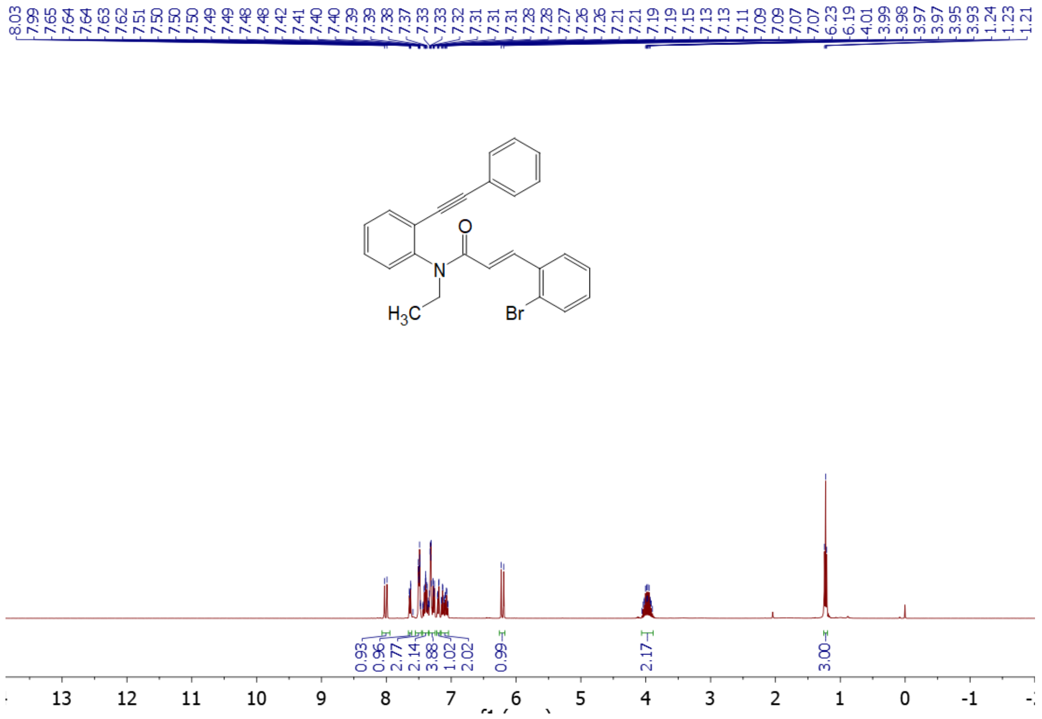

Supplementary Figure 283. ^{1}H NMR (400 MHz, $CDCl_3$) spectra of (*E*)-3-(2-bromophenyl)-*N*-ethyl-*N*-(2-(phenylethynyl)phenyl)acrylamide (**4d**)

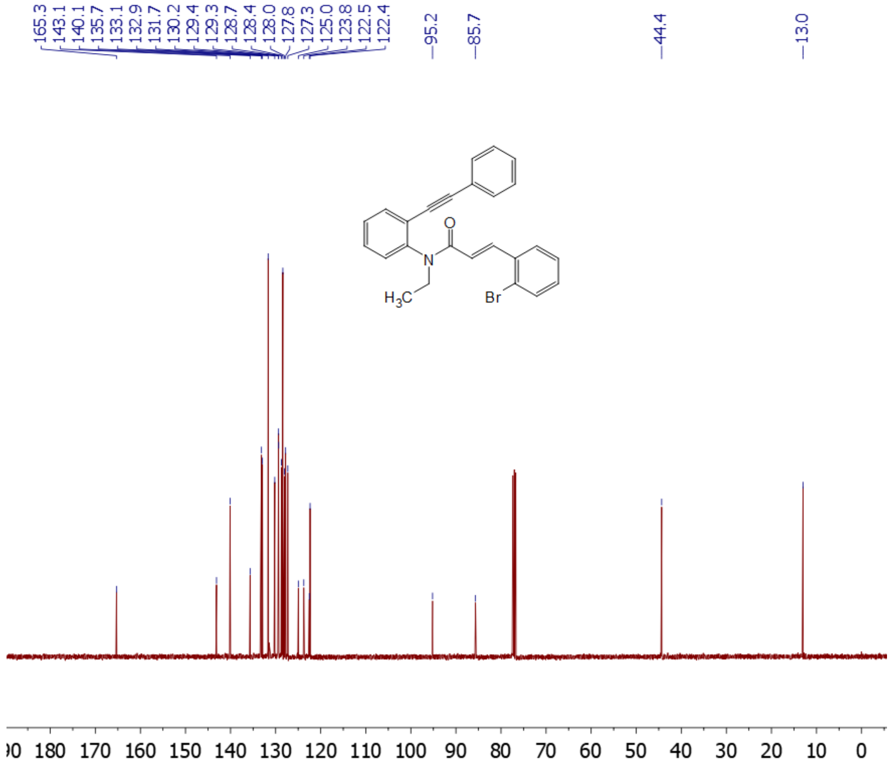

Supplementary Figure 284. ^{13}C NMR (100 MHz, $CDCl_3$) spectra of (*E*)-3-(2-bromophenyl)-*N*-ethyl-*N*-(2-(phenylethynyl)phenyl)acrylamide (**4d**)

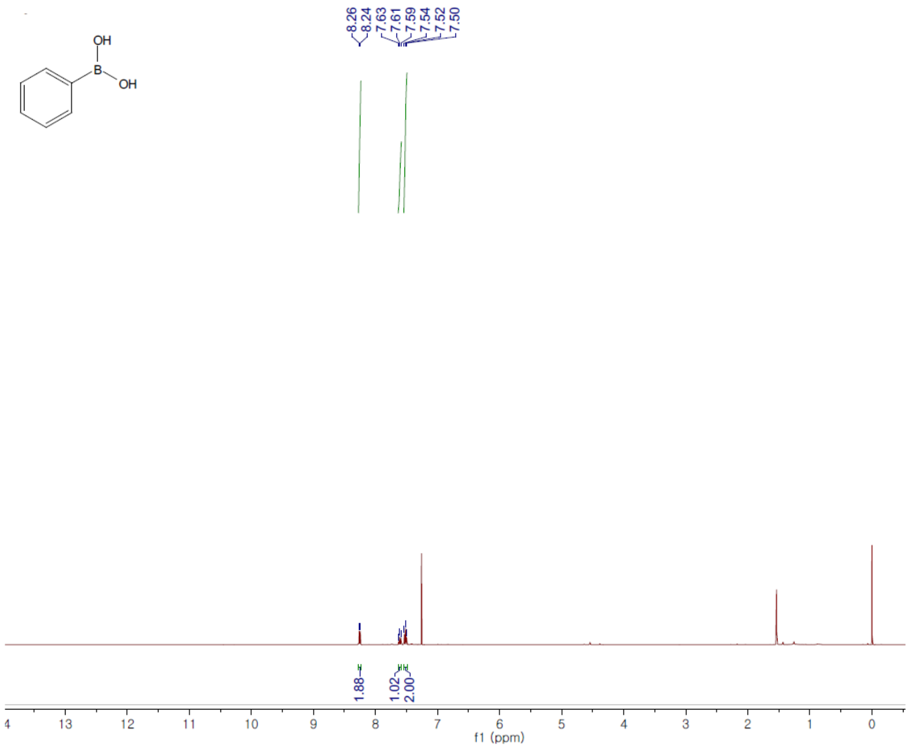

Supplementary Figure 285. ^{1}H NMR (400 MHz, $CDCl_3$) spectra of phenylboronic acid (**4′e**)

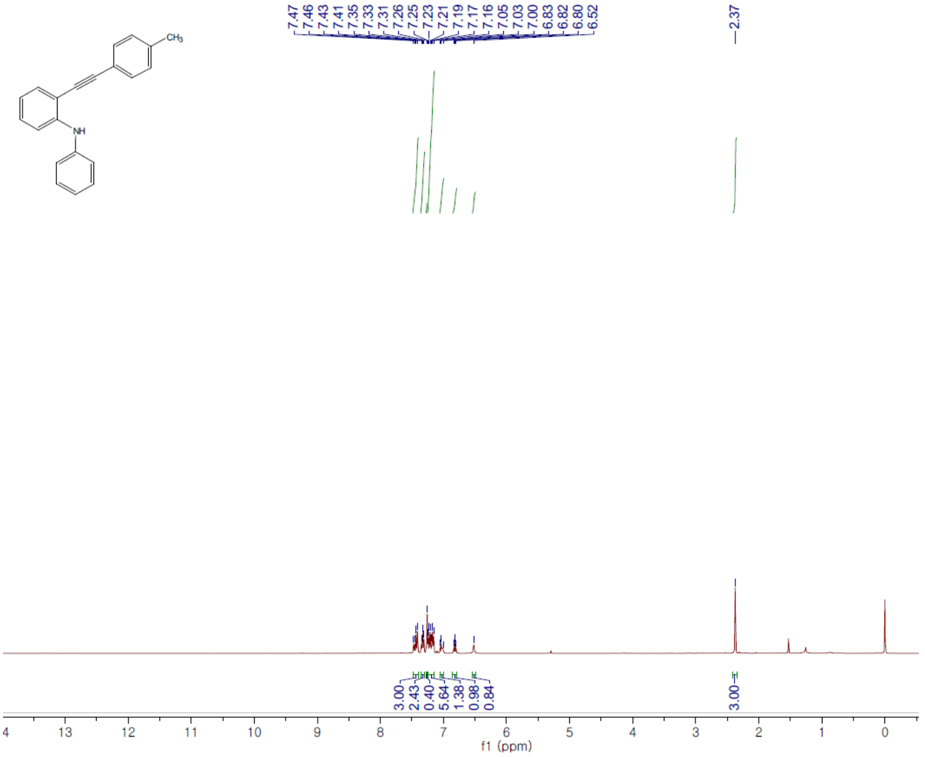

Supplementary Figure 286. ^{1}H NMR (400 MHz, $CDCl_3$) spectra of *N*-phenyl-2-(*p*-tolylethynyl)aniline (**4″e**)

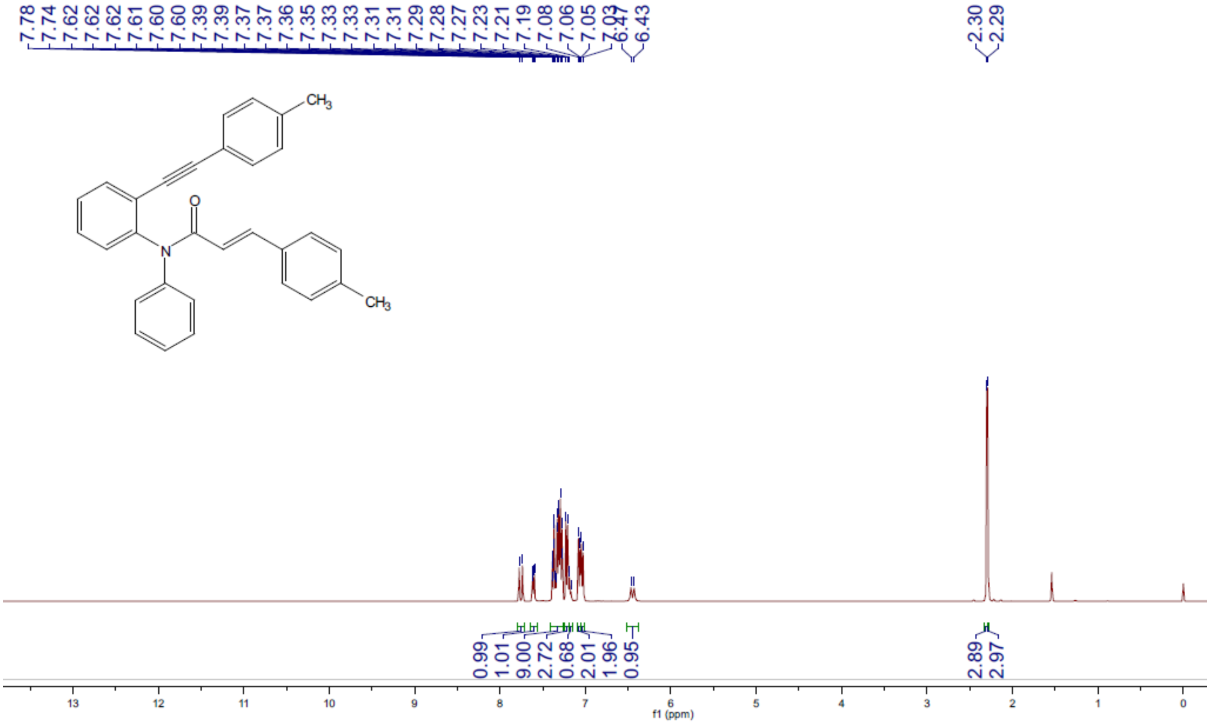

Supplementary Figure 287. ^{1}H NMR (400 MHz, $CDCl_3$) spectra of (*E*)-*N*-phenyl-3-(*p*-tolyl)-*N*-(2-(*p*-tolylethynyl)phenyl)acrylamide (**4e**)

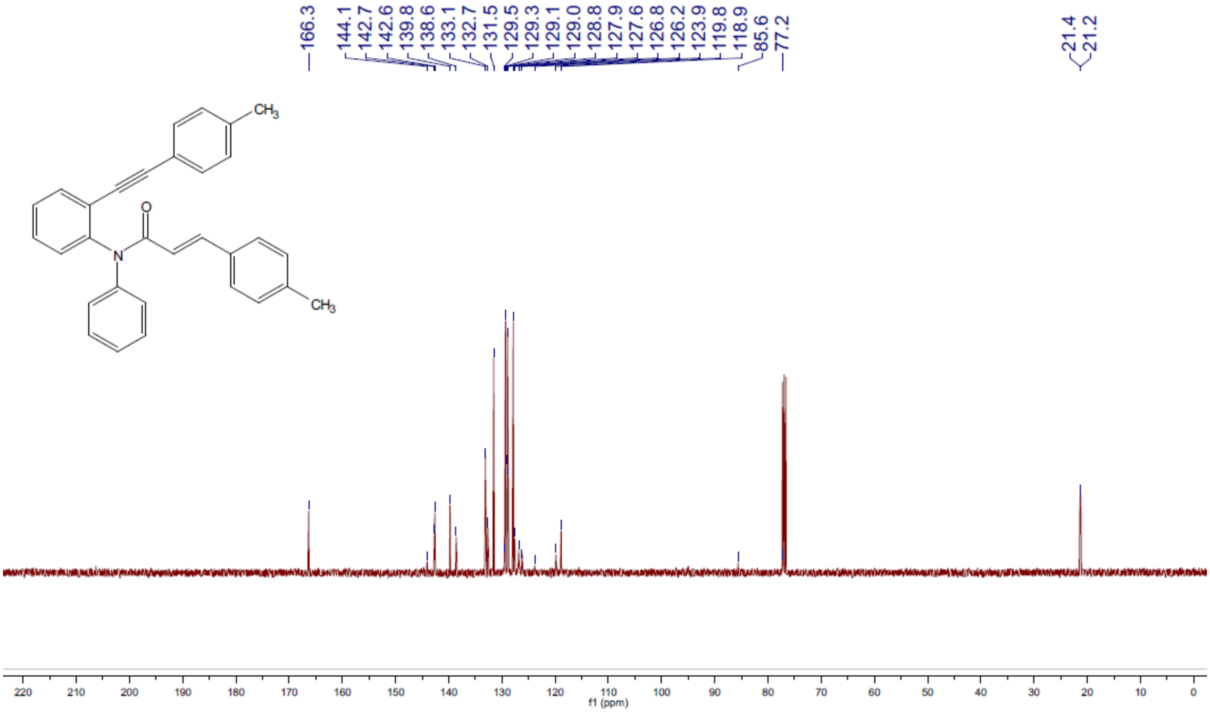

Supplementary Figure 288. ^{13}C NMR (100 MHz, $CDCl_3$) spectra of (*E*)-*N*-phenyl-3-(*p*-tolyl)-*N*-(2-(*p*-tolylethynyl)phenyl)acrylamide (**4e**)

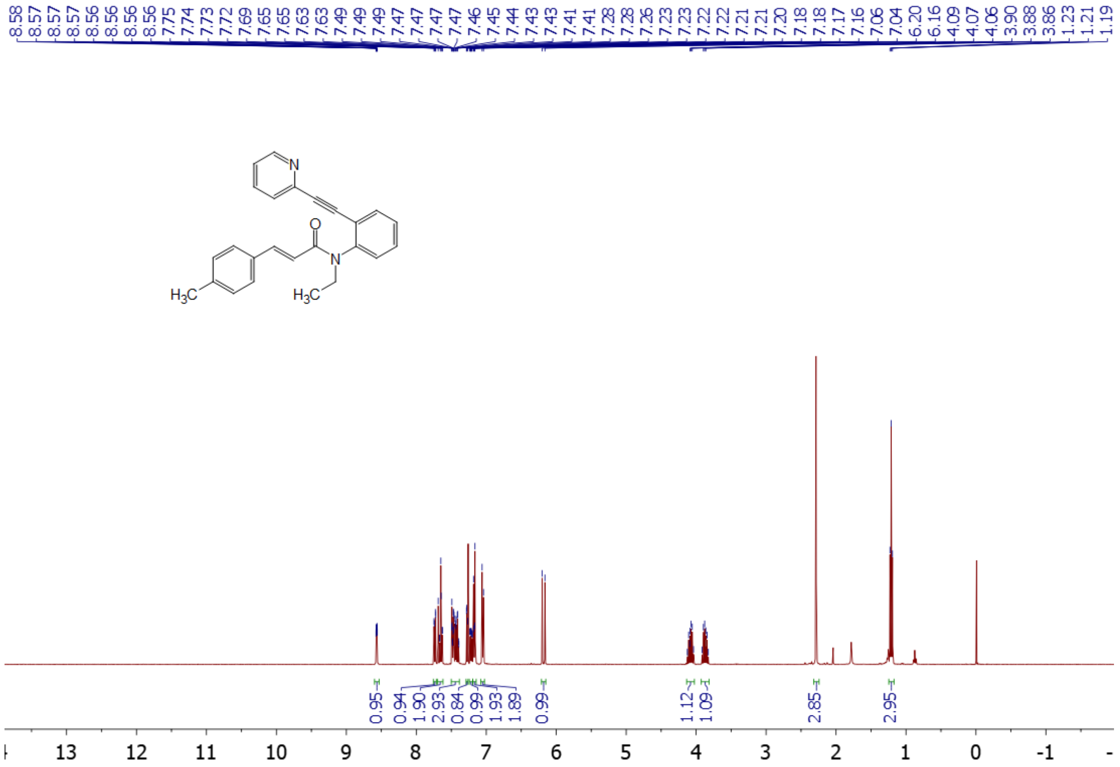

Supplementary Figure 289. ^{1}H NMR (400 MHz, $CDCl_3$) spectra of (*E*)-*N*-ethyl-*N*-(2-(pyridin-2-ylethynyl)phenyl)-3-(*p*-tolyl)acrylamide (**4f**)

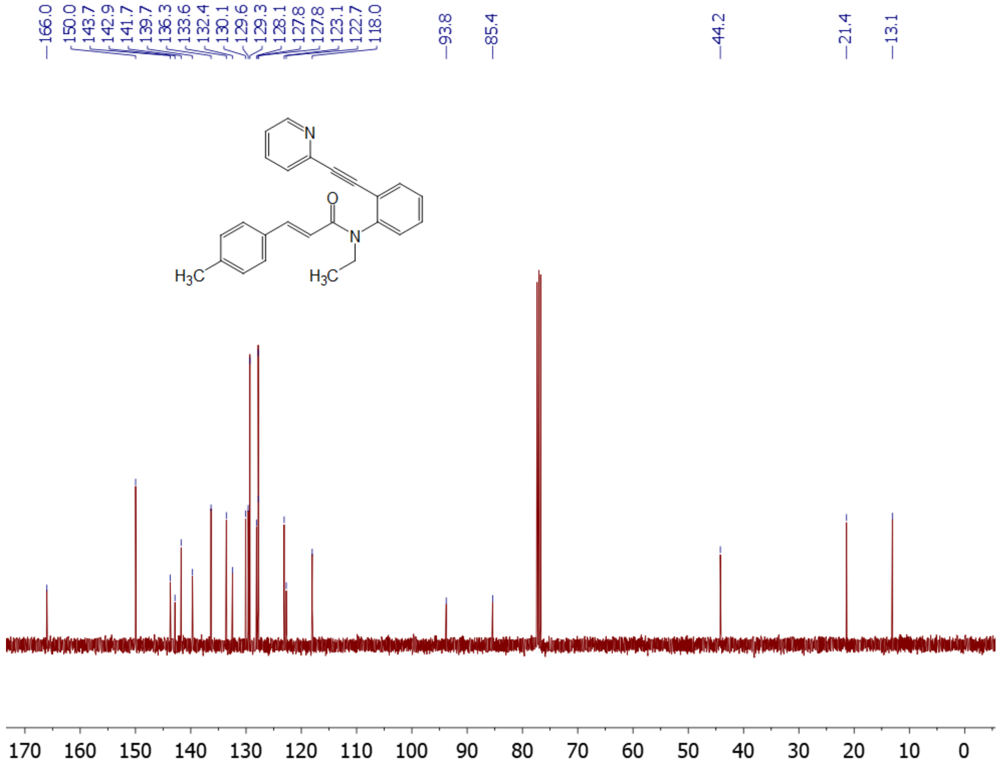

Supplementary Figure 290. ^{13}C NMR (100 MHz, $CDCl_3$) spectra of (*E*)-*N*-ethyl-*N*-(2-(pyridin-2-ylethynyl)phenyl)-3-(*p*-tolyl)acrylamide (**4f**)

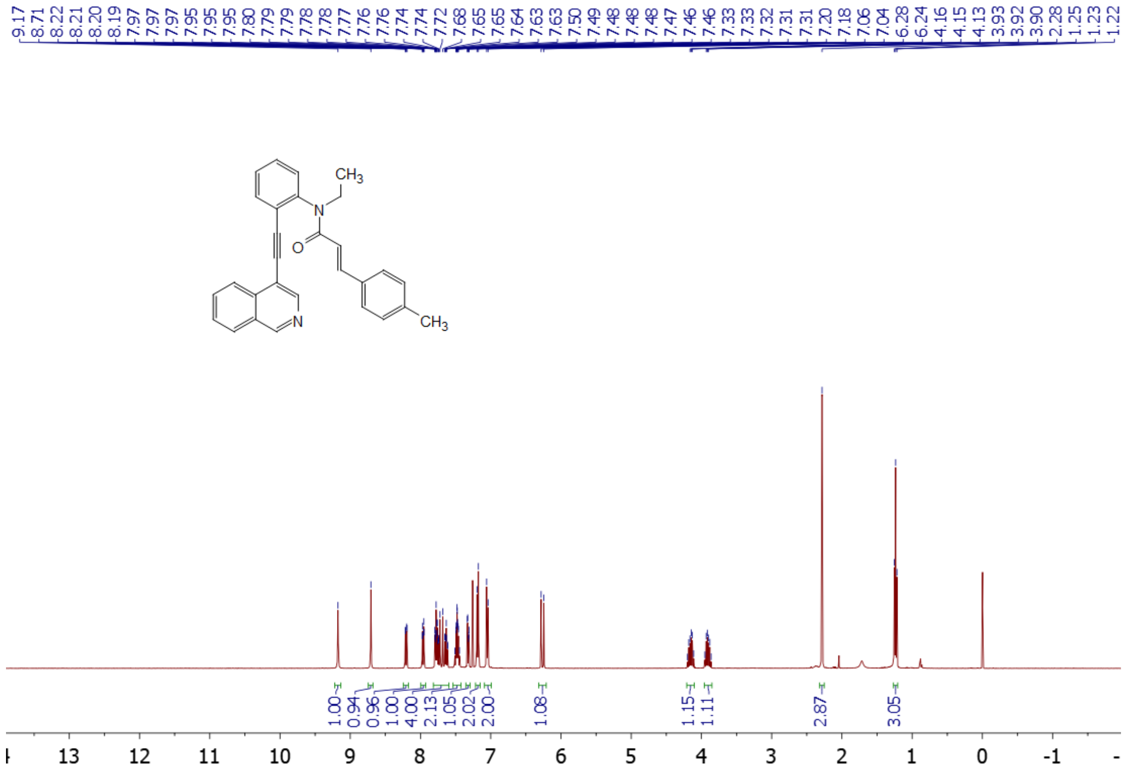

Supplementary Figure 291. ^{1}H NMR (400 MHz, $CDCl_3$) spectra of *(E)*-*N*-ethyl-*N*-(2-(isoquinolin-4-ylethynyl)phenyl)-3-(*p*-tolyl)acrylamide (**4g**)

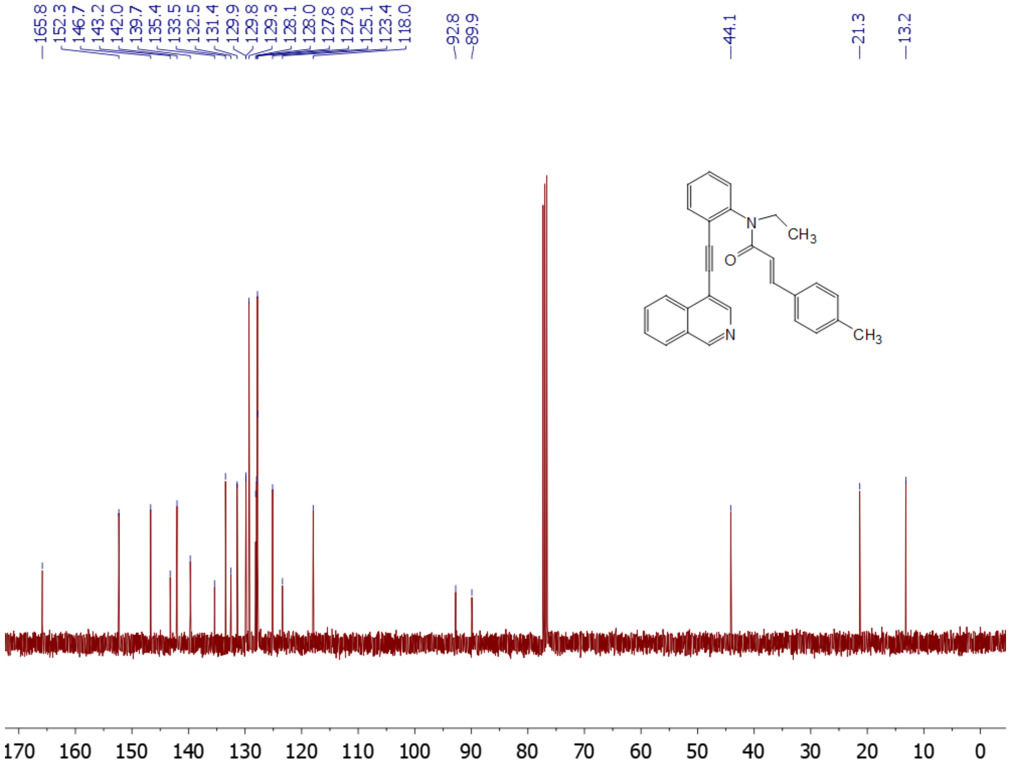

Supplementary Figure 292. ^{13}C NMR (100 MHz, $CDCl_3$) spectra of *(E)*-*N*-ethyl-*N*-(2-(isoquinolin-4-ylethynyl)phenyl)-3-(*p*-tolyl)acrylamide (**4g**)

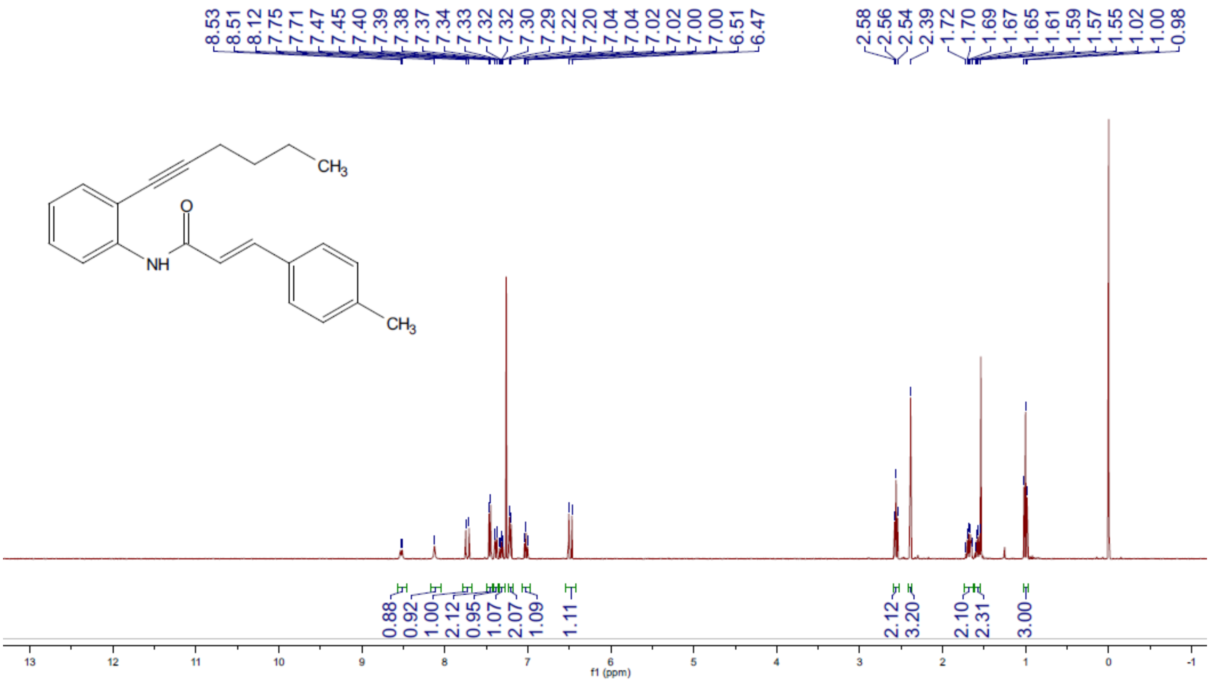

Supplementary Figure 293. ^{1}H NMR (400 MHz, $CDCl_3$) spectra of (*E*)-*N*-(2-(hex-1-yn-1-yl)phenyl)-3-(*p*-tolyl)acrylamide (**4′h**)

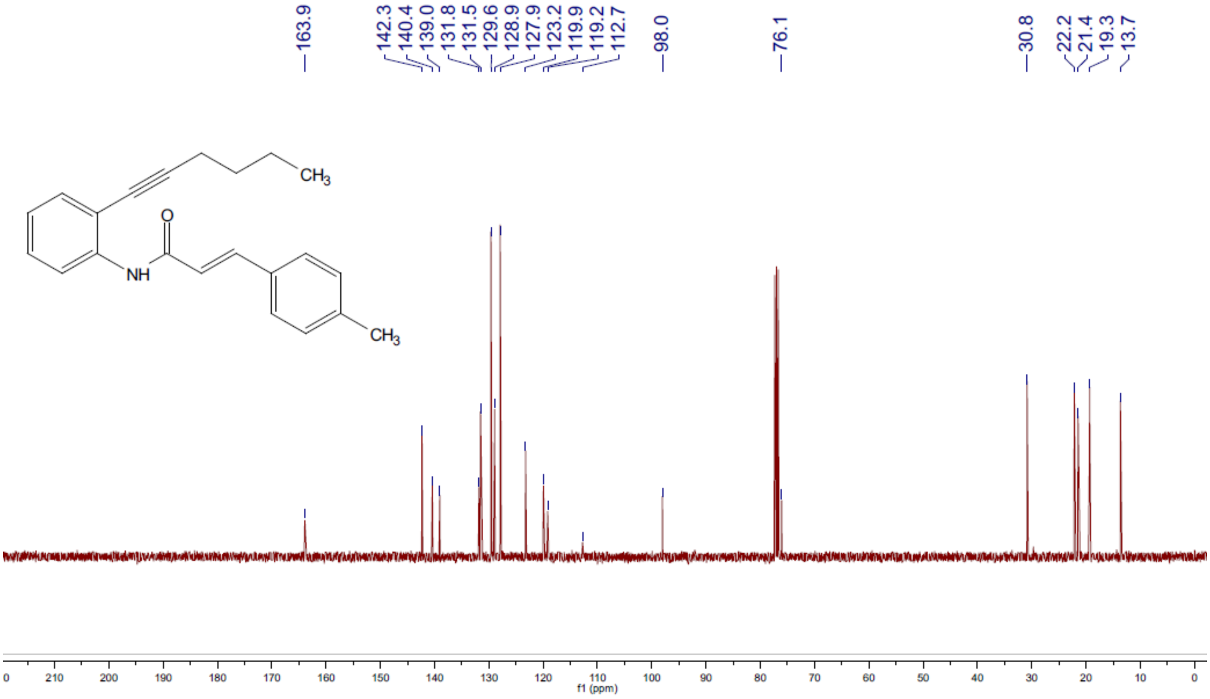

Supplementary Figure 294. ^{13}C NMR (100 MHz, $CDCl_3$) spectra of (*E*)-*N*-(2-(hex-1-yn-1-yl)phenyl)-3-(*p*-tolyl)acrylamide (**4′h**)

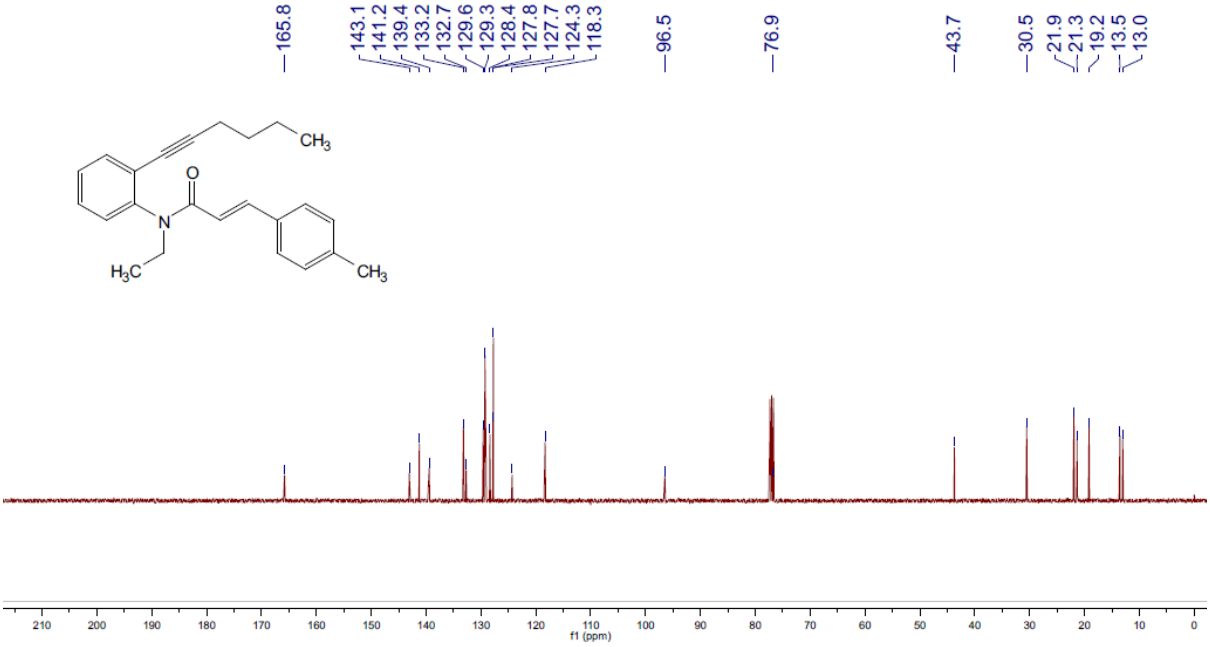

Supplementary Figure 295. ^{1}H NMR (400 MHz, $CDCl_3$) spectra of (*E*)-*N*-ethyl-*N*-(2-(hex-1-yn-1-yl)phenyl)-3-(*p*-tolyl)acrylamide (**4h**)

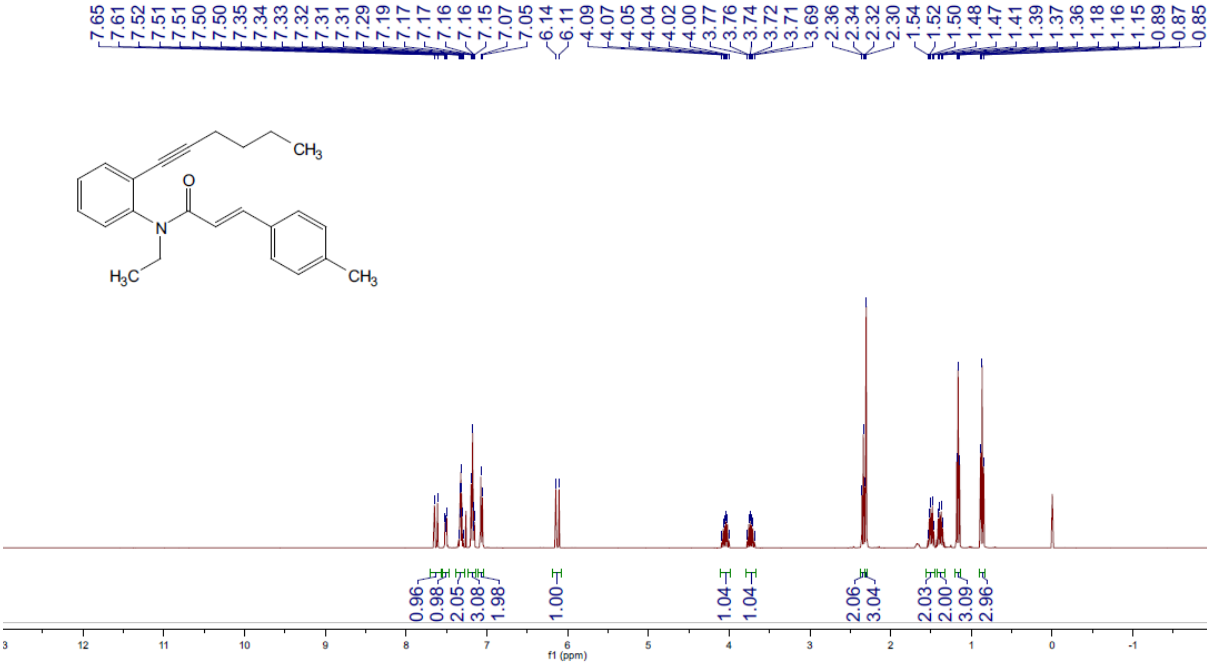

Supplementary Figure 296. ^{13}C NMR (100 MHz, $CDCl_3$) spectra of (*E*)-*N*-ethyl-*N*-(2-(hex-1-yn-1-yl)phenyl)-3-(*p*-tolyl)acrylamide (**4h**)

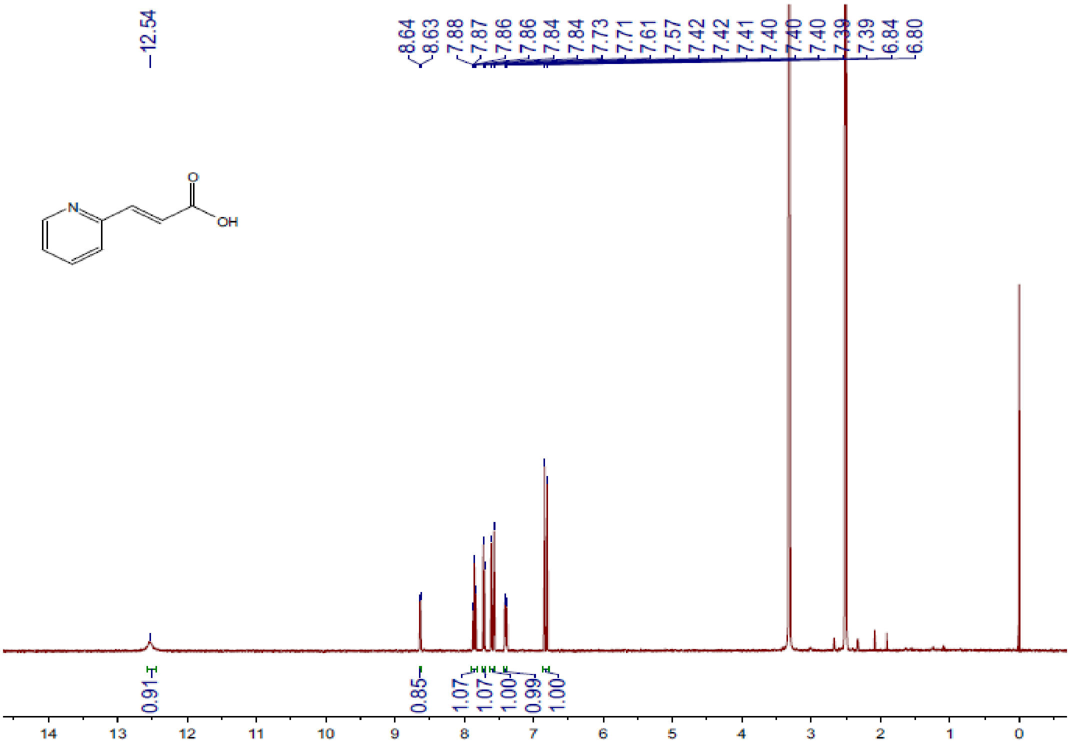

Supplementary Figure 297. ^{1}H NMR (400 MHz, DMSO-d$_6$) spectra of (*E*)-3-(pyridin-2-yl)acrylic acid (**4′i**)

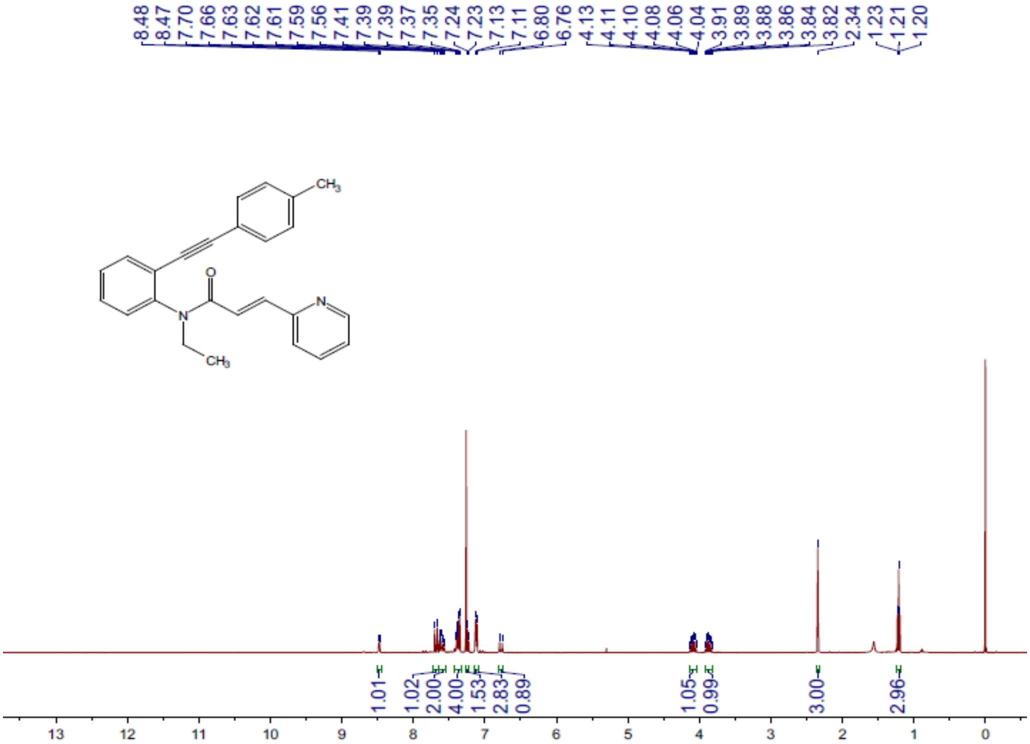

Supplementary Figure 298. ^{1}H NMR (400 MHz, $CDCl_3$) spectra of (*E*)-*N*-ethyl-3-(pyridin-2-yl)-*N*-(2-(*p*-tolylethynyl)phenyl)acrylamide (**4i**)

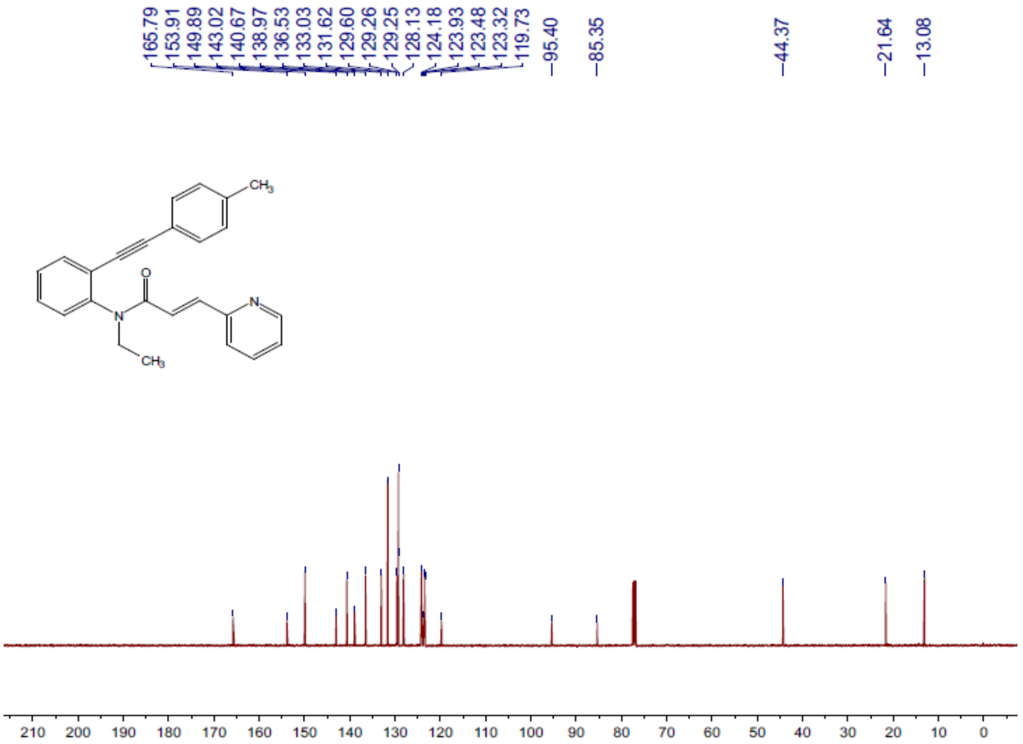

Supplementary Figure 299. ^{13}C NMR (100 MHz, $CDCl_3$) spectra of (*E*)-*N*-ethyl-3-(pyridin-2-yl)-*N*-(2-(*p*-tolylethynyl)phenyl)acrylamide (**4i**)

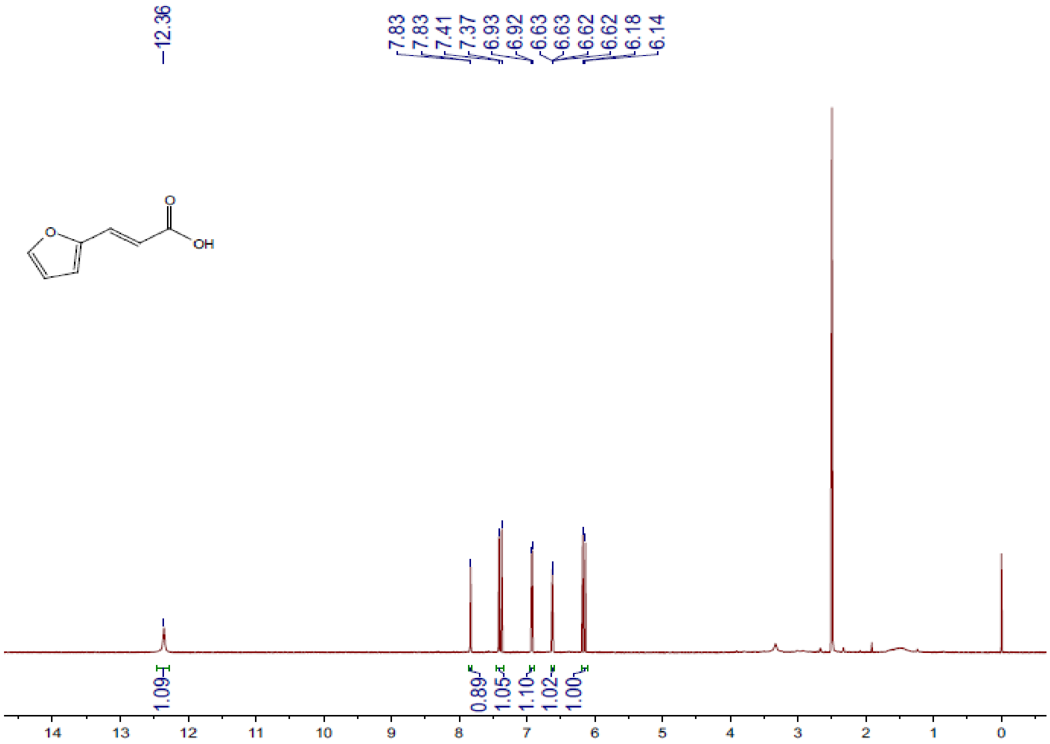

Supplementary Figure 300. ^{1}H NMR (400 MHz, $CDCl_3$) spectra of (*E*)-3-(furan-2-yl)acrylic acid (**4′j**)

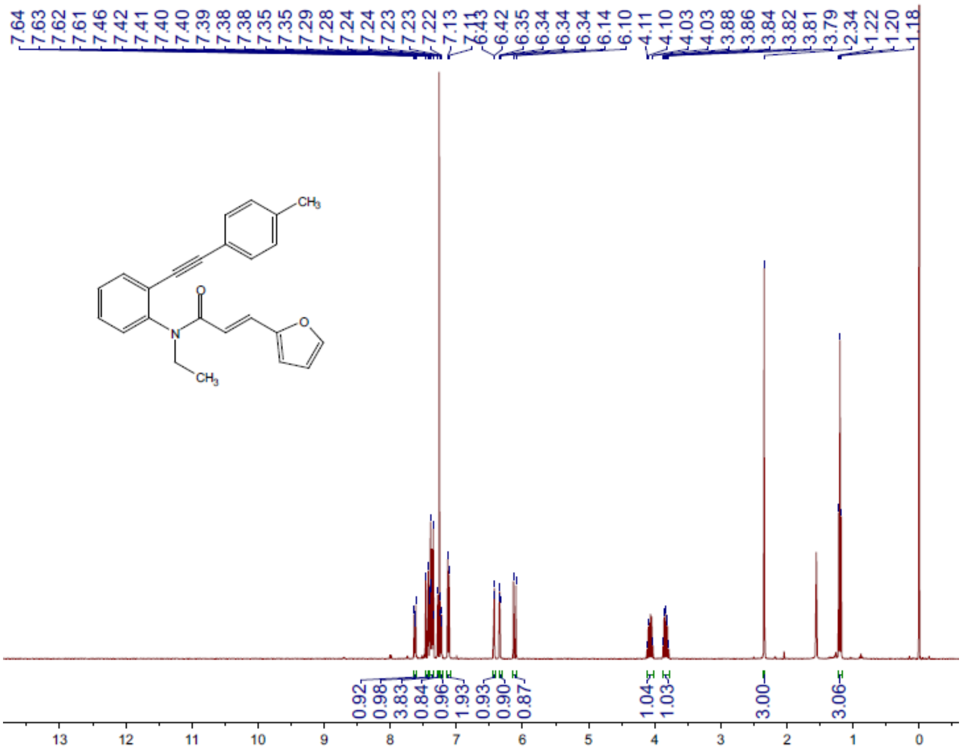

Supplementary Figure 301. ^{1}H NMR (400 MHz, $CDCl_3$) spectra of (*E*)-*N*-ethyl-3-(furan-2-yl)-*N*-(2-(*p*-tolylethynyl)phenyl)acrylamide (**4j**)

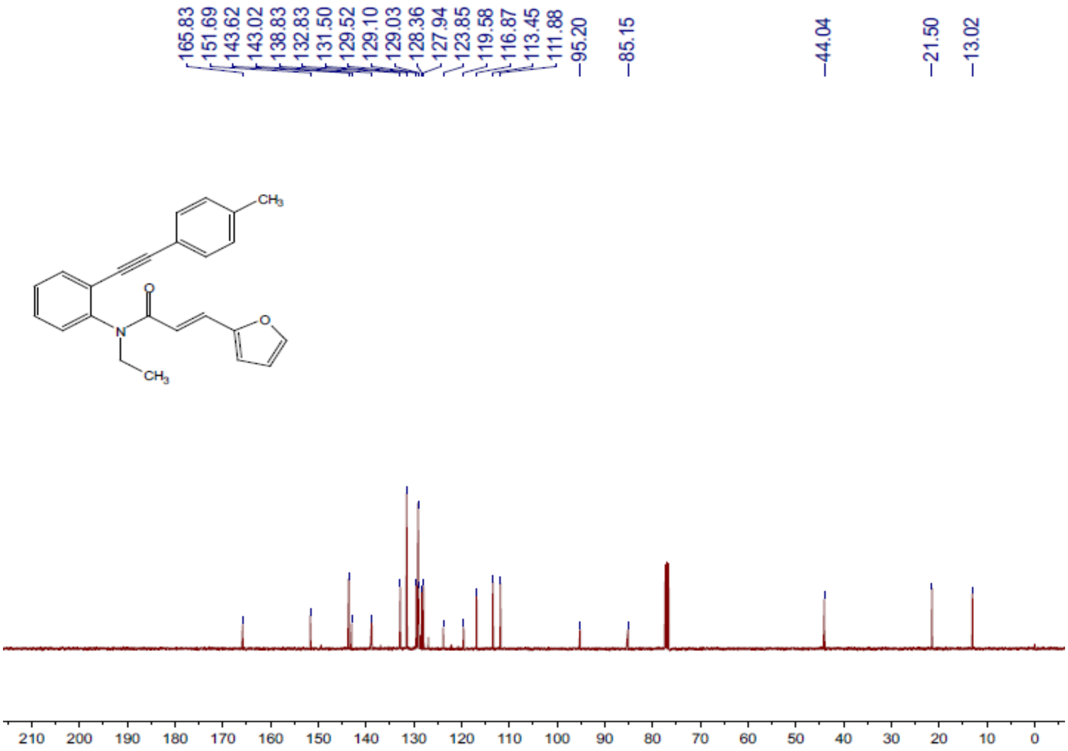

Supplementary Figure 302. ^{13}C NMR (100 MHz, $CDCl_3$) spectra of (*E*)-*N*-ethyl-3-(furan-2-yl)-*N*-(2-(*p*-tolylethynyl)phenyl)acrylamide (**4j**)

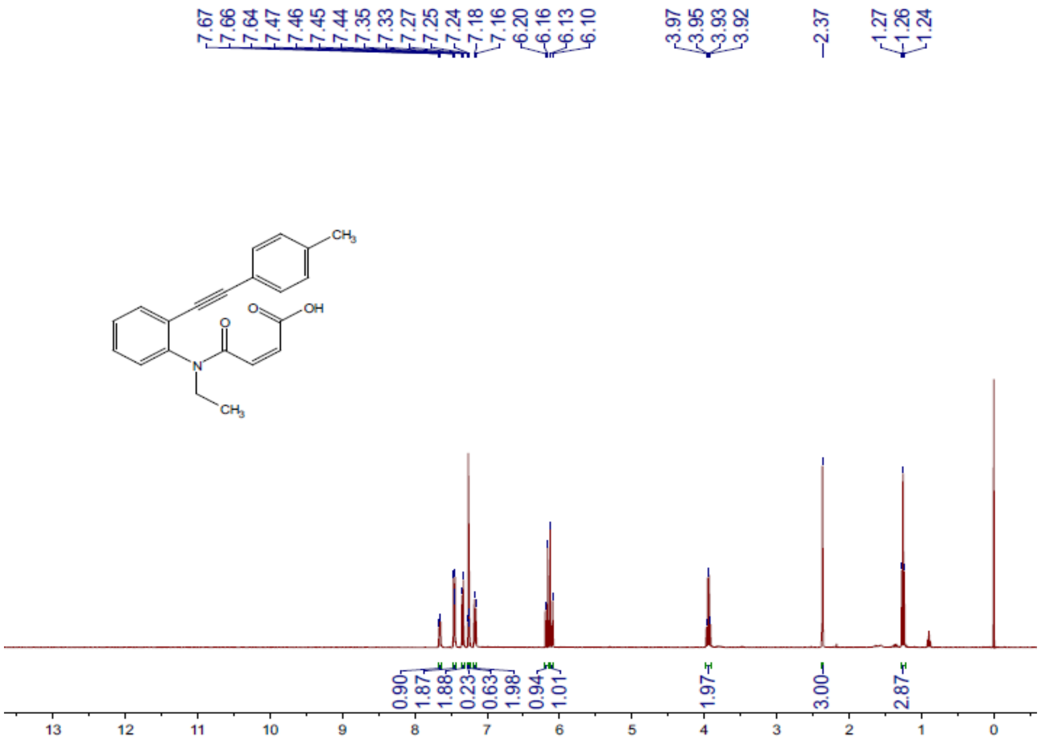

Supplementary Figure 303. ^{1}H NMR (400 MHz, $CDCl_3$) spectra of (*Z*)-4-(ethyl(2-(*p*-tolylethynyl)phenyl)amino)-4-oxobut-2-enoic acid (**4′k**)

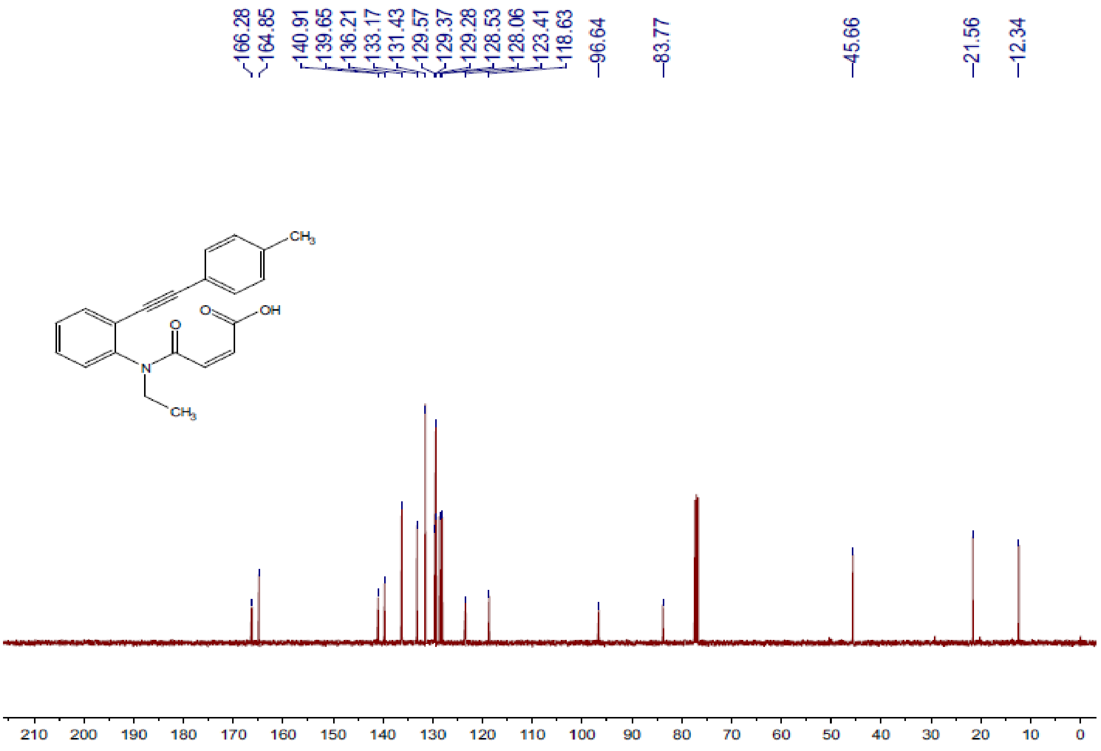

Supplementary Figure 304. ^{13}C NMR (100 MHz, $CDCl_3$) spectra of (*Z*)-4-(ethyl(2-(*p*-tolylethynyl)phenyl)amino)-4-oxobut-2-enoic acid (**4′k**)

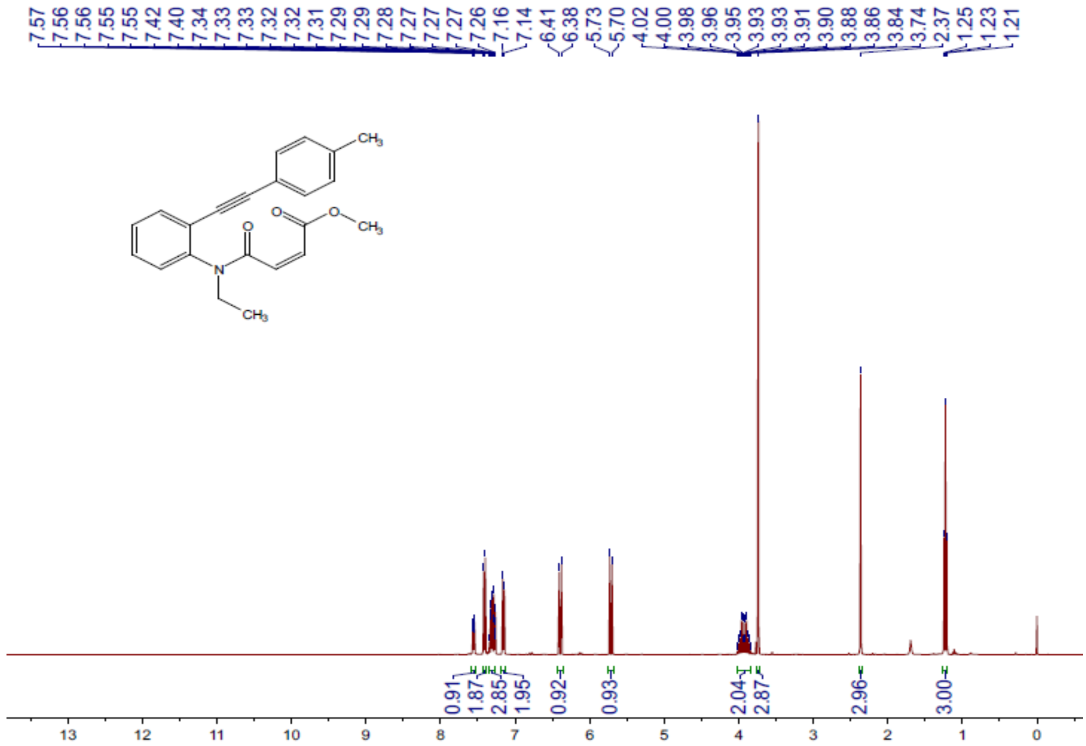

Supplementary Figure 305. ^{1}H NMR (400 MHz, $CDCl_3$) spectra of methyl (*Z*)-4-(ethyl(2-(*p*-tolylethynyl)phenyl)amino)-4-oxobut-2-enoate (**4k**)

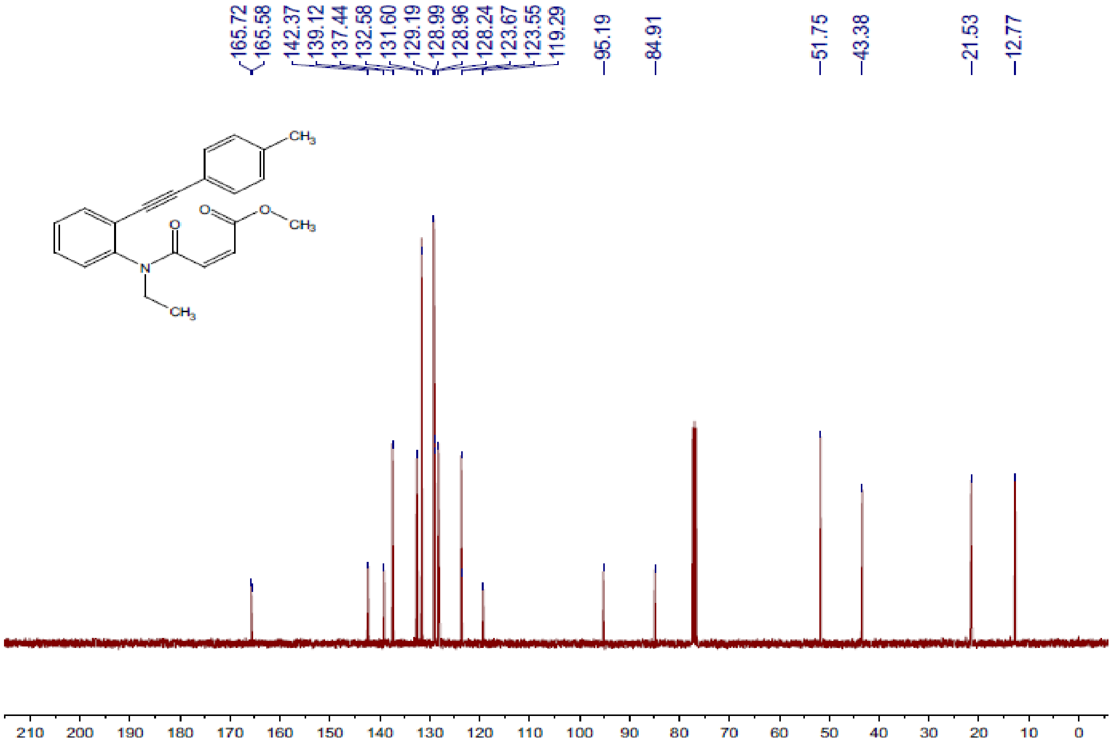

Supplementary Figure 306. ^{13}C NMR (100 MHz, $CDCl_3$) spectra of methyl (*Z*)-4-(ethyl(2-(*p*-tolylethynyl)phenyl)amino)-4-oxobut-2-enoate (**4k**)

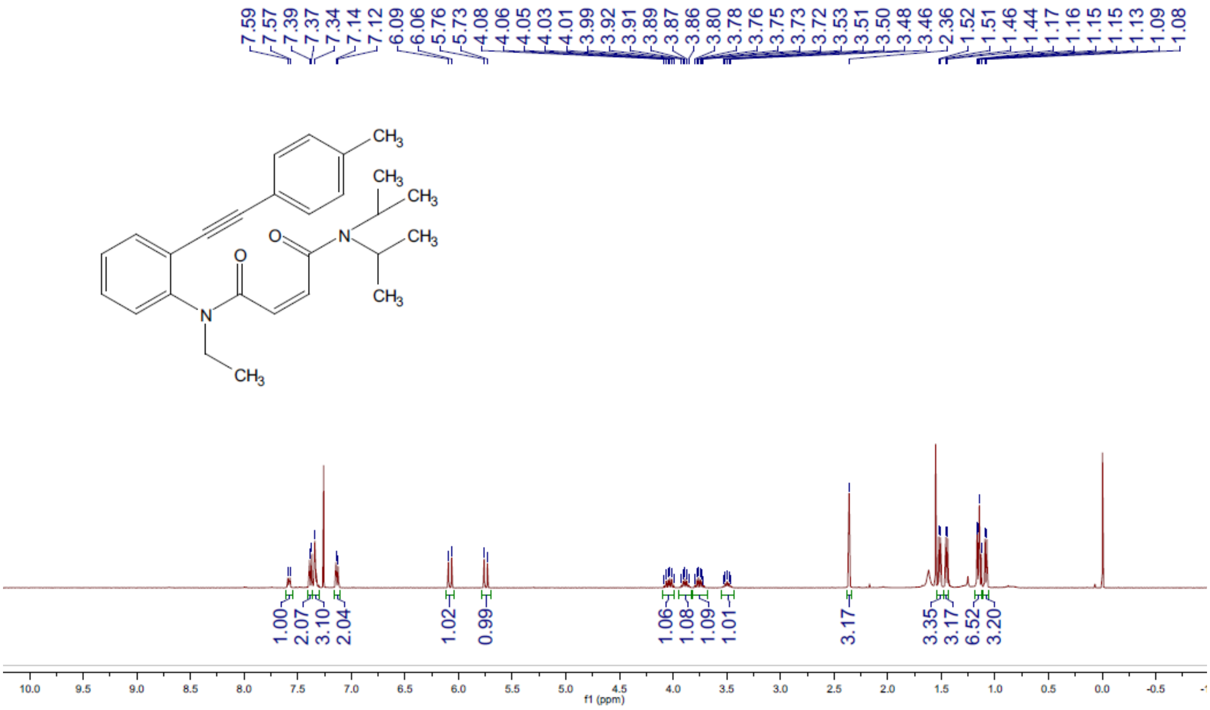

Supplementary Figure 307. ^{1}H NMR (400 MHz, $CDCl_3$) spectra of *N*^{1}-ethyl-*N*^{4},*N*^{4}-diisopropyl-*N*^{1}-(2-(*p*-tolylethynyl)phenyl)maleamide (**4l**)

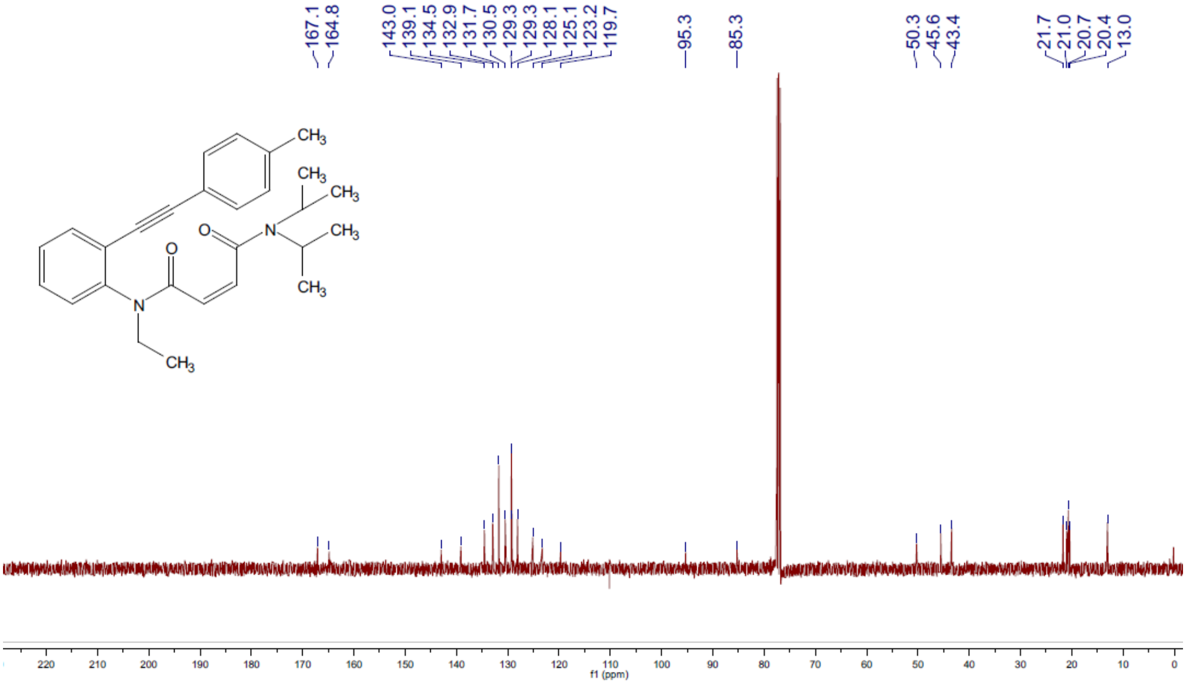

Supplementary Figure 308. ^{13}C NMR (100 MHz, $CDCl_3$) spectra of *N*^{1}-ethyl-*N*^{4},*N*^{4}-diisopropyl-*N*^{1}-(2-(*p*-tolylethynyl)phenyl)maleamide (**4l**)

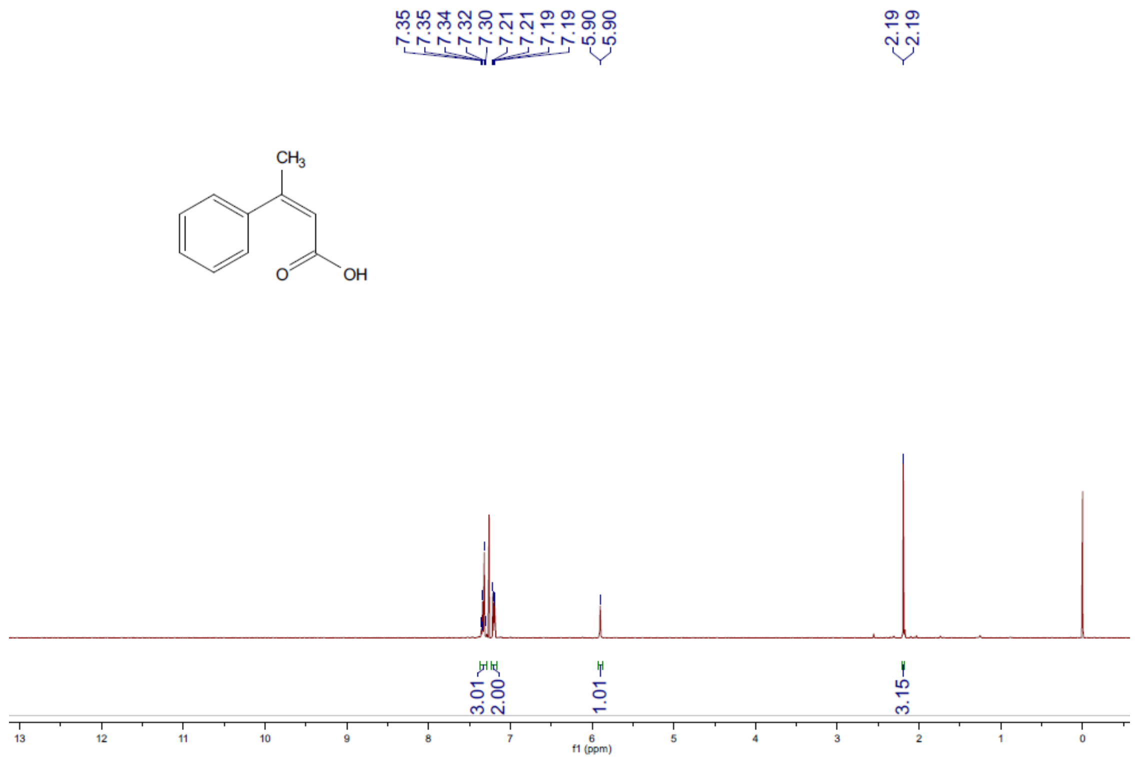

Supplementary Figure 309. ^{1}H NMR (400 MHz, $CDCl_3$) spectra of (*Z*)-3-phenylbut-2-enoic acid (**4′m**)

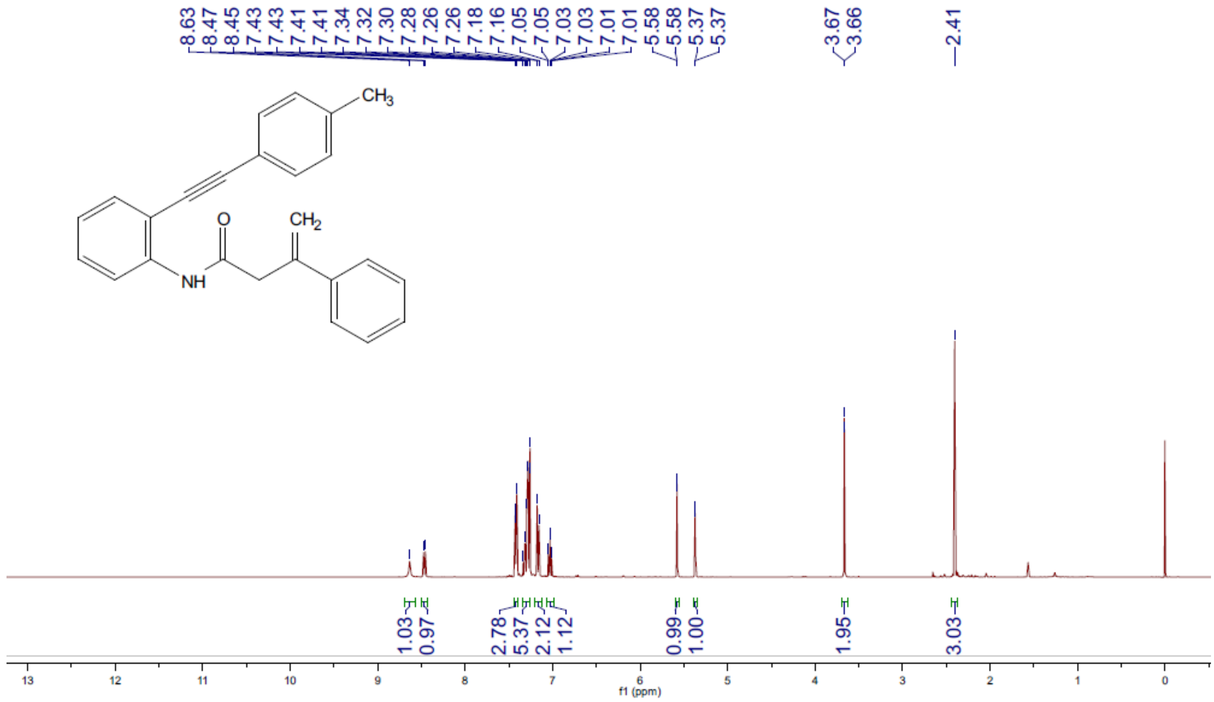

Supplementary Figure 310. ^{1}H NMR (400 MHz, $CDCl_3$) spectra of 3-phenyl-*N*-(2-(*p*-tolylethynyl)phenyl)but-3-enamide (**4″m**)

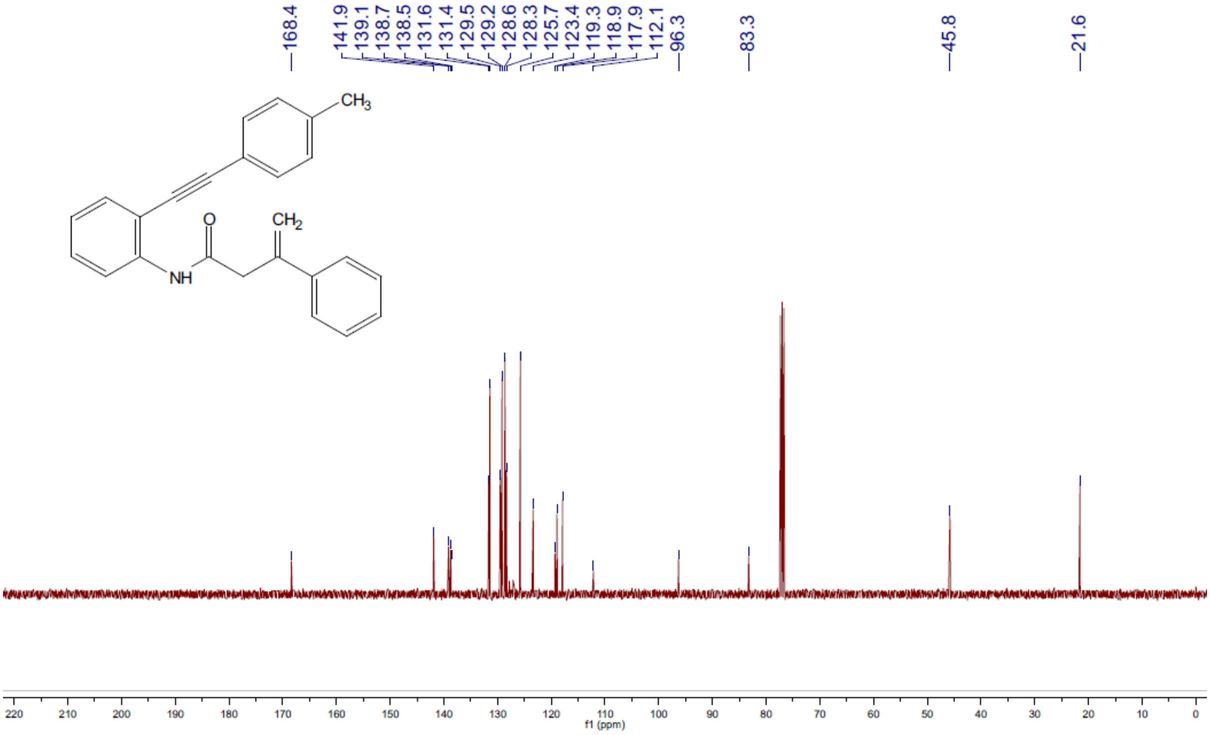

Supplementary Figure 311. ^{13}C NMR (100 MHz, $CDCl_3$) spectra of 3-phenyl-*N*-(2-(*p*-tolylethynyl)phenyl)but-3-enamide (**4″m**)

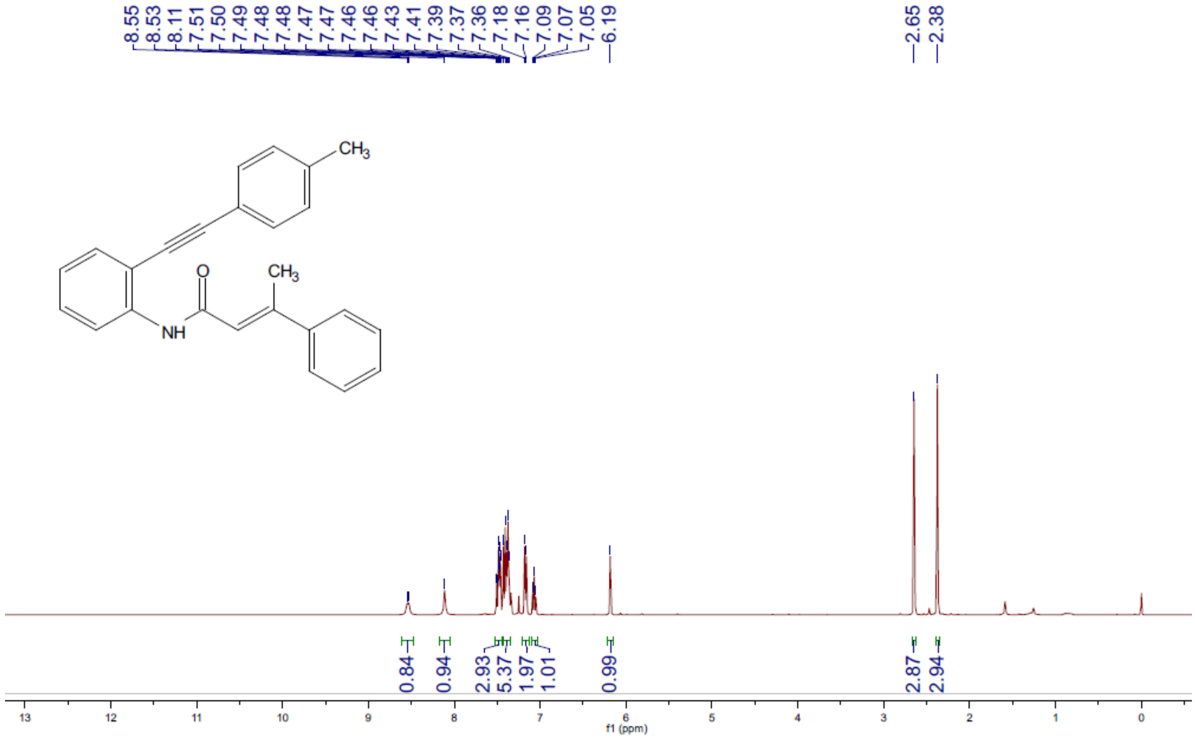

Supplementary Figure 312. ^{1}H NMR (400 MHz, $CDCl_3$) spectra of 3-phenyl-*N*-(*2*-(*p*-tolylethynyl)phenyl)but-2-enamide (**4‴m**)

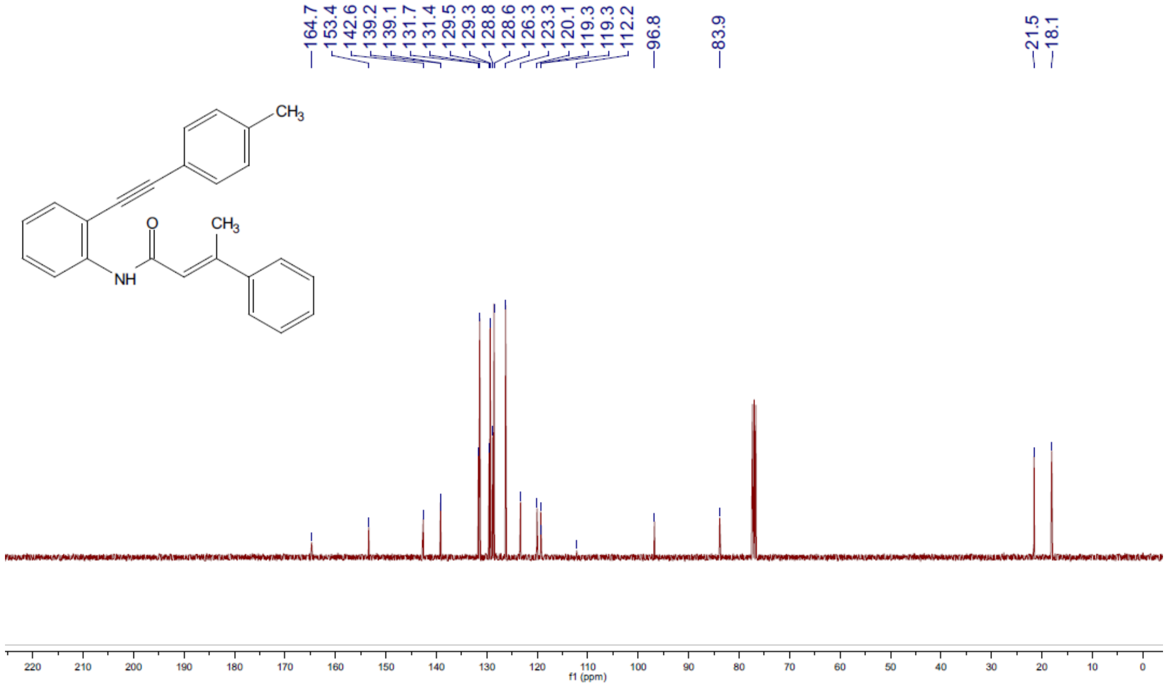

Supplementary Figure 313. ^{13}C NMR (100 MHz, $CDCl_3$) spectra of 3-phenyl-*N*-(*2*-(*p*-tolylethynyl)phenyl)but-2-enamide (**4′′′m**)

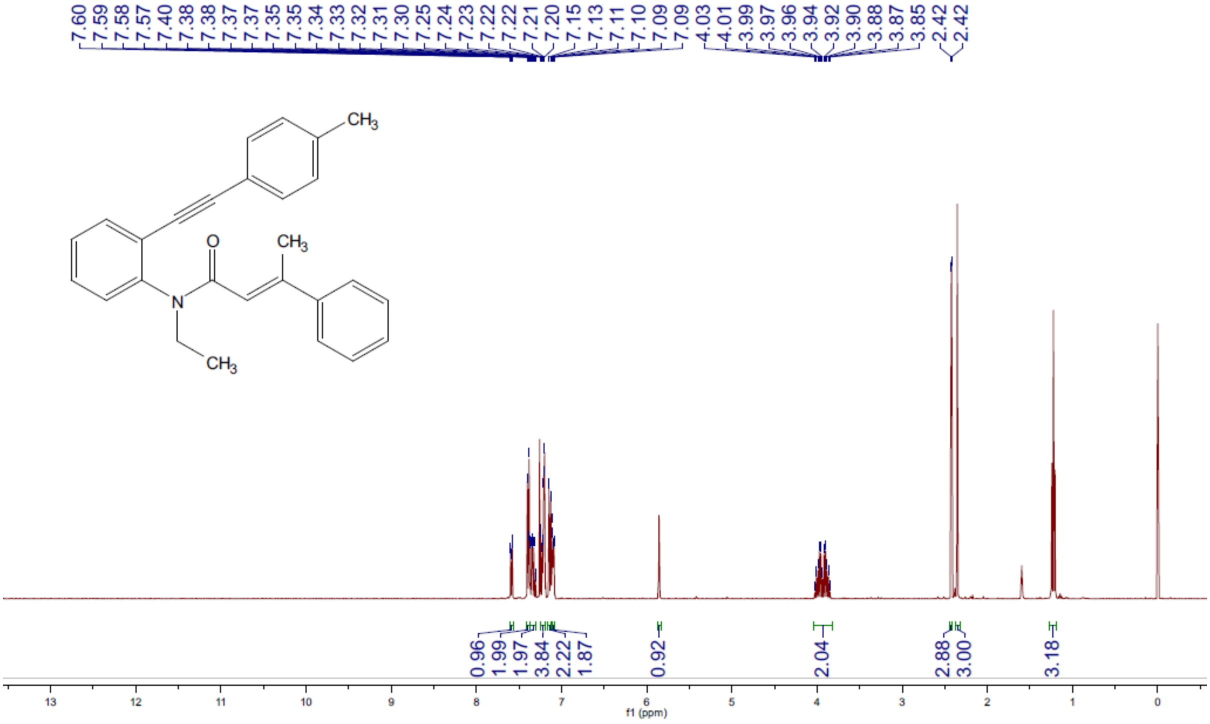

Supplementary Figure 314. ^{1}H NMR (400 MHz, $CDCl_3$) spectra of *(E)*-*N*-ethyl-3-phenyl-*N*-(2-(*p*-tolylethynyl)phenyl)but-2-enamide (**4m**)

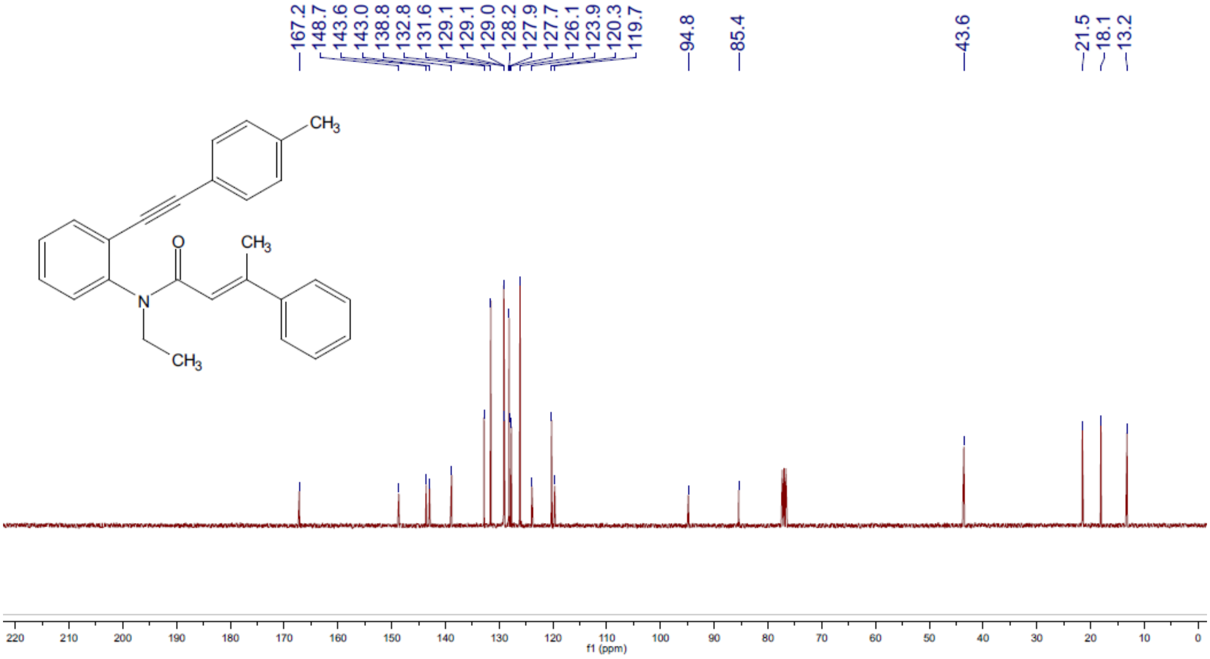

Supplementary Figure 315. ^{13}C NMR (100 MHz, $CDCl_3$) spectra of *(E)*-*N*-ethyl-3-phenyl-*N*-(2-(*p*-tolylethynyl)phenyl)but-2-enamide (**4m**)

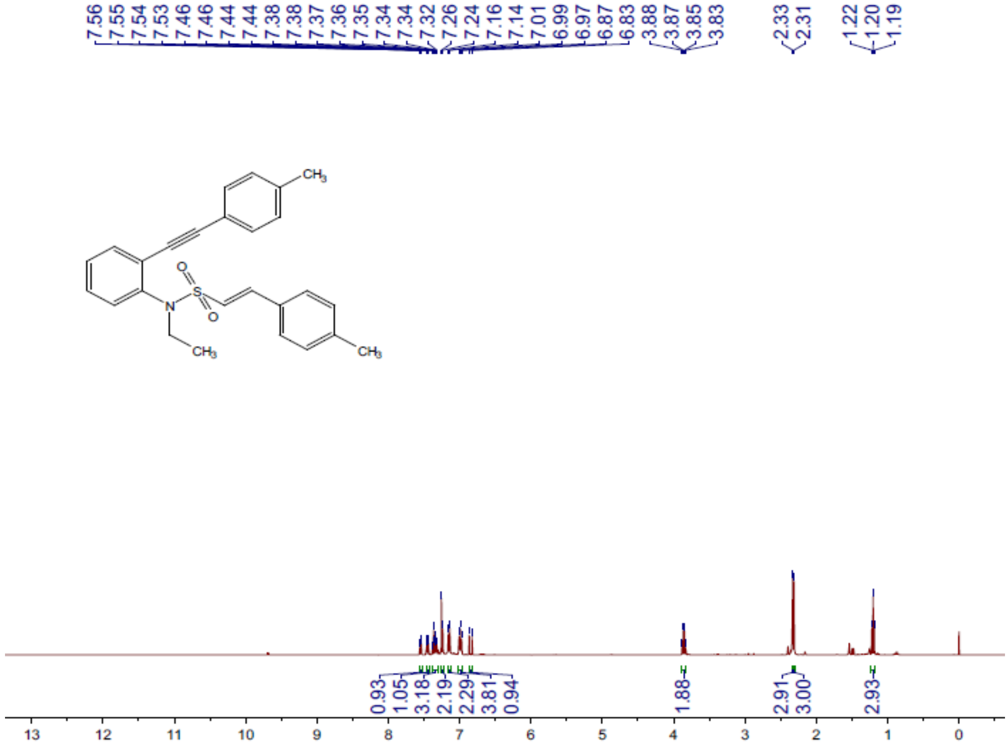

Supplementary Figure 316. ^{1}H NMR (400 MHz, $CDCl_3$) spectra of (*E*)-*N*-ethyl-2-(*p*-tolyl)-*N*-(2-(*p*-tolylethynyl)phenyl)ethene-1-sulfonamide (**4n**)

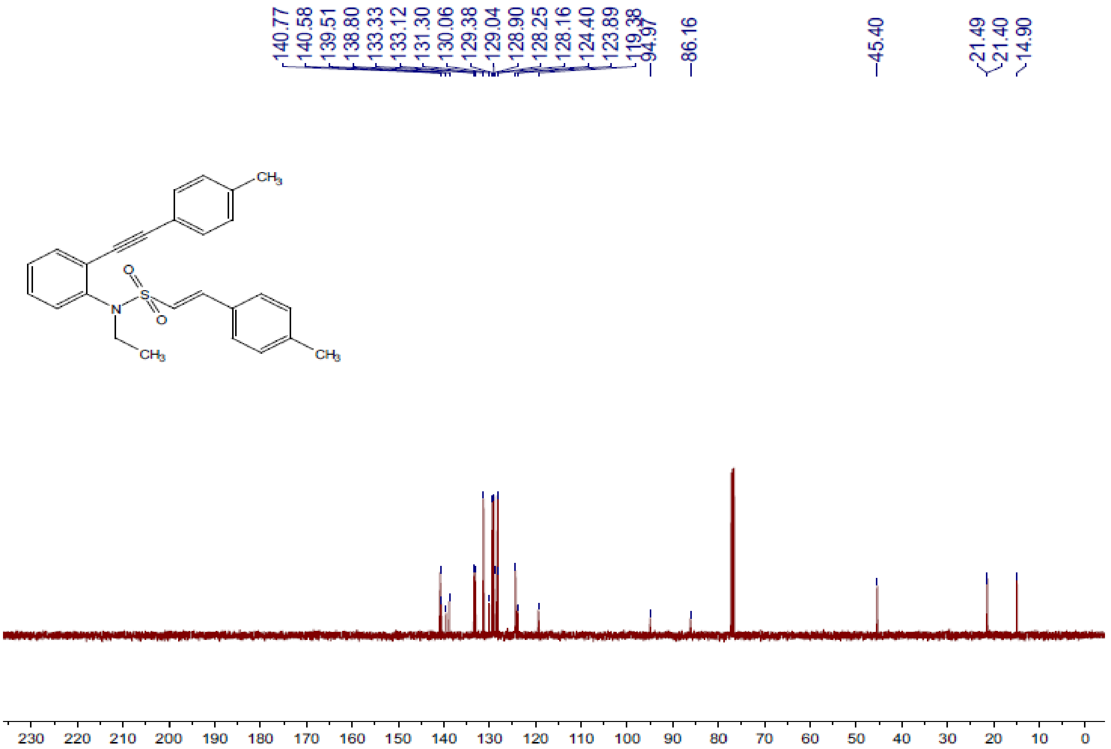

Supplementary Figure 317. ^{13}C NMR (100 MHz, $CDCl_3$) spectra of (*E*)-*N*-ethyl-2-(*p*-tolyl)-*N*-(2-(*p*-tolylethynyl)phenyl)ethene-1-sulfonamide (**4n**)

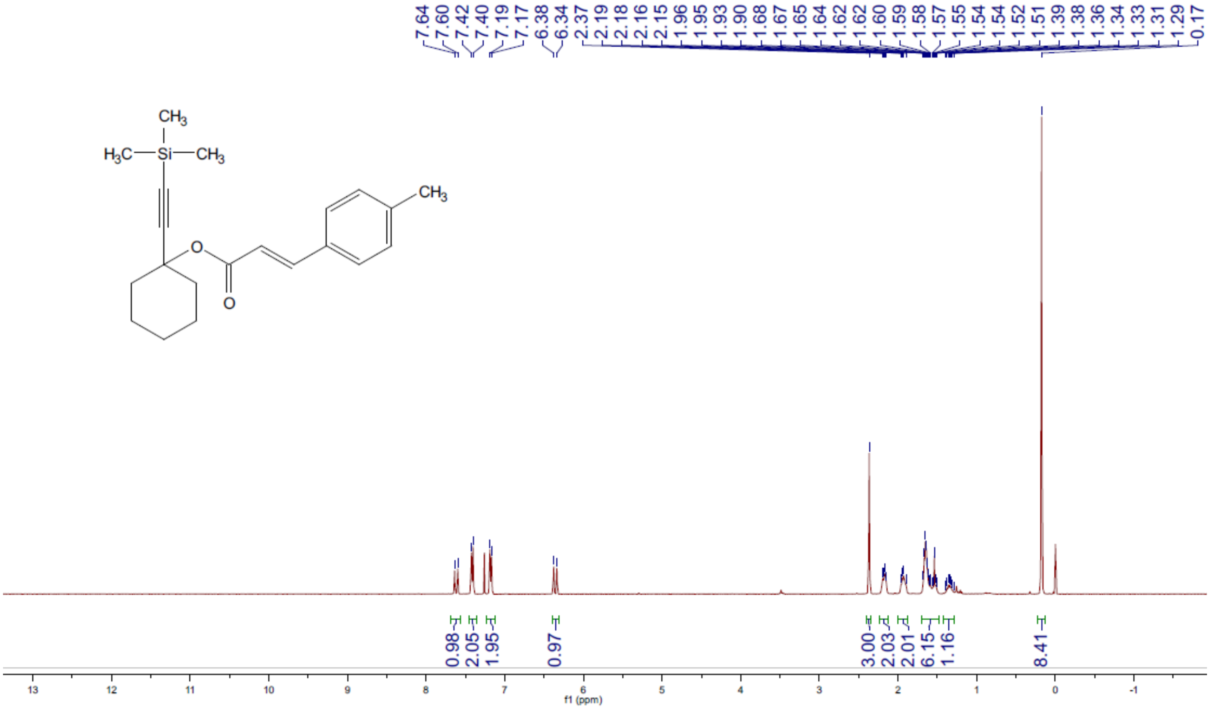

Supplementary Figure 318. ^{1}H NMR (400 MHz, $CDCl_3$) spectra of 1-((trimethylsilyl)ethynyl)cyclohexyl *(E)*-3-(*p*-tolyl)acrylate (**4o**)

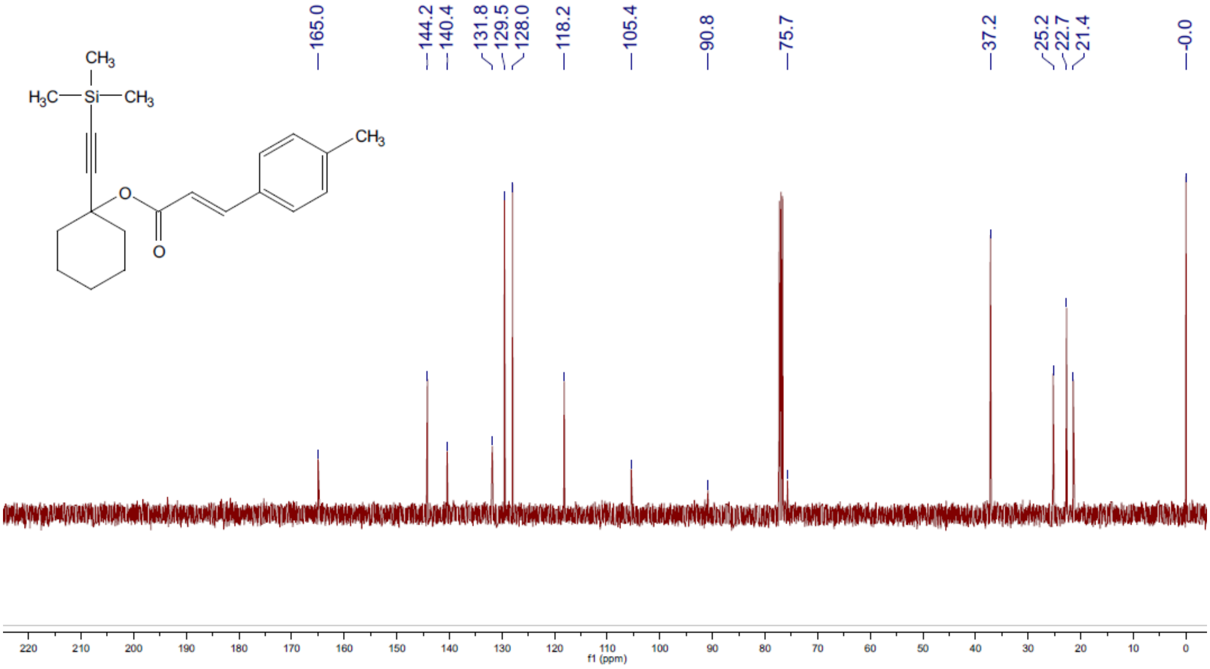

Supplementary Figure 319. ^{13}C NMR (100 MHz, $CDCl_3$) spectra of 1-((trimethylsilyl)ethynyl)cyclohexyl *(E)*-3-(*p*-tolyl)acrylate (**4o**)

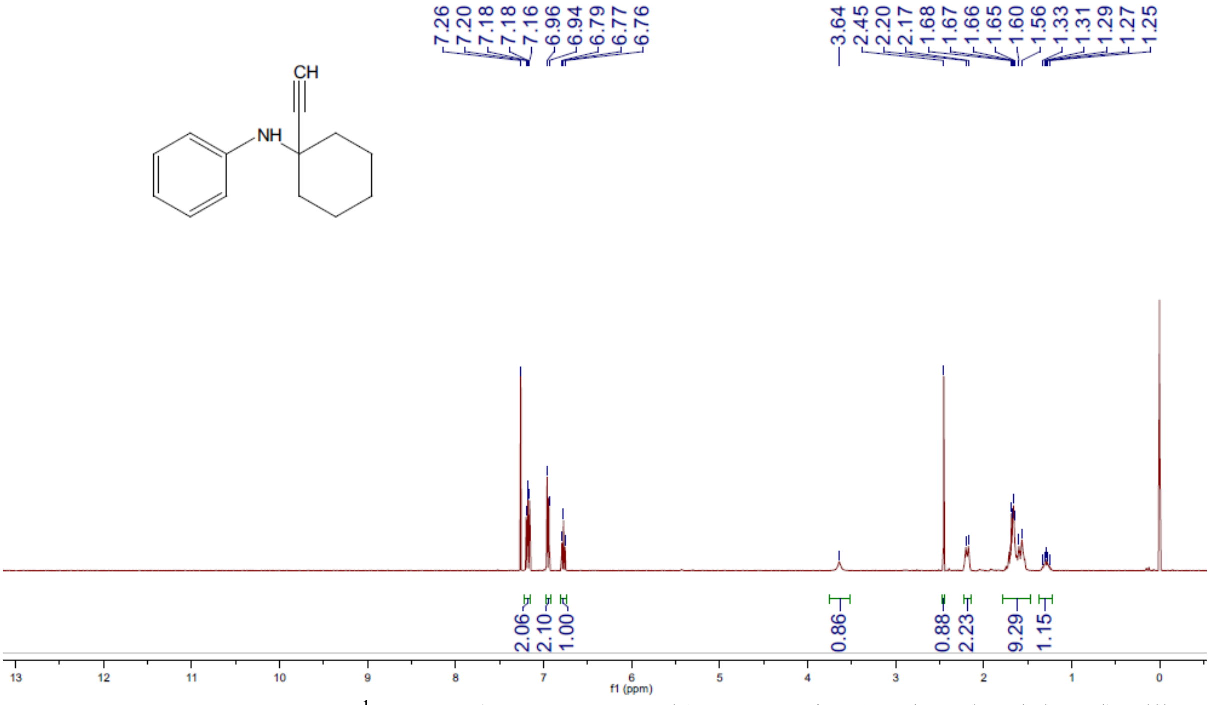

Supplementary Figure 320. ^{1}H NMR (400 MHz, $CDCl_3$) spectra of *N*-(1-ethynylcyclohexyl)aniline (**4′p**)

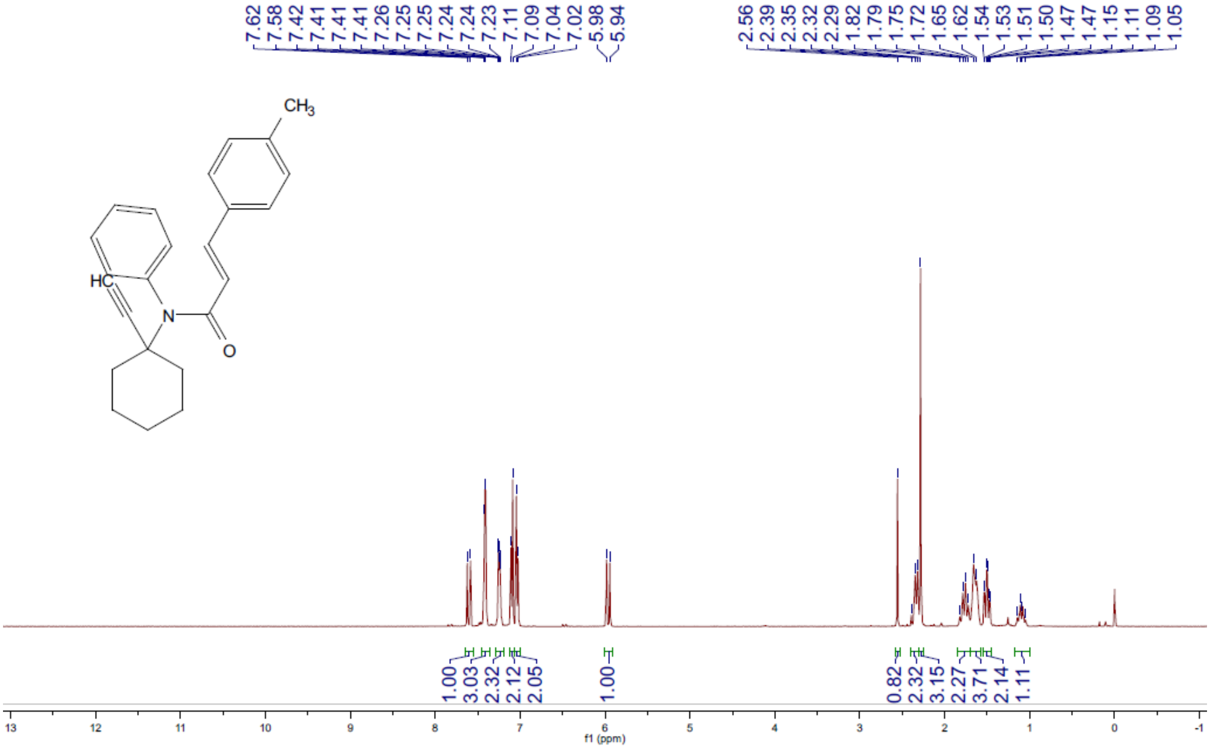

Supplementary Figure 321. ^{1}H NMR (400 MHz, $CDCl_3$) spectra of (*E*)-*N*-(1-ethynylcyclohexyl)-*N*-phenyl-3-(*p*-tolyl)acrylamide (**4″p**)

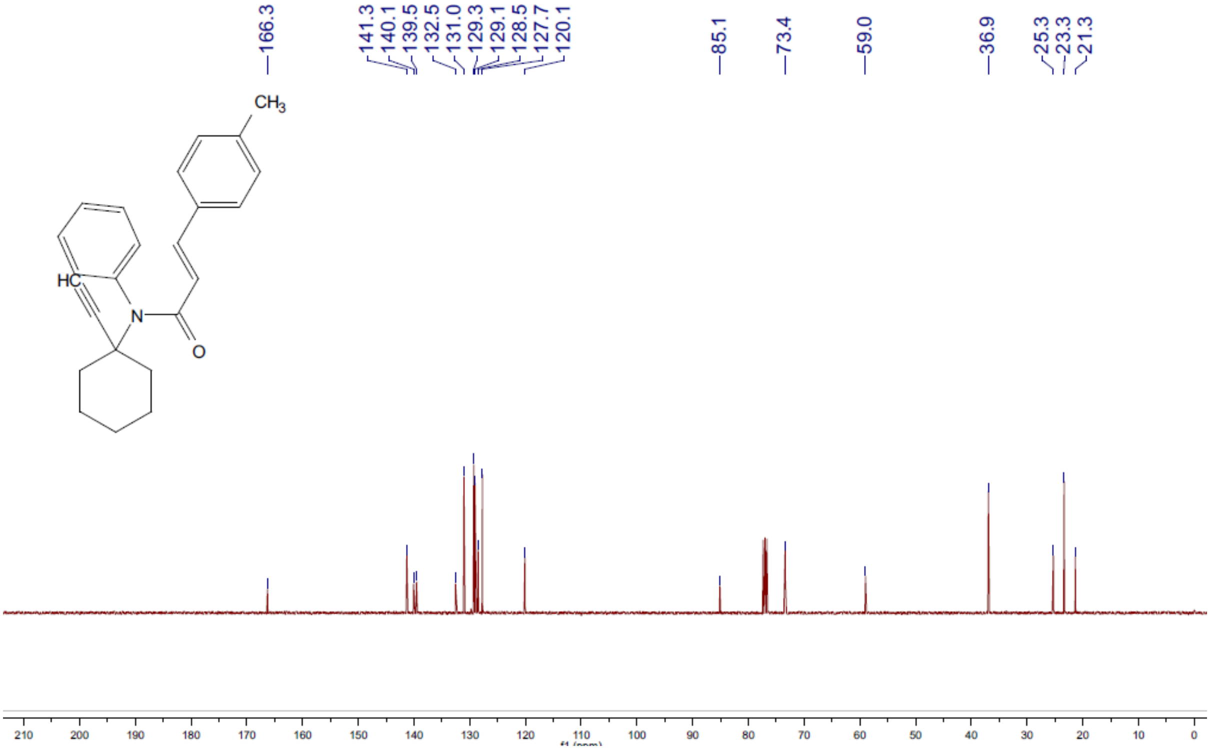

Supplementary Figure 322. ^{13}C NMR (100 MHz, $CDCl_3$) spectra of (*E)*-*N*-(1-ethynylcyclohexyl)-*N*-phenyl-3-(*p*-tolyl)acrylamide (**4″p**)

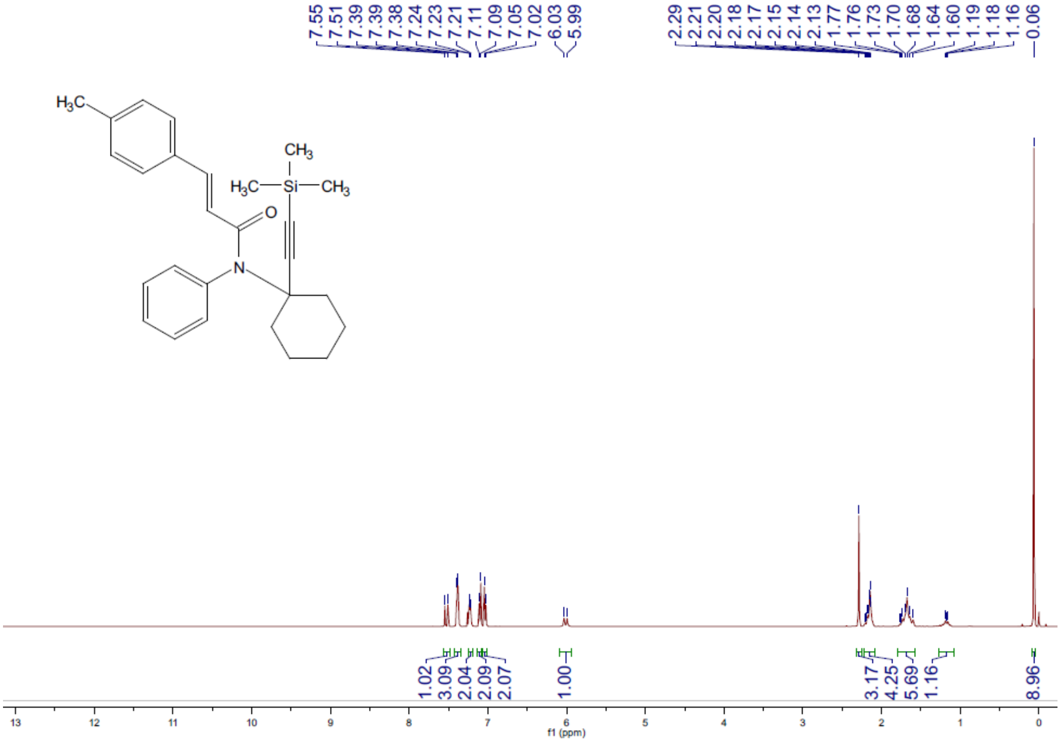

Supplementary Figure 323. ^{1}H NMR (400 MHz, $CDCl_3$) spectra of (*E*)-*N*-phenyl-3-(*p*-tolyl)-*N*-(1-((trimethylsilyl)ethynyl)cyclohexyl)acrylamide (**4p**)

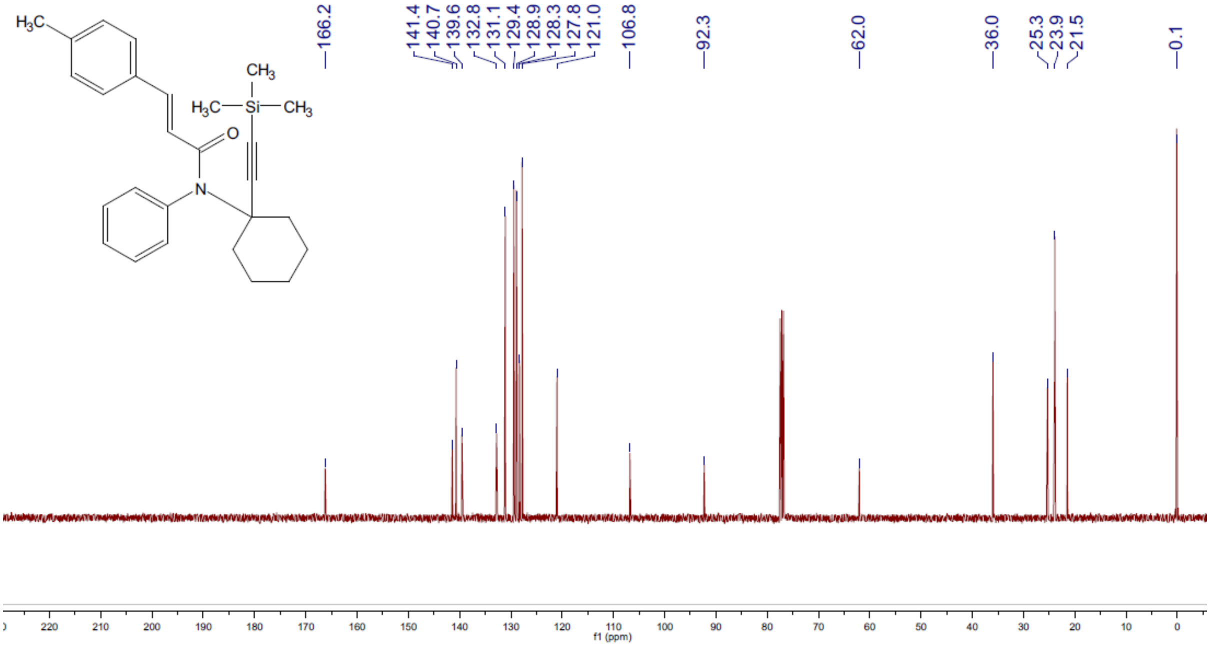

Supplementary Figure 324. ^{13}C NMR (100 MHz, $CDCl_3$) spectra of *(E)*-*N*-phenyl-3-(*p*-tolyl)-*N*-(1-((trimethylsilyl)ethynyl)cyclohexyl)acrylamide (**4p**)

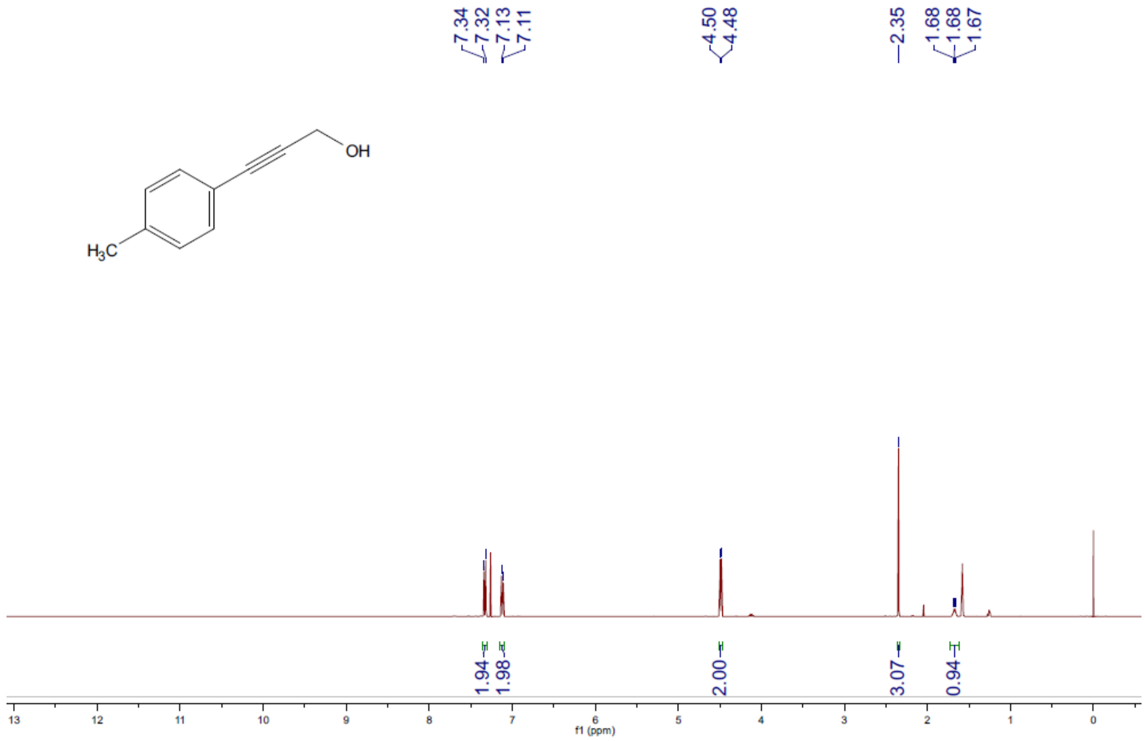

Supplementary Figure 325. ^{1}H NMR (400 MHz, $CDCl_3$) spectra of 3-(*p*-tolyl)prop-2-yn-1-ol (**S10**)

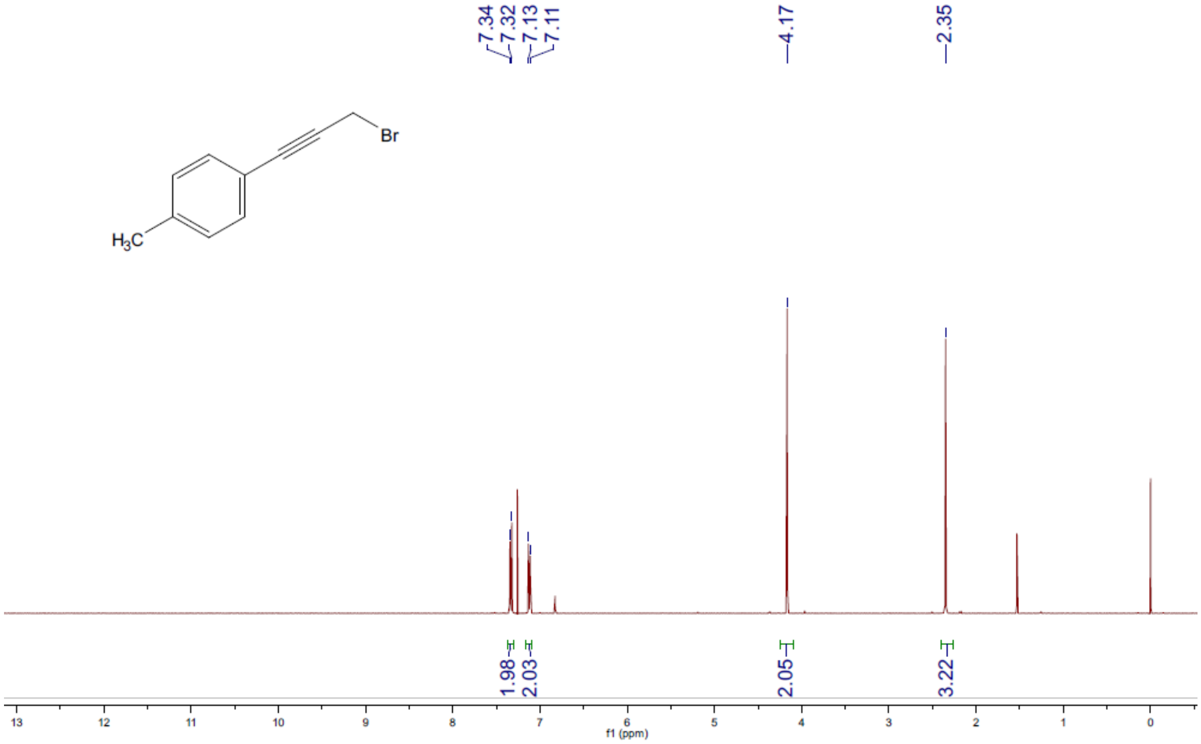

Supplementary Figure 326. ^{1}H NMR (400 MHz, $CDCl_3$) spectra of 1-(3-bromoprop-1-yn-1-yl)-4-methylbenzene (**S11′**)

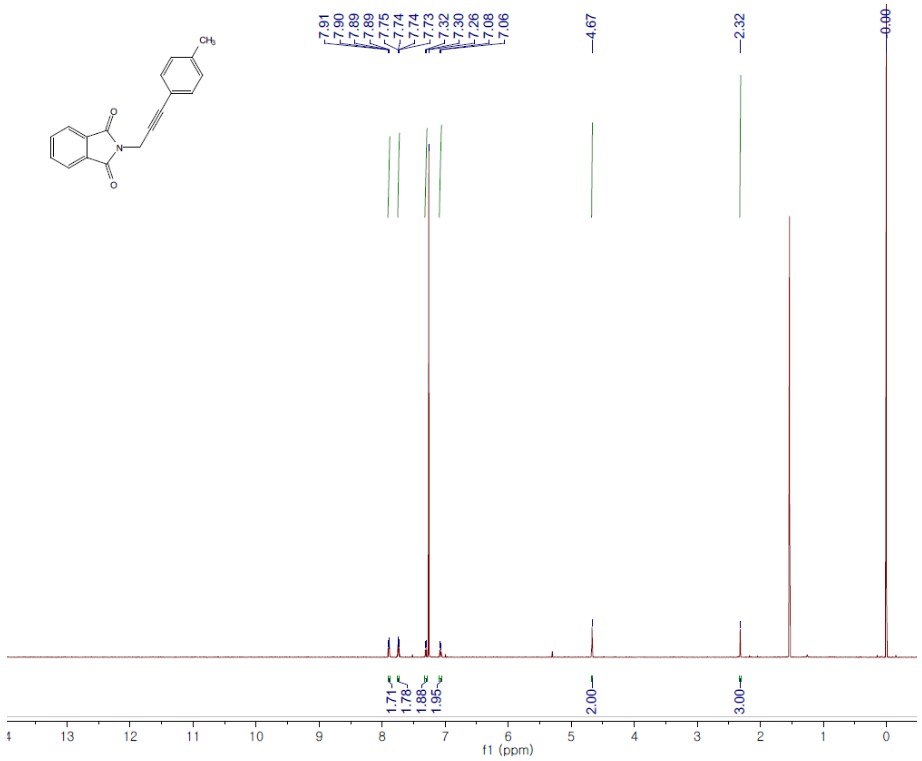

Supplementary Figure 327. ^{1}H NMR (400 MHz, $CDCl_3$) spectra of 2-(3-(*p*-tolyl)prop-2-yn-1-yl)isoindoline-1,3-dione (**S11″**)

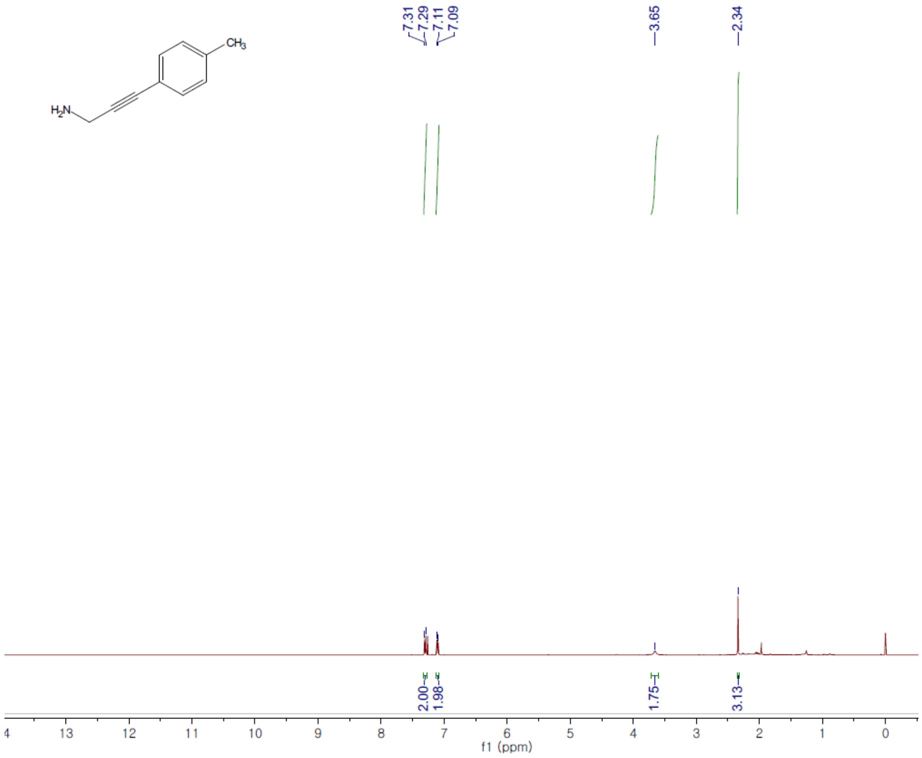

Supplementary Figure 328. ^{1}H NMR (400 MHz, $CDCl_3$) spectra of 3-(*p*-tolyl)prop-2-yn-1-amine (**S11**)

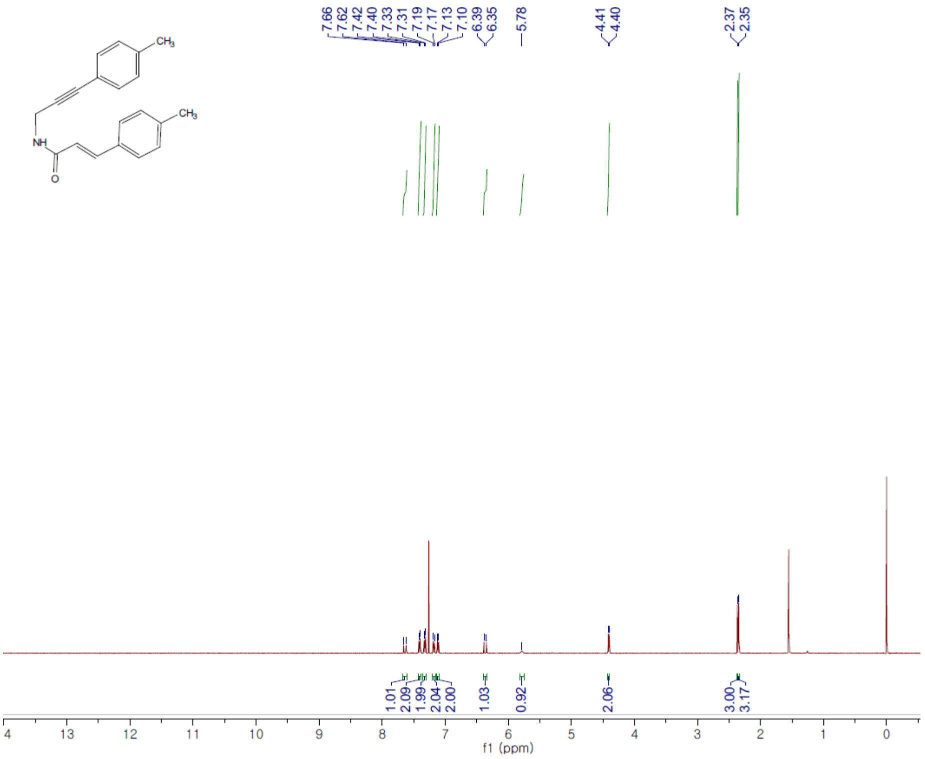

Supplementary Figure 329. ^{1}H NMR (400 MHz, $CDCl_3$) spectra of (*E*)-3-(*p*-tolyl)-*N*-(3-(*p*-tolyl)prop-2-yn-1-yl)acrylamide (**4q′**)

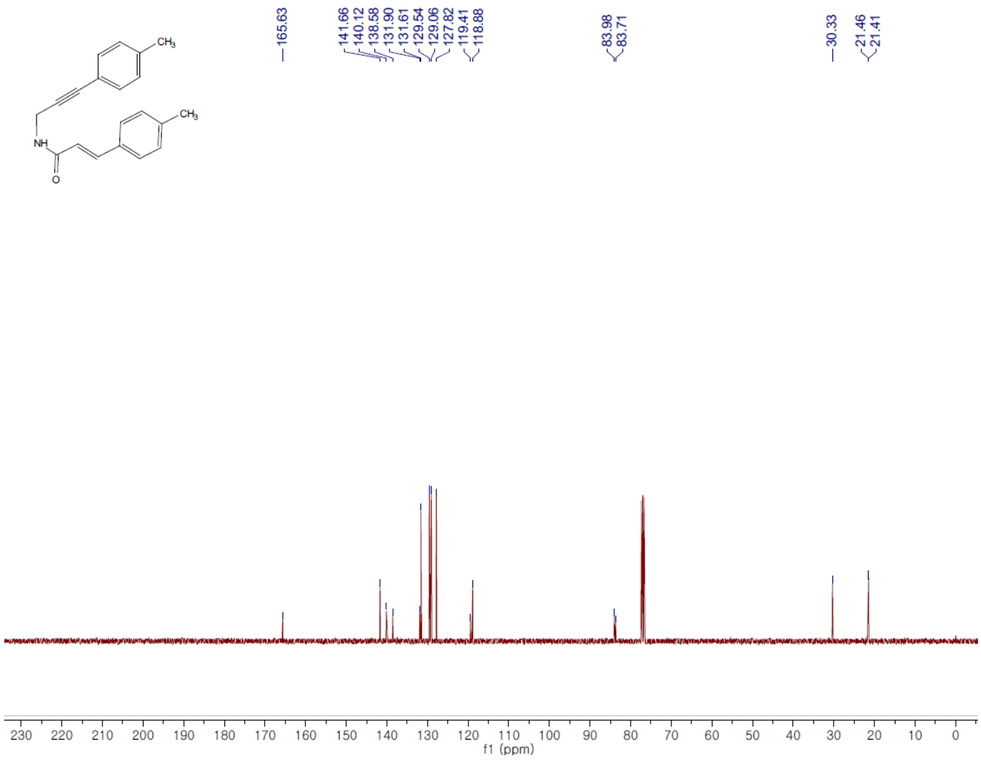

Supplementary Figure 330. ^{13}C NMR (100 MHz, $CDCl_3$) spectra of (*E*)-3-(*p*-tolyl)-*N*-(3-(*p*-tolyl)prop-2-yn-1-yl)acrylamide (**4q′**)

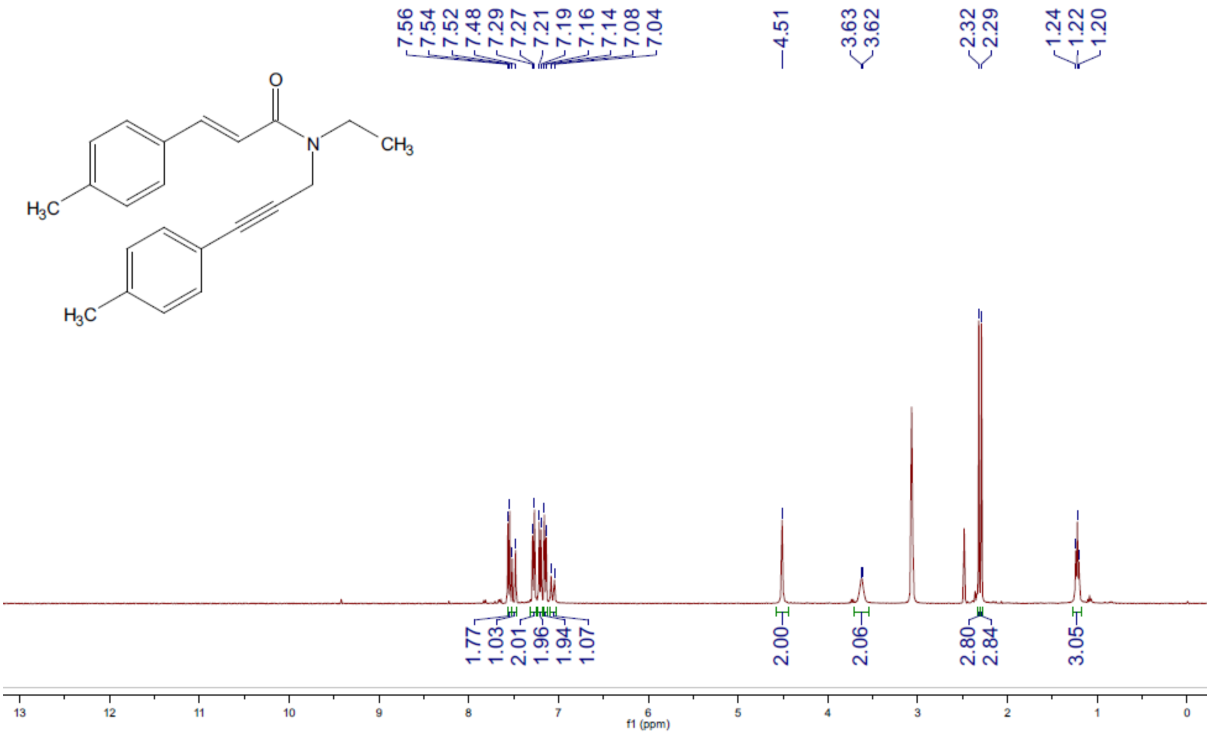

Supplementary Figure 331. ^{1}H NMR (400 MHz, DMSO-d$_6$, 90°C) of (*E*)-*N*-ethyl-3-(*p*-tolyl)-*N*-(3-(*p*-tolyl)prop-2-yn-1-yl)acrylamide (**4q**)

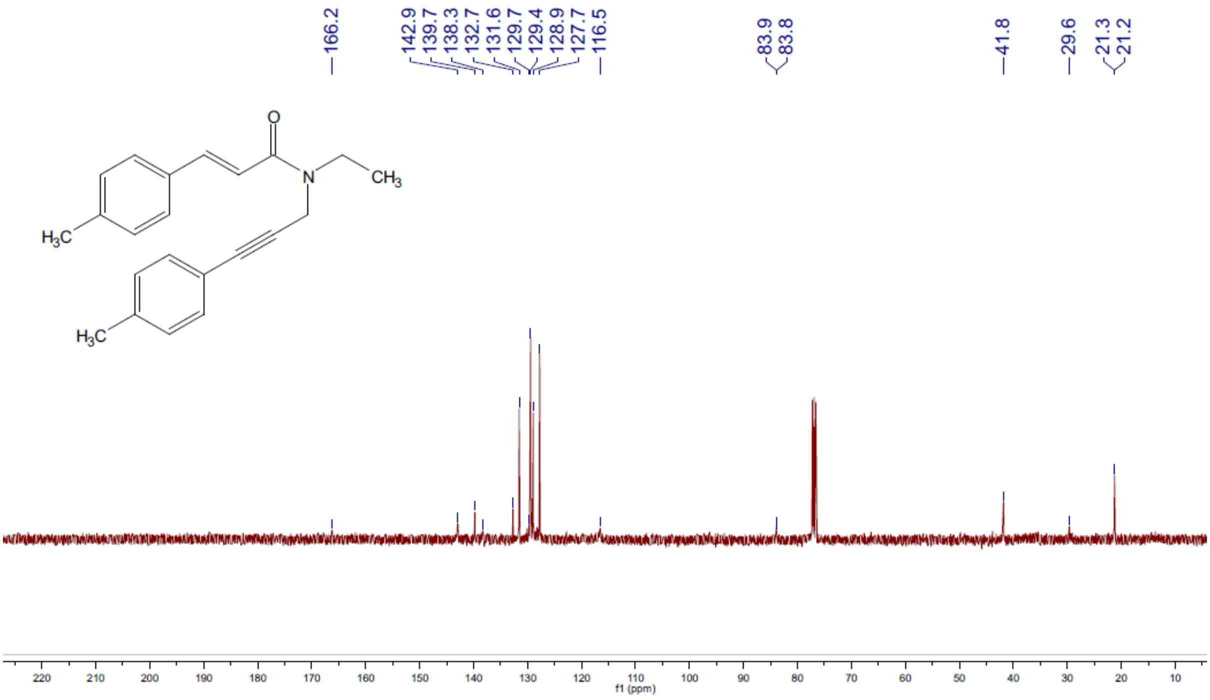

Supplementary Figure 332. ^{13}C NMR (100 MHz, $CDCl_3$, 45°C) of (*E*)-*N*-ethyl-3-(*p*-tolyl)-*N*-(3-(*p*-tolyl)prop-2-yn-1-yl)acrylamide (**4q**)

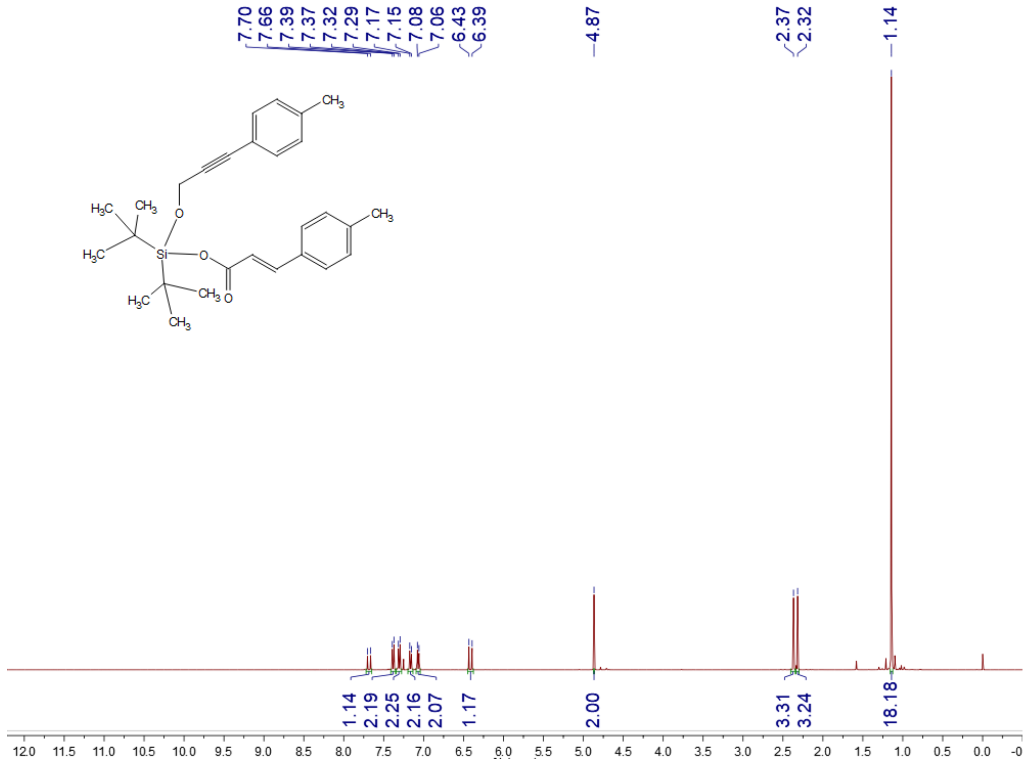

Supplementary Figure 333. ^{1}H NMR (400 MHz, $CDCl_3$) spectra of Di-*tert*-butyl((3-(*p*-tolyl)prop-2-yn-1-yl)oxy)silyl (*E*)-3-(*p*-tolyl)acrylate (**4r**)

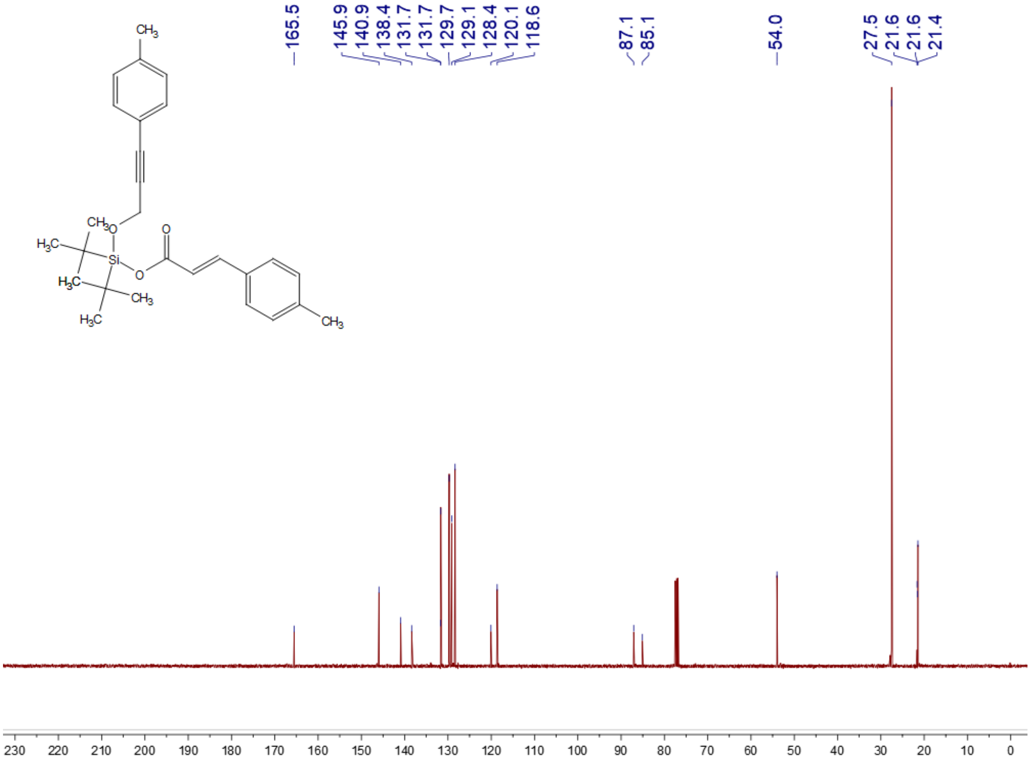

Supplementary Figure 334. ^{13}C NMR (100 MHz, $CDCl_3$) spectra of Di-tert-butyl((3-(*p*-tolyl)prop-2-yn-1-yl)oxy)silyl (*E*)-3-(*p*-tolyl)acrylate (**4r**)

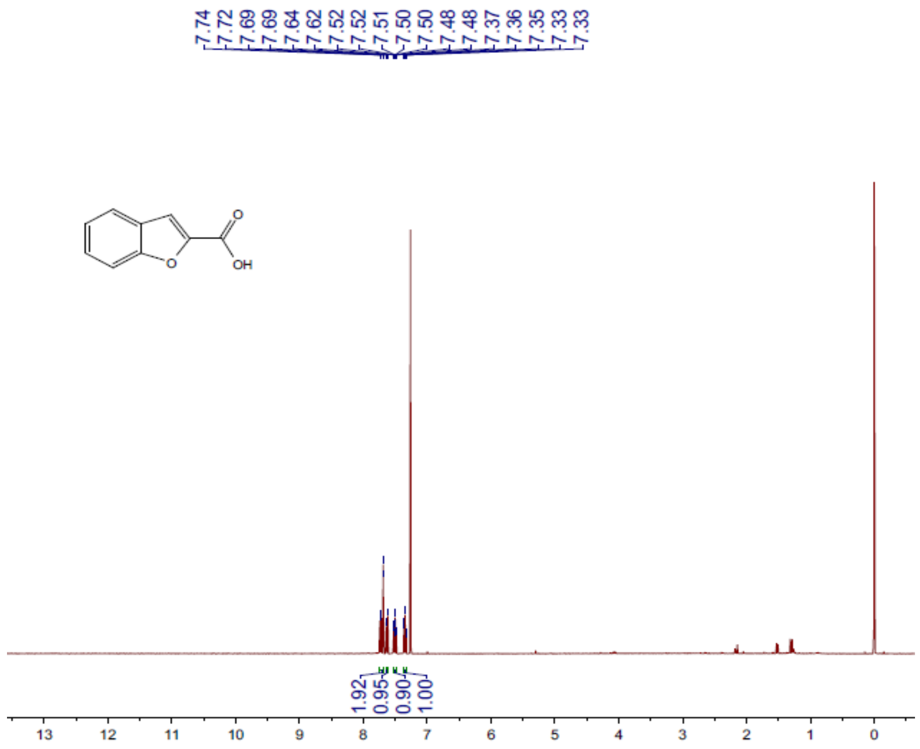

Supplementary Figure 335. ^{1}H NMR (400 MHz, $CDCl_3$) spectra of benzofuran-2-carboxylic acid (**4s′**)

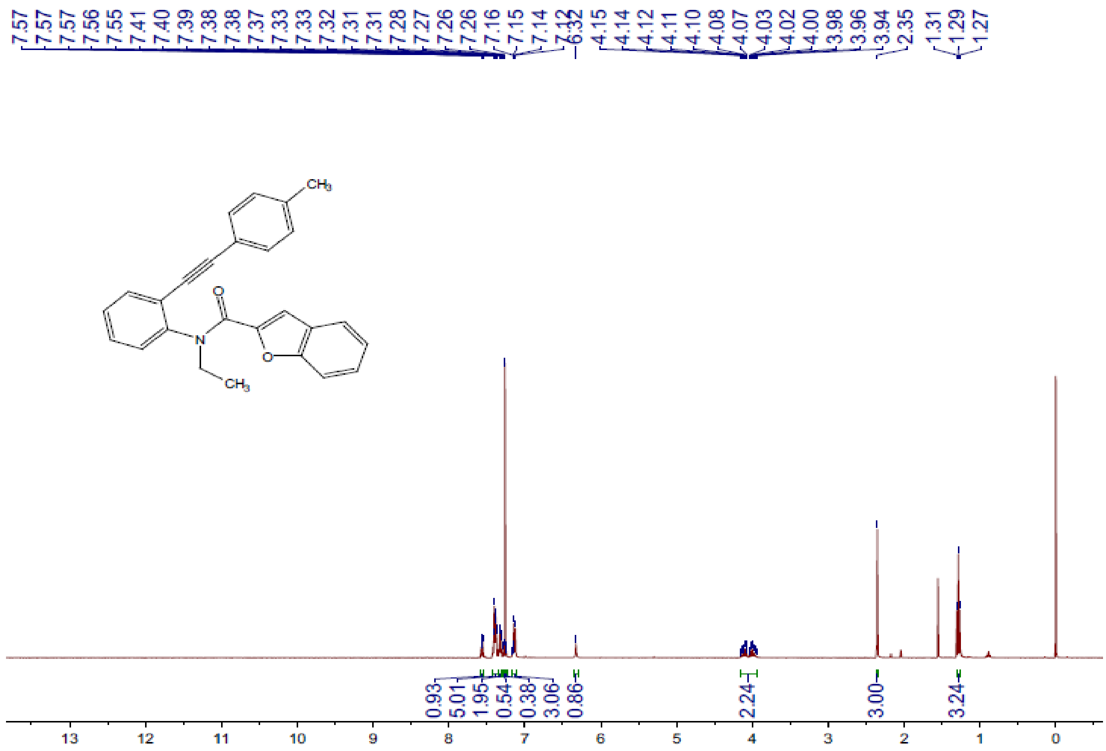

Supplementary Figure 336. ^{1}H NMR (400 MHz, $CDCl_3$) spectra of *N*-ethyl-*N*-(2-(*p*-tolylethynyl)phenyl)benzofuran-2-carboxamide (**4s**)

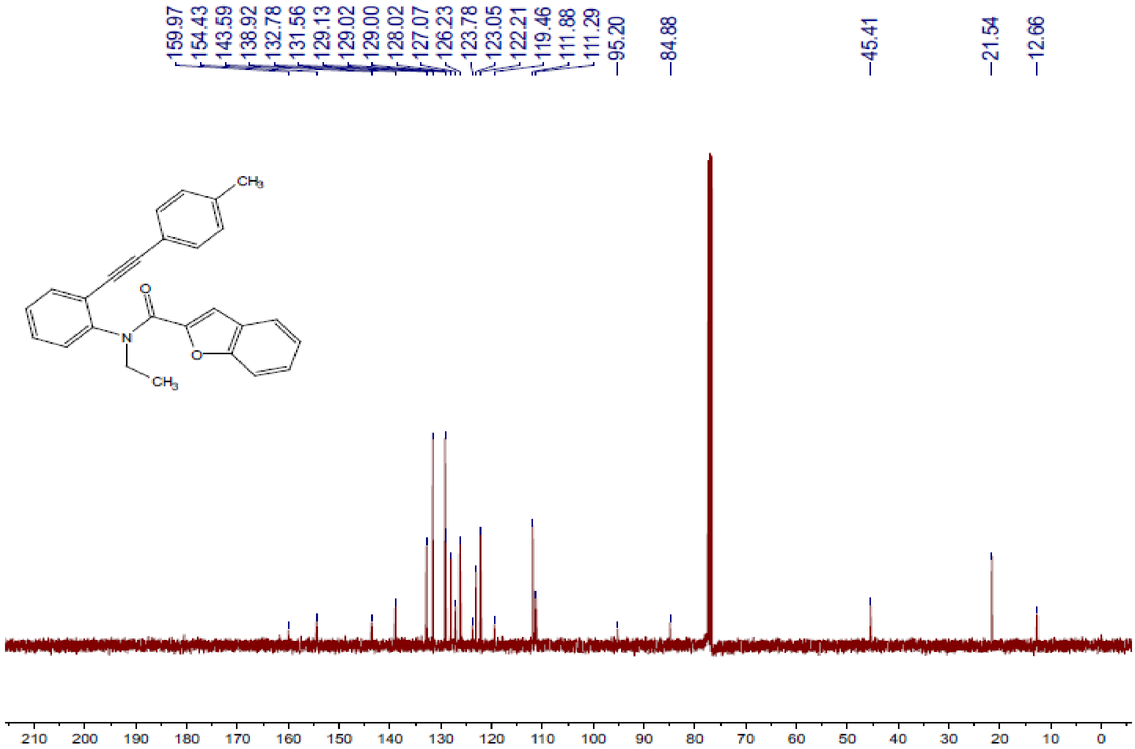

Supplementary Figure 337. ^{13}C NMR (100 MHz, $CDCl_3$) spectra of *N*-ethyl-*N*-(2-(*p*-tolylethynyl)phenyl)benzofuran-2-carboxamide (**4s**)

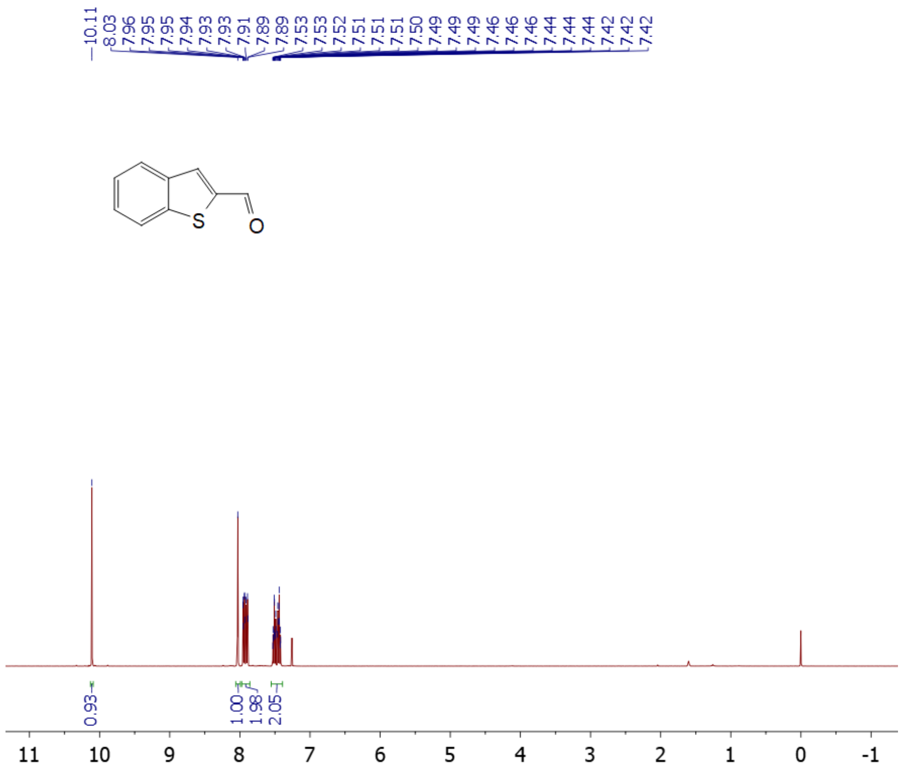

Supplementary Figure 338. ^{1}H NMR (400 MHz, $CDCl_3$) spectra of benzo[*b*]thiophene-2-carbaldehyde (**4′t**)

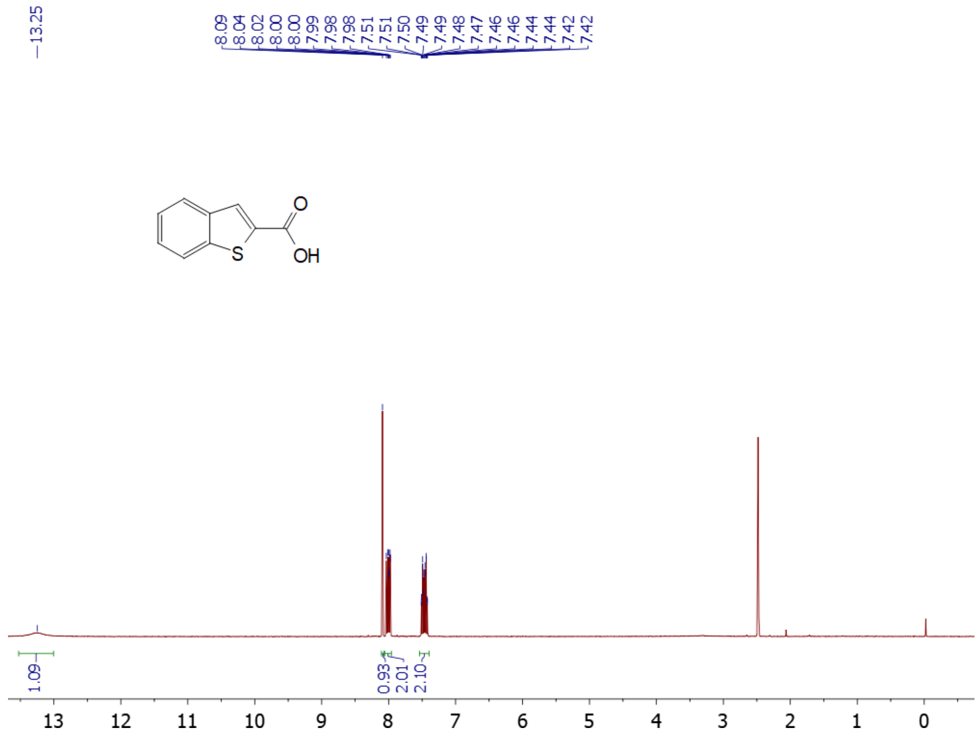

Supplementary Figure 339. ^{1}H NMR (400 MHz, DMSO-d$_6$) spectra of benzo[*b*]thiophene-2-carboxylic acid (**4″t**)

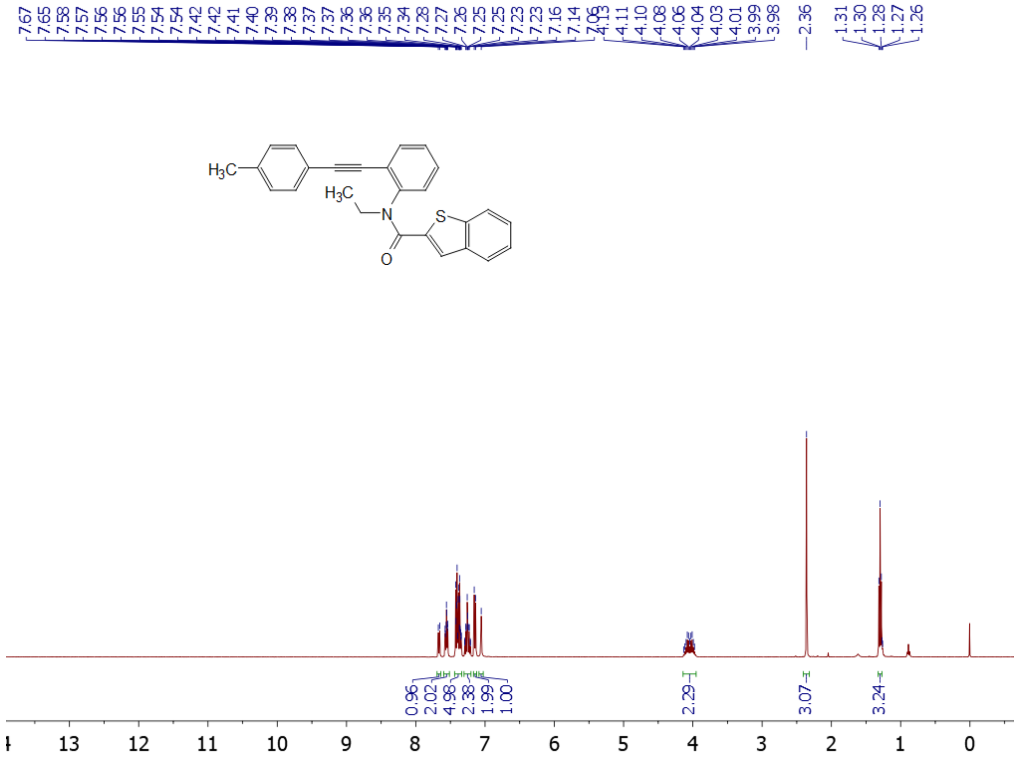

Supplementary Figure 340. ^{1}H NMR (400 MHz, CDCl$_3$) spectra of *N*-ethyl-*N*-(2-(*p*-tolylethynyl)phenyl)benzo[*b*]thiophene-2-carboxamide (**4t**)

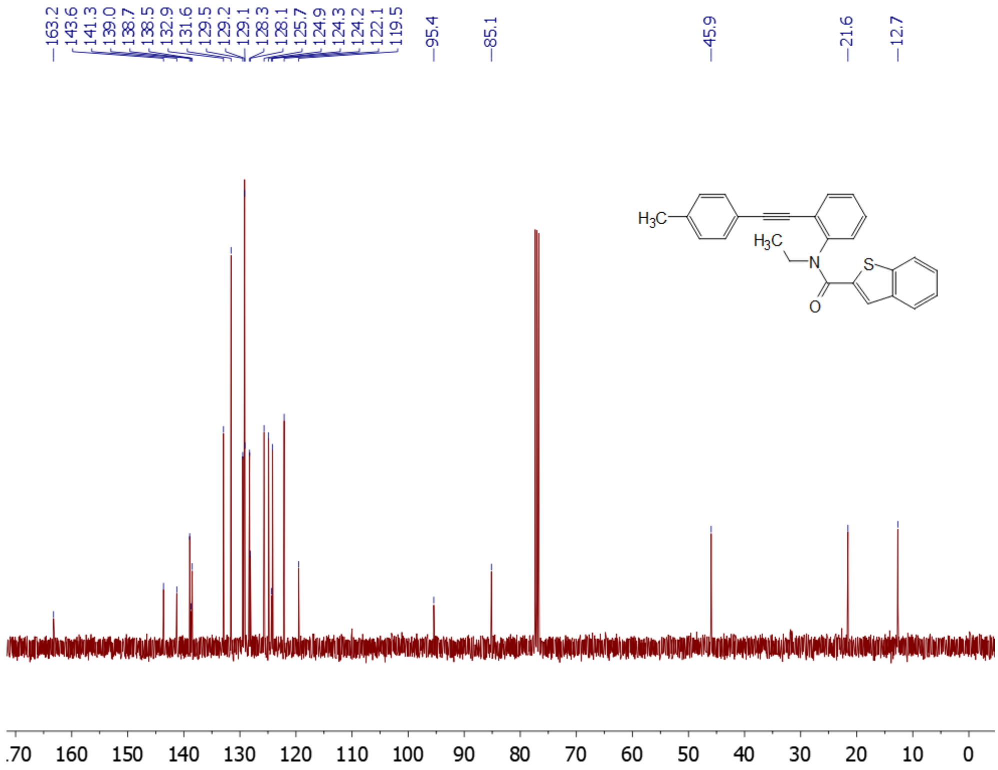

Supplementary Figure 341. ^{13}C NMR (100 MHz, $CDCl_3$) spectra of *N*-ethyl-*N*-(2-(*p*-tolylethynyl)phenyl)benzo[*b*]thiophene-2-carboxamide (**4t**)

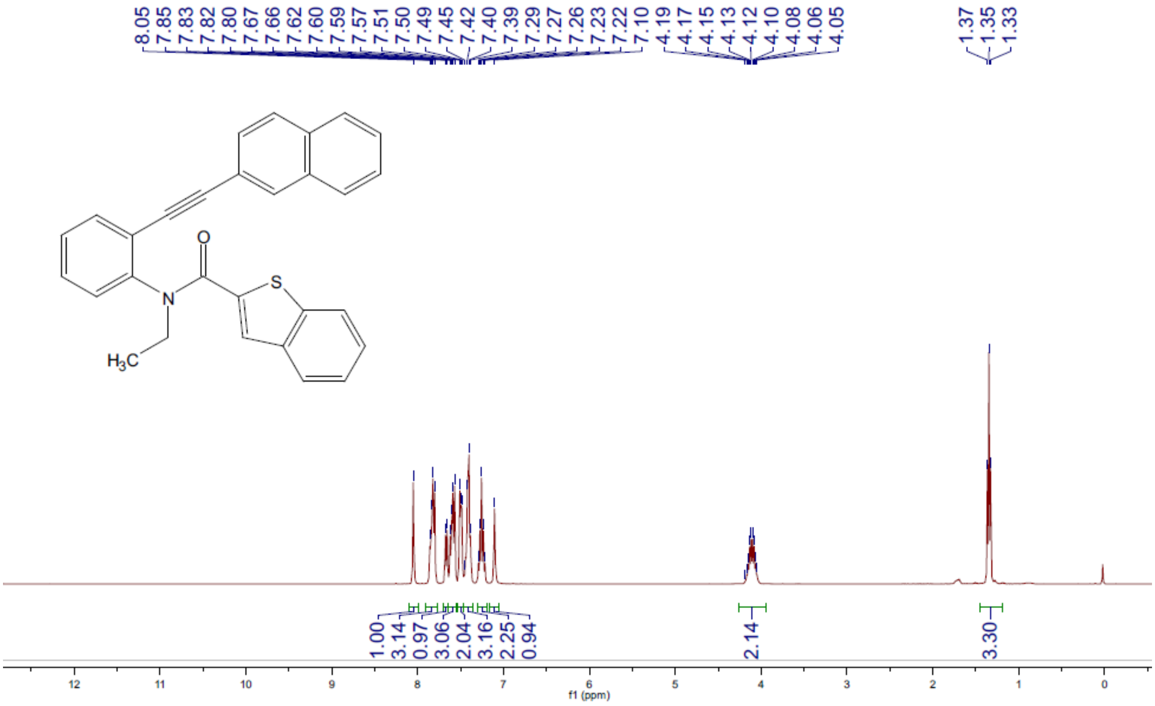

Supplementary Figure 342. ^{1}H NMR (400 MHz, $CDCl_3$) spectra of *N*-ethyl-*N*-(2-(naphthalen-2-ylethynyl)phenyl)benzo[*b*]thiophene-2-carboxamide (**4u**)

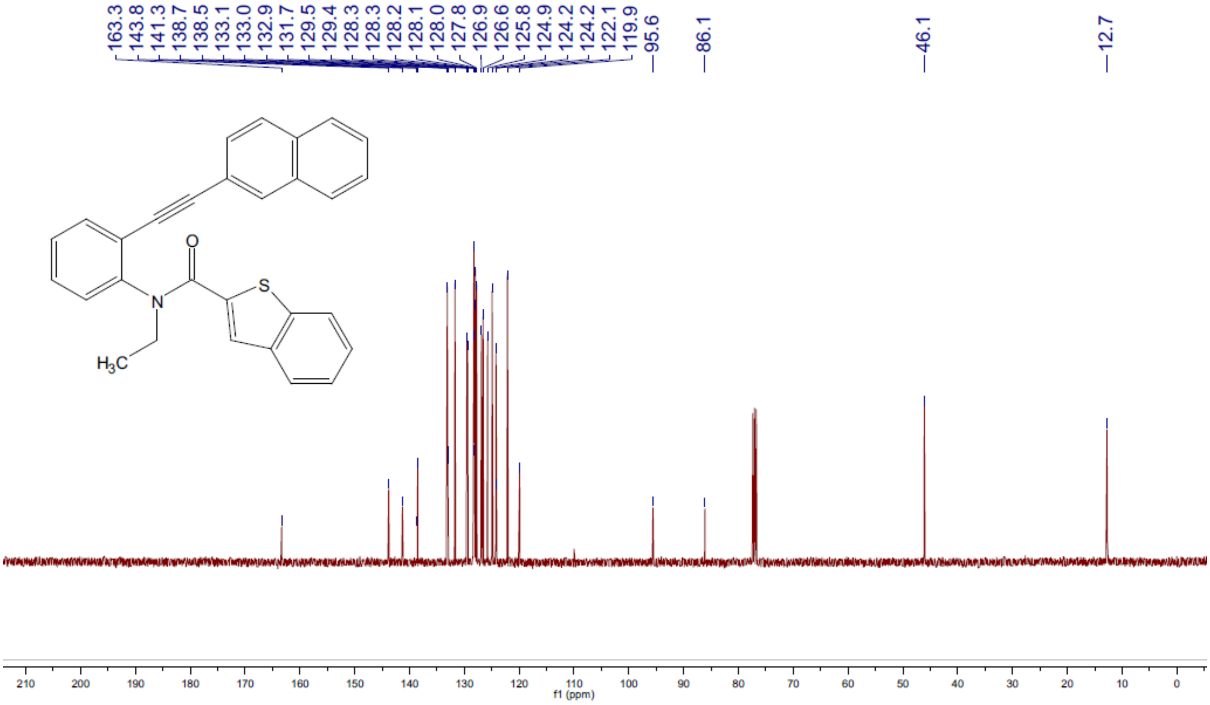

Supplementary Figure 343. ^{13}C NMR (100 MHz, $CDCl_3$) spectra of *N*-ethyl-*N*-(2-(naphthalen-2-ylethynyl)phenyl)benzo[*b*]thiophene-2-carboxamide (**4u**)

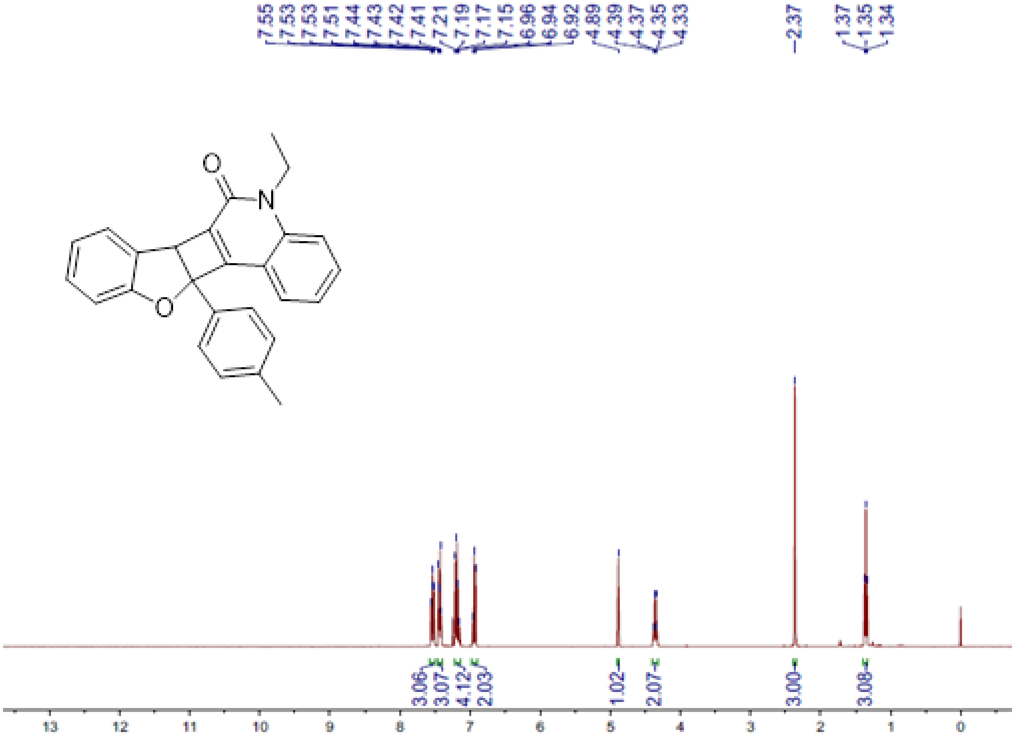

Supplementary Figure 344. ^{1}H NMR (400 MHz, $CDCl_3$) spectra of 5-ethyl-11a-(*p*-tolyl)-6b,11a-dihydrobenzofuro[2',3':3,4]cyclobuta[1,2-*c*]quinolin-6(5*H*)-one (**5s'**)

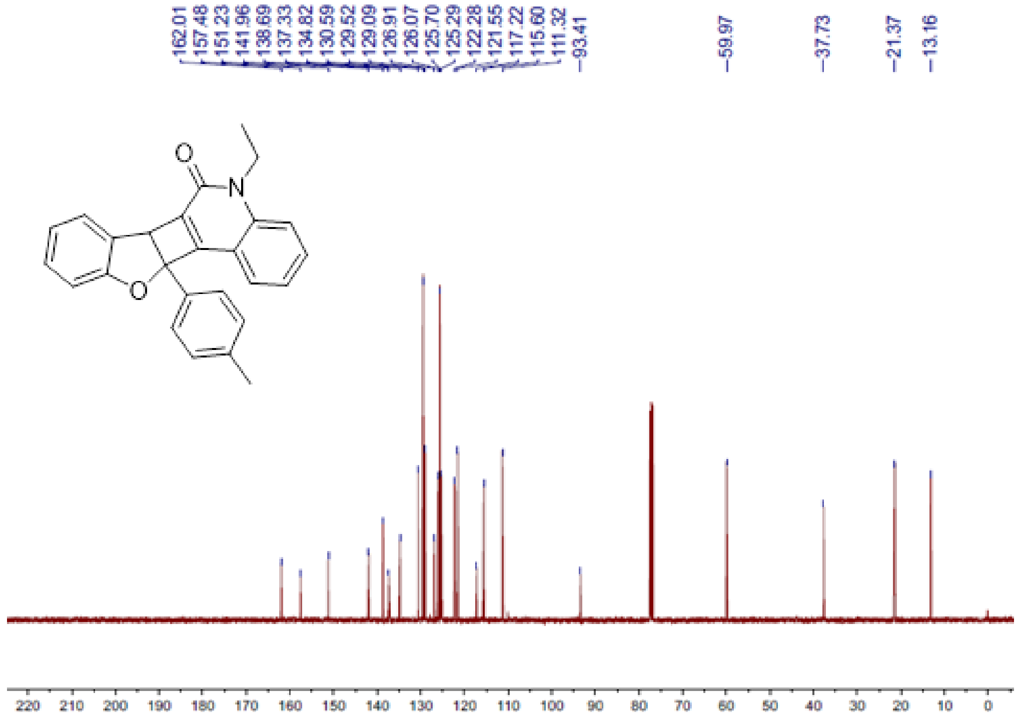

Supplementary Figure 345. ^{13}C NMR (100 MHz, $CDCl_3$) spectra of 5-ethyl-11a-(*p*-tolyl)-6b,11a-dihydrobenzofuro[2',3':3,4]cyclobuta[1,2-*c*]quinolin-6(5*H*)-one (**5s'**)

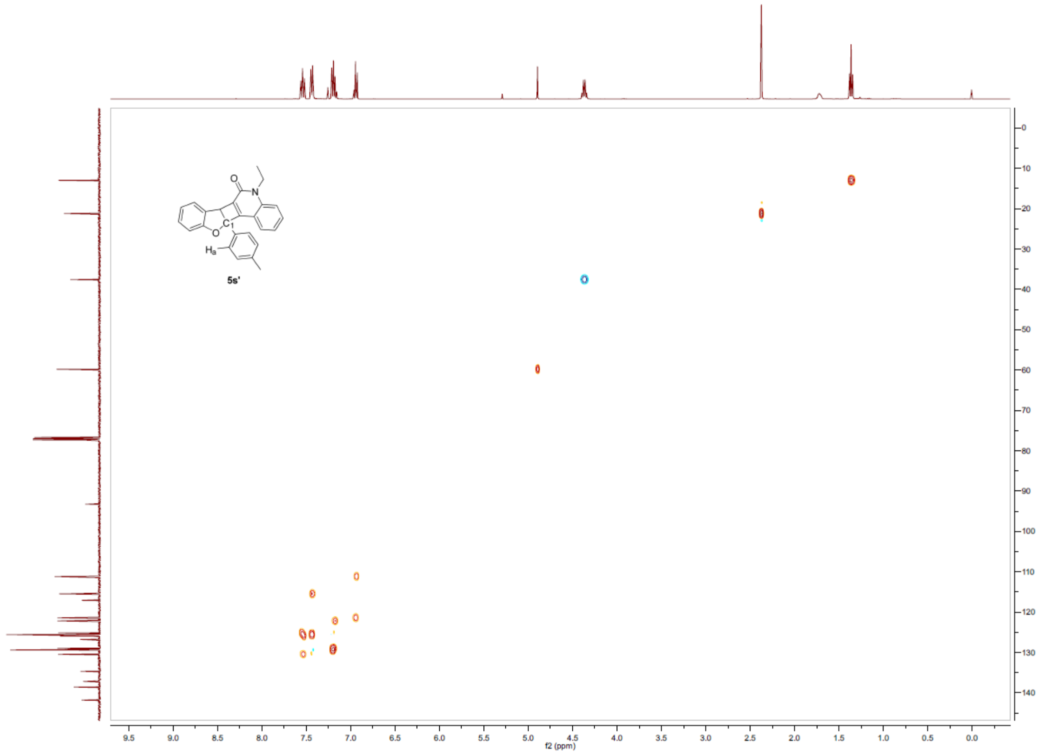

Supplementary Figure 346. HSQC of compound **5s'**.

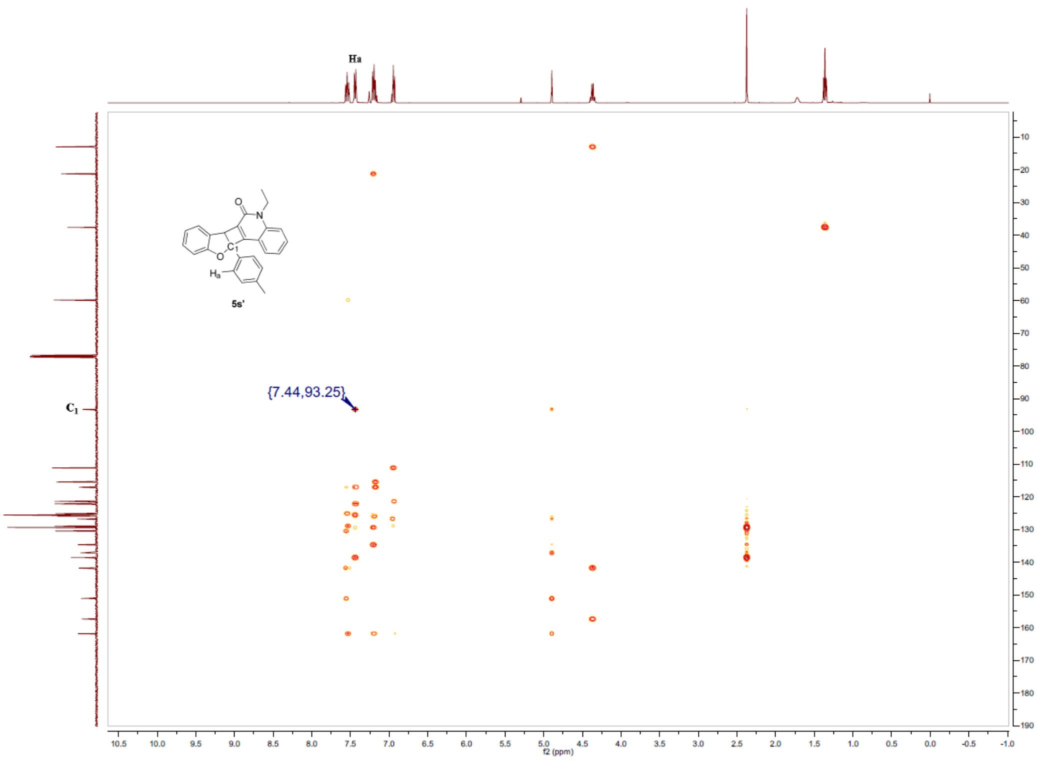

Supplementary Figure 347. HMBC of compound **5s'**.

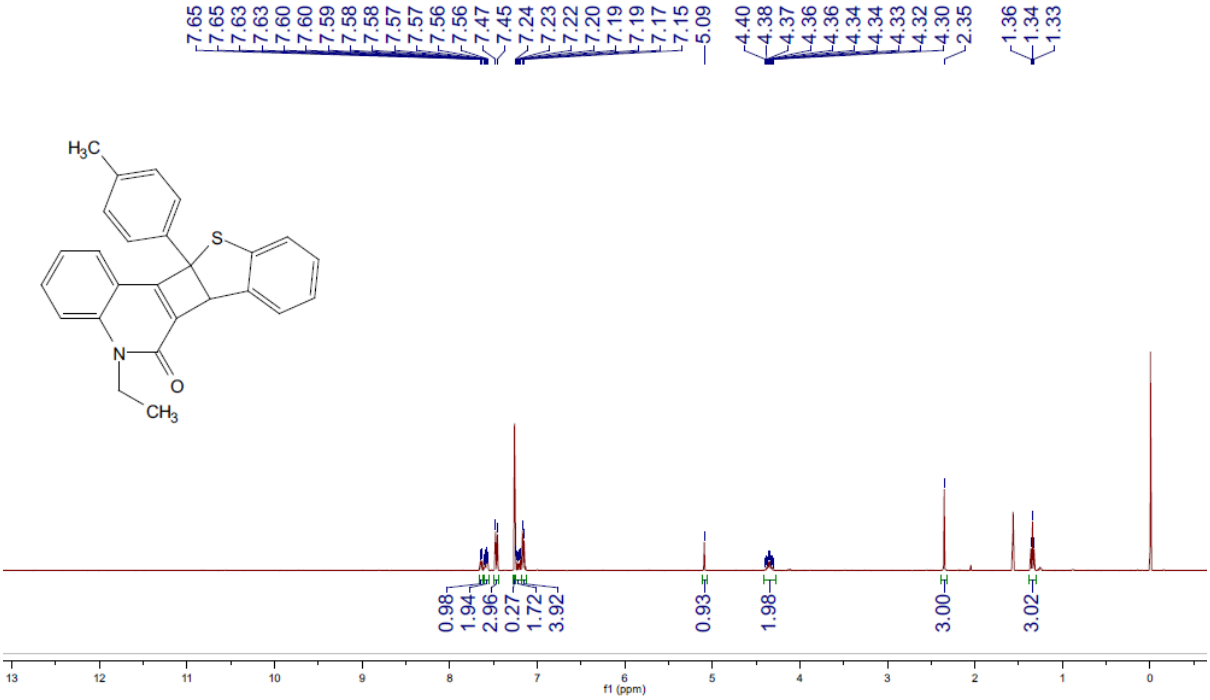

Supplementary Figure 348. ^{1}H NMR (400 MHz, $CDCl_3$) spectra of 5-ethyl-11a-(*p*-tolyl)-6b,11a-dihydrobenzo[4',5']thieno[2',3':3,4]cyclobuta[1,2-c]quinolin-6(5H)-one (**5t'**)

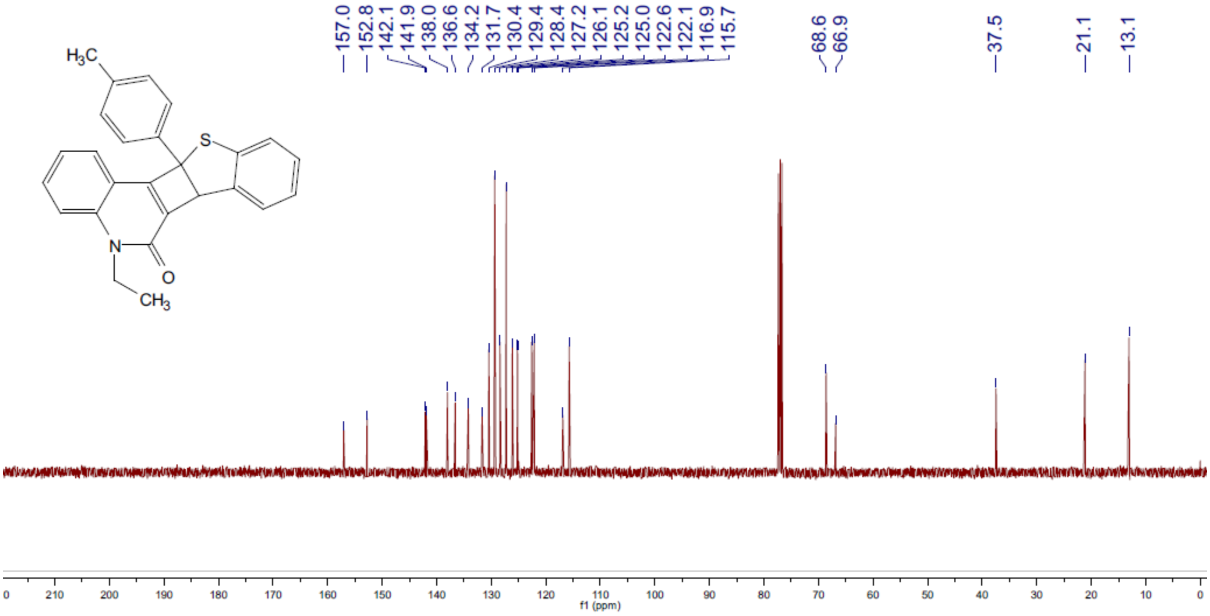

Supplementary Figure 349. ^{13}C NMR (100 MHz, $CDCl_3$) spectra of 5-ethyl-11a-(*p*-tolyl)-6b,11a-dihydrobenzo[4',5']thieno[2',3':3,4]cyclobuta[1,2-c]quinolin-6(5H)-one (**5t'**)

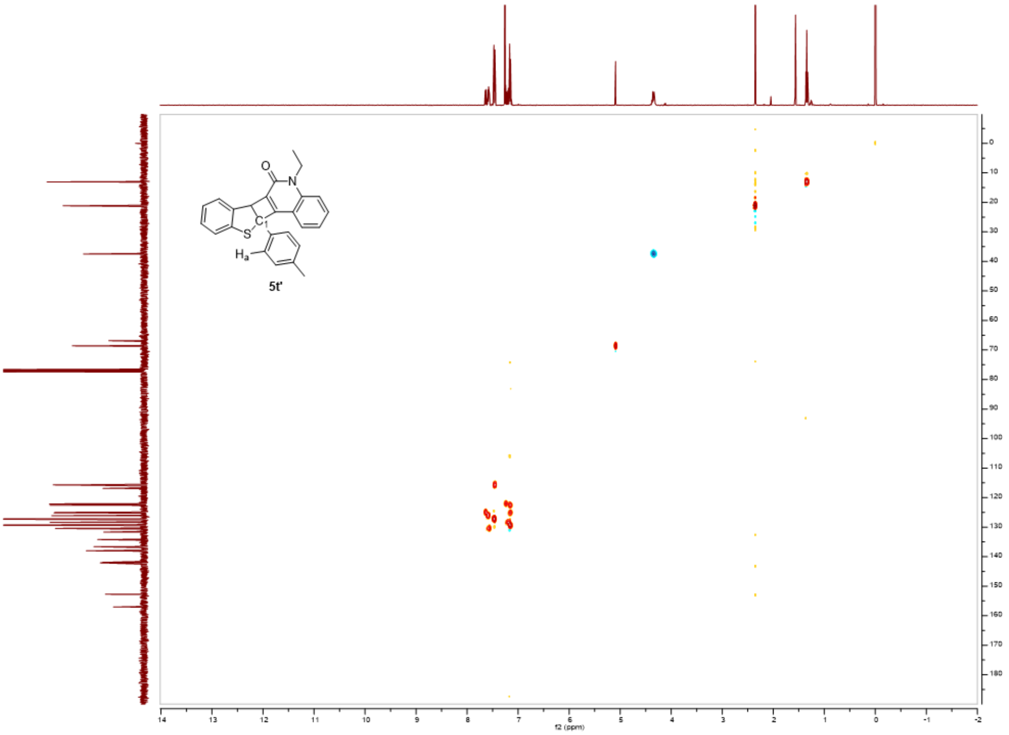

Supplementary Figure 350. HSQC of compound **5t'**.

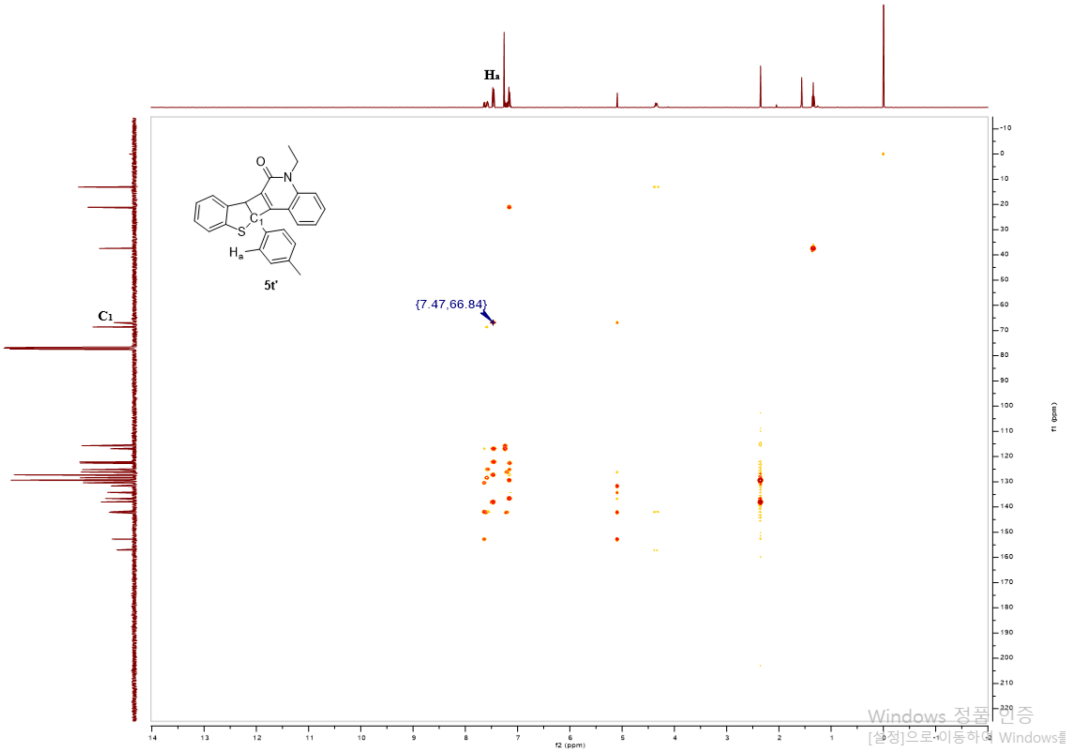

Supplementary Figure 351. HMBC of compound **5t'**.

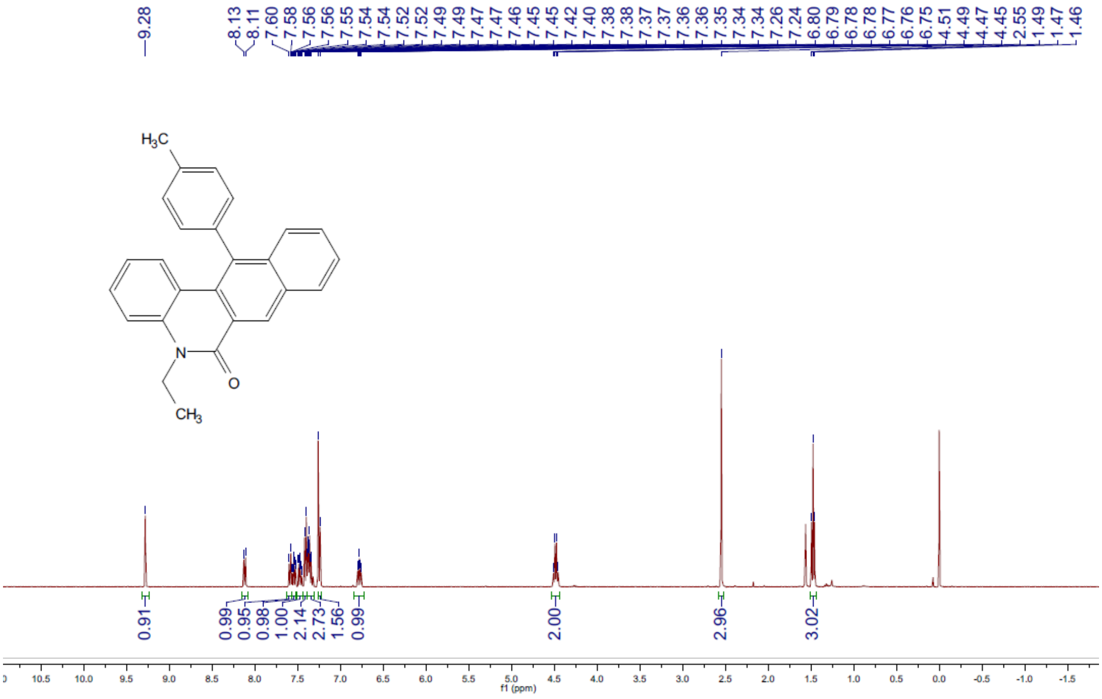

Supplementary Figure 352. ^{1}H NMR (400 MHz, $CDCl_3$) spectra of 5-ethyl-12-(*p*-tolyl)benzo[*j*]phenanthridin-6(5*H*)-one (**6t'**)

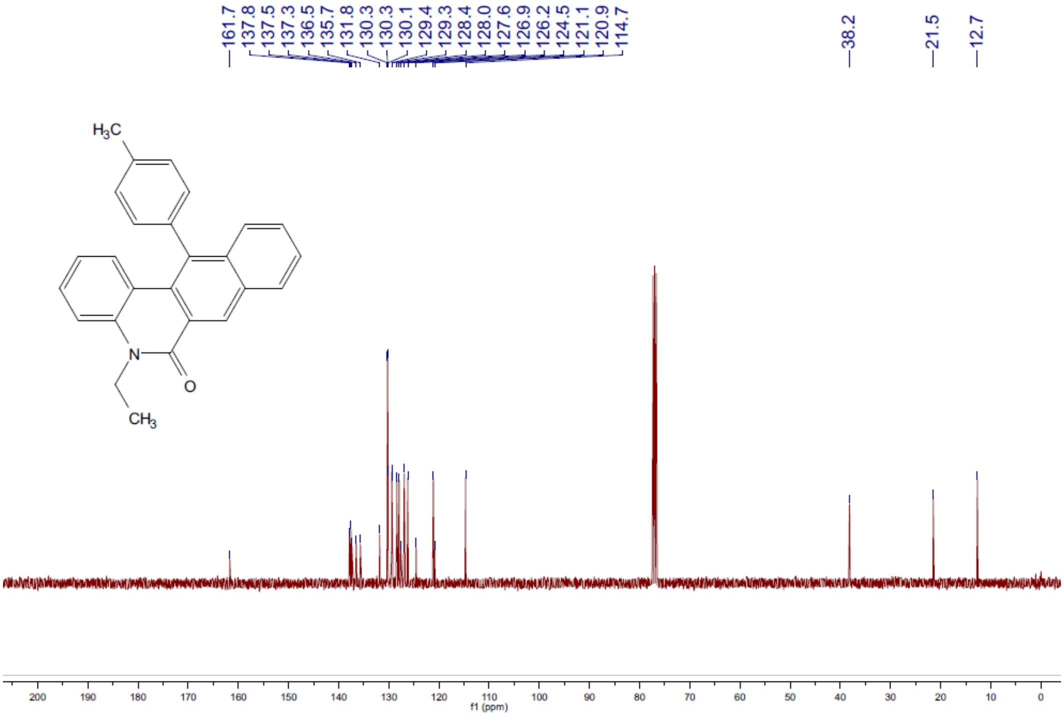

Supplementary Figure 353. ^{13}C NMR (100 MHz, $CDCl_3$) spectra of 5-ethyl-12-(*p*-tolyl)benzo[*j*]phenanthridin-6(5*H*)-one (**6t'**)

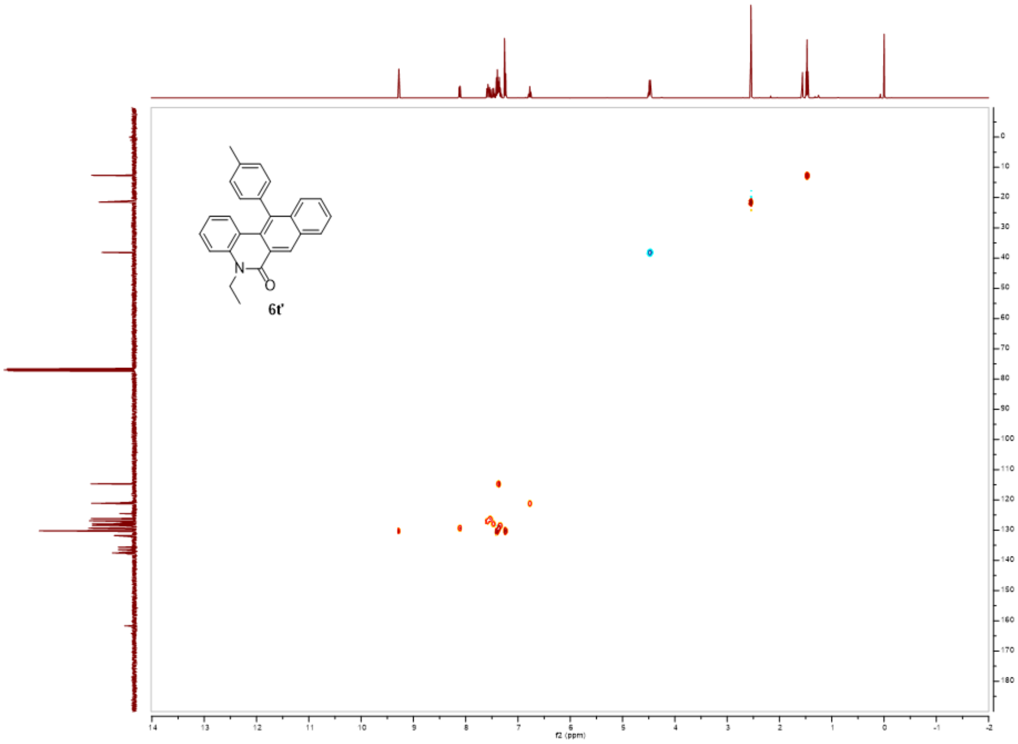

Supplementary Figure 354. HSQC of compound **6t'**.

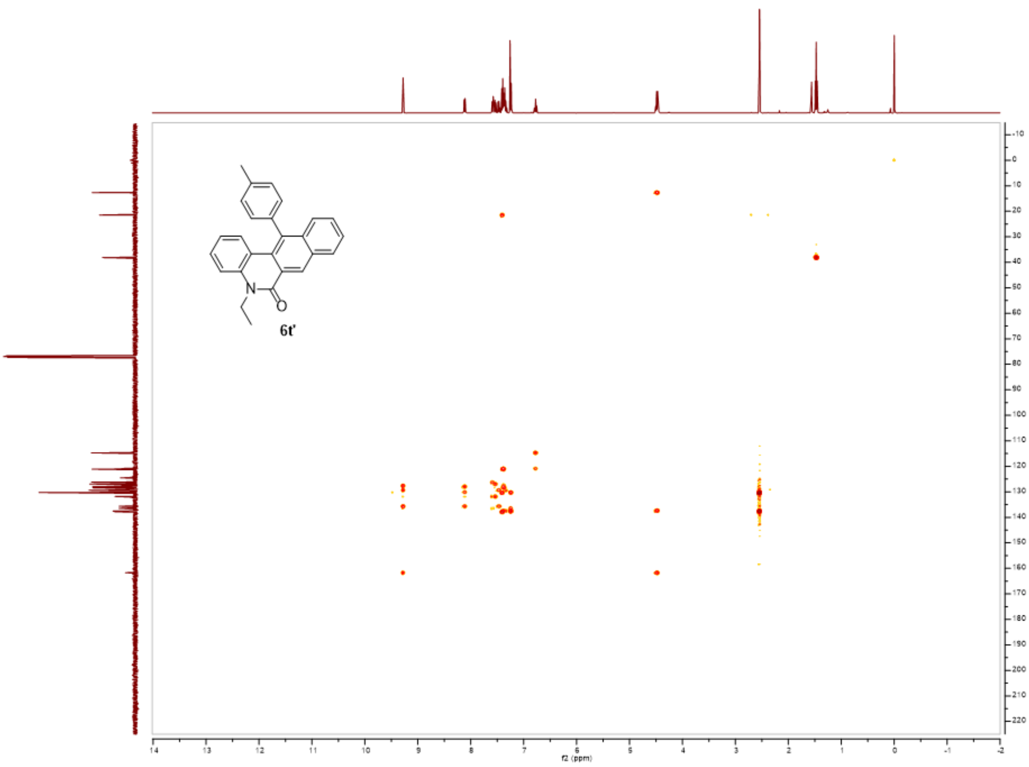

Supplementary Figure 355. HMBC of compound **6t'**.

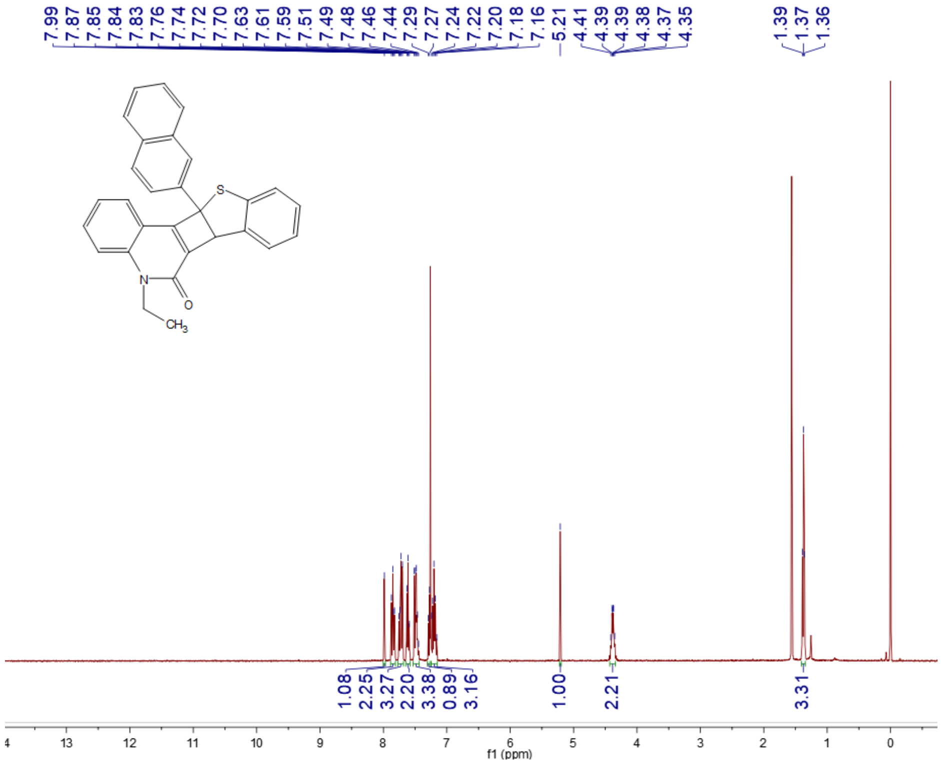

Supplementary Figure 356. ^{1}H NMR (400 MHz, $CDCl_3$) spectra of 5-ethyl-11a-(naphthalen-2-yl)-6b,11a-dihydrobenzo[4',5']thieno[2',3':3,4]cyclobuta[1,2-c]quinolin-6(5H)-one (**5u'**)

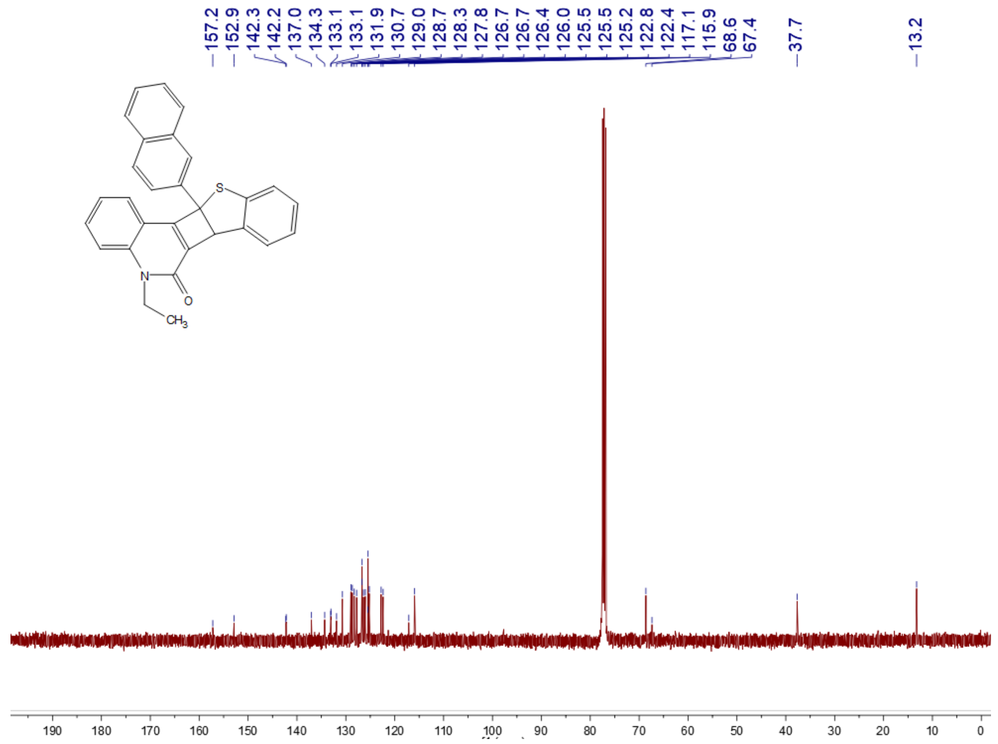

Supplementary Figure 357. ^{13}C NMR (100 MHz, CDCl3) spectra of 5-ethyl-11a-(naphthalen-2-yl)-6b,11a-dihydrobenzo[4',5']thieno[2',3':3,4]cyclobuta[1,2-c]quinolin-6(5H)-one (**5u'**)

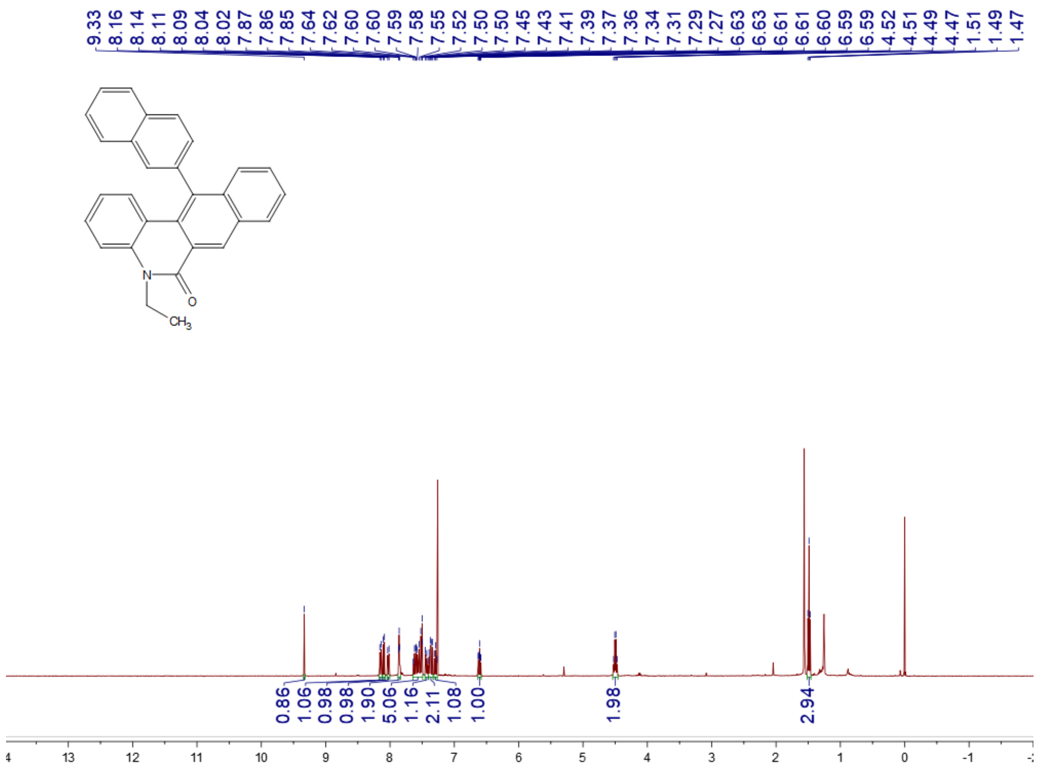

Supplementary Figure 358. ^{1}H NMR (400 MHz, $CDCl_3$) spectra of 5-ethyl-12-(naphthalen-2-yl)benzo[*j*]phenanthridin-6(5*H*)-one (**6u'**)

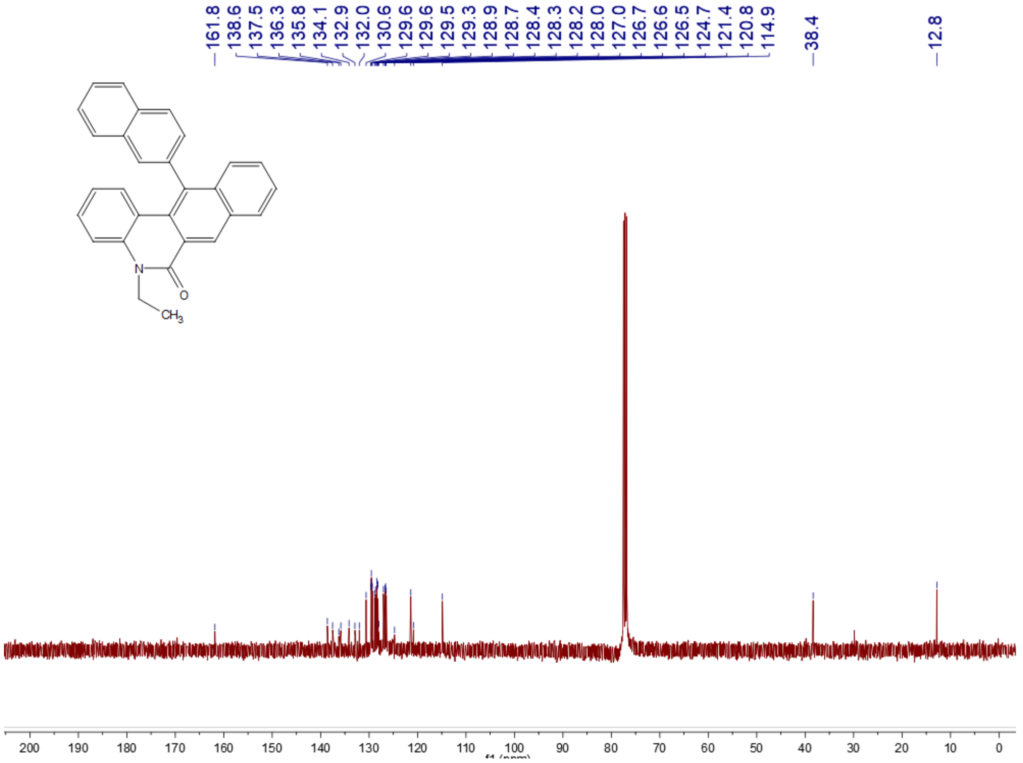

Supplementary Figure 359. ^{13}C NMR (100 MHz, $CDCl_3$) spectra of 5-ethyl-12-(naphthalen-2-yl)benzo[*j*]phenanthridin-6(5*H*)-one (**6u'**)

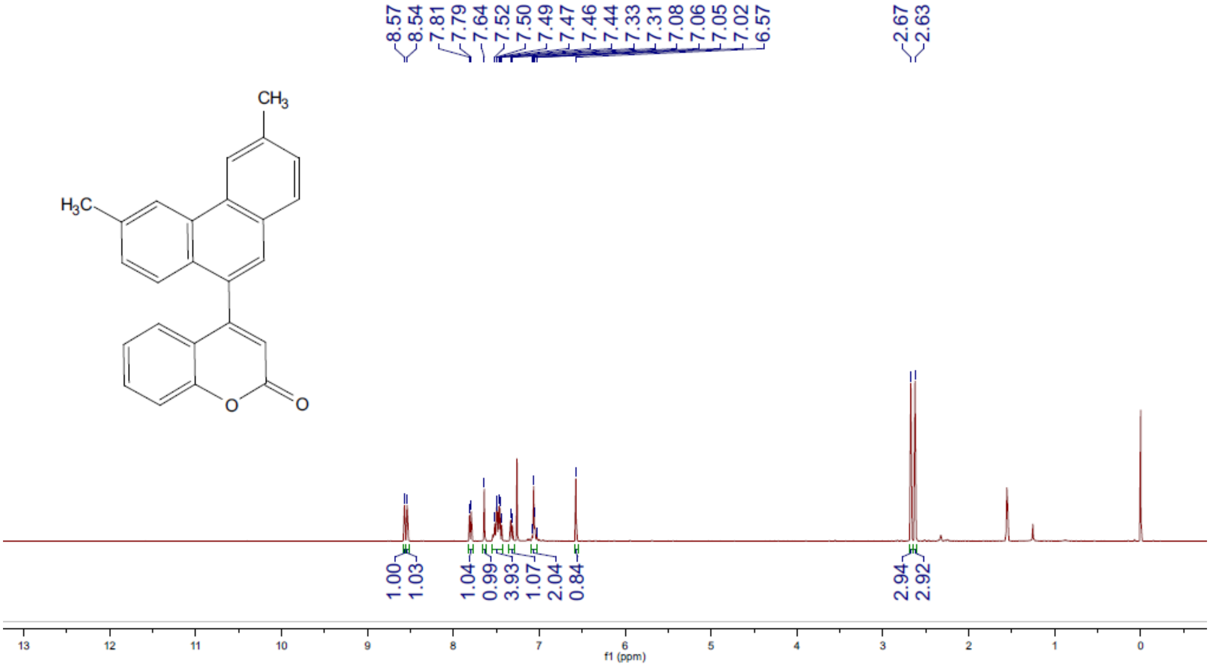

Supplementary Figure 360. ^{1}H NMR (400 MHz, $CDCl_3$) spectra of 4-(3,6-dimethylphenanthren-9-yl)-2*H*-chromen-2-one (**6a'**)

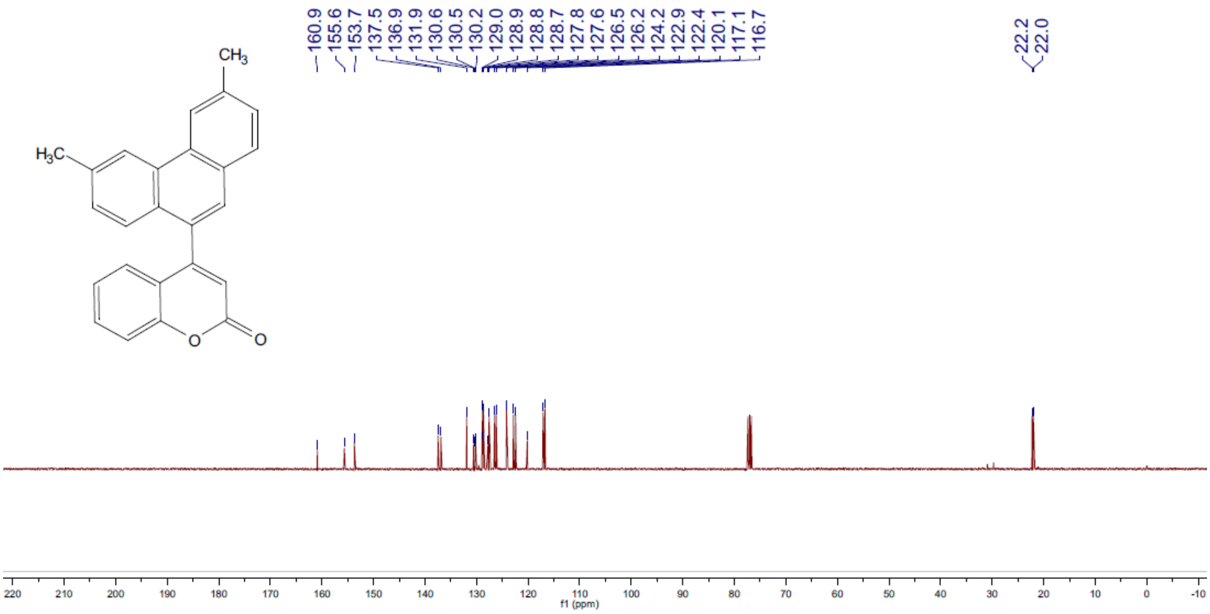

Supplementary Figure 361. ^{13}C NMR (100 MHz, $CDCl_3$) spectra of 4-(3,6-dimethylphenanthren-9-yl)-2*H*-chromen-2-one (**6a'**)

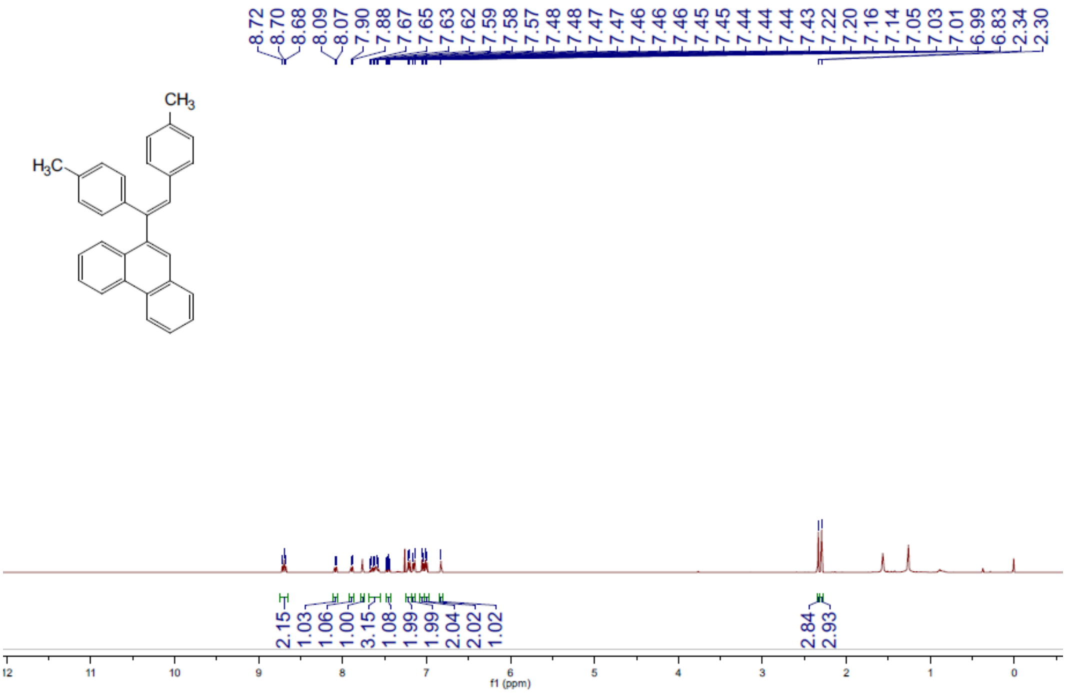

Supplementary Figure 362. ^{1}H NMR (400 MHz, $CDCl_3$) spectra of (*E*)-9-(1,2-di-*p*-tolylvinyl)phenanthrene (**6a''**)

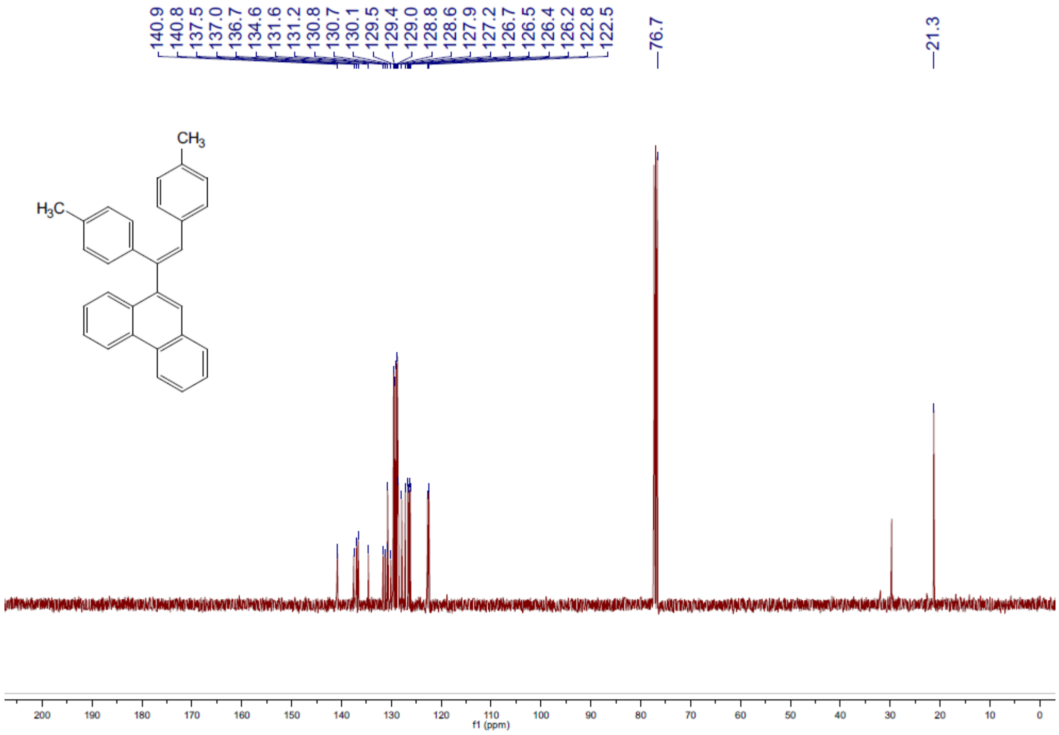

Supplementary Figure 363. ^{13}C NMR (100 MHz, $CDCl_3$) spectra of (*E*)-9-(1,2-di-*p*-tolylvinyl)phenanthrene (**6a''**)

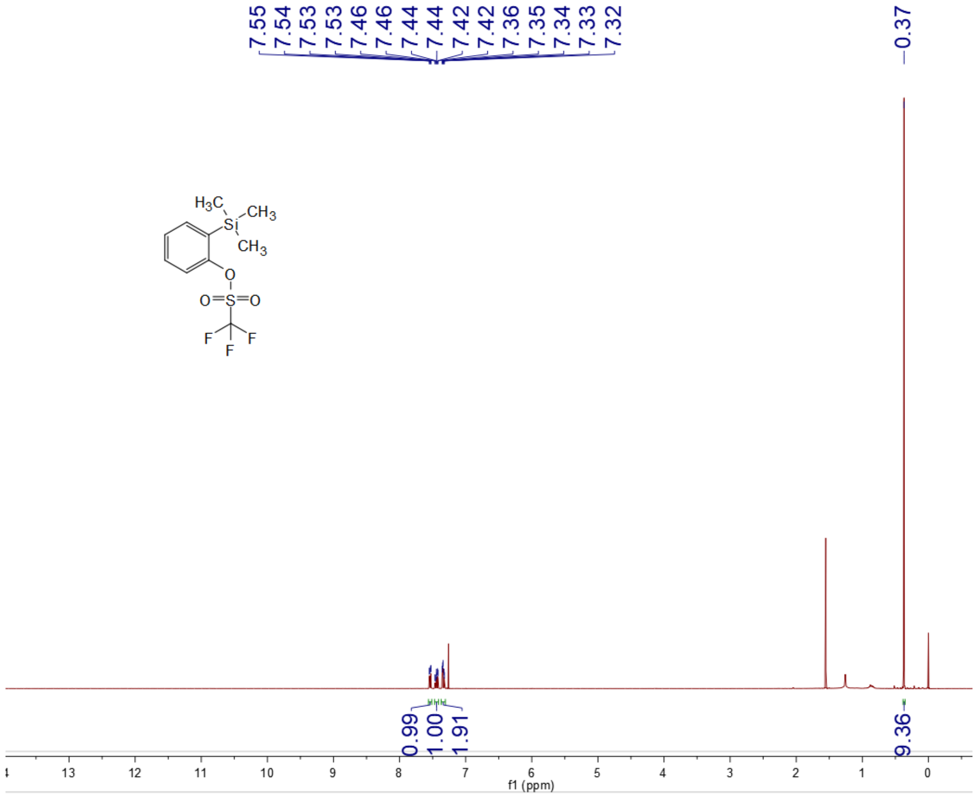

Supplementary Figure 364. ^{1}H NMR (400 MHz, $CDCl_3$) spectra of 2-(trimethylsilyl)phenyl trifluoromethanesulfonate (**S12**)

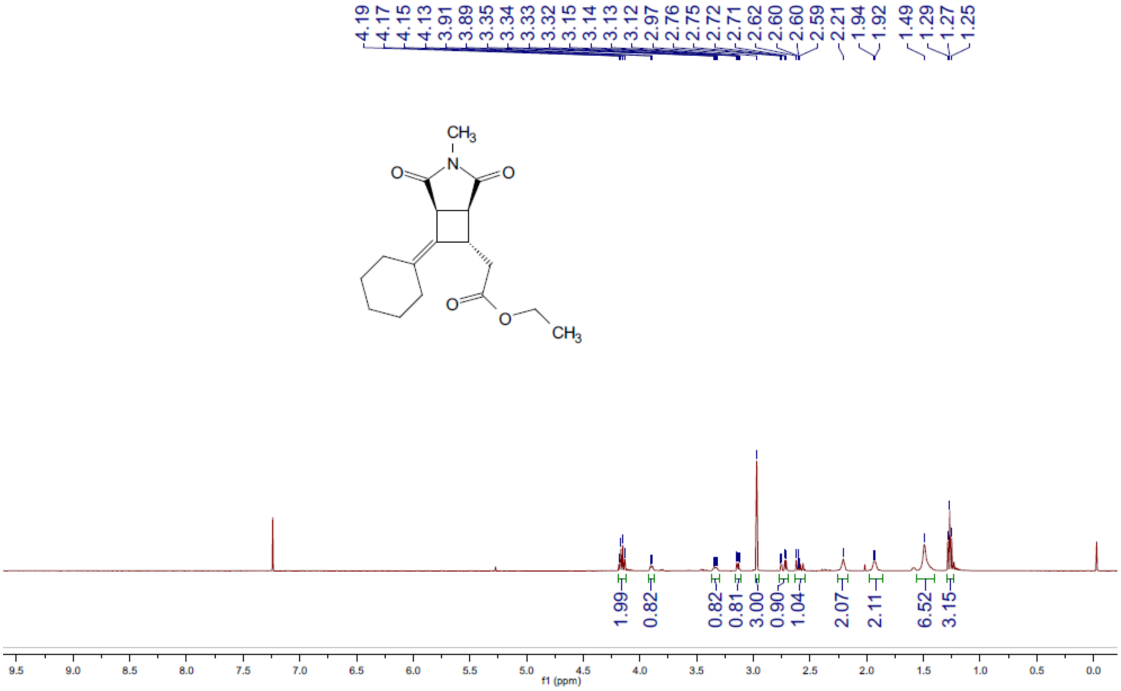

Supplementary Figure 365. ^{1}H NMR (400 MHz, $CDCl_3$) spectra of ethyl 2-(7-cyclohexylidene-3-methyl-2,4-dioxo-3-azabicyclo[3.2.0]heptan-6-yl)acetate (**7**)

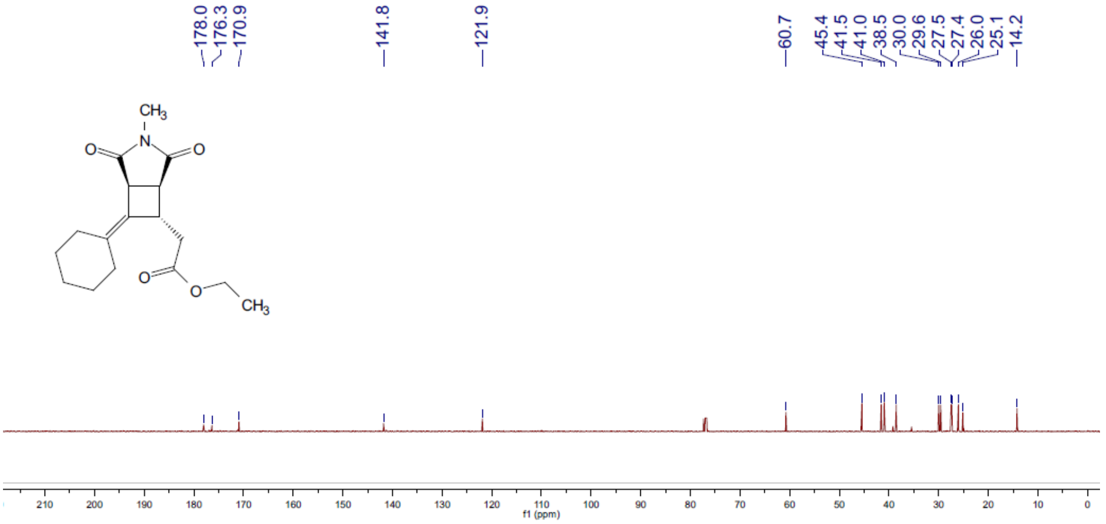

Supplementary Figure 366. ^{13}C NMR (100 MHz, $CDCl_3$) spectra of ethyl 2-(7-cyclohexylidene-3-methyl-2,4-dioxo-3-azabicyclo[3.2.0]heptan-6-yl)acetate (**7**)

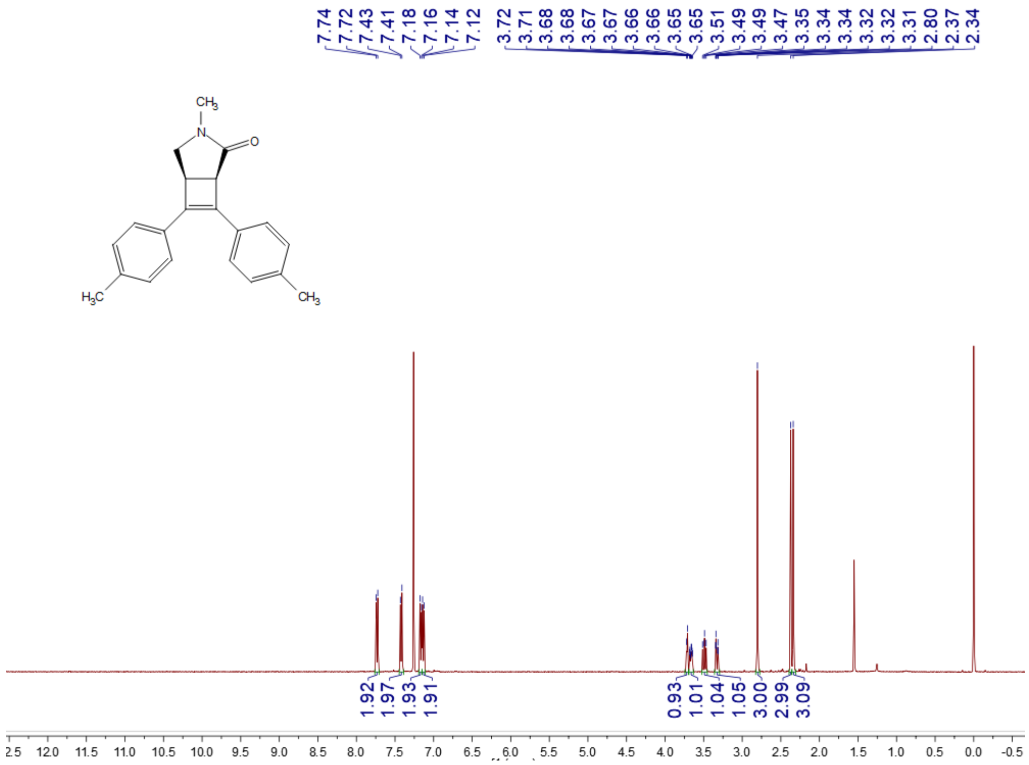

Supplementary Figure 367. ^{1}H NMR (400 MHz, $CDCl_3$) spectra of 3-methyl-6,7-di-*p*-tolyl-3-azabicyclo[3.2.0]hept-6-en-2-one (**8**)

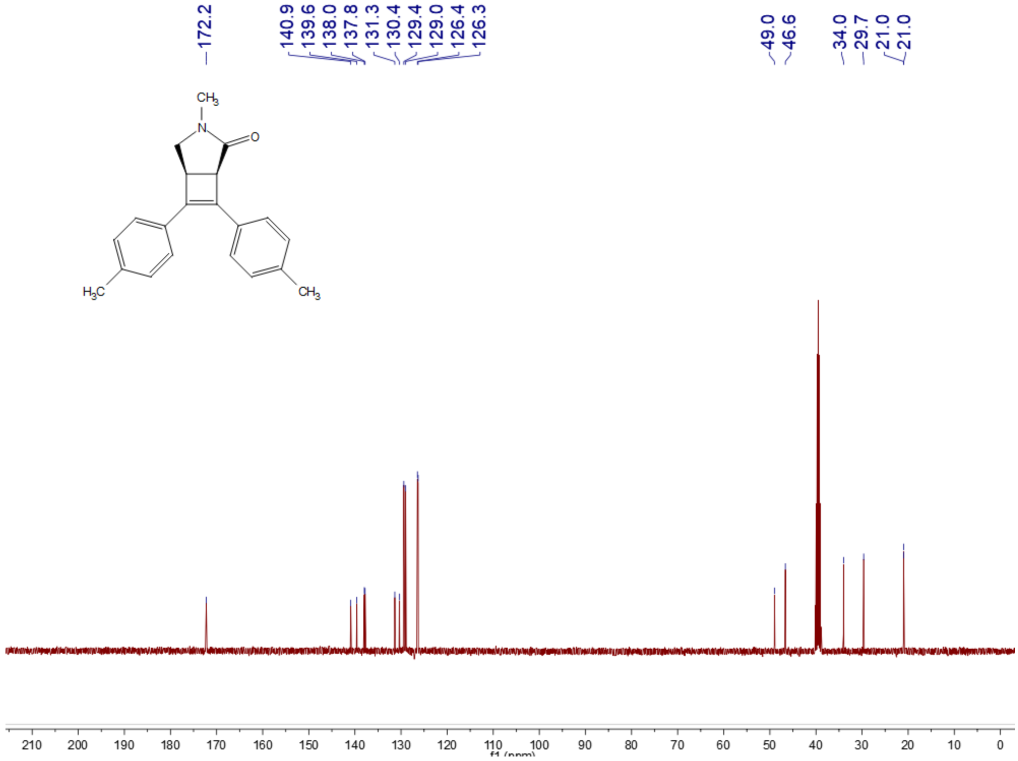

Supplementary Figure 368. ^{13}C NMR (100 MHz, $CDCl_3$) spectra of 3-methyl-6,7-di-*p*-tolyl-3-azabicyclo[3.2.0]hept-6-en-2-one (**8**)

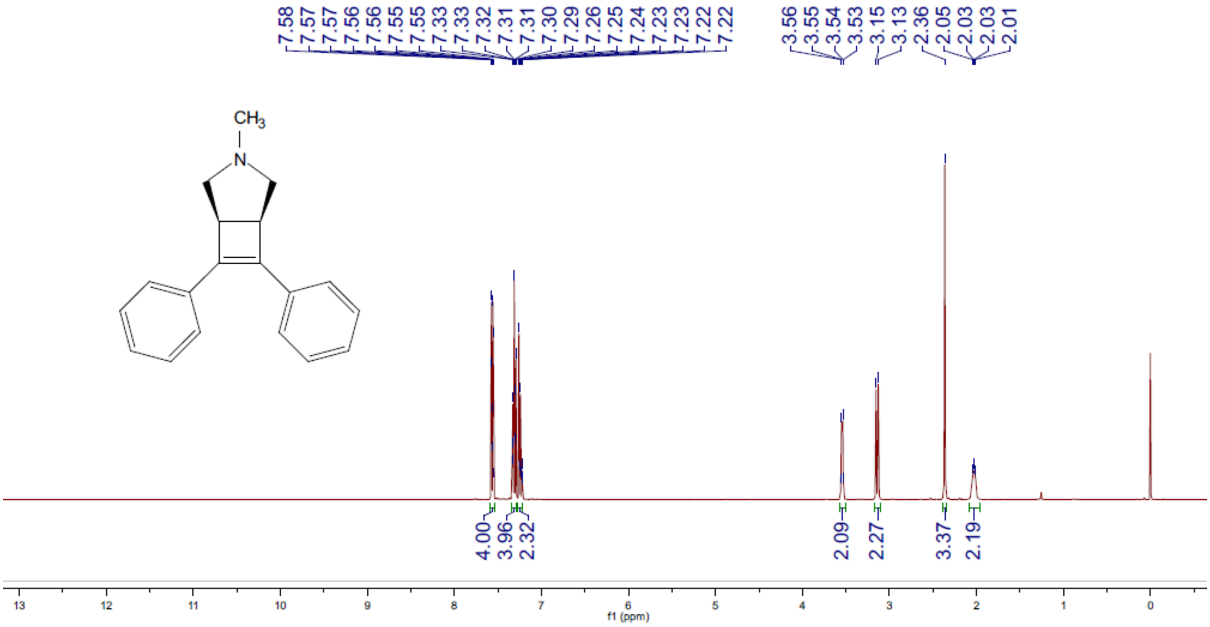

Supplementary Figure 369. ^{1}H NMR (400 MHz, $CDCl_3$) spectra of 3-methyl-6,7-diphenyl-3-azabicyclo[3.2.0]hept-6-ene (**9**)

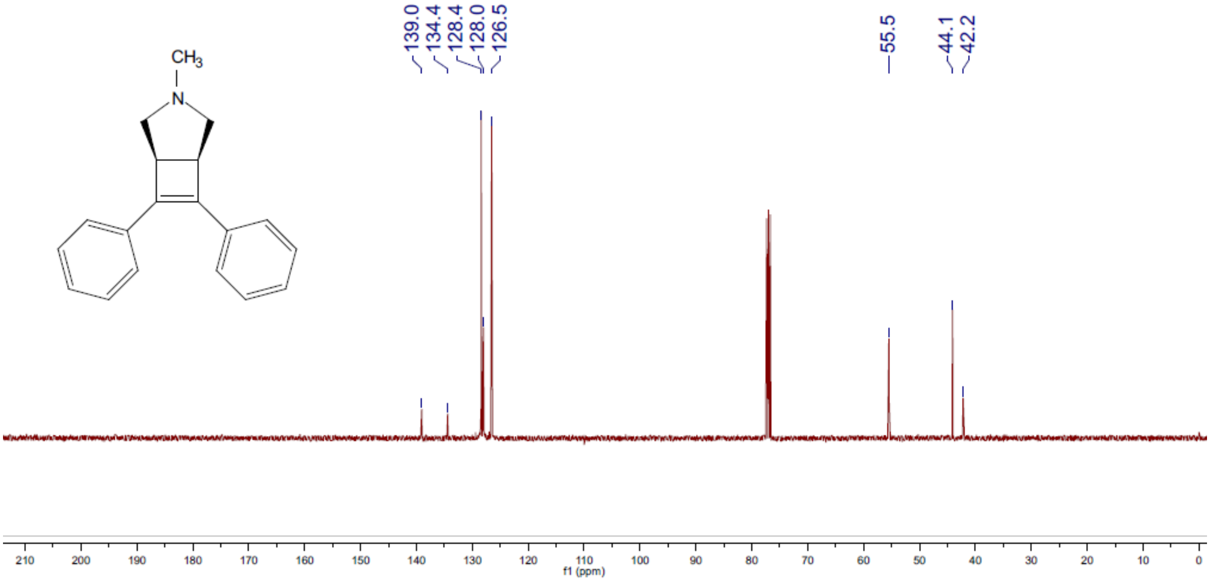

Supplementary Figure 370. ^{13}C NMR (100 MHz, $CDCl_3$) spectra of 3-methyl-6,7-diphenyl-3-azabicyclo[3.2.0]hept-6-ene (**9**)

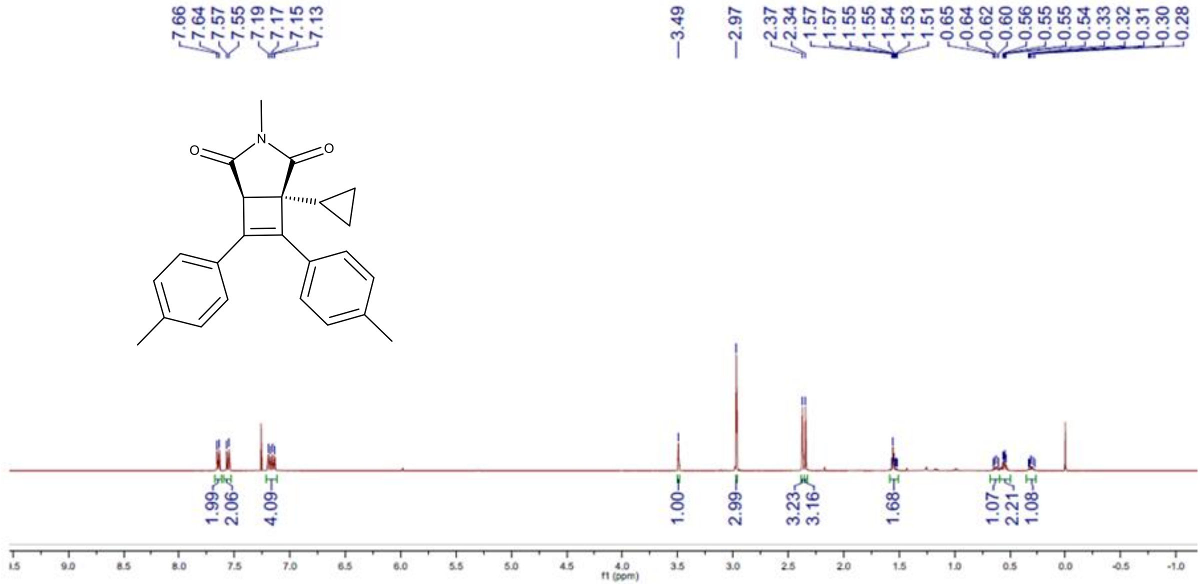

Supplementary Figure 371. ^{1}H NMR (400 MHz, $CDCl_3$) spectra of 1-cyclopropyl-3-methyl-6,7-di-*p*-tolyl-3-azabicyclo[3.2.0]hept-6-ene-2,4-dione (**3ay)**

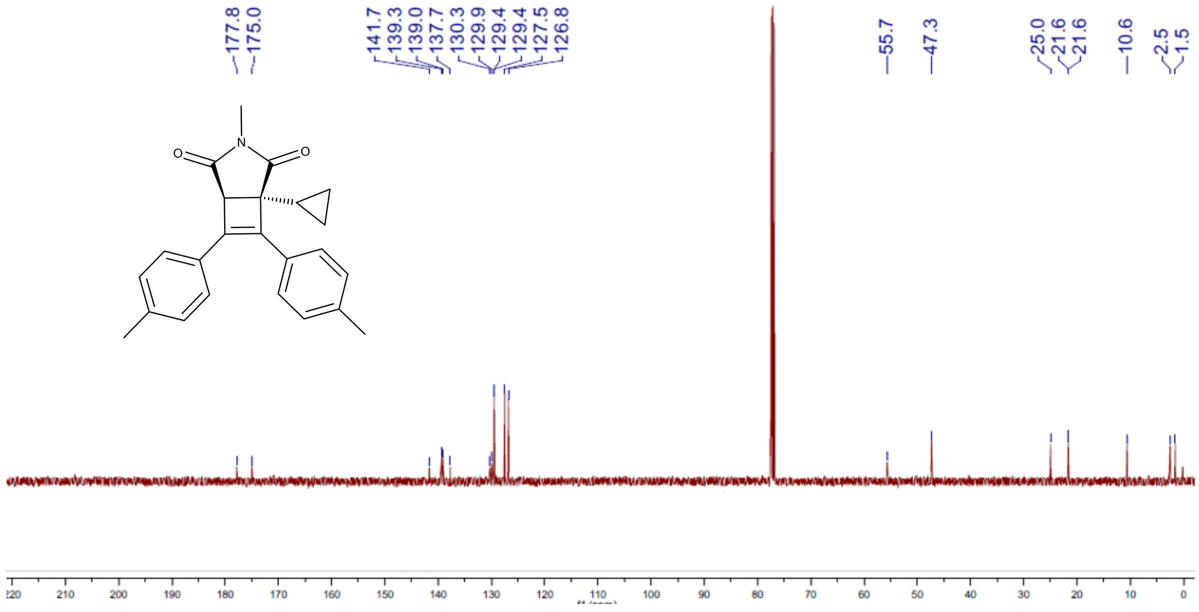

Supplementary Figure 372. ^{13}C NMR (100 MHz, $CDCl_3$) spectra of 1-cyclopropyl-3-methyl-6,7-di-*p*-tolyl-3-azabicyclo[3.2.0]hept-6-ene-2,4-dione (**3ay**)

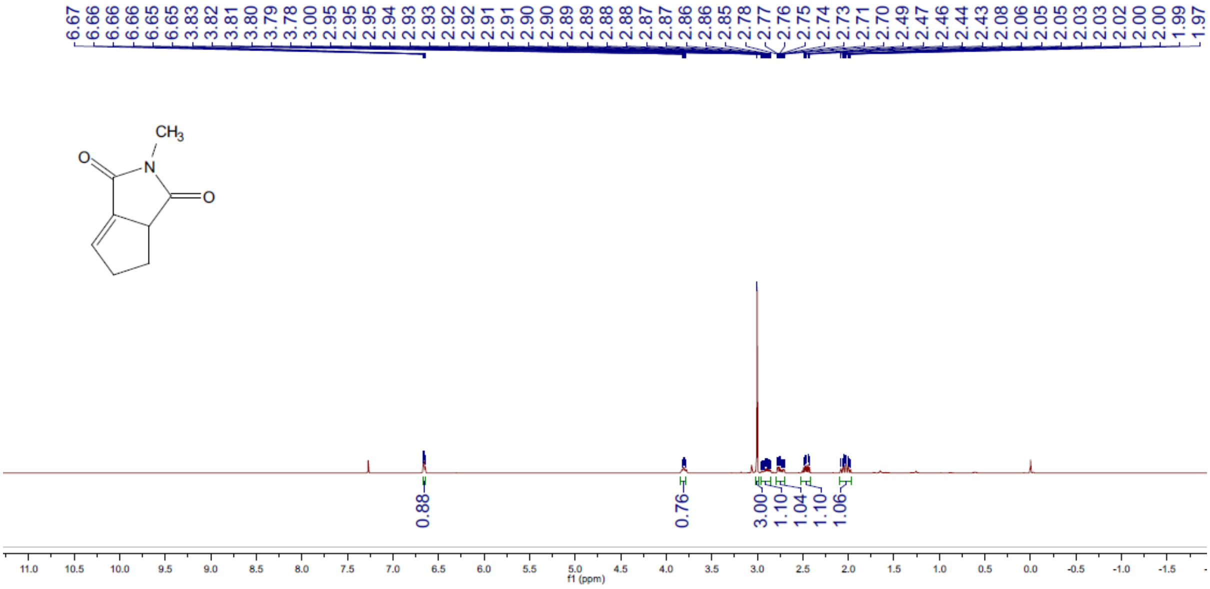

Supplementary Figure 373. ^{1}H NMR (400 MHz, $CDCl_3$) spectra of 2-methyl-4,5-dihydrocyclopenta[*c*]pyrrole-1,3(2*H*,3a*H*)-dione (**2y'**)

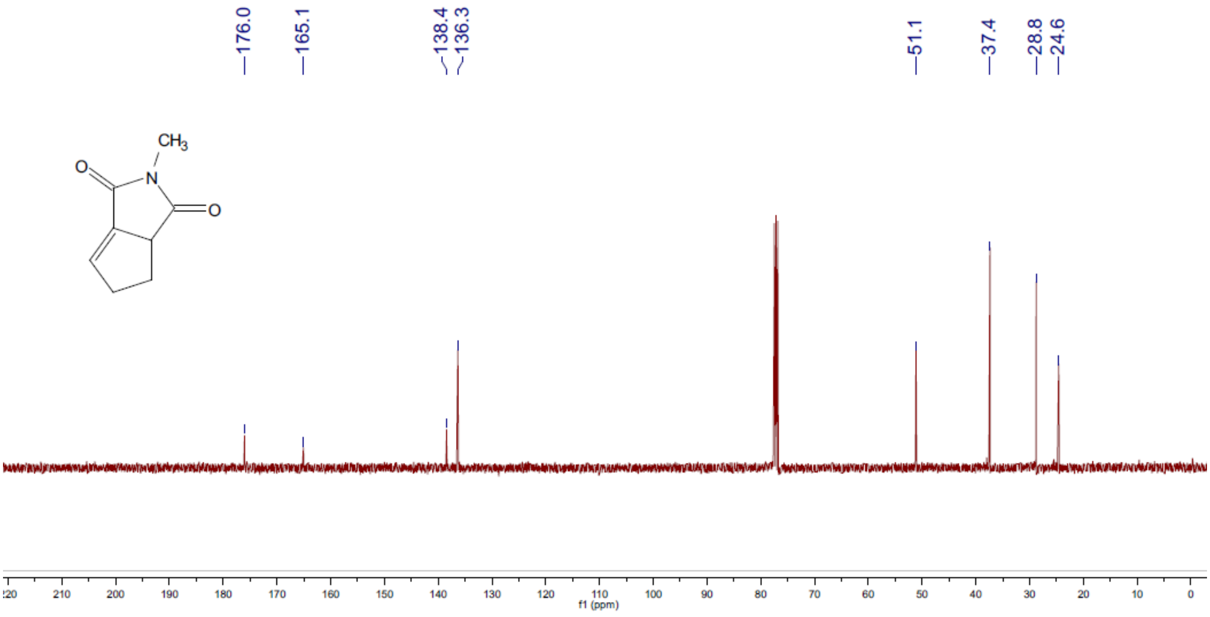

Supplementary Figure 374. ^{13}C NMR (100 MHz, $CDCl_3$) spectra of 2-methyl-4,5-dihydrocyclopenta[*c*]pyrrole-1,3(2*H*,3a*H*)-dione (**2y'**)

Supplementary References

1. Monos TM, Sun AC, McAtee RC, Devery JJ, Stephenson CRJ. Microwave-Assisted Synthesis of Heteroleptic Ir(III)+ Polypyridyl Complexes. *J. Org. Chem.* **81**, 6988-6994 (2016).

2. Zhou Y, Li W, Liu Y, Zeng L, Su W, Zhou M. Substituent effect of ancillary ligands on the luminescence of bis[4,6-(di-fluorophenyl)-pyridinato-N,C2']iridium(III) complexes. *Dalton Trans* **41**, 9373-9381 (2012).

3. Xiao X, Sakamoto J, Tanabe M, Yamazaki S, Yamabe S, Matsumura-Inoue T. Microwave synthesis and electrospectrochemical study on ruthenium(II) polypyridine complexes. *J. Electroanal. Chem.* **527**, 33-40 (2002).

4. Rapenne G, Jimenez-Bueno G. Molecular machines: synthesis and characterization of two prototypes of molecular wheelbarrows. *Tetrahedron* **63**, 7018-7026 (2007).

5. Zhang X*, et al.* Rhodium-Catalyzed Oxidative Benzannulation of N-Adamantyl-1-naphthylamines with Internal Alkynes via Dual C–H Bond Activation: Synthesis of Substituted Anthracenes. *Org. Lett.* **18**, 4246-4249 (2016).

6. Fu R, Li Z. Direct Synthesis of Symmetric Diarylethynes from Calcium Carbide and Arylboronic Acids/Esters. *Eur. J. Org. Chem.* **2017**, 6648-6651 (2017).

7. Park K, Bae G, Moon J, Choe J, Song KH, Lee S. Synthesis of Symmetrical and Unsymmetrical Diarylalkynes from Propiolic Acid Using Palladium-Catalyzed Decarboxylative Coupling. *J. Org. Chem.* **75**, 6244-6251 (2010).

8. Kalvet I, Magnin G, Schoenebeck F. Rapid Room-Temperature, Chemoselective C −C Coupling of Poly(pseudo)halogenated Arenes Enabled by Palladium(I) Catalysis in Air. *Angew. Chem. Int. Ed.* **56**, 1581-1585 (2017).

9. Chen M, Su N, Deng T, Wink DJ, Zhao Y, Driver TG. Controlling the Selectivity Patterns of Au-Catalyzed Cyclization–Migration Reactions. *Org. Lett.* **21**, 1555-1558 (2019).

10. Swamy NK, Yazici A, Pyne SG. Copper-Mediated Cyclization−Halogenation and Cyclization−Cyanation Reactions of β-Hydroxyalkynes and o-Alkynylphenols and Anilines. *J. Org. Chem.* **75**, 3412-3419 (2010).

11. Huang H, Jiang H, Chen K, Liu H. Efficient Iron/Copper Cocatalyzed Alkynylation of Aryl Iodides with Terminal Alkynes. *J. Org. Chem.* **73**, 9061-9064 (2008).

12. Jyothish K, Zhang W. Introducing A Podand Motif to Alkyne Metathesis Catalyst Design: A Highly Active Multidentate Molybdenum(VI) Catalyst that Resists Alkyne Polymerization. *Angew. Chem. Int. Ed.* **50**, 3435-3438 (2011).

13. Holmes BT, Pennington WT, Hanks TW. Efficient Synthesis of a Complete Donor/Acceptorbis(Aryl)diyne Family. *Synth. Commun.* **33**, 2447-2461 (2003).

14. Li C-W, Pati K, Lin G-Y, Sohel SMA, Hung H-H, Liu R-S. Gold-Catalyzed Oxidative Ring Expansions and Ring Cleavages of Alkynylcyclopropanes by Intermolecular Reactions Oxidized by Diphenylsulfoxide. *Angew. Chem. Int. Ed.* **49**, 9891-9894 (2010).

15. Jansone-Popova S, May JA. Synthesis of bridged polycyclic ring systems via carbene cascades terminating in C-H bond insertion. *J. Am. Chem. Soc.* **134**, 17877-17880 (2012).

16. Ahn DK, Kang YW, Woo SK. Oxidative Deprotection of p-Methoxybenzyl Ethers via Metal-Free Photoredox Catalysis. *J. Org. Chem.* **84**, 3612-3623 (2019).

17. Hill RK, Sawada S, Arfin SM. Stereochemistry of valine and isoleucine biosynthesis: IV. Synthesis, configuration, and enzymatic specificity of α-acetolactate and α-aceto-α-hydroxybutyrate. *Bioorg. Chem.* **8**, 175-189 (1979).

18. Azizi S*, et al.* Photoinduced proton transfer promoted by peripheral subunits for some Hantzsch esters. *J Phys Chem A* **119**, 39-49 (2015).

19. Sahani RL, Liu RS. Development of Gold-catalyzed [4+1] and [2+2+1]/[4+2] Annulations between Propiolate Derivatives and Isoxazoles. *Angew. Chem. Int. Ed.* **56**, 1026-1030 (2017).

20. Stulgies B*, et al.* Six- and eightfold palladium-catalyzed cross-coupling reactions of hexa- and octabromoarenes. *Chemistry* **11**, 308-320 (2004).

21. Suárez JR, Collado-Sanz D, Cárdenas DJ, Chiara JL. Nonafluorobutanesulfonyl Azide as a Shelf-Stable Highly Reactive Oxidant for the Copper-Catalyzed Synthesis of 1,3-Diynes from Terminal Alkynes. *J. Org. Chem.* **80**, 1098-1106 (2015).

22. Enyong AB, Moasser B. Ruthenium-Catalyzed N-Alkylation of Amines with Alcohols under Mild Conditions Using the Borrowing Hydrogen Methodology. *J. Org. Chem.* **79**, 7553-7563 (2014).

23. Choi J-H, Park C-M. Three-Component Synthesis of Quinolines Based on Radical Cascade Visible-Light Photoredox Catalysis. *Adv. Synth. Catal.* **360**, 3553-3562 (2018).

24. Burgess KL, Lajkiewicz NJ, Sanyal A, Yan W, Snyder JK. A New Chiral Anthracene for the Asymmetric Diels−Alder/Retro-Diels−Alder Sequence. *Org. Lett.* **7**, 31-34 (2005).

25. Issa F, Fischer J, Turner P, Coster MJ. Regioselective Reduction of 3-Methoxymaleimides: An Efficient Method for the Synthesis of Methyl 5-Hydroxytetramates. *J. Org. Chem.* **71**, 4703-4705 (2006).

26. Otohiko T, Shuji K, Satoru K, Shigeori T. NEW C–C BOND FORMATION WITH PYRIDINIUM METHYLIDE: HYDROMETHYLENATION OF OLEFIN. *Chem. Lett.* **13**, 281-284 (1984).

27. Zou C, Zeng C, Liu Z, Lu M, Sun X, Ye J. γ′-Selective Functionalization of Cyclic Enones: Construction of a Chiral Quaternary Carbon Center by [4+2] Cycloaddition/Retro-Mannich Reaction with 3-Substituted Maleimides. *Angew. Chem. Int. Ed.* **55**, 14257-14261 (2016).

28. Hegazy M-EF, Shishido K, Hirata T. Asymmetric hydrogenation of the C–C double bond of 1- and 1,2-methylated maleimides with cultured suspension cells of Marchantia polymorpha. *Tetrahedron: Asymmetry* **17**, 1859-1862 (2006).

29. De Sarkar S, Ackermann L. Ruthenium(II)-Catalyzed C H Activation with Isocyanates: A Versatile Route to Phthalimides. *Chem. Eur. J.* **20**, 13932-13936 (2014).

30. Schobert R, Barnickel B. A Regioselective Tsuji-Trost Pentadienylation of 3-Allyltetronic Acid. *Synthesis* **2009**, 2778-2784 (2009).

31. Gill GB, James GD, Oates KV, Pattenden G. The synthesis of 5-ylidenepyrrol-2(5H)-ones from maleimides and from pyrrol-2-(5H)-ones. *J. Chem. Soc., Perkin Trans. 1*, 2567-2579 (1993).

32. Lood CS, Laine AE, Högnäsbacka A, Nieger M, Koskinen AMP. Synthesis of Chiral (Indol-2-yl)methanamines and Insight into the Stereochemistry Protecting Effects of the 9-Phenyl-9-fluorenyl Protecting Group. *Eur. J. Org. Chem.* **2015**, 3793-3805 (2015).

33. Wolfe RM, Reynolds JR. Direct Imide Formation from Thiophene Dicarboxylic Acids Gives Expanded Side-Chain Selection in Thienopyrrolediones. *Org. Lett.* **19**, 996-999 (2017).

34. Knapp DM, Gillis EP, Burke MD. A General Solution for Unstable Boronic Acids: Slow-Release Cross-Coupling from Air-Stable MIDA Boronates. *J. Am. Chem. Soc.* **131**, 6961-6963 (2009).

35. Lee E, Ryu T, Park Y, Park S, Lee PH. Tandem Gold-Catalyzed Hydrosilyloxylation–Aldol and –Mannich Reaction with Alkynylaryloxysilanols in 6-exo Mode. *Adv. Synth. Catal.* **355**, 1585-1596 (2013).

36. Álvarez R, Martínez C, Madich Y, Denis JG, Aurrecoechea JM, de Lera ÁR. Corrigendum: A General Synthesis of Alkenyl-Substituted Benzofurans, Indoles, and Isoquinolones by Cascade Palladium-Catalyzed Heterocyclization/Oxidative Heck Coupling. *Chem. Eur. J.* **18**, 13894-13896 (2012).

37. Tsefrikas VM, Greene AK, Scott LT. 5-Azadibenzo[a,g]corannulene. *Org. Chem. Front.* **4**, 688-698 (2017).

38. Yanada R*, et al.* Indium(III)-Catalyzed Tandem Reaction with Alkynylbenzaldehydes and Alkynylanilines to Heteroaromatic Compounds. *J. Org. Chem.* **73**, 5135-5138 (2008).

39. Alfonsi M, Arcadi A, Aschi M, Bianchi G, Marinelli F. Gold-Catalyzed Reactions of 2-Alkynyl-phenylamines with α,β-Enones. *J. Org. Chem.* **70**, 2265-2273 (2005).

40. Yang X, Wu D, Lu Z, Sun H, Li A. A mild preparation of alkynes from alkenyl triflates. *Org Biomol Chem* **14**, 5591-5594 (2016).

41. Qiao Y, Yang Q, Schelter EJ. Photoinduced Miyaura Borylation by a Rare-Earth-Metal Photoreductant: The Hexachlorocerate(III) Anion. *Angew. Chem. Int. Ed.* **57**, 10999-11003 (2018).

42. Ganesan B, Senadi GC, Guo B-C, Hung M-Y, Lin W-Y. A copper(ii)-catalyzed annulative formylation

of o-alkynylanilines with DMF: a single-step strategy for 3-formyl indoles. *RSC Advances* **8**, 40968-40973 (2018).

43. Lu X*, et al.* A multi-functional probe to discriminate Lys, Arg, His, Cys, Hcy and GSH from common amino acids. *Chem. Commun.* **51**, 1498-1501 (2015).

44. SWAMY PV*, et al.* Synthesis, biological evaluation and molecular docking studies of some novel cyclopropane carbohydrazide derivatives as potential anticancer agents. *J. Chem. Sci.* **128**, 929-939 (2016).

45. Gao P, Liu L, Shi Z, Yuan Y. Iridium(iii)-catalyzed regioselective direct arylation of sp2 C–H bonds with diaryliodonium salts. *Org. Biomol. Chem.* **14**, 7109-7113 (2016).

46. Cooper MA, Lucas MA, Taylor JM, Ward AD, Williamson NM. A Convenient Method for the Aromatic Amino-Claisen Rearrangement of N-(1,1-Disubstituted-allyl)anilines. *Synthesis* **2001**, 0621-0625 (2001).

47. Cabrera-Lobera N, Rodríguez-Salamanca P, Nieto-Carmona JC, Buñuel E, Cárdenas DJ. Iron-Catalyzed Hydroborylative Cyclization of 1,6-Enynes. *Chem. Eur. J.* **24**, 784-788 (2018).

48. Domingo-Legarda P, Soler-Yanes R, Quirós-López MT, Buñuel E, Cárdenas DJ. Iron-Catalyzed Coupling of Propargyl Bromides and Alkyl Grignard Reagents. *Eur. J. Org. Chem.* **2018**, 4900-4904 (2018).

49. Zhou X, Jiang Z, Xue L, Lu P, Wang Y. Preparation of 1,2,5-Trisubstituted 1H-Imidazoles from Ketenimines and Prop-arg-ylic Amines by Silver-Catalyzed or Iodine-Promoted Electrophilic Cyclization Reaction of Alkynes. *Eur. J. Org. Chem.* **2015**, 5789-5797 (2015).

50. Ueno A, Takimoto M, O WWN, Nishiura M, Ikariya T, Hou Z. Copper-Catalyzed Formal C H Carboxylation of Aromatic Compounds with Carbon Dioxide through Arylaluminum Intermediates. *Chem. Asian J.* **10**, 1010-1016 (2015).

51. Xiao Z-F, Ding T-H, Mao S-W, Ning X-S, Kang Y-B. Zinc Iodide-Mediated Direct Synthesis of 2,3-Dihydroisoxazoles from Alkynes and Nitrones. *Adv. Synth. Catal.* **358**, 1859-1863 (2016).

52. Li X, Sun Y, Huang X, Zhang L, Kong L, Peng B. Synthesis of o-Aryloxy Triarylsulfonium Salts via Aryne Insertion into Diaryl Sulfoxides. *Org. Lett.* **19**, 838-841 (2017).

53. Cismesia MA, Yoon TP. Characterizing chain processes in visible light photoredox catalysis. *Chem. Sci.* **6**, 5426-5434 (2015).

54. Frisch MJ*, et al.* Gaussian 09, Revision D.01. (2013).

55. Zhao Y, Truhlar DG. The M06 suite of density functionals for main group thermochemistry, thermochemical kinetics, noncovalent interactions, excited states, and transition elements: two new functionals and systematic testing of four M06-class functionals and 12 other functionals. *Theor. Chem. Acc.* **120**, 215-241 (2008).

56. Clark T, Chandrasekhar J, Spitznagel GW, Schleyer PVR. Efficient diffuse function-augmented basis sets for anion calculations. III. The 3-21+G basis set for first-row elements, Li–F. *J. Comput. Chem.* **4**, 294-301 (1983).

57. Krishnan R, Binkley JS, Seeger R, Pople JA. Self-consistent molecular orbital methods. XX. A basis set for correlated wave functions. *J. Chem. Phys.* **72**, 650-654 (1980).

58. Schuchardt KL*, et al.* Basis Set Exchange: A Community Database for Computational Sciences. *J. Chem. Inf. Model.* **47**, 1045-1052 (2007).

59. Feller D. The role of databases in support of computational chemistry calculations. *J. Comput. Chem.* **17**, 1571-1586 (1996).

60. Marenich AV, Cramer CJ, Truhlar DG. Universal Solvation Model Based on Solute Electron Density and on a Continuum Model of the Solvent Defined by the Bulk Dielectric Constant and Atomic Surface Tensions. *J. Phys.Chem. B* **113**, 6378-6396 (2009).

61. Fukui K. The path of chemical reactions - the IRC approach. *Acc. Chem. Res.* **14**, 363-368 (1981).

62. Fukui K. Formulation of the reaction coordinate. *J. Phys. Chem.* **74**, 4161-4163 (1970).

63. Zhao J*, et al.* Intramolecular Crossed [2+2] Photocycloaddition through Visible Light-Induced Energy Transfer. *J. Am. Chem. Soc.* **139**, 9807-9810 (2017).

64. James MJ, Schwarz JL, Strieth-Kalthoff F, Wibbeling B, Glorius F. Dearomative Cascade Photocatalysis: Divergent Synthesis through Catalyst Selective Energy Transfer. *J. Am. Chem. Soc.* **140**, 8624-8628 (2018).

65. Blum TR, Miller ZD, Bates DM, Guzei IA, Yoon TP. Enantioselective photochemistry through Lewis acid–catalyzed triplet energy transfer. *Science* **354**, 1391-1395 (2016).

66. Luis-Barrera J*, et al.* Visible-Light Photocatalytic Intramolecular Cyclopropane Ring Expansion. *Angew. Chem. Int. Ed.* **56**, 7826-7830 (2017).
