# Supplementary material for: Alkyne–Alkene [2 + 2] cycloaddition based on visible light photocatalysis
Source: Nat Commun. 2020 May 19;11:2509. doi: 10.1038/s41467-020-16283-9 (PMC7237675; doi:10.1038/s41467-020-16283-9)
Supplement: Supplementary file 3 — Description of Additional Supplementary Files [file 41467_2020_16283_MOESM3_ESM.pdf]

Description of Additional Supplementary Files

File Name: Supplementary Data 1
Description: Cartesian coordinates for the DFT calculations.
