# Supplementary material for: Alkyne–Alkene [2 + 2] cycloaddition based on visible light photocatalysis
Source: Nat Commun. 2020 May 19;11:2509. doi: 10.1038/s41467-020-16283-9 (PMC7237675; doi:10.1038/s41467-020-16283-9)
Supplement: Supplementary file 4 — Supplementary Data 1 [file 41467_2020_16283_MOESM4_ESM.zip › 228896_2_data_set_4558614_q8xznq.docx]

Structures and coordinates of optimized geometries


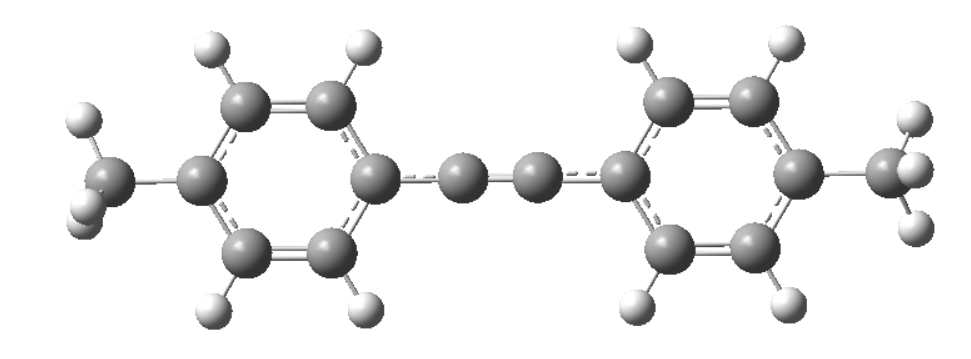


| C | -4.125453 | 1.199611 | -0.006764 |
| --- | --- | --- | --- |
| C | -4.843566 | 0.004532 | -0.006482 |
| C | -4.126723 | -1.194158 | -0.005069 |
| C | -2.743983 | -1.202831 | 0.000356 |
| C | -2.026579 | 0.001444 | 0.002056 |
| C | -2.740374 | 1.205291 | -0.001720 |
| C | -6.338566 | -0.002653 | 0.010054 |
| C | -0.604730 | -0.000414 | 0.002945 |
| C | 0.604731 | -0.001764 | 0.002898 |
| C | 2.026581 | -0.002371 | 0.001995 |
| C | 2.741850 | -1.205788 | -0.002461 |
| C | 4.126509 | -1.198610 | -0.008654 |
| C | 4.843593 | -0.002369 | -0.008159 |
| C | 4.125673 | 1.195152 | -0.006838 |
| C | 2.742525 | 1.202312 | -0.000416 |
| C | 6.338576 | 0.003339 | 0.012661 |
| H | -4.665052 | 2.143815 | -0.012621 |
| H | -4.669034 | -2.137267 | -0.008704 |
| H | -2.201805 | -2.144203 | 0.000148 |
| H | -2.197000 | 2.145944 | -0.003742 |
| H | -6.746944 | 0.981642 | -0.235403 |
| H | -6.742722 | -0.729657 | -0.702273 |
| H | -6.718927 | -0.281995 | 1.000118 |
| H | 2.199353 | -2.146952 | -0.005048 |
| H | 4.667278 | -2.142215 | -0.016277 |
| H | 4.666685 | 2.138872 | -0.012271 |
| H | 2.199476 | 2.143177 | -0.001290 |
| H | 6.747861 | -0.949228 | -0.335796 |
| H | 6.744472 | 0.803583 | -0.614298 |
| H | 6.715977 | 0.170138 | 1.029044 |


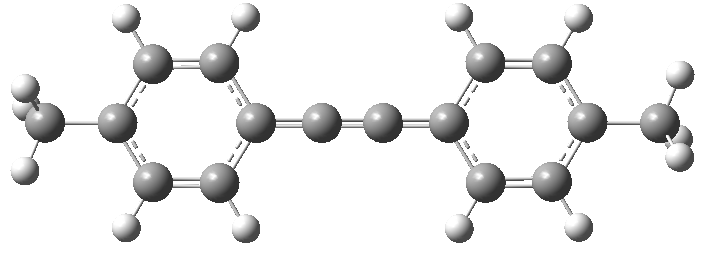


| C | -4.100804 | -1.215968 | 0.006166 |
| --- | --- | --- | --- |
| C | -4.829566 | -0.007220 | 0.006765 |
| C | -4.100761 | 1.207658 | 0.005930 |
| C | -2.736761 | 1.229350 | 0.002705 |
| C | -1.981852 | -0.001791 | 0.001210 |
| C | -2.733362 | -1.234629 | 0.003323 |
| C | -6.318389 | 0.005462 | -0.015263 |
| C | -0.628134 | 0.000364 | -0.000140 |
| C | 0.628133 | -0.000325 | -0.000218 |
| C | 1.981849 | 0.001821 | 0.000983 |
| C | 2.733375 | 1.234652 | 0.003388 |
| C | 4.100823 | 1.215973 | 0.006171 |
| C | 4.829566 | 0.007224 | 0.006420 |
| C | 4.100739 | -1.207655 | 0.005207 |
| C | 2.736747 | -1.229331 | 0.002032 |
| C | 6.318394 | -0.005531 | -0.015276 |
| H | -4.648662 | -2.155909 | 0.009074 |
| H | -4.651882 | 2.146138 | 0.007916 |
| H | -2.193582 | 2.169865 | 0.003666 |
| H | -2.188597 | -2.174123 | 0.005013 |
| H | -6.733076 | -0.985852 | 0.188456 |
| H | -6.725319 | 0.707740 | 0.722247 |
| H | -6.699529 | 0.328696 | -0.994081 |
| H | 2.188622 | 2.174152 | 0.005338 |
| H | 4.648687 | 2.155909 | 0.009300 |
| H | 4.651854 | -2.146141 | 0.006878 |
| H | 2.193555 | -2.169839 | 0.002709 |
| H | 6.733067 | 0.986104 | 0.186880 |
| H | 6.725105 | -0.706525 | 0.723593 |
| H | 6.699774 | -0.330529 | -0.993407 |


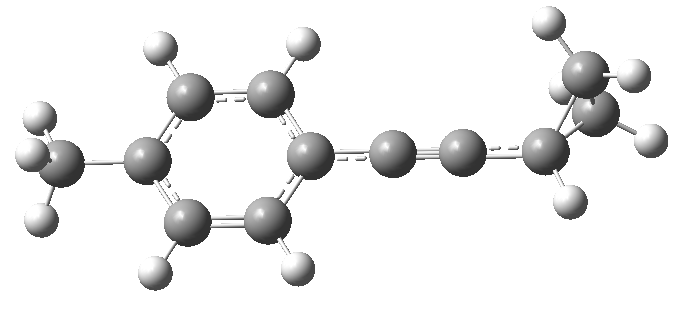


| C | 0.990701 | -0.284202 | -0.003706 |
| --- | --- | --- | --- |
| C | 2.191979 | -0.415627 | 0.000336 |
| C | -0.427494 | -0.145195 | -0.006284 |
| C | 3.614408 | -0.569785 | 0.006447 |
| C | -1.028810 | 1.117832 | -0.007267 |
| C | -2.409828 | 1.238854 | -0.009910 |
| C | -3.234328 | 0.115388 | -0.010082 |
| C | -2.629286 | -1.143135 | -0.013219 |
| C | -1.252621 | -1.277674 | -0.010286 |
| C | -4.724439 | 0.242613 | 0.010572 |
| C | 4.470932 | 0.423490 | -0.743405 |
| C | 4.465045 | 0.437954 | 0.743587 |
| H | 3.971847 | -1.595763 | 0.017523 |
| H | -0.401952 | 2.005079 | -0.007964 |
| H | -2.859955 | 2.228950 | -0.012888 |
| H | -3.255354 | -2.032941 | -0.018058 |
| H | -0.798638 | -2.264633 | -0.013309 |
| H | -5.043061 | 1.263438 | -0.218265 |
| H | -5.192739 | -0.433322 | -0.712664 |
| H | -5.128225 | -0.017581 | 0.996427 |
| H | 5.342855 | 0.029018 | -1.254128 |
| H | 3.950236 | 1.221489 | -1.262333 |
| H | 3.939966 | 1.245675 | 1.242703 |
| H | 5.332868 | 0.053819 | 1.268913 |


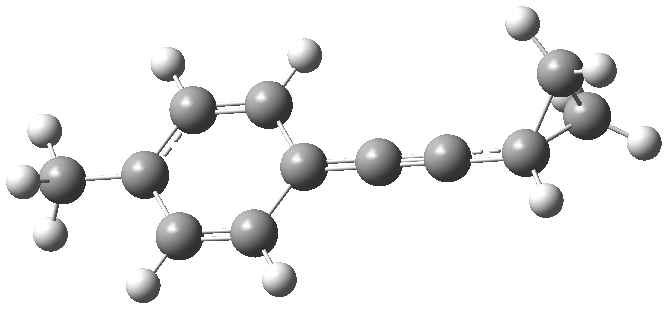


| C | 0.962931 | -0.306799 | -0.011547 |
| --- | --- | --- | --- |
| C | 2.194096 | -0.436321 | -0.001173 |
| C | -0.386980 | -0.168504 | -0.022664 |
| C | 3.604609 | -0.573413 | 0.021005 |
| C | -1.027339 | 1.167062 | -0.041339 |
| C | -2.374228 | 1.278928 | -0.030593 |
| C | -3.233552 | 0.132571 | -0.005312 |
| C | -2.615581 | -1.179236 | -0.000550 |
| C | -1.279170 | -1.337512 | -0.009296 |
| C | -4.710056 | 0.251520 | 0.027819 |
| C | 4.471623 | 0.418807 | -0.740651 |
| C | 4.449459 | 0.468602 | 0.739107 |
| H | 3.978533 | -1.592925 | 0.059140 |
| H | -0.385386 | 2.042575 | -0.062075 |
| H | -2.832926 | 2.265817 | -0.041596 |
| H | -3.266654 | -2.051320 | 0.011063 |
| H | -0.827220 | -2.325357 | -0.005352 |
| H | -5.035327 | 1.295149 | -0.008602 |
| H | -5.179597 | -0.280300 | -0.813215 |
| H | -5.132144 | -0.200727 | 0.938605 |
| H | 5.345049 | 0.006844 | -1.235225 |
| H | 3.947502 | 1.195718 | -1.287025 |
| H | 3.909015 | 1.279588 | 1.215660 |
| H | 5.307450 | 0.092972 | 1.286558 |


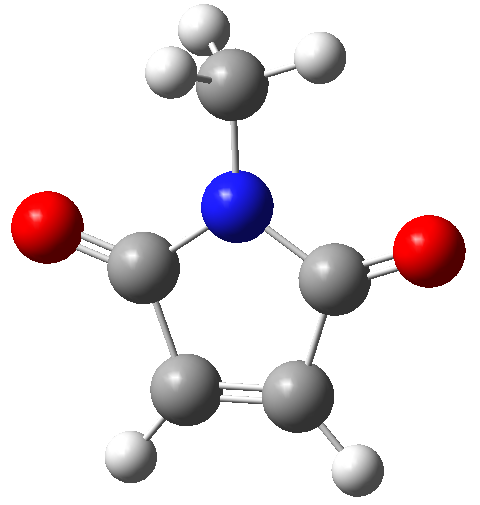


| C | 1.147001 | -0.172223 | 0.000135 |
| --- | --- | --- | --- |
| C | 0.709045 | -1.600067 | 0.000214 |
| C | -0.617798 | -1.636923 | -0.000123 |
| C | -1.129546 | -0.234482 | -0.000023 |
| N | -0.014165 | 0.589640 | -0.001912 |
| O | 2.272223 | 0.259729 | 0.000718 |
| O | -2.273976 | 0.145482 | 0.000963 |
| C | -0.073228 | 2.032195 | 0.000408 |
| H | 1.419979 | -2.415792 | 0.000403 |
| H | -1.281728 | -2.491149 | -0.000410 |
| H | 0.944141 | 2.415276 | -0.090643 |
| H | -0.668749 | 2.389027 | -0.843475 |
| H | -0.513305 | 2.402470 | 0.930387 |


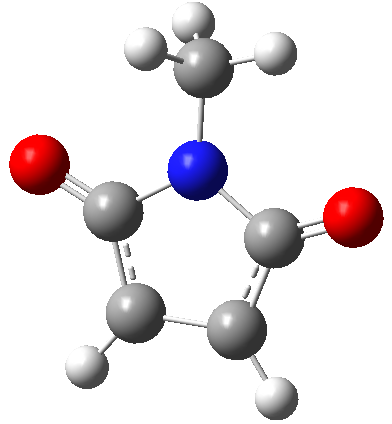


| C | 1.193911 | -0.196143 | 0.000012 |
| --- | --- | --- | --- |
| C | 0.783610 | -1.568685 | -0.000023 |
| C | -0.698956 | -1.607040 | -0.000040 |
| C | -1.172926 | -0.256134 | 0.000039 |
| N | -0.010500 | 0.541361 | 0.000038 |
| O | 2.306395 | 0.302751 | 0.000017 |
| O | -2.305316 | 0.198192 | 0.000032 |
| C | -0.070726 | 1.974681 | -0.000066 |
| H | 1.457077 | -2.414448 | 0.000053 |
| H | -1.328025 | -2.486166 | -0.000134 |
| H | 0.945820 | 2.368326 | -0.000175 |
| H | -0.609921 | 2.327525 | -0.885635 |
| H | -0.609559 | 2.327619 | 0.885700 |


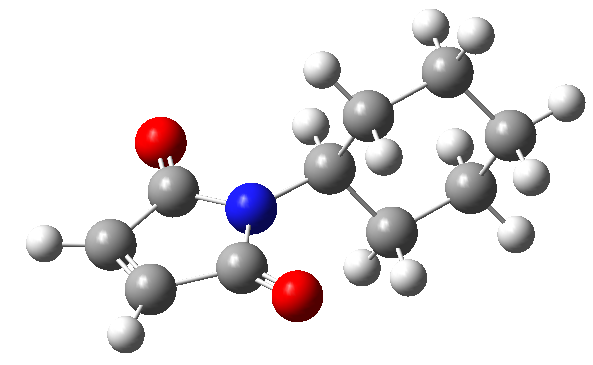


| C | -1.914844 | -1.077262 | 0.002150 |
| --- | --- | --- | --- |
| C | -3.222550 | -0.359542 | 0.000158 |
| C | -2.984924 | 0.945490 | -0.002082 |
| C | -1.506934 | 1.161570 | -0.001982 |
| N | -0.923459 | -0.100086 | 0.001015 |
| O | -1.727248 | -2.268414 | 0.004257 |
| O | -0.922514 | 2.216902 | -0.003960 |
| C | 0.509762 | -0.398028 | 0.001841 |
| C | 1.193883 | 0.116138 | 1.261887 |
| C | 2.667832 | -0.266885 | 1.255333 |
| C | 3.362805 | 0.238799 | -0.001626 |
| C | 2.667059 | -0.276694 | -1.254180 |
| C | 1.193105 | 0.106255 | -1.262620 |
| H | -4.166241 | -0.888862 | 0.000669 |
| H | -3.682392 | 1.772846 | -0.003857 |
| H | 0.558915 | -1.496175 | 0.006152 |
| H | 1.096860 | 1.209374 | 1.305341 |
| H | 0.686986 | -0.292295 | 2.144721 |
| H | 3.158718 | 0.125697 | 2.153177 |
| H | 2.757980 | -1.362912 | 1.301602 |
| H | 3.346313 | 1.339407 | -0.005957 |
| H | 4.417776 | -0.059459 | -0.000766 |
| H | 3.157312 | 0.108917 | -2.155377 |
| H | 2.757191 | -1.373045 | -1.291957 |
| H | 1.096151 | 1.199154 | -1.314592 |
| H | 0.685569 | -0.308998 | -2.141909 |


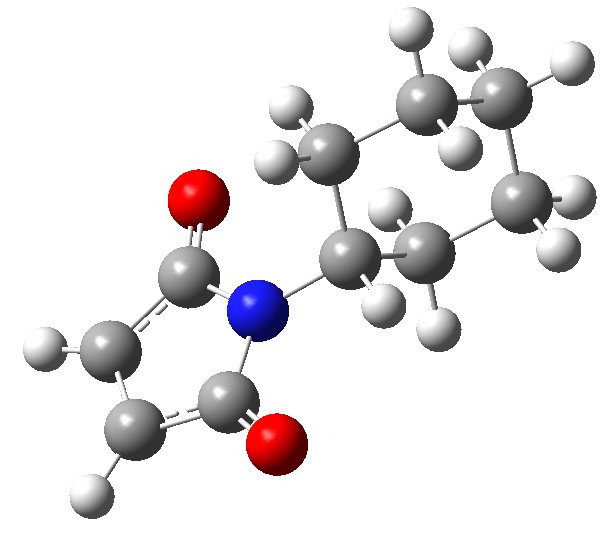


| C | -1.929258 | -1.109426 | 0.001216 |
| --- | --- | --- | --- |
| C | -3.184841 | -0.423744 | -0.000097 |
| C | -2.910966 | 1.029634 | -0.000896 |
| C | -1.491722 | 1.216999 | -0.000140 |
| N | -0.946056 | -0.088918 | 0.000825 |
| O | -1.679223 | -2.303540 | 0.001234 |
| O | -0.837233 | 2.247285 | -0.001563 |
| C | 0.473203 | -0.397770 | 0.000951 |
| C | 1.161658 | 0.117171 | 1.265603 |
| C | 2.629986 | -0.283917 | 1.254320 |
| C | 3.330806 | 0.217098 | -0.000917 |
| C | 2.629140 | -0.287339 | -1.254317 |
| C | 1.160778 | 0.113623 | -1.265653 |
| H | -4.152413 | -0.906238 | -0.000606 |
| H | -3.637379 | 1.830648 | -0.002090 |
| H | 0.519736 | -1.495333 | 0.002469 |
| H | 1.072133 | 1.209931 | 1.306983 |
| H | 0.650015 | -0.290611 | 2.145532 |
| H | 3.123104 | 0.103911 | 2.153157 |
| H | 2.708327 | -1.380617 | 1.300135 |
| H | 3.327276 | 1.317664 | -0.002429 |
| H | 4.381815 | -0.094338 | -0.000845 |
| H | 3.121613 | 0.098060 | -2.154548 |
| H | 2.707477 | -1.384157 | -1.297187 |
| H | 1.071099 | 1.206281 | -1.310091 |
| H | 0.648530 | -0.296712 | -2.144047 |


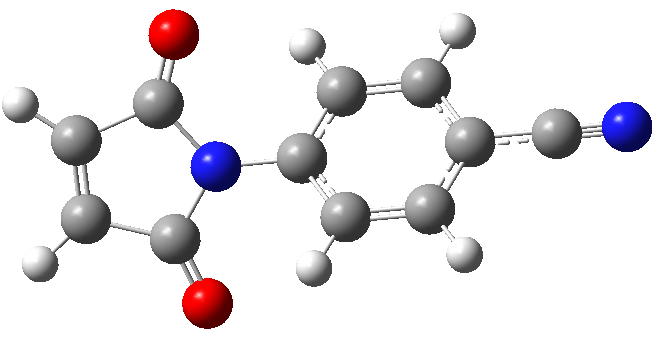


| C | -2.080373 | 1.036882 | 0.625644 |
| --- | --- | --- | --- |
| C | -0.698575 | 1.031725 | 0.631904 |
| C | -0.009760 | 0.000050 | 0.000421 |
| C | -0.697955 | -1.032144 | -0.630887 |
| C | -2.079750 | -1.038370 | -0.624247 |
| C | -2.774105 | -0.001010 | 0.000793 |
| N | 1.403506 | 0.000576 | 0.000255 |
| C | 2.213576 | -1.103781 | 0.315052 |
| C | 3.619473 | -0.636985 | 0.180604 |
| C | 3.618950 | 0.639889 | -0.180278 |
| C | 2.212677 | 1.105579 | -0.314568 |
| O | 1.819296 | -2.191516 | 0.637884 |
| O | 1.817506 | 2.193023 | -0.637299 |
| C | -4.199636 | -0.001541 | 0.000983 |
| N | -5.354198 | -0.001955 | 0.001137 |
| H | -2.627367 | 1.836774 | 1.113395 |
| H | -0.153676 | 1.824345 | 1.131426 |
| H | -0.152576 | -1.824335 | -1.130567 |
| H | -2.626261 | -1.838678 | -1.111857 |
| H | 4.455924 | -1.295825 | 0.371737 |
| H | 4.454853 | 1.299404 | -0.371481 |


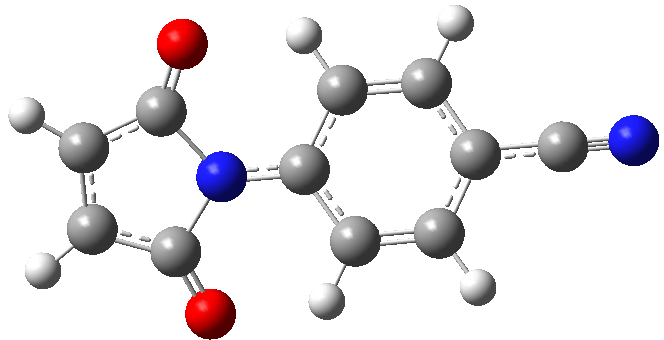


| C | -2.059785 | 1.139513 | 0.438730 |
| --- | --- | --- | --- |
| C | -0.692123 | 1.152697 | 0.430508 |
| C | 0.032593 | 0.000011 | 0.000587 |
| C | -0.691320 | -1.153218 | -0.429218 |
| C | -2.058991 | -1.140992 | -0.437393 |
| C | -2.755166 | -0.000973 | 0.000651 |
| N | 1.381514 | 0.000543 | 0.000474 |
| C | 2.246428 | -1.212726 | 0.027629 |
| C | 3.556644 | -0.702805 | 0.018700 |
| C | 3.556035 | 0.705707 | -0.018393 |
| C | 2.245402 | 1.214522 | -0.026929 |
| O | 1.792286 | -2.342158 | 0.093288 |
| O | 1.790320 | 2.343585 | -0.092426 |
| C | -4.177507 | -0.001435 | 0.000623 |
| N | -5.331861 | -0.001776 | 0.000599 |
| H | -2.613419 | 2.004205 | 0.787694 |
| H | -0.147812 | 2.021304 | 0.772633 |
| H | -0.146405 | -2.021453 | -0.771317 |
| H | -2.612028 | -2.006076 | -0.786332 |
| H | 4.423945 | -1.347526 | 0.057202 |
| H | 4.422775 | 1.351161 | -0.057260 |


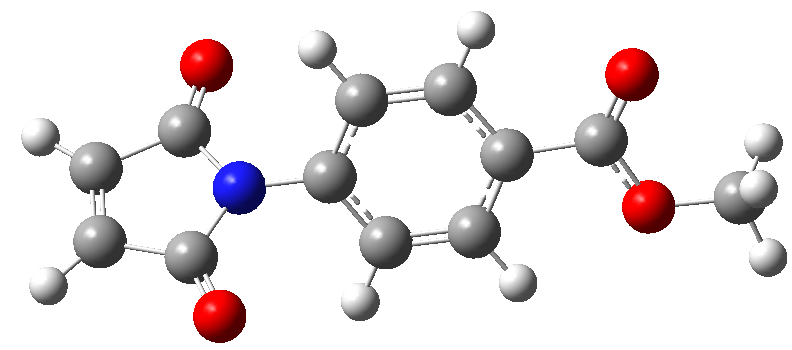


| C | 3.070522 | 0.999367 | 0.462139 |
| --- | --- | --- | --- |
| C | 4.461099 | 0.486166 | 0.322705 |
| C | 4.418994 | -0.747536 | -0.164491 |
| C | 2.997924 | -1.131324 | -0.388212 |
| N | 2.229287 | -0.029334 | 0.013694 |
| O | 2.710144 | 2.065990 | 0.882295 |
| O | 2.567018 | -2.161222 | -0.832967 |
| C | 0.814852 | 0.036740 | -0.029739 |
| C | 0.059942 | -0.945172 | 0.601776 |
| C | -1.321812 | -0.881695 | 0.554198 |
| C | -1.952797 | 0.168083 | -0.111555 |
| C | -1.185379 | 1.148322 | -0.737357 |
| C | 0.195540 | 1.083398 | -0.704520 |
| C | -3.429747 | 0.287826 | -0.181362 |
| O | -4.013453 | 1.174986 | -0.756324 |
| O | -4.051250 | -0.698313 | 0.467769 |
| C | -5.478907 | -0.657886 | 0.459286 |
| H | 5.318058 | 1.084741 | 0.601786 |
| H | 5.232345 | -1.422449 | -0.395529 |
| H | 0.555579 | -1.750643 | 1.133401 |
| H | -1.912665 | -1.646568 | 1.045336 |
| H | -1.684189 | 1.959689 | -1.257419 |
| H | 0.795628 | 1.835814 | -1.205204 |
| H | -5.806469 | -1.524605 | 1.032188 |
| H | -5.842621 | 0.259595 | 0.928644 |
| H | -5.861741 | -0.719505 | -0.562453 |


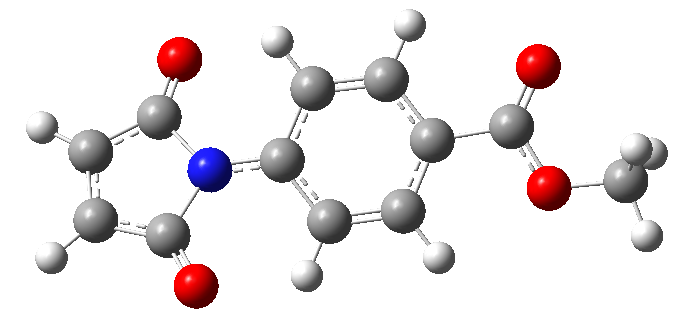


| C | 3.138224 | -1.103545 | -0.202802 |
| --- | --- | --- | --- |
| C | 4.414839 | -0.515800 | -0.143104 |
| C | 4.328048 | 0.869383 | 0.085871 |
| C | 2.988651 | 1.286548 | 0.183559 |
| N | 2.200524 | 0.036686 | -0.001667 |
| O | 2.751981 | -2.240098 | -0.418514 |
| O | 2.465623 | 2.363366 | 0.418601 |
| C | 0.854973 | -0.046713 | 0.006375 |
| C | 0.057717 | 1.100648 | -0.289594 |
| C | -1.308329 | 1.005394 | -0.284510 |
| C | -1.934890 | -0.209083 | 0.031995 |
| C | -1.162976 | -1.340776 | 0.331505 |
| C | 0.203661 | -1.280077 | 0.310696 |
| C | -3.417608 | -0.346780 | 0.071614 |
| O | -3.984101 | -1.378943 | 0.331106 |
| O | -4.032225 | 0.796354 | -0.207156 |
| C | -5.463053 | 0.769324 | -0.176953 |
| H | 5.318837 | -1.092197 | -0.285268 |
| H | 5.152953 | 1.558599 | 0.204536 |
| H | 0.546989 | 2.030513 | -0.542727 |
| H | -1.909954 | 1.872232 | -0.531994 |
| H | -1.665101 | -2.267178 | 0.588813 |
| H | 0.799175 | -2.147047 | 0.556153 |
| H | -5.785315 | 1.780942 | -0.418500 |
| H | -5.849125 | 0.063864 | -0.916624 |
| H | -5.817892 | 0.486898 | 0.817072 |


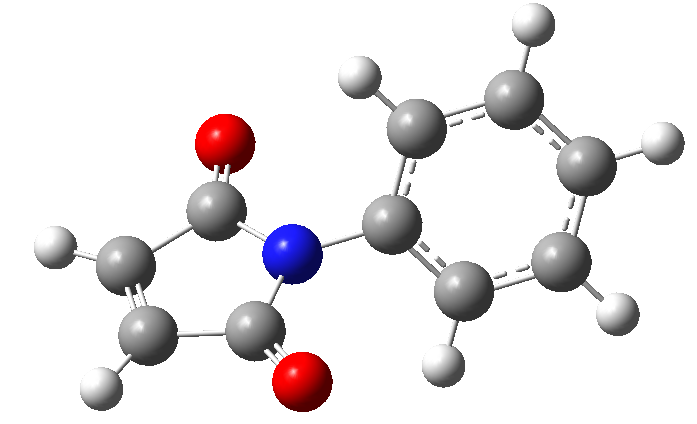


| C | 1.565620 | -1.108850 | -0.288322 |
| --- | --- | --- | --- |
| C | 2.975098 | -0.642611 | -0.165004 |
| C | 2.975099 | 0.642608 | 0.165004 |
| C | 1.565622 | 1.108850 | 0.288322 |
| N | 0.761476 | 0.000001 | -0.000001 |
| O | 1.170294 | -2.205687 | -0.583157 |
| O | 1.170299 | 2.205687 | 0.583157 |
| C | -0.659667 | 0.000001 | 0.000000 |
| C | -1.347058 | 0.874861 | -0.832335 |
| C | -2.734472 | 0.877457 | -0.822771 |
| C | -3.429528 | 0.000000 | 0.000001 |
| C | -2.734471 | -0.877457 | 0.822772 |
| C | -1.347056 | -0.874859 | 0.832335 |
| H | 3.810915 | -1.307040 | -0.339436 |
| H | 3.810917 | 1.307036 | 0.339436 |
| H | -0.794173 | 1.546782 | -1.481672 |
| H | -3.274206 | 1.564048 | -1.467739 |
| H | -4.515287 | -0.000001 | 0.000001 |
| H | -3.274204 | -1.564049 | 1.467740 |
| H | -0.794171 | -1.546780 | 1.481671 |


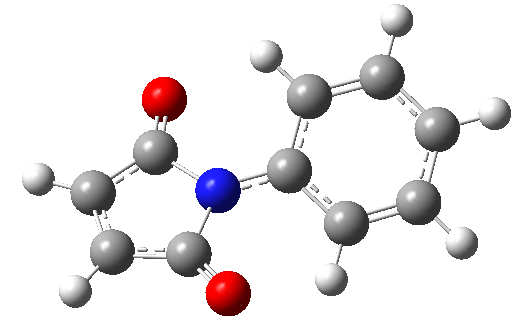


| C | -1.601157 | 1.200104 | -0.144138 |
| --- | --- | --- | --- |
| C | -2.913329 | 0.696117 | -0.084629 |
| C | -2.913324 | -0.696128 | 0.084630 |
| C | -1.601149 | -1.200106 | 0.144138 |
| N | -0.737301 | 0.000002 | 0.000002 |
| O | -1.146308 | 2.318786 | -0.327451 |
| O | -1.146291 | -2.318785 | 0.327448 |
| C | 0.612489 | 0.000003 | 0.000001 |
| C | 1.338108 | -1.178333 | -0.355719 |
| C | 2.707667 | -1.159077 | -0.362388 |
| C | 3.404463 | 0.000001 | -0.000001 |
| C | 2.707669 | 1.159080 | 0.362387 |
| C | 1.338110 | 1.178338 | 0.355721 |
| H | -3.778517 | 1.337016 | -0.186326 |
| H | -3.778508 | -1.337032 | 0.186333 |
| H | 0.790180 | -2.063751 | -0.645518 |
| H | 3.255614 | -2.047376 | -0.658118 |
| H | 4.490046 | 0.000000 | -0.000003 |
| H | 3.255619 | 2.047377 | 0.658116 |
| H | 0.790184 | 2.063756 | 0.645519 |


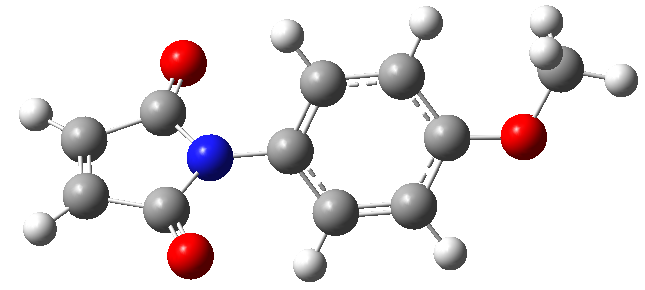


| C | -2.314249 | 1.152034 | -0.301711 |
| --- | --- | --- | --- |
| C | -3.742022 | 0.774035 | -0.103429 |
| C | -3.800059 | -0.494195 | 0.282656 |
| C | -2.413820 | -1.034043 | 0.371639 |
| N | -1.562124 | 0.013272 | 0.005441 |
| O | -1.871212 | 2.212517 | -0.657318 |
| O | -2.068876 | -2.140594 | 0.694232 |
| C | -0.144021 | -0.067641 | -0.048638 |
| C | 0.467692 | -0.986601 | -0.897460 |
| C | 1.844258 | -1.064094 | -0.951575 |
| C | 2.628158 | -0.211582 | -0.170047 |
| C | 2.016158 | 0.710426 | 0.676626 |
| C | 0.629735 | 0.769995 | 0.737870 |
| O | 3.964103 | -0.352923 | -0.303355 |
| C | 4.806798 | 0.490216 | 0.457870 |
| H | -4.545509 | 1.478572 | -0.272703 |
| H | -4.664141 | -1.101471 | 0.517841 |
| H | -0.143497 | -1.639336 | -1.513565 |
| H | 2.339401 | -1.776453 | -1.604358 |
| H | 2.603397 | 1.379512 | 1.294788 |
| H | 0.147731 | 1.479242 | 1.404096 |
| H | 5.830361 | 0.219634 | 0.196582 |
| H | 4.643087 | 1.546899 | 0.214059 |
| H | 4.660150 | 0.337214 | 1.533895 |


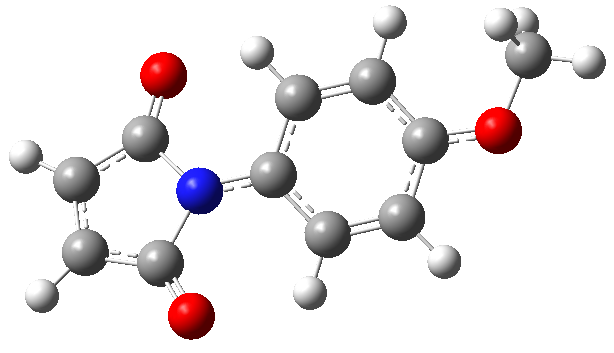


| C | 2.299748 | 1.275227 | 0.143673 |
| --- | --- | --- | --- |
| C | 3.651108 | 0.883822 | 0.049781 |
| C | 3.756998 | -0.496599 | -0.130275 |
| C | 2.481366 | -1.097752 | -0.165352 |
| N | 1.534131 | 0.021332 | 0.005369 |
| O | 1.765140 | 2.358947 | 0.349005 |
| O | 2.116690 | -2.253357 | -0.346534 |
| C | 0.186535 | -0.083039 | 0.022961 |
| C | -0.452535 | -1.315155 | 0.360427 |
| C | -1.808802 | -1.400767 | 0.384474 |
| C | -2.608100 | -0.279551 | 0.059087 |
| C | -1.996943 | 0.942054 | -0.288700 |
| C | -0.634559 | 1.039229 | -0.299198 |
| O | -3.910609 | -0.474491 | 0.108476 |
| C | -4.806157 | 0.603103 | -0.175304 |
| H | 4.460762 | 1.595729 | 0.137918 |
| H | 4.664892 | -1.071308 | -0.255176 |
| H | 0.155366 | -2.169537 | 0.620298 |
| H | -2.309434 | -2.321793 | 0.665242 |
| H | -2.596241 | 1.802339 | -0.562211 |
| H | -0.158570 | 1.970040 | -0.574946 |
| H | -5.807869 | 0.189589 | -0.070464 |
| H | -4.666898 | 1.417190 | 0.541735 |
| H | -4.662640 | 0.966210 | -1.196987 |


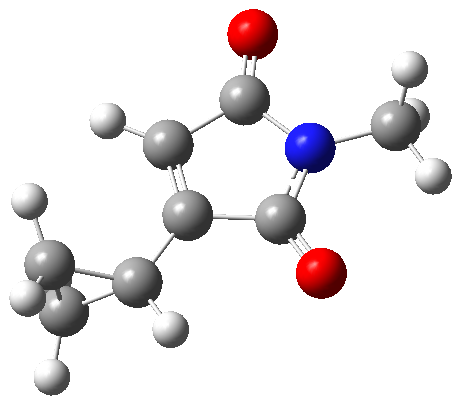


| C | 0.479416 | 0.998870 | -0.030253 |
| --- | --- | --- | --- |
| C | -0.671688 | 0.023124 | -0.054803 |
| C | -0.154002 | -1.210959 | -0.041492 |
| C | 1.318849 | -1.114004 | -0.003582 |
| N | 1.634426 | 0.245067 | -0.000770 |
| O | 0.412065 | 2.203197 | -0.034018 |
| O | 2.137662 | -2.001432 | 0.022069 |
| C | 2.980011 | 0.764906 | 0.041812 |
| H | -0.665613 | -2.165401 | -0.051657 |
| H | 2.924239 | 1.853085 | 0.095382 |
| H | 3.507022 | 0.388437 | 0.922291 |
| H | 3.537055 | 0.479795 | -0.854909 |
| C | -2.046312 | 0.498461 | -0.068920 |
| C | -3.072372 | -0.205073 | 0.797713 |
| C | -3.126210 | -0.367898 | -0.672435 |
| H | -2.156497 | 1.573106 | -0.173411 |
| H | -3.813756 | 0.423057 | 1.279757 |
| H | -2.705211 | -1.034649 | 1.394622 |
| H | -3.909255 | 0.142376 | -1.222705 |
| H | -2.802924 | -1.313962 | -1.096626 |


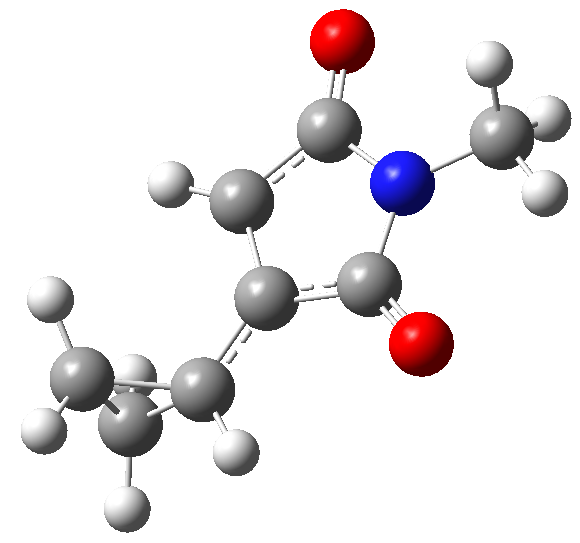


| C | 0.442400 | 1.045869 | 0.000053 |
| --- | --- | --- | --- |
| C | -0.672489 | 0.139786 | 0.000056 |
| C | -0.126806 | -1.231075 | 0.000058 |
| C | 1.306737 | -1.140513 | 0.000030 |
| N | 1.591703 | 0.228451 | 0.000025 |
| O | 0.464295 | 2.269845 | 0.000067 |
| O | 2.157546 | -2.020434 | -0.000016 |
| C | 2.937687 | 0.732350 | -0.000100 |
| H | -0.683263 | -2.159685 | 0.000171 |
| H | 2.900463 | 1.822152 | -0.000128 |
| H | 3.475224 | 0.380737 | 0.886330 |
| H | 3.475069 | 0.380686 | -0.886604 |
| C | -2.044288 | 0.524470 | 0.000085 |
| C | -3.084404 | -0.339483 | 0.732799 |
| C | -3.084314 | -0.339316 | -0.732985 |
| H | -2.229655 | 1.593859 | 0.000184 |
| H | -3.842426 | 0.229810 | 1.260372 |
| H | -2.708561 | -1.212470 | 1.256393 |
| H | -3.842254 | 0.230109 | -1.260535 |
| H | -2.708383 | -1.212169 | -1.256740 |


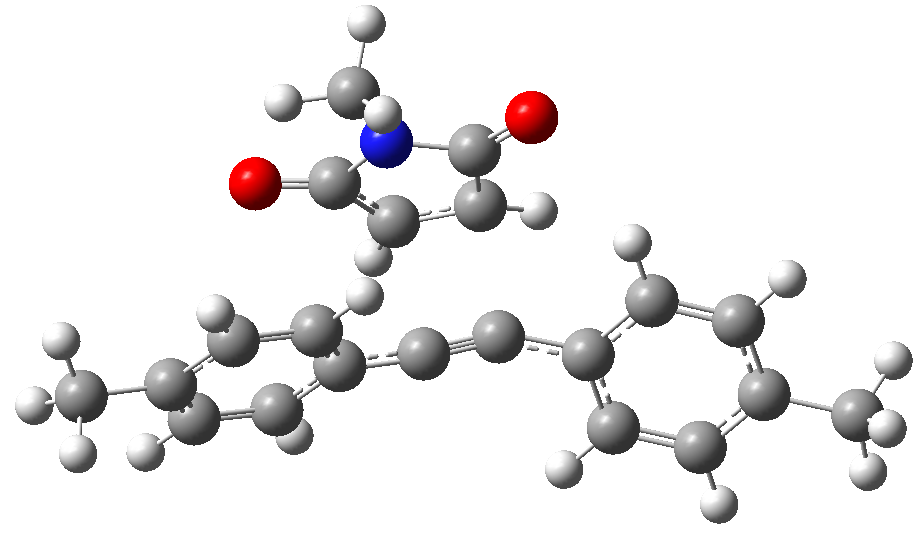


| C | -0.956034 | -0.448856 | 0.148794 |
| --- | --- | --- | --- |
| C | 0.220459 | -0.829794 | 0.060017 |
| C | 0.455945 | 1.288404 | 1.890012 |
| C | -0.722875 | 1.618115 | 1.140724 |
| C | -2.371698 | -0.598783 | 0.031726 |
| C | 1.568747 | -1.145793 | -0.108218 |
| C | -3.131279 | 0.275691 | -0.758621 |
| C | -4.492691 | 0.085954 | -0.884331 |
| C | -5.143480 | -0.965430 | -0.229145 |
| C | -4.383143 | -1.821298 | 0.565476 |
| C | -3.015423 | -1.645392 | 0.701128 |
| C | 2.284018 | -1.858157 | 0.874921 |
| C | 3.621937 | -2.133404 | 0.691775 |
| C | 4.296840 | -1.720286 | -0.463252 |
| C | 3.583875 | -1.011801 | -1.436591 |
| C | 2.245668 | -0.725561 | -1.271973 |
| C | -6.616128 | -1.155710 | -0.393696 |
| C | 5.738274 | -2.048330 | -0.665800 |
| C | 1.607601 | 1.744286 | 1.154748 |
| N | 1.097164 | 2.382563 | 0.011537 |
| C | -0.298248 | 2.380404 | -0.019412 |
| O | -0.962088 | 2.913900 | -0.899813 |
| O | 2.799369 | 1.631376 | 1.410939 |
| C | 1.909696 | 3.018826 | -0.992241 |
| H | 0.515669 | 0.764680 | 2.835206 |
| H | -1.736226 | 1.644370 | 1.518201 |
| H | -2.636990 | 1.105333 | -1.257676 |
| H | -5.074897 | 0.765743 | -1.502494 |
| H | -4.873148 | -2.640792 | 1.085422 |
| H | -2.432020 | -2.319820 | 1.321209 |
| H | 1.771042 | -2.169872 | 1.780348 |
| H | 4.170037 | -2.673629 | 1.459552 |
| H | 4.099994 | -0.678818 | -2.333765 |
| H | 1.697174 | -0.167911 | -2.026015 |
| H | -6.990235 | -1.970293 | 0.232022 |
| H | -7.164686 | -0.243518 | -0.133560 |
| H | -6.865445 | -1.387751 | -1.435811 |
| H | 6.253766 | -2.196370 | 0.287315 |
| H | 5.845813 | -2.975782 | -1.242703 |
| H | 6.253218 | -1.260750 | -1.224089 |
| H | 2.955040 | 2.775604 | -0.792929 |
| H | 1.635984 | 2.664284 | -1.990089 |
| H | 1.785152 | 4.106626 | -0.966152 |


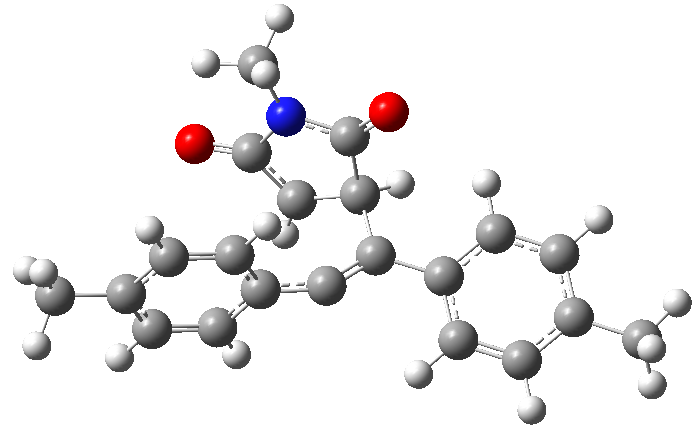


| C | -0.952404 | -0.060541 | 0.376357 |
| --- | --- | --- | --- |
| C | -0.022673 | -0.941893 | 0.076188 |
| C | 0.749372 | 1.217370 | 1.702620 |
| C | -0.480744 | 1.311710 | 0.885231 |
| C | -2.398236 | -0.316475 | 0.216424 |
| C | 1.318660 | -1.277424 | -0.071784 |
| C | -3.322179 | 0.720315 | 0.094436 |
| C | -4.672821 | 0.451699 | -0.091053 |
| C | -5.147000 | -0.854304 | -0.157768 |
| C | -4.219670 | -1.892329 | -0.029254 |
| C | -2.875069 | -1.631645 | 0.153846 |
| C | 2.036463 | -1.940450 | 0.956785 |
| C | 3.372416 | -2.232599 | 0.799496 |
| C | 4.061252 | -1.902428 | -0.376502 |
| C | 3.351616 | -1.267719 | -1.401064 |
| C | 2.011753 | -0.965197 | -1.268100 |
| C | -6.597109 | -1.151814 | -0.371743 |
| C | 5.515217 | -2.215265 | -0.514255 |
| C | 1.845090 | 1.874688 | 1.030799 |
| N | 1.305798 | 2.430689 | -0.143974 |
| C | -0.041619 | 2.182625 | -0.286905 |
| O | -0.720784 | 2.572254 | -1.207131 |
| O | 3.012099 | 1.961331 | 1.354907 |
| C | 2.081753 | 3.151514 | -1.124646 |
| H | 0.849285 | 0.705616 | 2.651375 |
| H | -1.298157 | 1.810481 | 1.422495 |
| H | -2.990525 | 1.754389 | 0.115398 |
| H | -5.373425 | 1.277766 | -0.190651 |
| H | -4.567737 | -2.922618 | -0.068730 |
| H | -2.170791 | -2.453912 | 0.260268 |
| H | 1.517341 | -2.198731 | 1.876010 |
| H | 3.910957 | -2.728715 | 1.604307 |
| H | 3.870376 | -1.008715 | -2.321334 |
| H | 1.473409 | -0.471859 | -2.073480 |
| H | -6.969051 | -1.875157 | 0.362215 |
| H | -7.207372 | -0.247484 | -0.297973 |
| H | -6.767785 | -1.588999 | -1.362879 |
| H | 6.118649 | -1.586008 | 0.151618 |
| H | 5.726548 | -3.255448 | -0.242614 |
| H | 5.866529 | -2.050075 | -1.536431 |
| H | 3.132759 | 3.090911 | -0.838907 |
| H | 1.945540 | 2.704735 | -2.113329 |
| H | 1.777928 | 4.201292 | -1.166398 |


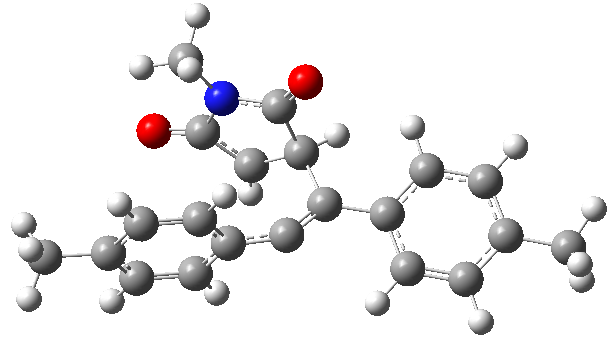


| C | -1.003351 | -0.159095 | 0.195159 |
| --- | --- | --- | --- |
| C | -0.112020 | -1.048938 | -0.168515 |
| C | 0.665749 | 1.152740 | 1.628104 |
| C | -0.452443 | 1.218374 | 0.673978 |
| C | -2.462227 | -0.367933 | 0.129727 |
| C | 1.255292 | -1.309789 | -0.229400 |
| C | -3.365159 | 0.686520 | 0.007715 |
| C | -4.731269 | 0.446072 | -0.085363 |
| C | -5.241167 | -0.847227 | -0.057706 |
| C | -4.333467 | -1.903678 | 0.063852 |
| C | -2.974911 | -1.671094 | 0.155651 |
| C | 1.957425 | -1.735820 | 0.923986 |
| C | 3.326057 | -1.908320 | 0.884820 |
| C | 4.053535 | -1.670427 | -0.285586 |
| C | 3.357163 | -1.268407 | -1.433952 |
| C | 1.990026 | -1.099438 | -1.420532 |
| C | -6.710190 | -1.113629 | -0.149409 |
| C | 5.538496 | -1.824352 | -0.303831 |
| C | 1.847146 | 1.747838 | 1.051151 |
| N | 1.471405 | 2.208211 | -0.227440 |
| C | 0.146251 | 1.973452 | -0.507643 |
| O | -0.414356 | 2.291031 | -1.530391 |
| O | 2.963321 | 1.870414 | 1.514811 |
| C | 2.380757 | 2.823154 | -1.163840 |
| H | 0.626292 | 0.766146 | 2.638442 |
| H | -1.293748 | 1.789239 | 1.087662 |
| H | -3.008621 | 1.711635 | -0.044285 |
| H | -5.415309 | 1.285480 | -0.187779 |
| H | -4.708399 | -2.924894 | 0.091657 |
| H | -2.284858 | -2.505877 | 0.256256 |
| H | 1.401397 | -1.921448 | 1.838950 |
| H | 3.854313 | -2.225972 | 1.780971 |
| H | 3.912741 | -1.086213 | -2.351034 |
| H | 1.460423 | -0.781533 | -2.315272 |
| H | -7.077436 | -1.624981 | 0.747921 |
| H | -7.278691 | -0.186772 | -0.265088 |
| H | -6.943646 | -1.762409 | -1.001300 |
| H | 6.026492 | -0.934954 | 0.114578 |
| H | 5.859765 | -2.678412 | 0.300688 |
| H | 5.916786 | -1.956707 | -1.321395 |
| H | 3.392421 | 2.736392 | -0.764195 |
| H | 2.324504 | 2.312979 | -2.129310 |
| H | 2.139168 | 3.880224 | -1.308938 |


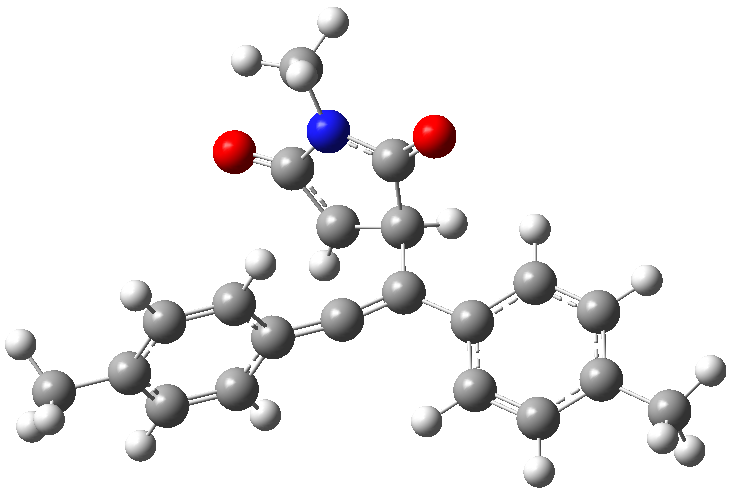


| C | -0.839245 | 0.257663 | 0.423745 |
| --- | --- | --- | --- |
| C | 0.193514 | -0.535169 | 0.258756 |
| C | 0.708293 | 1.726024 | 1.694383 |
| C | -0.545484 | 1.682788 | 0.902364 |
| C | -2.233327 | -0.161306 | 0.161693 |
| C | 1.406873 | -1.159163 | 0.117220 |
| C | -3.269011 | 0.759768 | 0.010091 |
| C | -4.566224 | 0.333041 | -0.248160 |
| C | -4.873874 | -1.018776 | -0.360147 |
| C | -3.833599 | -1.939945 | -0.207708 |
| C | -2.541917 | -1.522263 | 0.049156 |
| C | 1.955573 | -1.960778 | 1.156771 |
| C | 3.177104 | -2.578374 | 0.994083 |
| C | 3.916666 | -2.444754 | -0.186993 |
| C | 3.380616 | -1.652974 | -1.214854 |
| C | 2.163885 | -1.026188 | -1.083356 |
| C | -6.272378 | -1.483015 | -0.618208 |
| C | 5.234016 | -3.125771 | -0.366423 |
| C | 1.716645 | 2.480430 | 0.990416 |
| N | 1.103081 | 2.949526 | -0.185507 |
| C | -0.218780 | 2.574625 | -0.290453 |
| O | -0.953644 | 2.888630 | -1.196207 |
| O | 2.872733 | 2.698410 | 1.291894 |
| C | 1.770219 | 3.758707 | -1.178030 |
| H | 0.883573 | 1.222732 | 2.637086 |
| H | -1.406247 | 2.096309 | 1.444733 |
| H | -3.065566 | 1.825139 | 0.064823 |
| H | -5.355755 | 1.071013 | -0.371431 |
| H | -4.049672 | -3.003412 | -0.287861 |
| H | -1.749779 | -2.257731 | 0.172920 |
| H | 1.397979 | -2.073462 | 2.082599 |
| H | 3.580466 | -3.183897 | 1.802737 |
| H | 3.945577 | -1.533223 | -2.137292 |
| H | 1.763486 | -0.415770 | -1.888682 |
| H | -6.725685 | -1.896895 | 0.290827 |
| H | -6.909962 | -0.662848 | -0.960516 |
| H | -6.296145 | -2.274465 | -1.374670 |
| H | 6.001244 | -2.422923 | -0.710503 |
| H | 5.581792 | -3.581278 | 0.564910 |
| H | 5.171264 | -3.917870 | -1.123082 |
| H | 2.827236 | 3.817625 | -0.916032 |
| H | 1.663806 | 3.304835 | -2.166586 |
| H | 1.346308 | 4.766570 | -1.204629 |


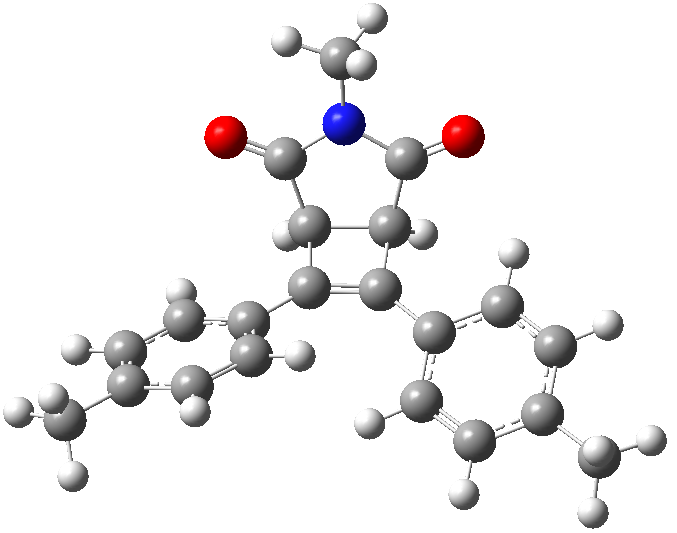


| C | 0.704785 | 0.372008 | -0.635292 |
| --- | --- | --- | --- |
| C | -0.648397 | 0.273314 | -0.686461 |
| C | -0.822444 | 1.693521 | -1.209284 |
| C | 0.706309 | 1.824399 | -1.095523 |
| C | 1.850662 | -0.475507 | -0.359379 |
| C | -1.690665 | -0.678723 | -0.347871 |
| C | 3.096607 | 0.110640 | -0.110290 |
| C | 4.214467 | -0.671699 | 0.134204 |
| C | 4.136031 | -2.062973 | 0.126590 |
| C | 2.898355 | -2.647268 | -0.152662 |
| C | 1.776292 | -1.874196 | -0.391387 |
| C | -2.949483 | -0.563328 | -0.944066 |
| C | -3.973050 | -1.442020 | -0.621879 |
| C | -3.782795 | -2.454044 | 0.315677 |
| C | -2.530796 | -2.553230 | 0.929650 |
| C | -1.503240 | -1.687287 | 0.606773 |
| C | 5.333666 | -2.906480 | 0.426481 |
| C | -4.878271 | -3.407487 | 0.671478 |
| C | -1.392976 | 2.646196 | -0.195287 |
| N | -0.321616 | 3.241146 | 0.464540 |
| C | 0.921282 | 2.840723 | -0.002846 |
| O | 1.967654 | 3.256976 | 0.435177 |
| O | -2.554000 | 2.861729 | 0.053008 |
| C | -0.467981 | 4.161302 | 1.571075 |
| H | -1.279661 | 1.811550 | -2.194845 |
| H | 1.287777 | 2.063922 | -1.990400 |
| H | 3.183974 | 1.194523 | -0.100497 |
| H | 5.171241 | -0.193235 | 0.331617 |
| H | 2.820311 | -3.731593 | -0.190345 |
| H | 0.833271 | -2.358639 | -0.628672 |
| H | -3.126583 | 0.227567 | -1.668647 |
| H | -4.942862 | -1.337556 | -1.102752 |
| H | -2.369273 | -3.323398 | 1.681211 |
| H | -0.548825 | -1.769031 | 1.119044 |
| H | 6.263615 | -2.379480 | 0.193109 |
| H | 5.368390 | -3.171893 | 1.490410 |
| H | 5.316868 | -3.844142 | -0.137918 |
| H | -5.806021 | -3.167680 | 0.144552 |
| H | -4.603769 | -4.438122 | 0.417993 |
| H | -5.084934 | -3.390604 | 1.747643 |
| H | -1.532661 | 4.319873 | 1.744826 |
| H | -0.008426 | 3.747510 | 2.472407 |
| H | 0.009499 | 5.115594 | 1.335794 |


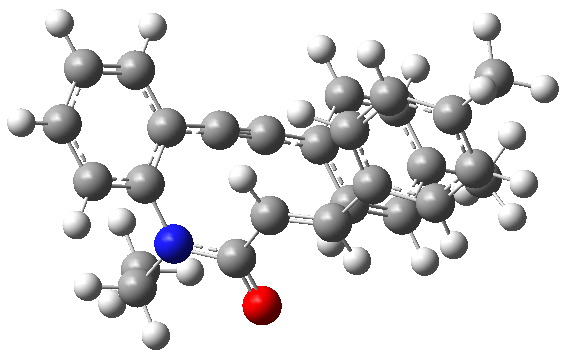


| C | -3.893281 | -1.763571 | 2.422809 |
| --- | --- | --- | --- |
| C | -4.490806 | -2.157034 | 1.230510 |
| C | -4.059925 | -1.614953 | 0.028718 |
| C | -3.036397 | -0.676015 | 0.001298 |
| C | -2.432868 | -0.265308 | 1.206466 |
| C | -2.871768 | -0.830287 | 2.410617 |
| N | -2.658607 | -0.089919 | -1.239552 |
| C | -1.394470 | 0.702026 | 1.187329 |
| C | -0.505121 | 1.515967 | 1.103948 |
| C | 0.532166 | 2.468680 | 0.923085 |
| C | 1.158184 | 3.087674 | 2.011353 |
| C | 2.164717 | 4.013773 | 1.801829 |
| C | 2.577032 | 4.355416 | 0.512515 |
| C | 1.953469 | 3.729844 | -0.567884 |
| C | 0.946824 | 2.799695 | -0.373687 |
| C | 3.641051 | 5.383815 | 0.298307 |
| C | -1.395850 | -0.174247 | -1.779533 |
| C | -3.610404 | 0.856893 | -1.831511 |
| C | -3.563022 | 2.219358 | -1.172685 |
| C | -0.508931 | -1.211094 | -1.216425 |
| O | -1.055469 | 0.549978 | -2.708225 |
| C | 0.771611 | -1.284818 | -1.597401 |
| C | 1.765524 | -2.246852 | -1.142117 |
| C | 3.023975 | -2.260525 | -1.750562 |
| C | 3.998736 | -3.165972 | -1.358842 |
| C | 3.753281 | -4.087401 | -0.344202 |
| C | 2.498910 | -4.065655 | 0.274838 |
| C | 1.523762 | -3.167507 | -0.113745 |
| C | 4.787379 | -5.085257 | 0.067266 |
| H | -4.225875 | -2.186482 | 3.365683 |
| H | -5.288945 | -2.892968 | 1.235530 |
| H | -4.513585 | -1.919868 | -0.910521 |
| H | -2.400893 | -0.515481 | 3.337033 |
| H | 0.847835 | 2.832410 | 3.020501 |
| H | 2.645511 | 4.485817 | 2.655691 |
| H | 2.267171 | 3.976915 | -1.579513 |
| H | 0.471470 | 2.313489 | -1.223199 |
| H | 4.149590 | 5.241486 | -0.659752 |
| H | 3.213229 | 6.393928 | 0.292684 |
| H | 4.390562 | 5.356387 | 1.095147 |
| H | -3.375536 | 0.932352 | -2.895294 |
| H | -4.609741 | 0.418041 | -1.739900 |
| H | -4.292833 | 2.890341 | -1.637169 |
| H | -3.800420 | 2.151741 | -0.105254 |
| H | -2.569784 | 2.668999 | -1.275472 |
| H | -0.924321 | -1.912357 | -0.498157 |
| H | 1.116902 | -0.555508 | -2.331707 |
| H | 3.233318 | -1.546885 | -2.544027 |
| H | 4.970597 | -3.157978 | -1.846993 |
| H | 2.294101 | -4.768820 | 1.079347 |
| H | 0.563946 | -3.171122 | 0.395844 |
| H | 5.786266 | -4.788248 | -0.264792 |
| H | 4.808537 | -5.214338 | 1.154185 |
| H | 4.575126 | -6.069896 | -0.367406 |


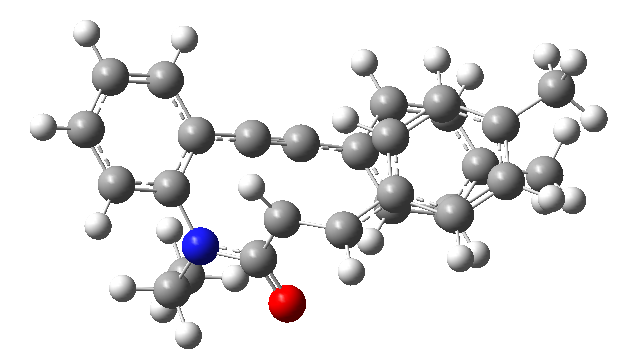


| C | -3.964031 | -0.631006 | 3.574926 |
| --- | --- | --- | --- |
| C | -4.671568 | -1.537140 | 2.792530 |
| C | -4.347895 | -1.693752 | 1.453168 |
| C | -3.322929 | -0.951364 | 0.879582 |
| C | -2.608827 | -0.022367 | 1.663390 |
| C | -2.941756 | 0.116047 | 3.017211 |
| N | -3.048588 | -1.096161 | -0.507379 |
| C | -1.564005 | 0.748936 | 1.089627 |
| C | -0.658906 | 1.354863 | 0.565446 |
| C | 0.451662 | 1.952421 | -0.087841 |
| C | 1.186887 | 2.983708 | 0.504764 |
| C | 2.319606 | 3.480610 | -0.119337 |
| C | 2.752825 | 2.970394 | -1.343480 |
| C | 2.009198 | 1.945517 | -1.933101 |
| C | 0.875341 | 1.441814 | -1.323106 |
| C | 3.965437 | 3.520545 | -2.022940 |
| C | -1.833675 | -1.520617 | -1.002054 |
| C | -4.020266 | -0.519595 | -1.441764 |
| C | -3.875942 | 0.980288 | -1.592143 |
| C | -0.957232 | -2.254149 | -0.088973 |
| O | -1.530513 | -1.353744 | -2.181856 |
| C | 0.346629 | -2.686669 | -0.567211 |
| C | 1.570154 | -2.083648 | -0.219880 |
| C | 2.760332 | -2.411420 | -0.921410 |
| C | 3.951073 | -1.785110 | -0.631084 |
| C | 4.042383 | -0.821732 | 0.385585 |
| C | 2.881036 | -0.515129 | 1.102768 |
| C | 1.674432 | -1.118641 | 0.813038 |
| C | 5.345131 | -0.160873 | 0.699530 |
| H | -4.211835 | -0.506516 | 4.624531 |
| H | -5.471754 | -2.127468 | 3.228040 |
| H | -4.887439 | -2.403578 | 0.831857 |
| H | -2.387383 | 0.828986 | 3.620087 |
| H | 0.869508 | 3.381386 | 1.464615 |
| H | 2.887006 | 4.278014 | 0.355165 |
| H | 2.337765 | 1.529136 | -2.882852 |
| H | 0.314925 | 0.631397 | -1.783938 |
| H | 4.523915 | 2.734287 | -2.540662 |
| H | 3.685755 | 4.265259 | -2.778444 |
| H | 4.636356 | 4.012224 | -1.312518 |
| H | -3.883897 | -1.019240 | -2.403868 |
| H | -5.019534 | -0.774027 | -1.071814 |
| H | -4.629484 | 1.364459 | -2.287507 |
| H | -4.013688 | 1.489209 | -0.631846 |
| H | -2.887001 | 1.242526 | -1.982326 |
| H | -1.351978 | -2.601397 | 0.866789 |
| H | 0.367324 | -3.480100 | -1.317409 |
| H | 2.709828 | -3.151889 | -1.716598 |
| H | 4.844119 | -2.036935 | -1.199892 |
| H | 2.933349 | 0.223370 | 1.900592 |
| H | 0.782224 | -0.848958 | 1.374058 |
| H | 5.920414 | 0.039749 | -0.210754 |
| H | 5.196540 | 0.786155 | 1.227775 |
| H | 5.972026 | -0.796765 | 1.338362 |


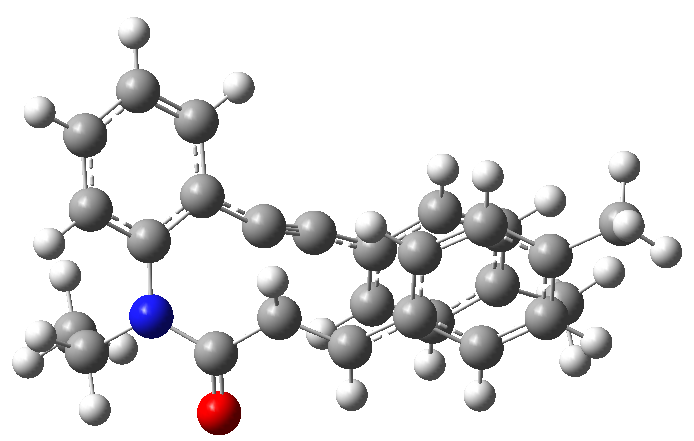


| C | -3.705055 | 0.210135 | 3.389894 |
| --- | --- | --- | --- |
| C | -4.591958 | -0.744085 | 2.910394 |
| C | -4.415451 | -1.294712 | 1.649247 |
| C | -3.355078 | -0.899334 | 0.838354 |
| C | -2.454796 | 0.072342 | 1.321198 |
| C | -2.640703 | 0.609200 | 2.597382 |
| N | -3.231227 | -1.419439 | -0.470007 |
| C | -1.329843 | 0.482891 | 0.533992 |
| C | -0.450578 | 1.201185 | 0.060620 |
| C | 0.629417 | 1.845872 | -0.566646 |
| C | 1.806546 | 2.131190 | 0.144823 |
| C | 2.879049 | 2.732502 | -0.491350 |
| C | 2.824035 | 3.067067 | -1.843707 |
| C | 1.649771 | 2.777098 | -2.550435 |
| C | 0.569753 | 2.181943 | -1.932965 |
| C | 3.975777 | 3.724728 | -2.532500 |
| C | -1.997927 | -1.543545 | -1.100920 |
| C | -4.420820 | -1.386145 | -1.328332 |
| C | -4.775231 | 0.026737 | -1.744303 |
| C | -0.828946 | -1.579897 | -0.238583 |
| O | -1.913765 | -1.612876 | -2.325313 |
| C | 0.491396 | -1.456067 | -0.835067 |
| C | 1.688108 | -1.416631 | -0.123488 |
| C | 2.919619 | -1.202430 | -0.812319 |
| C | 4.115396 | -1.130153 | -0.137413 |
| C | 4.175035 | -1.260610 | 1.259034 |
| C | 2.969046 | -1.465557 | 1.951747 |
| C | 1.762235 | -1.538931 | 1.297806 |
| C | 5.471536 | -1.194395 | 1.996542 |
| H | -3.837576 | 0.640050 | 4.377674 |
| H | -5.425390 | -1.072730 | 3.523987 |
| H | -5.108360 | -2.049159 | 1.290110 |
| H | -1.933348 | 1.352053 | 2.954526 |
| H | 1.866411 | 1.870408 | 1.197842 |
| H | 3.784879 | 2.945187 | 0.071949 |
| H | 1.592628 | 3.026916 | -3.607876 |
| H | -0.333146 | 1.952802 | -2.493563 |
| H | 4.298213 | 3.147878 | -3.406878 |
| H | 3.700149 | 4.721514 | -2.896869 |
| H | 4.832961 | 3.836792 | -1.863132 |
| H | -4.201426 | -2.003359 | -2.200646 |
| H | -5.251013 | -1.858687 | -0.796354 |
| H | -5.635998 | 0.025935 | -2.420536 |
| H | -5.031574 | 0.645325 | -0.876654 |
| H | -3.933652 | 0.498990 | -2.263466 |
| H | -0.933446 | -2.006832 | 0.755205 |
| H | 0.528937 | -1.343524 | -1.916783 |
| H | 2.893282 | -1.094757 | -1.894937 |
| H | 5.037673 | -0.965024 | -0.690690 |
| H | 2.998792 | -1.565070 | 3.035442 |
| H | 0.852545 | -1.692402 | 1.871893 |
| H | 6.303754 | -0.964422 | 1.325353 |
| H | 5.446516 | -0.428729 | 2.781262 |
| H | 5.695901 | -2.145926 | 2.494902 |


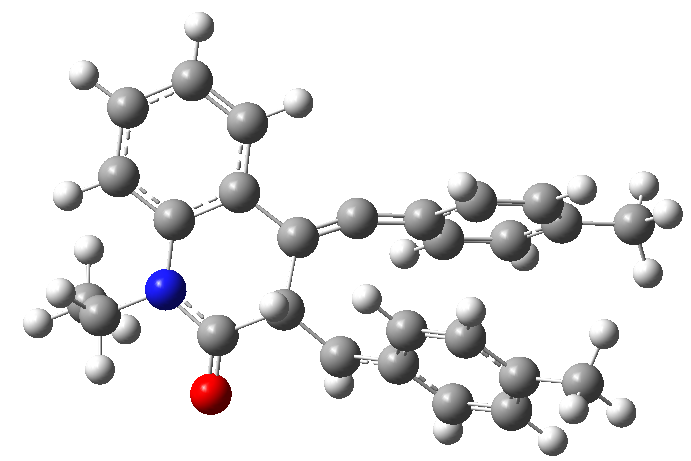


| C | -3.908431 | 1.408416 | 2.709983 |
| --- | --- | --- | --- |
| C | -4.938624 | 0.536174 | 2.385603 |
| C | -4.750761 | -0.457291 | 1.436428 |
| C | -3.520201 | -0.603271 | 0.791312 |
| C | -2.479046 | 0.282616 | 1.113029 |
| C | -2.688653 | 1.272426 | 2.070270 |
| N | -3.331898 | -1.583668 | -0.210035 |
| C | -1.190457 | 0.148750 | 0.417565 |
| C | -0.355404 | 1.137355 | 0.228567 |
| C | 0.751514 | 1.855411 | -0.168376 |
| C | 1.844087 | 2.066473 | 0.713985 |
| C | 2.950029 | 2.781763 | 0.304960 |
| C | 3.034563 | 3.327691 | -0.980834 |
| C | 1.957182 | 3.122401 | -1.856321 |
| C | 0.841638 | 2.413225 | -1.474028 |
| C | 4.227838 | 4.112707 | -1.418397 |
| C | -2.100300 | -1.878384 | -0.749007 |
| C | -4.491257 | -2.270273 | -0.791698 |
| C | -5.206543 | -1.417664 | -1.817037 |
| C | -0.891020 | -1.273995 | -0.055052 |
| O | -1.976681 | -2.629194 | -1.699038 |
| C | 0.327203 | -1.362523 | -0.900620 |
| C | 1.641846 | -1.421458 | -0.407105 |
| C | 2.734063 | -1.336152 | -1.308025 |
| C | 4.038127 | -1.338997 | -0.859581 |
| C | 4.338033 | -1.434784 | 0.504108 |
| C | 3.266885 | -1.529942 | 1.403763 |
| C | 1.957598 | -1.520425 | 0.973379 |
| C | 5.748826 | -1.438608 | 0.996848 |
| H | -4.053883 | 2.184200 | 3.454967 |
| H | -5.904943 | 0.623987 | 2.873267 |
| H | -5.581006 | -1.110883 | 1.198450 |
| H | -1.862398 | 1.938596 | 2.307876 |
| H | 1.799142 | 1.644756 | 1.714533 |
| H | 3.778581 | 2.925347 | 0.995163 |
| H | 2.011850 | 3.533377 | -2.862644 |
| H | 0.019361 | 2.257986 | -2.167212 |
| H | 4.641631 | 3.721409 | -2.355067 |
| H | 3.967335 | 5.162066 | -1.604148 |
| H | 5.018623 | 4.093761 | -0.663414 |
| H | -4.112577 | -3.182545 | -1.253493 |
| H | -5.159204 | -2.577532 | 0.016549 |
| H | -6.068057 | -1.955661 | -2.224342 |
| H | -5.569308 | -0.479125 | -1.385283 |
| H | -4.535041 | -1.173538 | -2.646896 |
| H | -0.773302 | -1.880124 | 0.861989 |
| H | 0.189696 | -1.275200 | -1.976578 |
| H | 2.522491 | -1.254666 | -2.372199 |
| H | 4.852913 | -1.262566 | -1.576564 |
| H | 3.480846 | -1.604168 | 2.468748 |
| H | 1.157326 | -1.581232 | 1.707032 |
| H | 6.458366 | -1.272230 | 0.181210 |
| H | 5.910527 | -0.659438 | 1.751104 |
| H | 6.003928 | -2.394048 | 1.471752 |


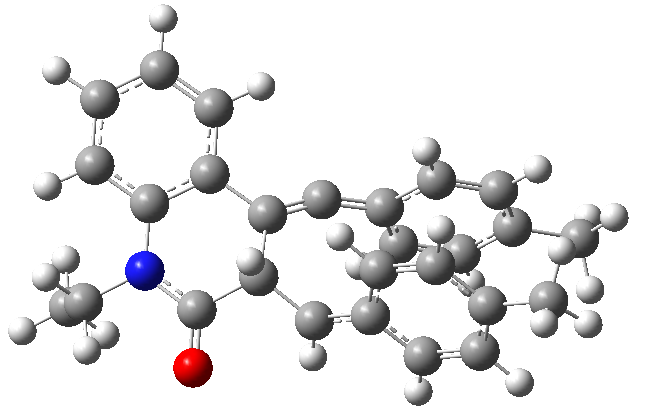


| C | -3.957772 | 1.287893 | 2.807785 |
| --- | --- | --- | --- |
| C | -4.967532 | 0.407650 | 2.442631 |
| C | -4.756723 | -0.536069 | 1.448693 |
| C | -3.523113 | -0.623894 | 0.799708 |
| C | -2.501323 | 0.268671 | 1.164065 |
| C | -2.734482 | 1.209532 | 2.164325 |
| N | -3.314738 | -1.550354 | -0.247298 |
| C | -1.208902 | 0.189513 | 0.467826 |
| C | -0.390192 | 1.199652 | 0.315170 |
| C | 0.725984 | 1.883365 | -0.128979 |
| C | 1.845337 | 2.101931 | 0.713686 |
| C | 2.967668 | 2.748685 | 0.237595 |
| C | 3.037235 | 3.219613 | -1.077505 |
| C | 1.924630 | 3.022759 | -1.908705 |
| C | 0.793514 | 2.380468 | -1.458474 |
| C | 4.252594 | 3.916726 | -1.596826 |
| C | -2.075174 | -1.798709 | -0.790417 |
| C | -4.460597 | -2.220545 | -0.872429 |
| C | -5.191812 | -1.316795 | -1.841372 |
| C | -0.882101 | -1.212438 | -0.054067 |
| O | -1.932787 | -2.496650 | -1.777317 |
| C | 0.348776 | -1.265128 | -0.883646 |
| C | 1.656726 | -1.332864 | -0.374001 |
| C | 2.760274 | -1.229056 | -1.259315 |
| C | 4.058089 | -1.243693 | -0.795122 |
| C | 4.340554 | -1.369694 | 0.570557 |
| C | 3.259038 | -1.478662 | 1.454373 |
| C | 1.954363 | -1.458957 | 1.007387 |
| C | 5.747387 | -1.409088 | 1.074504 |
| H | -4.121345 | 2.025194 | 3.587214 |
| H | -5.935303 | 0.450491 | 2.933184 |
| H | -5.571665 | -1.195901 | 1.176815 |
| H | -1.923585 | 1.881797 | 2.434718 |
| H | 1.812145 | 1.737105 | 1.736541 |
| H | 3.821119 | 2.891267 | 0.896909 |
| H | 1.964754 | 3.381859 | -2.935276 |
| H | -0.055781 | 2.225594 | -2.118485 |
| H | 4.635511 | 3.425726 | -2.499094 |
| H | 4.027548 | 4.953787 | -1.873759 |
| H | 5.054370 | 3.934481 | -0.853596 |
| H | -4.064007 | -3.092847 | -1.392241 |
| H | -5.122344 | -2.593865 | -0.087101 |
| H | -6.037572 | -1.845752 | -2.291326 |
| H | -5.579276 | -0.419532 | -1.347592 |
| H | -4.521735 | -0.999898 | -2.647579 |
| H | -0.775408 | -1.851278 | 0.841924 |
| H | 0.225654 | -1.154632 | -1.959138 |
| H | 2.562184 | -1.122237 | -2.323831 |
| H | 4.882312 | -1.152213 | -1.499537 |
| H | 3.458857 | -1.572291 | 2.520332 |
| H | 1.144468 | -1.529498 | 1.729474 |
| H | 6.450200 | -1.014415 | 0.334702 |
| H | 5.858849 | -0.829492 | 1.997247 |
| H | 6.058188 | -2.436002 | 1.306107 |


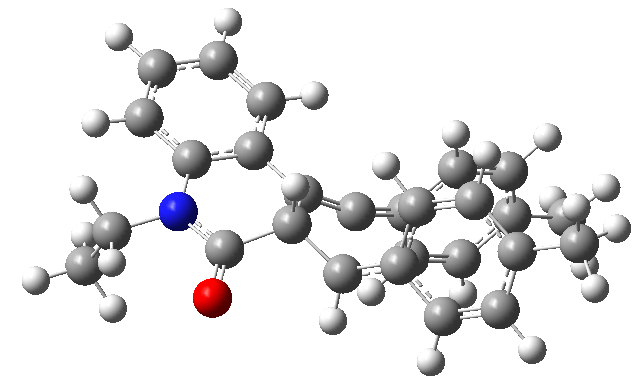


| C | -3.574058 | 2.000964 | 2.110093 |
| --- | --- | --- | --- |
| C | -4.643806 | 1.120645 | 2.014588 |
| C | -4.520063 | -0.077494 | 1.326400 |
| C | -3.312141 | -0.427817 | 0.719684 |
| C | -2.224204 | 0.458488 | 0.829849 |
| C | -2.370658 | 1.660452 | 1.516222 |
| N | -3.196065 | -1.616610 | -0.042953 |
| C | -0.964832 | 0.053580 | 0.205288 |
| C | 0.006473 | 0.812202 | -0.256608 |
| C | 0.792880 | 1.929322 | -0.489861 |
| C | 1.706867 | 2.379676 | 0.496137 |
| C | 2.532800 | 3.458153 | 0.249381 |
| C | 2.501823 | 4.136907 | -0.972540 |
| C | 1.606158 | 3.687248 | -1.953147 |
| C | 0.773836 | 2.612827 | -1.733021 |
| C | 3.381835 | 5.315999 | -1.234289 |
| C | -1.991165 | -2.097146 | -0.515799 |
| C | -4.408028 | -2.325294 | -0.472425 |
| C | -5.121146 | -1.611088 | -1.599956 |
| C | -0.760653 | -1.419997 | 0.043309 |
| O | -1.919690 | -3.032062 | -1.291384 |
| C | 0.475608 | -1.592095 | -0.787158 |
| C | 1.766358 | -1.861408 | -0.278303 |
| C | 2.849771 | -2.041071 | -1.172891 |
| C | 4.128812 | -2.274829 | -0.713494 |
| C | 4.409842 | -2.340700 | 0.656265 |
| C | 3.346906 | -2.162314 | 1.548573 |
| C | 2.061094 | -1.925838 | 1.104751 |
| C | 5.803419 | -2.564157 | 1.149157 |
| H | -3.676785 | 2.938238 | 2.647635 |
| H | -5.594917 | 1.365032 | 2.478341 |
| H | -5.382995 | -0.728962 | 1.260520 |
| H | -1.511519 | 2.323782 | 1.586251 |
| H | 1.747911 | 1.857823 | 1.449065 |
| H | 3.225144 | 3.788369 | 1.020803 |
| H | 1.573133 | 4.201604 | -2.911632 |
| H | 0.091688 | 2.274828 | -2.508011 |
| H | 2.802260 | 6.247655 | -1.249526 |
| H | 4.154413 | 5.419726 | -0.467127 |
| H | 3.876092 | 5.235681 | -2.208765 |
| H | -4.086701 | -3.315756 | -0.795434 |
| H | -5.059187 | -2.471440 | 0.393119 |
| H | -6.007288 | -2.175320 | -1.906365 |
| H | -5.446684 | -0.607001 | -1.308142 |
| H | -4.462766 | -1.515905 | -2.470066 |
| H | -0.636573 | -1.867789 | 1.044650 |
| H | 0.344002 | -1.580731 | -1.867384 |
| H | 2.653258 | -1.997583 | -2.241913 |
| H | 4.938233 | -2.414468 | -1.427319 |
| H | 3.543099 | -2.210743 | 2.617962 |
| H | 1.267931 | -1.782947 | 1.834391 |
| H | 6.386287 | -3.166645 | 0.445307 |
| H | 6.337231 | -1.613509 | 1.277094 |
| H | 5.809806 | -3.069178 | 2.120118 |


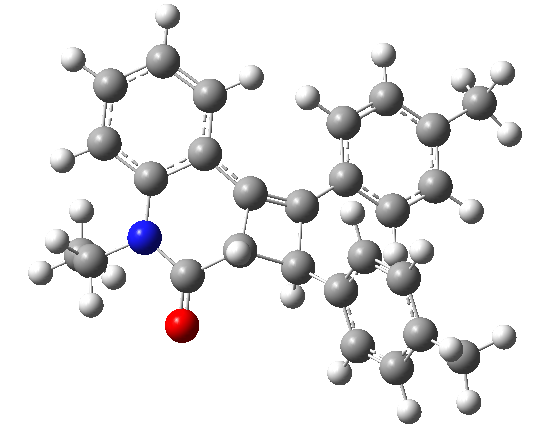


| C | -3.809802 | 1.725375 | 2.232495 |
| --- | --- | --- | --- |
| C | -4.829159 | 0.880057 | 1.820641 |
| C | -4.564072 | -0.201129 | 0.990608 |
| C | -3.266893 | -0.465132 | 0.551124 |
| C | -2.226959 | 0.408286 | 0.958335 |
| C | -2.517481 | 1.481691 | 1.797660 |
| N | -3.008549 | -1.544723 | -0.336523 |
| C | -0.919345 | 0.045743 | 0.469516 |
| C | 0.225560 | 0.507460 | -0.094983 |
| C | 0.871455 | 1.781913 | -0.337515 |
| C | 2.060277 | 1.822784 | -1.074427 |
| C | 2.700859 | 3.025982 | -1.330033 |
| C | 2.182514 | 4.230966 | -0.862358 |
| C | 0.989444 | 4.192392 | -0.135285 |
| C | 0.347939 | 2.996603 | 0.126523 |
| C | 2.874018 | 5.531712 | -1.118764 |
| C | -1.738205 | -1.991532 | -0.670660 |
| C | -4.131555 | -2.199506 | -1.018862 |
| C | -4.670653 | -1.365737 | -2.161016 |
| C | -0.639187 | -1.381870 | 0.140049 |
| O | -1.547913 | -2.830698 | -1.531913 |
| C | 0.660280 | -0.878590 | -0.563442 |
| C | 1.963716 | -1.445924 | -0.086883 |
| C | 2.715561 | -2.281641 | -0.906154 |
| C | 3.913995 | -2.828849 | -0.461490 |
| C | 4.399195 | -2.553015 | 0.813336 |
| C | 3.641776 | -1.714111 | 1.634034 |
| C | 2.446800 | -1.169624 | 1.193315 |
| C | 5.696478 | -3.120576 | 1.298995 |
| H | -4.018014 | 2.563610 | 2.889652 |
| H | -5.850112 | 1.054384 | 2.146923 |
| H | -5.390926 | -0.831391 | 0.687313 |
| H | -1.702734 | 2.114319 | 2.137172 |
| H | 2.486613 | 0.898140 | -1.455455 |
| H | 3.623437 | 3.031901 | -1.905978 |
| H | 0.563040 | 5.123785 | 0.232094 |
| H | -0.577630 | 2.999833 | 0.693034 |
| H | 3.693717 | 5.415989 | -1.833416 |
| H | 2.179345 | 6.280770 | -1.514387 |
| H | 3.293521 | 5.945658 | -0.193963 |
| H | -3.752631 | -3.150967 | -1.392781 |
| H | -4.907200 | -2.434732 | -0.285530 |
| H | -5.501722 | -1.882096 | -2.651450 |
| H | -5.034055 | -0.390831 | -1.819272 |
| H | -3.889834 | -1.193545 | -2.909666 |
| H | -0.440831 | -2.032571 | 1.005104 |
| H | 0.580332 | -0.990304 | -1.653009 |
| H | 2.355522 | -2.509402 | -1.907738 |
| H | 4.485371 | -3.481037 | -1.118672 |
| H | 4.002914 | -1.483475 | 2.634716 |
| H | 1.877670 | -0.513031 | 1.849538 |
| H | 6.118539 | -3.828341 | 0.579807 |
| H | 6.438382 | -2.330546 | 1.465194 |
| H | 5.570242 | -3.643122 | 2.253890 |


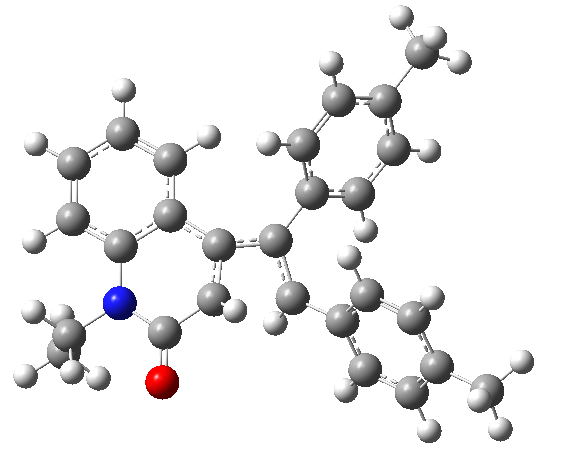


| C | -3.891008 | 2.549669 | 0.252340 |
| --- | --- | --- | --- |
| C | -4.980391 | 1.688857 | 0.176267 |
| C | -4.793921 | 0.317984 | 0.150088 |
| C | -3.509868 | -0.236240 | 0.216234 |
| C | -2.400053 | 0.637084 | 0.318658 |
| C | -2.617333 | 2.019010 | 0.316060 |
| N | -3.327530 | -1.630181 | 0.131147 |
| C | -1.090667 | 0.026354 | 0.344998 |
| C | 0.182878 | 0.401276 | -0.067588 |
| C | 0.898390 | 1.672207 | -0.006908 |
| C | 1.821455 | 2.019361 | -0.998315 |
| C | 2.523114 | 3.213558 | -0.932737 |
| C | 2.328671 | 4.107112 | 0.118936 |
| C | 1.409164 | 3.760241 | 1.111706 |
| C | 0.710527 | 2.566915 | 1.054110 |
| C | 3.068999 | 5.404960 | 0.187910 |
| C | -2.094774 | -2.261350 | 0.258321 |
| C | -4.479814 | -2.506862 | -0.107955 |
| C | -4.849571 | -2.570432 | -1.574598 |
| C | -0.967511 | -1.378222 | 0.540784 |
| O | -1.977574 | -3.476101 | 0.151346 |
| C | 0.724439 | -0.852406 | -0.541257 |
| C | 2.077670 | -1.336501 | -0.296481 |
| C | 2.653977 | -2.281847 | -1.149351 |
| C | 3.926428 | -2.775425 | -0.907447 |
| C | 4.661137 | -2.349520 | 0.197695 |
| C | 4.077045 | -1.417639 | 1.061928 |
| C | 2.808457 | -0.923269 | 0.825397 |
| C | 6.034191 | -2.874527 | 0.469368 |
| H | -4.036461 | 3.625219 | 0.259418 |
| H | -5.989840 | 2.085884 | 0.126615 |
| H | -5.665290 | -0.319065 | 0.064574 |
| H | -1.758062 | 2.680313 | 0.370761 |
| H | 1.980920 | 1.344933 | -1.836278 |
| H | 3.234507 | 3.462284 | -1.717235 |
| H | 1.253058 | 4.437969 | 1.948708 |
| H | 0.022844 | 2.303653 | 1.854637 |
| H | 3.861532 | 5.454765 | -0.563952 |
| H | 2.395382 | 6.253417 | 0.018256 |
| H | 3.523910 | 5.553008 | 1.173540 |
| H | -4.193291 | -3.496155 | 0.250218 |
| H | -5.315788 | -2.172753 | 0.510894 |
| H | -5.711219 | -3.228762 | -1.722136 |
| H | -5.104727 | -1.583667 | -1.974298 |
| H | -4.015245 | -2.969030 | -2.161285 |
| H | -0.261360 | -1.782232 | 1.261711 |
| H | 0.228458 | -1.311214 | -1.395636 |
| H | 2.089757 | -2.627456 | -2.012713 |
| H | 4.360650 | -3.507016 | -1.584782 |
| H | 4.633314 | -1.087505 | 1.936825 |
| H | 2.363630 | -0.211027 | 1.517133 |
| H | 6.347890 | -3.592006 | -0.293729 |
| H | 6.770191 | -2.062561 | 0.493613 |
| H | 6.081763 | -3.373760 | 1.443927 |


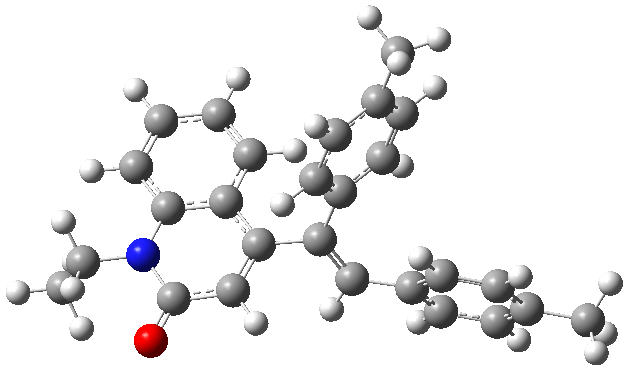


| C | -2.674910 | 1.901551 | -2.184327 |
| --- | --- | --- | --- |
| C | -4.028590 | 1.669877 | -1.944906 |
| C | -4.431888 | 0.714366 | -1.034659 |
| C | -3.485919 | -0.036502 | -0.319837 |
| C | -2.110795 | 0.206400 | -0.542551 |
| C | -1.736488 | 1.170945 | -1.489666 |
| N | -3.879913 | -1.017074 | 0.582880 |
| C | -1.138591 | -0.584052 | 0.178530 |
| C | 0.321636 | -0.339865 | 0.047974 |
| C | 0.809787 | 1.005664 | 0.435039 |
| C | 1.676807 | 1.732451 | -0.382391 |
| C | 2.131965 | 2.983850 | 0.001226 |
| C | 1.736577 | 3.556066 | 1.210640 |
| C | 0.860881 | 2.833607 | 2.019756 |
| C | 0.395834 | 1.584724 | 1.635499 |
| C | 2.207761 | 4.916990 | 1.616841 |
| C | -2.972702 | -1.849632 | 1.231226 |
| C | -5.300460 | -1.272461 | 0.845331 |
| C | -5.906376 | -2.229134 | -0.159712 |
| C | -1.574196 | -1.561875 | 1.005725 |
| O | -3.354393 | -2.755645 | 1.966061 |
| C | 1.103943 | -1.354683 | -0.363824 |
| C | 2.565733 | -1.441753 | -0.429237 |
| C | 3.145856 | -2.238634 | -1.419120 |
| C | 4.523401 | -2.377129 | -1.516115 |
| C | 5.371803 | -1.743642 | -0.612400 |
| C | 4.791755 | -0.971091 | 0.397387 |
| C | 3.419843 | -0.820665 | 0.491439 |
| C | 6.858027 | -1.896158 | -0.692021 |
| H | -2.363110 | 2.642865 | -2.912724 |
| H | -4.782070 | 2.234726 | -2.485816 |
| H | -5.491629 | 0.542935 | -0.893490 |
| H | -0.680924 | 1.335077 | -1.679331 |
| H | 1.993769 | 1.310226 | -1.333178 |
| H | 2.806564 | 3.533763 | -0.651461 |
| H | 0.536970 | 3.259500 | 2.967236 |
| H | -0.286330 | 1.041203 | 2.285551 |
| H | 3.109791 | 5.208831 | 1.071299 |
| H | 1.441669 | 5.675469 | 1.414316 |
| H | 2.425131 | 4.960909 | 2.688935 |
| H | -5.356287 | -1.696534 | 1.848803 |
| H | -5.827387 | -0.316092 | 0.868743 |
| H | -6.961977 | -2.402393 | 0.070826 |
| H | -5.842233 | -1.841881 | -1.181877 |
| H | -5.391108 | -3.194330 | -0.128914 |
| H | -0.874799 | -2.171063 | 1.570030 |
| H | 0.589410 | -2.255627 | -0.700911 |
| H | 2.501085 | -2.752636 | -2.128721 |
| H | 4.948146 | -2.994974 | -2.304067 |
| H | 5.435758 | -0.485187 | 1.127815 |
| H | 3.002946 | -0.224773 | 1.298471 |
| H | 7.159608 | -2.431374 | -1.596803 |
| H | 7.358451 | -0.921516 | -0.690090 |
| H | 7.245217 | -2.452315 | 0.170021 |


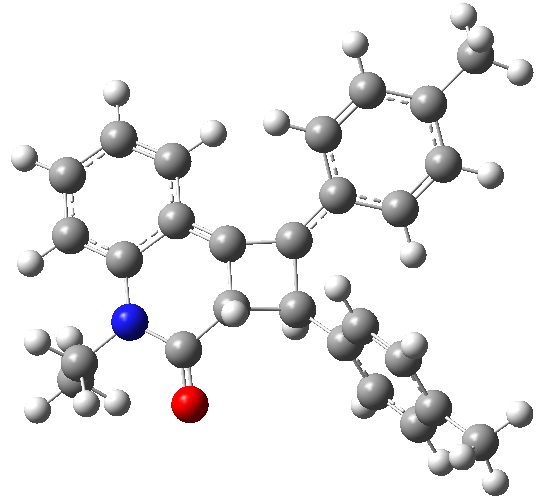


| C | -3.812021 | 2.956391 | 0.032397 |
| --- | --- | --- | --- |
| C | -4.903881 | 2.102259 | 0.220251 |
| C | -4.713365 | 0.725832 | 0.352333 |
| C | -3.441069 | 0.176052 | 0.350518 |
| C | -2.300884 | 1.059261 | 0.276789 |
| C | -2.537917 | 2.444745 | 0.042635 |
| N | -3.247307 | -1.223344 | 0.314223 |
| C | -1.047012 | 0.499569 | 0.422126 |
| C | 0.308885 | 0.682874 | -0.136777 |
| C | 1.259086 | 1.696686 | -0.170375 |
| C | 2.495880 | 1.526056 | -0.859592 |
| C | 3.434749 | 2.531735 | -0.889209 |
| C | 3.224863 | 3.753540 | -0.232509 |
| C | 2.014380 | 3.926623 | 0.461111 |
| C | 1.058299 | 2.940946 | 0.499063 |
| C | 4.250984 | 4.838069 | -0.248694 |
| C | -2.009096 | -1.830056 | 0.193282 |
| C | -4.409678 | -2.112013 | 0.181050 |
| C | -4.921409 | -2.199682 | -1.241087 |
| C | -0.836883 | -0.967700 | 0.520253 |
| O | -1.885704 | -3.006743 | -0.095336 |
| C | 0.365935 | -0.808807 | -0.471354 |
| C | 1.599689 | -1.617212 | -0.207939 |
| C | 2.052134 | -2.537443 | -1.147024 |
| C | 3.174767 | -3.319517 | -0.897415 |
| C | 3.878699 | -3.202173 | 0.296497 |
| C | 3.423522 | -2.273137 | 1.235945 |
| C | 2.306624 | -1.493352 | 0.989730 |
| C | 5.091169 | -4.033233 | 0.578605 |
| H | -3.971977 | 4.019131 | -0.122398 |
| H | -5.914107 | 2.499271 | 0.227193 |
| H | -5.588892 | 0.090851 | 0.415125 |
| H | -1.679742 | 3.093757 | -0.102813 |
| H | 2.687209 | 0.590198 | -1.378506 |
| H | 4.364953 | 2.379467 | -1.432784 |
| H | 1.839772 | 4.864098 | 0.986183 |
| H | 0.142530 | 3.095869 | 1.063846 |
| H | 5.087327 | 4.590616 | -0.908581 |
| H | 3.822622 | 5.789777 | -0.584703 |
| H | 4.658937 | 5.014666 | 0.754743 |
| H | -4.086770 | -3.095278 | 0.526129 |
| H | -5.184634 | -1.770423 | 0.871433 |
| H | -5.784840 | -2.870262 | -1.290874 |
| H | -5.231827 | -1.222762 | -1.624704 |
| H | -4.147157 | -2.596093 | -1.905417 |
| H | -0.452928 | -1.286558 | 1.502521 |
| H | 0.012718 | -0.989143 | -1.498264 |
| H | 1.513470 | -2.649725 | -2.085873 |
| H | 3.507958 | -4.037348 | -1.643906 |
| H | 3.959469 | -2.164130 | 2.176969 |
| H | 1.977057 | -0.774691 | 1.737902 |
| H | 5.251030 | -4.786273 | -0.198317 |
| H | 5.994069 | -3.413787 | 0.634865 |
| H | 5.001846 | -4.551751 | 1.539705 |


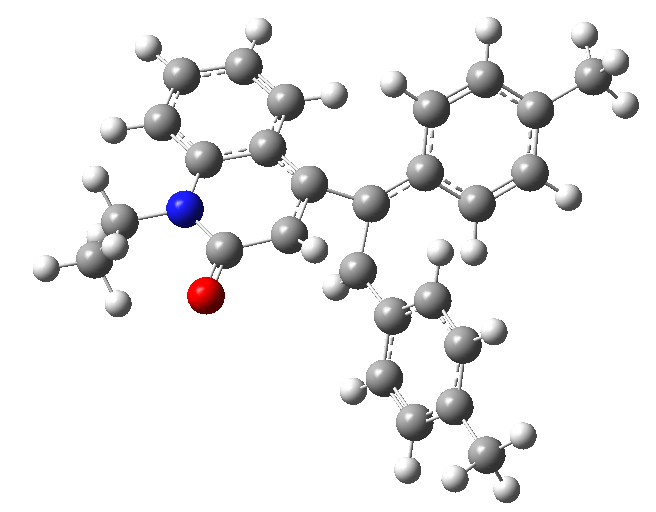


| C | 3.468773 | 2.942024 | 0.504653 |
| --- | --- | --- | --- |
| C | 4.642674 | 2.194233 | 0.384972 |
| C | 4.592112 | 0.848759 | 0.048841 |
| C | 3.373531 | 0.219820 | -0.207000 |
| C | 2.171049 | 0.992427 | -0.154066 |
| C | 2.255927 | 2.346015 | 0.253018 |
| N | 3.301158 | -1.154648 | -0.456768 |
| C | 0.955133 | 0.359789 | -0.467269 |
| C | -0.393875 | 0.619344 | 0.057815 |
| C | -1.245055 | 1.724539 | 0.033608 |
| C | -2.444648 | 1.749834 | 0.800353 |
| C | -3.274424 | 2.849428 | 0.782938 |
| C | -2.986174 | 3.973711 | -0.001487 |
| C | -1.819533 | 3.947384 | -0.784950 |
| C | -0.970570 | 2.867369 | -0.774820 |
| C | -3.895690 | 5.157142 | -0.036191 |
| C | 2.109383 | -1.849165 | -0.618279 |
| C | 4.524463 | -1.964807 | -0.492735 |
| C | 4.954927 | -2.414901 | 0.886921 |
| C | 0.893561 | -1.036116 | -0.680823 |
| O | 2.086071 | -3.068343 | -0.726813 |
| C | -0.592544 | -0.733241 | 0.674698 |
| C | -1.730509 | -1.595954 | 0.433613 |
| C | -1.947749 | -2.714179 | 1.257333 |
| C | -2.991611 | -3.584409 | 1.013664 |
| C | -3.862969 | -3.386981 | -0.063904 |
| C | -3.646276 | -2.281192 | -0.884848 |
| C | -2.603155 | -1.399429 | -0.647355 |
| C | -4.977177 | -4.349350 | -0.326436 |
| H | 3.512821 | 3.985081 | 0.802136 |
| H | 5.606384 | 2.655124 | 0.579161 |
| H | 5.519420 | 0.289551 | 0.015850 |
| H | 1.338902 | 2.917632 | 0.352838 |
| H | -2.692036 | 0.894924 | 1.423492 |
| H | -4.175530 | 2.849870 | 1.392282 |
| H | -1.589519 | 4.804961 | -1.414401 |
| H | -0.084881 | 2.870021 | -1.405015 |
| H | -4.668258 | 5.092714 | 0.735234 |
| H | -3.342082 | 6.091531 | 0.111575 |
| H | -4.400461 | 5.242409 | -1.007055 |
| H | 4.305730 | -2.829953 | -1.120264 |
| H | 5.304352 | -1.388805 | -0.995607 |
| H | 5.868353 | -3.014024 | 0.821897 |
| H | 5.153902 | -1.564511 | 1.547105 |
| H | 4.177574 | -3.031577 | 1.349063 |
| H | 0.135063 | -1.461056 | -1.337795 |
| H | -0.113654 | -0.855216 | 1.651643 |
| H | -1.280755 | -2.885480 | 2.099466 |
| H | -3.144904 | -4.439614 | 1.668787 |
| H | -4.314348 | -2.107440 | -1.725706 |
| H | -2.460922 | -0.542780 | -1.301641 |
| H | -4.594161 | -5.300461 | -0.716684 |
| H | -5.528783 | -4.582836 | 0.591023 |
| H | -5.686326 | -3.953346 | -1.059101 |


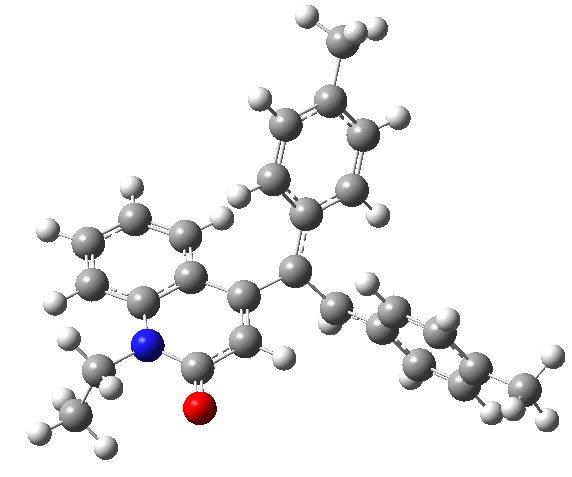


| C | 3.460609 | 2.110279 | 1.446864 |
| --- | --- | --- | --- |
| C | 4.612196 | 1.473629 | 0.985433 |
| C | 4.530215 | 0.280939 | 0.297629 |
| C | 3.283798 | -0.309136 | 0.032481 |
| C | 2.108804 | 0.352145 | 0.459241 |
| C | 2.231608 | 1.547132 | 1.184707 |
| N | 3.197217 | -1.529576 | -0.625395 |
| C | 0.822263 | -0.267667 | 0.194855 |
| C | -0.448385 | 0.375080 | 0.559120 |
| C | -0.803058 | 1.695570 | 0.141756 |
| C | -1.923049 | 2.353659 | 0.698264 |
| C | -2.284445 | 3.623322 | 0.290021 |
| C | -1.572524 | 4.298140 | -0.703889 |
| C | -0.476369 | 3.642468 | -1.280890 |
| C | -0.096114 | 2.382142 | -0.875619 |
| C | -1.969484 | 5.663104 | -1.162823 |
| C | 1.992749 | -2.211394 | -0.783100 |
| C | 4.399778 | -2.207339 | -1.121699 |
| C | 5.069532 | -3.048730 | -0.056409 |
| C | 0.808084 | -1.512876 | -0.352149 |
| O | 1.960423 | -3.329440 | -1.291095 |
| C | -1.374458 | -0.437293 | 1.359794 |
| C | -2.502483 | -1.110850 | 0.851320 |
| C | -3.312892 | -1.898681 | 1.706954 |
| C | -4.419837 | -2.566313 | 1.226257 |
| C | -4.784232 | -2.493105 | -0.123228 |
| C | -3.988523 | -1.715811 | -0.977682 |
| C | -2.881682 | -1.039747 | -0.515263 |
| C | -5.984482 | -3.209455 | -0.650935 |
| H | 3.530660 | 3.034086 | 2.011827 |
| H | 5.589422 | 1.905062 | 1.181370 |
| H | 5.445594 | -0.204884 | -0.016670 |
| H | 1.331417 | 2.031251 | 1.549324 |
| H | -2.497714 | 1.853426 | 1.473906 |
| H | -3.142506 | 4.108969 | 0.749913 |
| H | 0.080471 | 4.141882 | -2.071494 |
| H | 0.748462 | 1.900770 | -1.361249 |
| H | -2.757907 | 6.085099 | -0.533341 |
| H | -1.117246 | 6.352040 | -1.149659 |
| H | -2.340848 | 5.641533 | -2.194776 |
| H | 4.075657 | -2.839315 | -1.949810 |
| H | 5.076547 | -1.454969 | -1.533275 |
| H | 5.954647 | -3.544698 | -0.466484 |
| H | 5.386183 | -2.445364 | 0.800500 |
| H | 4.386106 | -3.822683 | 0.306823 |
| H | -0.126556 | -2.035179 | -0.532682 |
| H | -1.150286 | -0.559178 | 2.422174 |
| H | -3.043958 | -1.970761 | 2.758451 |
| H | -5.023901 | -3.165640 | 1.904051 |
| H | -4.257459 | -1.649806 | -2.030306 |
| H | -2.282612 | -0.444912 | -1.201260 |
| H | -6.455894 | -3.826257 | 0.119267 |
| H | -6.736690 | -2.503074 | -1.023042 |
| H | -5.721229 | -3.860129 | -1.493092 |


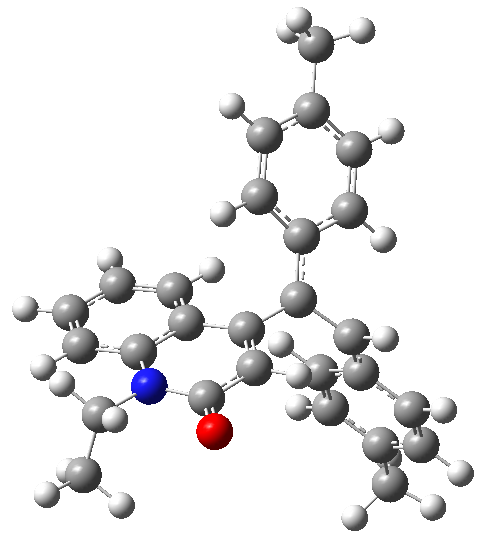


| C | -3.058911 | 1.249941 | -2.109100 |
| --- | --- | --- | --- |
| C | -4.420830 | 1.168770 | -1.821384 |
| C | -4.866239 | 0.483501 | -0.710642 |
| C | -3.957310 | -0.134438 | 0.164043 |
| C | -2.572115 | -0.024802 | -0.100279 |
| C | -2.157051 | 0.654890 | -1.255663 |
| N | -4.402050 | -0.853697 | 1.266007 |
| C | -1.634774 | -0.676449 | 0.796928 |
| C | -0.178554 | -0.577518 | 0.630982 |
| C | 0.519274 | 0.671122 | 0.555201 |
| C | 1.891970 | 0.708314 | 0.222405 |
| C | 2.586383 | 1.901369 | 0.176166 |
| C | 1.962985 | 3.118032 | 0.468175 |
| C | 0.609124 | 3.088242 | 0.821738 |
| C | -0.098877 | 1.905575 | 0.863840 |
| C | 2.710119 | 4.409529 | 0.393675 |
| C | -3.540974 | -1.587689 | 2.078236 |
| C | -5.829638 | -0.931834 | 1.592439 |
| C | -6.532054 | -2.048649 | 0.850032 |
| C | -2.134462 | -1.456172 | 1.793299 |
| O | -3.973386 | -2.277254 | 2.998423 |
| C | 0.574471 | -1.837828 | 0.720715 |
| C | 0.765107 | -2.725040 | -0.357288 |
| C | 1.439537 | -3.956951 | -0.168427 |
| C | 1.623009 | -4.837632 | -1.214138 |
| C | 1.157436 | -4.545949 | -2.501273 |
| C | 0.498309 | -3.323908 | -2.699979 |
| C | 0.302639 | -2.434512 | -1.666955 |
| C | 1.334556 | -5.506146 | -3.632338 |
| H | -2.712863 | 1.769489 | -2.996684 |
| H | -5.146706 | 1.632236 | -2.482963 |
| H | -5.932324 | 0.413824 | -0.533725 |
| H | -1.095921 | 0.704084 | -1.477708 |
| H | 2.406193 | -0.222280 | -0.005264 |
| H | 3.641005 | 1.897118 | -0.090734 |
| H | 0.108806 | 4.019916 | 1.078142 |
| H | -1.142249 | 1.923171 | 1.164981 |
| H | 3.783196 | 4.262300 | 0.548538 |
| H | 2.585930 | 4.880540 | -0.590001 |
| H | 2.348546 | 5.125732 | 1.138255 |
| H | -5.889651 | -1.100079 | 2.668779 |
| H | -6.284904 | 0.040457 | 1.391086 |
| H | -7.590150 | -2.079831 | 1.127465 |
| H | -6.470681 | -1.918724 | -0.235176 |
| H | -6.087084 | -3.016216 | 1.102183 |
| H | -1.474958 | -1.992402 | 2.468962 |
| H | 0.973501 | -2.131863 | 1.694611 |
| H | 1.805880 | -4.204048 | 0.825573 |
| H | 2.138440 | -5.779870 | -1.040229 |
| H | 0.132853 | -3.079825 | -3.695853 |
| H | -0.214846 | -1.494927 | -1.848885 |
| H | 1.934999 | -6.370633 | -3.335236 |
| H | 0.366953 | -5.878177 | -3.991172 |
| H | 1.825729 | -5.030311 | -4.489136 |


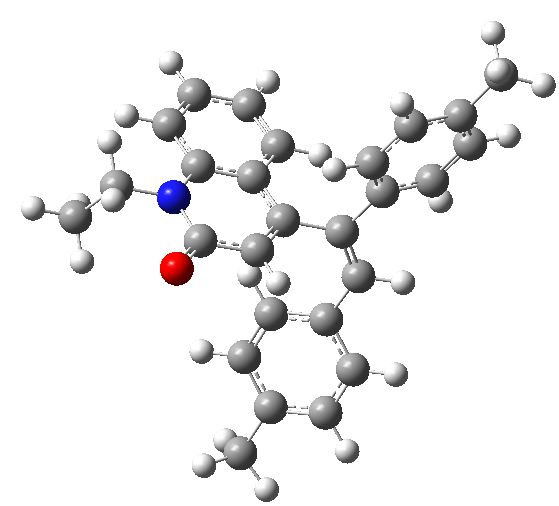


| C | -2.734172 | 1.436223 | -2.511225 |
| --- | --- | --- | --- |
| C | -4.091632 | 1.266733 | -2.238855 |
| C | -4.512004 | 0.521304 | -1.155796 |
| C | -3.577584 | -0.076334 | -0.295939 |
| C | -2.201199 | 0.099637 | -0.561974 |
| C | -1.806302 | 0.851973 | -1.677936 |
| N | -3.977223 | -0.838441 | 0.794984 |
| C | -1.240230 | -0.538953 | 0.303020 |
| C | 0.216690 | -0.340889 | 0.085213 |
| C | 0.760238 | 0.974224 | 0.491941 |
| C | 1.859645 | 1.541832 | -0.161916 |
| C | 2.378358 | 2.761432 | 0.240464 |
| C | 1.815702 | 3.473734 | 1.299743 |
| C | 0.710684 | 2.917678 | 1.940958 |
| C | 0.186130 | 1.697561 | 1.541916 |
| C | 2.356546 | 4.808237 | 1.705458 |
| C | -3.075152 | -1.506297 | 1.620872 |
| C | -5.400417 | -1.024876 | 1.099142 |
| C | -6.007249 | -2.173430 | 0.321320 |
| C | -1.673837 | -1.295596 | 1.333268 |
| O | -3.465610 | -2.219564 | 2.539694 |
| C | 1.021333 | -1.301868 | -0.407054 |
| C | 0.705659 | -2.631450 | -0.925828 |
| C | 1.740567 | -3.571871 | -0.980293 |
| C | 1.529233 | -4.855398 | -1.461058 |
| C | 0.277313 | -5.248152 | -1.926163 |
| C | -0.752435 | -4.304260 | -1.900014 |
| C | -0.548442 | -3.025164 | -1.415272 |
| C | 0.028671 | -6.627625 | -2.447780 |
| H | -2.413979 | 2.016351 | -3.370389 |
| H | -4.836087 | 1.717353 | -2.888568 |
| H | -5.574618 | 0.397008 | -0.988339 |
| H | -0.744617 | 0.964837 | -1.880970 |
| H | 2.304138 | 1.029967 | -1.011495 |
| H | 3.233015 | 3.180674 | -0.286330 |
| H | 0.252642 | 3.451260 | 2.771081 |
| H | -0.673079 | 1.290967 | 2.069141 |
| H | 3.430904 | 4.881026 | 1.510789 |
| H | 1.870637 | 5.616662 | 1.145134 |
| H | 2.184462 | 5.004019 | 2.768223 |
| H | -5.463438 | -1.220402 | 2.170336 |
| H | -5.921518 | -0.083632 | 0.910680 |
| H | -7.064803 | -2.287853 | 0.578111 |
| H | -5.937120 | -2.015898 | -0.759915 |
| H | -5.497434 | -3.111645 | 0.562550 |
| H | -0.974972 | -1.795542 | 1.997591 |
| H | 2.088927 | -1.079892 | -0.409172 |
| H | 2.729789 | -3.285425 | -0.630058 |
| H | 2.352883 | -5.565503 | -1.480038 |
| H | -1.734107 | -4.583074 | -2.278152 |
| H | -1.372542 | -2.319488 | -1.438172 |
| H | 0.932419 | -7.241900 | -2.407049 |
| H | -0.750734 | -7.134776 | -1.867435 |
| H | -0.316669 | -6.602995 | -3.487791 |


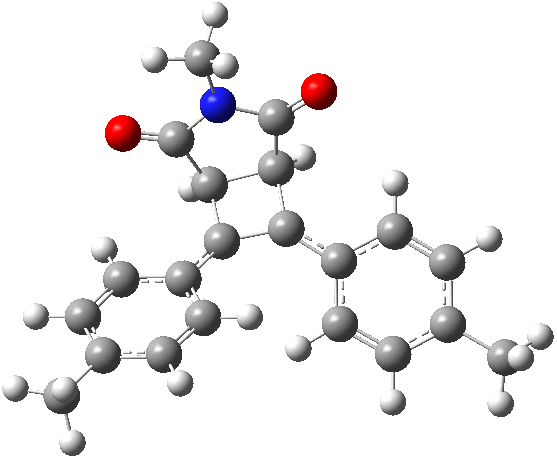


| C | 0.794909 | 0.511906 | -0.441986 |
| --- | --- | --- | --- |
| C | -0.607466 | 0.154576 | -0.744019 |
| C | -0.856076 | 1.533926 | -1.338891 |
| C | 0.553768 | 1.947034 | -0.858350 |
| C | 2.000548 | -0.157443 | -0.274014 |
| C | -1.497687 | -0.846387 | -0.372377 |
| C | 3.202101 | 0.582671 | -0.052991 |
| C | 4.404973 | -0.056041 | 0.115234 |
| C | 4.507837 | -1.458383 | 0.058663 |
| C | 3.338921 | -2.192703 | -0.192057 |
| C | 2.119189 | -1.577356 | -0.354959 |
| C | -2.823068 | -0.876215 | -0.902407 |
| C | -3.704027 | -1.870102 | -0.549570 |
| C | -3.346503 | -2.884247 | 0.354028 |
| C | -2.052288 | -2.850409 | 0.901124 |
| C | -1.149945 | -1.873447 | 0.558917 |
| C | 5.818249 | -2.138807 | 0.274346 |
| C | -4.302564 | -3.963452 | 0.738440 |
| C | -1.813015 | 2.345832 | -0.496529 |
| N | -1.062024 | 3.030786 | 0.451296 |
| C | 0.306182 | 2.888068 | 0.295563 |
| O | 1.131824 | 3.426877 | 0.992904 |
| O | -3.014845 | 2.392247 | -0.583715 |
| C | -1.639851 | 3.861369 | 1.486010 |
| H | -1.095094 | 1.597583 | -2.404007 |
| H | 1.268263 | 2.377693 | -1.566238 |
| H | 3.144270 | 1.667039 | 0.004536 |
| H | 5.304151 | 0.529542 | 0.295339 |
| H | 3.405963 | -3.275787 | -0.271732 |
| H | 1.241469 | -2.172420 | -0.586598 |
| H | -3.128026 | -0.104646 | -1.603575 |
| H | -4.704526 | -1.875407 | -0.976608 |
| H | -1.767730 | -3.613223 | 1.623462 |
| H | -0.170827 | -1.856548 | 1.027662 |
| H | 6.651435 | -1.528961 | -0.089661 |
| H | 5.999008 | -2.321256 | 1.342223 |
| H | 5.854198 | -3.109880 | -0.228957 |
| H | -5.291361 | -3.800578 | 0.300845 |
| H | -3.947211 | -4.947511 | 0.407218 |
| H | -4.417811 | -4.023208 | 1.827307 |
| H | -2.723556 | 3.747094 | 1.451398 |
| H | -1.269446 | 3.548849 | 2.465007 |
| H | -1.379944 | 4.910803 | 1.324742 |


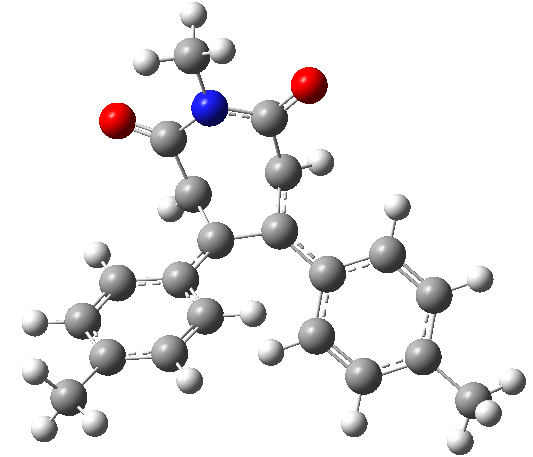


| C | 0.808062 | 0.716338 | -0.201737 |
| --- | --- | --- | --- |
| C | -0.553353 | 0.346186 | -0.539155 |
| C | -0.940459 | 1.517925 | -1.363496 |
| C | 0.830386 | 2.120226 | -0.435549 |
| C | 1.988805 | -0.099879 | -0.135572 |
| C | -1.370202 | -0.756855 | -0.219531 |
| C | 3.197690 | 0.449239 | 0.333544 |
| C | 4.351968 | -0.307614 | 0.373285 |
| C | 4.358511 | -1.641052 | -0.046266 |
| C | 3.162766 | -2.185817 | -0.523192 |
| C | 2.001073 | -1.440320 | -0.566120 |
| C | -2.637986 | -0.909918 | -0.839955 |
| C | -3.447233 | -1.979763 | -0.539823 |
| C | -3.063816 | -2.943164 | 0.407095 |
| C | -1.826112 | -2.787633 | 1.039488 |
| C | -0.993610 | -1.728808 | 0.741143 |
| C | 5.600048 | -2.467126 | 0.035051 |
| C | -3.969442 | -4.084049 | 0.731063 |
| C | -1.812658 | 2.528366 | -0.756151 |
| N | -1.253737 | 3.098792 | 0.384979 |
| C | 0.119655 | 3.067196 | 0.477078 |
| O | 0.742430 | 3.766645 | 1.251897 |
| O | -2.905176 | 2.853575 | -1.180795 |
| C | -2.021517 | 4.008716 | 1.213965 |
| H | -1.116086 | 1.368637 | -2.432485 |
| H | 1.769693 | 2.567049 | -0.773902 |
| H | 3.211959 | 1.473636 | 0.696486 |
| H | 5.271792 | 0.134436 | 0.749546 |
| H | 3.153146 | -3.215245 | -0.873777 |
| H | 1.094937 | -1.882319 | -0.969369 |
| H | -2.966197 | -0.169196 | -1.565114 |
| H | -4.410937 | -2.082918 | -1.034314 |
| H | -1.519653 | -3.515182 | 1.787600 |
| H | -0.048016 | -1.626926 | 1.265069 |
| H | 5.599706 | -3.263274 | -0.715236 |
| H | 6.498017 | -1.857647 | -0.104502 |
| H | 5.686511 | -2.947109 | 1.018171 |
| H | -4.923421 | -3.726914 | 1.137428 |
| H | -4.209359 | -4.666234 | -0.166554 |
| H | -3.520770 | -4.758792 | 1.464948 |
| H | -3.063365 | 3.688935 | 1.214840 |
| H | -1.627916 | 3.982882 | 2.230574 |
| H | -1.963770 | 5.034459 | 0.836368 |


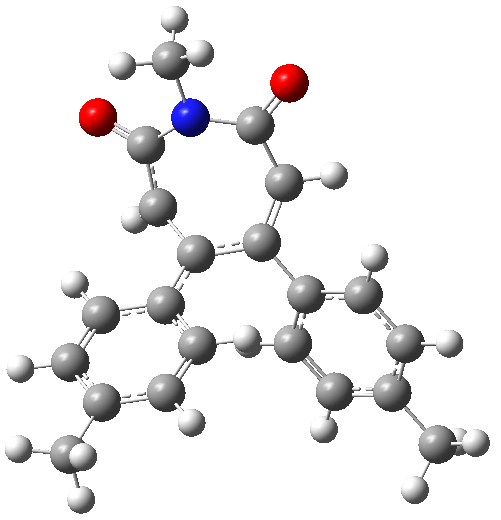


| C | 0.739499 | 0.827236 | -0.175030 |
| --- | --- | --- | --- |
| C | -0.588091 | 0.418914 | -0.534301 |
| C | -1.302174 | 1.386637 | -1.368667 |
| C | 1.009809 | 2.142881 | 0.091753 |
| C | 1.853519 | -0.145044 | -0.145797 |
| C | -1.230232 | -0.809200 | -0.168400 |
| C | 2.832179 | -0.093146 | 0.846785 |
| C | 3.884258 | -0.997906 | 0.850764 |
| C | 3.998876 | -1.972078 | -0.137990 |
| C | 3.016951 | -2.020569 | -1.130569 |
| C | 1.959810 | -1.128595 | -1.133681 |
| C | -2.475092 | -1.153772 | -0.737997 |
| C | -3.142132 | -2.304091 | -0.362942 |
| C | -2.612002 | -3.166385 | 0.597988 |
| C | -1.384319 | -2.823035 | 1.180903 |
| C | -0.707517 | -1.680749 | 0.815689 |
| C | 5.141164 | -2.936993 | -0.152371 |
| C | -3.319075 | -4.419612 | 0.994958 |
| C | -1.866361 | 2.624944 | -0.884591 |
| N | -1.272557 | 3.223489 | 0.234153 |
| C | 0.110097 | 3.229344 | 0.487362 |
| O | 0.580359 | 4.166354 | 1.113723 |
| O | -2.849146 | 3.124113 | -1.411050 |
| C | -2.029712 | 4.311413 | 0.846352 |
| H | -1.479327 | 1.170412 | -2.421836 |
| H | 2.045348 | 2.431039 | 0.253753 |
| H | 2.754431 | 0.647166 | 1.638678 |
| H | 4.630222 | -0.950782 | 1.640902 |
| H | 3.090953 | -2.773276 | -1.912671 |
| H | 1.208115 | -1.182372 | -1.917450 |
| H | -2.918426 | -0.502653 | -1.487105 |
| H | -4.098550 | -2.542698 | -0.822056 |
| H | -0.966556 | -3.470775 | 1.948600 |
| H | 0.227420 | -1.437427 | 1.310777 |
| H | 5.824526 | -2.723489 | -0.982738 |
| H | 5.718356 | -2.888371 | 0.774981 |
| H | 4.790863 | -3.966295 | -0.285044 |
| H | -4.355342 | -4.423466 | 0.646119 |
| H | -2.822301 | -5.301551 | 0.571592 |
| H | -3.319300 | -4.549396 | 2.082472 |
| H | -3.086435 | 4.047974 | 0.836525 |
| H | -1.690220 | 4.442139 | 1.872867 |
| H | -1.894023 | 5.255082 | 0.308240 |
